# Supplementary material for: Construction of boron-stereogenic compounds via enantioselective Cu-catalyzed desymmetric B–H bond insertion reaction
Source: Nat Commun. 2022 May 12;13:2624. doi: 10.1038/s41467-022-30287-7 (PMC9098526; doi:10.1038/s41467-022-30287-7)
Supplement: Supplementary file 1 — Supplementary information [file 41467_2022_30287_MOESM1_ESM.pdf]

Supplementary Information

Construction of Boron-Stereogenic Compounds via Enantioselective Cu-Catalyzed Desymmetric B–H Bond Insertion Reaction

Guan Zhang,[1†] Zhihan Zhang,[2†] Mengyuan Hou,[1] Xinping Cai, [1] Kai Yang, [1] Peiyuan Yu*[2] and Qiuling Song*[1,2,3,4]

[1]Key Laboratory of Molecule Synthesis and Function Discovery, Fujian Province University, College of Chemistry at Fuzhou University, Fuzhou, Fujian, 350108 (China)

[2]Department of Chemistry and Shenzhen Grubbs Institute, Guangdong Provincial Key Laboratory of Catalysis, Southern University of Science and Technology, Shenzhen, Guangdong, 518055 (China)

[3]Institute of Next Generation Matter Transformation, College of Materials Science Engineering at Huaqiao University 668 Jimei Boulevard, Xiamen, Fujian, 361021 (China)

[4]School of Chemistry and Chemical Engineering, Henan Normal University, Xinxiang, Henan, 453007

[†]These two are contributed equally to this work.

*E-mail: qsong@hqu.edu.cn; yupy@sustech.edu.cn

Table of Contents

1. Supplementary Notes 1

2. Supplementary Methods 1

2.1. General procedure for preparation of 2-arylpyridine-boranes [1] 1

2.2. General procedure for preparation of diaryl diazomethanes (Taking **2a** for example): 14

2.3. General procedure for preparation of α-diazoarylacetates. 18

2.4. Reaction optimization 25

2.5. General procedure for boron-stereogenic compounds 27

2.6. Gram-scale reaction and transformations. 63

2.7. Isotope labeling experiments. 67

2.8. Studies of the configurational stability 68

2.9. Crystal structure of compound **3n** and **5a** 69

3. Supplementary Discussion 72

3.1. Computational details 72

3.2. Additional computational results 73

4. Supplementary Figures 79

4.1. NMR spectra 79

4.2. Chiral HPLC charts 222

5. Supplementary References 275

1. Supplementary Notes

All experiments were conducted with a schlenk tube. Flash column chromatography was performed over silica gel (200-300 mesh). ^{1}H NMR and ^{13}C NMR spectra were recorded at ambient temperature using Bruker AVANCE III 500M spectrometers, Bruker 400M spectrometers, *J*EOL 500M spectrometers and *J*EOL 600M spectrometers, chemical shifts (in ppm) were referenced to $CDCl_3$ (δ = 7.26 ppm), acetone-d_6 (δ = 2.05 ppm) as internal standards. ^{13}C NMR spectra were obtained by using the same NMR spectrometers and were calibrated with $CDCl_3$ (δ = 77.0 ppm), acetone-d_6 (δ = 206.68 ppm). Data for 1H NMR are recorded as following abbreviations: multiplicity (s = singlet, d = doublet, t = triplet, q = quarter, m = multiplet), coupling constant (*J*, Hz). High resolution mass spectroscopy (HRMS) analyses were performed at an Exactive Plus (Thermo Scientific) or Agilent Mass Spectrometer. Unless otherwise noted, materials obtained from commercial suppliers were used without further purification.

2. Supplementary Methods

2.1. General procedure for preparation of 2-arylpyridine-boranes [1]

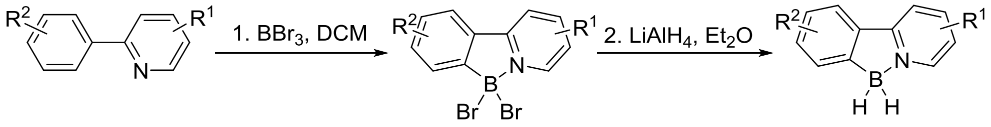

Supplementary Figure 1. Synthetic methods for 2-arylpyridine-boranes

Step 1: To a stirred solution of 2-phenylpyridine derivative (5 mmol) and *i*-Pr_2NEt (870 mg, 5 mmol) in DCM (10 mL) at 0 °C was added BBr_3 (15 mL, 1.0 M in DCM, 15 mmol). Caution: BBr_3 is highly moisture sensitive and decomposes in air with an evolution of corrosive HBr. It should be handled in a well-ventilated hood. After being stirred at room temperature for 12 h, saturated K_2CO_3 aqueous solution was added to the reaction mixture. Then filtered, filter residue is product, dried the filter residue.

Step 2: The filter residue from step 1 was dissolved in Et_2O (20 mL), added $LiAlH_4$ (569 mg, 3 equiv) at 0 °C, the reaction was allowed to stir at 0 ℃ for 10 hours. Then, this reaction was quenched by H_2SO_4 (0.5 M), extracted with ethyl acetate (20 mL × 3) and dried over Na_2SO_4. After removal of the solvent, the crude reaction mixture was purified on short column chromatography (petroleum ether and

ethyl acetate) to afford the crude products.

7-chloro-6H-5λ⁴-benzo[3,4][1,2]azaborolo[1,5-a]pyridine(1a)

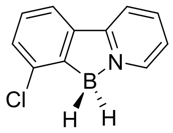

1a

Following the above procedure on 10 mmol scale, white solid (mp: 136.2 – 137.3 ^{o}C), yield: 35% (704 mg), R_f = 0.4 (silica gel, PE: DCM = 2:1, v/v), column chromatography (silica gel, PE: DCM = 2:1, v/v).

^{1}H NMR (500 MHz, $(CD_3)_2CO$) δ 8.79 (dd, *J* = 5.7, 0.8 Hz, 1H), 8.33 – 8.28 (m, 1H), 8.28 – 8.21 (m, 1H), 8.00 – 7.98 (m, 1H), 7.64 – 7.61(m, 1H), 7.40 (dd, *J* = 7.8, 0.8 Hz, 1H), 7.33 (dd, *J* = 11.8, 4.1 Hz, 1H), 3.77 – 3.18 (br, 2H).

^{13}C NMR (126 MHz, $CDCl_3$) δ 157.5, 144.0, 139.4, 138.1, 136.7, 130.2, 127.1, 121.5, 119.8, 118.4.

^{11}B NMR (160 MHz, $(CD_3)_2CO$) δ -9.23 (t, *J* = 103.1 Hz).

HRMS (EI) m/z: $[M-H]^-$ Calcd. for $C_{11}H_9BClN$ 200.0433; Found: 200.0405.

6H-5λ⁴-benzo[3,4][1,2]azaborolo[1,5-a]pyridine(1b)

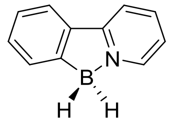

1b

Following the above procedure on 10 mmol scale, white solid (mp: 81.0 – 91.5 ^{o}C), yield: 60% (1002 mg), R_f = 0.4 (silica gel, PE: DCM = 2:1, v/v), column chromatography (silica gel, PE: DCM = 2:1, v/v). Known compound.[1]

^{1}H NMR (500 MHz, $(CD_3)_2CO$) δ 8.75 (d, *J* = 5.7 Hz, 1H), 8.26 (d, *J* = 8.1 Hz, 1H), 8.19 (t, *J* = 7.7 Hz, 1H), 8.03 – 8.01(m, 1H), 7.66 (d, *J* = 7.3 Hz, 1H), 7.56 – 7.53 (m, 1H), 7.41 – 7.8 (m, 1H), 7.31 – 7.28 (m, 1H), 3.76 – 3.18 (br, 2H).

^{13}C NMR (126 MHz, $(CD_3)_2CO$) δ 144.7, 140.7, 137.5, 130.3, 130.3, 125.5, 122.4, 122.2, 118.9.

^{11}B NMR (160 MHz, $(CD_3)_2CO$) δ-9.05 (t, *J* = 96.6 Hz).

HRMS (EI) m/z: $[M-H]^-$ Calcd. for $C_{11}H_{10}BN$ 166.0822; Found: 166.0780.

7-chloro-6H-5λ[4]-benzo[3,4][1,2]azaborolo[1,5-a]pyridine(1c)

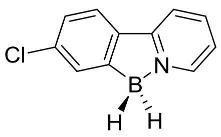

1c

Following the above procedure on 10 mmol scale, white solid (mp: 96.3 – 97.2 °C), yield: 32% (643 mg), R_f = 0.4 (silica gel, PE: DCM = 2:1, v/v), column chromatography (silica gel, PE: DCM = 2:1, v/v).

^{1}H NMR (500 MHz, $CDCl_3$) δ 8.60 (d, *J* = 5.7 Hz, 1H), 7.96 (t, *J* = 7.7 Hz, 1H), 7.91 (d, *J* = 8.1 Hz, 1H), 7.77 – 7.71 (m, 2H), 7.34 – 7.14 (m, 1H), 7.29 – 7.25 (m, 1H), 3.66 – 3.12 (br, 2H).

^{13}C NMR (126 MHz, $CDCl_3$) δ 157.5, 144.0, 139.3, 136.9, 134.9, 130.1, 125.6, 122.6, 121.0, 118.0.

^{11}B NMR (160 MHz, $CDCl_3$) δ -8.67 (s), -9.28 (s), -9.86 (s).

HRMS (ESI) m/z: $[M+Na]^+$ Calcd. for $C_{11}H_9BClN$ 224.0409; Found: 224.0405.

9-chloro-6H-5λ[4]-benzo[3,4][1,2]azaborolo[1,5-a]pyridine(1d)

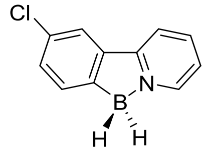

1d

Following the above procedure on 10 mmol scale, white solid (mp: 158.2 – 159.3 °C), yield: 35% (703 mg), R_f = 0.4 (silica gel, PE: DCM = 2:1, v/v), column chromatography (silica gel, PE: DCM = 2:1, v/v).

^{1}H NMR (500 MHz, $(CD_3)_2CO$) δ 8.75 – 8.71 (m, 1H), 8.31 – 8.28 (m, 1H), 8.21 – 8.16 (m, 1H), 8.03 – 8.00 (m, 1H), 7.60 – 7.53 (m, 2H), 7.34 (dd, *J* = 7.9, 1.9 Hz, 1H), 3.66 – 3.09 (br, 2H).

^{13}C NMR (126 MHz, $CDCl_3$) δ 157.2, 144.1, 139.4, 138.1, 131.4, 131.2, 130.2, 121.4, 121.3, 118.1.

^{11}B NMR (160 MHz, $(CD_3)_2CO$) δ -9.14 (t, *J* = 98.4 Hz).

HRMS (EI) m/z: $[M-H]^-$ Calcd. for $C_{11}H_9BClN$ 200.0433; Found: 200.0405.

10-fluoro-6H-5λ[4]-benzo[3,4][1,2]azaborolo[1,5-a]pyridine (1e)

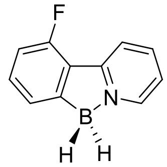

1e

Following the above procedure on 5 mmol scale, white solid (mp: 109.3 – 110.2 ^{o}C), yield: 50% (463 mg), R_f = 0.6 (silica gel, PE: DCM = 2:1, v/v), column chromatography (silica gel, PE: DCM = 2:1, v/v).

^{1}H NMR (500 MHz, $CDCl_3$) δ 8.65 (d, *J* = 5.7 Hz, 1H), 8.22 (d, *J* = 8.2 Hz, 1H), 7.98 (t, *J* = 7.8 Hz, 1H), 7.53 (d, *J* = 7.2 Hz, 1H), 7.43 – 7.39 (m, 1H), 7.37 – 7.32 (m, 1H), 6.96 (dd, *J* = 11.1, 8.1 Hz, 1H), 3.88 – 3.06 (br, 2H).

^{13}C NMR (126 MHz, $CDCl_3$) δ 161.1(d, *J* = 253.9 Hz), 155.41 (d, *J* = 4.5 Hz), 143.8, 139.5, 131.80 (d, *J* = 6.8 Hz), 125.51 (d, *J* = 3.2 Hz), 124.00 (d, *J* = 7.6 Hz), 121.97 (d, *J* = 10.2 Hz), 120.9, 111.58 (d, *J* = 19.6 Hz).

^{11}B NMR (160 MHz, $CDCl_3$) δ -8.82 (t, *J* = 94.9 Hz).

^{19}F NMR (376 MHz, $CDCl_3$) δ -117.91 (s).

HRMS (ESI) m/z: $[M+Na]^+$ Calcd. for $C_{11}H_9BFN$ 208.0704; Found: 208.0698.

4-methyl-6H-5λ^4-benzo[3,4][1,2]azaborolo[1,5-a]pyridine (1f)

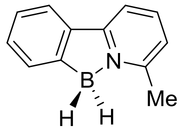

1f

Following the above procedure on 5 mmol scale, white solid (mp: 109.3 – 110.2 ^{o}C), yield: 52% (471 mg), R_f = 0.5 (silica gel, PE: DCM = 2:1, v/v), column chromatography (silica gel, PE: DCM = 2:1, v/v).

^{1}H NMR (500 MHz, $CDCl_3$) δ 7.88 – 7.84 (m, 2H), 7.81 (dd, *J* = 14.6, 7.3 Hz, 2H), 7.49 – 7.46 (m, 1H), 7.34 (t, *J* = 7.6 Hz, 1H), 7.14 (d, *J* = 7.2 Hz, 1H), 3.58 – 3.04 (br, 2H), 2.74 (s, 3H).

^{13}C NMR (126 MHz, $CDCl_3$) δ 158.0, 154.7, 139.0, 137.0, 130.0, 129.8, , 125.0, 121.5, 121.5, 114.9, 21.5.

^{11}B NMR (160 MHz, $CDCl_3$) δ -9.64 (t, *J* = 96.7 Hz).

HRMS (EI) m/z: [M-H]$^-$ Calcd. for $C_{12}H_{12}BN$ 180.0979; Found: 180.0972.

3-methyl-6H-5λ^4-benzo[3,4][1,2]azaborolo[1,5-a]pyridine (1g)

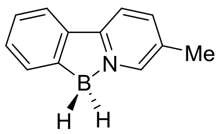

1g

Following the above procedure on 5 mmol scale, white solid (mp: 136.7 – 138.1 ^oC), yield: 52% (471 mg), R_f = 0.5 (silica gel, PE: DCM = 2:1, v/v), column chromatography (silica gel, PE: DCM = 2:1, v/v).

^{1}H NMR (500 MHz, $CDCl_3$) δ 8.45 (s, 1H), 7.84 – 7.79 (m, 2H), 7.79 – 7.71 (m, 2H), 7.44 – 7.41 (m, 1H), 7.30 (t, *J* = 7.4 Hz, 1H), 3.68 – 3.16 (br, 2H), 2.41 (s, 3H).

^{13}C NMR (126 MHz, $CDCl_3$) δ 156.0, 143.5, 140.2, 136.7, 131.0, 130.1, 129.7, 125.0, 121.1, 117.3, 18.0.

^{11}B NMR (160 MHz, $CDCl_3$) δ -9.14 (t, *J* = 93.6 Hz).

HRMS (EI) m/z: [M-H]$^-$ Calcd. for $C_{12}H_{12}BN$ 180.0979; Found: 180.0981.

2-methyl-6H-5λ^4-benzo[3,4][1,2]azaborolo[1,5-a]pyridine (1h)

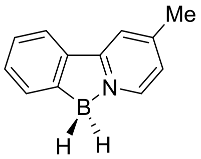

1h

Following the above procedure on 5 mmol scale, white solid (mp: 105.4 – 106.2 ^oC), yield: 56% (507 mg), R_f = 0.5 (silica gel, PE: DCM = 2:1, v/v), column chromatography (silica gel, PE: DCM = 2:1, v/v).

^{1}H NMR (500 MHz, $CDCl_3$) δ 8.36 (d, *J* = 5.9 Hz, 1H), 7.79 (dd, *J* = 17.4, 7.5 Hz, 2H), 7.62 (s, 1H), 7.48 – 7.42 (m, 1H), 7.30 (dd, *J* = 9.6, 5.3 Hz, 1H), 6.98 (dd, *J* = 5.9, 1.0 Hz, 1H), 3.74 – 3.19 (br, 2H), 2.42 (s, 3H).

^{13}C NMR (126 MHz, $CDCl_3$) δ 157.8, 151.4, 142.8, 136.6, 130.0, 129.8, 124.9, 122.1, 121.3, 118.0, 21.5.

^{11}B NMR (160 MHz, $CDCl_3$) δ -8.46 (t, *J* = 80.5 Hz).

HRMS (EI) m/z: $[M\text{-}H]^{-}$ Calcd. for $C_{12}H_{12}BN$ 180.0979; Found: 180.0973.

7-fluoro-6H-5λ^{4}-benzo[3,4][1,2]azaborolo[1,5-a]pyridine(1i)

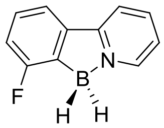

1i

Following the above procedure on 5 mmol scale, white solid (mp: 138.5 – 139.1 ^{o}C), yield: 35% (323 mg), R_f = 0.3 (silica gel, PE: DCM = 5:1, v/v), column chromatography (silica gel, PE: DCM = 10:1, v/v).

^{1}H NMR (400 MHz, $CDCl_3$) δ 8.64 (d, *J* = 5.6 Hz, 1H), 8.02 – 7.93 (m, 2H), 7.66 (d, *J* = 7.5 Hz, 1H), 7.37 (t, *J* = 6.2 Hz, 1H), 7.30 (dd, *J* = 13.0, 7.6 Hz, 1H), 7.09 (t, *J* = 7.9 Hz, 1H), 4.02 – 2.99 (br, 2H).

^{13}C NMR (101 MHz, $CDCl_3$) δ 164.0 (d, *J* = 241.1Hz), 157.4, 144.1, 139.7 (d, *J* = 14.5 Hz), 139. 5, 127.5 (d, *J* = 6.9 Hz), 121.6, 118.4, 117.7 (d, *J* = 3.2 Hz), 116.6 (d, *J* = 24.5 Hz).

^{11}B NMR (128 MHz, $CDCl_3$) δ -9.16 (t, *J* = 100.7 Hz).

^{19}F NMR (376 MHz, $CDCl_3$) δ -106.37 (s).

HRMS (ESI) m/z: $[M+Na]^{+}$ Calcd. for $C_{11}H_9BFN$ 208.0704; Found: 208.0706.

7-bromo-6H-5λ^{4}-benzo[3,4][1,2]azaborolo[1,5-a]pyridine (1j)

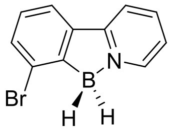

1j

Following the above procedure on 10 mmol scale, white solid (mp: 144.8 – 146.2 ^{o}C), yield: 30% (367.5 mg), R_f = 0.4 (silica gel, PE: DCM = 2:1, v/v), column chromatography (silica gel, PE: DCM = 2:1, v/v).

^{1}H NMR (400 MHz, $CDCl_3$) δ 8.57 (d, *J* = 5.5 Hz, 1H), 7.94 (t, *J* = 7.7 Hz, 1H), 7.86 (d, *J* = 8.1 Hz, 1H), 7.73 (d, *J* = 7.6 Hz, 1H), 7.54 (d, *J* = 7.8 Hz, 1H), 7.32 (t, *J* = 6.5 Hz, 1H), 7.15 (t, *J* = 7.7 Hz, 1H), 3.71 – 3.05 (br, 2H).

^{13}C NMR (101 MHz, $CDCl_3$) δ 157.3, 143.8, 139.4, 137.8, 133.2, 127.2, 125.7, 121.5, 120.3, 118.4.

^{11}B NMR (128 MHz, $CDCl_3$) δ -7.60 (t, *J* = 91.0 Hz).

HRMS (EI) m/z: $[M+Na]^{+}$ Calcd. for $C_{11}H_9BBrN$ 267.9904; Found: 267.9904.

7-chloro-10-fluoro-6H-5λ^{4}-benzo[3,4][1,2]azaborolo[1,5-a]pyridine(1k)

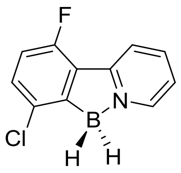

1k

Following the above procedure on 5 mmol scale, white solid (mp: 149.2 – 151.2 °C), yield: 40% (438 mg), R_f = 0.5 (silica gel, PE: DCM = 2:1, v/v), column chromatography (silica gel, PE: DCM = 10:1, v/v).

^{1}H NMR (500 MHz, $CDCl_3$) δ 8.66 (d, *J* = 5.7 Hz, 1H), 8.18 (d, *J* = 8.2 Hz, 1H), 8.02 (t, *J* = 7.8 Hz, 1H), 7.42 – 7.39 (m, 1H), 7.34 (dd, *J* = 8.5, 4.0 Hz, 1H), 6.92 (dd, *J* = 10.0, 8.6 Hz, 1H), 3.70 – 3.17 (br, 2H).

^{13}C NMR (126 MHz, $CDCl_3$) δ 158.7 (d, *J* = 253.5 Hz), 154.6 (d, *J* = 5.4 Hz), 144.0, 139.9, 131. 6 (d, *J* = 7.7 Hz), 130.8 (d, *J* = 3.0 Hz), 124.8 (d, *J* = 9.4 Hz), 122.2 (d, *J* = 10.3 Hz), 121.7, 113.9 (d, *J* = 22 Hz), 99.9.

^{11}B NMR (128 MHz, $CDCl_3$) δ -8.08 (t, *J* = 99.6 Hz).

^{19}F NMR (376 MHz, $CDCl_3$) δ -120.99 (s).

HRMS (EI) m/z: $[M+H]^+$ Calcd. for $C_{11}H_8BFClN$ 220.0495; Found: 220.0489.

7-chloro-8-fluoro-6H-5λ^4-benzo[3,4][1,2]azaborolo[1,5-a]pyridine(1l)

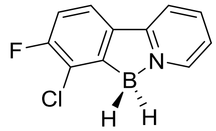

1l

Following the above procedure on 5 mmol scale, white solid (mp: 168.0 – 169.0 °C), yield: 25% (273.8 mg), R_f = 0.5 (silica gel, PE: DCM = 2:1, v/v), column chromatography (silica gel, PE: DCM = 10:1, v/v).

^{1}H NMR (400 MHz, $CDCl_3$) δ 8.58 (d, *J* = 5.4 Hz, 1H), 7.97 (t, *J* = 7.7 Hz, 1H), 7.86 (d, *J* = 8.1 Hz, 1H), 7.67 (dd, *J* = 8.2, 3.8 Hz, 1H), 7.34 (t, *J* = 6.4 Hz, 1H), 7.05 (t, *J* = 8.7 Hz, 1H), 3.72 – 3.07 (br, 2H).

^{13}C NMR (101 MHz, $CDCl_3$) δ 159.5 (d, *J* = 252.5 Hz), 156.9, 144.0, 139.6, 133.1 (d, *J* = 2.9 Hz), 121.4 (d, *J* = 8.3 Hz), 121.2, 118.1, 114.3 (d, *J* = 24.0 Hz), 99.9.

^{11}B NMR (128 MHz, CDCl$_3$) δ -8.43 (t, *J* = 101.8 Hz).

^{19}F NMR (376 MHz, CDCl$_3$) δ -112.98 (s).

HRMS (EI) m/z: $[M+H]^+$ Calcd. for $C_{11}H_8BFClN$ 220.0495; Found: 220.0489.

7,8-dichloro-6H-5λ^4-benzo[3,4][1,2]azaborolo[1,5-a]pyridine(1m)

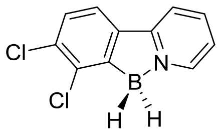

1m

Following the above procedure on 5 mmol scale, white solid (mp: 149.3 – 151.4 °C), yield: 30% (352.5 mg), R_f = 0.5 (silica gel, PE: DCM = 2:1, v/v), column chromatography (silica gel, PE: DCM = 10:1, v/v).

^{1}H NMR (400 MHz, CDCl$_3$) δ 8.59 (d, *J* = 5.5 Hz, 1H), 7.98 (t, *J* = 7.7 Hz, 1H), 7.87 (d, *J* = 8.1 Hz, 1H), 7.60 (d, *J* = 8.1 Hz, 1H), 7.36 (dd, *J* = 12.6, 7.1 Hz, 2H), 3.67 – 3.04 (br, 2H).

^{13}C NMR (101 MHz, CDCl$_3$) δ 156.7, 143.9, 139.6, 135.8, 134.7, 134.1, 128.0, 121.6, 120.6, 118.4.

^{11}B NMR (128 MHz, CDCl$_3$) δ -8.25 (t, *J* = 101.12Hz).

HRMS (EI) m/z: $[M+H]^+$ Calcd. for $C_{11}H_8BCl_2N$ 236.0200; Found: 236.0207.

7,9-dichloro-6H-5λ^4-benzo[3,4][1,2]azaborolo[1,5-a]pyridine (1n)

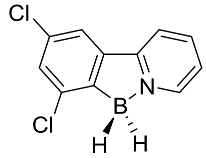

1n

Following the above procedure on 5 mmol scale, white solid (mp: 184.1 – 185.6 °C), yield: 45% (528 mg), R_f = 0.5 (silica gel, PE: DCM = 2:1, v/v), column chromatography (silica gel, PE: DCM = 2:1, v/v).

^{1}H NMR (500 MHz, CDCl$_3$) δ 8.65 (d, *J* = 4.6 Hz, 1H), 8.02 (t, *J* = 7.5 Hz, 1H), 7.90 (d, *J* = 7.7 Hz, 1H), 7.68 (s, 1H), 7.41 (dd, *J* = 14.5, 7.5 Hz, 2H), 3.64 – 3.14 (br, 2H).

^{13}C NMR (126 MHz, CDCl$_3$) δ 156.4, 144.3, 139.7, 138.8, 137.2, 132.0, 130.0, 122.2, 120.0, 118.6.

^{11}B NMR (160 MHz, CDCl$_3$) δ -8.39 (d, *J* = 96.3 Hz).

HRMS (EI) m/z: $[M-H]^-$ Calcd. for $C_{11}H_8BClN$ 234.0043; Found: 234.0040.

7-chloro-10-methyl-6H-5λ⁴-benzo[3,4][1,2]azaborolo[1,5-a]pyridine(1o)

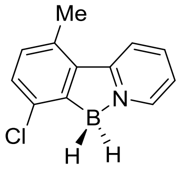

1o

Following the above procedure on 5 mmol scale, white solid (mp: 166.9 – 168.8 ^oC), yield: 36% (387 mg), R_f = 0.5 (silica gel, PE: DCM = 2:1, v/v), column chromatography (silica gel, PE: DCM = 10:1, v/v).

^{1}H NMR (400 MHz, $CDCl_3$) δ 8.62 (d, *J* = 5.4 Hz, 1H), 8.04 (d, *J* = 8.3 Hz, 1H), 7.94 (t, *J* = 7.8 Hz, 1H), 7.32 (t, *J* = 6.5 Hz, 1H), 7.23 (d, *J* = 7.9 Hz, 1H), 6.98 (d, *J* = 7.9 Hz, 1H), 3.73 – 3.05 (br, 2H), 2.61 (s, 3H).

^{13}C NMR (101 MHz, $CDCl_3$) δ 158.0, 144.3, 139.3, 136.3, 133.7, 132.9, 130.1, 129.6, 121.6, 120.8, 21.5.

^{11}B NMR (128 MHz, $CDCl_3$) δ -8.69 (t, *J* = 94.0 Hz).

HRMS (EI) m/z: $[M+Na]^+$ Calcd. for $C_{12}H_{11}BClN$ 238.0565; Found: 238.0560.

12H-11λ⁴-naphtho[1',2':3,4][1,2]azaborolo[1,5-a]pyridine (1p)

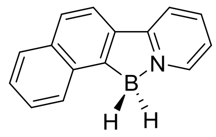

1p

Following the above procedure on 5 mmol scale, white solid (mp: 158.9 – 159.8 ^oC), yield: 35% (543 mg), R_f = 0.4 (silica gel, PE: DCM = 2:1, v/v), column chromatography (silica gel, PE: DCM = 2:1, v/v).

^{1}H NMR (500 MHz, $CDCl_3$) δ 8.65 (d, *J* = 5.7 Hz, 1H), 8.22 (dd, *J* = 5.2, 4.3 Hz, 1H), 7.92 – 7.85 (m, 3H), 7.83 (d, *J* = 8.4 Hz, 1H), 7.76 (d, *J* = 8.4 Hz, 1H), 7.59 – 7.51 (m, 2H), 7.25 – 7.20 (m, 1H), 4.03 – 3.48 (br, 2H).

^{13}C NMR (126 MHz, $CDCl_3$) δ 159.2, 144.2, 139.3, 134.9, 134.1, 133.3, 130.4, 128.4, 127.3, 126.6,

126.1, 120.2, 118.8, 117.8.

^{11}B NMR (160 MHz, CDCl$_3$) δ -9.10 (t, *J* = 92.0 Hz).

HRMS (ESI) m/z: $[M+K]^+$ Calcd. for $C_{15}H_{12}BN$ 256.0694; Found: 256.0690.

8-fluoro-6H-5λ^4-benzo[3,4][1,2]azaborolo[1,5-a]pyridine(1q)

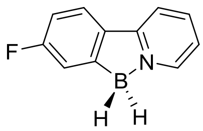

1q

Following the above procedure on 10 mmol scale, white solid (mp: 108.4 – 109.3 °C), yield: 30% (555 mg), R_f = 0.5 (silica gel, PE: DCM = 2:1, v/v), column chromatography (silica gel, PE: DCM = 2:1, v/v).

^{1}H NMR (500 MHz, CDCl$_3$) δ 8.59 (d, *J* = 5.7 Hz, 1H), 7.94 (t, *J* = 7.7 Hz, 1H), 7.89 (d, *J* = 8.1 Hz, 1H), 7.81 (dd, *J* = 8.4, 4.8 Hz, 1H), 7.42 (dd, *J* = 8.9, 2.1 Hz, 1H), 7.32 – 7.27 (m, 1H), 7.01 – 6.97 (m, 1H), 3.68 – 3.11 (br, 1H).

^{13}C NMR (126 MHz, CDCl$_3$) δ 157.6 (d, *J* = 2.6 Hz), 144.0, 139.3, 132.6 (d, *J* = 1.7 Hz), 123.3 (d, *J* = 9.1 Hz), 120.5, 117.7, 116.3 (d, *J* = 19.8 Hz), 113.1, 112.9.

^{11}B NMR (160 MHz, CDCl$_3$) δ -8.30 (t, *J* = 98.4 Hz).

^{19}F NMR (471 MHz, CDCl$_3$) δ -110.30 (td, *J* = 9.1, 4.3 Hz).

HRMS (ESI) m/z: $[M+Na]^+$ Calcd. for $C_{11}H_9BFN$ 208.0704; Found:208.0707.

7-chloro-8-methyl-6H-5λ^4-benzo[3,4][1,2]azaborolo[1,5-a]pyridine(1r)

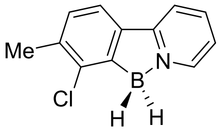

1r

Following the above procedure on 5 mmol scale, white solid (mp: 169.1 – 171.3 °C), yield: 20% (215 mg), R_f = 0.5 (silica gel, PE: DCM = 2:1, v/v), column chromatography (silica gel, PE: DCM = 10:1, v/v).

^{1}H NMR (400 MHz, CDCl$_3$) δ 8.58 (d, *J* = 5.5 Hz, 1H), 7.93 (t, *J* = 7.7 Hz, 1H), 7.86 (d, *J* = 8.1 Hz, 1H), 7.61 (d, *J* = 7.7 Hz, 1H), 7.30 (t, *J* = 6.5 Hz, 1H), 7.17 (d, *J* = 7.7 Hz, 1H), 3.69 – 3.10 (br, 2H),

2.46 (s, 3H).

^{13}C NMR (101 MHz, $CDCl_3$) δ 157.7, 143.9, 139.3, 138.3, 136.7, 135.8, 128.7, 121.0, 119.7, 118.0, 20.5.

^{11}B NMR (128 MHz, $CDCl_3$) δ -8.34 (t, *J* = 99.0 Hz).

HRMS (EI) m/z: $[M+Na]^+$Calcd. for $C_{12}H_{11}BClN$ 238.0565; Found: 238.0563.

7,10-dichloro-6H-5λ^4-benzo[3,4][1,2]azaborolo[1,5-a]pyridine(1s)

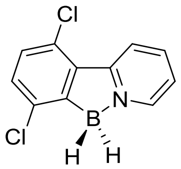

1s

Following the above procedure on 5 mmol scale, white solid (mp: 155.7 – 156.3 ^{o}C), yield: 42% (493.5 mg), R_f = 0.5 (silica gel, PE: DCM = 2:1, v/v), column chromatography (silica gel, PE: DCM = 10:1, v/v).

^{1}H NMR (400 MHz, $CDCl_3$) δ 8.83 (d, *J* = 8.4 Hz, 1H), 8.69 (d, *J* = 5.6 Hz, 1H), 8.03 (t, *J* = 7.9 Hz, 1H), 7.44 (t, *J* = 6.6 Hz, 1H), 7.30 (d, *J* = 8.3 Hz, 1H), 7.22 (d, *J* = 8.3 Hz, 1H), 3.77 – 3.07 (br, 2H).

^{13}C NMR (101 MHz, $CDCl_3$) δ 155.9, 144.2, 139.5, 134.5, 133.8, 130.7, 128.9, 128.6, 122.4, 121.9.

^{11}B NMR (128 MHz, $CDCl_3$) δ -8.51 (t, *J* = 98.7 Hz).

HRMS (EI) m/z: $[M+H]^+$ Calcd. for $C_{11}H_8BCl_2N$ 236.0200; Found: 236.0196.

7-(trifluoromethoxy)-6H-5λ^4-benzo[3,4][1,2]azaborolo[1,5-a]pyridine (**1t**)

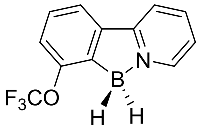

1t

Following the above procedure on 5 mmol scale, white solid (mp: 119.8 – 121.7 ^{o}C), yield: 42% (493.5 mg), R_f = 0.5 (silica gel, PE: DCM = 2:1, v/v), column chromatography (silica gel, PE: DCM = 10:1, v/v).

^{1}H NMR (500 MHz, $CDCl_3$) δ 8.64 (d, *J* = 5.7 Hz, 1H), 7.98 (t, *J* = 8.5 Hz, 2H), 7.79 (d, *J* = 7.4 Hz, 1H), 7.41 – 7.32 (m, 2H), 7.29 (d, *J* = 8.1 Hz, 1H), 3.73 – 3.21 (br, 2H).

^{13}C NMR (126 MHz, $CDCl_3$) δ 157.1 (d, *J* = 1.6 Hz), 150.9 (d, *J* = 2.3 Hz), 144.0, 139.5, 139.3, 127.2,

122.5, 121.7, 121.0 (q, J = 204.1 Hz), 120.3, 118.4.

^{11}B NMR (128 MHz, CDCl$_3$) δ -8.77 (t, J = 100.5 Hz).

^{19}F NMR (376 MHz, CDCl$_3$) δ -56.75 (s).

HRMS (EI) m/z: [M+H]$^+$ Calcd. for $C_{12}H_9BF_3NO$ 252.0802; Found: 252.0797.

7,9-dimethyl-6H-5λ^4-benzo[3,4][1,2]azaborolo[1,5-a]pyridine (1u)

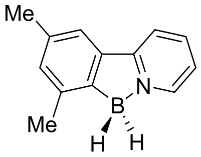

1u

Following the above procedure on 10 mmol scale, white solid (mp: 156.8 – 157.5 °C), yield: 45% (878 mg), R_f = 0.4 (silica gel, PE: DCM = 2:1, v/v), column chromatography (silica gel, PE: DCM = 2:1, v/v).

^{1}H NMR (500 MHz, CDCl$_3$) δ 8.60 (d, J = 5.6 Hz, 1H), 7.89 (d, J = 3.9 Hz, 2H), 7.49 (s, 1H), 7.27 – 7.22 (m, 1H), 7.09 (s, 1H), 3.38 (dd, J = 179.4, 68.8 Hz, 2H), 2.43 (s, 3H), 2.40 (s, 3H).

^{13}C NMR (126 MHz, CDCl$_3$) δ 158.8, 143.9, 139.8, 138.9, 136.5, 135.3, 131.9, 120.3, 119.1, 117.9, 21.4, 21.3.

^{11}B NMR (160 MHz,CDCl$_3$) δ -8.58 (t, J = 85.8 Hz).

HRMS (EI) m/z: [M - H]$^-$ Calcd. for $C_{13}H_{14}BN$ 194.1135; Found: 194.1141.

9H-8λ^4-thieno[2',3':3,4][1,2]azaborolo[1,5-a]pyridine(1v)

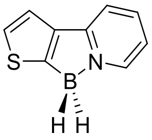

1v

Following the above procedure on 5 mmol scale, white solid (mp: 88.9 – 90.0 °C), yield: 35% (303 mg), R_f = 0.6 (silica gel, PE: DCM = 2:1, v/v), column chromatography (silica gel, PE: DCM = 2:1, v/v).

^{1}H NMR (500 MHz, CDCl$_3$) δ 8.50 (d, J = 5.7 Hz, 1H), 7.88 – 7.83 (m, 1H), 7.62 (d, J = 8.1 Hz, 1H), 7.46 (d, J = 4.8 Hz, 1H), 7.40 (d, J = 4.8 Hz, 1H), 7.15 – 7.12 (m, 1H), 3.65 – 3.07(br, 2H).

^{13}C NMR (126 MHz, CDCl$_3$) δ 154.8, 144.1, 141.2, 139.5, 130.9, 119.3, 118.4, 117.3.

^{11}B NMR (160 MHz, CDCl$_3$) δ -10.01(t, J =109.4 Hz).

HRMS (EI) m/z: [M-H]$^-$ Calcd. for C_9H_8BNS 172.0387; Found: 172.0369.

6H-7λ⁴-benzo[4',5']thieno[2',3':3,4][1,2]azaborolo[1,5-a]pyridine (1w)

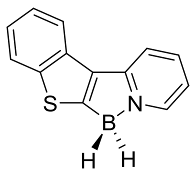

1w

Following the above procedure on 5 mmol scale, white solid (mp: 153.6 – 154.5 °C), yield: 35% (446 mg), R_f = 0.4 (silica gel, PE: DCM = 2:1, v/v), column chromatography (silica gel, PE: DCM = 2:1, v/v).

^{1}H NMR (500 MHz, CDCl$_3$) δ 8.52 (d, *J* = 5.7 Hz, 1H), 8.00 (d, *J* = 8.0 Hz, 1H), 7.91 (dd, *J* = 8.2, 4.4 Hz, 3H), 7.47 – 7.42 (m, 1H), 7.34 – 7.30 (m, 1H), 7.16 – 7.11 (m, 1H), 3.66 – 3.17 (br, 2H).

^{13}C NMR (126 MHz, CDCl$_3$) δ 154.7, 147.0, 144.3, 139.5, 134.2, 133.8, 124.4, 123.2, 123.0, 120.0, 117.9, 117.2.

^{11}B NMR (160 MHz, CDCl$_3$) δ -10.21 (t, *J* = 108.4 Hz).

HRMS (EI) m/z: [M-H]$^-$ Calcd. for $C_{13}H_{10}BNS$ 222.0543; Found: 222.0530.

1-fluoro-6H-5λ⁴-benzo[3,4][1,2]azaborolo[1,5-a]pyridine (1x)

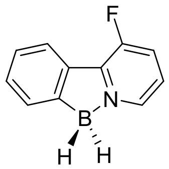

1x

Following the above procedure on 5 mmol scale, white solid (mp: 130.7 – 131.5°C), yield: 30% (278 mg), R_f = 0.6 (silica gel, PE: DCM = 2:1, v/v), column chromatography (silica gel, PE: DCM = 2:1, v/v).

^{1}H NMR (400 MHz, CDCl$_3$) δ 8.49 (d, *J* = 5.3 Hz, 1H), 8.13 (d, *J* = 7.8 Hz, 1H), 7.79 (d, *J* = 7.3 Hz, 1H), 7.68 (t, *J* = 9.0 Hz, 1H), 7.47 (t, *J* = 7.3 Hz, 1H), 7.34 (t, *J* = 7.6 Hz, 1H), 7.32 – 7.25 (m, 1H), 3.84 – 3.15 (br, 2H).

^{13}C NMR (126 MHz, CDCl$_3$) δ 156.7 (d, *J* = 254.9 Hz), 148.7, 140.2 (d, *J* = 4.4 Hz), 134.5 (d, *J* = 4.8 Hz), 130.4, 129.7, 125.5, 125.4, 125.3 (d, *J* = 10.5 Hz), 120.9 (d, *J* = 5.8 Hz).

^{11}B NMR (128 MHz, $CDCl_3$) δ -7.31 (t, *J* = 99.5 Hz).

^{19}F NMR (376 MHz, $CDCl_3$) δ -122.94 (s).

HRMS (EI) m/z: $[M-H]^-$ Calcd. for $C_{11}H_9BFN$ 184.0728; Found: 184.0717.

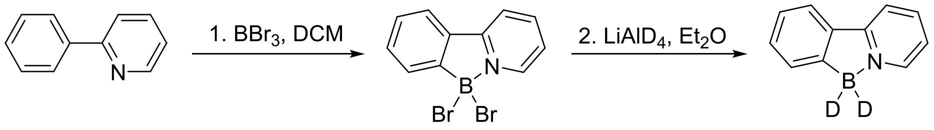

Supplementary Figure 2. Synthetic methods for 2-arylpyridine-borane (1b-*d*$_2$)

Step 1: To a stirred solution of 2-phenylpyridine (776 mg, 5 mmol) and *i*-Pr_2NEt (870 mg, 5 mmol) in DCM (10 mL) at 0 °C was added BBr_3 (15 mL, 1.0 M in DCM, 15 mmol). Caution: BBr_3 is highly moisture sensitive and decomposes in air with an evolution of corrosive HBr. It should be handled in a well-ventilated hood. After being stirred at room temperature for 12 h, saturated K_2CO_3 aqueous solution was added to the reaction mixture. Then filtered, filter residue is product, dried the filter residue.

Step 2: The filter residue from step 1 was dissolved in Et_2O (20 mL), added $LiAlD_4$ (630 mg, 3 equiv) at 0 °C, the reaction was allowed to stir at 0 ℃ for 10 hours. Then, this reaction was quenched by H_2SO_4 (0.5 M), extracted with ethyl acetate (20 mL × 3) and dried over Na_2SO_4. After removal of the solvent, the crude reaction mixture was purified on short column chromatography (petroleum ether and ethyl acetate) to afford the products (**10-*d*$_2$**). **^{1}H NMR (600 MHz, $CDCl_3$)** δ 8.63 – 8.62 (m, 1H), 7.97 – 7.92 (m, 2H), 7.87 – 7.86(m, 1H), 7.80 (d, *J* = 7.4 Hz, 1H), 7.47 – 7.44(m, 1H), 7.34 – 7.28 (m, 2H).

2.2. General procedure for preparation of diaryl diazomethanes (Taking 2a for example):

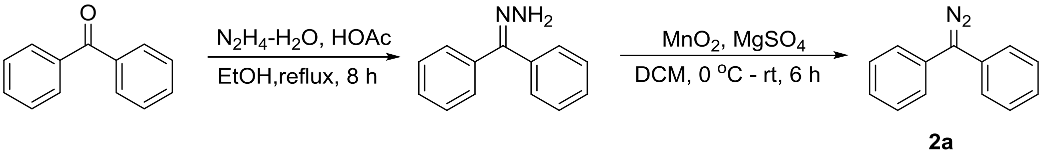

2a

Supplementary Figure 3. Synthetic methods for diaryl diazomethanes

Step 1: Hydrazine monohydrate (in water 70%) (1.43g, 20 mmol, 4 equiv) was added to benzophenone (911 mg, 5 mmol, 1 equiv) in ethanol (20 mL). Then HOAc (10 mol %) was added and the mixture was heated at reflux for 8 h. After cooling to room temperature, and removal of the solvent, the crude reaction mixture was purified on silica gel (petroleum ether and ethyl acetate) to afford the desired

products.

Step 2: Benzophenone hydrazone (785 mg, 4 mmol), anhydrous $MgSO_4$ (1.93g, 16 mmol, 4 equiv), and 20 mL DCM was cooled to 0 ^oC. To this rapidly stirring mixture was added activated MnO_2 (16 mmol, 4 equiv) in one portion. The reaction mixture was warmed to room temperature and kept stirring for 6 h. Then filtered, filtrate was collected. After removal of the solvent under reduced pressure, the residue was purified by Al_2O_3 base with PE/Et_3N = 20:1 as eluent to afford **2a** as a purple solid (3.8 g, 85% yield), which was kept at 0 - 8 ^oC.

(diazomethylene)dibenzene (2a)

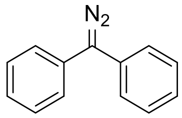

2a

Following the above procedure on 5 mmol scale, the product was obtained as a purple solid, yield: 90% (873 mg), R_f = 0.5 (Al_2O_3 base, PE/Et_3N = 10:1), column chromatography (Al_2O_3 base, PE/Et_3N = 20:1). Known compound.[2]

^{1}H NMR (500 MHz, $CDCl_3$) δ 7.40 (t, *J* = 7.5 Hz, 4H), 7.32 (d, *J* = 8.0 Hz, 4H), 7.20 (dd, *J* = 11.0, 4.1 Hz, 2H).

4,4'-(diazomethylene)bis(fluorobenzene) (**2b**)

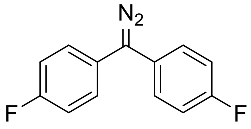

2b

Following the above procedure on 5 mmol scale, the product was obtained as a purple solid, yield: 65% (747 mg), R_f = 0.5 (Al_2O_3 base, PE/Et_3N = 10:1), column chromatography (Al_2O_3 base, PE/Et_3N = 20:1). Known compound.[3]

^{1}H NMR (500 MHz, $CDCl_3$) δ 7.24 – 7.19 (m, 4H), 7.13 – 7.07 (m, 4H).

^{19}F NMR (376 MHz, $CDCl_3$) δ -116.37 (s).

4,4'-(diazomethylene)bis(chlorobenzene)(**2c**)

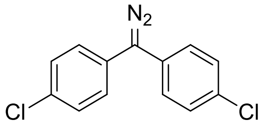

2c

Following the above procedure on 5 mmol scale, the product was obtained as a purple solid, yield: 72% (943 mg), R_f = 0.5 (Al_2O_3 base, PE/Et_3N = 10:1), column chromatography (Al_2O_3 base, PE/Et_3N = 20:1). Known compound.[3]

^{1}H NMR (500 MHz, $CDCl_3$) δ 7.38 – 7.34 (m, 4H), 7.20 – 7.17 (m, 4H).

3,3'-(diazomethylene)bis(chlorobenzene)(2d)

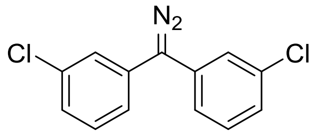

2d

Following the above procedure on 5 mmol scale, the product was obtained as a purple solid, yield: 60% (786 mg), R_f = 0.5 (Al_2O_3 base, PE/Et_3N = 10:1), column chromatography (Al_2O_3 base, PE/Et_3N = 20:1).

^{1}H NMR (500 MHz, $CDCl_3$) δ 7.32 (t, *J* = 7.9 Hz, 2H), 7.25 (s, 2H), 7.20 – 7.11 (m, 4H).

^{13}C NMR (101 MHz, $CDCl_3$) δ 135.3, 131.1, 130.4, 125.9, 124.8, 123.1.

HRMS (EI) m/z: $[M-H]^-$ Calcd. for $C_{13}H_8Cl_2N_2$ 263.0138; Found: 263.0138.

4,4'-(diazomethylene)bis(methoxybenzene)(2e)

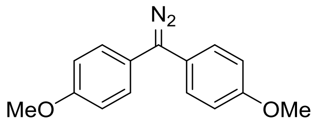

2e

Following the above procedure on 5 mmol scale, the product was obtained as a purple solid, yield: 58% (734 mg), R_f = 0.4 (Al_2O_3 base, PE/Et_3N = 10:1), column chromatography (Al_2O_3 base, PE/Et_3N = 20:1). Known compound.[3]

^{1}H NMR (500 MHz, $CDCl_3$) δ 7.22 – 7.16 (m, 4H), 6.98 – 6.92 (m, 4H), 3.83 (s, 6H).

3,3'-(diazomethylene)bis(methoxybenzene)(2f)

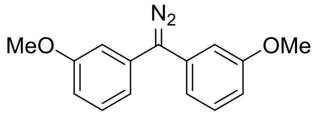

2f

Following the above procedure on 5 mmol scale, the product was obtained as a purple solid, yield: 62% (787 mg), R_f = 0.4 (Al_2O_3 base, PE/Et_3N = 10:1), column chromatography (Al_2O_3 base, PE/Et_3N = 20:1).

^{1}H NMR (500 MHz, $CDCl_3$) δ 7.31 (t, *J* = 8.0 Hz, 2H), 6.91 (dd, *J* = 7.1, 1.2 Hz, 2H), 6.86 (t, *J* = 1.8 Hz, 2H), 6.75 (dd, *J* = 8.3, 2.5 Hz, 2H), 3.81 (s, 6H).

^{13}C NMR (126 MHz, $CDCl_3$) δ 160.2, 131.0, 130.1, 117.8, 111.1, 110.8, 55.3.

HRMS (EI) m/z: $[M-H]^-$ Calcd. for $C_{15}H_{14}N_2O_2$ 255.1128; Found: 255.1124.

3,3'-(diazomethylene)bis(methylbenzene)(2g)

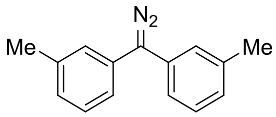

2g

Following the above procedure on 5 mmol scale, the product was obtained as a purple solid, yield: 46% (510 mg), R_f = 0.5 (Al_2O_3 base, PE/Et_3N = 10:1), column chromatography (Al_2O_3 base, PE/Et_3N = 20:1).

^{1}H NMR (500 MHz, $CDCl_3$) δ 7.32 – 7.27 (m, 2H), 7.13 (d, *J* = 6.6 Hz, 4H), 7.02 (d, *J* = 7.6 Hz, 2H), 2.38 (s, 6H).

^{13}C NMR (126 MHz, $CDCl_3$) δ 138.8, 129.5, 128.9, 126.4, 125.7, 122.4, 21.5.

HRMS (EI) m/z: $[M-H]^-$ Calcd. for $C_{15}H_{14}N_2$ 223.123; Found: 223.1228.

2,7-dibromo-9-diazo-9H-fluorene (2h)

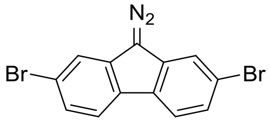

2h

Following the above procedure on 5 mmol scale, the product was obtained as a purple solid, yield: 35% (607 mg), R_f = 0.5 (Al_2O_3 base, PE/Et_3N = 10:1), column chromatography (Al_2O_3 base, PE/Et_3N = 20:1).

^{1}H NMR (400 MHz, $CDCl_3$) δ 7.72 (d, *J* = 8.2 Hz, 2H), 7.59 (d, *J* = 1.4 Hz, 2H), 7.42 (dd, *J* = 8.2, 1.3 Hz, 2H).

^{13}**C NMR (101 MHz, CDCl$_3$)** δ 134.4, 129.3, 127.9, 122.1, 122.0, 120.5.

HRMS (EI) m/z: [M-H]$^-$ Calcd. for $C_{13}H_6Br_2N_2$ 348.8971; Found: 348.8980.

9-diazo-9H-fluorene (**2i**)

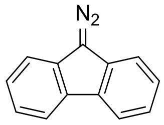

2i

Following the above procedure on 5 mmol scale, the product was obtained as a purple solid, yield: 45% (432 mg), R_f = 0.5 (Al_2O_3 base, PE/Et_3N = 10:1), column chromatography (Al_2O_3 base, PE/Et_3N = 20:1). Known compound.[4]

^{1}**H NMR (500 MHz, CDCl$_3$)** δ 7.95 (d, *J* = 7.8 Hz, 2H), 7.52 (d, *J* = 7.5 Hz, 2H), 7.42 – 7.37 (m, 2H), 7.35 – 7.31 (m, 2H).

9-diazo-9H-thioxanthene (**2j**)

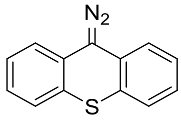

2j

Following the above procedure on 5 mmol scale, the product was obtained as a purple solid, yield: 50% (560 mg), R_f = 0.5 (Al_2O_3 base, PE/Et_3N = 10:1), column chromatography (Al_2O_3 base, PE/Et_3N = 20:1). Known compound.[5]

^{1}**H NMR (500 MHz, CDCl$_3$)** δ 7.12 – 7.09 (m, 2H), 7.03 – 6.95 (m, 4H), 6.75 (dd, *J* = 7.7, 1.1 Hz, 2H).

2.3. General procedure for preparation of α-diazoarylacetates.

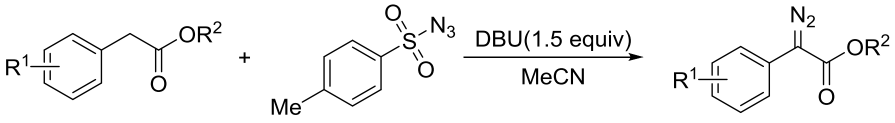

Supplementary Figure 4. Synthetic methods for α-diazoarylacetates

To a mixture of ester (5 mmol) and tosyl azide (1.48 g, 7.5 mmol) in anhydrous MeCN (15 mL), 1,8-diazabicyclo[5.4.0]undec-7-ene (DBU) (1.12 mL, 1.14 g, 7.5 mmol) was added. The reaction mixture was stirred at room temperature for overnight. Upon complete consumption of the starting

materials, the reaction mixture was quenched with saturated aqueous solution of NH_4Cl (5 mL), extracted with CH_2Cl_2 (3 × 30 mL), washed with brine (3 × 10 mL), dried over Na_2SO_4, and concentrated under reduced pressure to give the product. The residue was purified by flash chromatography (hexane/EtOAc, 9:1) to afford the α-diazoester.

10-diazoanthracen-9(10H)-one (**2k**)

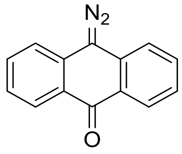

2k

Following the above procedure on 5 mmol scale, the product was obtained as a purple solid, yield: 58% (638 mg), R_f = 0.5 (Al_2O_3 base, PE/Et_3N = 10:1), column chromatography (Al_2O_3 base, PE/Et_3N = 20:1). Known compound.[6]

^{1}H NMR (400 MHz, $CDCl_3$) δ 8.55 (d, *J* = 8.0 Hz, 2H), 7.76 – 7.67 (m, 2H), 7.42 (t, *J* = 7.6 Hz, 2H), 7.36 (d, *J* = 8.1 Hz, 2H).

ethyl 2-diazo-2-phenylacetate (4a)

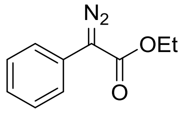

4a

Following the above procedure on 5 mmol scale, the product was obtained as a red oil, yield: 95% (903mg), R_f = 0.7 (silica gel, hexane/EtOAc = 9:1, v/v), column chromatography (silica gel, hexane/EtOAc = 10:1, v/v). Known compound.[7]

^{1}H NMR (600 MHz, $CDCl_3$) δ 7.49 (d, *J* = 7.9 Hz), 7.38 (t, *J* = 7.8 Hz), 7.18 (t, *J* = 7.4 Hz), 4.34 (q, *J* = 7.1 Hz), 1.35 (t, *J* = 7.1 Hz).

methyl 2-diazo-2-phenylacetate (4b)

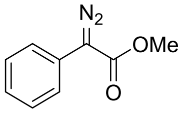

4b

Following the above procedure on 5 mmol scale, the product was obtained as a red oil, yield: 95%

(836mg), R_f = 0.7 (silica gel, hexane/EtOAc = 9:1, v/v), column chromatography (silica gel, hexane/EtOAc = 10:1, v/v). Known compound.[7]

^{1}H NMR (500 MHz, CDCl$_3$) δ 7.50 – 7.47 (m, 2H), 7.41 – 7.37 (m, 2H), 7.21 – 7.17 (m, 1H), 3.87 (s, 3H)

benzyl 2-diazo-2-phenylacetate (4c)

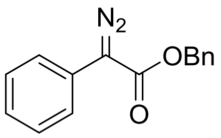

4c

Following the above procedure on 5 mmol scale, the product was obtained as a red solid, yield: 95% (836mg), R_f = 0.7 (silica gel, hexane/EtOAc = 9:1, v/v), column chromatography (silica gel, hexane/EtOAc = 10:1, v/v). Known compound.[8]

^{1}H NMR (500 MHz, CDCl$_3$) δ 7.52 – 7.49 (m, 2H), 7.43 – 7.34 (m, 7H), 7.21 – 7.18 (m, 1H), 5.33 (s, 2H).

***tert*-butyl 2-diazo-2-phenylacetate (4d)**

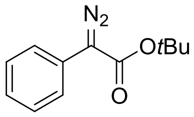

4d

Following the above procedure on 5 mmol scale, the product was obtained as a red solid, yield: 80% (926 mg), R_f = 0.7 (silica gel, hexane/EtOAc = 9:1, v/v), column chromatography (silica gel, hexane/EtOAc = 10:1, v/v). Known compound.[7]

^{1}H NMR (400 MHz, CDCl$_3$) δ 7.48 (d, *J* = 8.0 Hz, 2H), 7.37 (t, *J* = 7.8 Hz, 2H), 7.17 (t, *J* = 7.4 Hz, 1H), 1.57 (s, 9H).

phenyl 2-diazo-2-phenylacetate (4e)

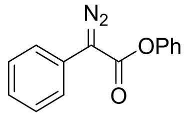

4e

Following the above procedure on 5 mmol scale, the product was obtained as a red solid, yield: 81% (964 mg), R_f = 0.7 (silica gel, hexane/EtOAc = 9:1, v/v), column chromatography (silica gel, hexane/EtOAc = 10:1, v/v). Known compound.[9]

^{1}H NMR (400 MHz, $CDCl_3$) δ 7.54 (d, *J* = 7.8 Hz, 2H), 7.41 (t, *J* = 7.8 Hz, 4H), 7.30 – 7.16 (m, 4H).

ethyl 2-(4-(tert-butyl)phenyl)-2-diazoacetate (4f)

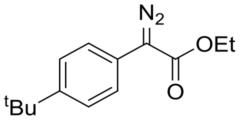

4f

Following the above procedure on 5 mmol scale, the product was obtained as a red solid, yield: 95% (1107mg), R_f = 0.7 (silica gel, hexane/EtOAc = 9:1, v/v), column chromatography (silica gel, hexane/EtOAc = 10:1, v/v). Known compound.[10]

^{1}H NMR (500 MHz, $CDCl_3$) δ 7.41 (s, 4H), 4.33 (q, *J* = 7.1 Hz, 2H), 1.34 (t, *J* = 7.1 Hz, 3H), 1.32 (s, 9H).

ethyl 2-diazo-2-(p-tolyl)acetate (4g)

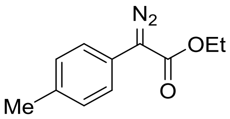

4g

Following the above procedure on 5 mmol scale, the product was obtained as a red solid, yield: 95% (969mg), R_f = 0.7 (silica gel, hexane/EtOAc = 9:1, v/v), column chromatography (silica gel, hexane/EtOAc = 10:1, v/v). Known compound.[10]

^{1}H NMR (600 MHz, $CDCl_3$) δ 7.38 – 7.34 (m, 2H), 7.20 (d, *J* = 8.1 Hz, 2H), 4.33 (q, *J* = 7.1 Hz, 2H), 2.34 (s, 3H), 1.34 (t, *J* = 7.1 Hz, 3H).

ethyl 2-diazo-2-(m-tolyl)acetate (4h)

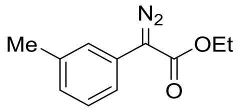

4h

Following the above procedure on 5 mmol scale, the product was obtained as a red oild, yield: 95% (969mg), R_f = 0.7 (silica gel, hexane/EtOAc = 9:1, v/v), column chromatography (silica gel, hexane/EtOAc = 10:1, v/v). Known compound.[10]

^{1}H NMR (500 MHz, CDCl$_3$) δ 7.29 (d, *J* = 17.5 Hz, 3H), 6.99 (s, 1H), 4.33 (dd, *J* = 4.4, 1.7 Hz, 2H), 2.35 (s, 3H), 1.37 – 1.31 (m, 3H).

ethyl 2-diazo-2-(3-methoxyphenyl)acetate (4i)

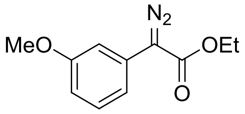

4i

Following the above procedure on 5 mmol scale, the product was obtained as a red solid, yield: 95% (1045mg), R_f = 0.7 (silica gel, hexane/EtOAc = 9:1, v/v), column chromatography (silica gel, hexane/EtOAc = 10:1, v/v). Known compound.[10]

^{1}H NMR (400 MHz, CDCl$_3$) δ 7.32 – 7.25 (m, 1H), 7.17 (s, 1H), 6.98 (d, *J* = 7.8 Hz, 1H), 6.72 (d, *J* = 8.2 Hz, 1H), 4.33 (q, *J* = 7.1 Hz, 2H), 3.82 (s, 3H), 1.34 (t, *J* = 7.1 Hz, 3H).

ethyl 2-(benzo[d][1,3]dioxol-5-yl)-2-diazoacetate (4j)

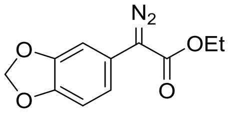

4j

Following the above procedure on 5 mmol scale, the product was obtained as a red solid, yield: 65% (760 mg), R_f = 0.7 (silica gel, hexane/EtOAc = 9:1, v/v), column chromatography (silica gel, hexane/EtOAc = 10:1, v/v). Known compound.[10]

^{1}H NMR (600 MHz, CDCl$_3$) δ 7.06 (d, *J* = 1.8 Hz, 1H), 6.85 (dd, *J* = 8.2, 1.8 Hz, 1H), 6.83 (d, *J* = 8.2 Hz, 1H), 5.96 (s, 2H), 4.31 (q, *J* = 7.1 Hz, 2H), 1.33 (t, *J* = 7.1 Hz, 3H).

ethyl 2-([1,1'-biphenyl]-4-yl)-2-diazoacetate (4k)

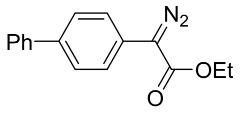

4k

Following the above procedure on 5 mmol scale, the product was obtained as a red solid, yield: 95% (1264mg), R_f = 0.7 (silica gel, hexane/EtOAc = 9:1, v/v), column chromatography (silica gel, hexane/EtOAc = 10:1, v/v). Known compound.[10]

^{1}H NMR (500 MHz, CDCl$_3$) δ 7.65 – 7.62 (m, 2H), 7.61 – 7.59 (m, 2H), 7.58 – 7.55 (m, 2H), 7.45 (dd, *J* = 10.5, 4.9 Hz, 2H), 7.37 – 7.33 (m, 1H), 4.36 (q, *J* = 7.1 Hz, 2H), 1.37 (t, *J* = 7.1 Hz, 3H).

methyl 3-(1-diazo-2-ethoxy-2-oxoethyl)benzoate (4l)

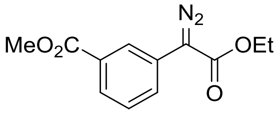

4l

Following the above procedure on 5 mmol scale, the product was obtained as a red solid, yield: 95% (1107mg), R_f = 0.7 (silica gel, hexane/EtOAc = 9:1, v/v), column chromatography (silica gel, hexane/EtOAc = 10:1, v/v). Known compound.[11]

^{1}H NMR (500 MHz, CDCl$_3$) δ 8.06 (t, *J* = 1.8 Hz, 1H), 7.83 (dd, *J* = 7.7, 1.4 Hz, 1H), 7.78 – 7.75 (m, 1H), 7.45 (t, *J* = 7.9 Hz, 1H), 4.34 (q, *J* = 7.1 Hz, 2H), 3.91 (s, 3H), 1.34 (t, *J* = 7.1 Hz, 3H).

ethyl 2-diazo-2-(4-fluorophenyl)acetate (4m)

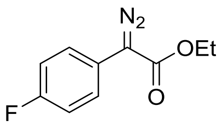

4m

Following the above procedure on 5 mmol scale, the product was obtained as a red oild, yield: 95% (988 mg), R_f = 0.7 (silica gel, hexane/EtOAc = 9:1, v/v), column chromatography (silica gel, hexane/EtOAc = 10:1, v/v). Known compound.[12]

^{1}H NMR (500 MHz, CDCl$_3$) δ 7.47 – 7.42 (m, 2H), 7.12 – 7.06 (m, 2H), 4.33 (q, *J* = 7.1 Hz, 2H), 1.34 (t, *J* = 7.1 Hz, 3H).

^{19}F NMR (376 MHz, CDCl$_3$) δ -116.40 (s).

ethyl 2-(4-chlorophenyl)-2-diazoacetate (4n)

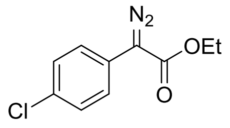

4n

Following the above procedure on 5 mmol scale, the product was obtained as a red solid, yield: 95% (1064 mg), R_f = 0.7 (silica gel, hexane/EtOAc = 9:1, v/v), column chromatography (silica gel, hexane/EtOAc = 10:1, v/v). Known compound.[10]

^{1}H NMR (500 MHz, $CDCl_3$) δ 7.43 – 7.40 (m, 2H), 7.36 – 7.32 (m, 2H), 4.33 (q, *J* = 7.1 Hz, 2H), 1.34 (t, *J* = 7.1 Hz, 3H).

ethyl 2-(4-bromophenyl)-2-diazoacetate (4o)

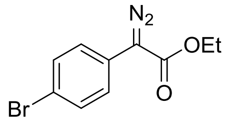

4o

Following the above procedure on 5 mmol scale, the product was obtained as a red solid, yield: 95% (1273 mg), R_f = 0.7 (silica gel, hexane/EtOAc = 9:1, v/v), column chromatography (silica gel, hexane/EtOAc = 10:1, v/v). Known compound.[12]

^{1}H NMR (500 MHz, $CDCl_3$) δ 7.50 – 7.47 (m, 2H), 7.38 – 7.34 (m, 2H), 4.33 (q, *J* = 7.1 Hz, 2H), 1.33 (t, *J* = 7.1 Hz, 3H).

ethyl 2-diazo-2-(naphthalen-2-yl)acetate (4p)

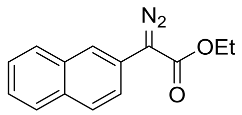

4p

Following the above procedure on 5 mmol scale, the product was obtained as a red solid, yield: 80% (960 mg), R_f = 0.7 (silica gel, hexane/EtOAc = 9:1, v/v), column chromatography (silica gel, hexane/EtOAc = 10:1, v/v). Known compound.[10]

^{1}H NMR (500 MHz, $CDCl_3$) δ 8.02 (d, *J* = 1.8 Hz, 1H), 7.85 (d, *J* = 8.8 Hz, 1H), 7.80 (d, *J* = 8.7 Hz, 2H), 7.54 (dd, *J* = 8.7, 2.0 Hz, 1H), 7.50 – 7.42 (m, 2H), 4.38 (q, *J* = 7.1 Hz, 2H), 1.38 (t, *J* = 7.1 Hz,

3H).

2.4. Reaction optimization

Supplementary Table 1. Effect of ligands [a]

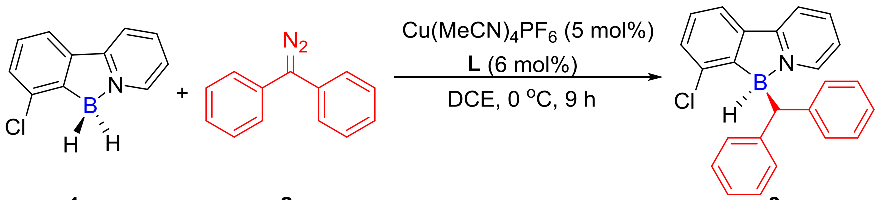

1a **2a** **3a**

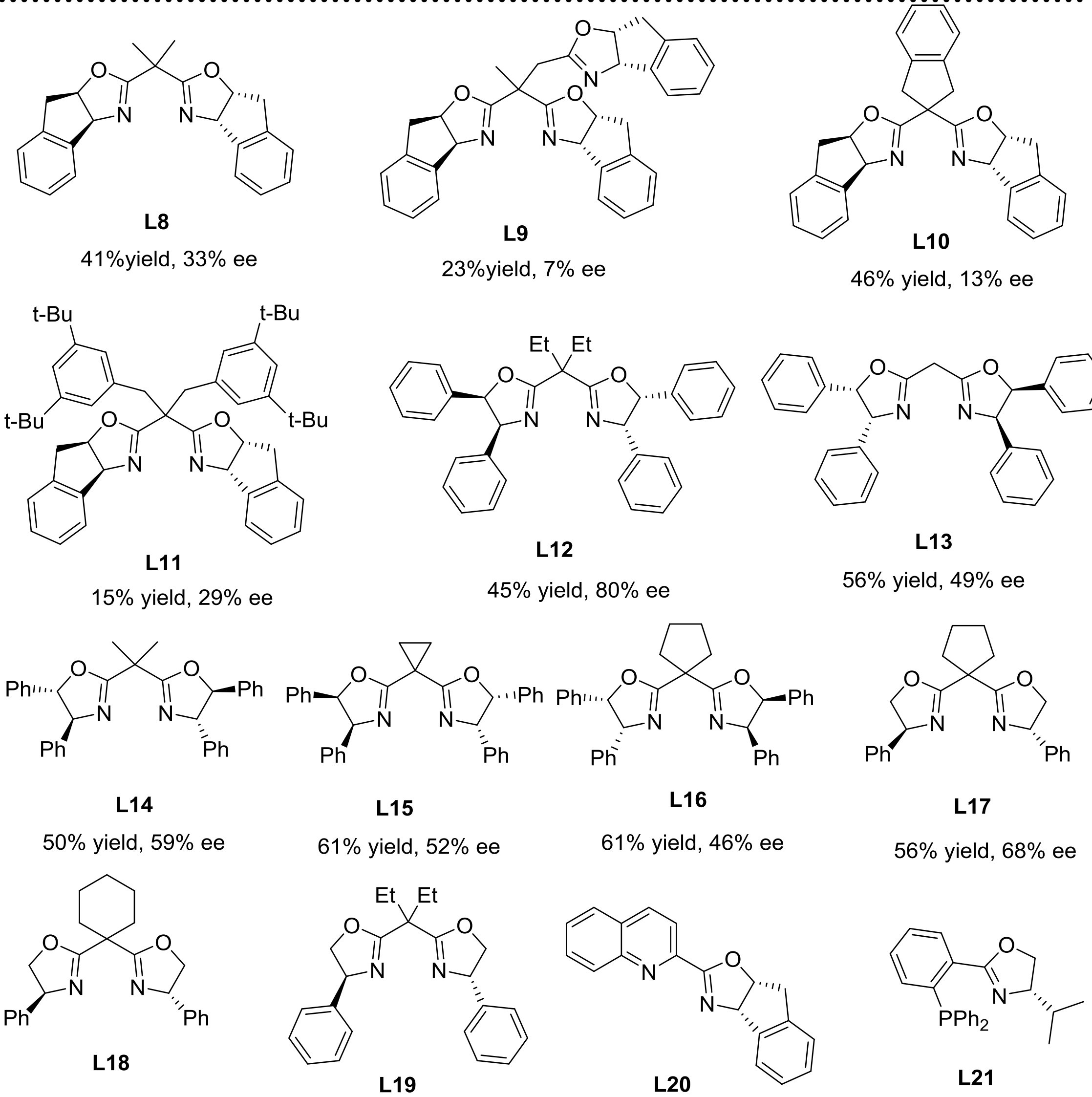

L8 41%yield, 33% ee

L9 23%yield, 7% ee

L10 46% yield, 13% ee

L11 15% yield, 29% ee

L12 45% yield, 80% ee

L13 56% yield, 49% ee

L14 50% yield, 59% ee

L15 61% yield, 52% ee

L16 61% yield, 46% ee

L17 56% yield, 68% ee

L18 35% yield, 45% ee

L19 63% yield, 50% ee

L20 no reaction

L21 no reaction

[a]Conditions: 2-arylpyridine-borane (**1a**) (0.12 mmol, 1.2 equiv), ethyl α-diazophenylacetate (**2a**) (0.1 mmol, 1.0 equiv), $Cu(MeCN)_4PF_6$ (5 mol%), ligand (6 mol%), solvent (1 mL) at 0 °C for 9 h. Yield determined by ^1H NMR. Enantioselectivity determined by chiral HPLC.

Supplementary Table 2. Effect of other variable [a]

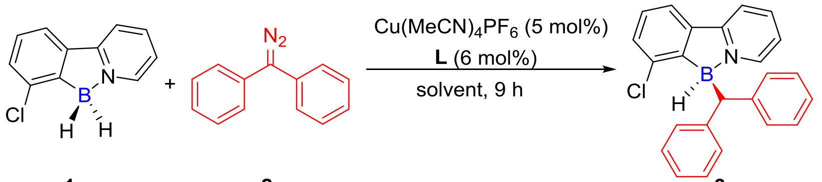

1a **2a** **3a**

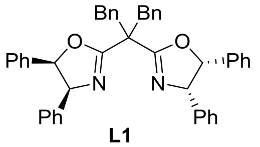

L1

entry	**1a** (x equiv)	**2a** (x equiv)	solvent	temp (°C)	yield (%)[b]	ee (%)[d]
1	1.2	1	DCE	-20	90	85
2	1	1.2	DCE	-20	90	84
3	1	1.5	DCE	-20	90	84
4	1	2	DCE	-20	90	84
5	1	1..2	DCM	-20	95	86
6	1	1.2	CCl_3H	-20	65	75
7	1	1.2	CCl_4	-20	95	82
8[c]	1	1.2	DCM	-35	95	91

[a]Conditions: 2-arylpyridine-borane **1a** (0.1 mmol, 1.0 equiv), symmetric diaryl diazomethane **2a** (0.12 mmol, 1.2 equiv), $Cu(MeCN)_4PF_6$ (5 mol%), **L** (6 mol%), solvent (1 mL) at -35 °C for 9 h. [b]Determined by ^1H NMR. [c] Isolated yield. [d] Determined by chiral HPLC.

2.5. General procedure for boron-stereogenic compounds

General procedure A for the boron-stereogenic compounds with 2-arylpyridine-borane and diaryl diazomethane

In air, a 10 mL schlenk tube was charged with **1** (0.10 mmol, 1.0 equiv), **2** (0.12 mmol, 1.2 equiv), $Cu(MeCN)_4PF_6$ (5 mol%), **L1** (6 mol%). The tube was evacuated and filled with argon for three cycles. Then, 1 mL of DCM was added under argon. The reaction was allowed to stir at -35 °C for 9 hours. Upon completion, proper amount of silica gel was added to the reaction mixture. After removal of the solvent, the crude reaction mixture was purified on silica gel (petroleum ether and ethyl acetate) to afford the desired products.

6(*S*)-benzhydryl-7-chloro-6H-5λ⁴-benzo[3,4][1,2]azaborolo[1,5-a]pyridine (3a)

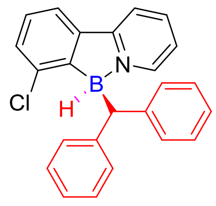

3a

Following the above procedure on 0.1 mmol scale, isolated yield: 95%, (34.9 mg), white solid (mp: 156.7 – 158.6 °C), R_f = 0.3 (silica gel, hexane/EtOAc = 10:1, v/v), column chromatography (silica gel, hexane/EtOAc = 10:1, v/v).

^{1}H NMR (500 MHz, $CDCl_3$) δ 7.93 (t, *J* = 7.7 Hz, 1H), 7.86 (d, *J* = 5.7 Hz, 1H), 7.79 (d, *J* = 8.0 Hz, 1H), 7.50 (dd, *J* = 7.6, 2.6 Hz, 3H), 7.32 – 7.25 (m, 3H), 7.16 – 7.09 (m, 3H), 6.89 – 6.81 (m, 3H), 6.54 – 6.48 (m, 2H), 4.58 (d, *J* = 2.8 Hz, 1H).

^{13}C NMR (126 MHz, $CDCl_3$) δ 157.2, 147.1, 145.3, 143.5, 140.1, 137.8, 136.7, 130.6, 128.9, 128.3, 127.9, 127.2, 127.1, 124.0, 123.7, 121.3, 119.5, 118.1.

^{11}B NMR (128 MHz, $CDCl_3$) δ -0.48 (d, *J* = 88.7 Hz).

HRMS (ESI) m/z: $[M+H]^+$ Calcd. for $C_{24}H_{19}BClN$ 368.1372; Found: 368.1375.

HPLC analysis: DAICEL CHIRALCEL AD-H, hexane/isopropanol = 90/10, 1mL/min, λ = 254 nm, t_R (minor) = 7.955 min, t_R (major) = 9.056 min, 91% ee.

$[\alpha]^{25}_D$: -60.29 (*c* 0.57, $CHCl_3$).

6(*S*)-(bis(4-fluorophenyl)methyl)-7-chloro-6H-5λ^4-benzo[3,4][1,2]azaborolo[1,5-a]pyridine (3b)

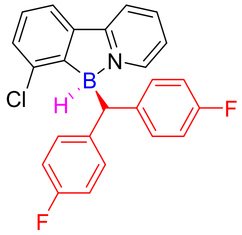

3b

Following the above procedure on 0.1 mmol scale, isolated yield: 93% (37.5 mg), white solid (mp: 141.9 – 143.2 °C), R_f = 0.3 (silica gel, hexane/EtOAc = 10:1, v/v), column chromatography (silica gel, hexane/EtOAc = 10:1, v/v).

^{1}H NMR (400 MHz, $CDCl_3$) δ 8.01 – 7.94 (m, 1H), 7.90 (d, *J* = 5.8 Hz, 1H), 7.83 (d, *J* = 8.1 Hz, 1H), 7.52 (dd, *J* = 7.6, 0.5 Hz, 1H), 7.37 (dd, *J* = 8.2, 5.7 Hz, 2H), 7.29 (dd, *J* = 7.8, 0.5 Hz, 1H), 7.20 (ddd, *J* = 7.2, 5.9, 1.2 Hz, 1H), 7.13 (t, *J* = 7.7 Hz, 1H), 6.99 – 6.92 (m, 2H), 4.46 (s, 1H).

^{13}C NMR (101 MHz, $CDCl_3$) δ 161.3 (d, *J* = 27.5 Hz), 158.9 (d, *J* = 27 Hz), 157.3, 143.2, 142.3 (d, *J* = 3.3 Hz), 140.9 (d, *J* = 3.1 Hz), 140.3, 137.8, 136.9, 130.8, 130.0 (d, *J* = 7.3 Hz), 129.2 (d, *J* = 7.4 Hz), 127.4, 121.4, 119.6, 118.3, 114.5 (d, *J* = 20.5 Hz), 114.0 (d, *J* = 20.5 Hz).

^{11}B NMR (128 MHz, $CDCl_3$) δ - 0.64 (d, *J* = 91.4 Hz).

^{19}F NMR (376 MHz, CDCl$_3$) δ -120.08 (s), -120.23 (s).

HRMS (ESI) m/z: [M+H]$^+$ Calcd. for $C_{24}H_{17}BClF_2N$ 404.1183; Found: 404.1181.

HPLC analysis: DAICEL CHIRALCEL OD-H, hexane/isopropanol = 90/10, 1mL/min, λ = 254 nm, t_R (minor) = 11.317 min, t_R (major) = 14.719 min, 91% ee.

$[\alpha]^{25}_D$: - 72.63 (*c* 0.57, $CHCl_3$).

6(*S*)-(bis(4-chlorophenyl)methyl)-7-chloro-6H-5λ^4-benzo[3,4][1,2]azaborolo[1,5-a]pyridine (3c)

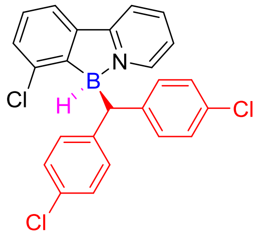

3c

Following the above procedure on 0.1 mmol scale, isolated yield: 96%, (41.8 mg), white solid (mp: 120.2 – 121.7 ^{o}C), R_f = 0.3 (silica gel, hexane/EtOAc = 10:1, v/v), column chromatography (silica gel, hexane/EtOAc = 10:1, v/v).

^{1}H NMR (400 MHz, CDCl$_3$) δ 7.98 (t, *J* = 7.7 Hz, 1H), 7.91 (d, *J* = 5.7 Hz, 1H), 7.83 (d, *J* = 8.1 Hz, 1H), 7.52 (d, *J* = 7.6 Hz, 1H), 7.35 (d, *J* = 8.4 Hz, 2H), 7.29 (d, *J* = 7.8 Hz, 1H), 7.25 – 7.20 (m, 3H), 7.13 (t, *J* = 7.7 Hz, 1H), 6.82 (d, *J* = 8.4 Hz, 2H), 6.41 (d, *J* = 8.4 Hz, 2H), 4.47 (d, *J* = 2.6 Hz, 1H).

^{13}C NMR (101 MHz, CDCl$_3$) δ 157.3, 145.1, 143.5, 143.2, 140.4, 137.7, 136.6, 130.9, 130.1, 129.8, 129.5, 129.3, 128.0, 127.5, 127.4, 121.5, 119.7, 118.4.

^{11}B NMR (128 MHz, CDCl$_3$) δ -0.24 (d, *J* = 22.6 Hz).

HRMS (ESI) m/z: [M+H]$^+$ Calcd. for $C_{24}H_{17}BCl_3N$ 436.0592; Found: 436.0588.

HPLC analysis: DAICEL CHIRALCEL AD-H, hexane/isopropanol = 90/10, 1mL/min, λ = 254 nm, t_R (minor) = 8.850 min, t_R (major) = 12.301 min, 92% ee.

$[\alpha]^{25}_D$: -85.13 (*c* 0.52, $CHCl_3$).

6(*S*)-(bis(3-chlorophenyl)methyl)-7-chloro-6H-5λ^4-benzo[3,4][1,2]azaborolo[1,5-a]pyridine (3d)

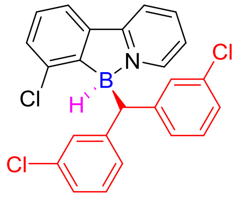

3d

Following the above procedure on 0.1 mmol scale, isolated yield: 98% (42.6 mg), white solid (mp: 130.5 – 132.1 ^oC), R_f = 0.3 (silica gel, hexane/EtOAc = 10:1, v/v), column chromatography (silica gel, hexane/EtOAc = 10:1, v/v).

^{1}H NMR (400 MHz, CDCl$_3$) δ 8.04 – 7.96 (m, 1H), 7.91 (d, *J* = 5.7 Hz, 1H), 7.83 (dd, *J* = 8.1, 3.4 Hz, 1H), 7.51 (dd, *J* = 7.6, 1.7 Hz, 1H), 7.44 (s, 1H), 7.30 (t, *J* = 7.6 Hz, 2H), 7.26 – 7.17 (m, 2H), 7.13 (t, *J* = 7.1 Hz, 2H), 6.81 – 6.75 (m, 2H), 6.47 – 6.39 (m, 1H), 6.36 – 6.29 (m, 1H), 4.46 (d, *J* = 2.7 Hz, 1H).

^{13}C NMR (126 MHz, CDCl$_3$) δ 157.3, 148.6, 146.6, 143.2, 140.6, 137.8, 136.6, 134.0, 133.0, 130.8, 129.2, 128.8, 128.6, 127.8, 127.5, 127.0, 126.4, 124.5, 124.1, 121.6, 119.6, 118.3.

^{11}B NMR (128 MHz, CDCl$_3$) δ -0.8 (d, *J* = 94.5 Hz).

HRMS (ESI) m/z: $[M+H]^+$ Calcd. for $C_{24}H_{17}BCl_3N$ 436.0592; Found: 436.0599.

HPLC analysis: DAICEL CHIRALCEL AD-H, hexane/isopropanol = 90/10, 1mL/min, λ = 254 nm, t_R (minor) = 7.639 min, t_R (major) = 10.251 min, 89% ee.

$[\alpha]^{25}_D$: - 31.39 (*c* 0.55, $CHCl_3$).

6(*S*)-(bis(4-methoxyphenyl)methyl)-7-chloro-6H-5λ^4-benzo[3,4][1,2]azaborolo[1,5-a]pyridine (3e)

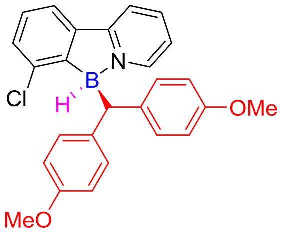

3e

Following the above procedure on 0.1 mmol scale, isolated yield: 88%, (37.6 mg), white solid (mp: 150.2 – 151.3 ^oC), R_f = 0.3 (silica gel, hexane/EtOAc = 8:1, v/v), column chromatography (silica gel, hexane/EtOAc = 8:1, v/v).

^{1}H NMR (500 MHz, CDCl$_3$) δ 7.92 (dd, *J* = 12.6, 6.7 Hz, 2H), 7.80 (d, *J* = 8.0 Hz, 1H), 7.50 (d, *J* = 7.6 Hz, 1H), 7.37 (d, *J* = 8.7 Hz, 2H), 7.27 (dd, *J* = 7.8, 0.6 Hz, 1H), 7.16 (dd, *J* = 6.9, 6.3 Hz, 1H),

7.10 (t, J = 7.7 Hz, 1H), 6.84 (d, J = 8.7 Hz, 2H), 6.44 – 6.37 (m, 4H), 4.43 (d, J = 3.1 Hz, 1H), 3.81 (s, 3H), 3.61 (s, 3H).

^{13}C NMR (101 MHz, $CDCl_3$) δ 157.2, 156.4, 156.1, 143.4, 140.0, 139.6, 138.0, 137.8, 136.7, 130.6, 129.7, 128.9, 127.1, 121.2, 119.5, 118.1, 113.3, 112.7, 55.3, 54.9.

^{11}B NMR (128 MHz, $CDCl_3$) δ -0.51 (d, J = 61.2 Hz).

HRMS (ESI) m/z: $[M+H]^+$ Calcd. for $C_{26}H_{23}BClNO_2$ 428.1583; Found: 428.1584.

HPLC analysis: DAICEL CHIRALCEL AD-H, hexane/isopropanol = 80/20, 1mL/min, λ = 254 nm, t_R (minor) = 10.920 min, t_R (major) = 18.294 min, 86% ee.

$[\alpha]^{25}_D$: -110.58 (c 0.52, $CHCl_3$).

6(*S*)-(bis(3-methoxyphenyl)methyl)-7-chloro-6H-5λ^4-benzo[3,4][1,2]azaborolo[1,5-a]pyridine (**3f**)

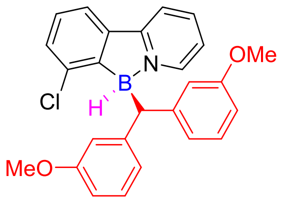

3f

Following the above procedure on 0.1 mmol scale, isolated yield: 97%, (41.4 mg), white solid (mp: 127.3 – 128.7 ^oC), R_f = 0.3 (silica gel, hexane/EtOAc = 8:1, v/v), column chromatography (silica gel, hexane/EtOAc = 8:1, v/v).

^{1}H NMR (500 MHz, $CDCl_3$) δ 7.93 (dd, J = 14.1, 6.7 Hz, 2H), 7.80 (d, J = 8.1 Hz, 1H), 7.50 (d, J = 7.4 Hz, 1H), 7.27 (dd, J = 7.8, 0.5 Hz, 1H), 7.21 – 7.15 (m, 2H), 7.13 – 7.06 (m, 3H), 6.73 (t, J = 7.9 Hz, 1H), 6.70 (dd, J = 8.1, 2.5 Hz, 1H), 6.37 – 6.35 (m, 1H), 6.13 – 6.10 (m, 1H), 6.04 (d, J = 7.6 Hz, 1H), 4.54 (d, J = 3.2 Hz, 1H), 3.78 (s, 3H), 3.50 (s, 3H).

^{13}C NMR (101 MHz, $CDCl_3$) δ 159.5, 158.7, 157.2, 148.8, 146.8, 143.6, 140.1, 137.9, 136.7, 130.6, 128.7, 127.9, 127.2, 121.5, 121.3, 120.8, 119.5, 118.1, 114.7, 113.6, 109.9, 109.5, 55.1, 54.8.

^{11}B NMR (128 MHz, $CDCl_3$) δ 0.00 (s).

HRMS (ESI) m/z: $[M+H]^+$ Calcd. for $C_{26}H_{23}BClNO_2$ 428.1583; Found: 428.1579.

HPLC analysis: DAICEL CHIRALCEL AD-H, hexane/isopropanol = 70/30, 1mL/min, λ = 254 nm, t_R (minor) = 5.788 min, t_R (major) = 19.167 min, 90% ee.

$[\alpha]^{25}_D$: -60.29 (c 0.57, $CHCl_3$).

7(*S*)-chloro-6-(di-m-tolylmethyl)-6H-5λ^4-benzo[3,4][1,2]azaborolo[1,5-a]pyridine (**3g**)

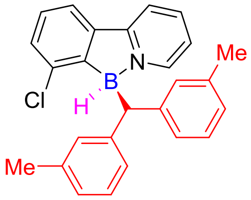

3g

Following the above procedure on 0.1 mmol scale, isolated yield: 95%, (37.5 mg), white solid (mp: 157.6 – 158.5 °C), R_f = 0.3 (silica gel, hexane/EtOAc = 10:1, v/v), column chromatography (silica gel, hexane/EtOAc = 10:1, v/v).

^{1}H NMR (500 MHz, CDCl$_3$) δ 7.96 – 7.88 (m, 2H), 7.78 (d, *J* = 8.1 Hz, 1H), 7.51 – 7.47 (m, 1H), 7.38 (s, 1H), 7.30 – 7.25 (, 2Hm), 7.20 – 7.14 (m, 2H), 7.10 (t, *J* = 7.7 Hz, 1H), 6.96 (d, *J* = 7.4 Hz, 1H), 6.72 (t, *J* = 7.5 Hz, 1H), 6.62 (d, *J* = 7.5 Hz, 1H), 6.32 (s, 1H), 6.24 (d, *J* = 7.7 Hz, 1H), 4.49 (d, *J* = 3.2 Hz, 2H), 2.35 (s, 3H), 2.00 (s, 3H).

^{13}C NMR (126 MHz, CDCl$_3$) δ 157.2, 147.1, 145.2, 143.6, 140.0, 137.8, 137.3, 136.7, 136.3, 130.5, 129.9, 129.2, 127.7, 127.1, 126.9, 125.9, 125.2, 124.7, 124.3, 121.1, 119.4, 117.9, 21.7, 21.3.

^{11}B NMR (128 MHz, CDCl$_3$) δ -0.55 (d, *J* = 75.6 Hz).

HRMS (ESI) m/z: $[M+Na]^+$ Calcd. for $C_{26}H_{23}BClN$ 418.1504; Found: 418.1506.

HPLC analysis: DAICEL CHIRALCEL AD-H, hexane/isopropanol = 80/20, 1mL/min, λ = 254 nm, t_R (minor) = 4.463 min, t_R (major) = 5.895 min, 86% ee.

$[\alpha]^{25}_D$: -76.87.0 (*c* 0.41, CHCl$_3$).

7(*S*)-chloro-6-(2,7-dibromo-9H-fluoren-9-yl)-6H-5λ^4-benzo[3,4][1,2]azaborolo[1,5-a]pyridine (**3h**)

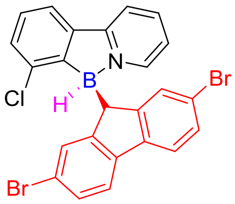

3h

Following the above procedure on 0.1 mmol scale, isolated yield: 90%, (46.9 mg), white solid (mp: 176.3 – 177.5 °C), R_f = 0.3 (silica gel, hexane/EtOAc = 10:1, v/v), column chromatography (silica gel,

hexane/EtOAc = 10:1, v/v).

^{1}H NMR (400 MHz, $CDCl_3$) δ 8.13 (s, 1H), 7.67 (d, *J* = 8.0 Hz, 2H), 7.63 (dd, *J* = 9.3, 4.7 Hz, 1H), 7.57 (d, *J* = 7.8 Hz, 1H), 7.52 (d, *J* = 8.1 Hz, 1H), 7.46 (dd, *J* = 8.1, 1.2 Hz, 1H), 7.40 (t, *J* = 7.7 Hz, 1H), 7.24 (s, 1H), 7.01 (dd, *J* = 8.1, 1.3 Hz, 1H), 6.78 – 6.72 (m, 2H), 6.00 (s, 1H), 4.59 (s, 1H).

^{13}C NMR (101 MHz, $CDCl_3$) δ 156.2, 153.3, 150.4, 141.6, 140.1, 138.8, 138.5, 137.4, 137.1, 131.0, 128.4, 128.3, 128.1, 127.2, 126.4, 121.1, 120.8, 120.4, 120.0, 119.9, 119.5, 117.8.

^{11}B NMR (128 MHz, $CDCl_3$) δ -1.48 (d, *J* = 92.5 Hz).

HRMS (ESI) m/z: $[M+H]^+$ Calcd. for $C_{24}H_{15}BClBr_2N$ 521.9426; Found: 521.9428.

HPLC analysis: DAICEL CHIRALCEL AD-H, hexane/isopropanol = 90/10, 1mL/min, λ = 254 nm, t_R (minor) = 7.145 min, t_R (major) = 9.221 min, 83% ee.

$[\alpha]^{25}_D$: -142.27 (*c* 0.5, $CHCl_3$).

7(*S*)-chloro-6-(9H-fluoren-9-yl)-6H-5λ^4-benzo[3,4][1,2]azaborolo[1,5-a]pyridine (**3i**)

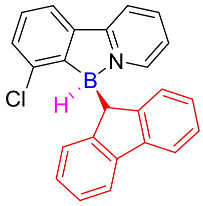

3i

Following the above procedure on 0.1 mmol scale, isolated yield: 87%, (31.8 mg), white solid (mp: 184.5 – 185.7 °C), R_f = 0.3 (silica gel, hexane/EtOAc = 10:1, v/v), column chromatography (silica gel, hexane/EtOAc = 10:1, v/v).

^{1}H NMR (400 MHz, $CDCl_3$) δ 8.05 (d, *J* = 7.5 Hz, 1H), 7.74 (d, *J* = 7.5 Hz, 1H), 7.64 (dd, *J* = 7.6, 0.5 Hz, 1H), 7.62 – 7.54 (m, 3H), 7.51 – 7.44 (m, 2H), 7.37 (t, *J* = 7.7 Hz, 2H), 6.92 (t, *J* = 7.4 Hz, 1H), 6.72 – 6.65 (m, 3H), 6.09 – 6.03 (m, 1H), 4.69 (s, 1H).

^{13}C NMR (101 MHz, $CDCl_3$) δ 156.0, 151.3, 148.6, 141.8, 141.0, 139.7, 139.6, 138.6, 137.2, 130.7, 127.8, 126.4, 125.4, 125.1, 124.7, 123.9, 123.2, 120.9, 119.7, 119.1, 118.7, 117.5.

^{11}B NMR (128 MHz, $CDCl_3$) δ -1.09 (d, *J* = 103.5 Hz).

HRMS (ESI) m/z: $[M+H]^+$ Calcd. for $C_{24}H_{17}BClN$ 366.1215; Found: 366.1217.

HPLC analysis: DAICEL CHIRALCEL AD-H, hexane/isopropanol = 90/10, 1mL/min, λ = 254 nm, t_R (minor) = 10.358 min, t_R (major) = 15.207 min, 98% ee.

[α]$^{25}_{D}$: -312.31 (*c* 0.52, $CHCl_3$).

7(*S*)-chloro-6-(9H-thioxanthen-9-yl)-6H-5λ^4-benzo[3,4][1,2]azaborolo[1,5-a]pyridine (**3j**)

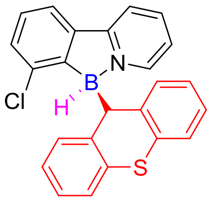

3j

Following the above procedure on 0.1 mmol scale, isolated yield: 57%, (22.6 mg), yellow solid (mp: 178.5 – 180.0 °C), R_f = 0.3 (silica gel, hexane/EtOAc = 10:1, v/v), column chromatography (silica gel, hexane/EtOAc = 10:1, v/v).

^{1}H NMR (400 MHz, $CDCl_3$) δ 7.90 – 7.76 (m, 1H), 7.77 (d, *J* = 5.7 Hz, 1H), 7.62 (d, *J* = 8.1 Hz, 1H), 7.55 (d, *J* = 7.6 Hz, 1H), 7.46 – 7.37 (m, 2H), 7.30 – 7.24 (m, 1H), 7.19 – 7.08 (m, 3H), 7.06 – 7.03 (m, 1H), 6.76 (dd, *J* = 7.5, 1.3 Hz, 1H), 6.73 – 6.66 (m, 2H), 6.66 – 6.60 (m, 1H), 4.38 (d, *J* = 3.8 Hz, 1H).

^{13}C NMR (101 MHz, $CDCl_3$) δ 145.0, 140.3, 130.8, 130.5, 129.2, 129.2, 127.9, 127.5, 126.6, 125.4, 124.8, 124.7, 123.8, 121.2, 119.6, 117.5.

^{11}B NMR (128 MHz, $CDCl_3$) δ 0.49 (d, *J* = 115.1 Hz).

HRMS (ESI) m/z: $[M+H]^+$ Calcd. for $C_{24}H_{17}BClNS$ 398.0936; Found: 398.0940.

HPLC analysis: DAICEL CHIRALCEL AD-H, hexane/isopropanol = 70/30, 1mL/min, λ = 254 nm, t_R (minor) = 5.562 min, t_R (major) = 16.458 min, 93% ee.

[α]$^{25}_{D}$: -12.85 (*c* 0.55, $CHCl_3$).

10(*S*)-(7-chloro-6H-5λ^4-benzo[3,4][1,2]azaborolo[1,5-a]pyridin-6-yl)anthracen-9(10H)-one (3k)

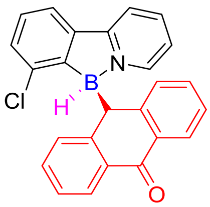

3k

Following the above procedure on 0.1 mmol scale, isolated yield: 59%, (23.2 mg), white solid (mp:

171.6 – 172.9 °C), R_f = 0.3 (silica gel, hexane/EtOAc = 10:1, v/v), column chromatography (silica gel, hexane/EtOAc = 10:1, v/v).

^{1}H NMR (500 MHz, $CDCl_3$) δ 8.30 (dd, *J* = 7.9, 1.4 Hz, 1H), 8.00 (dd, *J* = 4.4, 4.0 Hz, 1H), 7.81 (dd, *J* = 7.8, 1.5 Hz, 1H), 7.79 – 7.75 (m, 1H), 7.72 – 7.69 (m, 1H), 7.53 (dd, *J* = 7.8, 0.7 Hz, 1H), 7.51 – 7.49 (m, 1H), 7.45 – 7.40 (m, 2H), 7.28 (t, *J* = 7.7 Hz, 1H), 7.03 – 7.00 (m, 1H), 6.96 (d, *J* = 5.7 Hz, 1H), 6.91 – 6.88 (m, 1H), 6.85 – 6.81 (m, 1H), 6.44 – 6.40 (m, 1H), 5.04 (s, 1H).

^{13}C NMR (126 MHz, $CDCl_3$) δ 185.2, 156.0, 150.8, 146.9, 142.7, 140.6, 138.2, 137.2, 132.6, 132.1, 130.8, 130.5, 130.3, 129.5, 128.2, 128.1, 126.9, 126.1, 125.8, 124.8, 123.8, 121.8, 119.7, 117.8.

^{11}B NMR (128 MHz, $CDCl_3$) δ 1.15(d, *J* = 99.7 Hz).

HRMS (ESI) m/z: $[M+H]^+$ Calcd. for $C_{25}H_{17}BClNO$ 394.1165; Found: 394.1162.

HPLC analysis: DAICEL CHIRALCEL AD-H, hexane/isopropanol = 90/10, 1mL/min, λ = 254 nm, t_R (minor) = 11.777 min, t_R (major) = 13.525 min, 91% ee.

$[\alpha]^{25}_{D}$: -114.69 (*c* 0.69, $CHCl_3$).

6(*S*)-benzhydryl-7-fluoro-6H-5λ^4-benzo[3,4][1,2]azaborolo[1,5-a]pyridine (3l)

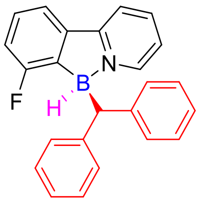

3l

Following the above procedure on 0.1 mmol scale, isolated yield: 85%, (29.8 mg), white solid (mp: 168.5 – 170.6 °C), R_f = 0.3 (silica gel, hexane/EtOAc = 10:1, v/v), column chromatography (silica gel, hexane/EtOAc = 10:1, v/v).

^{1}H NMR (400 MHz, $CDCl_3$) δ 7.80 (t, *J* = 7.6 Hz, 1H), 7.73 (d, *J* = 7.9 Hz, 1H), 7.68 (d, *J* = 5.5 Hz, 1H), 7.40 (d, *J* = 7.4 Hz, 1H), 7.24 (d, *J* = 7.4 Hz, 2H), 7.10 (t, *J* = 7.0 Hz, 3H), 7.03 – 6.91 (m, 4H), 6.91 – 6.78 (m, 4H), 3.75 (d, *J* = 4.0 Hz, 1H).

^{13}C NMR (101 MHz, $CDCl_3$) δ 164.0 (d, *J* = 243Hz), 157.0 (d, *J* = 0.7 Hz), 147.2, 146.7, 143.5, 140.0, 139.2 (d, *J* = 14.5 Hz), 128.8 (d, *J* = 8.1 Hz), 127.64 (d, *J* = 9.8 Hz), 124.1 (d, *J* = 4.1 Hz), 121.2, 118.1, 117.4 (d, *J* = 3.2 Hz), 117.1, 116.8.

^{11}B NMR (128 MHz, $CDCl_3$) δ -0.97 (d, *J* = 96.1 Hz).

^{19}F NMR (376 MHz, CDCl$_3$) δ -105.14 (s).

HRMS (ESI) m/z: $[M+H]^+$ Calcd. for $C_{24}H_{19}BFN$ 352.1667; Found: 352.1661.

HPLC analysis: DAICEL CHIRALCEL AD-H, hexane/isopropanol = 90/10, 1mL/min, λ = 254 nm, t_R (minor) = 5.733 min, t_R (major) = 6.761 min, 92% ee.

$[\alpha]^{25}_D$: -31.39 (*c* 0.55, $CHCl_3$).

6(*S*)-benzhydryl-7-bromo-6H-5λ^4-benzo[3,4][1,2]azaborolo[1,5-a]pyridine (**3m**)

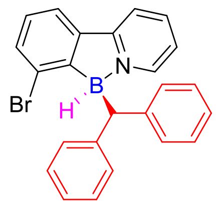

3m

Following the above procedure on 0.1 mmol scale, isolated yield: 93%, (38.2 mg), white solid (mp: 174.3 – 176.8 ^{o}C), R_f = 0.3 (silica gel, hexane/EtOAc = 10:1, v/v), column chromatography (silica gel, hexane/EtOAc = 10:1, v/v).

^{1}H NMR (500 MHz, CDCl$_3$) δ 7.94 (t, *J* = 7.7 Hz, 1H), 7.86 (d, *J* = 5.7 Hz, 1H), 7.78 (d, *J* = 8.2 Hz, 1H), 7.52 (d, *J* = 7.6 Hz, 3H), 7.46 (d, *J* = 7.9 Hz, 1H), 7.29 (t, *J* = 7.6 Hz, 2H), 7.17 – 7.13(m, 2H), 7.01 (t, *J* = 7.7 Hz, 1H), 6.86 – 6.78 (m, 3H), 6.44 (dd, *J* = 7.6, 1.2 Hz, 2H), 4.70 (d, *J* = 2.7 Hz, 1H).

^{13}C NMR (126 MHz, CDCl$_3$) δ 157.2, 147.0, 145.0, 143.5, 140.1, 137.8, 133.8, 129.0, 128.2, 127.9, 127.3, 127.1, 126.0, 124.0, 123.7, 121.3, 120.0, 118.1, 99.9.

^{11}B NMR (128 MHz, CDCl$_3$) δ-0.01 (d, *J* = 89.1 Hz).

HRMS (ESI) m/z: $[M+H]^+$ Calcd. for $C_{24}H_{19}BBrN$ 412.0867; Found: 412.0864.

HPLC analysis: DAICEL CHIRALCEL AD-H, hexane/isopropanol = 90/10, 1mL/min, λ = 254 nm, t_R (minor) = 7.752 min, t_R (major) = 9.173 min, 89% ee.

$[\alpha]^{25}_D$: -96.46 (*c* 0.41, $CHCl_3$).

6(*S*)-benzhydryl-7-chloro-10-fluoro-6H-5λ^4-benzo[3,4][1,2]azaborolo[1,5-a]pyridine (**3n**)

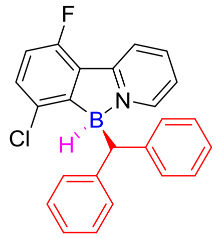

3n

Following the above procedure on 0.1 mmol scale, isolated yield: 79%, (30.4 mg), white solid (mp: 164.4 – 166.7 ^{o}C), R_f = 0.4 (silica gel, hexane/EtOAc = 10:1, v/v), column chromatography (silica gel, hexane/EtOAc = 10:1, v/v).

^{1}H NMR (400 MHz, CDCl$_3$) δ 8.08 (d, *J* = 8.2 Hz, 1H), 7.99 (t, *J* = 7.8 Hz, 1H), 7.86 (d, *J* = 5.7 Hz, 1H), 7.47 (d, *J* = 8.1 Hz, 2H), 7.31 – 7.26 (m, 2H), 7.23 – 7.17 (m, 2H), 7.13 (t, *J* = 7.3 Hz, 1H), 6.89 – 6.82 (m, 3H), 6.77 (dd, *J* = 10.1, 8.6 Hz, 1H), 6.49 – 6.43 (m, 2H), 4.57 (d, *J* = 3.1 Hz, 1H).

^{13}C NMR (101 MHz, CDCl$_3$) δ 158.4 (d, *J* = 153.5Hz), 154.34 (d, *J* = 5.1 Hz), 146.7, 144.9, 143.5, 140.5, 131.9 (d, *J* = 7.4 Hz), 130.9 (d, *J* = 2.6 Hz), 128.9, 128.1 (d, *J* = 20.0 Hz), 127.3, 124.6 (d, *J* = 9.1 Hz),124.1 (d, *J* = 11.4 Hz), 122.0 (d, *J* = 11.0 Hz), 121.4, 114.1, 113.9.

^{11}B NMR (128 MHz, CDCl$_3$) δ -0.29 (d, *J* = 74.0 Hz).

^{19}F NMR (376 MHz, CDCl$_3$) δ -121.19 (s).

HRMS (ESI) m/z: [M+H]$^{+}$ Calcd. for $C_{24}H_{18}BClFN$ 386.1278; Found: 386.1272.

HPLC analysis: DAICEL CHIRALCEL AD-H, hexane/isopropanol = 90/10, 1mL/min, λ = 254 nm, t_R (minor) = 9.554 min, t_R (major) = 10.155 min, 90% ee.

[α]$^{25}_{D}$: -48.53 (*c* 0.52, CHCl$_3$).

6(*S*)-benzhydryl-7-chloro-8-fluoro-6H-5λ^{4}-benzo[3,4][1,2]azaborolo[1,5-a]pyridine (3o)

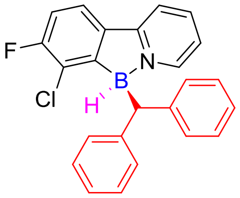

3o

Following the above procedure on 0.1 mmol scale, isolated yield: 95%, (36.6 mg), white solid (mp: 173.1 – 174.9 ^{o}C), R_f = 0.3 (silica gel, hexane/EtOAc = 10:1, v/v), column chromatography (silica gel, hexane/EtOAc = 10:1, v/v).

^{1}H NMR (400 MHz, $CDCl_3$) δ 7.95 (t, *J* = 7.8 Hz, 1H), 7.83 (d, *J* = 5.8 Hz, 1H), 7.76 (d, *J* = 8.1 Hz, 1H), 7.50 – 7.44 (m, 3H), 7.27 (dd, *J* = 10.2, 5.1 Hz, 2H), 7.14 (dt, *J* = 11.9, 5.9 Hz, 2H), 6.93 (t, *J* = 8.7 Hz, 1H), 6.89 – 6.79 (m, 3H), 6.51 – 6.44 (m, 2H), 4.53 (d, *J* = 3.0 Hz, 1H).

^{13}C NMR (101 MHz, $CDCl_3$) δ 159.6 (d, *J* = 252.2 Hz), 156.6, 146.8, 145.0, 143.5, 140.2, 132.9 (d, *J* = 2.8 Hz), 128.9, 128.2, 128.0, 127.3, 124.0 (d, *J* = 17.5 Hz), 122.7 (d, *J* = 16.6 Hz), 120.9 (d, *J* = 8.1 Hz), 117.8, 114.4 (d, *J* = 24.1 Hz).

^{11}B NMR (128 MHz, $CDCl_3$) δ -0.49(d, *J* = 90.0 Hz).

^{19}F NMR (376 MHz, $CDCl_3$) δ -112.52 (s).

HRMS (ESI) m/z: $[M+H]^+$ Calcd. for $C_{24}H_{18}BClFN$ 412.1278; Found: 412.1278.

HPLC analysis: DAICEL CHIRALCEL AD-H, hexane/isopropanol = 70/30, 1mL/min, λ = 254 nm, t_R (minor) = 11.623 min, t_R (major) = 12.964 min, 89% ee.

$[\alpha]^{25}_{D}$: -47.74 (*c* 0.53, $CHCl_3$).

6(*S*)-benzhydryl-7,8-dichloro-6H-5λ^4-benzo[3,4][1,2]azaborolo[1,5-a]pyridine (**3p**)

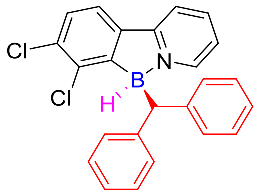

3p

Following the above procedure on 0.1 mmol scale, isolated yield: 82%, (32.9 mg), 6.5:1 dr, white solid (mp: 141.3 – 143.8 ^{o}C), R_f = 0.2 (silica gel, hexane/EtOAc = 10:1, v/v), column chromatography (silica gel, hexane/EtOAc = 10:1, v/v).

^{1}H NMR (500 MHz, $CDCl_3$) δ 7.95 (t, *J* = 7.8 Hz, 1H), 7.84 (d, *J* = 5.7 Hz, 1H), 7.75 (d, *J* = 8.1 Hz, 1H), 7.47 (d, *J* = 1.6 Hz, 1H), 7.46 (d, *J* = 8.1 Hz, 2H), 7.29 (t, *J* = 7.6 Hz, 3H), 7.23 (d, *J* = 8.1 Hz, 1H), 7.18 – 7.13 (m, 3H), 6.87 – 6.82 (m, 3H), 6.47 – 6.42 (m, 2H), 4.59 (d, *J* = 2.9 Hz, 1H).

^{13}C NMR (101 MHz, $CDCl_3$) δ 156.5, 146.8, 144.9, 143.5, 140.3, 135.7, 134.9, 134.3, 128.9, 128.1, 128.1, 128.0, 127.3, 124.1, 123.9, 121.4, 120.2, 118.0.

^{11}B NMR (128 MHz, $CDCl_3$) δ -0.46 (d, *J* = 110.5 Hz).

HRMS (ESI) m/z: $[M+H]^+$ Calcd. for $C_{24}H_{18}BCl_2N$ 402.0982; Found: 402.0985.

HPLC analysis: DAICEL CHIRALCEL AD-H, hexane/isopropanol = 90/10, 1mL/min, λ = 254 nm, t_R (minor) = 9.107 min, t_R (major) = 12.653 min, 91% ee.

$[\alpha]^{25}_{D}$: -162.77 (*c* 0.53, $CHCl_3$).

6(*S*)-benzhydryl-7,9-dichloro-6H-5λ^4-benzo[3,4][1,2]azaborolo[1,5-a]pyridine (3q)

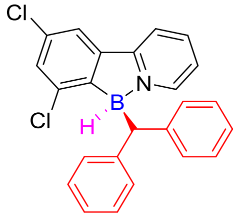

3q

Following the above procedure on 0.1 mmol scale, isolated yield: 90%, (36.1 mg), white solid (mp: 170.0 – 172.5 ^{o}C), R_f = 0.3 (silica gel, hexane/EtOAc = 5:1, v/v), column chromatography (silica gel, hexane/EtOAc = 5:1, v/v).

^{1}H NMR (500 MHz, CDCl$_3$) δ 8.00 – 7.95 (m), 7.85 (d, *J* = 5.7 Hz), 7.77 (d, *J* = 8.1 Hz), 7.46 (dd, *J* = 8.2, 4.8 Hz), 7.27 (dd, *J* = 8.0, 6.4 Hz), 7.21 – 7.18 (m, 1H), 7.13 (t, *J* = 7.3 Hz, 1H), 6.91 – 6.83 (m, 3H), 6.51 – 6.46 (m, 2H), 4.51 (d, *J* = 3.3 Hz, 1H).

^{13}C NMR (101 MHz, CDCl$_3$) δ 156.0, 146.8, 145.1, 143.7, 140.3, 138.7, 137.3, 132.0, 130.2, 128.9, 128.2, 128.0, 127.4, 124.1, 124.0, 121.9, 119.7, 118.3.

^{11}B NMR (128 MHz, CDCl$_3$) δ -0.49 (d, *J* = 71.4 Hz).

HRMS (ESI) m/z: $[M+H]^+$ Calcd. for $C_{24}H_{18}BCl_2N$ 402.0982; Found: 402.0984.

HPLC analysis: DAICEL CHIRALCEL AD-H, hexane/isopropanol = 90/10, 1mL/min, λ = 254 nm, t_R (minor) = 7.658 min, t_R (major) = 8.917 min, 88% ee.

$[\alpha]^{25}_{D}$: -103.60(*c* 0.5, $CHCl_3$).

6(*S*)-benzhydryl-7-chloro-10-methyl-6H-5λ^4-benzo[3,4][1,2]azaborolo[1,5-a]pyridine (3r)

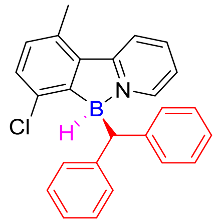

3r

Following the above procedure on 0.1 mmol scale, isolated yield: 95%, (36.2 mg), white solid (mp: 163.4 – 164.6 ^{o}C), R_f = 0.3 (silica gel, hexane/EtOAc = 10:1, v/v), column chromatography (silica gel, hexane/EtOAc = 10:1, v/v).

^{1}H NMR (500 MHz, CDCl$_3$) δ 8.00 – 7.91 (m, 3H), 7.51 (d, *J* = 7.5 Hz, 2H), 7.29 (t, *J* = 7.7 Hz, 2H), 7.19 – 7.10 (m, 3H), 6.88 – 6.79 (m, 4H), 6.43 (dd, *J* = 7.8, 1.4 Hz, 2H), 4.62 (d, *J* = 2.7 Hz, 1H), 2.51 (s, 3H).

^{13}C NMR (126 MHz, CDCl$_3$) δ 157.8, 147.1, 145.0, 143.6, 140.0, 136.2, 133.9, 132.3, 130.3, 130.0, 128.9, 128.1, 127.9, 127.1, 123.9, 123.7, 121.3, 120.5, 21.5.

^{11}B NMR (128 MHz, CDCl$_3$) δ -0.95 (d, *J* = 96.5 Hz).

HRMS (ESI) m/z: $[M+H]^+$ Calcd. for $C_{25}H_{21}BClN$ 382.1528; Found:382.1525.

HPLC analysis: DAICEL CHIRALCEL AD-H, hexane/isopropanol = 90/10, 1mL/min, λ = 254 nm, t_R (minor) = 13.075 min, t_R (major) = 13.895 min, 91% ee.

$[\alpha]^{25}_{D}$: -52.07 (*c* 0.58, CHCl$_3$).

12(*S*)-benzhydryl-12H-11λ^4-naphtho[1',2':3,4][1,2]azaborolo[1,5-a]pyridine (3s)

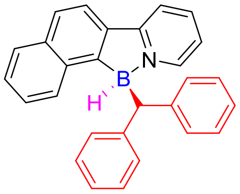

3s

Following the above procedure on 0.1 mmol scale, isolated yield: 90%, (34.5 mg), white solid (mp: 128.8 – 130.2 ^{o}C), R_f = 0.3 (silica gel, hexane/EtOAc = 10:1, v/v), column chromatography (silica gel, hexane/EtOAc = 10:1, v/v).

^{1}H NMR (500 MHz, CDCl$_3$) δ 8.02 (d, *J* = 5.8 Hz, 1H), 7.87 – 7.82 (m, 1H), 7.80 (d, *J* = 8.0 Hz, 2H), 7.70 (s, 2H), 7.65 (d, *J* = 8.2 Hz, 1H), 7.46 – 7.43 (m, 4H), 7.28 – 7.25 (m, 1H), 7.20 (d, *J* = 7.4 Hz, 2H), 7.12 (dd, *J* = 10.7, 4.3 Hz, 2H), 7.09 – 7.03 (m, 2H), 7.00 – 6.90 (m, 3H), 6.81 (dd, *J* = 5.2, 3.2 Hz, 2H), 4.09 (d, *J* = 4.7 Hz, 1H).

^{13}C NMR (101 MHz, CDCl$_3$) δ 158.4, 146.3, 143.3, 139.6, 134.8, 134.1, 133.1, 130.6, 129.3, 128.4, 128.1, 127.7, 127.5, 126.8, 126.7, 125.4, 124.2, 123.9, 119.8, 118.3, 117.4, 46.2.

^{11}B NMR (128 MHz, CDCl$_3$) δ 0.30 (d, *J* = 70.0 Hz).

HRMS (ESI) m/z: $[M+H]^+$ Calcd. for $C_{28}H_{22}BN$ 384.1918; Found: 384.1921.

HPLC analysis: DAICEL CHIRALCEL AD-H, hexane/isopropanol = 90/10, 1mL/min, λ = 254 nm, t_R (minor) = 3.700 min, t_R (major) = 10.276 min, 93% ee.

$[\alpha]^{25}_D$: -14.448 (*c* 0.59, $CHCl_3$).

General procedure B for the boron-stereogenic compounds with 2-arylpyridine-borane and α-diazoarylacetate

In air, a 10 mL schlenk tube was charged with **1** (0.12 mmol, 1.2 equiv), **4** (0.1 mmol, 1 equiv), $Cu(MeCN)_4PF_6$ (5 mol%), **L2** (6 mol%). The tube was evacuated and filled with argon for three cycles. Then, 1 mL of 1,2 - Dichlorethan was added under argon. The reaction was allowed to stir at 0 °C for 20 hours. Upon completion, proper amount of silica gel was added to the reaction mixture. After removal of the solvent, the crude reaction mixture was purified on silica gel (petroleum ether and ethyl acetate) to afford the desired products.

ethyl (*S*)-2-(7-chloro-(*R*)6H-5λ⁴-benzo[3,4][1,2]azaborolo[1,5-a]pyridin-6-yl)-2-phenylacetate (5a)

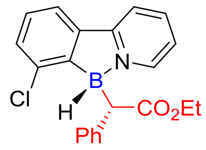

5a

Following the above procedure on 0.1 mmol scale, isolated yield: 95%, (34.5 mg), ＞20:1 dr, white solid (mp: 172.5-173.1 °C), R_f = 0.3 (silica gel, hexane/EtOAc = 10:1, v/v), column chromatography (silica gel, hexane/EtOAc = 10:1, v/v).

^{1}H NMR (500 MHz, $CDCl_3$) δ 8.95 (d, *J* = 5.8 Hz, 1H), 7.92 (t, *J* = 7.8 Hz, 1H), 7.63 (d, *J* = 8.1 Hz, 1H), 7.42 – 7.37 (m, 3H), 7.15 (t, *J* = 7.7 Hz, 1H), 6.75 – 6.72 (m, 3H), 6.70 – 6.68(m, 2H), 4.34 – 4.23 (m, 2H), 4.16 (s, 1H), 1.37 (t, *J* = 7.1 Hz, 3H).

^{13}C NMR (126 MHz, $CDCl_3$) δ 178.6, 157.0, 145.3, 140.4, 139.6, 138.1, 136.8, 130.8, 127.7, 126.9, 126.4, 123.8, 121.5, 119.5, 117.7, 59.8, 14.5.

^{11}B NMR (128 MHz, $CDCl_3$) δ -2.02 (d, *J* = 89.3 Hz).

HRMS (ESI) m/z: $[M+H]^+$ Calcd. for $C_{21}H_{19}BClNO_2$ 364.1270; Found: 364.1271.

HPLC analysis: DAICEL CHIRALCEL AD-H, hexane/isopropanol = 90/10, 1mL/min, λ = 254 nm, t_R (minor) = 7.82 min, t_R (major) = 10.02 min, 98% ee.

$[\alpha]^{25}_D$: -16.8 (*c* 0.5, $CHCl_3$).

ethyl 2-(7-chloro-6H-5λ^4-benzo[3,4][1,2]azaborolo[1,5-a]pyridin-6-yl)-2-phenylacetate (5a')

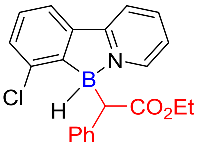

5a'

^{1}H NMR (400 MHz, $CDCl_3$) δ 7.98 (t, *J* = 7.7 Hz, 1H), 7.89 (d, *J* = 7.6 Hz, 1H), 7.68 (d, *J* = 7.6 Hz, 1H), 7.53 – 7.45 (m, 3H), 7.43 (d, *J* = 7.8 Hz, 1H), 7.35 – 7.27 (m, 3H), 7.19 – 7.13 (m, 2H), 4.40 (d, *J* = 3.4 Hz, 1H), 3.56 – 3.35 (m, 2H), 0.74 (t, *J* = 7.1 Hz, 3H).

^{11}B NMR (128 MHz, $CDCl_3$) δ -1.10 (d, *J* = 107.3 Hz).

^{13}C NMR (101 MHz, $CDCl_3$) δ 174.9, 156.9, 143.8, 141.6, 140.6, 138.3, 137.6, 130.5, 128.0, 127.9 127.9, 124.5, 121.1, 119.6, 117.9, 58.6, 13.7.

ethyl (S)-2-(7-fluoro-6H-5λ^4-benzo[3,4][1,2]azaborolo[1,5-a]pyridin-6-yl)-2-phenylacetate (**5b**)

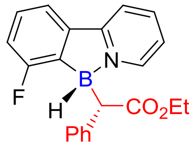

5b

Following the above procedure on 0.1 mmol scale, isolated yield: 84%, (29.2 mg), ＞20:1 dr, white solid (mp: 140.0 – 142.7 ^{o}C), R_f = 0.3 (silica gel, hexane/EtOAc = 5:1, v/v), column chromatography (silica gel, hexane/EtOAc = 5:1, v/v).

^{1}H NMR (400 MHz, $CDCl_3$) δ 8.56 (d, *J* = 5.2 Hz, 1H), 7.94 (t, *J* = 7.7 Hz, 1H), 7.73 (d, *J* = 7.9 Hz, 1H), 7.42 (d, *J* = 7.5 Hz, 1H), 7.32 (t, *J* = 6.6 Hz, 1H), 7.23 (dd, *J* = 12.4, 7.2 Hz, 1H), 7.08 (t, *J* = 7.9 Hz, 1H), 6.94 – 6.82 (m, 5H), 4.24 – 4.18 (m, 2H), 3.63 (s, 1H), 1.30 (t, *J* = 7.1 Hz, 3H).

^{13}C NMR (101 MHz, $CDCl_3$) δ 178.0, 164.1(d, *J* = 242.3 Hz), 157.1, 144.9, 140.5, 140.1, 139.2 (d, *J* = 14.1 Hz), 128.3 (d, *J* = 7.0 Hz), 127.2 (d, *J* = 9.5 Hz), 124.3, 121.5, 117.9, 117.5 (d, *J* = 3.2 Hz), 117.2, 116.9, 99.9, 59.8, 14.4.

^{11}B NMR (128 MHz, $CDCl_3$) δ -2.15 (d, *J* = 80.8 Hz).

^{19}F NMR (376 MHz, CDCl$_3$) δ -107.38 (s).

HRMS (ESI) m/z: $[M+H]^+$ Calcd. for $C_{21}H_{19}BFNO_2$ 348.1566; Found:348.1567.

HPLC analysis: DAICEL CHIRALCEL AD-H, hexane/isopropanol = 90/10, 1mL/min, λ = 254 nm, t_R (minor) = 12.019 min, t_R (major) = 13.027 min, 94% ee.

$[\alpha]^{25}_D$: -53.59 (*c* 0.52, CHCl$_3$).

ethyl(*S*)-2-(8-fluoro-(*R*)6H-5λ^4-benzo[3,4][1,2]azaborolo[1,5-a]pyridin-6-yl)-2-phenylacetate (5c)

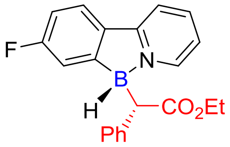

5c

Following the above procedure on 0.1 mmol scale, isolated yield: 76%, (26.4 mg), 3.3:1 dr, white solid (mp: 93.8 – 94.2 °C), R_f = 0.3 (silica gel, hexane/EtOAc = 10:1, v/v), column chromatography (silica gel, hexane/EtOAc = 10:1, v/v).

^{1}H NMR (500 MHz, CDCl$_3$) δ 8.35 (d, *J* = 5.8 Hz, 1H), 7.94 – 7.90 (m, 1H), 7.73 (dd, *J* = 8.2, 0.8 Hz, 1H), 7.65 (dd, *J* = 8.4, 4.7 Hz, H), 7.32 (dd, *J* = 8.8, 2.4 Hz), 7.25 – 7.22 (m, 1H), 7.12 – 7.09 (m, 2H), 7.07 – 7.03 (m, 2H), 7.00 – 6.93 (m, 2H), 4.13 – 4.06 (m, 2H), 3.38 (d, *J* = 4.0 Hz, 1H), 1.19 (t, *J* = 7.1 Hz, 3H).

^{13}C NMR (126 MHz, CDCl$_3$) δ 177.2, 157.2, 144.4, 140.4 (d, *J* = 4.9 Hz), 132.3 (d, *J* = 2.0 Hz), 127.9, 127.5, 124.6, 123.1 (d, *J* = 8.9 Hz), 120.5, 117.3, 116.7 (d, *J* = 20 Hz), 113.7(d, *J* = 24.1 Hz), 99.9, 59.5, 14.3.

^{11}B NMR (128 MHz, CDCl$_3$) δ-1.93 (d, *J* = 78.3 Hz).

^{19}F NMR (471 MHz, CDCl$_3$) δ -109.7.

HRMS (ESI) m/z: $[M+H]^+$ Calcd. for $C_{21}H_{19}BFNO_2$ 348.1566; Found: 348.1562.

HPLC analysis: DAICEL CHIRALCEL OD-H, hexane/isopropanol = 90/10, 1mL/min, λ = 254 nm, t_R (minor) = 10.43 min, t_R (major) = 18.37 min, 90% ee.

$[\alpha]^{25}_D$: -43.6 (*c* 0.32, CHCl$_3$).

ethyl (S)-2-(7-bromo-6H-5λ^4-benzo[3,4][1,2]azaborolo[1,5-a]pyridin-6-yl)-2-phenylacetate (5d)

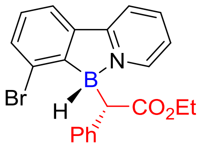

5d

Following the above procedure on 0.1 mmol scale, isolated yield: 81%, (33.0 mg), ＞20:1 dr, white solid (mp: 209.9 – 210.8 °C), R_f = 0.3 (silica gel, hexane/EtOAc = 10:1, v/v), column chromatography (silica gel, hexane/EtOAc = 10:1, v/v).

^{1}H NMR (500 MHz, $CDCl_3$) δ 8.98 (d, *J* = 6.4 Hz, 1H), 7.94 – 7.90(m, 1H), 7.67 – 7.59 (m, 1H), 7.56 (dd, *J* = 7.9, 0.8 Hz, 1H), 7.49 – 7.35 (m, 2H), 7.07 (t, *J* = 7.8 Hz, 1H), 6.77 – 6.69 (m, 3), 6.68 – 6.61 (m, 2H), 4.38 – 4.18 (m, 3H), 1.37 (t, *J* = 7.2 Hz, 3H).

^{13}C NMR (126 MHz, $CDCl_3$) δ 178.6, 157.0, 145.3, 140.4, 139.5, 138.1, 133.9, 127.8, 126.8, 126.3, 126.1, 123.8, 121.5, 120.0, 117.7, 99.9, 59.8, 14.5.

^{11}B NMR (128 MHz, $CDCl_3$) δ -1.52 (d, *J* = 97.2 Hz).

HRMS (ESI) m/z: $[M+H]^+$ Calcd. for $C_{21}H_{19}BBrNO_2$ 408. 0765; Found:408.0762.

HPLC analysis: DAICEL CHIRALCEL AD-H, hexane/isopropanol = 90/10, 1mL/min, λ = 254 nm, t_R (minor) = 7.802 min, t_R (major) = 10.446 min, 97% ee.

$[\alpha]^{25}_D$: -6.40 (*c* 0.5, $CHCl_3$).

ethyl(S)-2-(7-chloro-8-fluoro-6H-5l4-benzo[3,4][1,2]azaborolo[1,5-a]pyridin-6-yl)-2-phenylacetate (5e)

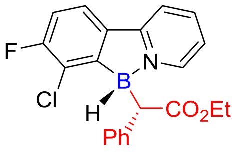

5e

Following the above procedure on 0.1 mmol scale, isolated yield: 88%, (33.5 mg), ＞20:1 dr, white solid (mp: 213.2 – 214.6 °C), R_f = 0.3 (silica gel, hexane/EtOAc = 10:1, v/v), column chromatography (silica gel, hexane/EtOAc = 10:1, v/v).

^{1}H NMR (400 MHz, $CDCl_3$) δ 8.93 (d, *J* = 5.6 Hz, 1H), 7.94 (t, *J* = 7.7 Hz, 1H), 7.59 (d, *J* = 8.1 Hz, 1H), 7.39 (dd, *J* = 12.6, 5.4 Hz, 2H), 6.98 (t, *J* = 8.6 Hz, 1H), 6.75 (s, 3H), 6.68 (s, 2H), 4.36 – 4.21 (m, 2H), 4.11 (s, 1H), 1.36 (t, *J* = 7.0 Hz, 3H).

^{13}C NMR (101 MHz, $CDCl_3$) δ 178.4, 161.0, 159.7(d, *J* = 252.6 Hz), 145.3, 140.6, 139.4, 133.2 (d, *J* =

2.9 Hz), 127.0, 126.3, 124.0, 122.8 (d, J = 17.1 Hz), 121.2, 121.0 (d, J = 8.0 Hz), 117.4, 114.9 (d, J = 23.9Hz), 59.9, 14.5.

^{11}B NMR (128 MHz, $CDCl_3$) δ-2.34 (d, J = 80.9 Hz).

^{19}F NMR (376 MHz, $CDCl_3$) δ -112.44 (s).

HRMS (ESI) m/z: $[M+H]^+$ Calcd. for $C_{21}H_{18}BClFNO_2$ 382.1176; Found:382.1179.

HPLC analysis: DAICEL CHIRALCEL AD-H, hexane/isopropanol = 90/10, 1mL/min, λ = 254 nm, t_R (minor) = 8.444 min, t_R (major) = 13.535 min, 93% ee.

$[α]^{25}_D$: -27.26 (c 0.51, $CHCl_3$).

ethyl (S)-2-(7,8-dichloro-6H-5λ^4-benzo[3,4][1,2]azaborolo[1,5-a]pyridin-6-yl)-2-phenylacetate (**5f**)

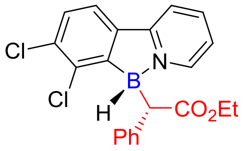

5f

Following the above procedure on 0.1 mmol scale, isolated yield: 95%, (37.7 mg), ＞20:1 dr, white solid (mp: 218.6 – 219.4 °C), R_f = 0.3 (silica gel, hexane/EtOAc = 10:1, v/v), column chromatography (silica gel, hexane/EtOAc = 10:1, v/v).

^{1}H NMR (400 MHz, $CDCl_3$) δ 8.93 (d, J = 5.7 Hz, 1H), 7.94 (t, J = 7.8 Hz, 1H), 7.60 (d, J = 8.1 Hz, 1H), 7.42 (t, J = 6.6 Hz, 1H), 7.34 – 7.27 (m, 2H), 6.74 (d, J = 2.8 Hz, 3H), 6.65 (d, J = 1.5 Hz, 2H), 4.34 – 4.21 (m, 2H), 4.13 (s, 1H), 1.36 (t, J = 7.1 Hz, 3H).

^{13}C NMR (101 MHz, $CDCl_3$) δ 178.4, 156.3, 145.3, 140.6, 139.4, 136.0, 135.0, 134.4, 128.6, 127.0, 126.3, 124.0, 121.6, 120.2, 117.7, 59.9, 14.5.

^{11}B NMR (128 MHz, $CDCl_3$) δ -1.92 (d, J = 58.4 Hz).

HRMS (ESI) m/z: $[M+Na]^+$ Calcd. for $C_{21}H_{18}BCl_2NO_2$ 420.0700; Found:420.0698.

HPLC analysis: DAICEL CHIRALCEL AD-H, hexane/isopropanol = 90/10, 1mL/min, λ = 254 nm, t_R (minor) = 7.922 min, t_R (major) = 12.211 min, 93% ee.

$[α]^{25}_D$: -54.10 (c 0.52, $CHCl_3$).

ethyl(S)-2-(7-chloro-8-methyl-6H-5λ^4-benzo[3,4][1,2]azaborolo[1,5-a]pyridin-6-yl)-2-phenylacetat-e (**5g**)

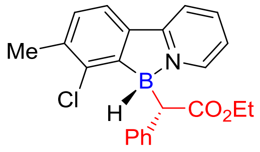

5g

Following the above procedure on 0.1 mmol scale, isolated yield: 75%, (28.3 mg), >20:1 dr, white solid (mp: 210.3 – 212.6 °C), R_f = 0.3 (silica gel, hexane/EtOAc = 10:1, v/v), column chromatography (silica gel, hexane/EtOAc = 10:1, v/v).

^{1}H NMR (400 MHz, $CDCl_3$) δ 8.90 (d, *J* = 5.7 Hz, 1H), 7.88 (t, *J* = 7.8 Hz, 1H), 7.57 (d, *J* = 8.1 Hz, 1H), 7.38 – 7.29 (m, 2H), 7.09 (d, *J* = 7.6 Hz, 1H), 6.73 (d, *J* = 2.2 Hz, 3H), 6.68 (s, 2H), 4.35 – 4.22 (m, 2H), 4.18 (s, 1H), 2.48 (s, 3H), 1.37 (t, *J* = 7.1 Hz, 3H).

^{13}C NMR (101 MHz, $CDCl_3$) δ 178.7, 157.3, 145.1, 140.2, 139.7, 138.7, 136.9, 135.9, 129.3 126.8, 126.4, 123.8, 121.0, 119.3, 117.3, 59.8, 20.6, 14.5.

^{11}B NMR (128 MHz, $CDCl_3$) δ -1.95 (d, *J* = 78.2 Hz).

HRMS (ESI) m/z: $[M+H]^+$ Calcd. for $C_{22}H_{21}BClNO_2$ 378.1427; Found:378.0430.

HPLC analysis: DAICEL CHIRALCEL AD-H, hexane/isopropanol = 90/10, 1mL/min, λ = 254 nm, t_R (minor) = 6.551 min, t_R (major) = 8.386 min, 98% ee.

$[\alpha]^{25}_D$: -31.77 (*c* 0.51, $CHCl_3$).

ethyl(*S*)-2-(7,9-dichloro-(*R*)6H-5λ^4-benzo[3,4][1,2]azaborolo[1,5-a]pyridin-6-yl)-2-phenylacetate (5h)

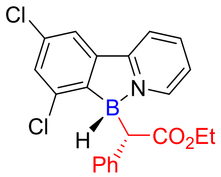

5h

Following the above procedure on 0.1 mmol scale, isolated yield: 88%, (34.9 mg), >20:1 dr, white solid (mp: 115.8 – 117.3 °C), R_f = 0.3 (silica gel, hexane/EtOAc = 10:1, v/v), column chromatography (silica gel, hexane/EtOAc = 10:1, v/v).

^{1}H NMR NMR (500 MHz, $CDCl_3$) δ 8.97 (d, *J* = 5.8 Hz, 1H), 7.94 (t, *J* = 7.8 Hz, 1H), 7.58 (dd, *J* = 8.1, 1.0 Hz, 1H), 7.46 – 7.43 (m, 1H), 7.38 (dd, *J* = 7.0, 1.4 Hz, 2H), 6.75 (q, *J* = 3.7 Hz, 3H), 6.67 (dd, *J* = 6.7, 2.9 Hz, 2H), 4.31 – 4.23 (m, 2H), 4.10 (s, 1H), 1.36 (t, *J* = 7.1 Hz, 3H).

^{13}C NMR (126 MHz, $CDCl_3$) δ 178.4, 155.8, 145.5, 140.7, 139.3, 139.0, 137.3, 132.7, 130.4, 127.0, 126.3, 124.1, 122.2, 119.7, 117.9, 59.9, 14.5.

^{11}B NMR (128 MHz, $CDCl_3$) δ -1.80 (s).

HRMS (ESI) m/z: $[M+H]^+$ Calcd. for $C_{21}H_{19}BCl_2NO_2$ 398.0881; Found: 398.0887.

HPLC analysis: DAICEL CHIRALCEL AZ-3, hexane/isopropanol = 90/10, 1mL/min, λ = 254 nm, t_R (minor) = 23.61 min, t_R (major) = 28.63 min, 96% ee.

$[\alpha]^{25}_D$: + 3.7 (*c* 0.5, $CHCl_3$).

ethyl (S)-2-(7,10-dichloro-6H-5λ^4-benzo[3,4][1,2]azaborolo[1,5-a]pyridin-6-yl)-2-phenylacetate (5i)

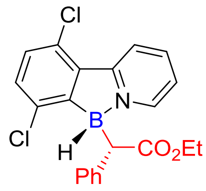

5i

Following the above procedure on 0.1 mmol scale, isolated yield: 88%, (34.9 mg), >20:1 dr, white solid (mp: 126.2 – 127.0 °C), R_f = 0.3 (silica gel, hexane/EtOAc = 5:1, v/v), column chromatography (silica gel, hexane/EtOAc = 5:1, v/v).

^{1}H NMR (400 MHz, $CDCl_3$) δ 8.97 (d, *J* = 5.7 Hz, 1H), 8.56 (d, *J* = 8.4 Hz, 1H), 7.99 (t, *J* = 7.9 Hz, 1H), 7.48 (t, *J* = 6.6 Hz, 1H), 7.30 (d, *J* = 8.3 Hz, 1H), 7.13 (d, *J* = 8.3 Hz, 1H), 6.75 (d, *J* = 5.2 Hz, 3H), 6.61 (d, *J* = 4.9 Hz, 2H), 4.30 – 4.24 (m, 2H), 4.12 (s, 1H), 1.36 (t, *J* = 7.1 Hz, 3H).

^{13}C NMR (101 MHz, $CDCl_3$) δ 178.3, 155.4, 145.4, 140.6, 139.2, 134.7, 134.1, 131.2, 129.6, 128.4, 127.0, 126.2, 124.1, 121.9, 121.7, 59.9, 14.5.

^{11}B NMR (128 MHz, $CDCl_3$) δ-2.34 (d, *J* = 80.9 Hz).

HRMS (ESI) m/z: $[M+Na]^+$ Calcd. for $C_{21}H_{18}BCl_2NO_2$ 420.0700; Found: 420.0698.

HPLC analysis: DAICEL CHIRALCEL AD-H, hexane/isopropanol = 90/10, 1mL/min, λ = 254 nm, t_R (minor) = 11.492 min, t_R (major) = 12.596 min, 98% ee.

$[\alpha]^{25}_D$: -87.61 (*c* 0.53, $CHCl_3$).

ethyl(S)-2-(7-chloro-10-fluoro-6H-5l4-benzo[3,4][1,2]azaborolo[1,5-a]pyridin-6-yl)-2-phenylaceta-te (**5j**)

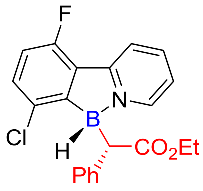

5j

Following the above procedure on 0.1 mmol scale, isolated yield: 85%, (32.4 mg), >20:1 dr, white solid (mp: 138.3 – 138.8 °C), R_f = 0.3 (silica gel, hexane/EtOAc = 10:1, v/v), column chromatography (silica gel, hexane/EtOAc = 10:1, v/v).

^{1}H NMR (400 MHz, $CDCl_3$) δ 8.98 (d, *J* = 5.7 Hz, 1H), 7.97 (t, *J* = 7.8 Hz, 1H), 7.90 (d, *J* = 8.1 Hz, 1H), 7.45 (t, *J* = 6.5 Hz, 1H), 7.33 (dd, *J* = 8.5, 3.8 Hz, 1H), 6.83 (t, *J* = 9.3 Hz, 1H), 6.79 – 6.74 (m, 3H), 6.67 (d, *J* = 4.7 Hz, 2H), 4.35 – 4.21 (m, 2H), 4.14 (s, 1H), 1.36 (t, *J* = 7.1 Hz, 3H).

^{13}C NMR (101 MHz, $CDCl_3$) δ 178.4, 158.2 (d, *J* = 254.0 Hz), 154.09 (d, *J* = 5.1 Hz), 145.3, 140.8, 139.3, 132.10 (d, *J* = 7.4 Hz), 131.02 (d, *J* = 2.6 Hz), 127.0, 126.3, 124.9(d, *J* = 9.1 Hz), 124.1, 121.7, 121.55 (d, *J* = 10.9 Hz), 114.7 (d, *J* = 22 Hz), 59.9, 14.5.

^{11}B NMR (128 MHz, $CDCl_3$) δ -1.80 (d, *J* = 76.7 Hz).

^{19}F NMR (376 MHz, $CDCl_3$) δ -120.89 (s).

HRMS (ESI) m/z: $[M+H]^+$ Calcd. for $C_{21}H_{18}BClFNO_2$ 382.1176; Found: 382.1169.

HPLC analysis: DAICEL CHIRALCEL AD-H, hexane/isopropanol = 90/10, 1mL/min, λ = 254 nm, t_R (minor) = 5.889 min, t_R (major) = 6.324 min, 98% ee.

$[\alpha]^{25}_D$: -31.39 (*c* 0.55, $CHCl_3$).

ethyl(S)-2-(7-chloro-10-methyl-6H-5λ^4-benzo[3,4][1,2]azaborolo[1,5-a]pyridin-6-yl)-2-phenylacet-ate (**5k**)

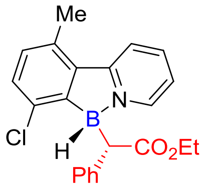

5k

Following the above procedure on 0.1 mmol scale, isolated yield: 67%, (25.3 mg), >20:1 dr, white solid (mp: 155.8 – 156.7 °C), R_f = 0.3 (silica gel, hexane/EtOAc = 10:1, v/v), column chromatography

(silica gel, hexane/EtOAc = 10:1, v/v).

^{1}H NMR (400 MHz, CDCl$_3$) δ 8.94 (d, *J* = 5.7 Hz, 1H), 7.93 (t, *J* = 7.9 Hz, 1H), 7.79 (d, *J* = 8.3 Hz, 1H), 7.40 (t, *J* = 6.6 Hz, 1H), 7.26 (d, *J* = 7.8 Hz, 1H), 6.91 (d, *J* = 7.9 Hz, 1H), 6.71 (d, *J* = 3.9 Hz, 3H), 6.61 (d, *J* = 4.6 Hz, 2H), 4.30 – 4.24(m, 2H), 4.14 (s, 1H), 2.42 (s, 3H), 1.36 (t, *J* = 7.1 Hz, 3H).

^{13}C NMR (101 MHz, CDCl$_3$) δ 178.6, 157.6, 145.5, 140.3, 139.5, 136.5, 134.0, 132.5, 130.8, 130.2, 126.7, 126.3, 123.8, 120.9, 120.8, 59.7, 21.4, 14.5.

^{11}B NMR (128 MHz, CDCl$_3$) δ-2.45 (d, *J* = 75.4 Hz).

HRMS (ESI) m/z: $[M+H]^+$ Calcd. for $C_{22}H_{21}BClNO_2$ 378.1427; Found: 378.1426.

HPLC analysis: DAICEL CHIRALCEL AD-H, hexane/isopropanol = 90/10, 1mL/min, λ = 254 nm, t_R (minor) = 6.506 min, t_R (major) = 8.169 min, 99% ee.

$[\alpha]^{25}_{D}$: -70.78 (*c* 0.51, CHCl$_3$).

ethyl(S)-2-phenyl-2-(7-(trifluoromethoxy)-6H-5λ^4-benzo[3,4][1,2]azaborolo[1,5-a]pyridin-6-yl)acetate (5l)

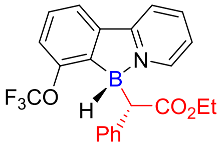

5l

Following the above procedure on 0.1 mmol scale, isolated yield: 73%, (30.2 mg), ＞20:1 dr, white solid (mp: 164.4 – 165.2 °C), R_f = 0.3 (silica gel, hexane/EtOAc = 10:1, v/v), column chromatography (silica gel, hexane/EtOAc = 10:1, v/v).

^{1}H NMR (400 MHz, CDCl$_3$) δ 8.77 (d, *J* = 5.7 Hz, 1H), 7.88 (t, *J* = 7.7 Hz, 1H), 7.65 (d, *J* = 8.0 Hz, 1H), 7.30 (t, *J* = 6.6 Hz, 1H), 7.25 – 7.16 (m, 2H), 6.90 (d, *J* = 7.1 Hz, 1H), 6.78 (s, 5H), 4.24 (q, *J* = 7.1 Hz, 2H), 4.00 (s, 3H), 3.92 (s, 1H), 1.33 (t, *J* = 7.1 Hz, 3H).

^{13}C NMR (101 MHz, CDCl$_3$) δ 178.7, 160.9, 158.0, 144.9, 140.13 (d, *J* = 13.6 Hz), 137.8, 128.0, 126.78 (d, *J* = 7.3 Hz), 123.8, 120.8, 117.6, 114.0, 111.9, 59.6, 55.4, 14.5.

^{11}B NMR (128 MHz, CDCl$_3$) δ -2.11 (d, *J* = 60.2 Hz).

^{19}F NMR (376 MHz, CDCl$_3$) δ -56.49 (s).

HRMS (ESI) m/z: $[M+H]^+$ Calcd. for $C_{22}H_{19}BF_3NO_3$ 414.1483; Found: 414.1479

HPLC analysis: DAICEL CHIRALCEL AD-H, hexane/isopropanol = 90/10, 1mL/min, λ = 254 nm, t_R (minor) = 12.786 min, t_R (major) = 14.662 min, 99% ee.

[α]$^{25}_{D}$: 13.33 (*c* 0.51, $CHCl_3$).

ethyl(*S*)-2-(7,9-dimethyl-(*R*)6H-5λ^4-benzo[3,4][1,2]azaborolo[1,5-a]pyridin-6-yl)-2-phenylacetate (5m)

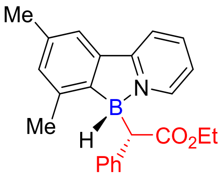

5m

Following the above procedure on 0.1 mmol scale, isolated yield: 55%, (19.6 mg), 6.5:1 dr, white solid (mp: 131.1-132.3°C), R_f = 0.3 (silica gel, hexane/EtOAc = 10:1, v/v), column chromatography (silica gel, hexane/EtOAc = 10:1, v/v).

^{1}H NMR (400 MHz, $CDCl_3$) δ 8.82 (d, *J* = 5.7 Hz, 1H), 7.81 (t, *J* = 7.8 Hz, 1H), 7.57 (d, *J* = 8.1 Hz, 1H), 7.25 (t, *J* = 6.6 Hz, 1H), 7.19 (s, 1H), 7.06 (s, 1H), 6.74 (s, 3H), 6.71 – 6.64 (m, 2H), 4.29 – 4.19 (m, 2H), 3.78 (s, 1H), 2.59 (s, 3H), 2.32 (s, 3H), 1.33 (t, *J* = 7.1 Hz, 3H).

^{13}C NMR (126 MHz, $CDCl_3$) δ 178.8, 158.4, 145.0, 139.9, 139.7, 139.6, 136.7, 135.8, 132.7, 126.8, 126.4, 123.7, 120.4, 119.0, 117.2, 99.9, 59.6, 21.2, 21.1, 14.5.

^{11}B NMR (128 MHz, $CDCl_3$) δ -2.19 (d, *J* = 83.8 Hz).

HRMS (ESI) m/z: $[M+H]^+$ Calcd. for $C_{23}H_{24}BNO_2$ 358.1973; Found: 358.1981.

HPLC analysis: DAICEL CHIRALCEL AS-H, hexane/isopropanol = 98/2, 1mL/min, λ = 254 nm, t_R (minor) = 7.97 min, t_R (major) =8.83 min, 98% ee.

[α]$^{25}_{D}$: -15.0 (*c* 1.2, $CHCl_3$).

ethyl (*S*)-2-((*R*)9H-8λ^4-thieno[2',3':3,4][1,2]azaborolo[1,5-a]pyridin-9-yl)-2-phenylacetate (5n)

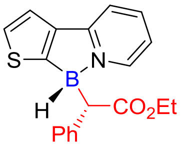

5n

Following the above procedure on 0.1 mmol scale, isolated yield: 82%, (27.5 mg), 5:1 dr, white solid (mp: 150.2 – 151.5 °C), R_f = 0.3 (silica gel, hexane/EtOAc = 10:1, v/v), column chromatography (silica gel, hexane/EtOAc = 10:1, v/v).

^{1}H NMR (500 MHz, CDCl$_3$) δ 8.10 (d, *J* = 5.8 Hz, 1H), 7.85 (t, *J* = 7.7 Hz, 1H), 7.52 (d, *J* = 8.1 Hz, 1H), 7.44 (d, *J* = 4.8 Hz, 1H), 7.33 – 7.29 (m, 3H), 7.19 (dd, *J* = 10.5, 4.8 Hz, 2H), 7.11 – 7.05 (m, 2H), 4.06 – 4.01 (m, 2H), 3.38 (d, *J* = 4.7 Hz, 1H), 1.13 (t, *J* = 7.1 Hz, 3H).

^{13}C NMR (126 MHz, CDCl$_3$) δ 176.4, 154.4, 144.4, 141.0, 140.7, 140.5, 131.6, 128.4, 127.7, 124.9, 119.1, 118.5, 117.1, 59.5, 14.3.

^{11}B NMR (128 MHz, CDCl$_3$) δ -2.21 (d, *J* = 81.3 Hz).

HRMS (ESI) m/z: $[M+H]^+$ Calcd. for $C_{19}H_{18}BNO_2S$ 336.1224; Found: 336.1220.

HPLC analysis: DAICEL CHIRALCEL AD-H, hexane/isopropanol = 90/10, 1mL/min, λ = 254 nm, t_R (minor) = 20.55 min, t_R (major) =23.54 min, 93% ee.

$[\alpha]^{25}_{D}$: - 116.0 (*c* 1.76, CHCl$_3$).

ethyl(*S*)-2-((*R*)6H-7λ^4-benzo[4',5']thieno[2',3':3,4][1,2]azaborolo[1,5-a]pyridin-6-yl)-2-phenylacetate (5o)

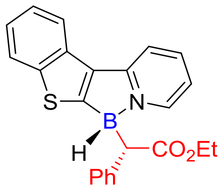

5o

Following the above procedure on 0.1 mmol scale, isolated yield: 75%, (28.9 mg), 5.5:1 dr, yellow solid (mp: 121.1-121.8 ^{o}C), R_f = 0.3 (silica gel, hexane/EtOAc = 10:1, v/v), column chromatography (silica gel, hexane/EtOAc = 10:1, v/v).

^{1}H NMR (400 MHz, CDCl$_3$) δ 8.18 (d, *J* = 5.7 Hz, 1H), 7.94 (dd, *J* = 13.9, 7.8 Hz, 3H), 7.85 (d, *J* = 8.1 Hz, 1H), 7.42 (t, *J* = 7.5 Hz, 1H), 7.33 (dd, *J* = 14.3, 7.4 Hz, 3H), 7.19 (t, *J* = 7.4 Hz, 2H), 7.09 (t, *J* = 6.5 Hz, 2H), 4.05 (q, *J* = 7.0 Hz, 2H), 3.45 (d, *J* = 3.7 Hz, 1H), 1.11 (t, *J* = 7.1 Hz, 3H).

^{13}C NMR (101 MHz, CDCl$_3$) δ 176.4, 154.4, 147.1, 144.6, 140.6, 140.5, 134.1, 133.5, 128.4, 127.8, 125.0, 124.5, 123.4, 123.3, 120.2, 118.2, 117.1, 59.6, 14.3.

^{11}B NMR (128 MHz, CDCl$_3$) δ -2.31 (s).

HRMS (ESI) m/z: $[M+H]^+$ Calcd. for $C_{23}H_{20}BNO_2S$ 386.1381; Found: 386.1383.

HPLC analysis: DAICEL CHIRALCEL AS-H, hexane/isopropanol = 90/10, 1mL/min, λ = 254 nm, t_R (minor) = 11.54 min, t_R (major) = 28.35 min, 93% ee.

[α]^{25}$_{D}$: - 62.1 (*c* 0.96, $CHCl_3$).

ethyl (*S*)-2-(1-fluoro-(*R*)6H-5λ^4-benzo[3,4][1,2]azaborolo[1,5-a]pyridin-6-yl)-2-phenylacetate (5p)

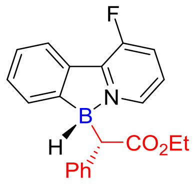

5p

Following the above procedure on 0.1 mmol scale, isolated yield: 70%, (24.3 mg), 3:1 dr, white solid (mp: 75.3 – 76.1 °C), R_f = 0.3 (silica gel, hexane/EtOAc = 10:1, v/v), column chromatography (silica gel, hexane/EtOAc = 10:1, v/v).

^{1}H NMR (500 MHz, $CDCl_3$) δ 8.31 (d, *J* = 5.6 Hz, 1H), 7.96 (dd, *J* = 7.3, 0.5 Hz, 1H), 7.71 (d, *J* = 7.3 Hz, 1H), 7.67 (t, *J* = 8.9 Hz, 1H), 7.48 – 7.45 (m, 1H), 7.33 – 7.30 (m, 1H), 7.25 – 7.22 (m, 1H), 7.08 – 7.06 (m, 2H), 7.05 – 7.00 (m, 2H), 6.99 – 6.95 (m, 1H), 4.14 – 4.07 (m, 2H), 3.41 (d, *J* = 3.7 Hz, 1H), 1.20 (t, *J* = 7.1 Hz, 3H).

^{13}C NMR (151 MHz, $CDCl_3$) δ 177.3, 157.1, 155.4, 148.1, 147.9, 140.84 (d, *J* = 4.6 Hz), 140.3 134.27 (d, *J* = 5.1 Hz), 130.6, 129.9, 127.7, 127.4, 126.68 (d, *J* = 18.2 Hz), 126.3, 125.06 (d, *J* = 10.9 Hz), 124.6, 121.01 (d, *J* = 6.3 Hz), 59.5, 14.3.

^{11}B NMR (160 MHz, $CDCl_3$) δ -0.96 (d, *J* = 82.2 Hz).

^{19}F NMR (565 MHz, $CDCl_3$) δ -122.94 (dd, *J* = 9.6, 4.2 Hz).

HRMS (ESI) m/z: $[M+H]^+$ Calcd. for $C_{21}H_{19}BFNO_2$ 348.1566; Found: 348.1568.

HPLC analysis: DAICEL CHIRALCEL AD-H, hexane/isopropanol = 90/10, 1mL/min, λ = 254 nm, t_R (minor) = 10.84 min, t_R (major) = 11.56 min, 91% ee.

[α]^{25}$_{D}$: - 63.0 (*c* 0.42, $CHCl_3$).

methyl (*S*)-2-(7-chloro-6H-5λ^4-benzo[3,4][1,2]azaborolo[1,5-a]pyridin-6-yl)-2-phenylacetate (5q)

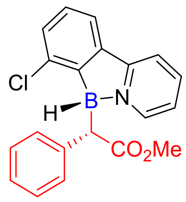

5q

Following the above procedure on 0.1 mmol scale, isolated yield: 64%, (22.3 mg), ＞20:1 dr, white solid (mp: 194.0 – 194.3 °C), R_f = 0.3 (silica gel, hexane/EtOAc = 10:1, v/v), column chromatography (silica gel, hexane/EtOAc = 10:1, v/v).

^{1}H NMR (500 MHz, $CDCl_3$) δ 8.93 (d, *J* = 5.7 Hz, 1H), 7.93 (t, *J* = 7.8 Hz, 1H), 7.66 – 7.62 (m, 1H), 7.44 – 7.37 (m, 3H), 7.16 (t, *J* = 7.7 Hz, 1H), 6.76 – 6.72 (m, 3H), 6.71 – 6.67 (m, 2H), 4.18 (d, *J* = 0.9 Hz, 1H), 3.81 (s, 3H).

^{13}C NMR (101 MHz, $CDCl_3$) δ 179.1, 157.1, 145.2, 140.5, 139.4, 138.1, 136.8, 130.8, 127.8, 126.9, 126.3, 123.9, 121.6, 119.5, 117.8, 51.4.

^{11}B NMR (128 MHz, $CDCl_3$) δ -2.04 (d, *J* = 80.3 Hz).

HRMS (ESI) m/z: $[M+H]^+$ Calcd. for $C_{20}H_{17}BClNO_2$ 350.1114; Found: 350.1111.

HPLC analysis: DAICEL CHIRALCEL AD-H, hexane/isopropanol = 90/10, 1mL/min, λ = 254 nm, t_R (minor) = 17.439 min, t_R (major) = 22.408 min, 98% ee.

$[\alpha]^{25}_D$: -18.30 (*c* 0.55, $CHCl_3$).

benzyl (S)-2-(7-chloro-6H-5λ^4-benzo[3,4][1,2]azaborolo[1,5-a]pyridin-6-yl)-2-phenylacetate (5r)

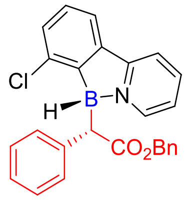

5r

Following the above procedure on 0.1 mmol scale, isolated yield: 97%, (40.1 mg), ＞20:1 dr, white solid (mp: 81.7 – 83.9 °C), R_f = 0.3 (silica gel, hexane/EtOAc = 10:1, v/v), column chromatography (silica gel, hexane/EtOAc = 10:1, v/v).

^{1}H NMR (400 MHz, $CDCl_3$) δ 8.53 (d, *J* = 5.7 Hz, 1H), 7.87 (t, *J* = 7.8 Hz, 1H), 7.59 (d, *J* = 8.1 Hz, 1H), 7.52 (d, *J* = 7.5 Hz, 2H), 7.36 (dd, *J* = 10.8, 7.3 Hz, 4H), 7.33 – 7.23 (m, 2H), 7.12 (t, *J* = 7.7 Hz, 1H), 6.72 (d, *J* = 3.0 Hz, 3H), 6.66 (d, *J* = 4.5 Hz, 2H), 5.28 (dd, *J* = 50.0, 12.5 Hz, 2H), 4.25 (s, 1H).

^{13}C NMR (126 MHz, CDCl$_3$) δ 178.2, 157.0, 145.0, 140.4, 139.4, 138.1, 137.2, 136.8, 130.78, 128.4, 128.1, 127.7, 127.7, 126.9, 126.5, 123.9, 121.5, 119.5, 117.7, 65.6.

^{11}B NMR (128 MHz, CDCl$_3$) δ -2.04 (d, *J* = 59.3 Hz).

HRMS (ESI) m/z: $[M+H]^+$ Calcd. for $C_{26}H_{21}BNO_2$ 426.1427; Found: 426.1425.

HPLC analysis: DAICEL CHIRALCEL AD-H, hexane/isopropanol = 90/10, 1mL/min, λ = 254 nm, t_R (minor) = 17.616 min, t_R (major) = 21.902 min, 98% ee.

$[\alpha]^{25}_D$: -28.48 (*c* 0.57, $CHCl_3$).

benzyl (S)-2-(7-chloro-6H-5λ^4-benzo[3,4][1,2]azaborolo[1,5-a]pyridin-6-yl)-2-phenylacetate(5s)

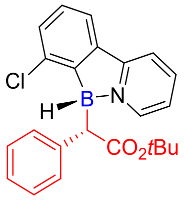

5s

Following the above procedure on 0.1 mmol scale, isolated yield: 83%, (32.5 mg), >20:1 dr, white solid (mp: 188.7 – 189.2 °C), R_f = 0.3 (silica gel, hexane/EtOAc = 10:1, v/v), column chromatography (silica gel, hexane/EtOAc = 10:1, v/v).

^{1}H NMR (400 MHz, CDCl$_3$) δ 9.02 (d, *J* = 5.7 Hz, 1H), 7.92 (t, *J* = 7.7 Hz, 1H), 7.61 (d, *J* = 8.1 Hz, 1H), 7.46 – 7.36 (m, 3H), 7.14 (t, *J* = 7.7 Hz, 1H), 6.70 (d, *J* = 2.4 Hz, 3H), 6.67 (s, 2H), 4.13 (s, 1H), 1.59 (s, 9H).

^{13}C NMR (101 MHz, CDCl$_3$) δ 178.3, 157.0, 145.3, 140.3, 140.0, 138.1, 136.8, 130.7, 127.6, 126.7, 126.3, 123.6, 121.5, 119.4, 117.7, 78.8, 28.4.

^{11}B NMR (128 MHz, CDCl$_3$) δ -2.04 (d, *J* = 77.7 Hz).

HRMS (ESI) m/z: $[M+H]^+$ Calcd. for $C_{23}H_{23}BClNO_2$ 392.1583; Found: 392.1581.

HPLC analysis: DAICEL CHIRALCEL AD-H, hexane/isopropanol = 90/10, 1mL/min, λ = 254 nm, t_R (minor) = 4.994 min, t_R (major) = 5.414 min, 99% ee.

$[\alpha]^{25}_D$: -28.01 (*c* 0.52, $CHCl_3$).

phenyl (S)-2-(7-chloro-6H-5λ^4-benzo[3,4][1,2]azaborolo[1,5-a]pyridin-6-yl)-2-phenylacetate(5t)

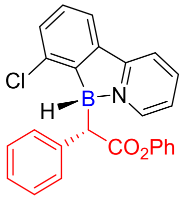

5t

Following the above procedure on 0.1 mmol scale, isolated yield: 90%, (37.0 mg), >20:1 dr, white solid (mp: 199.3 – 200.6 °C), R_f = 0.3 (silica gel, hexane/EtOAc = 10:1, v/v), column chromatography (silica gel, hexane/EtOAc = 10:1, v/v).

^{1}H NMR (400 MHz, $CDCl_3$) δ 8.90 (d, *J* = 5.7 Hz, 1H), 7.90 (t, *J* = 7.7 Hz, 1H), 7.61 (d, *J* = 8.1 Hz, 1H), 7.41 (t, *J* = 8.0 Hz, 4H), 7.35 (t, *J* = 6.6 Hz, 1H), 7.23 (d, *J* = 7.8 Hz, 3H), 7.17 (t, *J* = 7.7 Hz, 1H), 6.73 (d, *J* = 3.5 Hz, 5H), 4.48 (s, 1H).

^{13}C NMR (126 MHz, $CDCl_3$) δ 177.5, 157.1, 151.5, 145.1, 140.6, 138.7, 138.2, 136.8, 130.9, 129.3, 127.9, 127.0, 126.2, 125.4, 124.1, 122.2, 121.7, 119.6, 117.8, 99.8.

^{11}B NMR (128 MHz, $CDCl_3$) δ -2.05 (d, *J* = 48.8 Hz).

HRMS (ESI) m/z: $[M+H]^+$ Calcd. for $C_{25}H_{19}BClNO_2$ 412.1270; Found: 412.1276.

HPLC analysis: DAICEL CHIRALCEL AD-H, hexane/isopropanol = 90/10, 1mL/min, λ = 254 nm, t_R (minor) = 14.082 min, t_R (major) = 20.315 min, 97% ee.

$[\alpha]^{25}_D$: -10.39 (*c* 0.52, $CHCl_3$).

ethyl(*S*)-2-(4-(tert-butyl)phenyl)-2-(7-chloro-6H-5λ^4-benzo[3,4][1,2]azaborolo[1,5-a]pyridin-6-yl) acetate (5u)

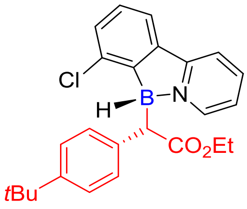

5u

Following the above procedure on 0.1 mmol scale, isolated yield: 93%, (39.0 mg), >20:1 dr, white solid (mp: 206.3 – 207.2 °C), R_f = 0.3 (silica gel, hexane/EtOAc = 10:1, v/v), column chromatography (silica gel, hexane/EtOAc = 10:1, v/v).

^{1}H NMR (500 MHz, $CDCl_3$) δ 8.88 (d, *J* = 5.8 Hz, 1H), 7.93 – 7.88 (m, 1H), 7.60 (d, *J* = 8.1 Hz, 1H), 7.39 – 7.35 (m, 3H), 7.14 (t, *J* = 7.7 Hz, 1H), 6.75 (d, *J* = 8.4 Hz, 2H), 6.60 (d, *J* = 8.3 Hz, 2H), 4.26

(qd, J = 7.1, 3.6 Hz, 2H), 4.08 (s, 1H), 1.35 (t, J = 7.1 Hz, 3H), 1.08 (s, 9H).

^{13}C NMR (126 MHz, $CDCl_3$) δ 178.8, 157.1, 146.5, 145.2, 140.3, 138.2, 136.8, 136.4, 130.7, 127.6, 126.0, 123.7, 121.4, 119.4, 117.6, 99.9, 59.7, 33.8, 31.2, 14.5.

^{11}B NMR (128 MHz, $CDCl_3$) δ -1.96 (d, J = 67.7 Hz).

HRMS (ESI) m/z: $[M+H]^+$ Calcd. for $C_{25}H_{27}BClNO_2$ 420.1896; Found: 420.1899.

HPLC analysis: DAICEL CHIRALCEL AD-H, hexane/isopropanol = 98/2, 1mL/min, λ = 254 nm, t_R (minor) = 13.179 min, t_R (major) = 15.011 min, 99% ee.

$[\alpha]^{25}_D$: - 13.84 (c 0.53, $CHCl_3$).

ethyl (S)-2-(7-chloro-6H-5λ^4-benzo[3,4][1,2]azaborolo[1,5-a]pyridin-6-yl)-2-(p-tolyl)acetate (5v)

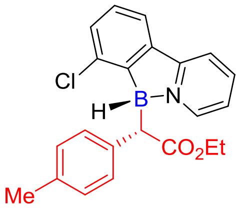

5v

Following the above procedure on 0.1 mmol scale, isolated yield: 71%, (26.8 mg), >20:1 dr, white solid (mp: 157.8 – 158.3 °C),, R_f = 0.3 (silica gel, hexane/EtOAc = 10:1, v/v), column chromatography (silica gel, hexane/EtOAc = 10:1, v/v).

^{1}H NMR (500 MHz, $CDCl_3$) δ 8.96 (d, J = 5.8 Hz, 1H), 7.93 (td, J = 7.9, 1.4 Hz, 1H), 7.66 (d, J = 8.1 Hz, 1H), 7.45 – 7.42 (m, 1H), 7.42 – 7.40 (m, 1H), 7.40 – 7.36 (m, 1H), 7.16 (t, J = 7.7 Hz, 1H), 6.59 – 6.54 (m, 2H), 4.30 – 4.23 (m, 2H), 4.11 (s, 1H), 2.03 (s, 3H), 1.35 (t, J = 7.1 Hz, 3H).

^{13}C NMR (126 MHz, $CDCl_3$) δ 178.9, 157.1, 145.3, 140.3, 138.2, 136.8, 136.5, 133.1, 130.8, 127.6, 126.3, 121.5, 119.6, 117.8, 59.8, 20.7, 14.5.

^{11}B NMR (128 MHz, $CDCl_3$) δ-2.05 (d, J = 85.76 Hz).

HRMS (ESI) m/z: $[M+H]^+$ Calcd. for $C_{22}H_{21}BClNO_2$ 378.1427; Found: 378.1430.

HPLC analysis: DAICEL CHIRALCEL AD-H, hexane/isopropanol = 90/10, 1mL/min, λ = 254 nm, t_R (minor) = 8.957 min, t_R (major) = 13.574 min, 99% ee.

$[\alpha]^{25}_D$: -14.49 (c 0.49, $CHCl_3$).

ethyl (*S*)-2-(7-chloro-6H-5λ^4-benzo[3,4][1,2]azaborolo[1,5-a]pyridin-6-yl)-2-(m-tolyl)acetate (5w)

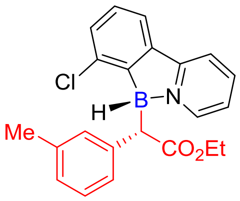

5w

Following the above procedure on 0.1 mmol scale, isolated yield: 84%, (32.7 mg), >20:1 dr, white solid (mp: 92.7 – 93.9 °C),, R_f = 0.3 (silica gel, hexane/EtOAc = 10:1, v/v), column chromatography (silica gel, hexane/EtOAc = 10:1, v/v).

^{1}H NMR (500 MHz, $CDCl_3$) δ 8.95 (d, *J* = 5.8 Hz, 1H), 7.95 – 7.90 (m, 1H), 7.65 – 7.61 (m, 1H), 7.42 – 7.37 (m, 3H), 7.15 (t, *J* = 7.7 Hz, 1H), 6.61 (t, *J* = 7.5 Hz, 1H), 6.54 (d, *J* = 7.5 Hz, 1H), 6.49 (s, 1H), 6.45 (d, *J* = 7.8 Hz, 1H), 4.31 – 4.24 (m, 2H), 4.09 (s, 1H), 1.92 (s, 3H), 1.36 (t, *J* = 7.1 Hz, 3H).

^{13}C NMR (126 MHz, $CDCl_3$) δ 178.7, 157.1, 145.3, 140.3, 139.4, 138.2, 136.8, 136.1, 130.7, 127.7, 127.2, 126.7, 124.57, 123.5, 121.4, 119.4, 117.6, 59.8, 21.1, 14.5.

^{11}B NMR (128 MHz, $CDCl_3$) δ -1.99 (d, *J* = 83.6 Hz).

HRMS (ESI) m/z: $[M+H]^+$ Calcd. for $C_{22}H_{21}BClNO_2$ 378.1427; Found: 378.1422.

HPLC analysis: DAICEL CHIRALCEL AD-H, hexane/isopropanol = 90/10, 1mL/min, λ = 254 nm, t_R (minor) = 13.385 min, t_R (major) = 14.126 min, 97% ee.

$[\alpha]^{25}_D$: -15.05 (*c* 0.52, $CHCl_3$).

ethyl(*S*)-2-(7-chloro-6H-5λ^4-benzo[3,4][1,2]azaborolo[1,5-a]pyridin-6-yl)-2-(3-methoxyphenyl)acetate (5x)

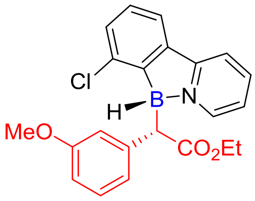

5x

Following the above procedure on 0.1 mmol scale, isolated yield: 75%, (29.5mg), >20:1 dr, white solid (mp: 53.5 – 54.9 °C), R_f = 0.3 (silica gel, hexane/EtOAc = 10:1, v/v), column chromatography (silica gel, hexane/EtOAc = 10:1, v/v).

^{1}H NMR (500 MHz, CDCl$_3$) δ 8.97 (d, *J* = 5.8 Hz, 1H), 7.94 – 7.91 (m, 1H), 7.67 – 7.63 (m, 1H), 7.44 – 7.38 (m, 3H), 7.16 (t, *J* = 7.7 Hz, 1H), 6.63 (t, *J* = 7.9 Hz, 1H), 6.33 – 6.25 (m, 3H), 4.32 – 4.24 (m, 2H), 4.15 (s, 1H), 3.40 (s, 3H), 1.37 (t, *J* = 7.1 Hz, 3H).

^{13}C NMR (126 MHz, CDCl$_3$) δ 178.5, 158.3, 157.1, 145.3, 141.2, 140.4, 138.2, 136.8, 130.8, 127.8, 127.7, 121.5, 119.6, 118.8, 117.8, 111.0, 110.6, 59.8, 54.7, 14.5.

^{11}B NMR (128 MHz, CDCl$_3$) δ -1.55 (s).

HRMS (ESI) m/z: $[M+H]^+$ Calcd. for $C_{22}H_{21}BClNO_3$ 394.1376; Found: 394.1379.

HPLC analysis: DAICEL CHIRALCEL AD-H, hexane/isopropanol = 90/10, 1mL/min, λ = 254 nm, t_R (minor) = 9.680 min, t_R (major) = 12.249 min, 96% ee.

$[\alpha]^{25}_{D}$: -17.30 (*c* 0.53, CHCl$_3$).

ethyl(*S*)-2-(benzo[d][1,3]dioxol-5-yl)-2-(7-chloro-6H-5λ^4-benzo[3,4][1,2]azaborolo[1,5-a]pyridin-6-yl)acetate (5y)

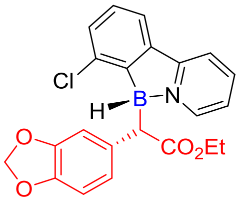

5y

Following the above procedure on 0.1 mmol scale, isolated yield: 63%, (25.6 mg), ＞20:1 dr, white solid (mp: 139.5 – 140.5 °C), R_f = 0.3 (silica gel, hexane/EtOAc = 10:1, v/v), column chromatography (silica gel, hexane/EtOAc = 10:1, v/v).

^{1}H NMR (400 MHz, CDCl$_3$) δ 8.88 (d, *J* = 5.7 Hz, 1H), 7.96 (t, *J* = 7.8 Hz, 1H), 7.70 (d, *J* = 8.1 Hz, 1H), 7.46 (d, *J* = 7.6 Hz, 1H), 7.44 – 7.35 (m, 2H), 7.16 (t, *J* = 7.7 Hz, 1H), 6.30 (s, 1H), 6.18 (dd, *J* = 39.8, 8.1 Hz, 2H), 5.64 (d, *J* = 6.1 Hz, 2H), 4.33 – 4.21 (m, 2H), 4.07 (s, 1H), 1.36 (t, *J* = 7.1 Hz, 3H).

^{13}C NMR (126 MHz, CDCl$_3$) δ 178.7, 157.1, 146.3, 145.1, 143.9, 140.5, 138.1, 136.8, 133.7, 130.9, 127.8, 121.6, 119.6, 119.4, 117.9, 107.3, 106.9, 100.1, 59.9, 14.5

^{11}B NMR (128 MHz, CDCl$_3$) δ -1.97 (d, *J* = 46.1 Hz).

HRMS (ESI) m/z: $[M+H]^+$ Calcd. for $C_{22}H_{19}BClNO_4$ 408.1169; Found: 408.1173.

HPLC analysis: DAICEL CHIRALCEL AD-H, hexane/isopropanol = 90/10, 1mL/min, λ = 254 nm, t_R (minor) = 15.402 min, t_R (major) = 19.516 min, 98% ee.

[α]^{25}$_{D}$: -14.708 (c 0.49, $CHCl_3$).

ethyl(*S*)-2-([1,1'-biphenyl]-4-yl)-2-(7-chloro-6H-5λ^4-benzo[3,4][1,2]azaborolo[1,5-a]pyridin-6-yl)acetate (5z)

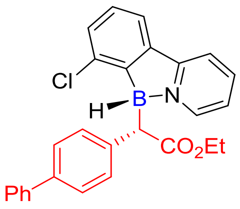

5z

Following the above procedure on 0.1 mmol scale, isolated yield: 74%, (32.5mg), ＞20:1 dr, white solid (mp: 188.7 – 189.6 °C), R_f = 0.3 (silica gel, hexane/EtOAc = 10:1, v/v), column chromatography (silica gel, hexane/EtOAc = 10:1, v/v).

^{1}H NMR (400 MHz, $CDCl_3$) δ 8.96 (d, J = 5.7 Hz, 1H), 7.89 (t, J = 7.7 Hz, 1H), 7.60 (d, J = 8.1 Hz, 1H), 7.43 – 7.32 (m, 5H), 7.28 (t, J = 7.4 Hz, 2H), 7.21 (q, J = 7.4 Hz, 1H), 7.13 (t, J = 7.7 Hz, 1H), 6.99 (d, J = 7.9 Hz, 2H), 6.75 (d, J = 7.8 Hz, 2H), 4.31 – 4.27 (m, 2H), 4.21 (s, 1H), 1.37 (t, J = 7.1 Hz, 3H).

^{13}C NMR (101 MHz, $CDCl_3$) δ 178.6, 157.1, 145.2, 140.9, 140.4, 139.0, 138.1, 136.8, 136.4, 130.8, 128.5, 127.8, 126.7, 126.5, 126.5, 125.4, 121.6, 119.6, 117.8, 59.9, 14.5.

^{11}B NMR (128 MHz, $CDCl_3$) δ -1.90 (s).

HRMS (ESI) m/z: $[M+H]^+$ Calcd. for $C_{27}H_{23}BClNO_2$ 440.1583; Found: 440.1585.

HPLC analysis: DAICEL CHIRALCEL AD-H, hexane/isopropanol = 90/10, 1mL/min, λ = 254 nm, t_R (minor) = 10.700 min, t_R (major) = 12.366 min, 98% ee.

[α]^{25}$_{D}$: 0.57 (c 0.53, $CHCl_3$).

methyl(*S*)-3-(1-(7-chloro-(*R*)6H-5λ^4-benzo[3,4][1,2]azaborolo[1,5-a]pyridin-6-yl)-2-ethoxy-2-oxoethyl)benz-oate (5aa)

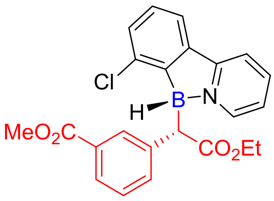

5aa

Following the above procedure on 0.1 mmol scale, isolated yield: 94%, (39.6 mg), ＞20:1 dr, white solid (mp: 126.3 – 127.4 ^{o}C), R_f = 0.3 (silica gel, hexane/EtOAc = 10:1, v/v), column chromatography (silica gel, hexane/EtOAc = 10:1, v/v).

^{1}H NMR (500 MHz, $CDCl_3$) δ 8.91 (d, *J* = 5.7 Hz, 1H), 7.94 (t, *J* = 7.8 Hz, 1H), 7.62 (dd, *J* = 8.0, 1.7 Hz, 1H), 7.45 (dd, *J* = 7.2, 6.0 Hz,1H), 7.41 (d, *J* = 7.6 Hz, 1H), 7.39 – 7.36 (m, 2H), 7.34 (t, *J* = 1.7 Hz, 1H), 7.15 – 7.11 (m, 1H), 6.85 – 6.83 (m, 1H), 6.78 (t, *J* = 7.7 Hz, 1H), 4.35 – 4.24 (m, 2H), 4.21 (s, 1H), 3.73 (d, *J* = 0.9 Hz, 3H), 1.37 (td, *J* = 7.1, 0.6 Hz, 3H).

^{13}C NMR (101 MHz, $CDCl_3$) δ 178.2, 167.2, 157.0, 145.2, 140.6, 140.0, 138.0, 136.8, 131.3, 130.9, 128.6, 127.8, 127.3, 126.8, 125.3, 121.7, 119.5, 117.8, 59.9, 51.6, 14.5.

^{11}B NMR (128 MHz, $CDCl_3$) δ -2.00 (d, *J* = 62.0 Hz).

HRMS (ESI) m/z: $[M+H]^+$ Calcd. for $C_{23}H_{21}BClNO_4$ 422.1325; Found: 422.1325.

HPLC analysis: DAICEL CHIRALCEL AD-H, hexane/isopropanol = 90/10, 1mL/min, λ = 254 nm, t_R (minor) = 15.827 min, t_R (major) = 24.783 min, 99% ee.

$[\alpha]^{25}_{D}$: - 11.91 (*c* 0.5, $CHCl_3$).

ethyl(S)-2-(7-chloro-6H-5λ^{4}-benzo[3,4][1,2]azaborolo[1,5-a]pyridin-6-yl)-2-(4-fluorophenyl)acetate (5ab)

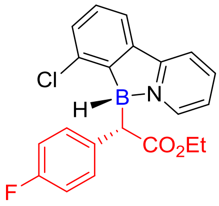

5ab

Following the above procedure on 0.1 mmol scale, isolated yield: 65%, (24.8 mg), ＞20:1 dr, white solid (mp: 189.2 – 190.3 ^{o}C), R_f = 0.3 (silica gel, hexane/EtOAc = 10:1, v/v), column chromatography (silica gel, hexane/EtOAc = 10:1, v/v).

^{1}H NMR (500 MHz, $CDCl_3$) δ 8.89 (d, *J* = 5.3 Hz, 1H), 7.96 (t, *J* = 7.6 Hz, 1H), 7.67 (d, *J* = 7.9 Hz, 1H), 7.43 (d, *J* = 7.1 Hz, 2H), 7.38 (d, *J* = 7.7 Hz, 1H), 7.16 (t, *J* = 7.6 Hz, 1H), 6.68 – 6.59 (m, 2H), 6.42 (t, *J* = 8.6 Hz, 2H), 4.33 – 4.24 (m, 2H), 4.15 (s, 1H), 1.37 (t, *J* = 7.0 Hz, 3H).

^{13}C NMR (101 MHz, $CDCl_3$) δ 178.6, 160.0(d, *J* = 239.9 Hz), 157.1, 145.1, 140.6, 138.1, 136.7, 135.23 (d, *J* = 3.0 Hz), 130.9 9, 127.9, 127.47 (d, *J* = 7.4 Hz), 121.6, 119.6, 117.9, 113.5(d, *J* = 20.6Hz),

59.9, 14.5.

^{11}B NMR (128 MHz, CDCl$_3$) δ -2.01 (d, *J* = 69.4 Hz).

^{19}F NMR (376 MHz, CDCl$_3$) δ -120.16 (s).

HRMS (ESI) m/z: $[M+H]^+$ Calcd. for $C_{21}H_{18}BClFNO_2$ 382.1176; Found:382.1173.

HPLC analysis: DAICEL CHIRALCEL AD-H, hexane/isopropanol = 90/10, 1mL/min, λ = 254 nm, t_R (minor) = 7.553 min, t_R (major) = 10.143 min, 94% ee.

$[\alpha]^{25}_D$: -39.42 (*c* 0.42, $CHCl_3$).

ethyl(*S*)-2-(7-chloro-6H-5λ^4-benzo[3,4][1,2]azaborolo[1,5-a]pyridin-6-yl)-2-(4-chlorophenyl)acetate (5ac)

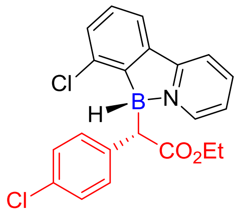

5ac

Following the above procedure on 0.1 mmol scale, isolated yield: 58%, (23.0 mg), ＞20:1 dr, white solid (mp: 144.9 – 146.3 °C), R_f = 0.3 (silica gel, hexane/EtOAc = 10:1, v/v), column chromatography (silica gel, hexane/EtOAc = 10:1, v/v).

^{1}H NMR (500 MHz, CDCl$_3$) δ 8.89 (d, *J* = 5.8 Hz, 1H), 7.96 – 7.92 (m, 1H), 7.66 (d, *J* = 8.1 Hz, 1H), 7.44 – 7.39 (m, 2H), 7.37 (dd, *J* = 7.8, 0.6 Hz, 1H), 7.16 (t, *J* = 7.7 Hz, 1H), 6.70 – 6.66 (m, 2H), 6.64 – 6.60 (m, 1H), 4.32 – 4.21 (m, 2H), 4.15 (s, 1H), 1.36 (t, *J* = 7.1 Hz, 3H).

^{13}C NMR (126 MHz, CDCl$_3$) δ 178.4, 157.1, 145.1, 140.6, 138.2, 138.0, 136.7, 130.9, 129.5, 127.9, 127.6, 126.9, 121.6, 119.7, 117.9, 59.9, 14.5.

^{11}B NMR (128 MHz, CDCl$_3$) δ -2.14 (d, *J* = 59.0 Hz).

HRMS (ESI) m/z: $[M+Na]^+$ Calcd. for $C_{21}H_{18}BCl2NO_2$ 420.0700; Found: 420.0707.

HPLC analysis: DAICEL CHIRALCEL AD-H, hexane/isopropanol = 90/10, 1mL/min, λ = 254 nm, t_R (minor) = 8.405 min, t_R (major) = 13.750 min, 93% ee.

$[\alpha]^{25}_D$: - 24.50(*c* 0.66, $CHCl_3$).

ethyl(*S*)-2-(4-bromophenyl)-2-(7-chloro-6H-5λ^4-benzo[3,4][1,2]azaborolo[1,5-a]pyridin-6-yl)acetate (5ad)

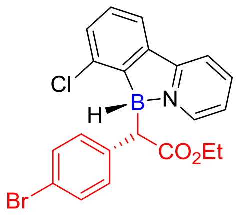

5ad

Following the above procedure on 0.1 mmol scale, isolated yield: 82%, (36.2 mg), >20:1 dr, white solid (mp: 158.2 – 159.5 ^oC), R_f = 0.3 (silica gel, hexane/EtOAc = 10:1, v/v), column chromatography (silica gel, hexane/EtOAc = 10:1, v/v).

^{1}H NMR (400 MHz, $CDCl_3$) δ 8.91 (d, *J* = 5.6 Hz, 1H), 7.96 (t, *J* = 7.7 Hz, 1H), 7.68 (d, *J* = 8.1 Hz, 1H), 7.53 – 7.35 (m, 3H), 7.17 (t, *J* = 7.7 Hz, 1H), 6.84 (d, *J* = 8.2 Hz, 2H), 6.57 (d, *J* = 8.2 Hz, 2H), 4.28 (dd, *J* = 6.9, 4.8 Hz, 2H), 4.14 (s, 1H), 1.36 (t, *J* = 7.1 Hz, 3H).

^{13}C NMR (101 MHz, $CDCl_3$) δ 178.3, 157.1, 145.1, 140.6, 138.7, 138.1, 136.7, 130.9, 129.8, 128.0, 127.9, 121.7, 119.7, 118.0, 117.6, 59.9, 14.5.

^{11}B NMR (128 MHz, $CDCl_3$) δ -2.11 (d, *J* = 55.4 Hz).

HRMS (ESI) m/z: $[M+H]^+$ Calcd. for $C_{21}H_{18}BClBrNO_2$ 422.0375; Found: 422.0371.

HPLC analysis: DAICEL CHIRALCEL AD-H, hexane/isopropanol = 90/10, 1mL/min, λ = 254 nm, t_R (minor) = 8.955 min, t_R (major) = 14.917 min, 89% ee.

$[\alpha]^{25}_D$: -17.94 (*c* 0.51, $CHCl_3$).

ethyl(*S*)-2-(7-chloro-6H-5λ^4-benzo[3,4][1,2]azaborolo[1,5-a]pyridin-6-yl)-2-(naphthalen-2-yl)acetate(5ae)

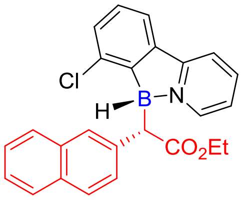

5ae

Following the above procedure on 0.1 mmol scale, isolated yield: 95%, (39.2 mg), >20:1 dr, white solid (mp: 119.1-120.8 ^oC), R_f = 0.3 (silica gel, hexane/EtOAc = 10:1, v/v), column chromatography (silica gel, hexane/EtOAc = 10:1, v/v).

^{1}**H NMR (400 MHz, CDCl$_3$)** δ 9.02 (d, *J* = 5.7 Hz, 1H), 7.80 (t, *J* = 6.2 Hz, 1H), 7.48 (d, *J* = 7.6 Hz, 1H), 7.45 – 7.33 (m, 4H), 7.27 – 7.15 (m, 5H), 7.08 (t, *J* = 7.7 Hz, 1H), 6.76 (d, *J* = 8.5 Hz, 1H), 4.36 – 4.25 (m, 3H), 1.37 (t, *J* = 7.1 Hz, 3H).

^{13}**C NMR (101 MHz, CDCl$_3$)** δ 178.7, 156.9, 145.2, 140.3, 138.1, 137.4, 136.8, 132.9, 130.8, 130.7, 127.8, 127.3, 127.0, 126.5, 126.0, 124.9, 124.0, 123.6, 121.5, 119.6, 117.7, 59.9, 14.5.

^{11}**B NMR (128 MHz, CDCl$_3$)** δ -2.01 (s).

HRMS (ESI) m/z: $[M+H]^+$ Calcd. for $C_{25}H_{21}BClNO_2$ 414.1427; Found: 414.1422.

HPLC analysis: DAICEL CHIRALCEL AD-H, hexane/isopropanol = 90/10, 1mL/min, λ = 254 nm, t_R (minor) = 14.795 min, t_R (major) = 17.818 min, 99% ee.

$[\alpha]^{25}_D$: 49.38 (*c* 0.54, $CHCl_3$).

2.6. Gram-scale reaction and transformations.

A Gram-scale reaction

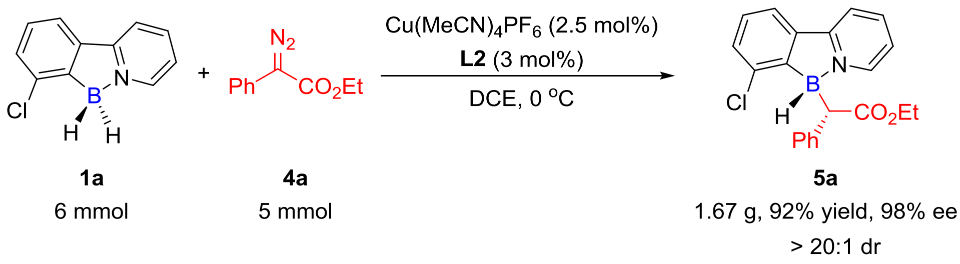

Supplementary Figure 5. Gram-scale reaction

In air, a 10 mL schlenk tube was charged with **1a** (6 mmol, 1.2 equiv), **2a** (5 mmol, 1 equiv), $Cu(MeCN)_4PF_6$ (2.5 mol%), **L2** (3 mol%). The tube was evacuated and filled with argon for three cycles. Then, 1 mL of 1,2-dichlorethane was added under argon. The reaction was allowed to stir at 0 °C for 16 hours. Upon completion, proper amount of silica gel was added to the reaction mixture. After removal of the solvent, the crude reaction mixture was purified on silica gel (petroleum ether and ethyl acetate) to afford the desired products, isolated yield: 92%, 98% ee.

B Transformations

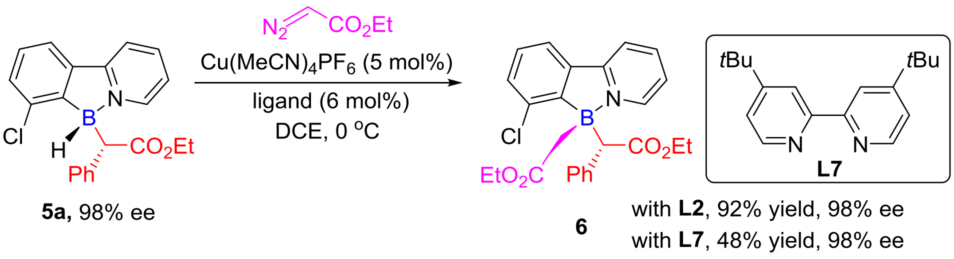

Supplementary Figure 6. Second B-H bond insertion reaction

In air, a 10 mL schlenk tube was charged with **5a** (0.1 mmol, 1 equiv), ethyl diazoacetate (0.2 mmol, 2 equiv), $Cu(MeCN)_4PF_6$ (5 mol%), **L2** (6 mol%). The tube was evacuated and filled with argon for three cycles. Then, 1 mL of 1, 2-dichlorethane was added under argon. The reaction was allowed to stir at 0 °C for 20 hours. Upon completion, proper amount of silica gel was added to the reaction mixture. After removal of the solvent, the crude reaction mixture was purified on silica gel (petroleum ether and ethyl acetate) to afford the desired products, isolated yield: 92% (41.3 mg), if **L7** was used, isolated yield: 48%, white solid (mp: 110.9 – 112.3 °C), (21.6 mg), R_f = 0.3 (silica gel, hexane/EtOAc = 5:1, v/v), column chromatography (silica gel, hexane/EtOAc = 5:1, v/v).

^{1}H NMR (500 MHz, $CDCl_3$) δ 9.02 (d, *J* = 5.8 Hz, 1H), 7.99 – 7.95 (m, 1H), 7.59 (d, *J* = 8.1 Hz, 1H), 7.52 – 7.50 (m, 1H), 7.38 – 7.35 (m, 1H), 7.32 (d, *J* = 7.5 Hz, 1H), 7.12 (t, *J* = 7.7 Hz, 1H), 6.72 – 6.63 (m, 5H), 4.33 – 4.26 (m, 1H), 4.21 – 4.15 (m, 1H), 4.14 (s, 1H), 3.45 (q, *J* = 7.1 Hz, 2H), 2.75 (d, *J* = 11.9 Hz, 1H), 2.00 (d, *J* = 11.9 Hz, 1H), 1.35 (t, *J* = 7.1 Hz, 3H), 0.54 (t, *J* = 7.1 Hz, 3H).

^{13}C NMR (126 MHz, $CDCl_3$) δ 177.5, 176.1, 156.7, 144.9, 141.1, 139.4, 137.9, 137.3, 131.1, 128.1, 126.8, 126.3, 124.0, 121.8, 119.3, 117.5, 59.8, 58.5, 14.6, 13.6.

^{11}B NMR (128 MHz, $CDCl_3$) δ 1.25 (s).

HRMS (ESI) m/z: $[M+Na]^+$ Calcd. for $C_{25}H_{25}BClNO_4$ 472.1457; Found: 472.1466.

HPLC analysis: DAICEL CHIRALCEL AD-H, hexane/isopropanol = 90/10, 1mL/min, λ = 254 nm, t_R (minor) = 9.324 min, t_R (major) = 11.741 min, 98% ee.

$[\alpha]^{25}_D$: -4.69 (*c* 0.48, $CHCl_3$).

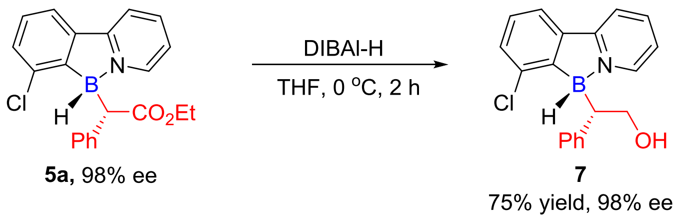

Supplementary Figure 7. Reduction reaction

In air, a 10 mL schlenk tube was charged with **5a** (0.1 mmol, 1 equiv). The tube was evacuated and filled with argon for three cycles. Then, 1 mL of THF (1mL) and DIBAl-H (1.0mol/l in hexane) (0.15 mmol, 1.5 equiv) were added under argon. The reaction was allowed to stir at 0 °C for 3 hours. Upon completion, proper amount of silica gel was added to the reaction mixture. After removal of the solvent, the crude reaction mixture was purified on silica gel (petroleum ether and ethyl acetate) to afford the desired products, isolated yield: 75%, 98% ee, white oild, (24.1 mg), R_f = 0.5 (silica gel, hexane/DCM = 3:1, v/v), column chromatography (silica gel, hexane/EtOAc = 10:1, v/v).

^{1}H NMR (500 MHz, $CDCl_3$) δ 8.30 (d, *J* = 5.8 Hz, 1H), 7.93 – 7.88 (m, 1H), 7.71 (d, *J* = 8.1 Hz, 1H), 7.50 (dd, *J* = 7.6, 0.5 Hz, 1H), 7.39 (dd, *J* = 7.8, 0.5 Hz, 1H), 7.30 – 7.27 (m, 1H), 7.18 (t, *J* = 7.7 Hz, 1H), 6.88 – 6.83 (m, 2H), 6.83 – 6.79 (m, 1H), 6.56 (dd, *J* = 5.3, 3.2 Hz, 2H), 4.39 (t, *J* = 10.5 Hz, 1H), 4.31 (dd, *J* = 10.8, 5.7 Hz, 1H), 3.02 – 2.93 (m, 1H).

^{13}C NMR (126 MHz, $CDCl_3$) δ 156.9, 144.3, 143.8, 139.9, 138.0, 136.8, 130.8, 127.5, 127.4, 126.8, 123.9, 121.4, 119.7, 118.1, 66.4.

^{11}B NMR (128 MHz, $CDCl_3$) δ -1.96 (d, *J* = 99.6 Hz).

HRMS (ESI) m/z: $[M+H]^+$ Calcd. for $C_{19}H_{17}BClNO$ 322.1165; Found: 322.1166.

HPLC analysis: DAICEL CHIRALCEL AD-H, hexane/isopropanol = 90/10, 1mL/min, λ = 254 nm, t_R (minor) = 9.872 min, t_R (major) = 18.288 min, 98% ee.

$[\alpha]^{25}_{D}$: -9.55 (*c* 0.52, $CHCl_3$).

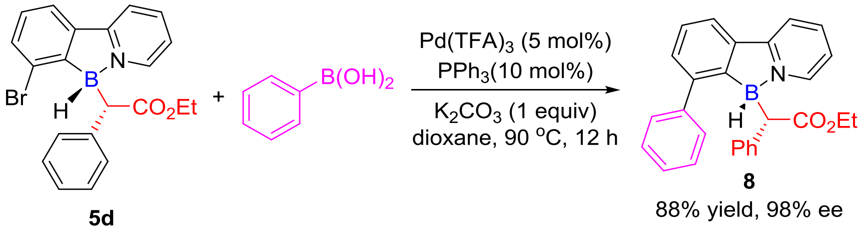

Supplementary Figure 8. Palladium-catalyzed cross-coupling reaction

In air, a 10 mL schlenk tube was charged with **5d** (0.1 mmol, 1 equiv), phenylboronic acid (0.12 mmol, 1.2 equiv), $Pd(TFA)_3$ (5 mol%), PPh_3 (10 mol%) and K_2CO_3(1 equiv). The tube was evacuated and filled with argon for three cycles. Then, 1 mL of 1,4-dioxane was added under argon. The reaction was allowed to stir at 90 °C for 12 hours. Upon completion, proper amount of silica gel was added to the reaction mixture. After removal of the solvent, the crude reaction mixture was purified on silica gel (petroleum ether and ethyl acetate) to afford the desired products, isolated yield: 88% (35.7 mg), white solid (mp: 105.2 – 106.6 °C), R_f = 0.5 (silica gel, hexane/EtOAc = 5:1, v/v), column chromatography (silica gel, hexane/EtOAc = 10:1, v/v).

^{1}H NMR (500 MHz, $CDCl_3$) δ 9.00 (d, *J* = 5.8 Hz, 1H), 7.97 (dd, *J* = 8.1, 1.0 Hz, 2H), 7.88 (t, *J* = 7.8 Hz, 1H), 7.67 (d, *J* = 8.1 Hz, , 1H), 7.57 – 7.52 (m, 4H), 7.43 (t, *J* = 7.4 Hz, 1H), 7.39 – 7.31 (m, 2H), 6.70 – 6.65 (m, 3H), 6.50 – 6.44 (m, 2H), 4.27 – 4.20 (m, 1H), 4.11 – 4.05 (m, 1H), 3.32 (s, 1H), 1.32 (t, *J* = 7.1 Hz, 3H).

^{13}C NMR (126 MHz, $CDCl_3$) δ 179.0, 157.9, 144.9, 143.9, 142.3, 140.0, 139.5, 137.2, 130.6, 128.9, 128.4, 127.1, 126.9, 126.6, 126.2, 123.5, 121.0, 120.0, 117.4, 59.4, 14.4.

^{11}B NMR (128 MHz, $CDCl_3$) δ -1.58 (s).

HRMS (ESI) m/z: $[M+H]^+$ Calcd. for $C_{27}H_{24}BNO$ 406.1973; Found: 406.1977.

HPLC analysis: DAICEL CHIRALCEL AD-H, hexane/isopropanol = 90/10, 1mL/min, λ = 254 nm, t_R (minor) =6.897 min, t_R (major) = 12.769 min, 98% ee.

$[\alpha]^{25}_D$: 139.80 (*c* 0.51, $CHCl_3$).

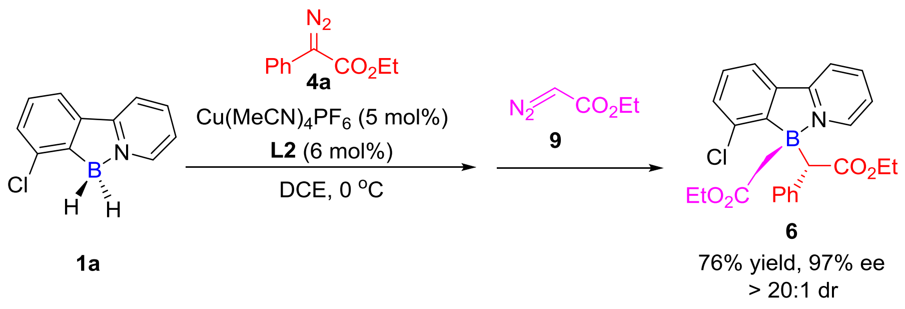

Supplementary Figure 9. The double B-H bond insertion reactions

In air, a 10 mL schlenk tube was charged with **1a** (0.12 mmol, 1.2 equiv), **4a** (0.1 mmol, 1 equiv), $Cu(MeCN)_4PF_6$ (5 mol%), **L2** (6 mol%). The tube was evacuated and filled with argon for three cycles. Then, 1 mL of 1,2 -dichlorethane was added under argon. The reaction was allowed to stir at 0 °C for

12 hours. Further, ethyl diazoacetate (0.2 mmol, 2 equiv) was added, the reaction was allowed to stir at 0 °C for 12 hours. Upon completion, proper amount of silica gel was added to the reaction mixture. After removal of the solvent, the crude reaction mixture was purified on silica gel (petroleum ether and ethyl acetate) to afford the desired products, isolated yield: 76%, 97% ee。

2.7. Isotope labeling experiments.

A Deuterium labeling experiment

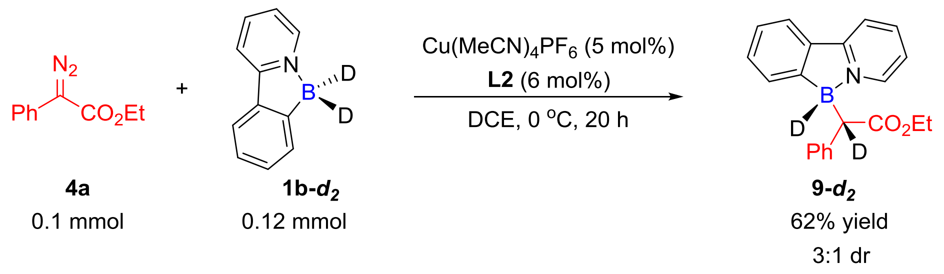

Supplementary Figure 10. Deuterium labeling experiment

In air, a 10 mL schlenk tube was charged with **1b-*d*$_2$** (0.12 mmol, 1.2 equiv), **4a** (0.1 mmol, 1 equiv), $Cu(MeCN)_4PF_6$ (5 mol%), **L2** (6 mol%). The tube was evacuated and filled with argon for three cycles. Then, 1 mL of 1,2 - Dichlorethan was added under argon. The reaction was allowed to stir at 0 °C for 16 hours. Upon completion, proper amount of silica gel was added to the reaction mixture. After removal of the solvent, the crude reaction mixture was purified on silica gel (petroleum ether and ethyl acetate) to afford the desired products.

B KIE experiment

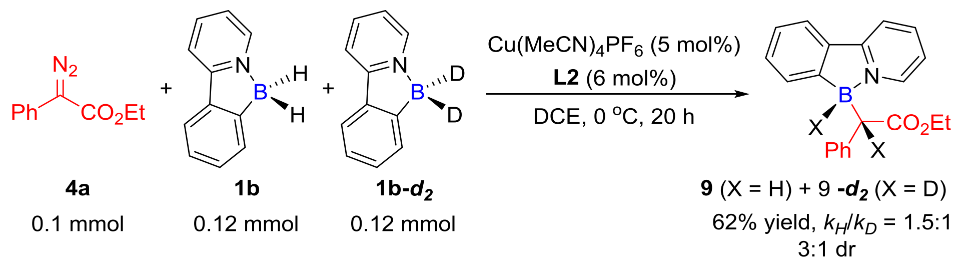

Supplementary Figure 11. KIE experiment

In air, a 10 mL schlenk tube was charged with **1b-*d*$_2$** (20.3 mg, 0.12 mmol, 1.2 equiv), **1b** (20.0 mg, 0.12 mmol, 1.2 equiv), **4a** (19 mg, 0.1 mmol, 1 equiv), $Cu(MeCN)_4PF_6$ (1.8 mg, 5 mol%), **L2** (2.4 mg, 6 mol%). The tube was evacuated and filled with argon for three cycles. Then, 1 mL of 1,2-dichlorethane was added under argon. The reaction was allowed to stir at 0 °C for 16 hours. Upon

completion, proper amount of silica gel was added to the reaction mixture. After removal of the solvent, the crude reaction mixture was purified on silica gel (petroleum ether and ethyl acetate) to afford the desired products.

2.8. Studies of the configurational stability

Supplementary Table 3. Studies of the configurational stability of 5p (91% ee)

Entry	Solvent	T(^{o}C)	Time	ee %
1	toluene	100	24	91
2	toluene	110	24	91
3	toluene	120	24	91

Unless noted otherwise, the configurational stability of the product **5p** was studied by heating a solution of 10 mg **5p** in 2 mL solvent. The enantiomeric excess (ee) was determined by HPLC.

Supplementary Table 4. Studies of the configurational stability of 6 (97% ee)

Entry	Solvent	T(^{o}C)	Time	ee %
1	toluene	100	24	97
2	toluene	110	24	97
3	toluene	120	24	97

Unless noted otherwise, the configurational stability of the product **6** was studied by heating a solution of 10 mg **6** in 2 mL solvent. The enantiomeric excess (ee) was determined by HPLC

2.9. Crystal structure of compound 3n and 5a

For **3n**: the data was collected by using molybdenum (Cu) irradiation source at room temperature.

The crystal sample of **3n** was recrystallized from a mixture of DCM and PE.

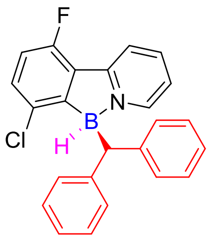

3n

(CCDC 2125214)

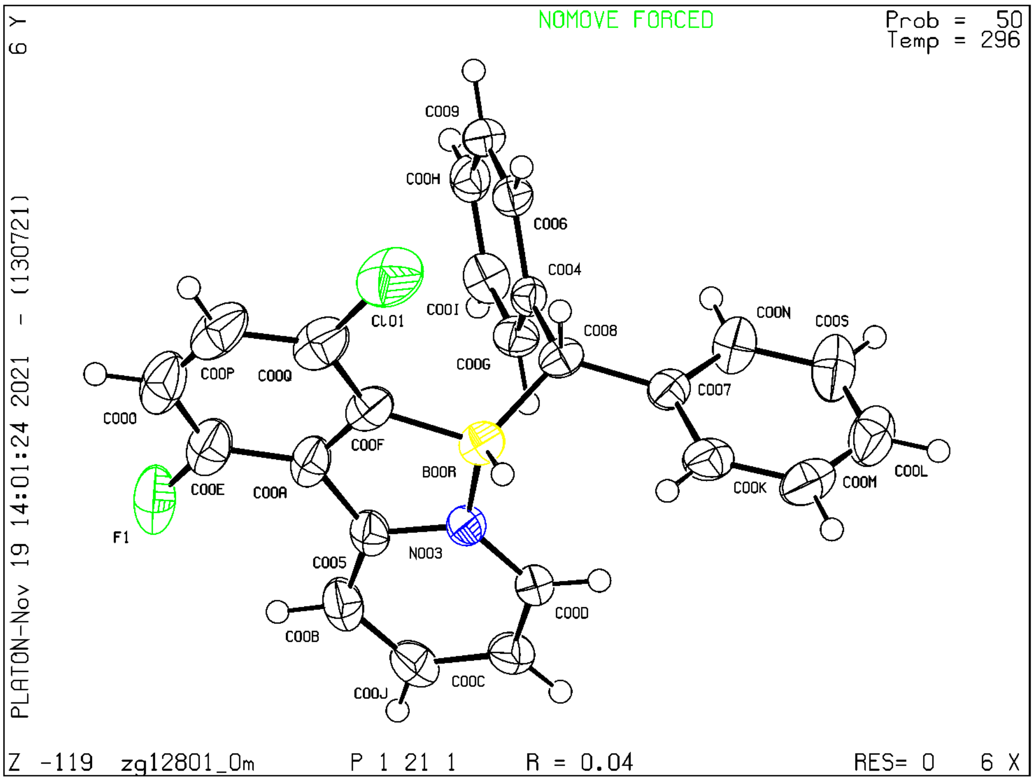

Supplementary Figure 12. Crystal structure of compound 3n

Bond precision:	C-C = 0.0054 A		Wavelength=1.54184
Cell:	a = 7.3954(10)	b = 15.198(3)	c = 9.2120(15)
	alpha=90	beta=111.170(12)	gamma=90
Temperature:	296 K		
	Calculated		Reported

Volume	965.5(3)	965.5(3)
Space group	P 21	P 1 21 1
Hall group	P 2yb	P 2yb
Moiety formula	C24 H18 B Cl F N	C24 H18 B Cl F N
Sum formula	C24 H18 B Cl F N	C24 H18 B Cl F N
Mr	385.65	385.65
Dx, g cm-3	1.327	1.327
Z	2	2
Mu (mm-1)	1.890	1.890
F000	400.0	400.0
F000’	401.77	
h, k, lmax	8, 17, 10	8, 17, 10
Nref	3078 [1604]	3044
Tmin, Tmax	0.782, 0.828	0.650, 0.752
Tmin’	0.782	

Correction method = # Reported T Limits: Tmin = 0.650 Tmax= 0.752

AbsCorr = MULTI-SCAN

Data completeness = 1.90/0.99 Theta(max) = 62.467

R(reflections)= 0.0364(2722) wR2(reflections)= 0.0902(3044)

S = 1.023 Npar= 253

For **5a**: the data was collected by using molybdenum (Mo) irradiation source at room temperature.

The crystal sample of **5a** was recrystallized from a mixture of DCM and PE.

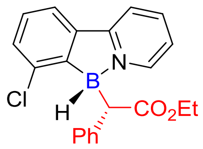

5a

(CCDC 2104867)

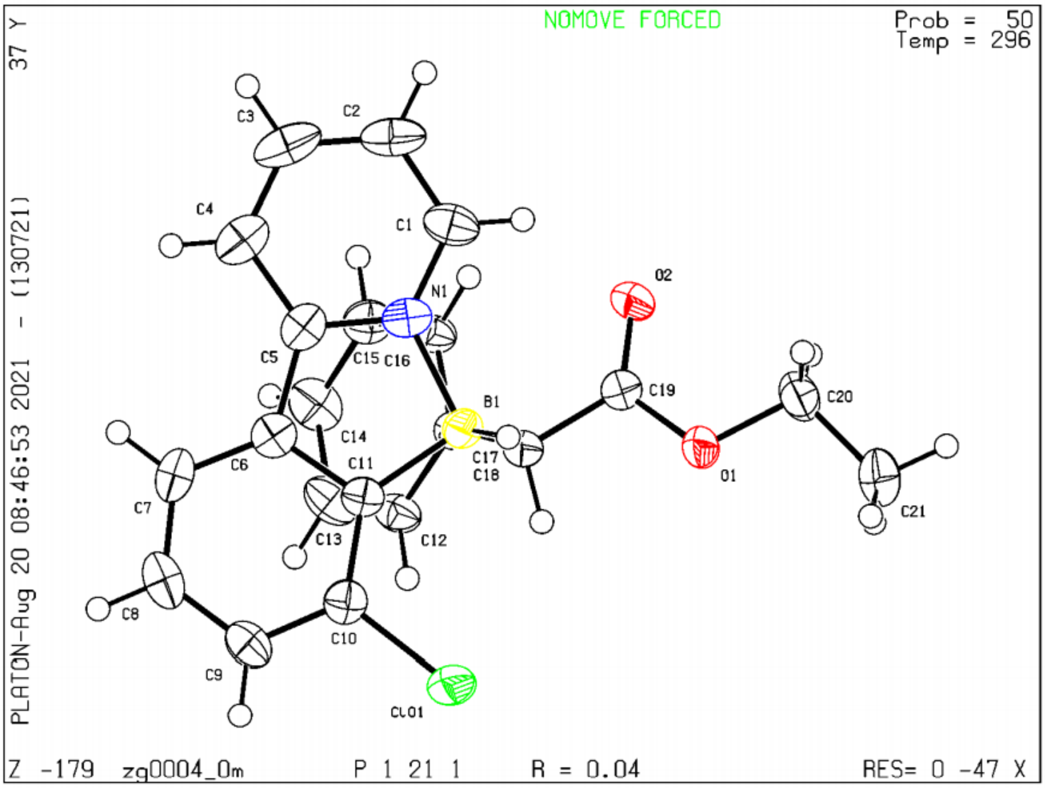

Supplementary Figure 13. Crystal structure of compound 5a

Bond precision:	C-C = 0.0049 A		Wavelength=0.71073
Cell:	a=7.7657(10)	b=15.050(2)	c=8.2425(11)
	alpha=90	beta=107.054(4)	gamma=90
Temperature: 296 K			

	Calculated	Reported
Volume	921.0(2)	921.0(2)
Space group	P 21	P 1 21 1
Hall group	P 2yb	P 2yb
Moiety formula	C21 H19 B Cl N O2	C21 H19 B Cl N O2
Sum formula	C21 H19 B Cl N O2	C21 H19 B Cl N O2
Mr	363.63	363.63
Dx, g cm-3	1.311	1.311

Z	2	2
Mu (mm-1)	0.222	0.222
F000	380.0	380.0
F000’	380.44	
h,k,lmax	9,17,9	9,17,9
Nref	3242 [1689]	3141
Tmin,Tmax	0.967,0.974	
Tmin’	0.967	

Correction method= Not given

Data completeness= 1.86/0.97 Theta(max)= 24.991

R(reflections)= 0.0364(2725) wR2(reflections)= 0.0761(3141)

S = 1.054 Npar= 236

3. Supplementary Discussion

3.1. Computational details

All of the calculations were performed using the Gaussian 16 A.03 program.[13] Structures were optimized at the B3LYP level of density functional theory[14] in solution (DCE), using the SMD model.[15] For optimizations the def2SVP was used for all atoms.[16] Frequency calculations have been performed to verify the optimized structures as local minima or transition states and to obtain Gibbs free energy at 298 K. To reduce error caused by the breakdown of the harmonic oscillator approximation, Truhlar’s quasiharmonic correction was used to compute molecular entropies by setting all positive frequencies that are less than 100 cm^{-1} to 100 cm^{-1}.[17] Intrinsic reaction coordinate (IRC) calculations were carried out to make sure that every transition state links relevant intermediates.[18] The more accurate relative stabilities were further refined by carrying out single-point energy calculations using M06 functional[19] with def2TZVP basis set for for all atoms.[16] The SMD solvation model with DCE as the solvent was employed to account for solvation effect.[15] The distortion-interaction analysis were performed at the M06/SMD/def2TZVP level of theory based on the geometries of optimized transition states. The three-dimensional (3D) structures were depicted using CYLview software.[20]

3.2. Additional computational results

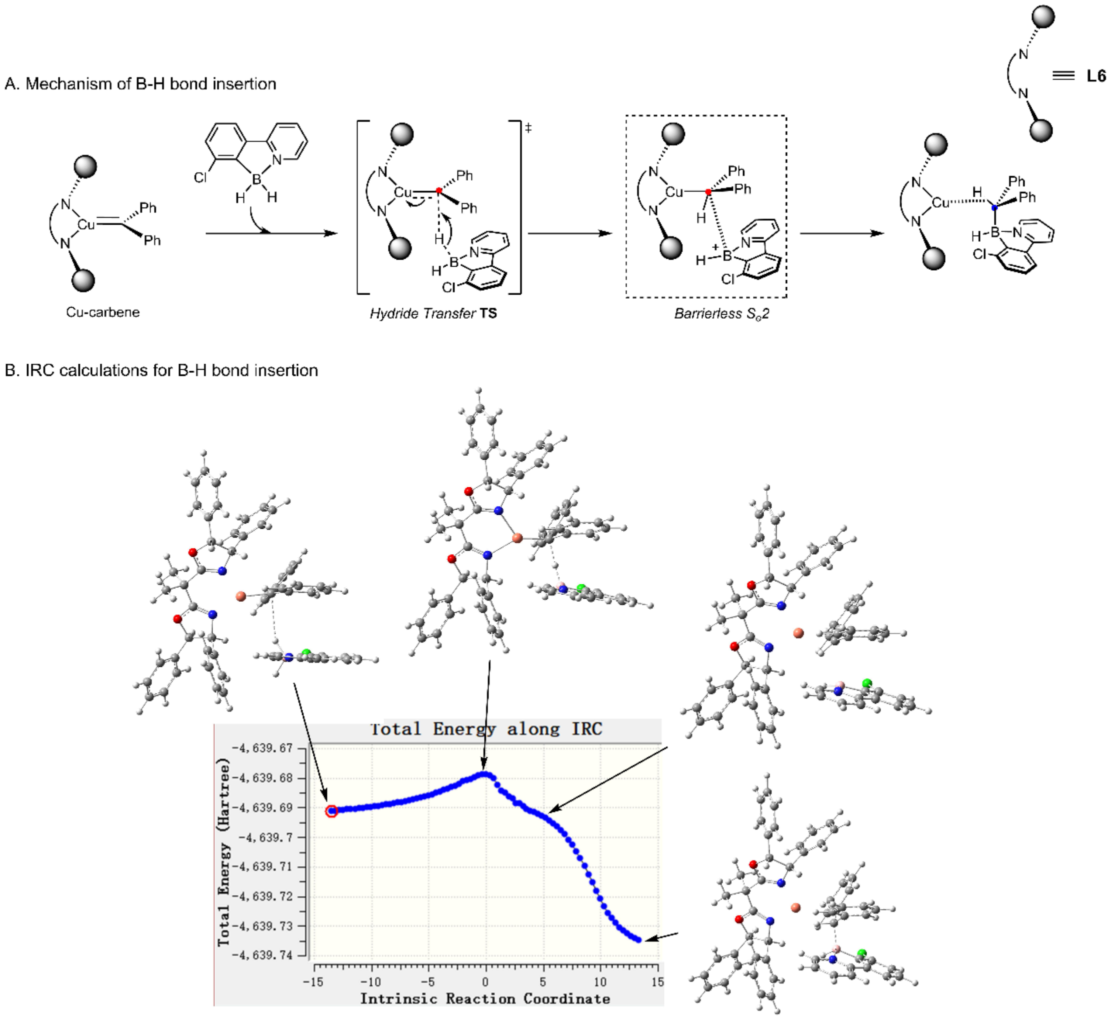

Supplementary Figure 14. Computational investigation on the mechanism of B–H bond insertion into copper carbene.

Comments on **Supplementary Figure 14**: IRC calculations verified that the calculated transition state links reactant and product and after hydride transfer step, the formation of B–C bond immediately delivers product without any energy penalty. Our calculations indicate that the boron cation bonds with the carbon atom immediately supported by the deuterium experiments (see Scheme 4) where no crossover product was observed.

Supplementary Table 5. Distortion-interaction analysis for transition states in Figure 2; energies are given in kcal/mol

	$TS_{S\text{-}3a\text{-}L6}$	$TS_{R\text{-}3a\text{-}L6}$	$TS_{S\text{-}3b\text{-}L6}$	$TS_{R\text{-}3b\text{-}L6}$
ΔE_{dist}(carbene)	7.9	8.3	7.2	7.0
ΔE_{dist}(borane)	2.7	2.2	2.0	2.0
ΔE_{dist}(total)	10.7	10.5	9.2	9.0
ΔE_{int}	-10.1	-8.3	-8.8	-8.0
$\Delta\Delta E_{int}$	0.0	1.8	0.0	0.8
$\Delta\Delta E^{\ddagger}$	0.0	1.6	0.0	0.6

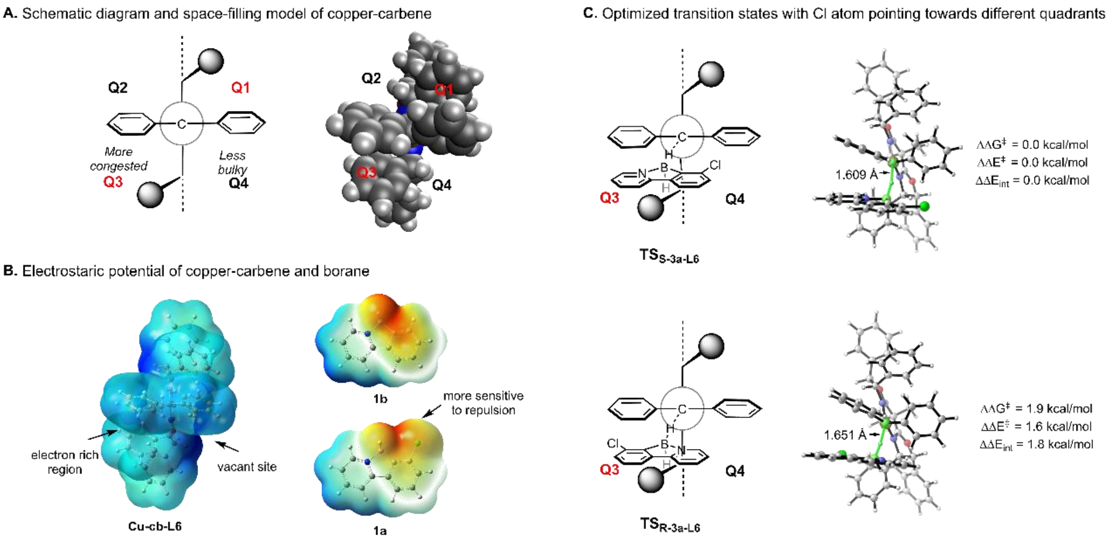

Supplementary Figure 15. Comparison of DFT-calculated structures of $TS_{S\text{-}3a\text{-}L6}$ and $TS_{R\text{-}3a\text{-}L6}$.

Comments on **Supplementary Figure 15**: Electrostatic potential shows that quadrant 3 (**Q3**) in Newman projection formula was rich in electron density while **Q4** is less bulky. Herein, borane prefers to approach copper carbene with highly repulsive Cl atom occupying **Q4** in **$TS_{S\text{-}3a\text{-}L6}$** to avoid steric hindrance in **Q3**.

Supplementary Table 6. Distortion-interaction analysis for transition states in Figure 3; energies are given in kcal/mol

	$TS_{SR\text{-}5h\text{-}L3}$	$TS_{SS\text{-}5h\text{-}L3}$	$TS_{RS\text{-}5h\text{-}L3}$	$TS_{RR\text{-}5h\text{-}L3}$	$TS_{SR\text{-}11\text{-}L3}$	$TS_{SS\text{-}11\text{-}L3}$	$TS_{RS\text{-}11\text{-}L3}$	$TS_{RR\text{-}11\text{-}L3}$
ΔE_{dist}(carbene)	6.4	7.4	8.2	10.9	5.3	5.3	7.0	7.7
ΔE_{dist}(borane)	1.2	1.3	0.9	1.8	0.9	0.7	0.9	0.8
ΔE_{dist}(total)	7.6	8.7	9.1	12.8	6.1	6.0	8.0	8.5
ΔE_{int}	-9.6	-7.7	-7.9	-8.6	-8.3	-7.4	-7.5	-6.9
$\Delta\Delta E_{int}$	0.0	1.9	1.8	1.0	0.0	0.9	0.8	1.4
$\Delta\Delta E_{dist}$(carbene)	0.0	1.0	1.8	4.5	0.0	0.0	1.8	2.4
$\Delta\Delta E^{\ddagger}$	0.0	3.1	3.3	6.2	0.0	0.7	2.6	3.8

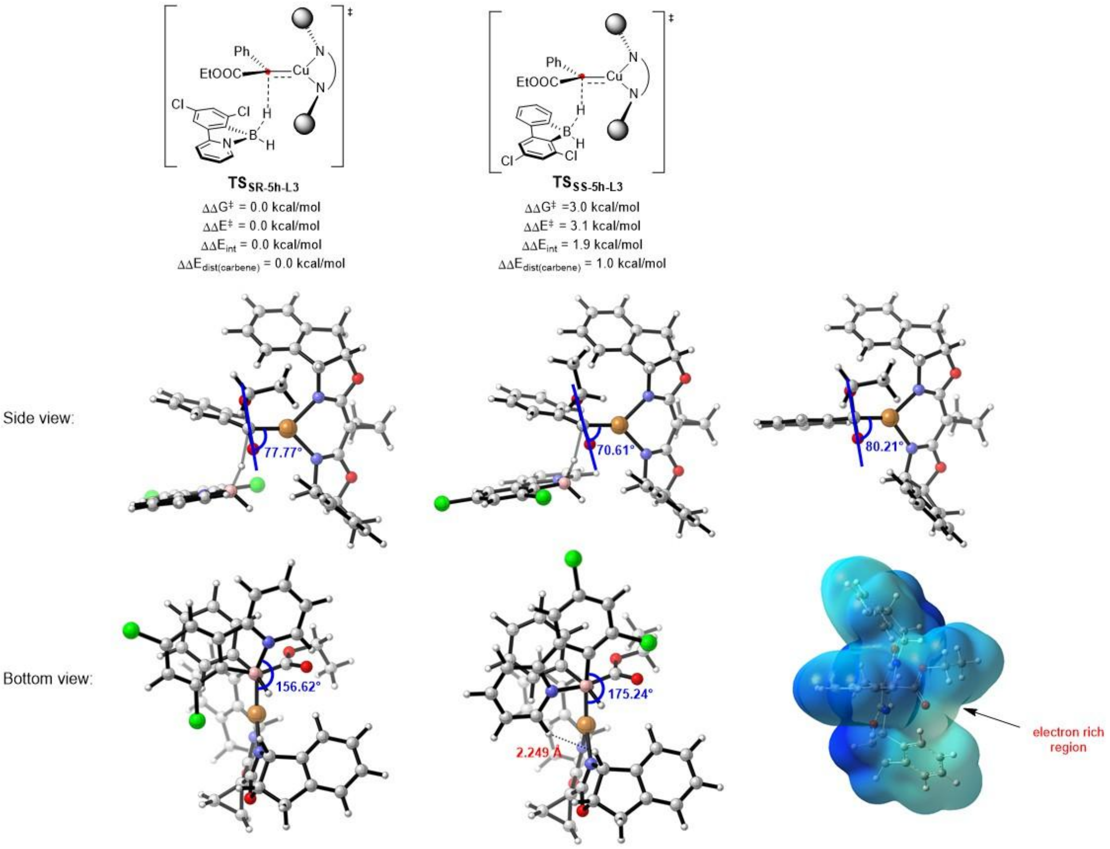

Supplementary Figure 16. Comparison of DFT-calculated structures of $TS_{SR\text{-}5h\text{-}L3}$ and $TS_{SS\text{-}5h\text{-}L3}$.

Comments on **Supplementary Figure 16**: From side view of the optimized structures, the distortion of the dihedral angle ∠Cu-C-C-O in **$TS_{SS\text{-}5h\text{-}L3}$** is larger than that in **$TS_{SR\text{-}5h\text{-}L3}$** by 7.16°. The electrostatic potential shows that ester group in carbene is relatively rich in electron density and is distorted away from ideal when Cl atom of borane enters the same space. In the meanwhile, ester group also pushes

the borane substrate towards ligand, causing repulsion between the latter two fragments (H–H distance is only 2.249 Å) and thus less interaction in the transition state.

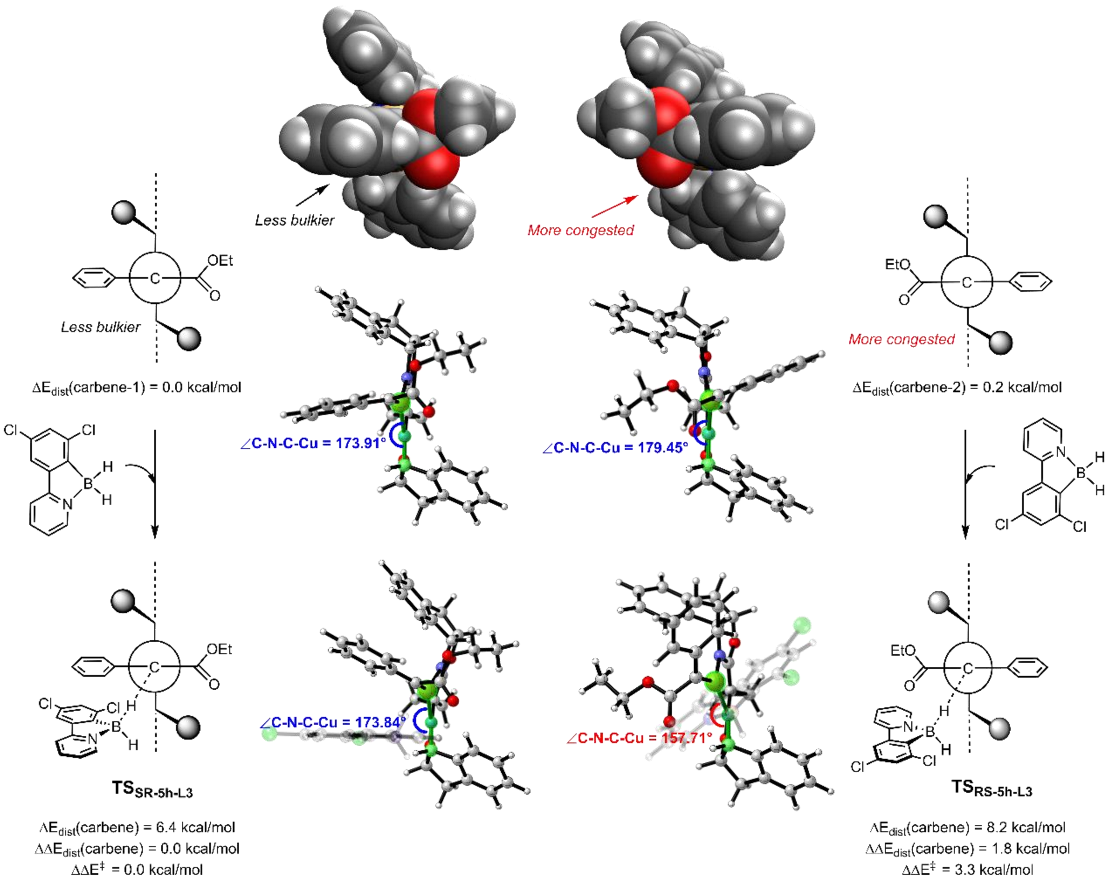

Supplementary Figure 17. Comparison of DFT-calculated structures of TS$_{SR\text{-}5h\text{-}L3}$ and TS$_{RS\text{-}5h\text{-}L3}$.

Comments on **Supplementary Figure 17**: In **TS$_{RS\text{-}5h\text{-}L3}$**, borane substrate could only approach carbene in a more congested space which results in a larger distortion of ligand. Compared with the copper-carbene species and **TS$_{SR\text{-}5h\text{-}L3}$**, a much smaller dihedral angle ∠C-N-C-Cu in **TS$_{RS}$** indicates that the lone pair of N atom in ligand contains a worse overlap with metal d orbital and this slightly weaker coordination accounts for the destabilization of carbene fragment in **TS$_{RS\text{-}5h\text{-}L3}$** as well as the origin of enantio-selectivity.

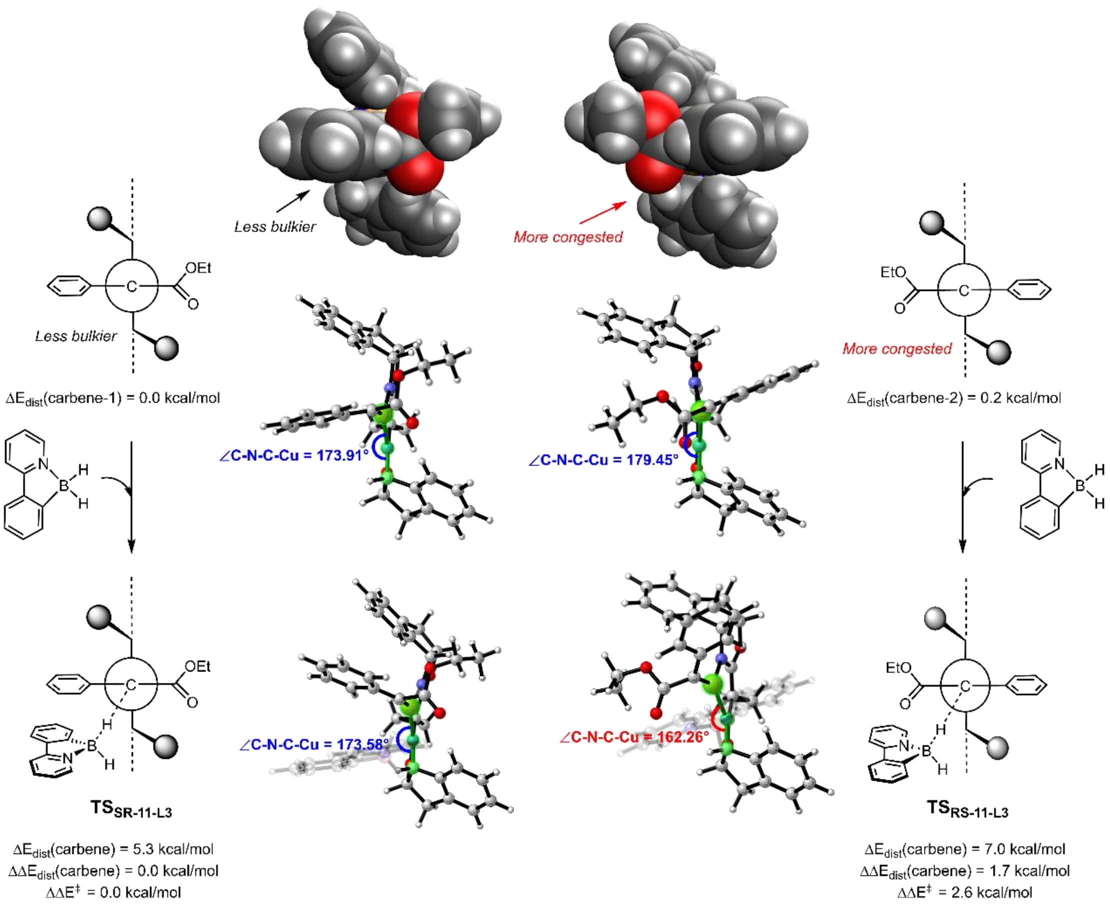

Supplementary Figure 18. Comparison of DFT-calculated structures of $TS_{SR-11-L3}$ and $TS_{RS-11-L3}$.

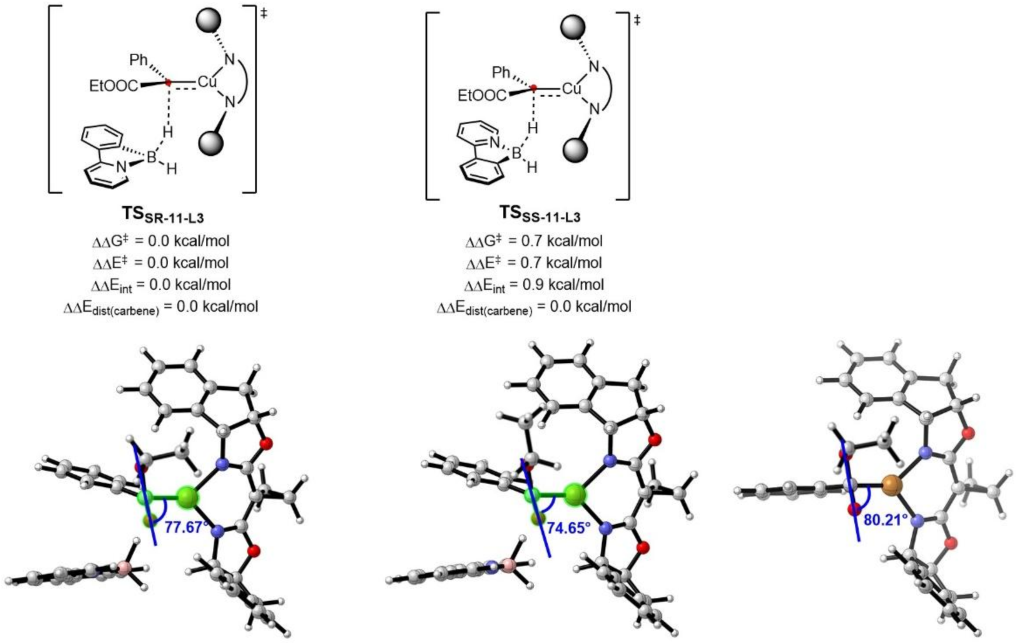

Supplementary Figure 19. Comparison of DFT-calculated structures of $TS_{SR-11-L3}$ and **$TS_{SS-11-L3}$.**

Comments on **Supplementary Figure 19**: From side view of the optimized structures, the distortion of the dihedral angle ∠Cu-C-C-O in $\mathbf{TS_{SS\text{-}5h\text{-}L3}}$ is comparable with that in $\mathbf{TS_{SR\text{-}5h\text{-}L3}}$ and thus the diastereomer ratio is low.

4. Supplementary Figures

4.1. NMR spectra

7-chloro-6H-5λ[4]-benzo[3,4][1,2]azaborolo[1,5-a]pyridine(1a)

^{1}H NMR (500 MHz, room temperature, $(CD_3)_2CO$)

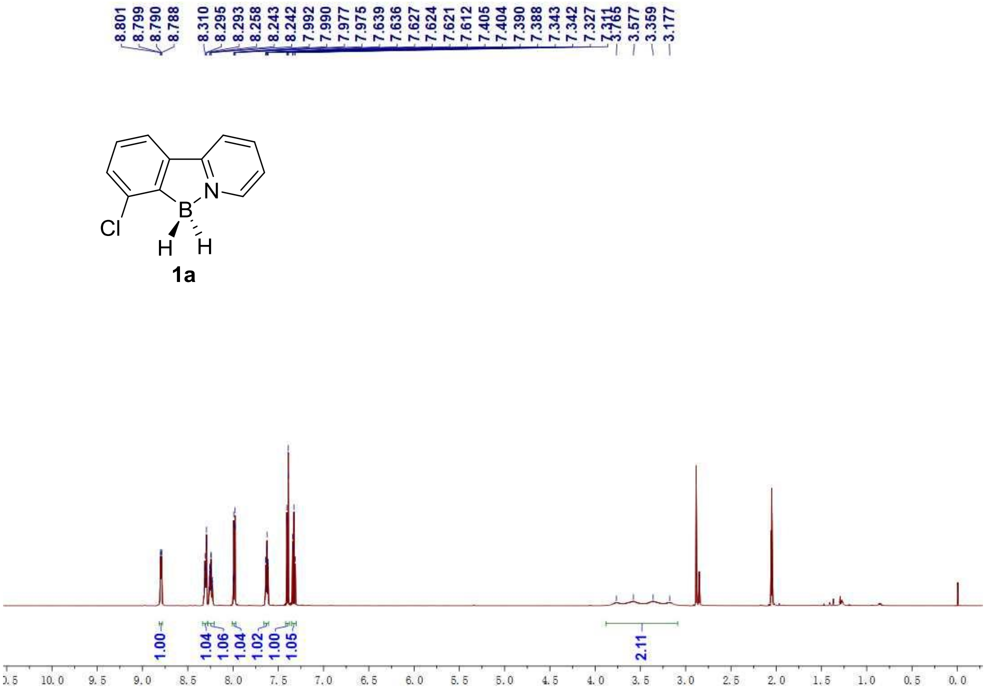

Supplementary Figure 20. ^{1}H NMR spectrum of compound 1a

^{13}C NMR (126 MHz, room temperature, $CDCl_3$)

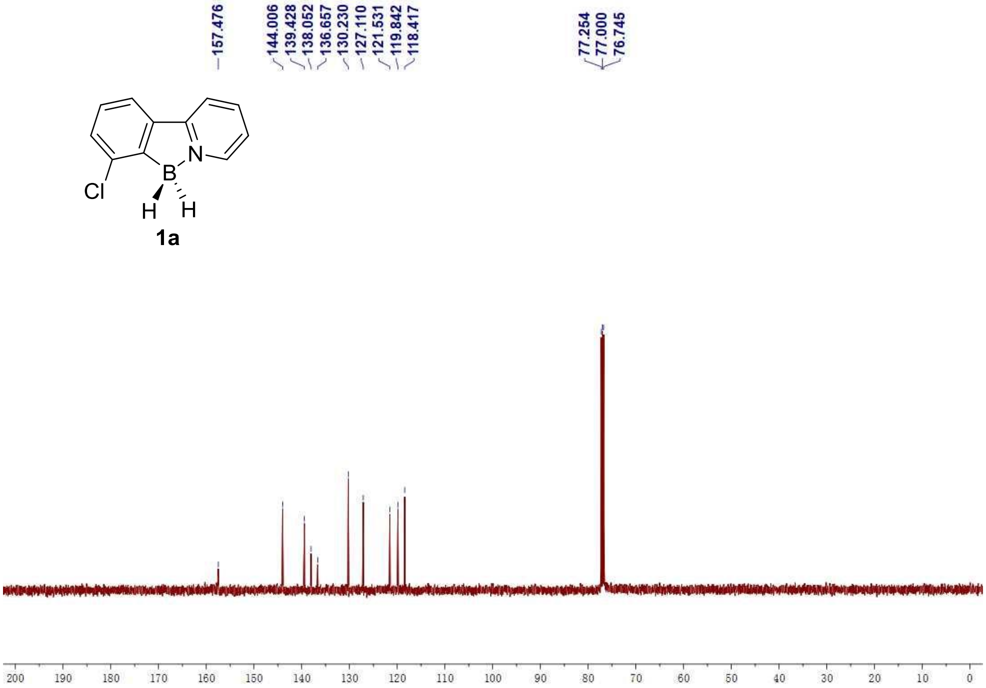

Supplementary Figure 21. ^{13}C NMR spectrum of compound 1a

^{11}B NMR (160 MHz, room temperature, $(CD_3)_2CO$)

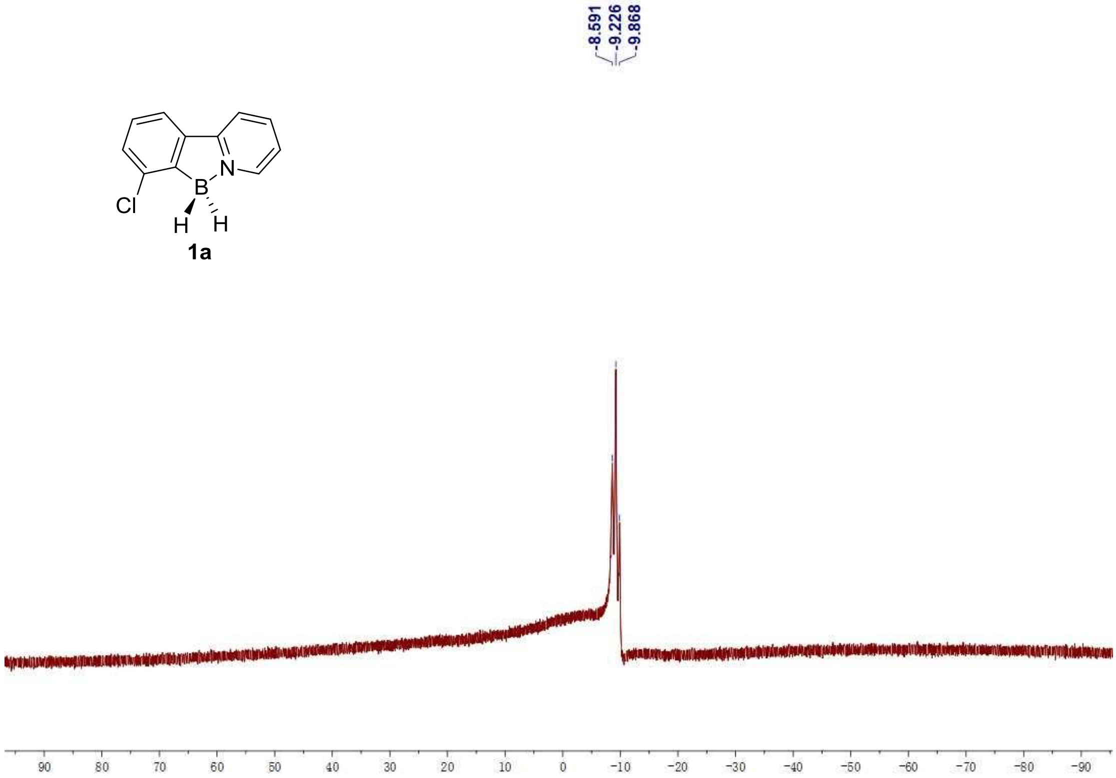

Supplementary Figure 22. ^{11}B NMR spectrum of compound 1a

6H-5λ^4-benzo[3,4][1,2]azaborolo[1,5-a]pyridine(1b)

^{1}H NMR (500 MHz, room temperature, $(CD_3)_2CO$)

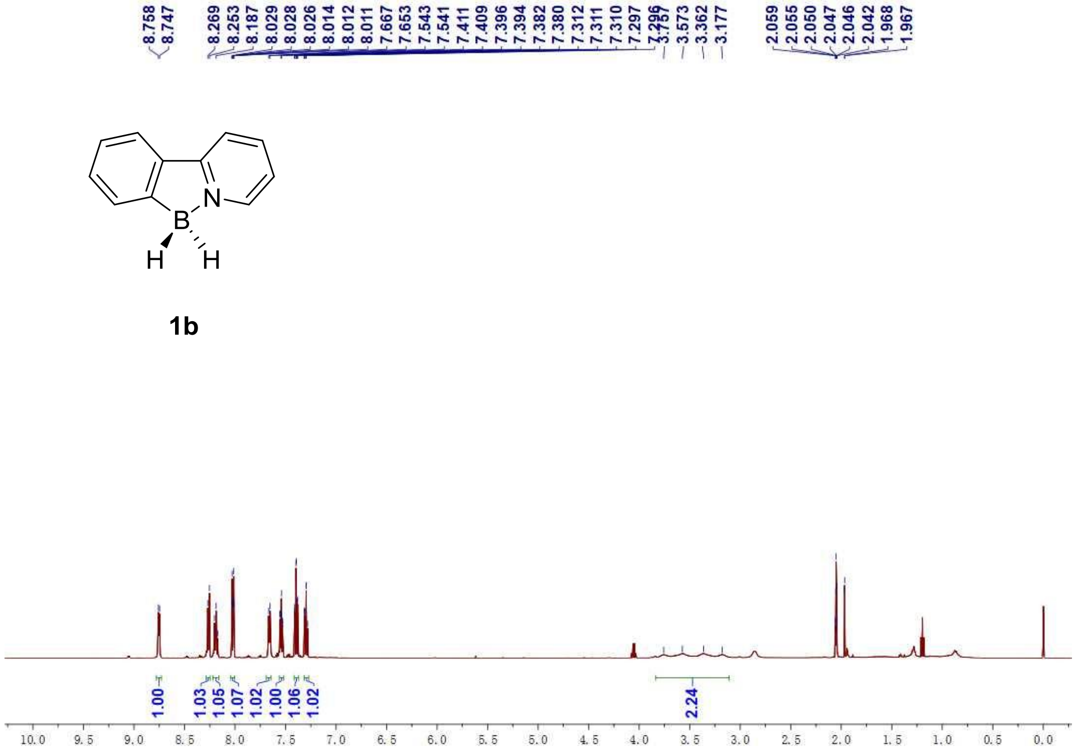

Supplementary Figure 23. ^{1}H NMR spectrum of compound 1b

^{13}C NMR (126 MHz, room temperature, $(CD_3)_2CO$)

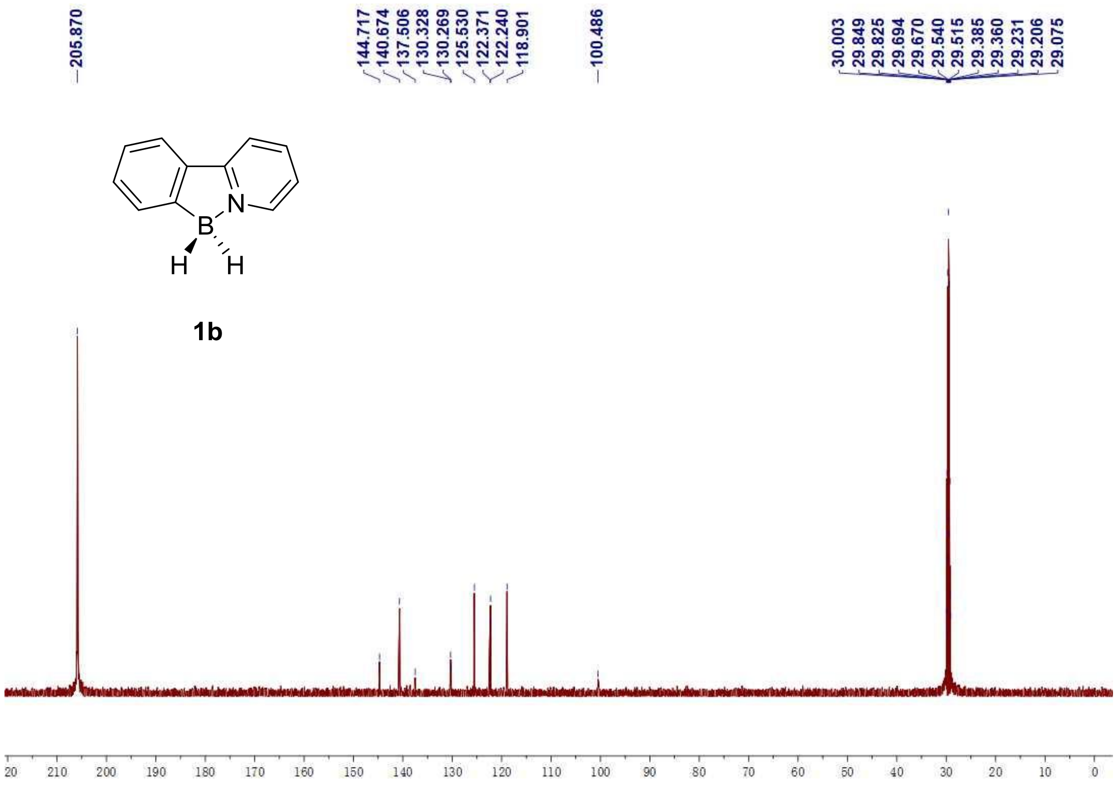

Supplementary Figure 24. ^{13}C NMR spectrum of compound 1b

^{11}B NMR (160 MHz, room temperature, $(CD_3)_2CO$)

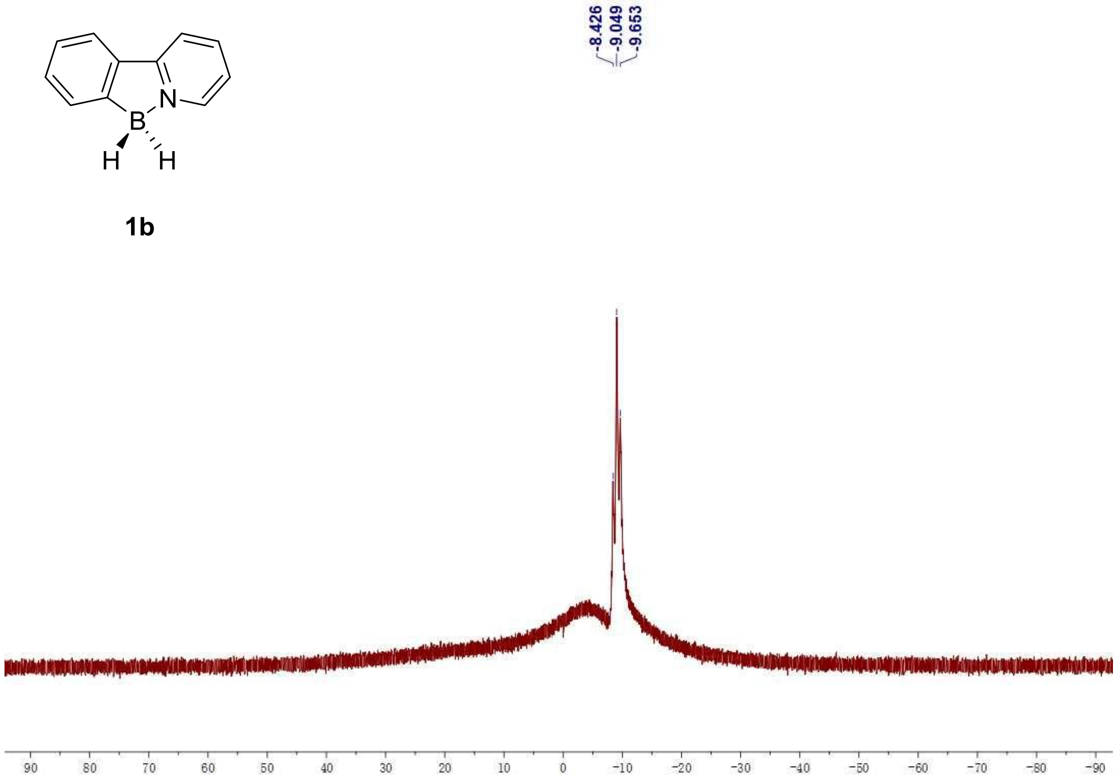

Supplementary Figure 25. ^{11}B NMR spectrum of compound 1b

7-chloro-6H-5λ[4]-benzo[3,4][1,2]azaborolo[1,5-a]pyridine(1c)

^{1}H NMR (500 MHz, room temperature, $CDCl_3$)

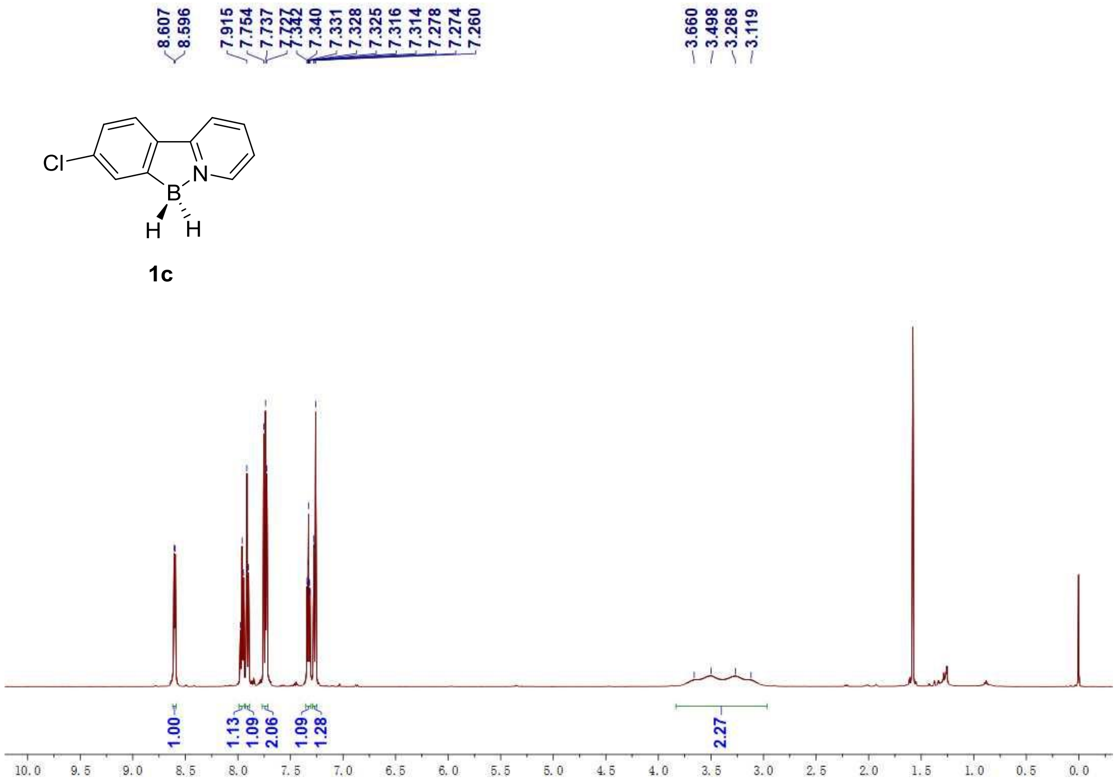

Supplementary Figure 26. ^{1}H NMR spectrum of compound 1c

^{13}C NMR (126 MHz, room temperature, $CDCl_3$)

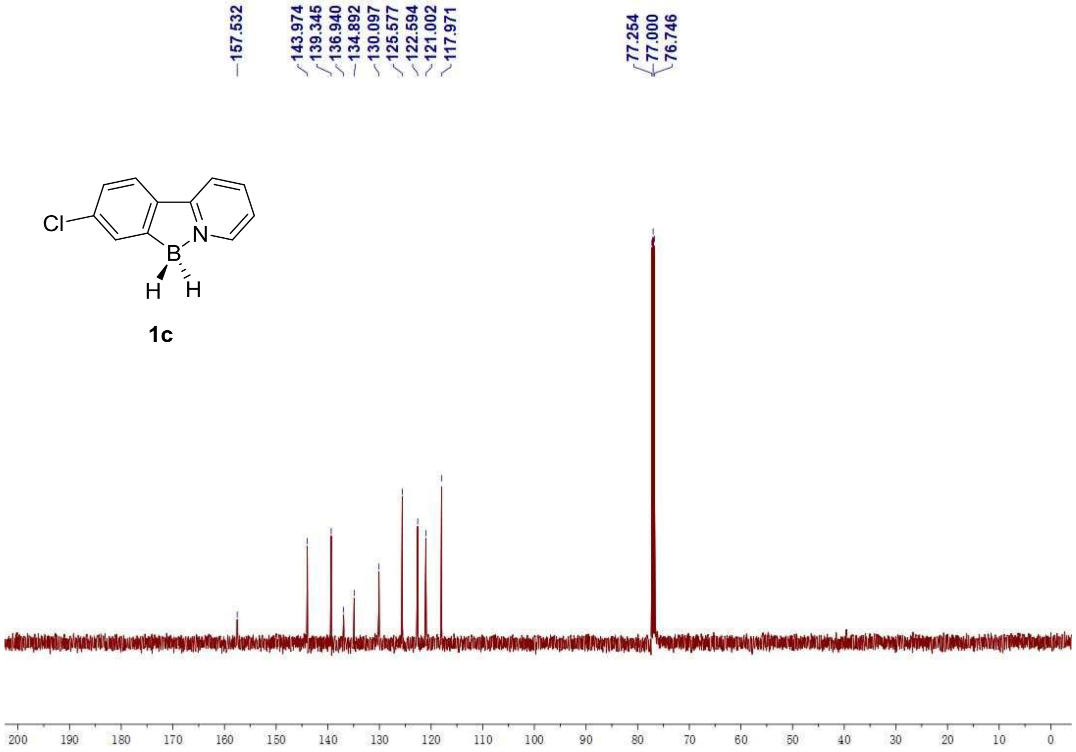

Supplementary Figure 27. ^{13}C NMR spectrum of compound 1c

^{11}B NMR (160 MHz, room temperature, $CDCl_3$)

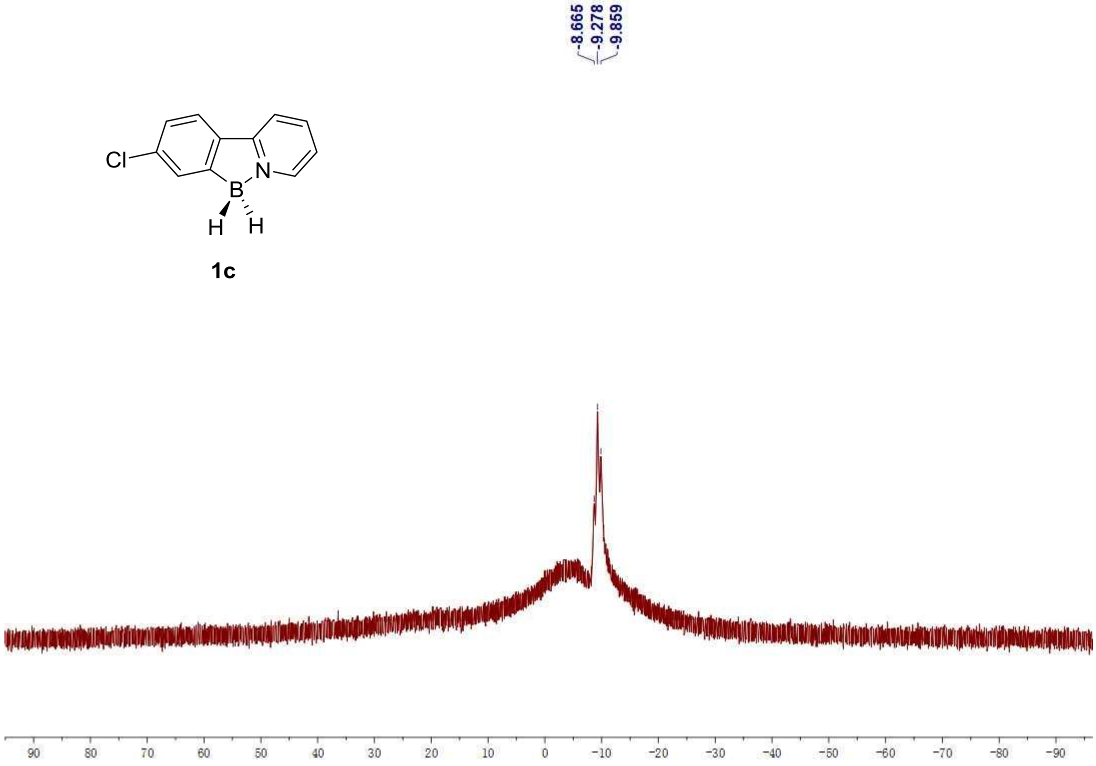

Supplementary Figure 28. ^{11}B NMR spectrum of compound 1c

9-chloro-6H-5λ[4]-benzo[3,4][1,2]azaborolo[1,5-a]pyridine(1d)

^{1}H NMR (500 MHz, room temperature, $(CD_3)_2CO$)

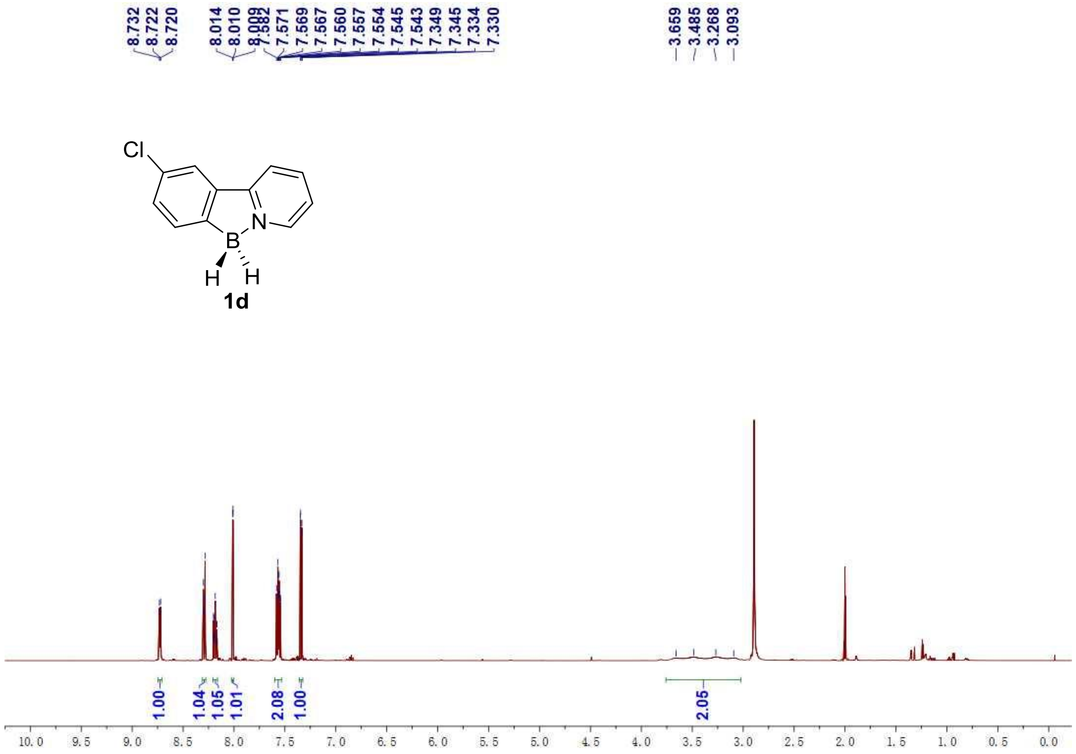

Supplementary Figure 29. ^{1}H NMR spectrum of compound 1d

^{13}C NMR (126 MHz, room temperature, $CDCl_3$)

1d

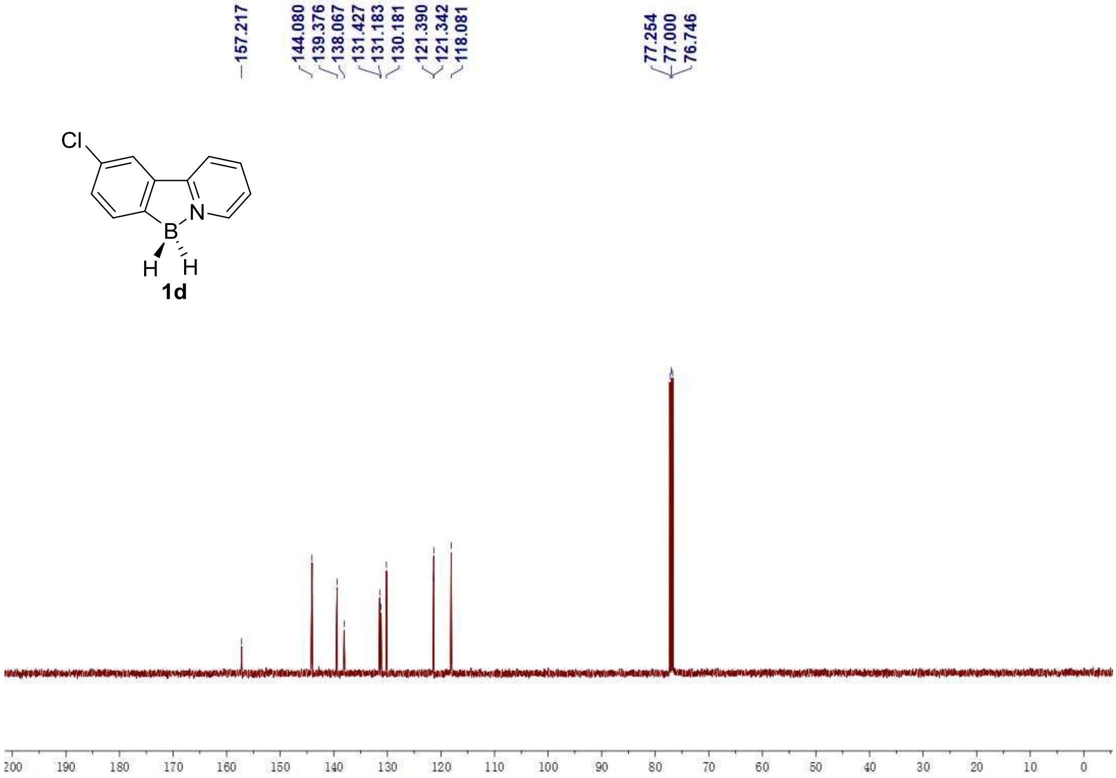

Supplementary Figure 30. ^{13}C NMR spectrum of compound 1d

^{11}B NMR (160 MHz, room temperature, $(CD_3)_2CO$)

1d

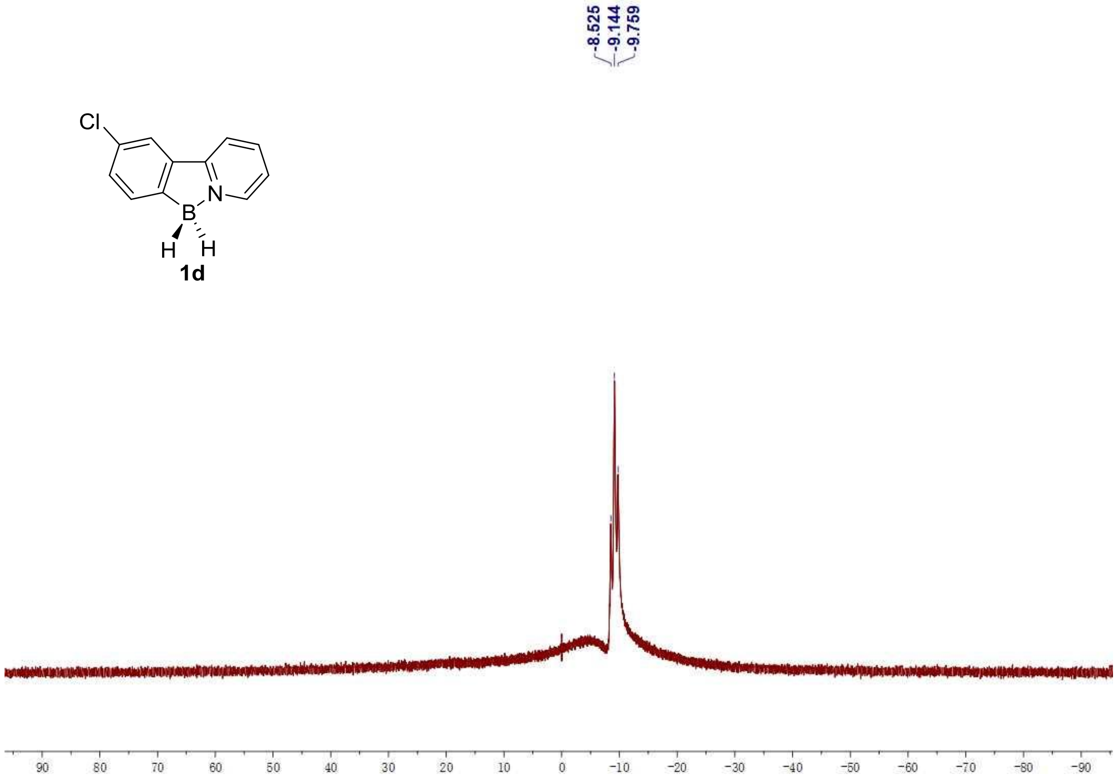

Supplementary Figure 31. ^{11}B NMR spectrum of compound 1d

10-fluoro-6H-5λ⁴-benzo[3,4][1,2]azaborolo[1,5-a]pyridine (1e)

^{1}H NMR (500 MHz, room temperature, $CDCl_3$)

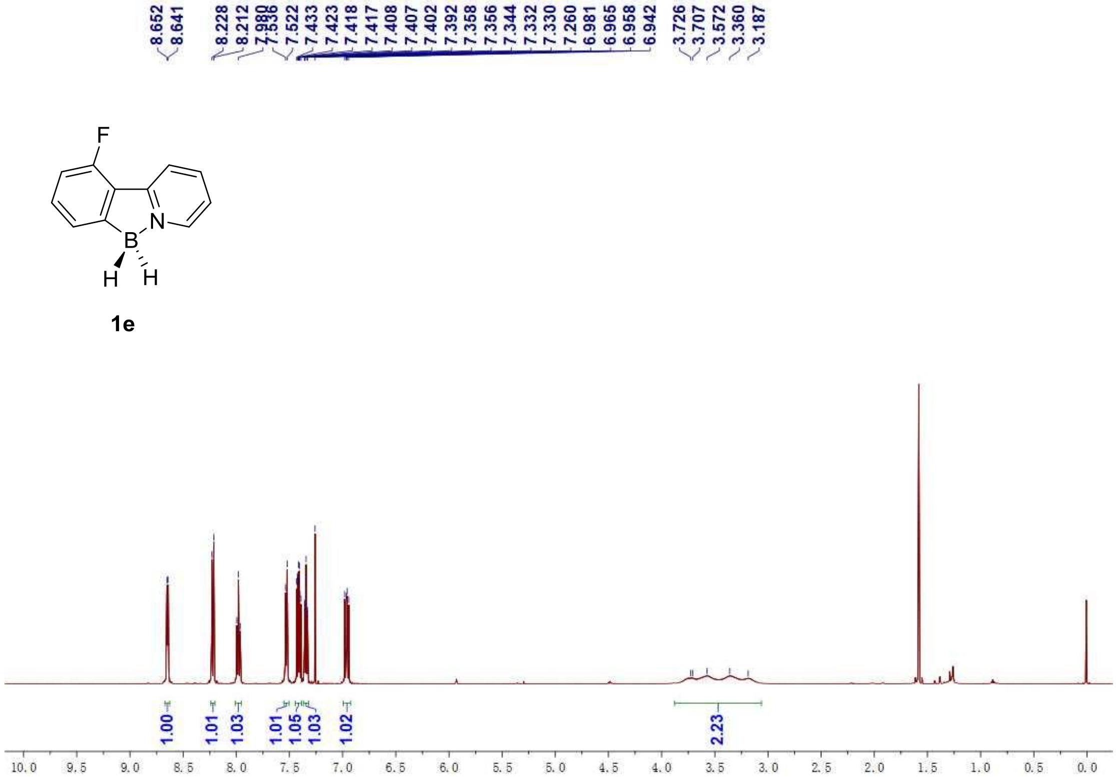

Supplementary Figure 32. ^{1}H NMR spectrum of compound 1e

^{13}C NMR (126 MHz, room temperature, $CDCl_3$)

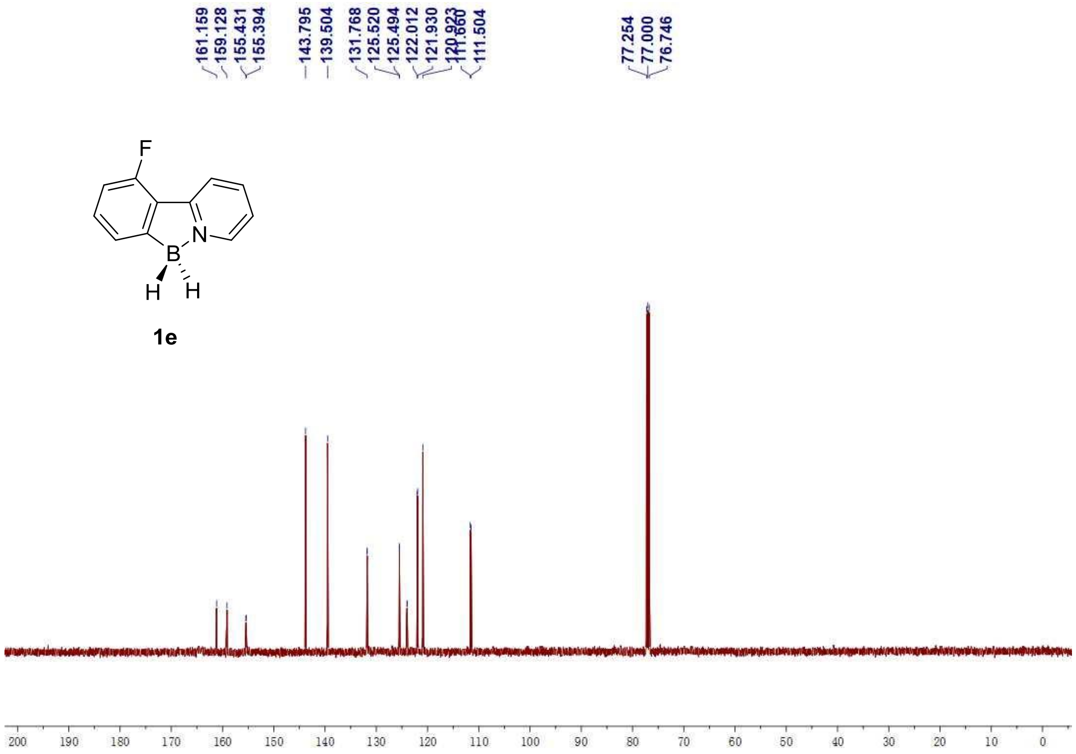

Supplementary Figure 33. ^{13}C NMR spectrum of compound 1e

^{11}B NMR (160 MHz, **room temperature**, $CDCl_3$)

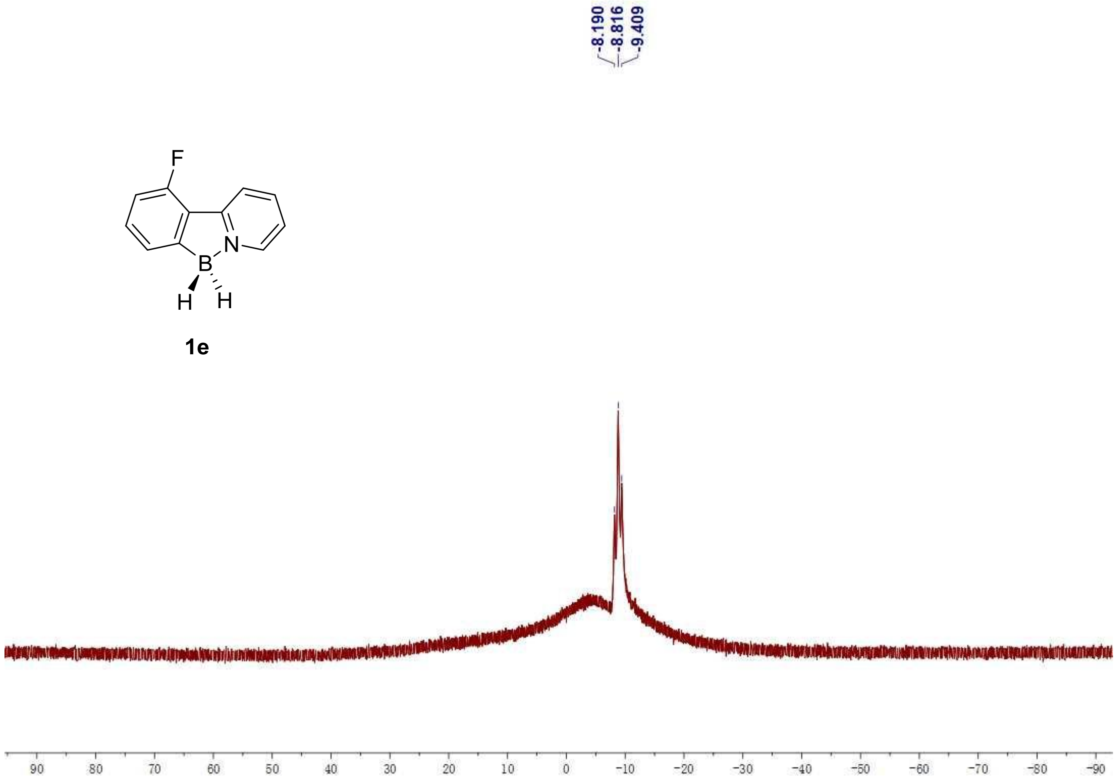

Supplementary Figure 34. ^{11}B NMR spectrum of compound 1e

^{19}F NMR (376 MHz, **room temperature**, $CDCl_3$)

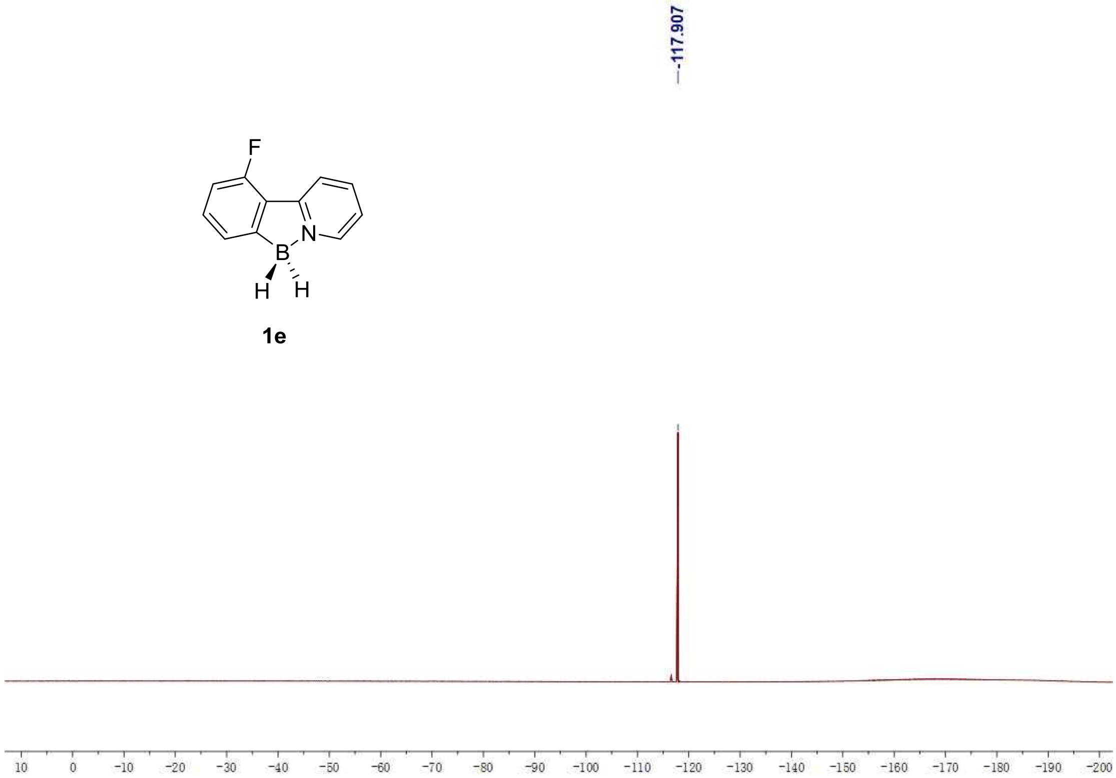

Supplementary Figure 35. ^{19}F NMR spectrum of compound 1e

4-methyl-6H-5λ[4]-benzo[3,4][1,2]azaborolo[1,5-a]pyridine (1f)

^{1}H NMR (500 MHz, room temperature, $CDCl_3$)

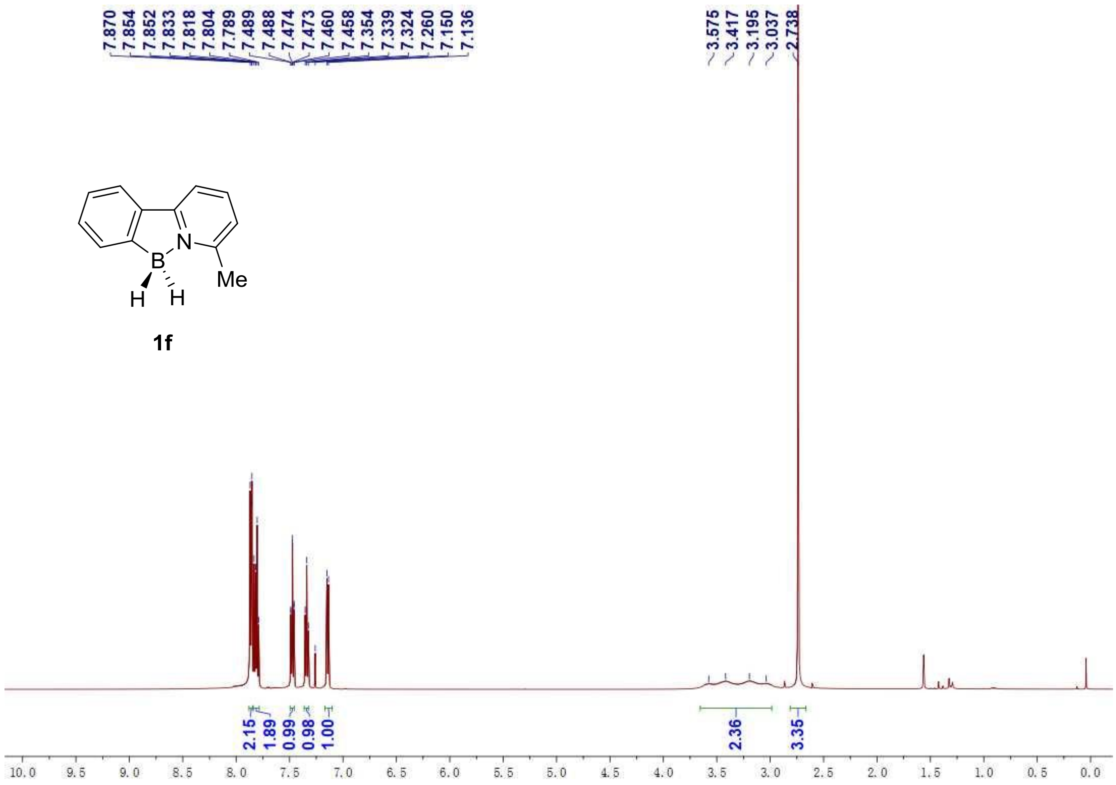

Supplementary Figure 36. ^{1}H NMR spectrum of compound 1f

^{13}C NMR (126 MHz, room temperature, $CDCl_3$)

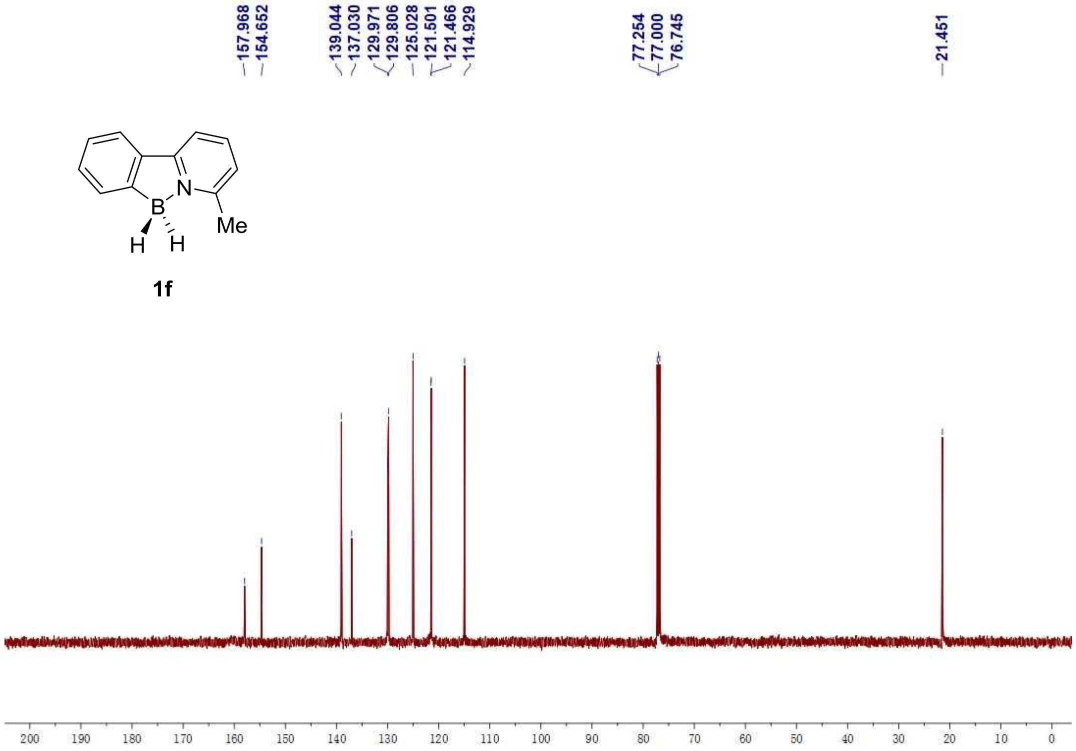

Supplementary Figure 37. ^{13}C NMR spectrum of compound 1f

^{11}B NMR (160 MHz, room temperature, $CDCl_3$)

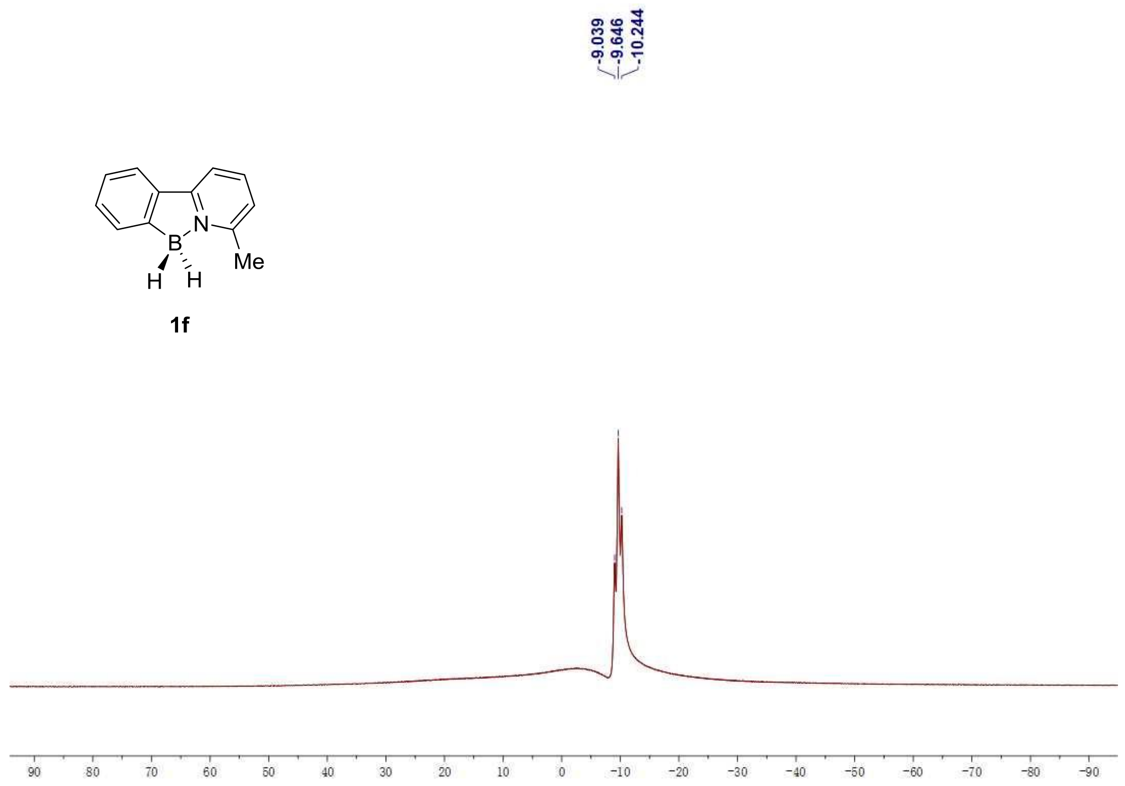

Supplementary Figure 38. ^{11}B NMR spectrum of compound 1f

3-methyl-6H-5 ^{4}-benzo[3,4][1,2]azaborolo[1,5-a]pyridine (1g)

^{1}H NMR (500 MHz, room temperature, $CDCl_3$)

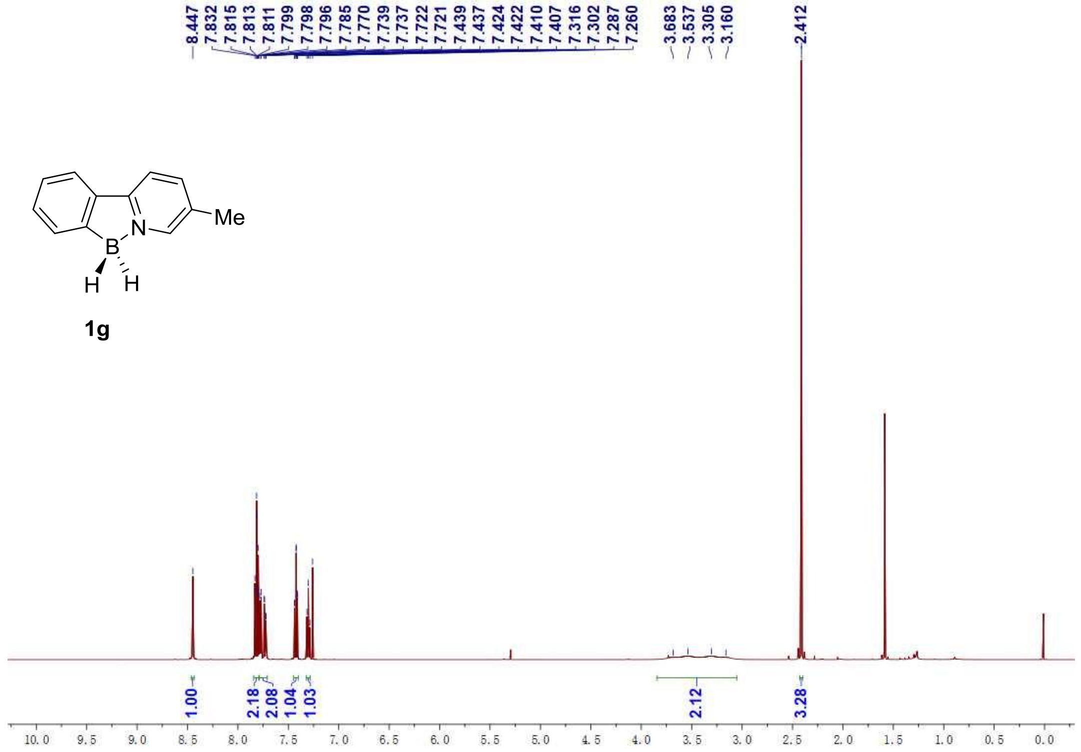

Supplementary Figure 39. ^{1}H NMR spectrum of compound 1g

^{13}C NMR (126 MHz, room temperature, $CDCl_3$)

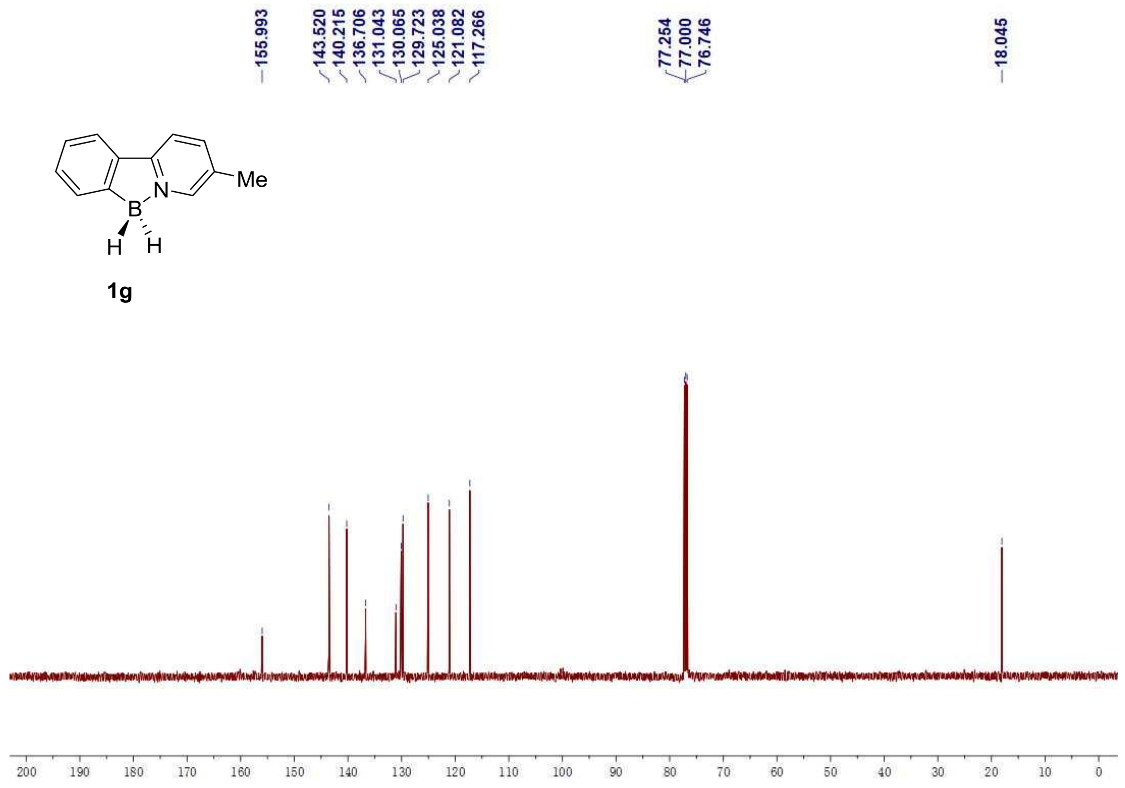

Supplementary Figure 40. ^{13}C NMR spectrum of compound 1g

^{11}B NMR (160 MHz, room temperature, $CDCl_3$)

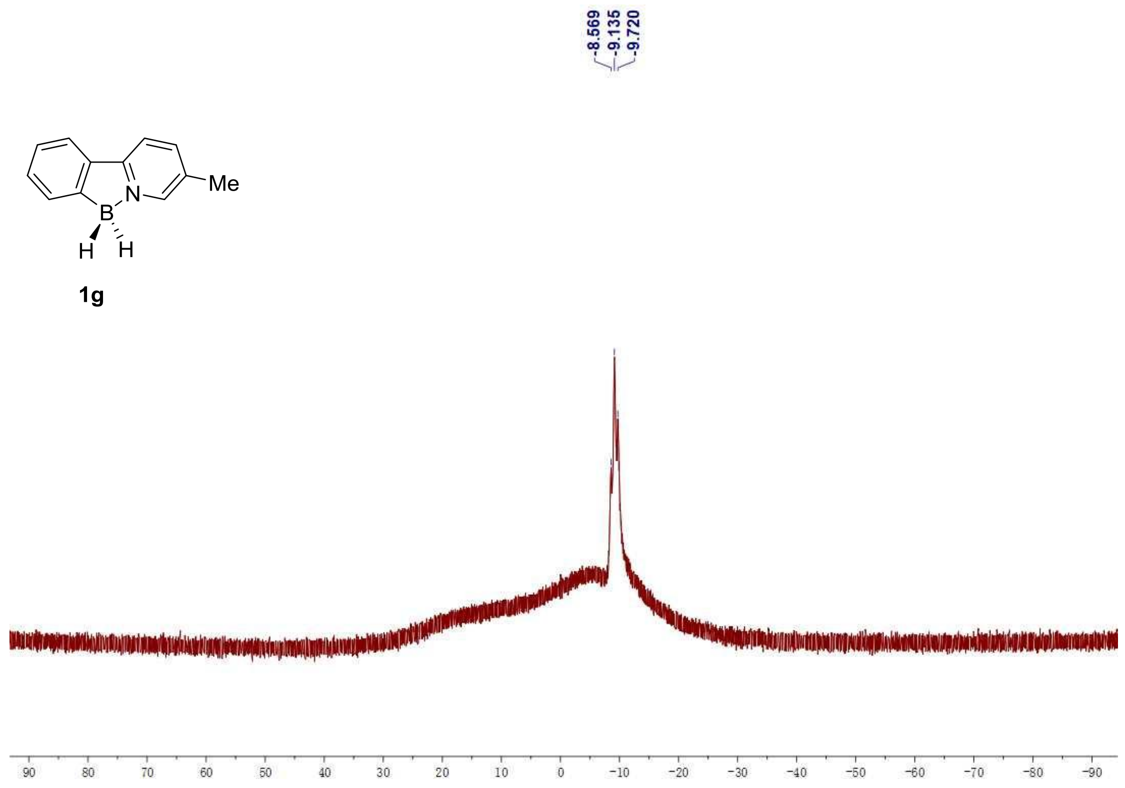

Supplementary Figure 41. ^{11}B NMR spectrum of compound 1g

2-methyl-6H-5 ^{4}-benzo[3,4][1,2]azaborolo[1,5-a]pyridine (1h)

^{1}H NMR (500 MHz, room temperature, $CDCl_3$)

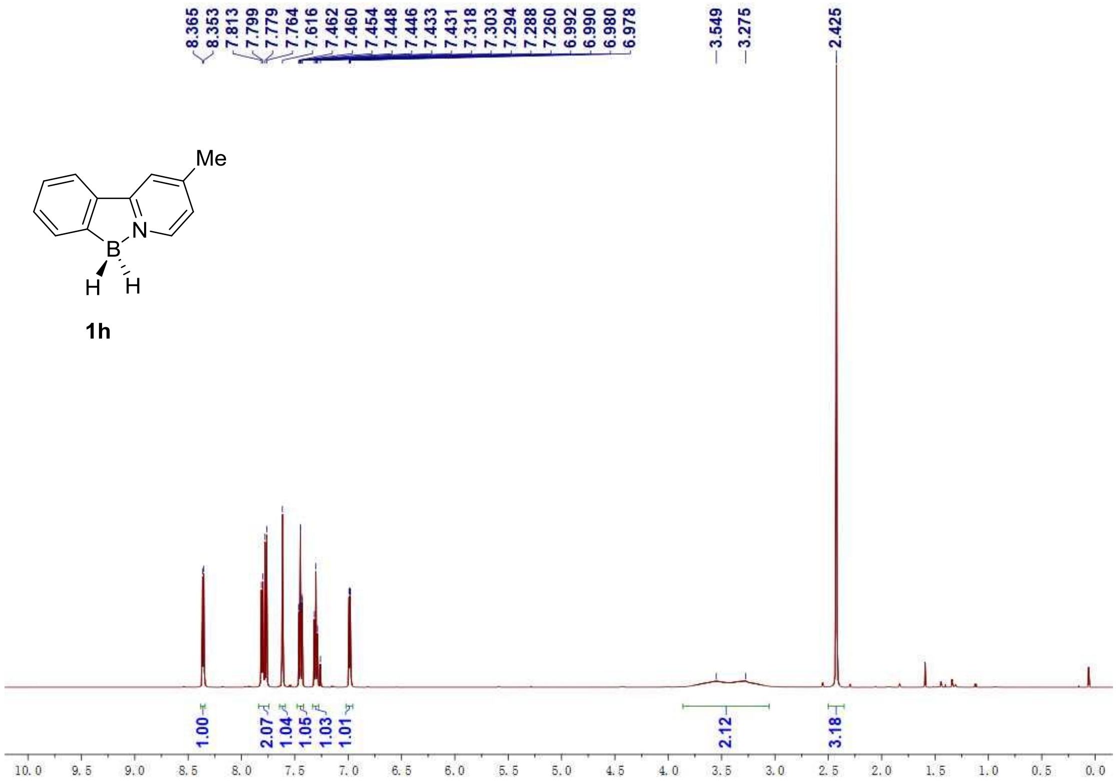

Supplementary Figure 42. ^{1}H NMR spectrum of compound 1h

^{13}C NMR (126 MHz, room temperature, $CDCl_3$)

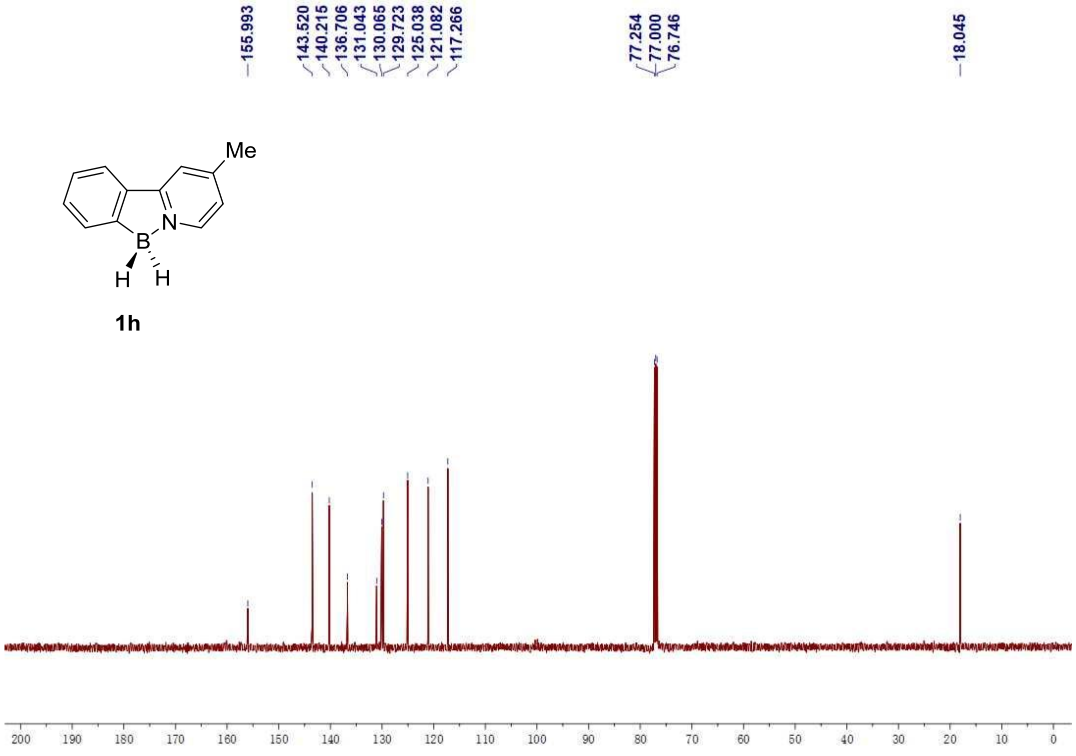

Supplementary Figure 43. ^{13}C NMR spectrum of compound 1h

^{11}B NMR (160 MHz, room temperature, $CDCl_3$)

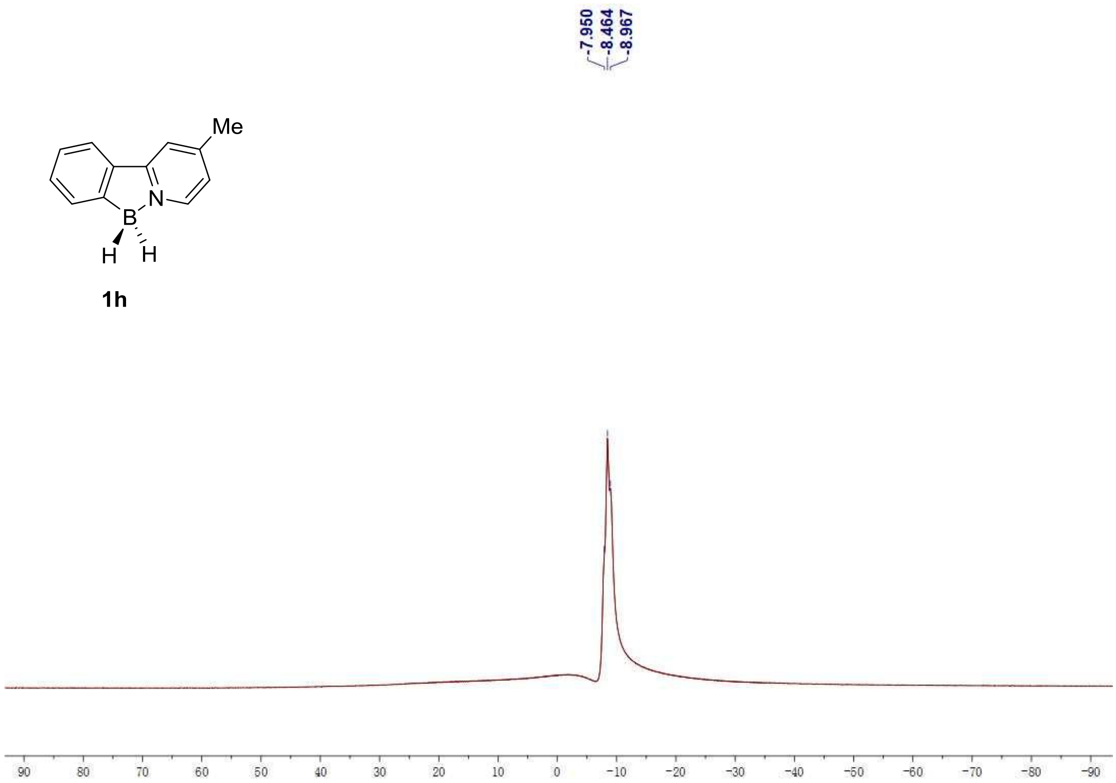

Supplementary Figure 44. ^{11}B NMR spectrum of compound 1h

7-fluoro-6H-5 ^{4}-benzo[3,4][1,2]azaborolo[1,5-a]pyridine(1i)

^{1}H NMR (400 MHz, room temperature, $CDCl_3$)

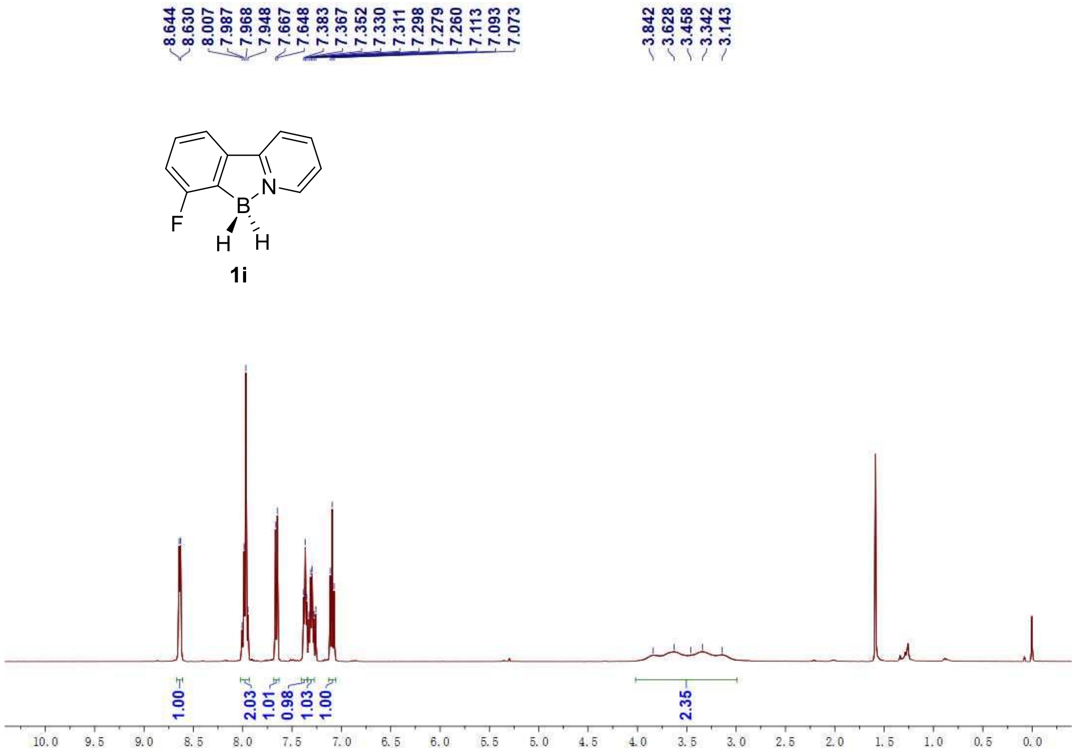

Supplementary Figure 45. ^{1}H NMR spectrum of compound 1i

^{13}C NMR (101 MHz, room temperature, $CDCl_3$)

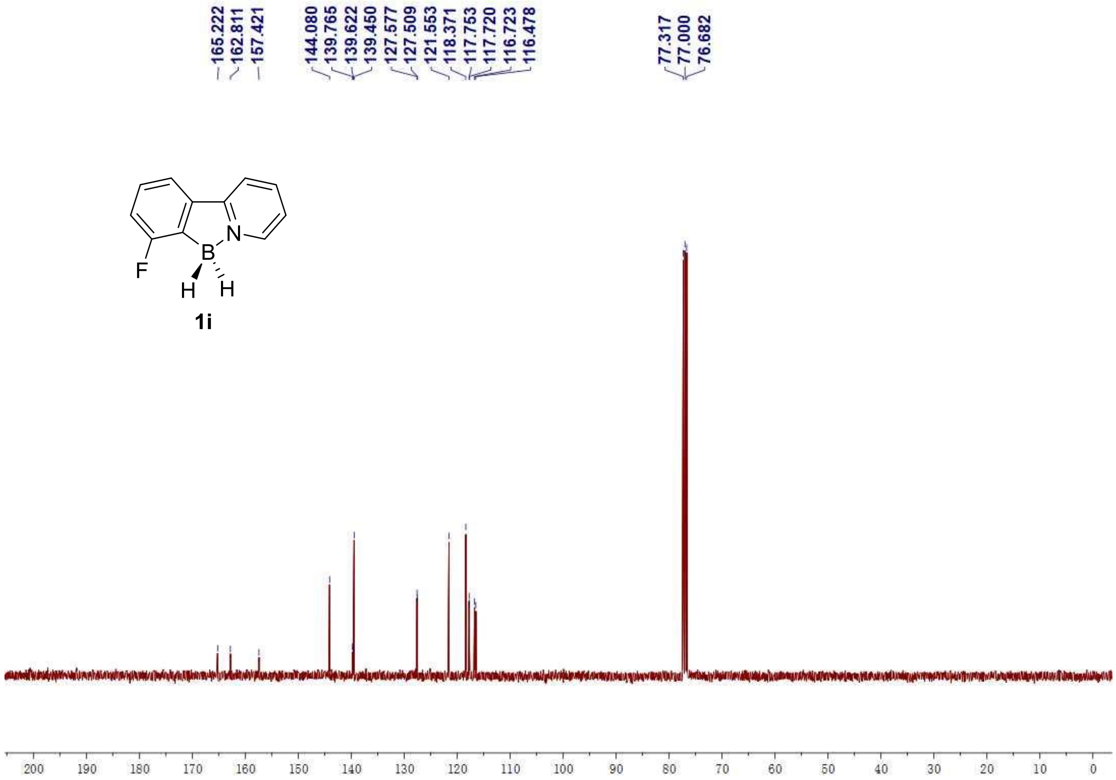

Supplementary Figure 46. ^{13}**C NMR spectrum of compound 1i**

^{11}B NMR (128 MHz, room temperature, $CDCl_3$)

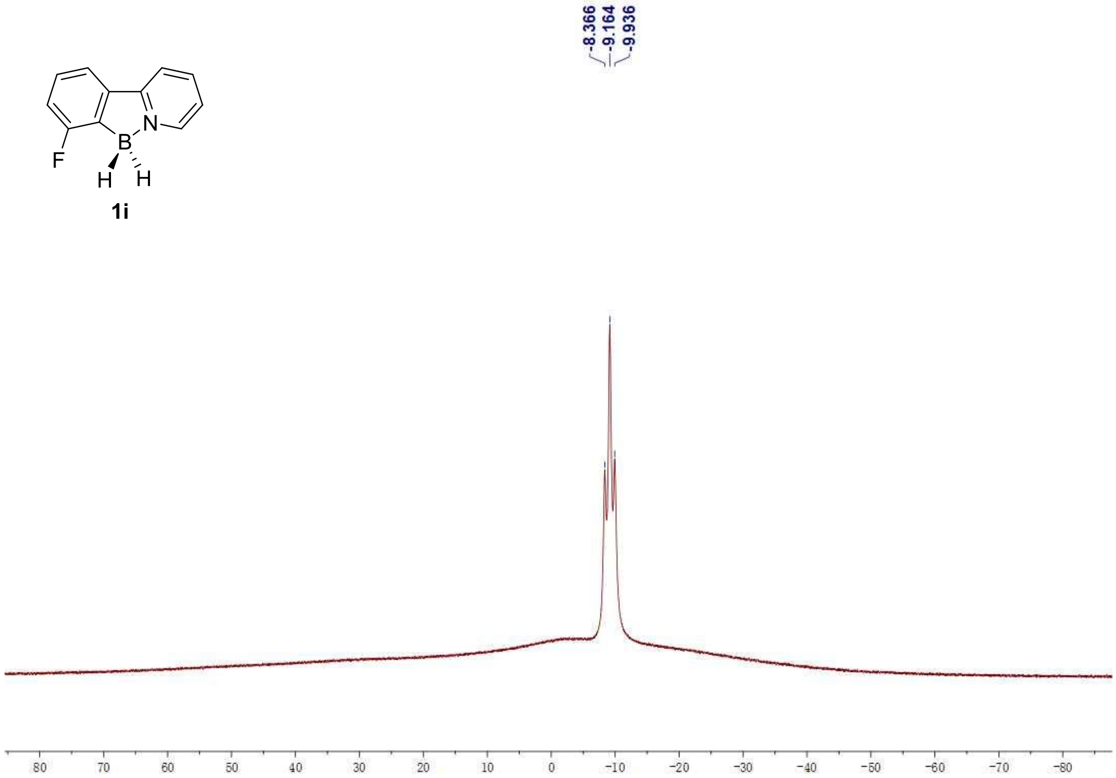

Supplementary Figure 47. ^{11}**B NMR spectrum of compound 1i**

^{19}F NMR (376 MHz, room temperature, $CDCl_3$)

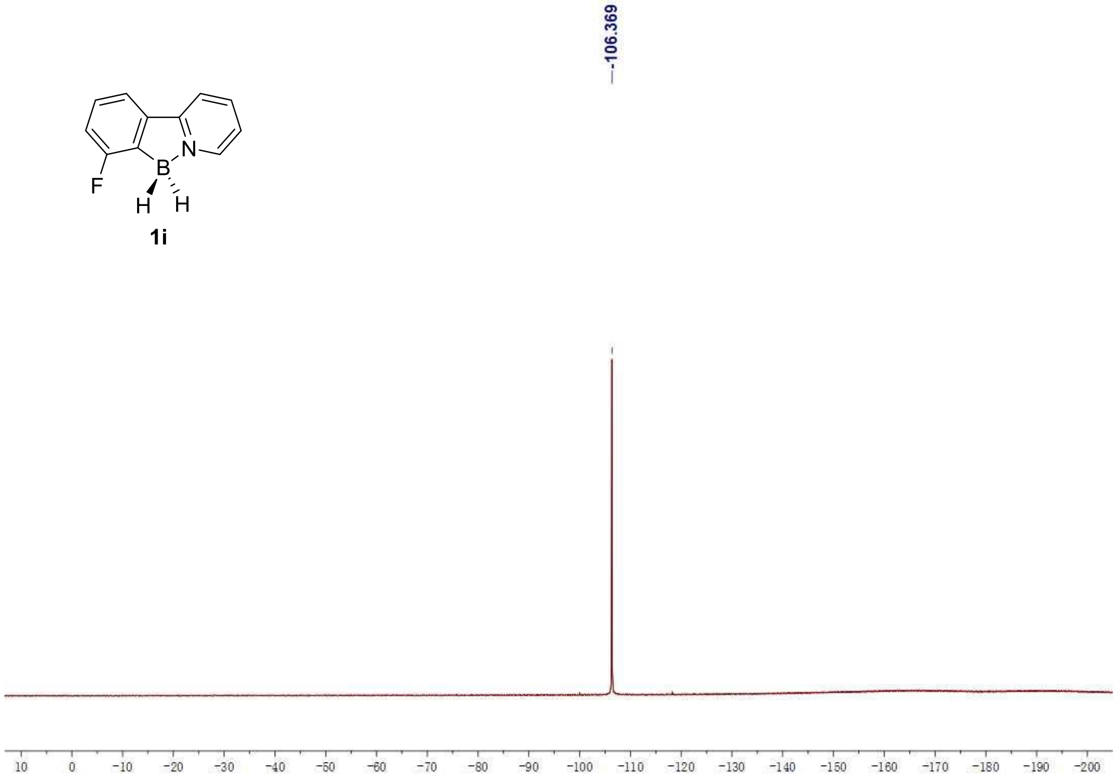

Supplementary Figure 48. ^{19}F NMR spectrum of compound 1i

7-bromo-6H-5 ^{4}-benzo[3,4][1,2]azaborolo[1,5-a]pyridine (1j)

^{1}H NMR (400 MHz, room temperature, $CDCl_3$)

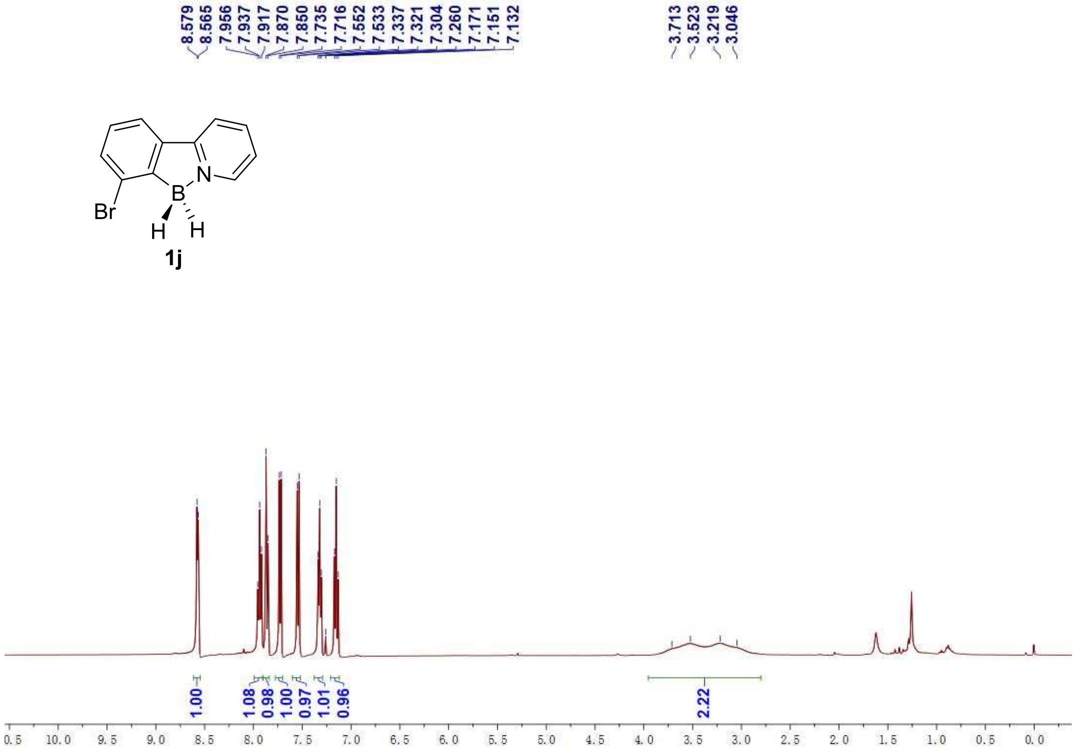

Supplementary Figure 49. ^{1}H NMR spectrum of compound 1j

^{13}C NMR (101 MHz, room temperature, $CDCl_3$)

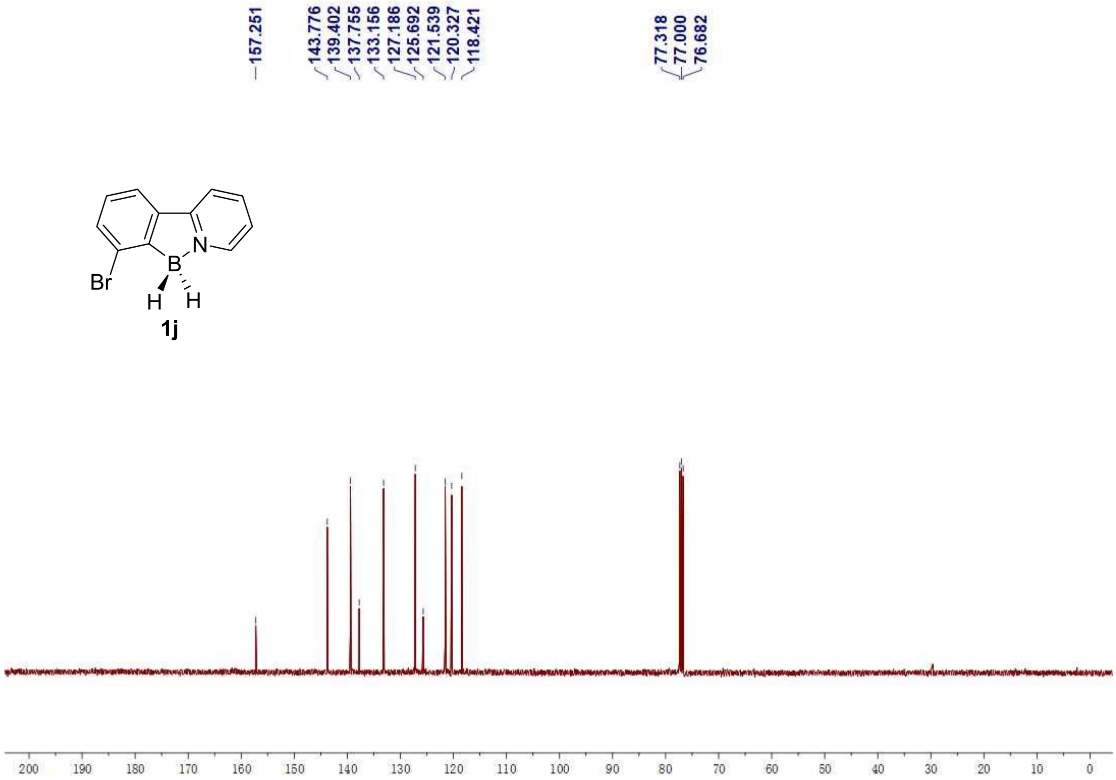

Supplementary Figure 50. ^{13}C NMR spectrum of compound 1j

^{11}B NMR (128 MHz, room temperature, $CDCl_3$)

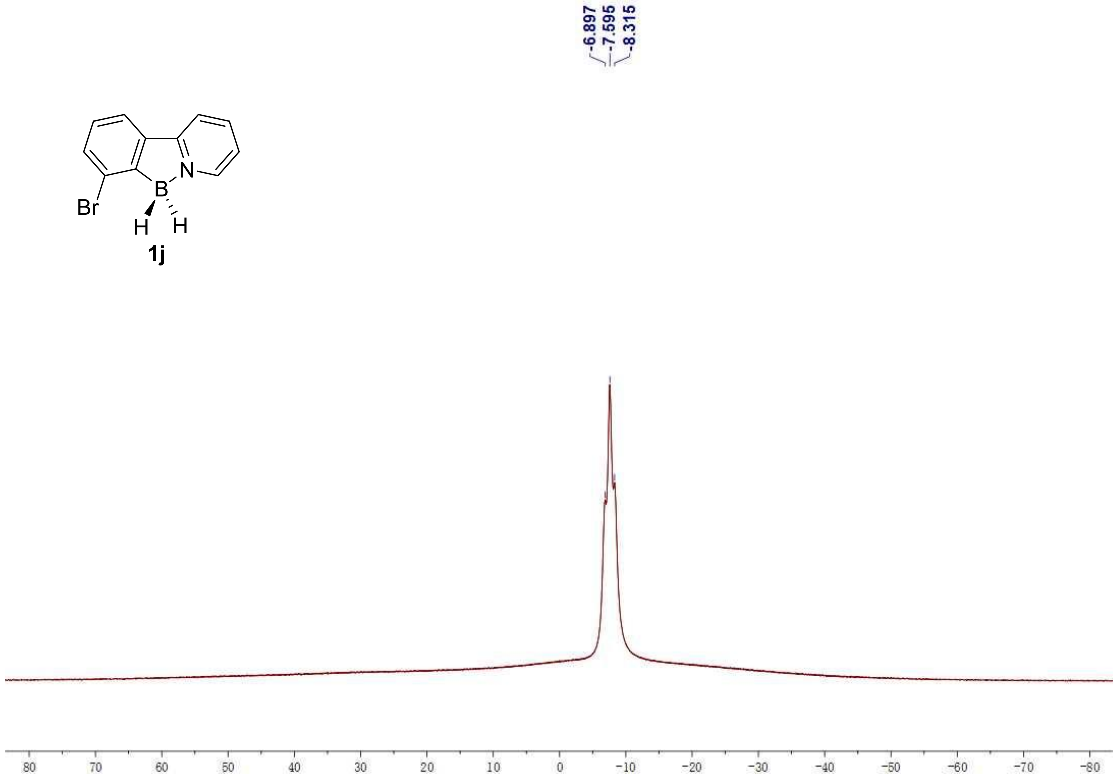

Supplementary Figure 51. ^{11}B NMR spectrum of compound 1j

7-chloro-10-fluoro-6H-5 ^{4}-benzo[3,4][1,2]azaborolo[1,5-a]pyridine(1k)

^{1}H NMR (500 MHz, room temperature, $CDCl_3$)

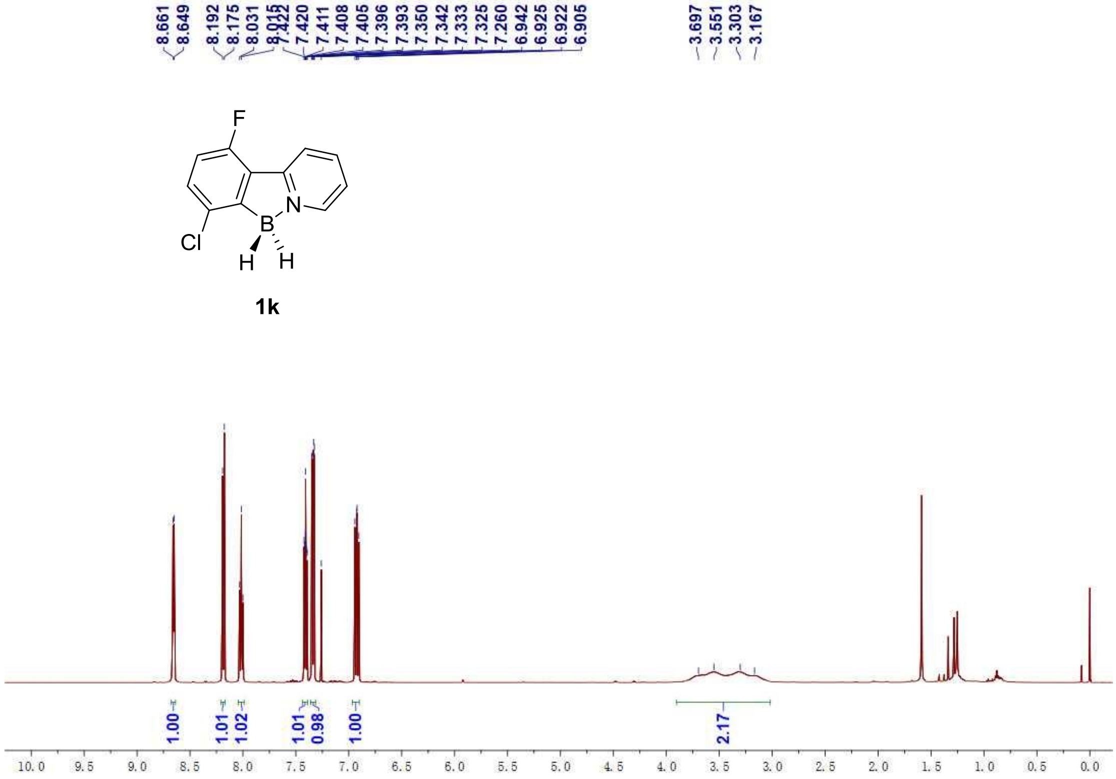

Supplementary Figure 52. ^{1}H NMR spectrum of compound 1k

^{13}C NMR (126 MHz, room temperature, $CDCl_3$)

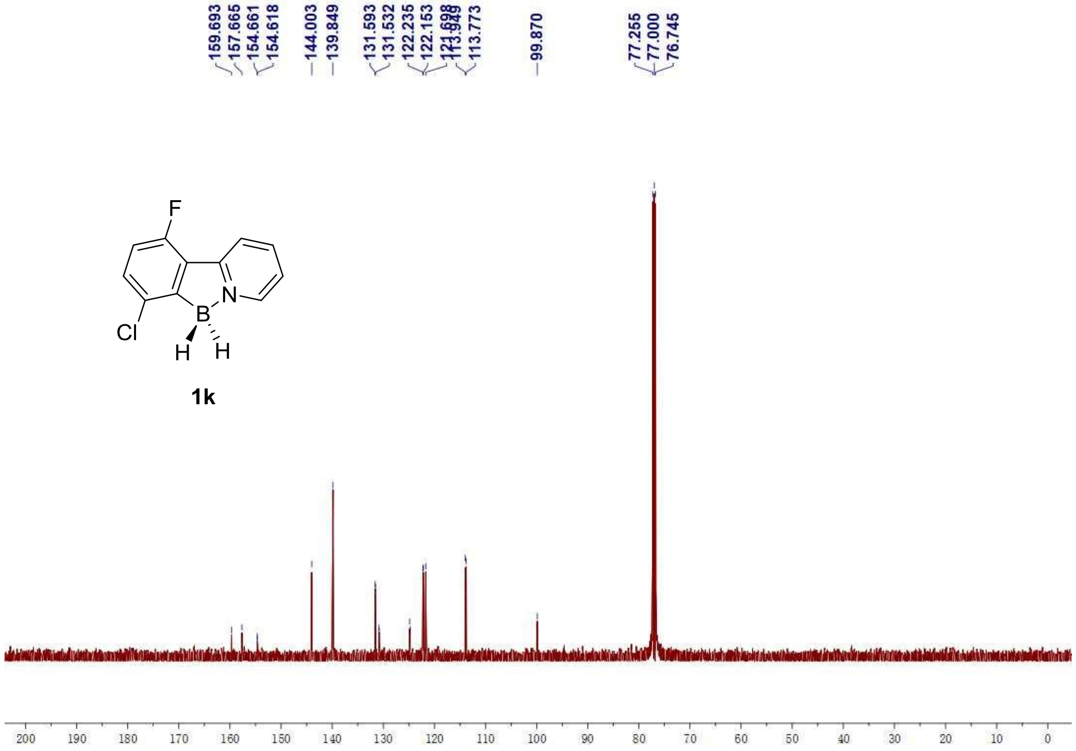

Supplementary Figure 53. ^{13}C NMR spectrum of compound 1k

^{11}B NMR (128 MHz, room temperature, CDCl$_3$)

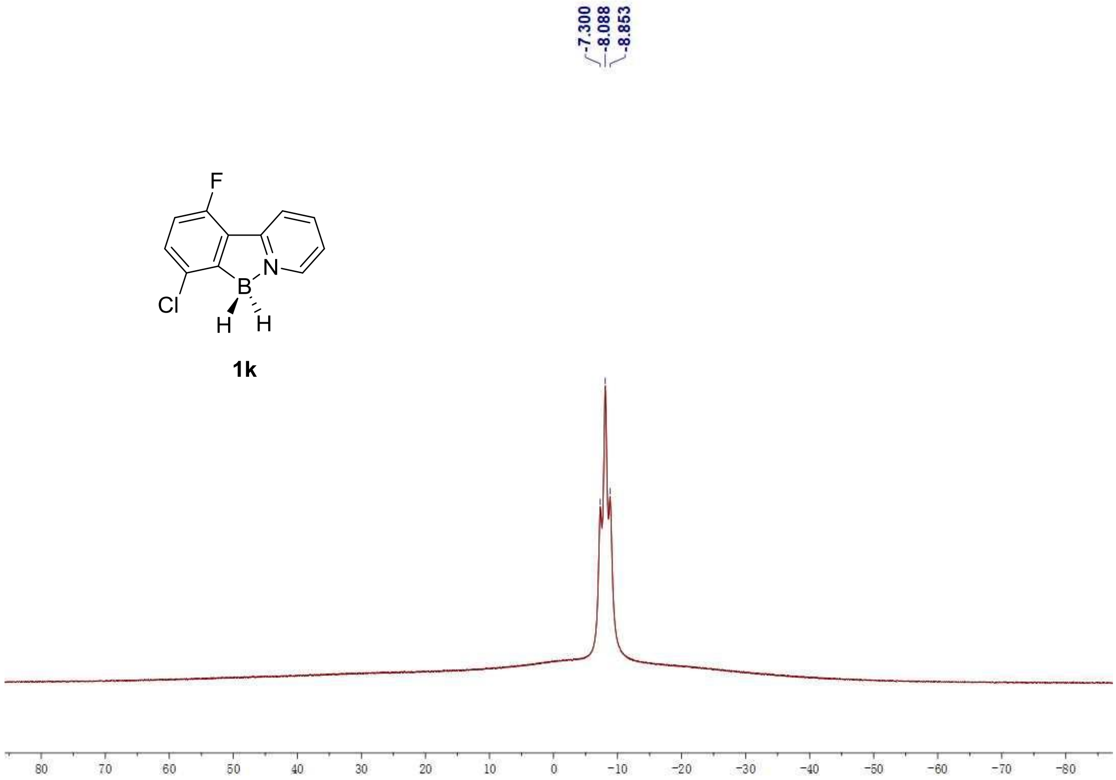

Supplementary Figure 54. ^{11}B NMR spectrum of compound 1k

^{19}F NMR (376 MHz, room temperature, CDCl$_3$)

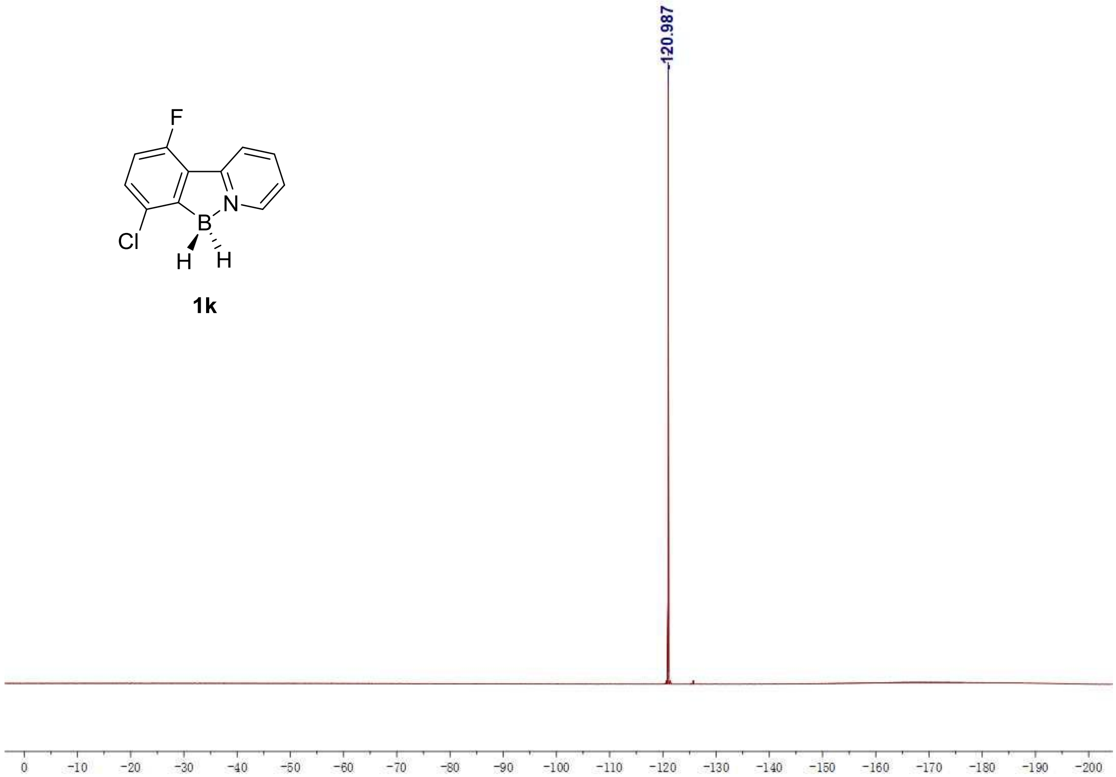

Supplementary Figure 55. ^{19}F NMR spectrum of compound 1k

7-chloro-8-fluoro-6H-5 ^{4}-benzo[3,4][1,2]azaborolo[1,5-a]pyridine(1l)

^{1}H NMR (400 MHz, room temperature, $CDCl_3$)

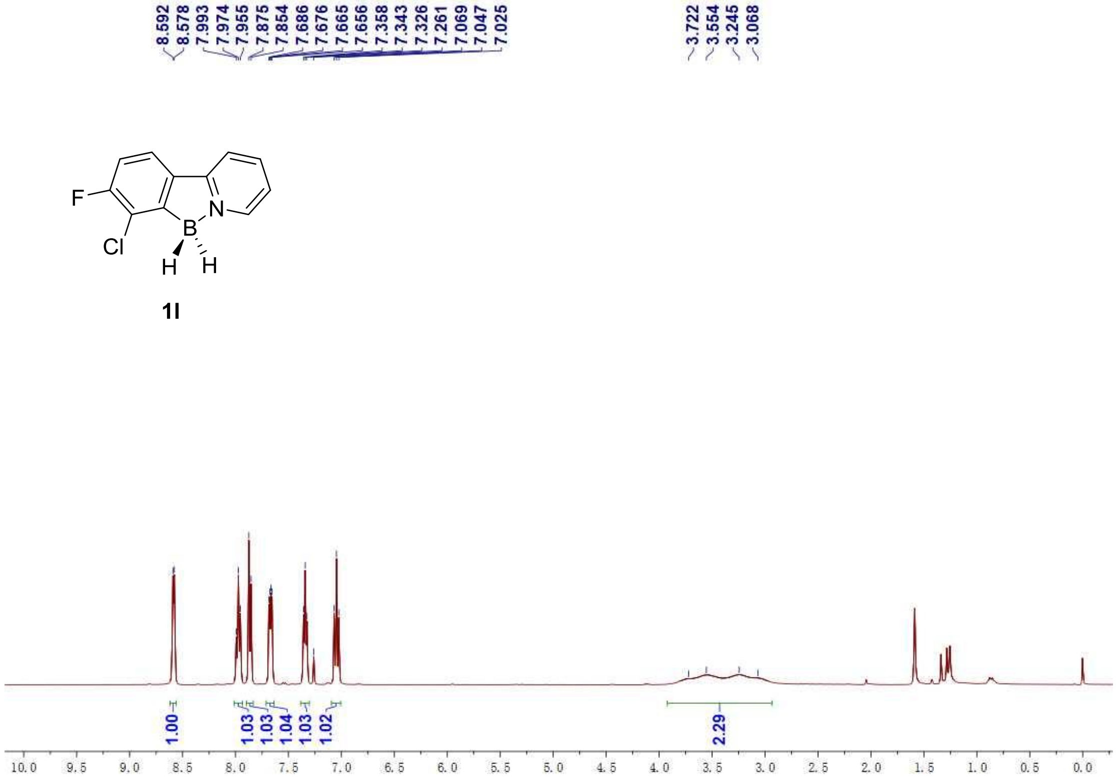

Supplementary Figure 56. ^{1}H NMR spectrum of compound 1l

^{13}C NMR (101 MHz, room temperature, $CDCl_3$)

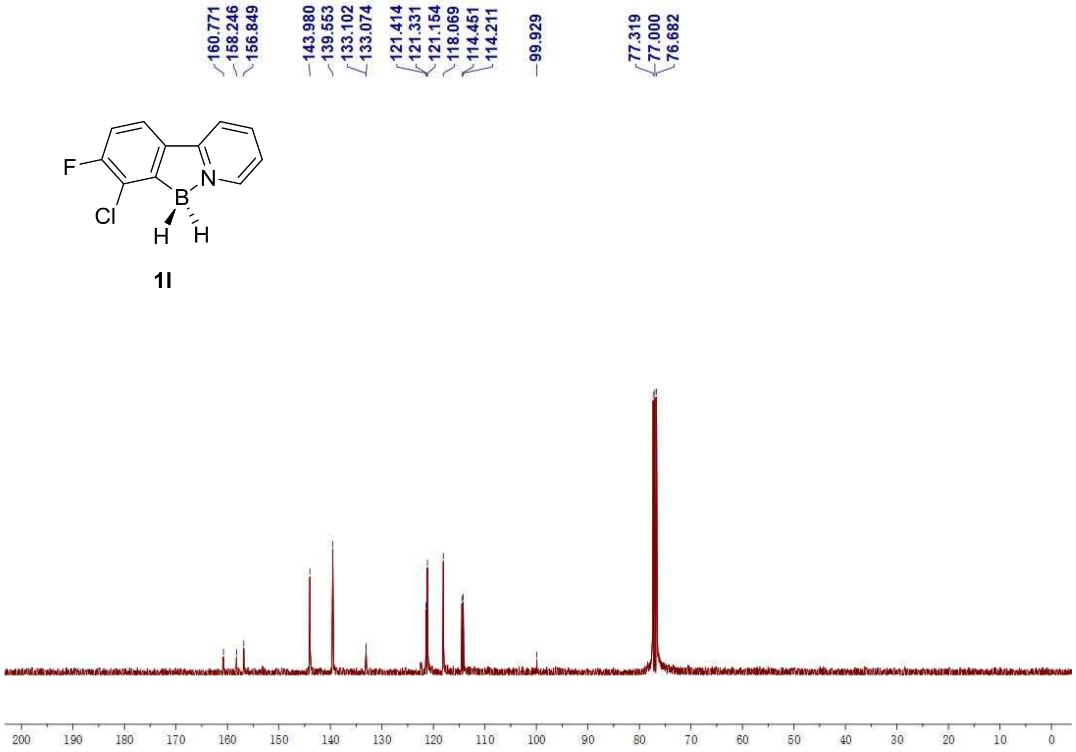

Supplementary Figure 57. ^{13}C NMR spectrum of compound 1l

^{11}B NMR (128 MHz, room temperature, $CDCl_3$)

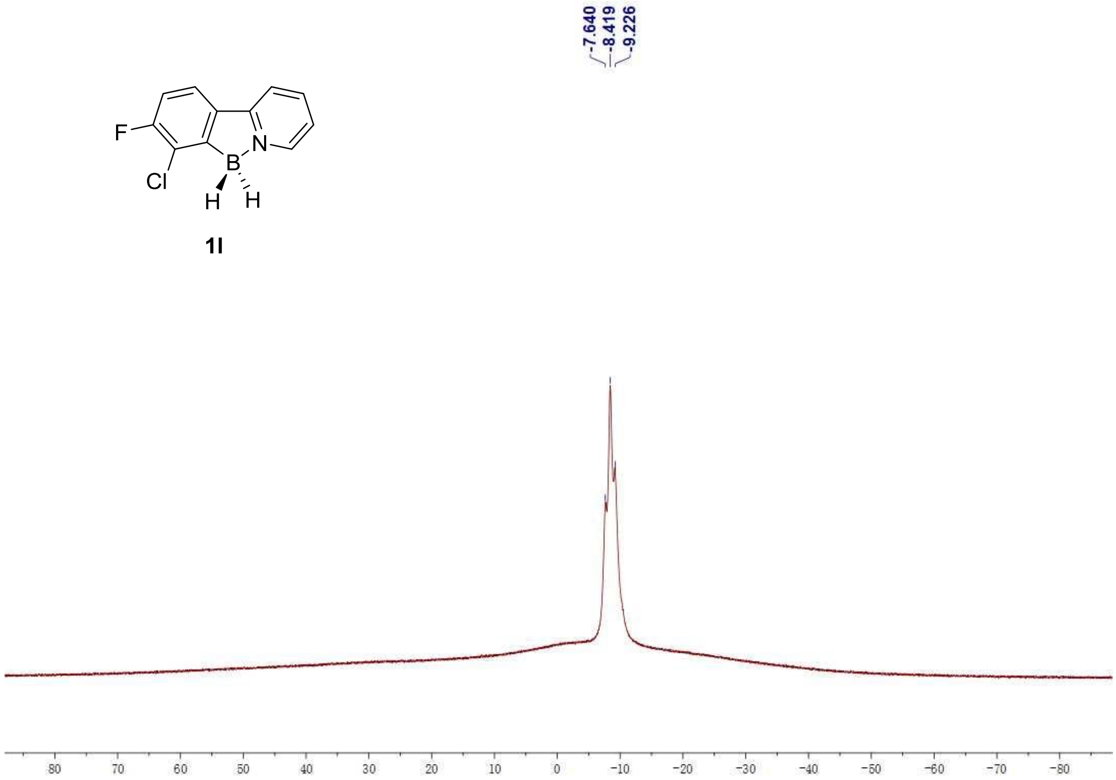

Supplementary Figure 58. ^{11}B NMR spectrum of compound 1l

^{19}F NMR (376 MHz, room temperature, $CDCl_3$)

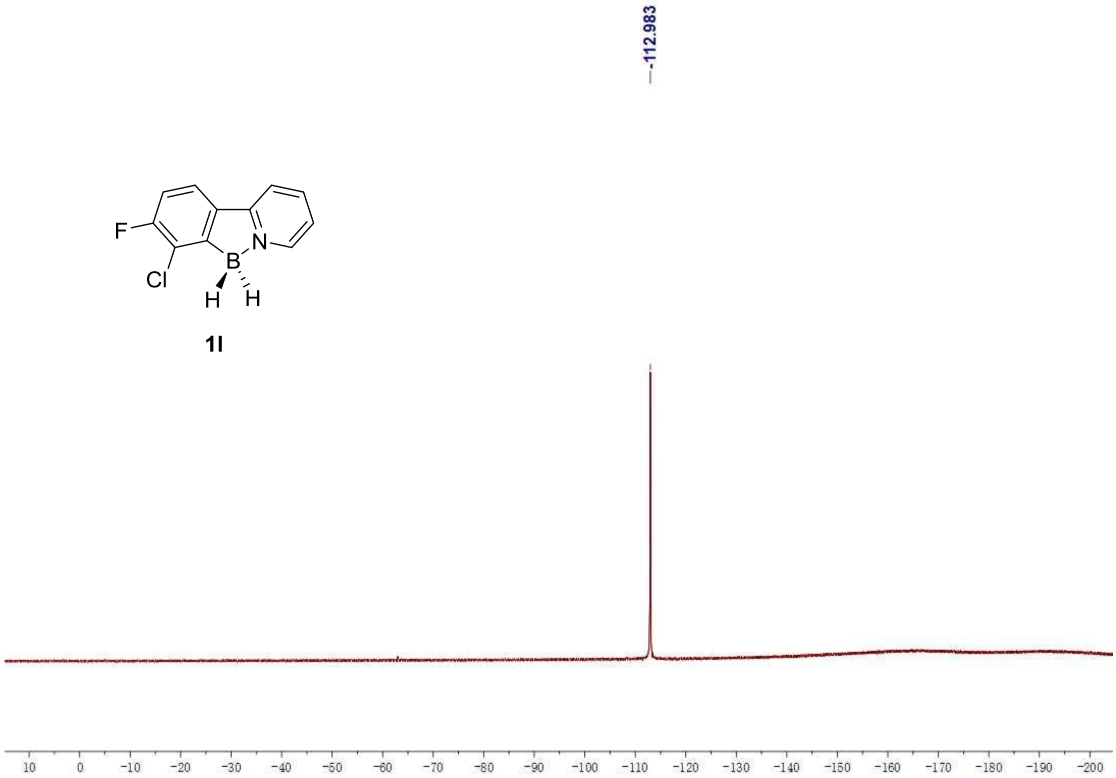

Supplementary Figure 59. ^{19}F NMR spectrum of compound 1l

7,8-dichloro-6H-5 ^{4}-benzo[3,4][1,2]azaborolo[1,5-a]pyridine(1m)

^{1}H NMR (400 MHz, room temperature, $CDCl_3$)

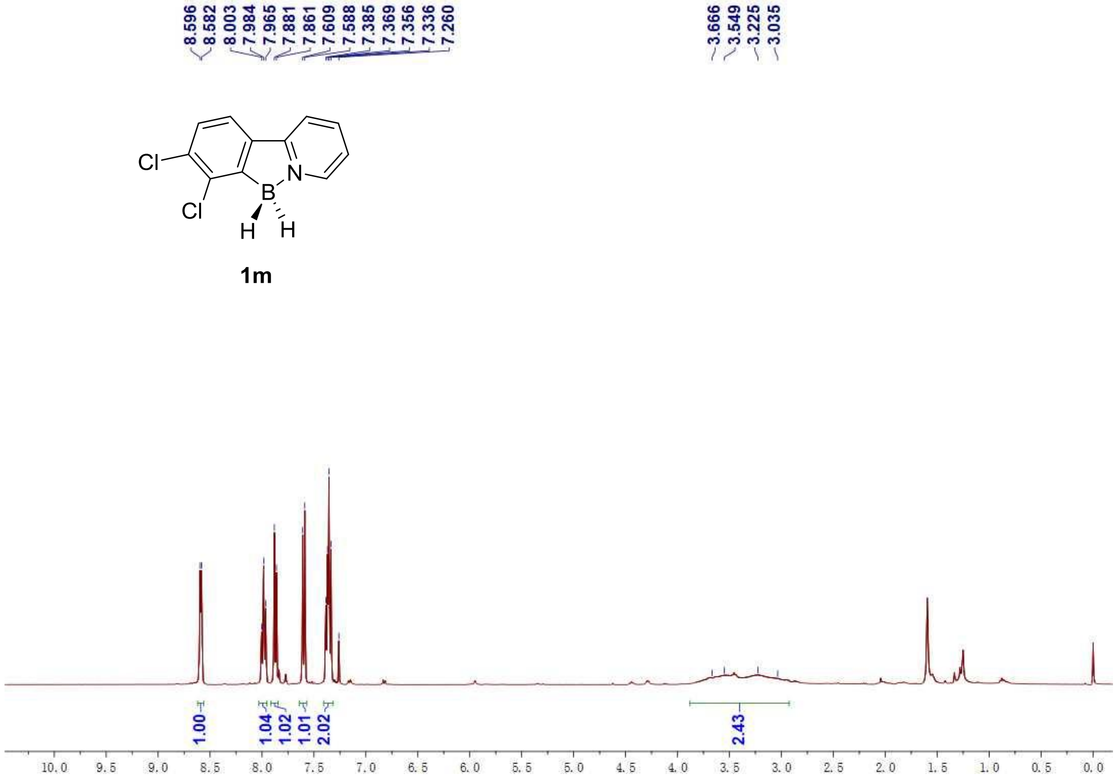

Supplementary Figure 60. ^{1}H NMR spectrum of compound 1m

^{13}C NMR (101 MHz, room temperature, $CDCl_3$)

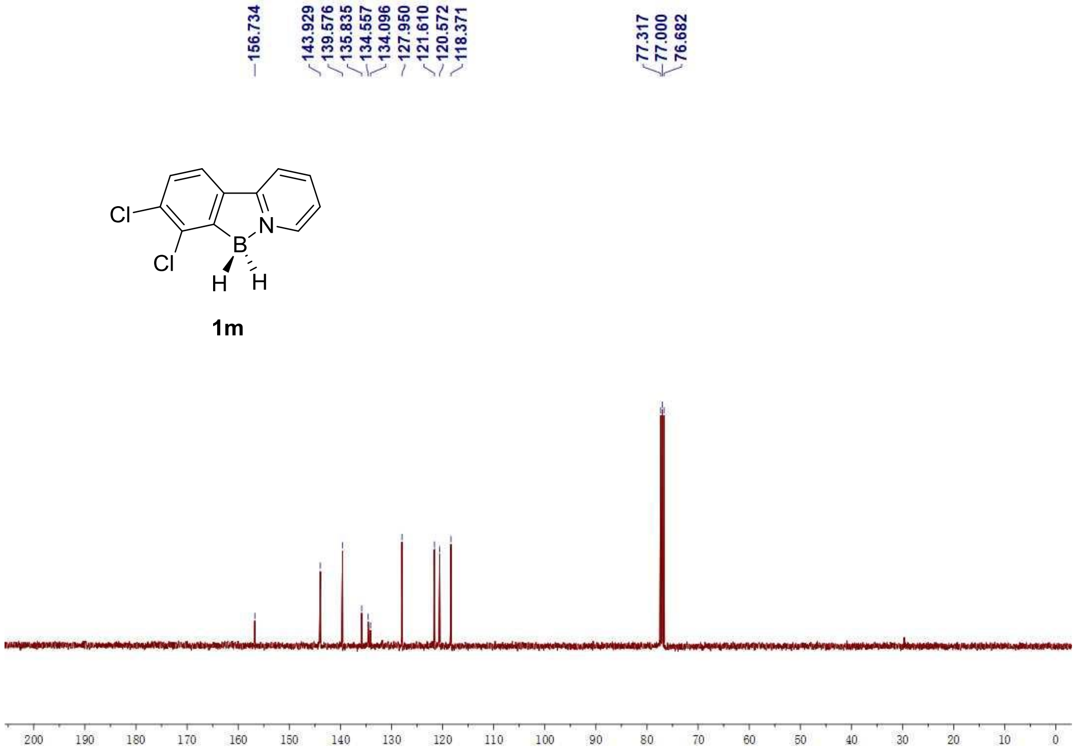

Supplementary Figure 61. ^{13}C NMR spectrum of compound 1m

^{11}B NMR (128 MHz, room temperature, $CDCl_3$)

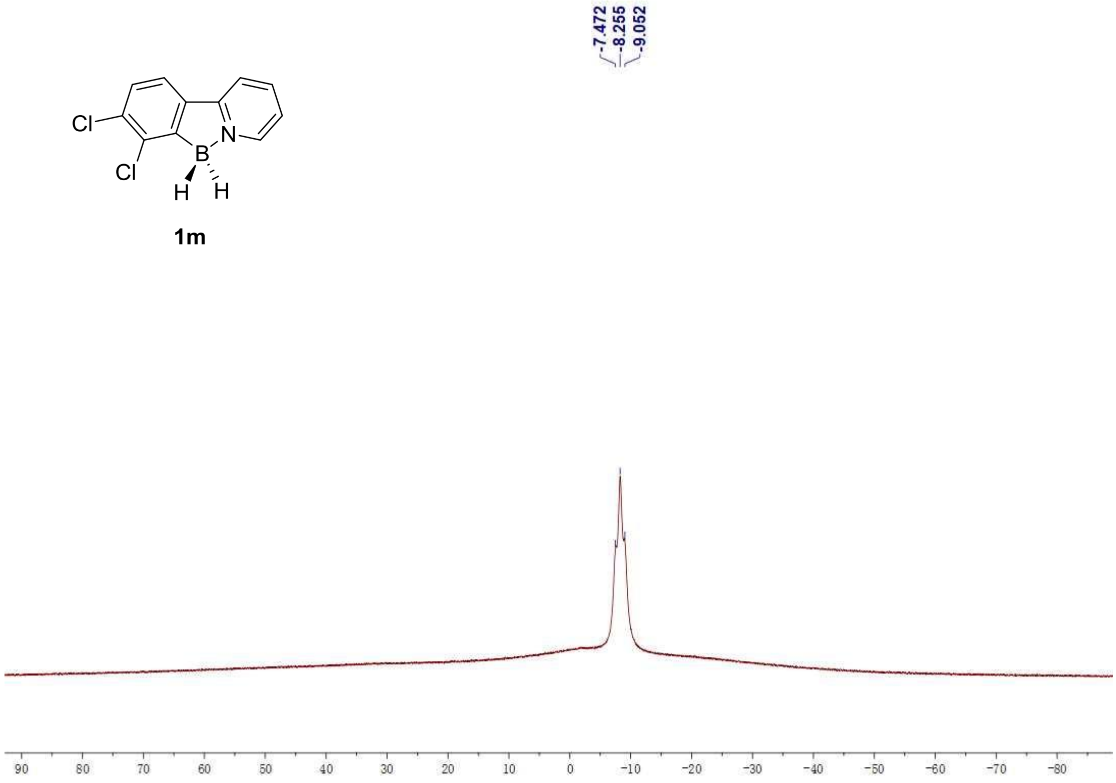

Supplementary Figure 62. ^{11}B NMR spectrum of compound 1m

7,9-dichloro-6H-5 ^{4}-benzo[3,4][1,2]azaborolo[1,5-a]pyridine (1n)

^{1}H NMR (500 MHz, room temperature, $CDCl_3$)

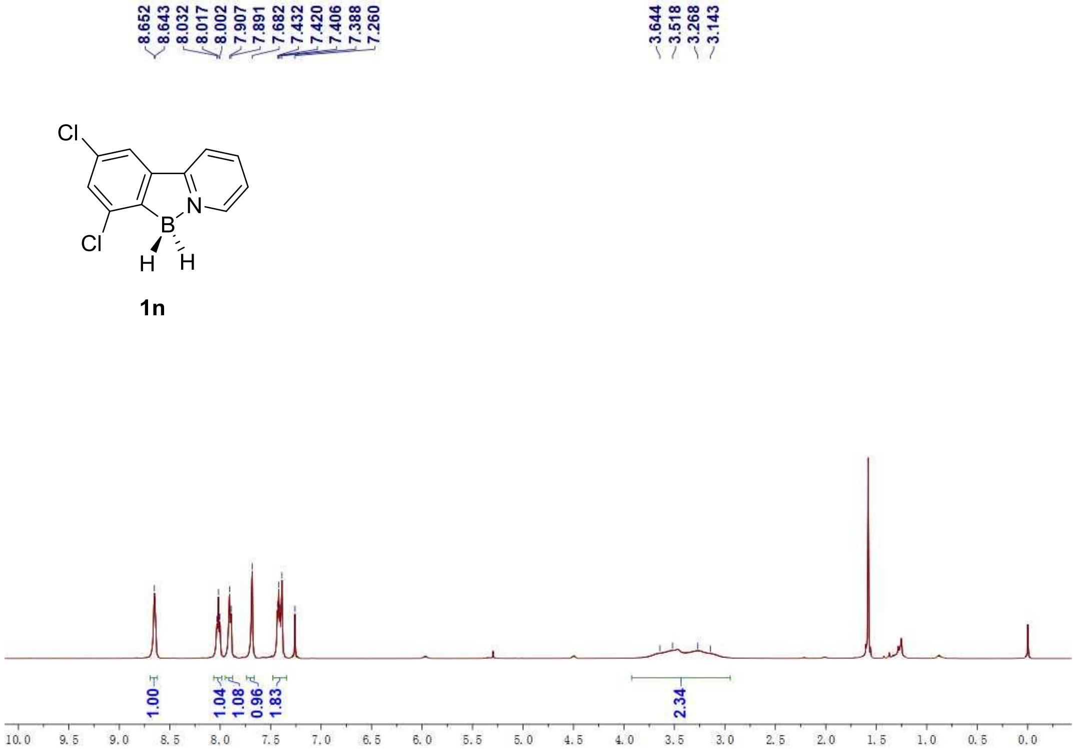

Supplementary Figure 63. ^{1}H NMR spectrum of compound 1n

^{13}C NMR (126 MHz, room temperature, $CDCl_3$)

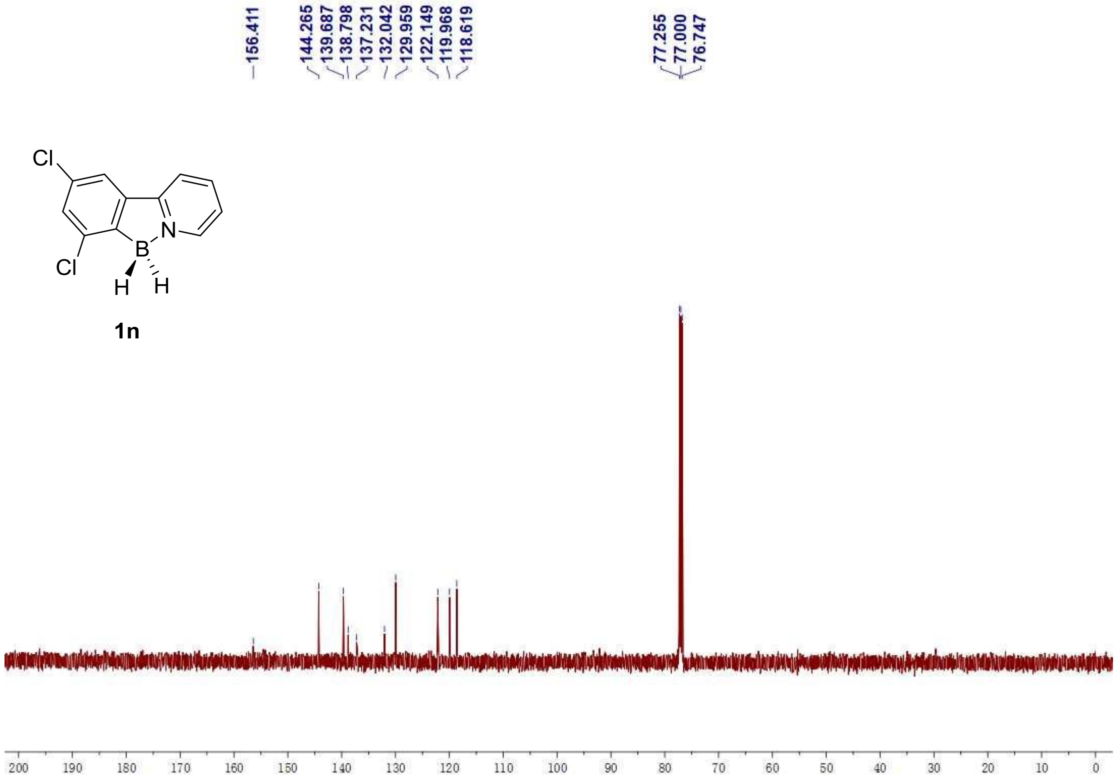

Supplementary Figure 64. ^{13}C NMR spectrum of compound 1n

^{11}B NMR (160 MHz, room temperature, $CDCl_3$)

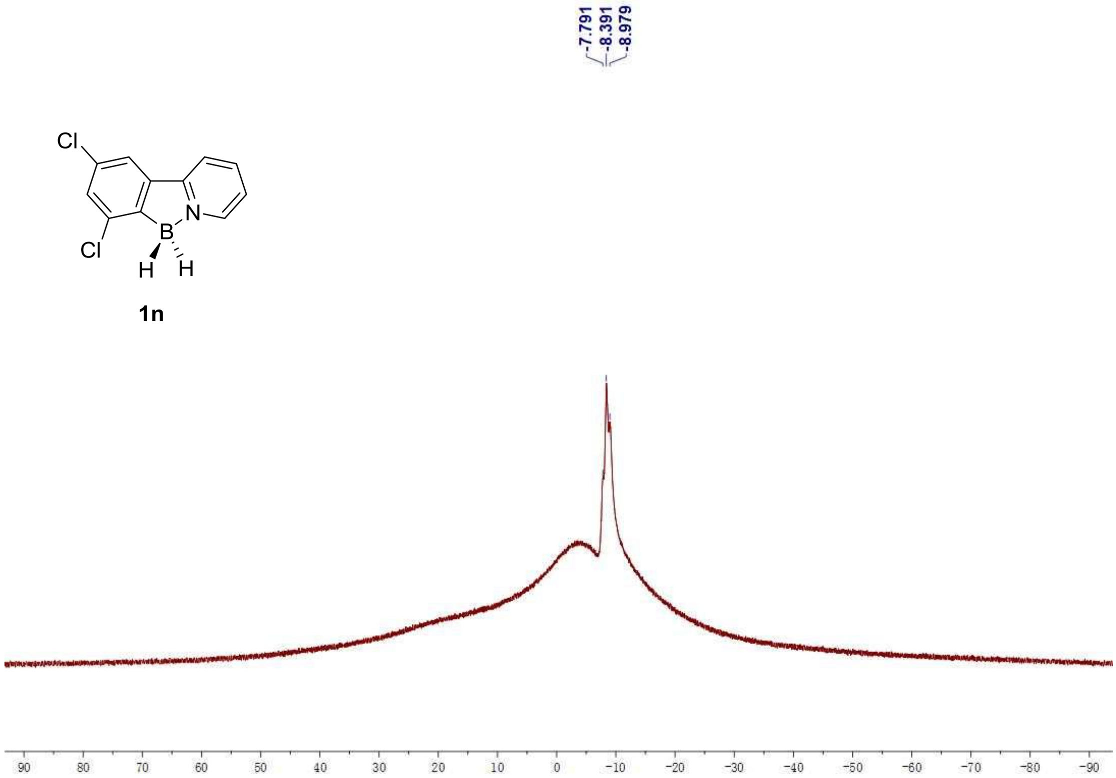

Supplementary Figure 65. ^{11}B NMR spectrum of compound 1n

7-chloro-10-methyl-6H-5 ^{4}-benzo[3,4][1,2]azaborolo[1,5-a]pyridine(1o)

^{1}H NMR (400 MHz, room temperature, $CDCl_3$)

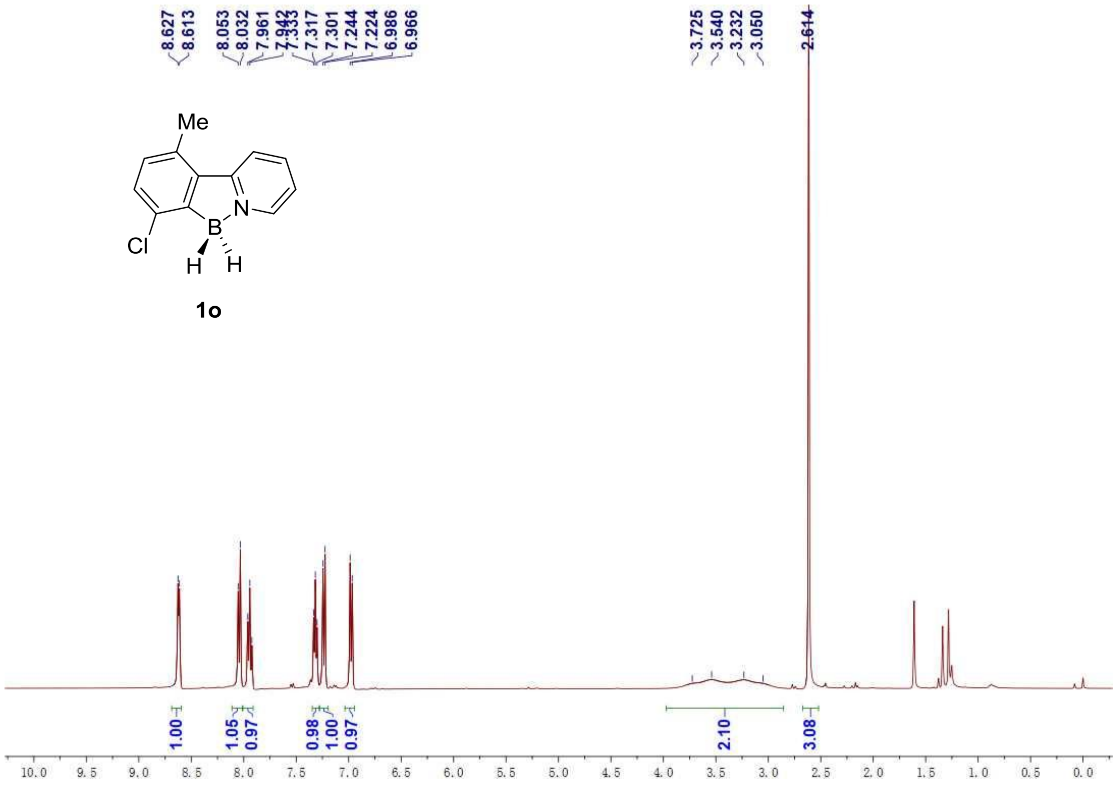

Supplementary Figure 66. ^{1}H NMR spectrum of compound 1o

^{13}C NMR (101 MHz, room temperature, $CDCl_3$)

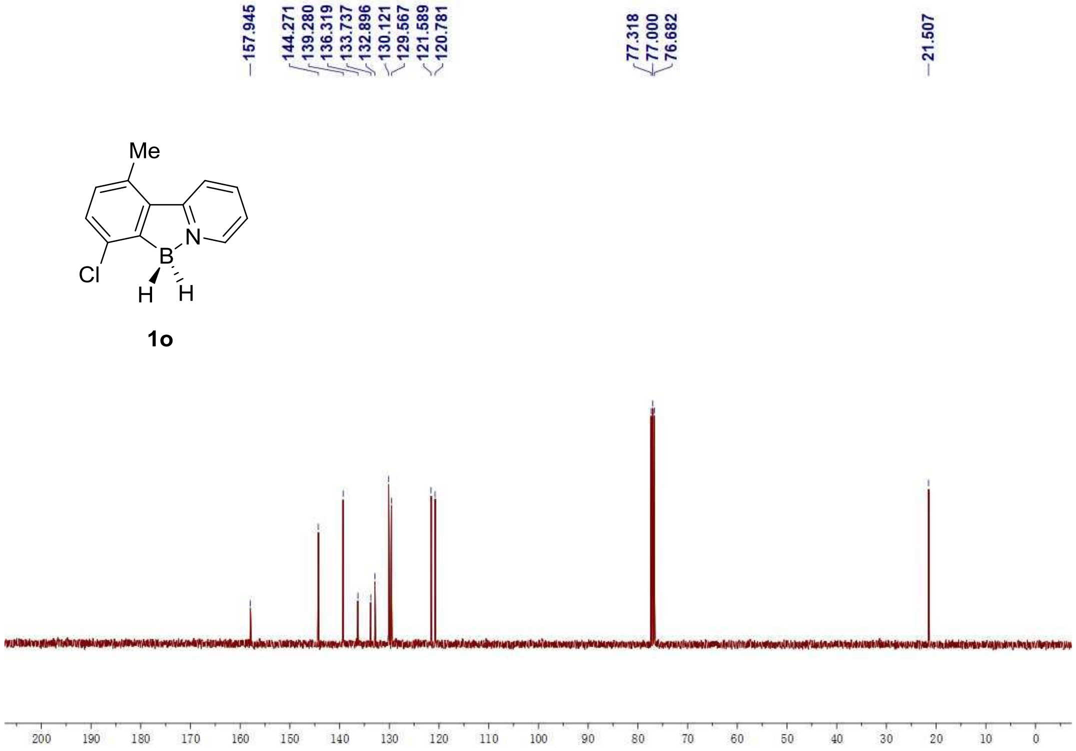

Supplementary Figure 67. ^{13}C NMR spectrum of compound 1o

^{11}B NMR (128 MHz, room temperature, $CDCl_3$)

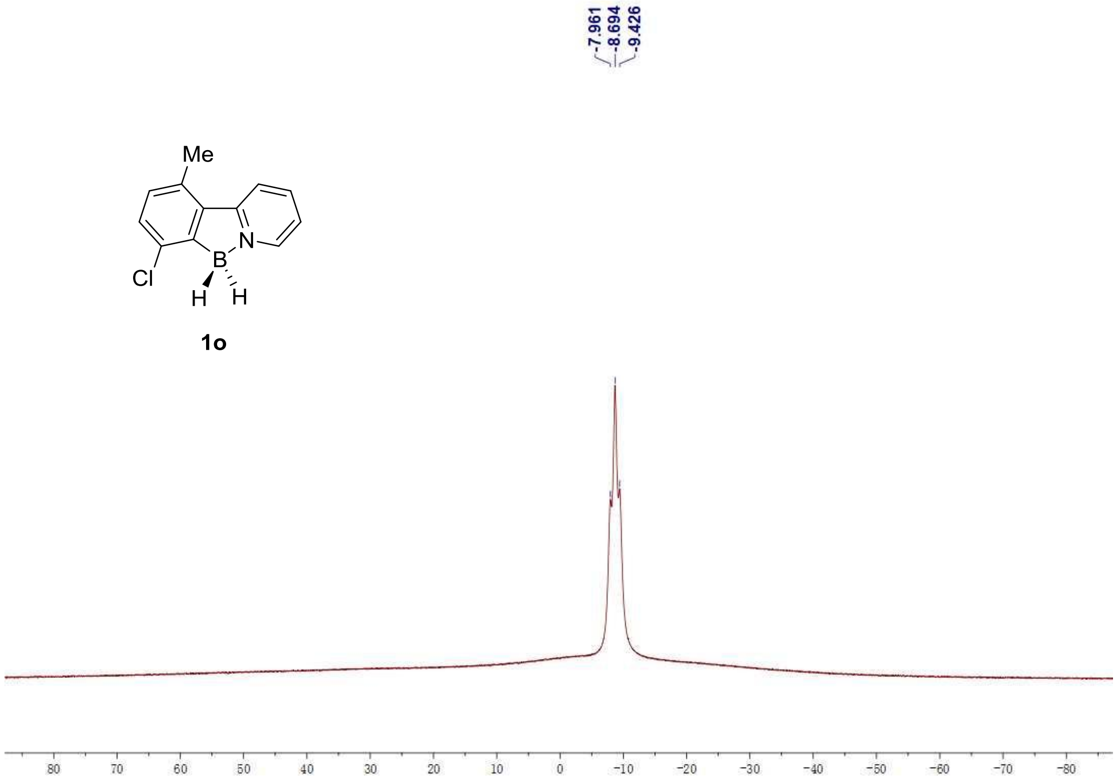

Supplementary Figure 68. ^{11}B NMR spectrum of compound 1o

12H-11 ^{4}-naphtho[1',2':3,4][1,2]azaborolo[1,5-a]pyridine (1p)

^{1}H NMR (500 MHz, room temperature, $CDCl_3$)

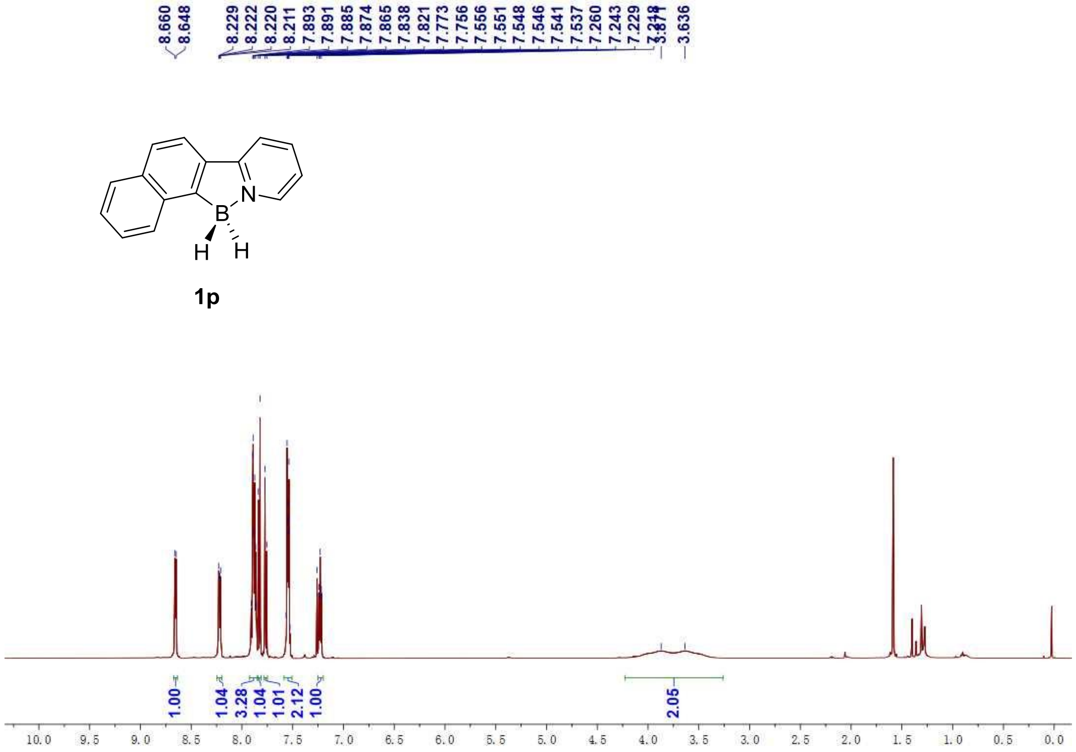

Supplementary Figure 69. ^{1}H NMR spectrum of compound 1p

^{13}C NMR (126 MHz, room temperature, $CDCl_3$)

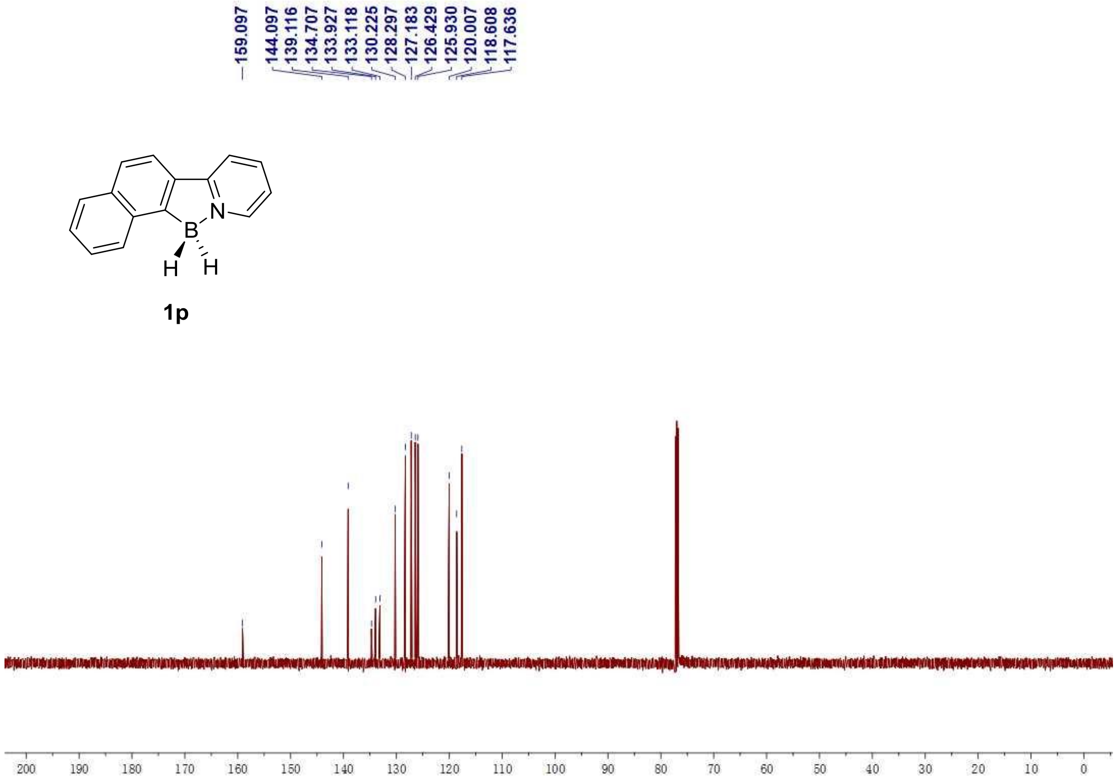

Supplementary Figure 70. ^{13}**C NMR spectrum of compound 1p**

^{11}B NMR (160 MHz, room temperature, $CDCl_3$)

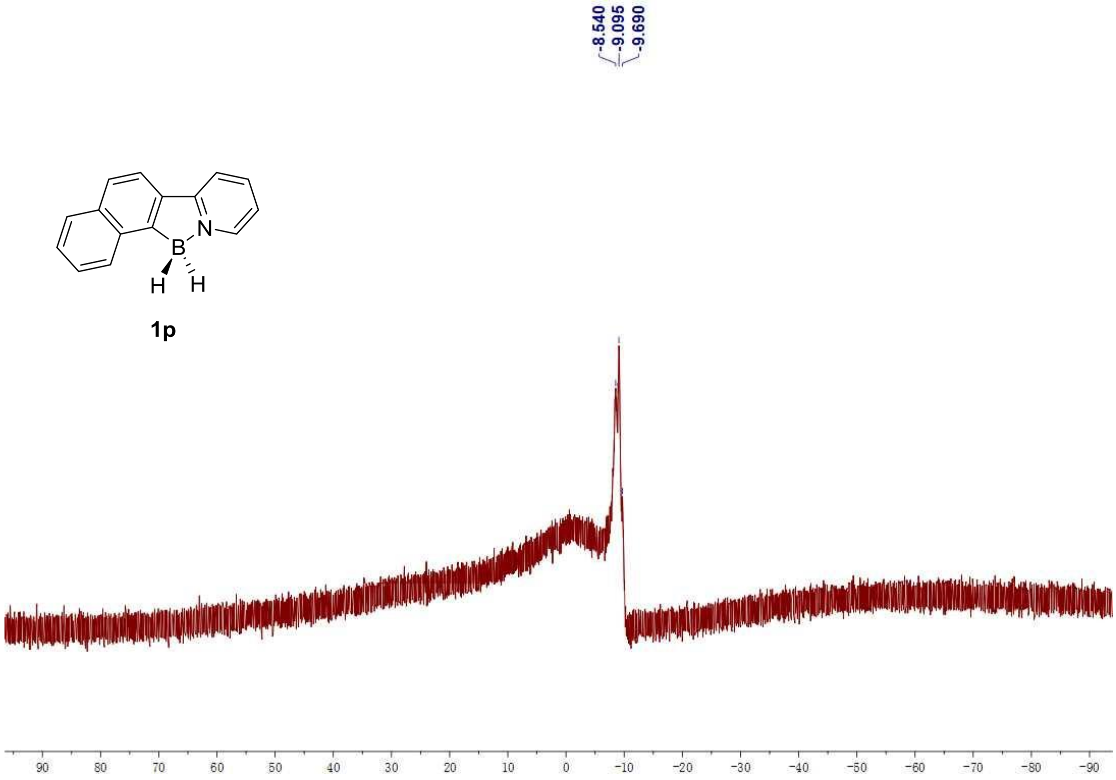

Supplementary Figure 71. ^{11}**B NMR spectrum of compound 1p**

8-fluoro-6H-5 ^{4}-benzo[3,4][1,2]azaborolo[1,5-a]pyridine(1q)

^{1}H NMR (500 MHz, room temperature, $CDCl_3$)

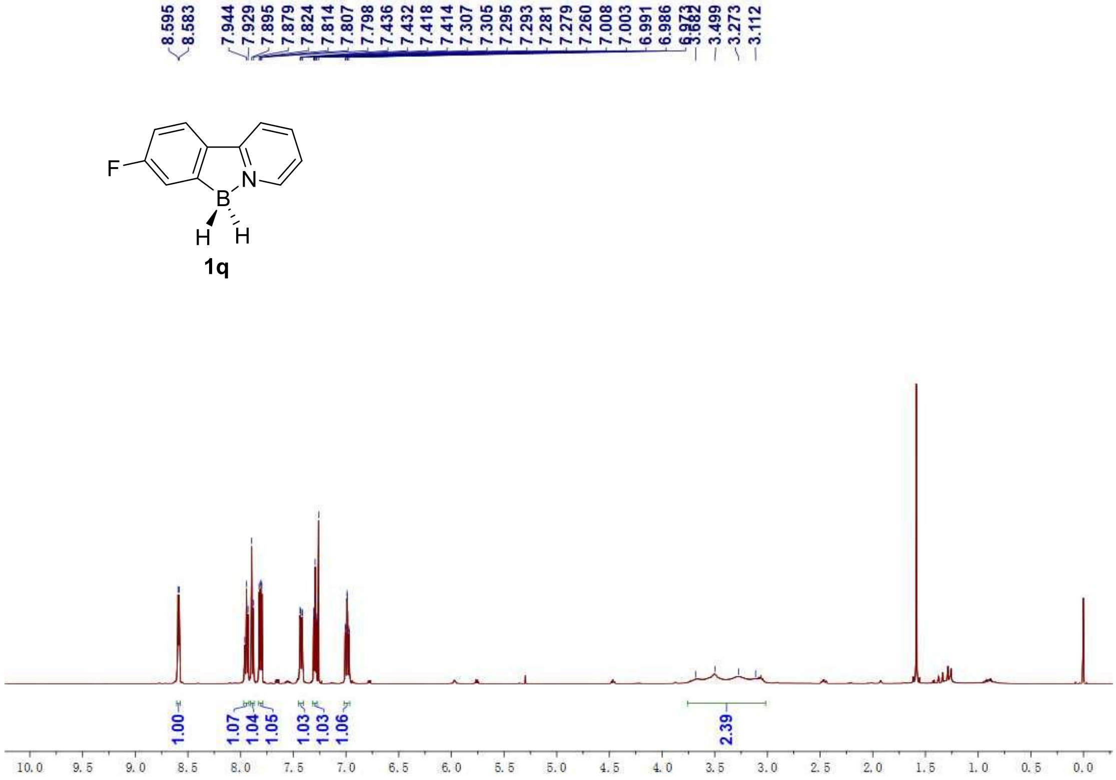

Supplementary Figure 72. ^{1}H NMR spectrum of compound 1q

^{13}C NMR (126 MHz, room temperature, $CDCl_3$)

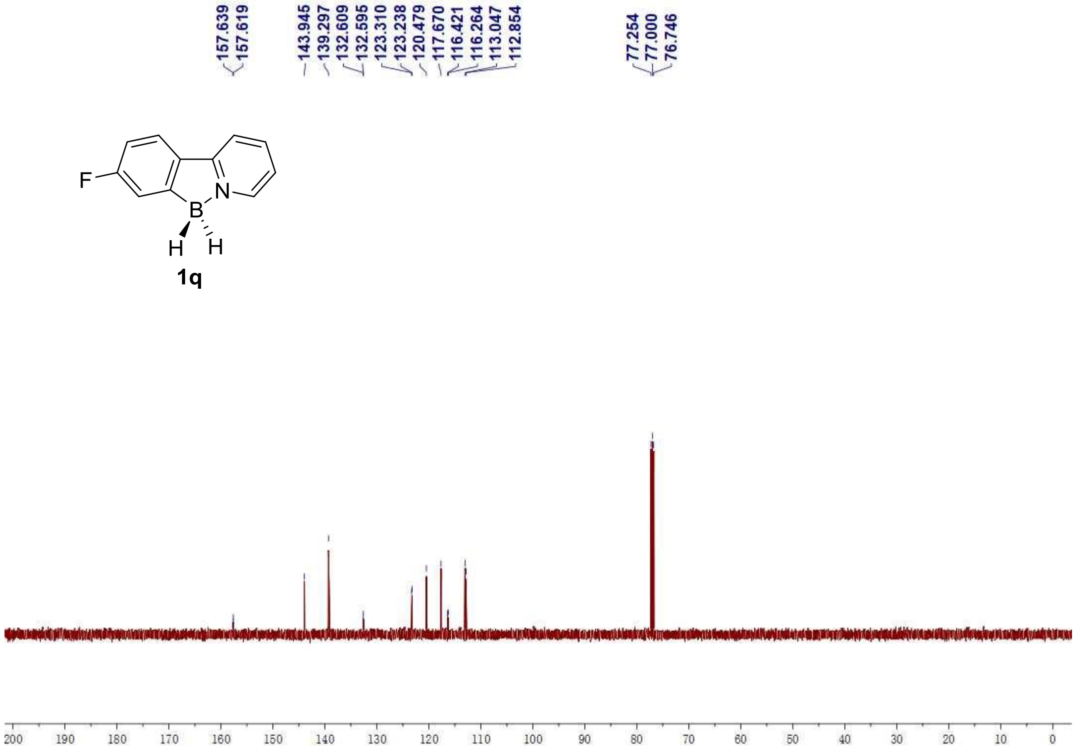

Supplementary Figure 73. ^{13}C NMR spectrum of compound 1q

^{11}**B NMR (160 MHz, room temperature**, **CDCl$_3$**)

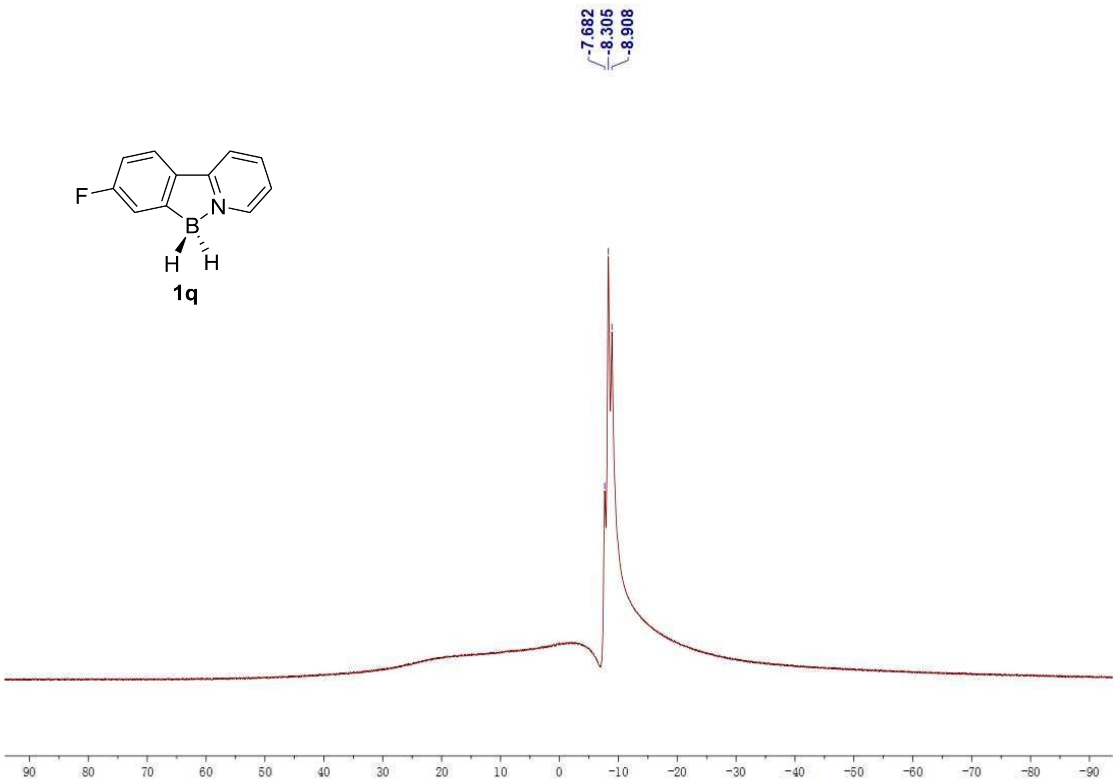

Supplementary Figure 74. ^{11}**B NMR spectrum of compound 1q**

^{19}**F NMR (376 MHz, room temperature**, **CDCl$_3$**)

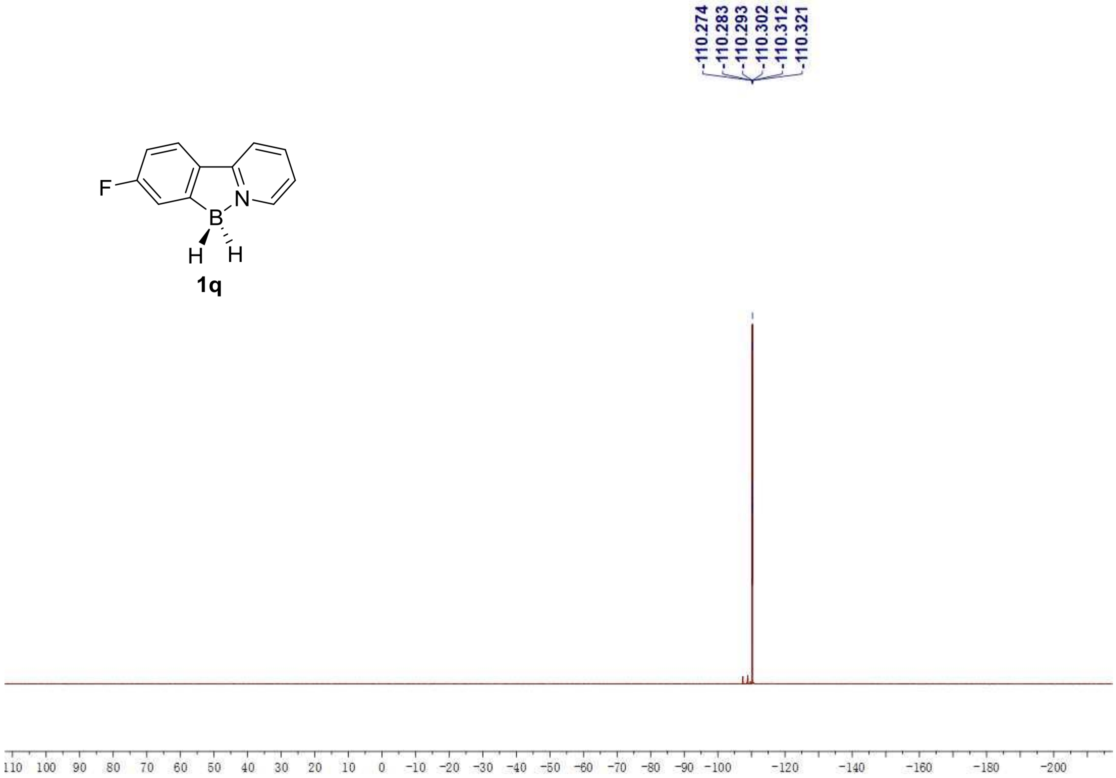

Supplementary Figure 75. ^{19}**F NMR spectrum of compound 1q**

7-chloro-8-methyl-6H-5 ^{4}-benzo[3,4][1,2]azaborolo[1,5-a]pyridine(1r)

^{1}H NMR (400 MHz, room temperature, $CDCl_3$)

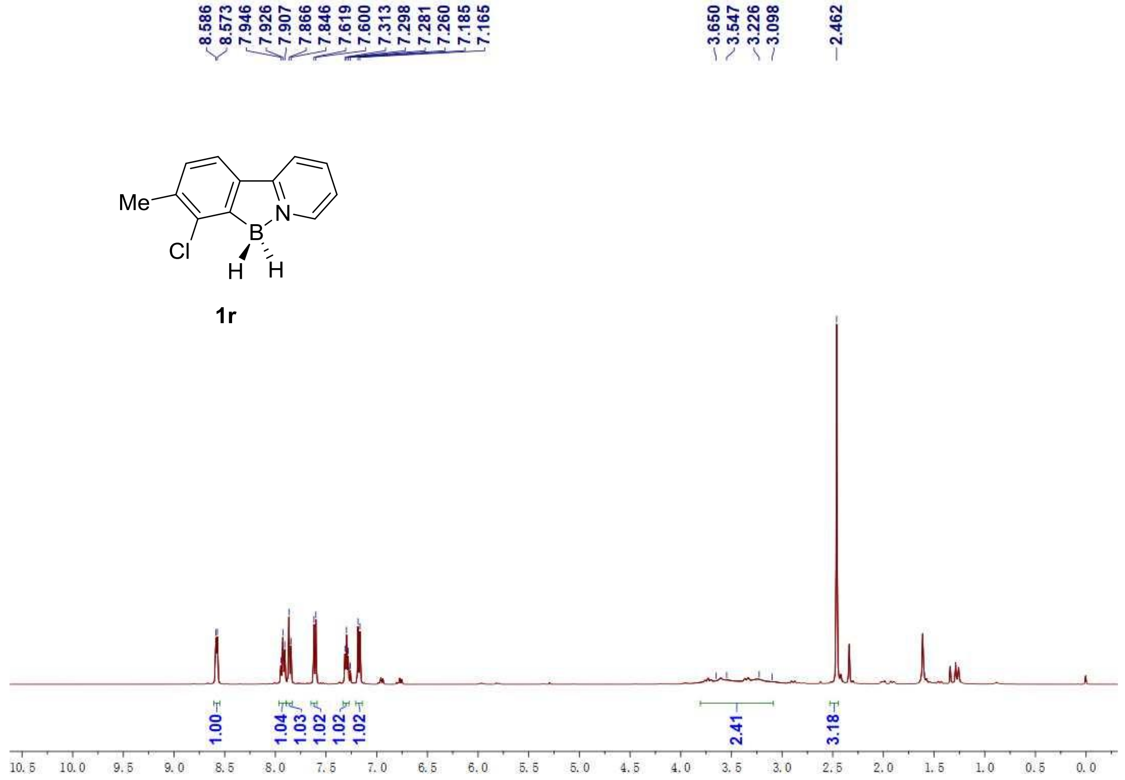

Supplementary Figure 76. ^{1}H NMR spectrum of compound 1r

^{13}C NMR (101 MHz, room temperature, $CDCl_3$)

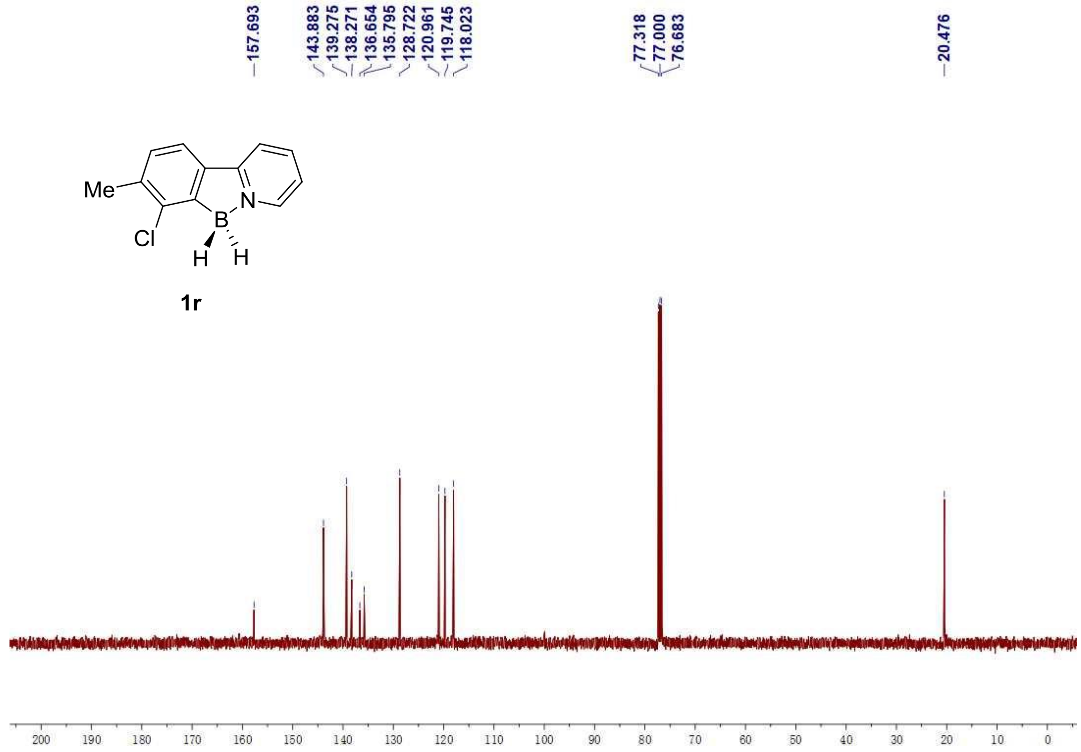

Supplementary Figure 77. ^{13}C NMR spectrum of compound 1r

^{11}B NMR (128 MHz, room temperature, $CDCl_3$)

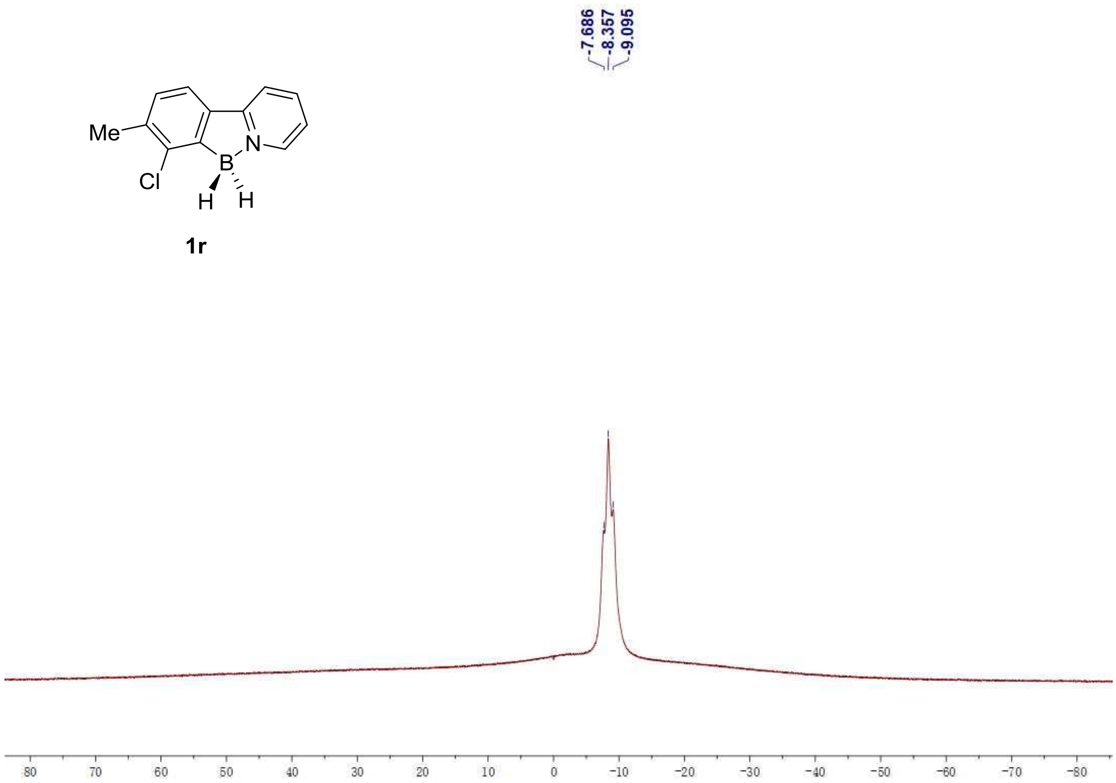

Supplementary Figure 78. ^{11}B NMR spectrum of compound 1r

7,10-dichloro-6H-5 ^{4}-benzo[3,4][1,2]azaborolo[1,5-a]pyridine(1s)

^{1}H NMR (400 MHz, room temperature, $CDCl_3$)

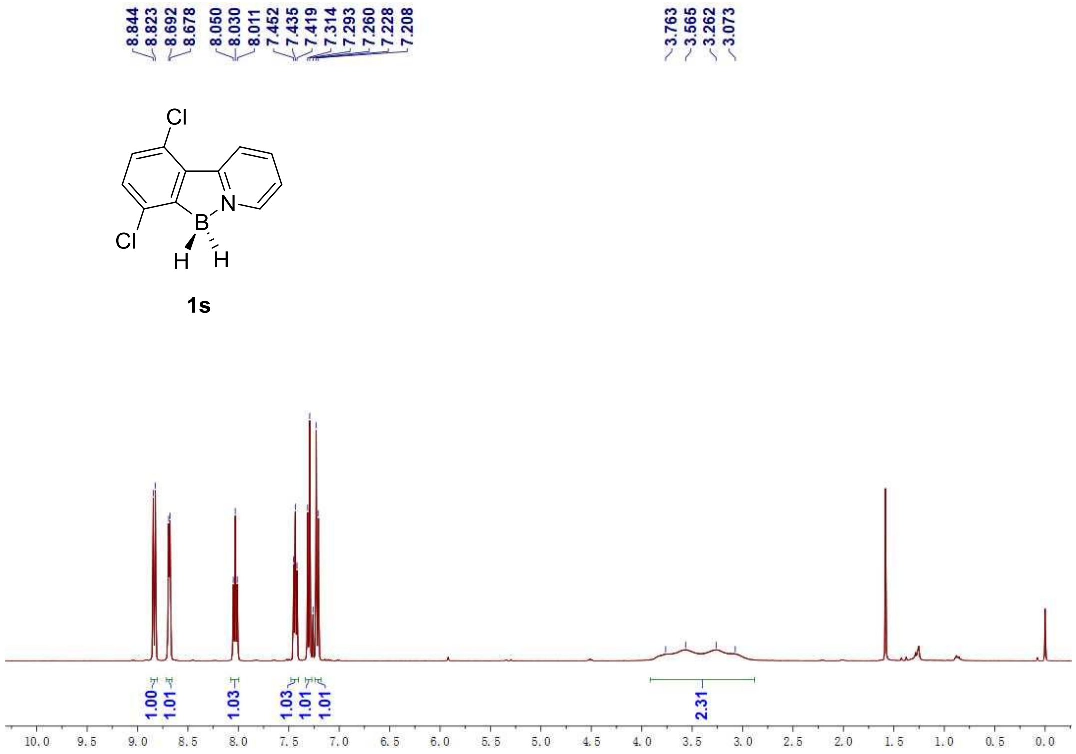

Supplementary Figure 79. ^{1}H NMR spectrum of compound 1s

^{13}C NMR (101 MHz, room temperature, $CDCl_3$)

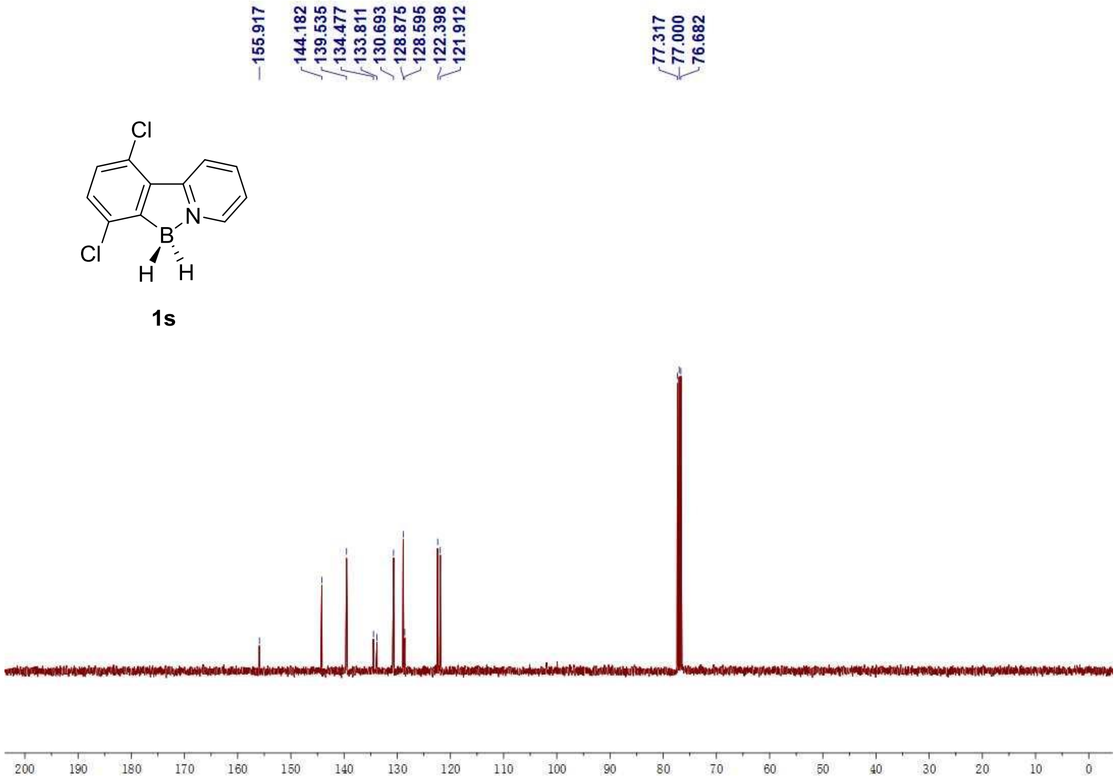

Supplementary Figure 80. ^{13}C NMR spectrum of compound 1s

^{11}B NMR (128 MHz, room temperature, $CDCl_3$)

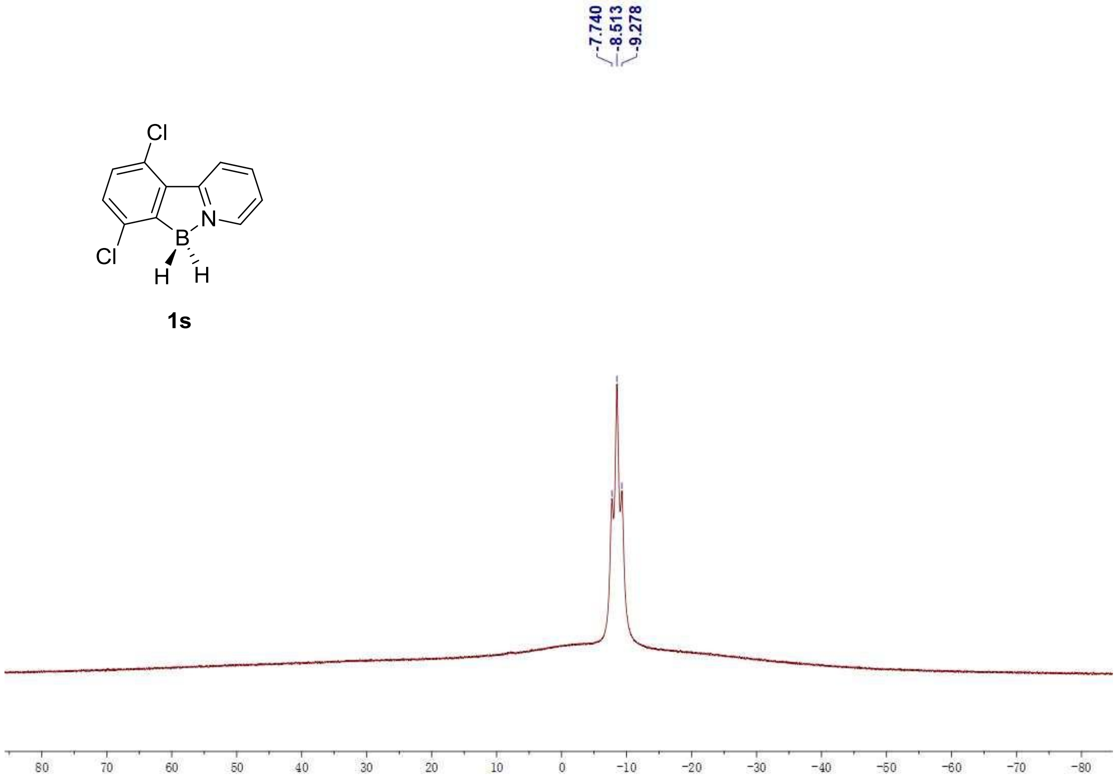

Supplementary Figure 81. ^{11}B NMR spectrum of compound 1s

7-(trifluoromethoxy)-6H-5 ^{4}-benzo[3,4][1,2]azaborolo[1,5-a]pyridine (1t)

^{1}H NMR (500 MHz, room temperature, $CDCl_3$)

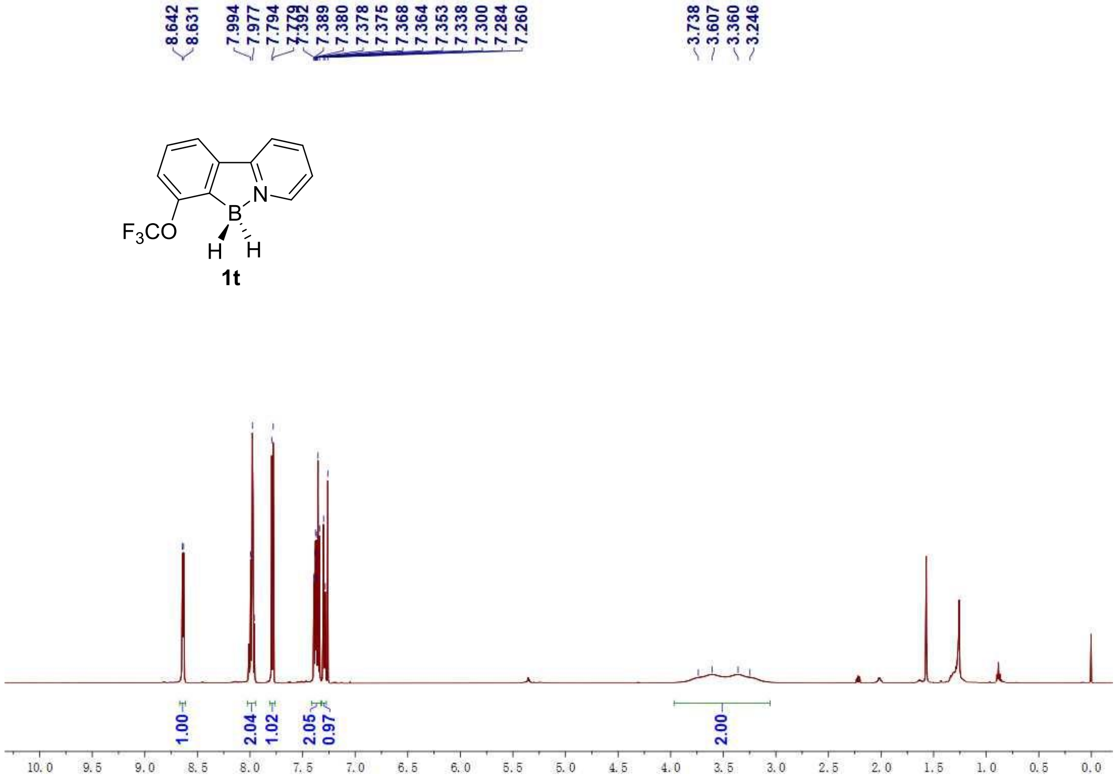

Supplementary Figure 82. ^{1}H NMR spectrum of compound 1t

^{13}C NMR (126 MHz, room temperature, $CDCl_3$)

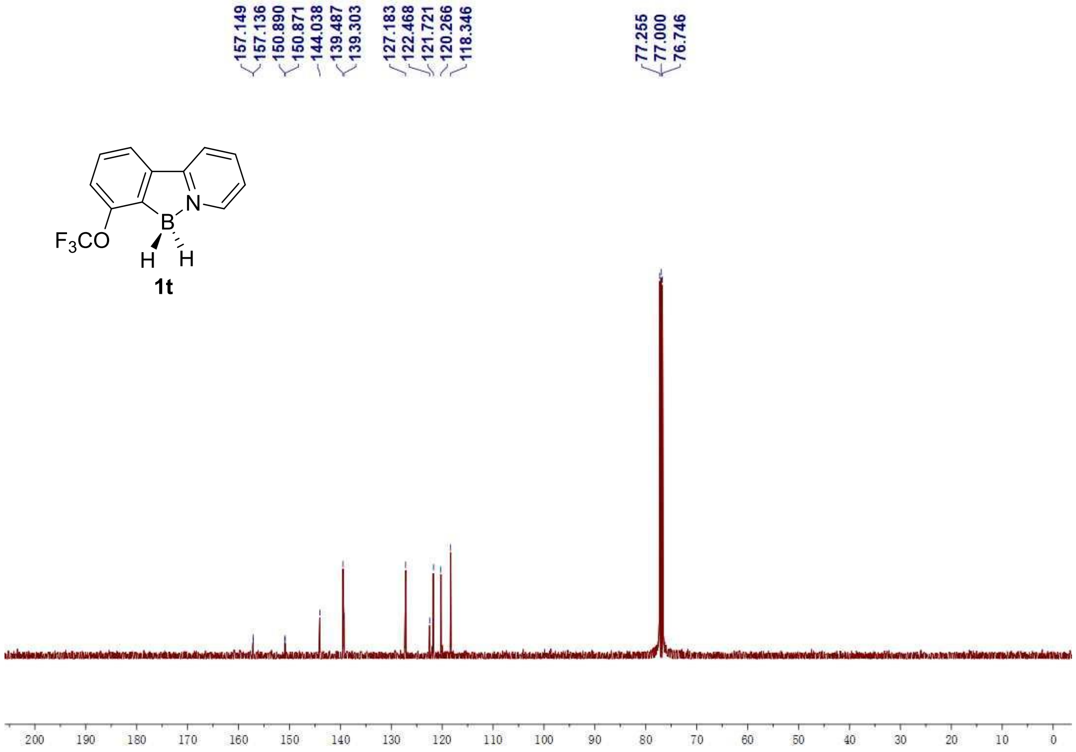

Supplementary Figure 83. ^{13}C NMR spectrum of compound 1t

^{11}B NMR (128 MHz, room temperature, $CDCl_3$)

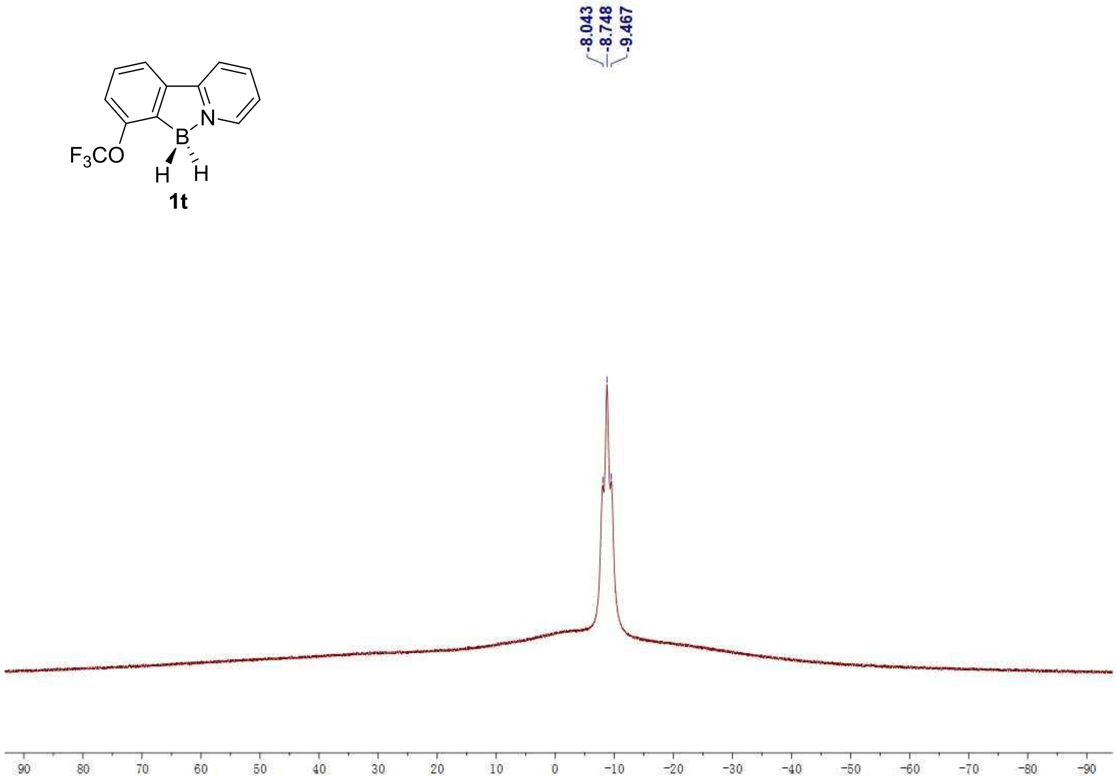

Supplementary Figure 84. ^{11}B NMR spectrum of compound 1t

^{19}F NMR (376 MHz, room temperature, $CDCl_3$)

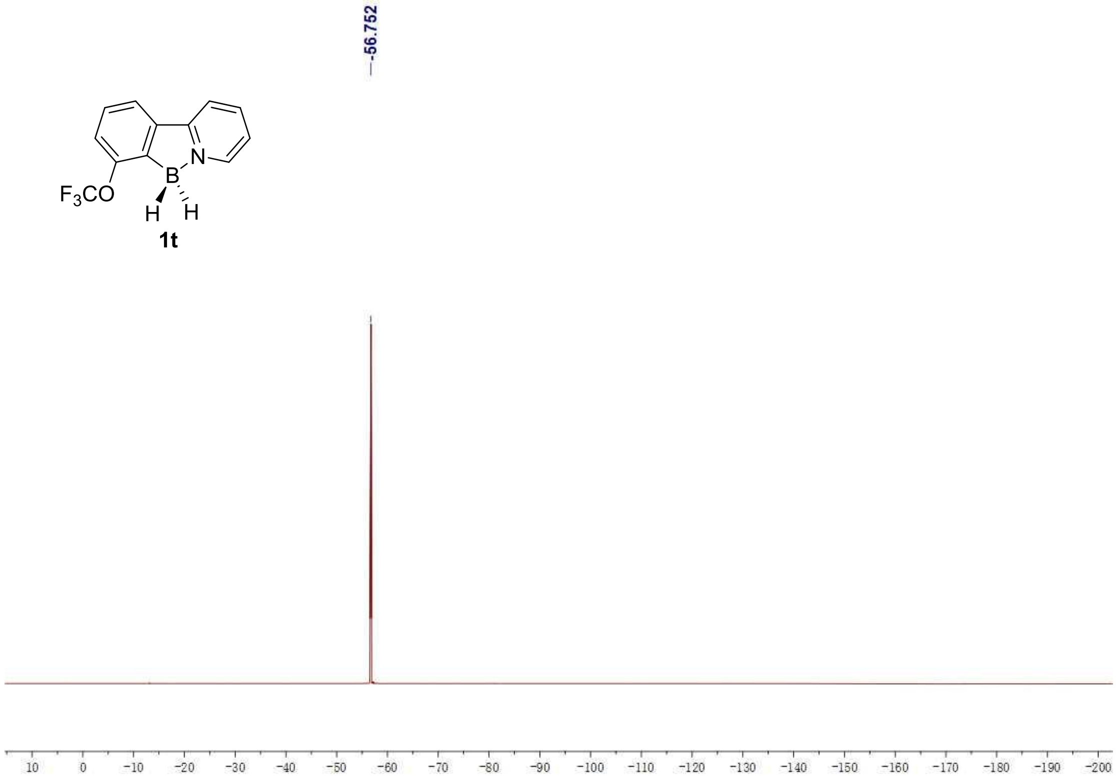

Supplementary Figure 85. ^{19}F NMR spectrum of compound 1t

7,9-dimethyl-6H-5 ^{4}-benzo[3,4][1,2]azaborolo[1,5-a]pyridine (1u)

^{1}H NMR (500 MHz, room temperature, $CDCl_3$)

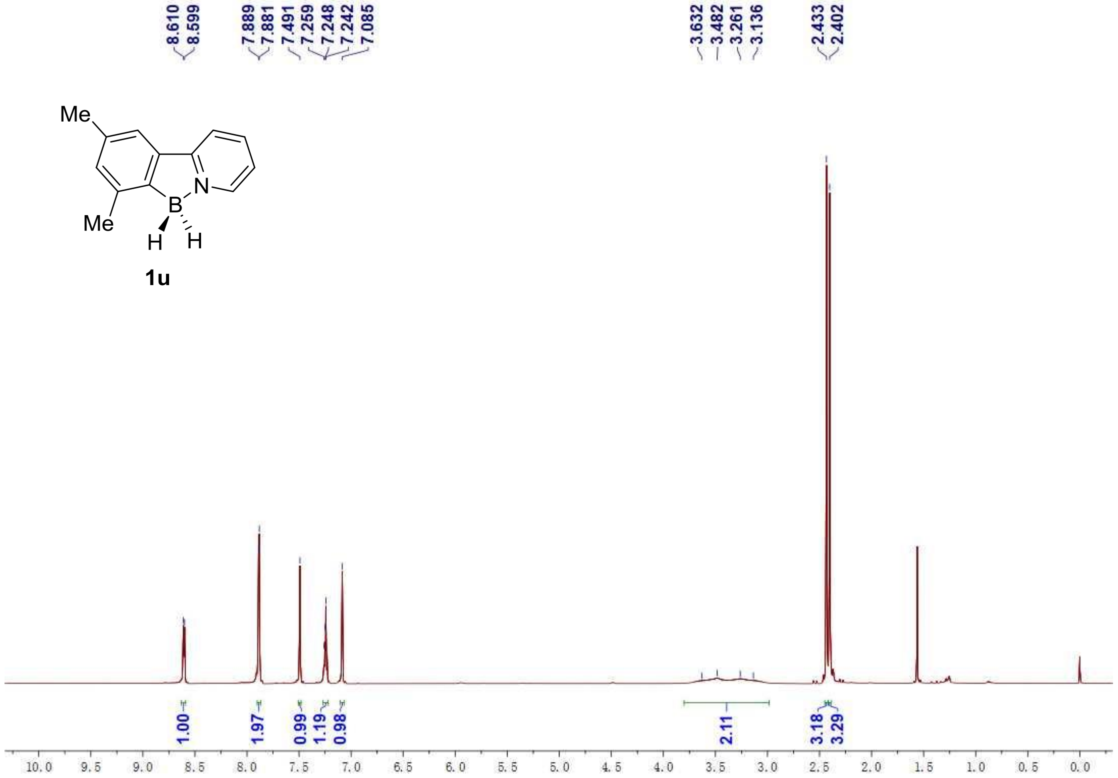

Supplementary Figure 86. ^{1}H NMR spectrum of compound 1u

^{13}C NMR (126 MHz, room temperature, $CDCl_3$)

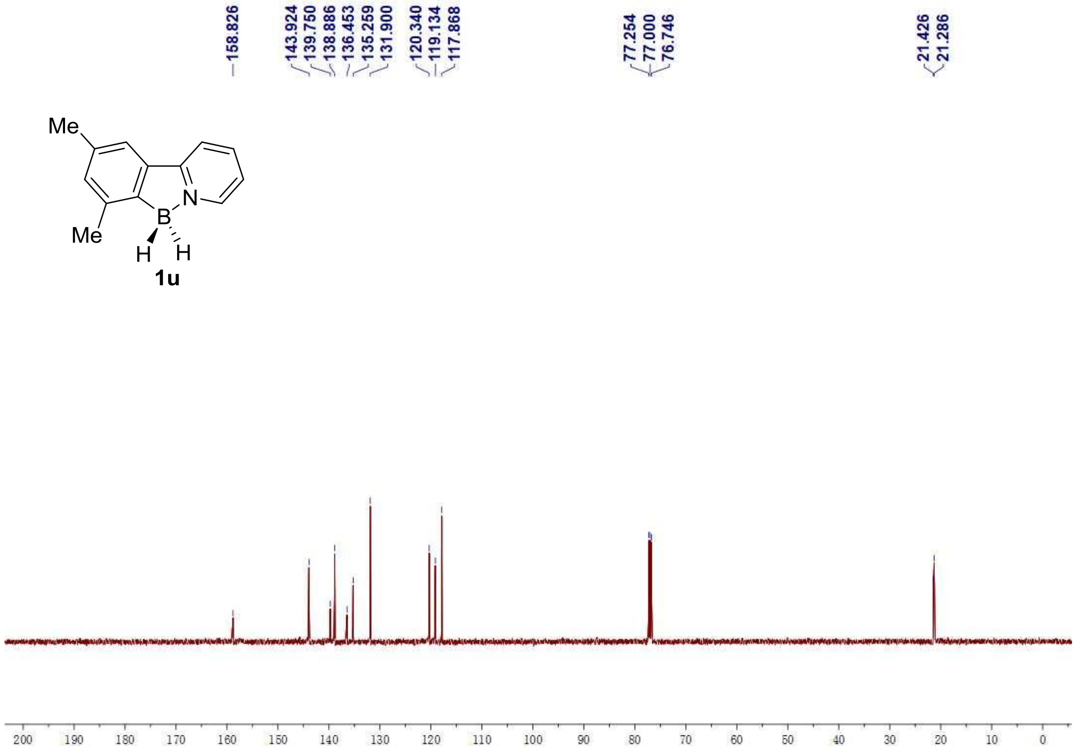

Supplementary Figure 87. ^{13}C NMR spectrum of compound 1u

^{11}B NMR (160 MHz, room temperature, $CDCl_3$)

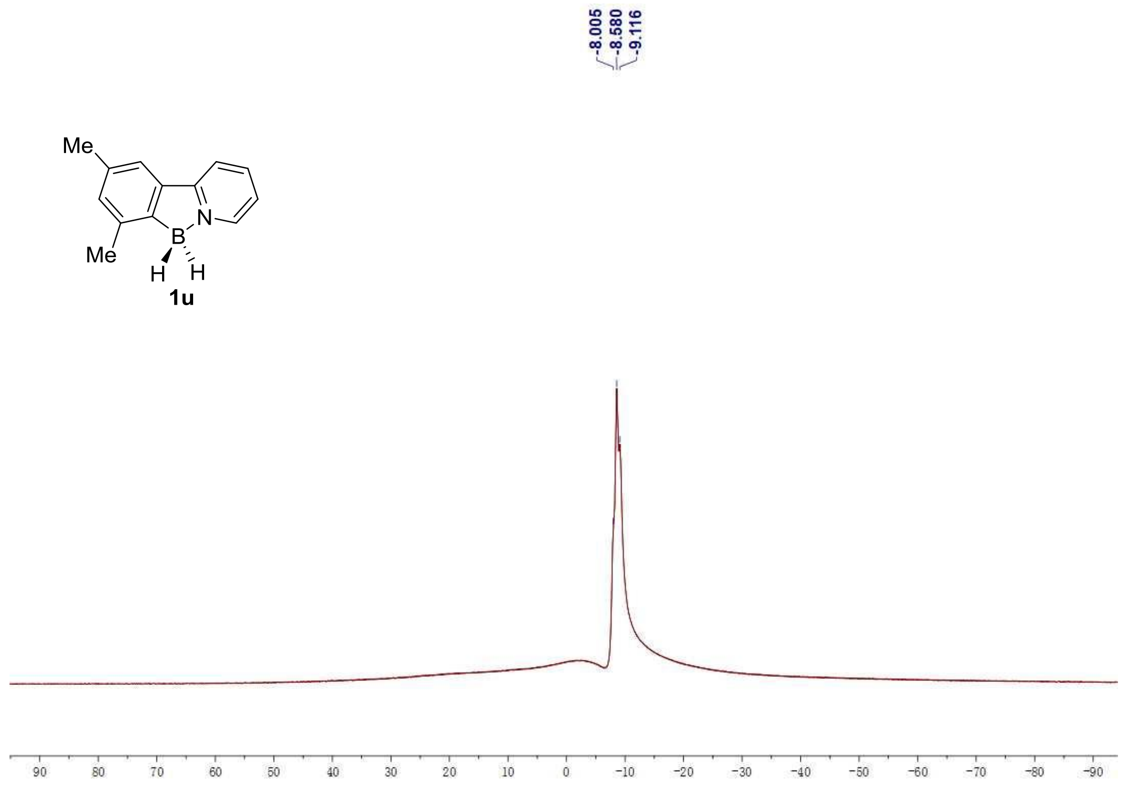

Supplementary Figure 88. ^{11}B NMR spectrum of compound 1u

9H-8 ^{4}-thieno[2',3':3,4][1,2]azaborolo[1,5-a]pyridine(1v)

^{1}H NMR (500 MHz, room temperature, $CDCl_3$)

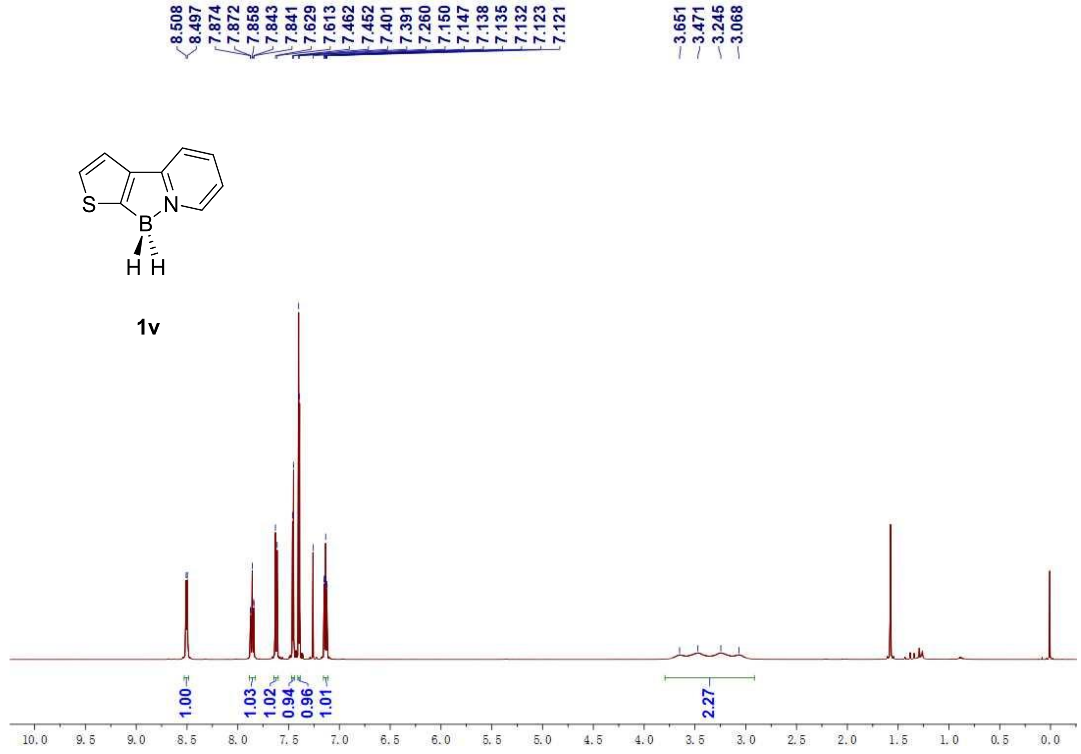

Supplementary Figure 89. ^{1}H NMR spectrum of compound 1v

^{13}C NMR (126 MHz, room temperature, $CDCl_3$)

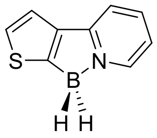

1v

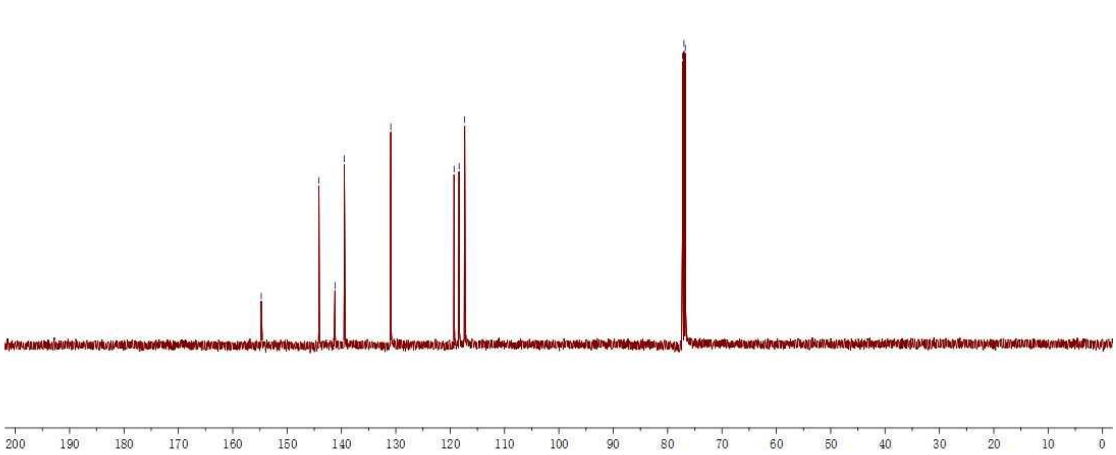

Supplementary Figure 90. ^{13}C NMR spectrum of compound 1v

^{11}B NMR (160 MHz, room temperature, $CDCl_3$)

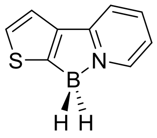

1v

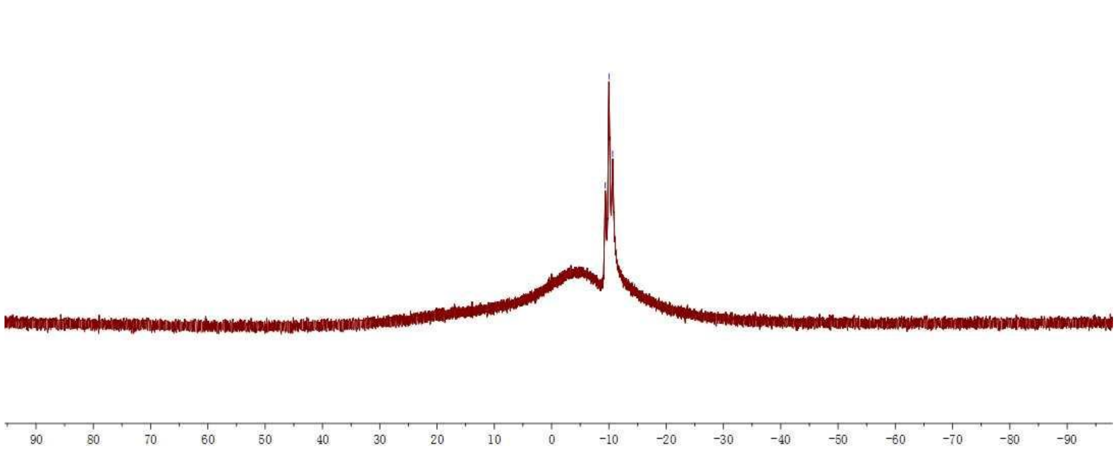

Supplementary Figure 91. ^{11}B NMR spectrum of compound 1v

6H-7 [4]-benzo[4',5']thieno[2',3':3,4][1,2]azaborolo[1,5-a]pyridine(1w)

^{1}H NMR (500 MHz, room temperature, $CDCl_3$)

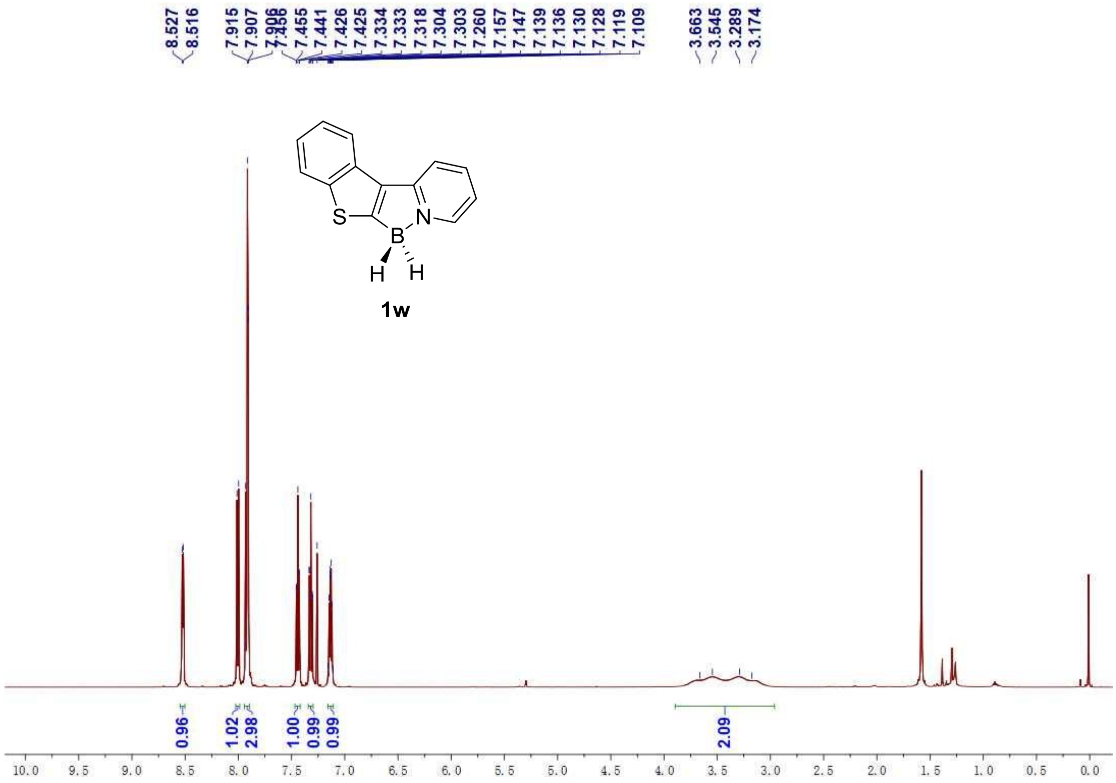

Supplementary Figure 92. ^{1}H NMR spectrum of compound 1w

^{13}C NMR (126 MHz, room temperature, $CDCl_3$)

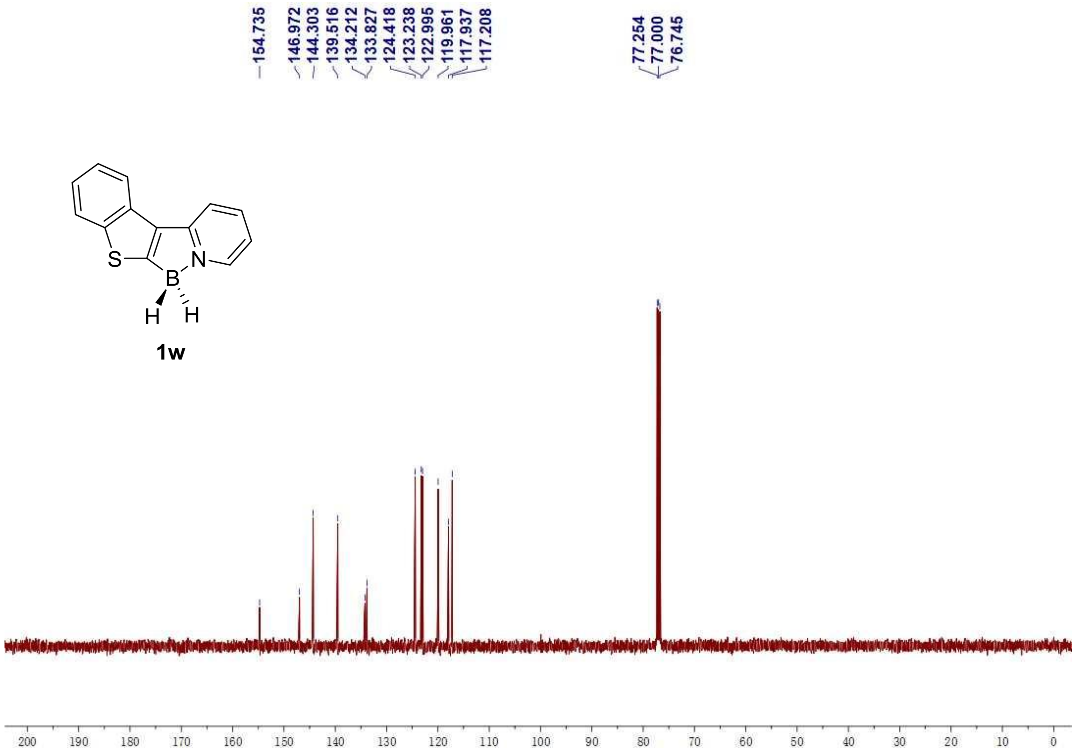

Supplementary Figure 93. ^{13}C NMR spectrum of compound 1w

^{11}B NMR (160 MHz, room temperature, CDCl$_3$)

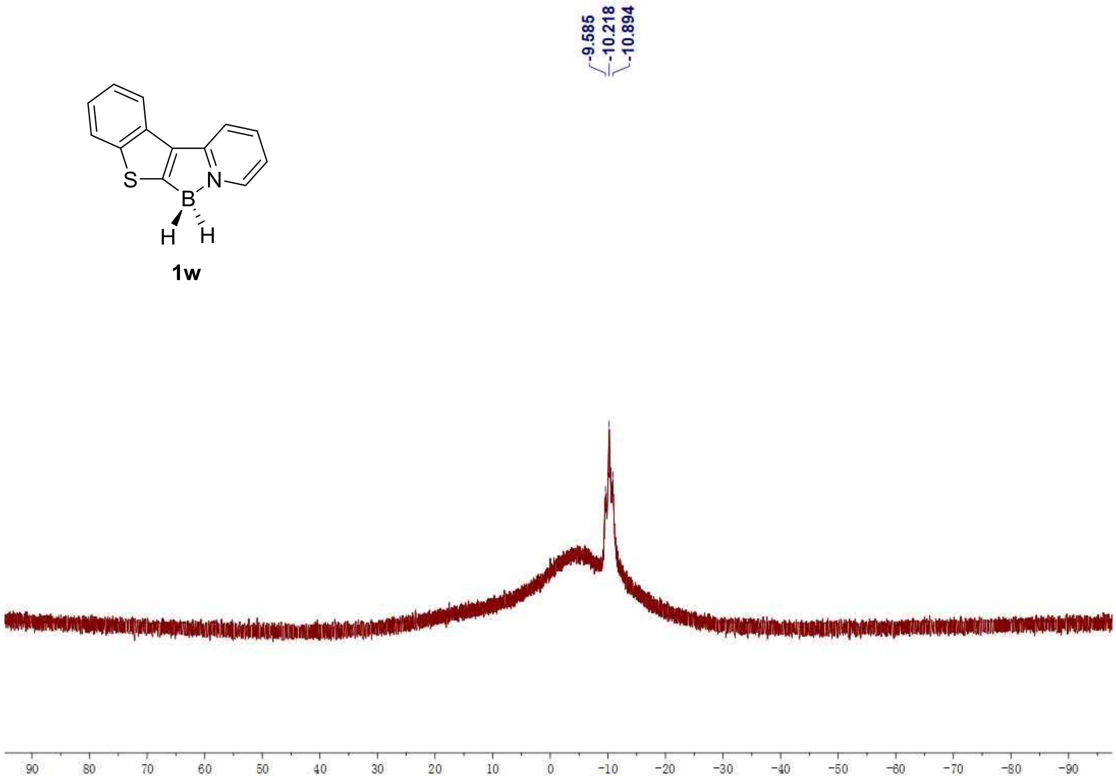

Supplementary Figure 94. ^{11}B NMR spectrum of compound 1w

1-fluoro-6H-5 ^{4}-benzo[3,4][1,2]azaborolo[1,5-a]pyridine (1x)

^{1}H NMR (400 MHz, room temperature, CDCl$_3$)

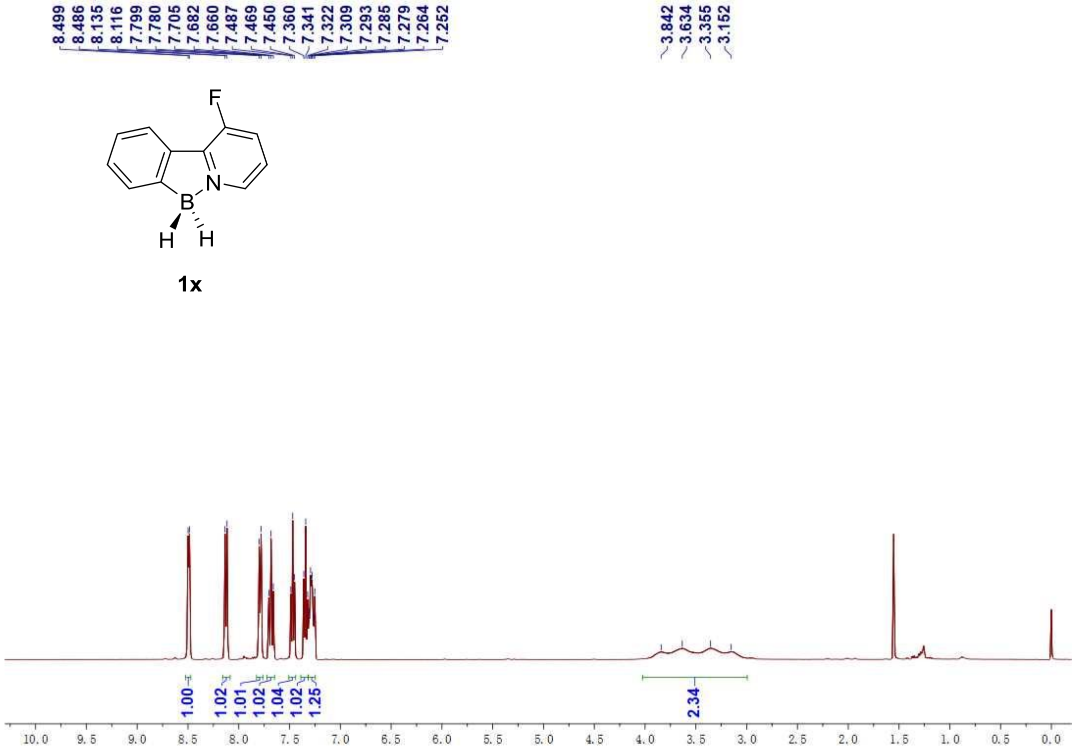

Supplementary Figure 95. ^{1}H NMR spectrum of compound 1x

^{13}C NMR (126 MHz, room temperature, $CDCl_3$)

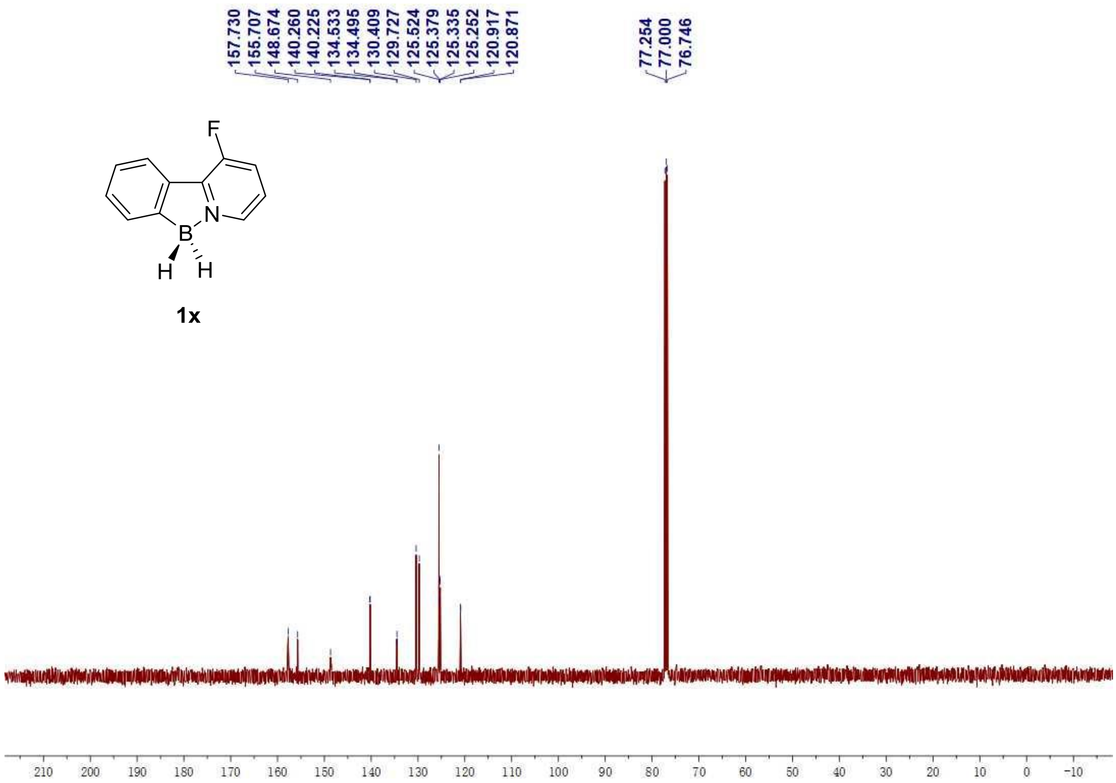

Supplementary Figure 96. ^{13}C NMR spectrum of compound 1x

^{11}B NMR (128 MHz, room temperature, $CDCl_3$)

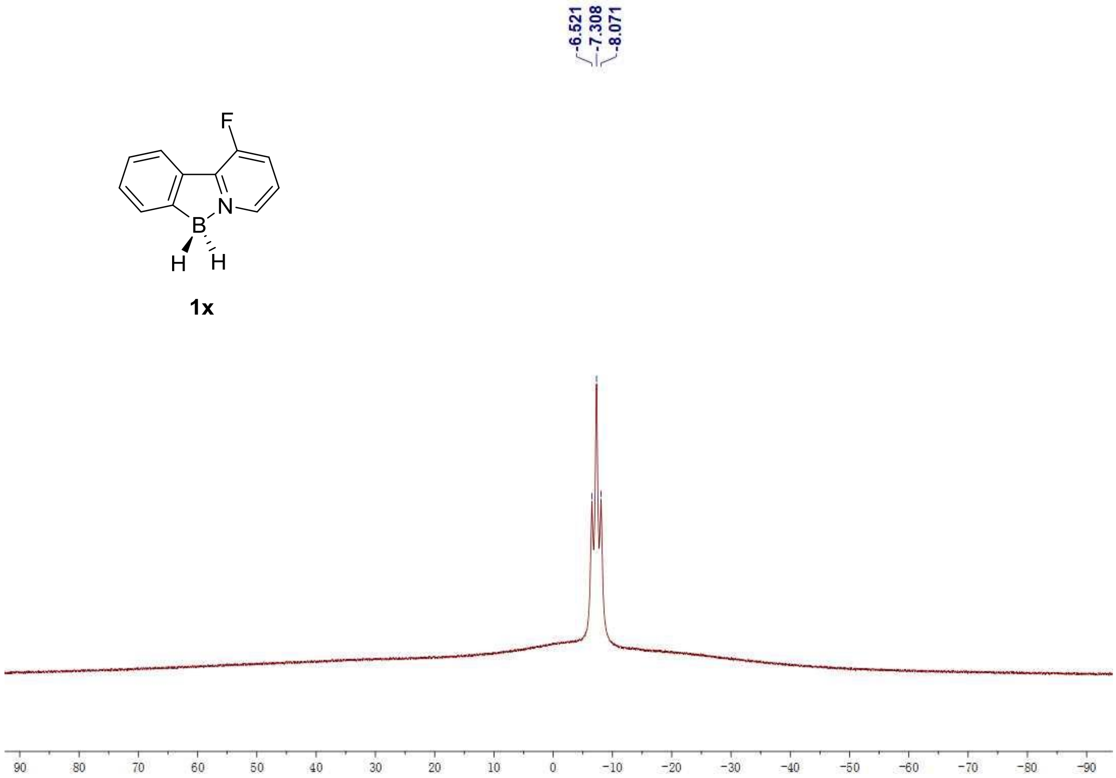

Supplementary Figure 97. ^{11}B NMR spectrum of compound 1x

^{19}F NMR (376 MHz, room temperature, $CDCl_3$)

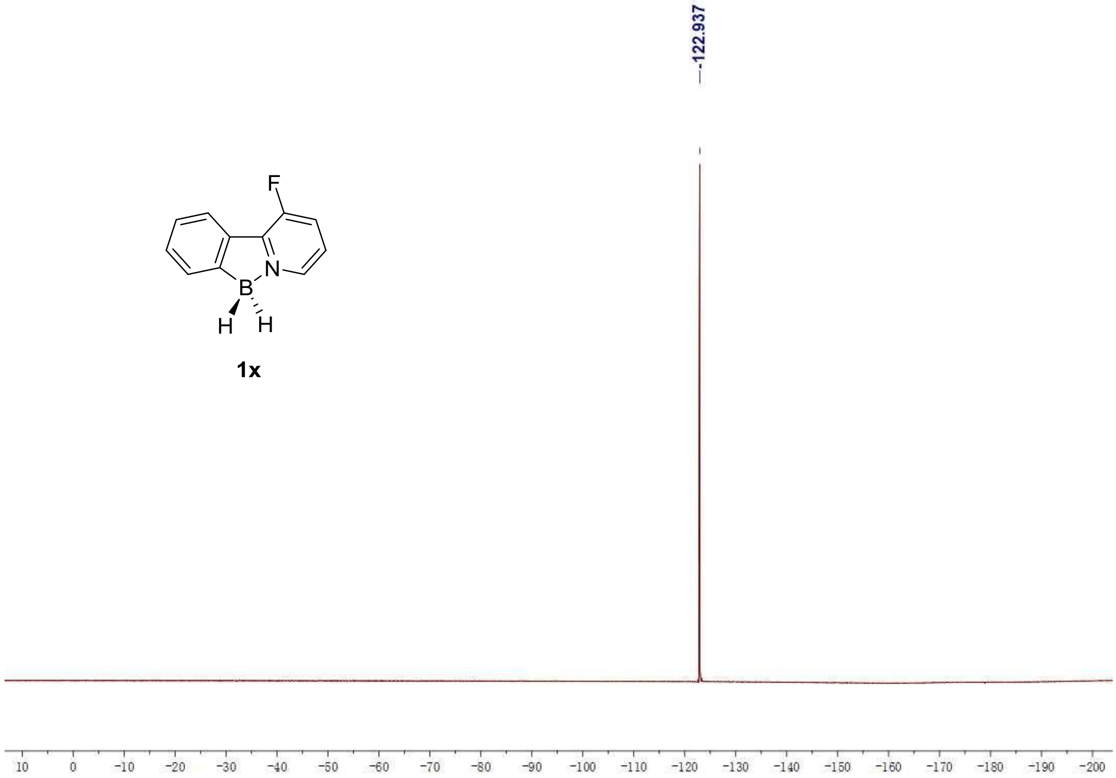

Supplementary Figure 98. ^{19}F NMR spectrum of compound 1x

(diazomethylene)dibenzene (2a)

^{1}H NMR (500 MHz, room temperature, $CDCl_3$)

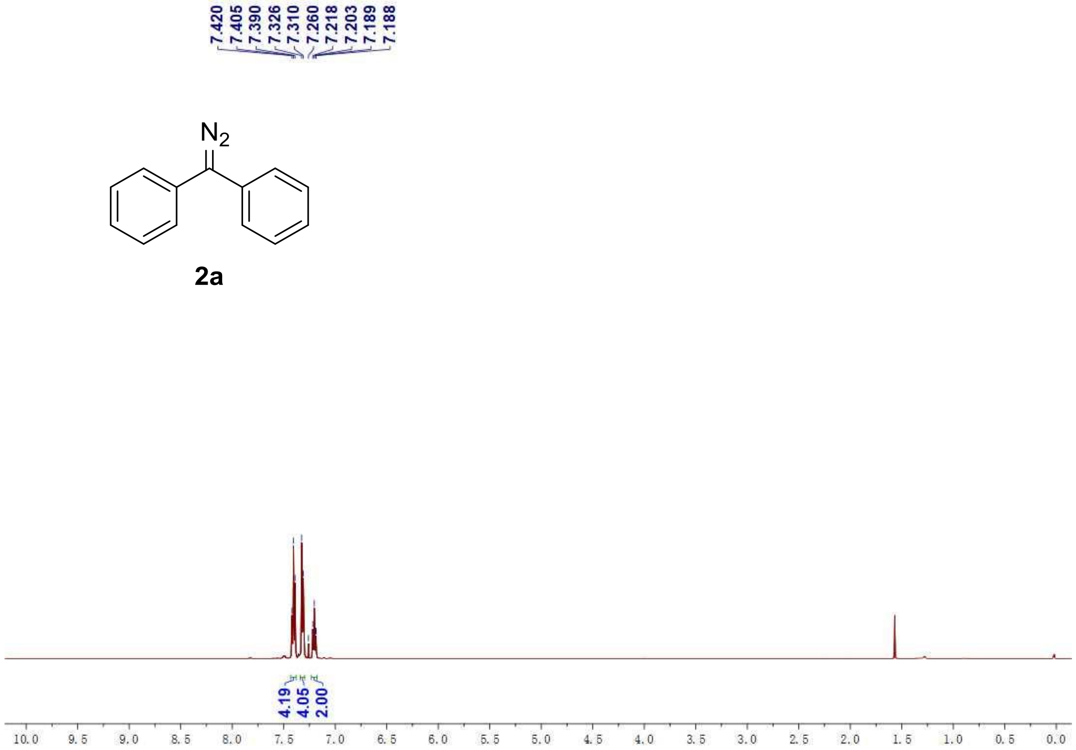

Supplementary Figure 99. ^{1}H NMR spectrum of compound 2a

4,4'-(diazomethylene)bis(fluorobenzene) (2b)

^{1}H NMR (500 MHz, room temperature, $CDCl_3$)

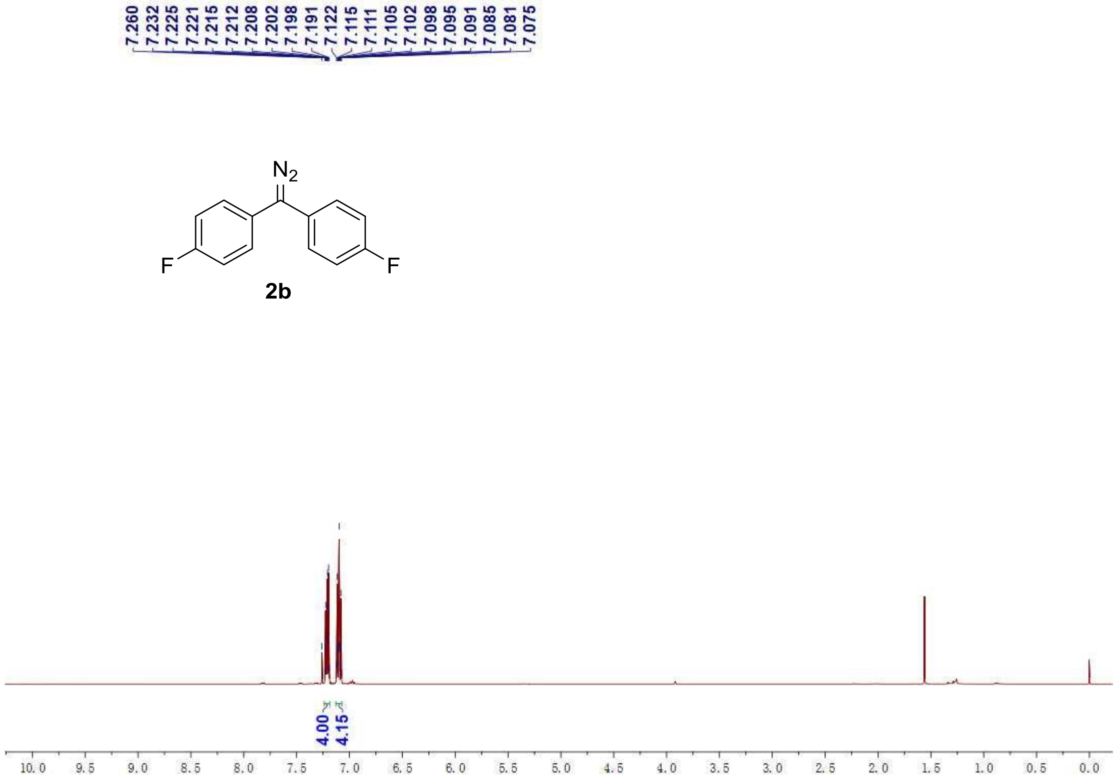

Supplementary Figure 100. ^{1}H NMR spectrum of compound 2b

^{19}F NMR (376 MHz, room temperature, $CDCl_3$)

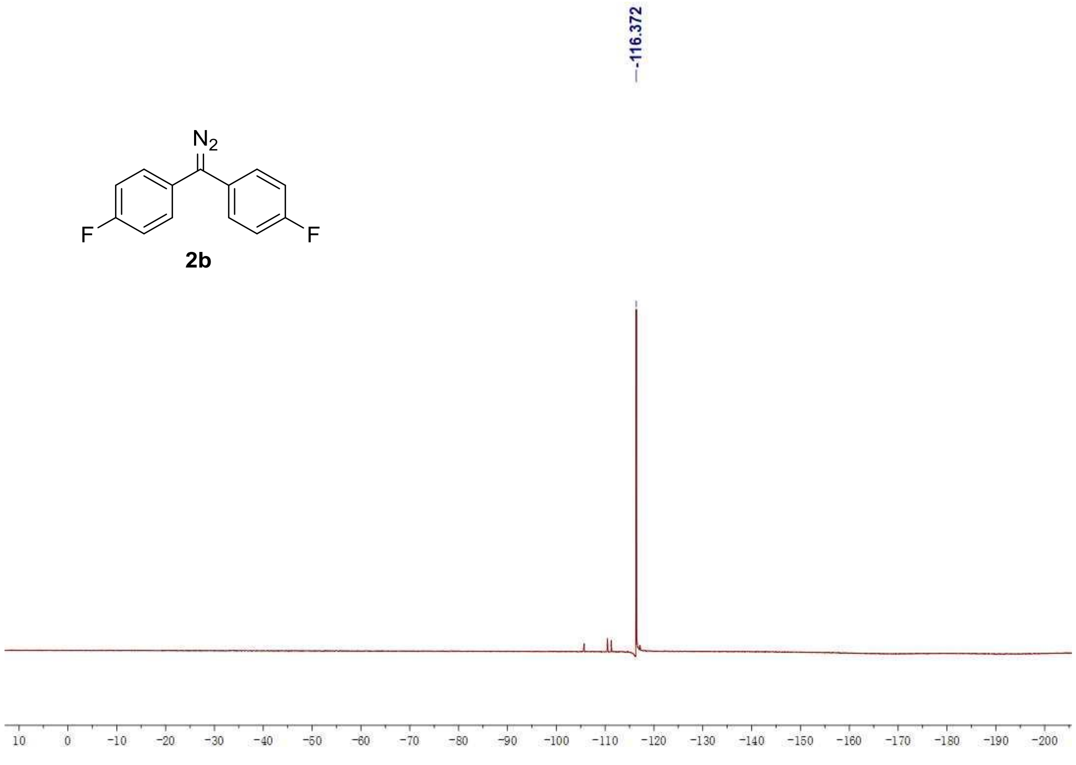

Supplementary Figure 101. ^{19}F NMR spectrum of compound 2b

4,4'-(diazomethylene)bis(chlorobenzene)(2c)

^{1}H NMR (500 MHz, room temperature, $CDCl_3$)

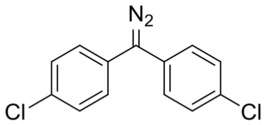

2c

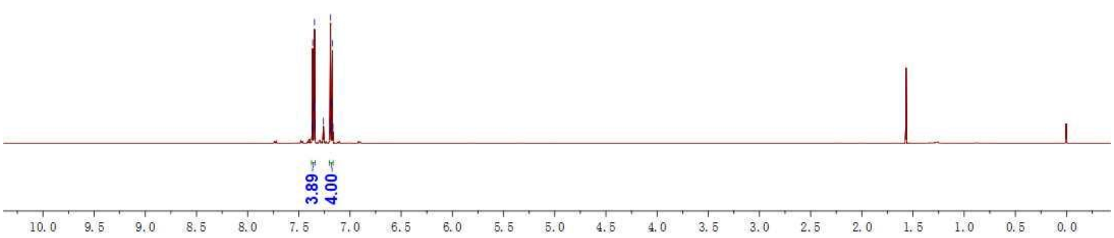

Supplementary Figure 102. ^{1}H NMR spectrum of compound 2c

3,3'-(diazomethylene)bis(chlorobenzene)(2d)

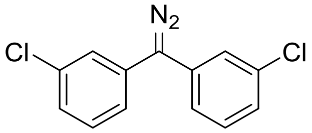

2d

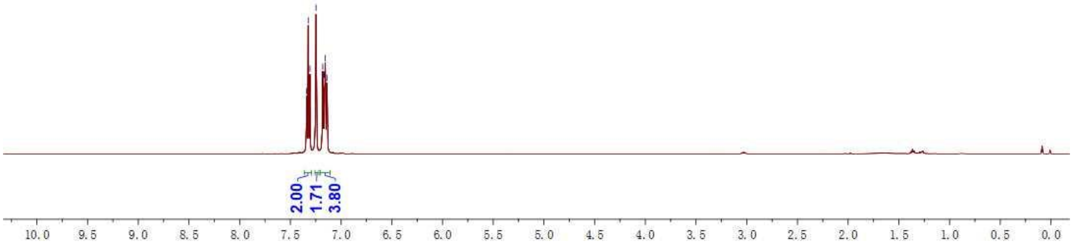

Supplementary Figure 103. ^{1}H NMR spectrum of compound 2d

^{13}C NMR (101 MHz, room temperature, $CDCl_3$)

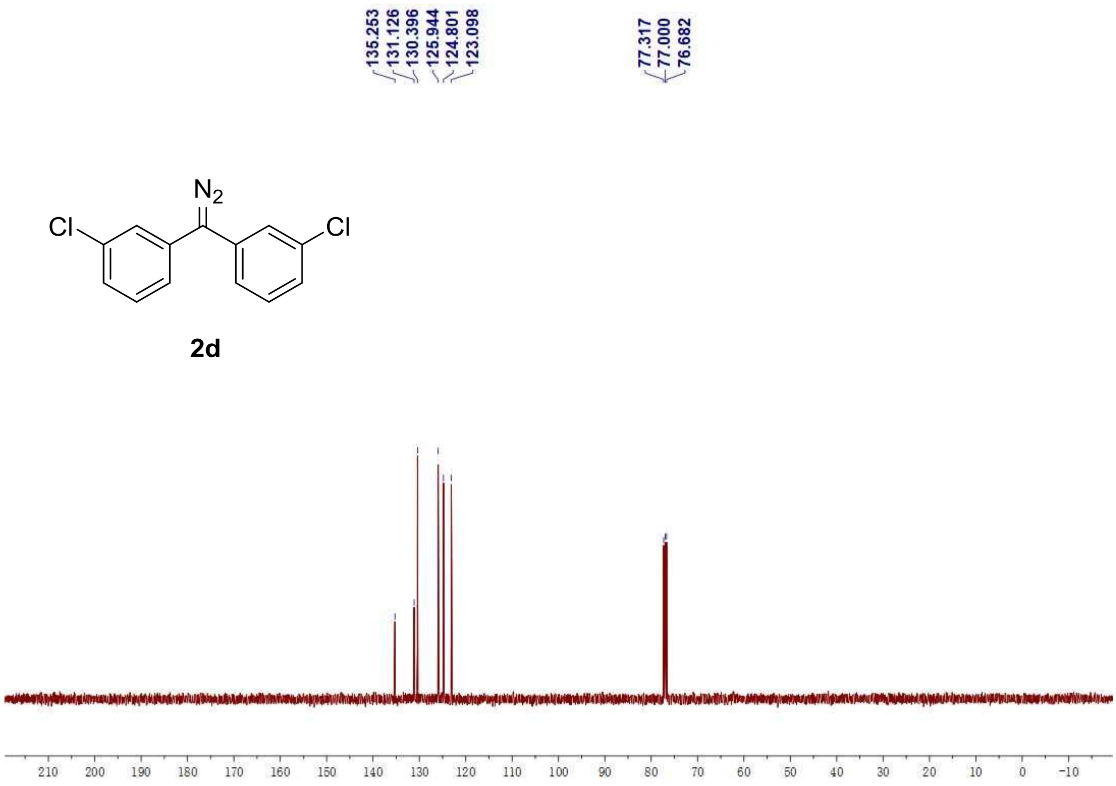

Supplementary Figure 104. ^{13}C NMR spectrum of compound 2d

4,4'-(diazomethylene)bis(methoxybenzene)(2e)

^{1}H NMR (500 MHz, room temperature, $CDCl_3$)

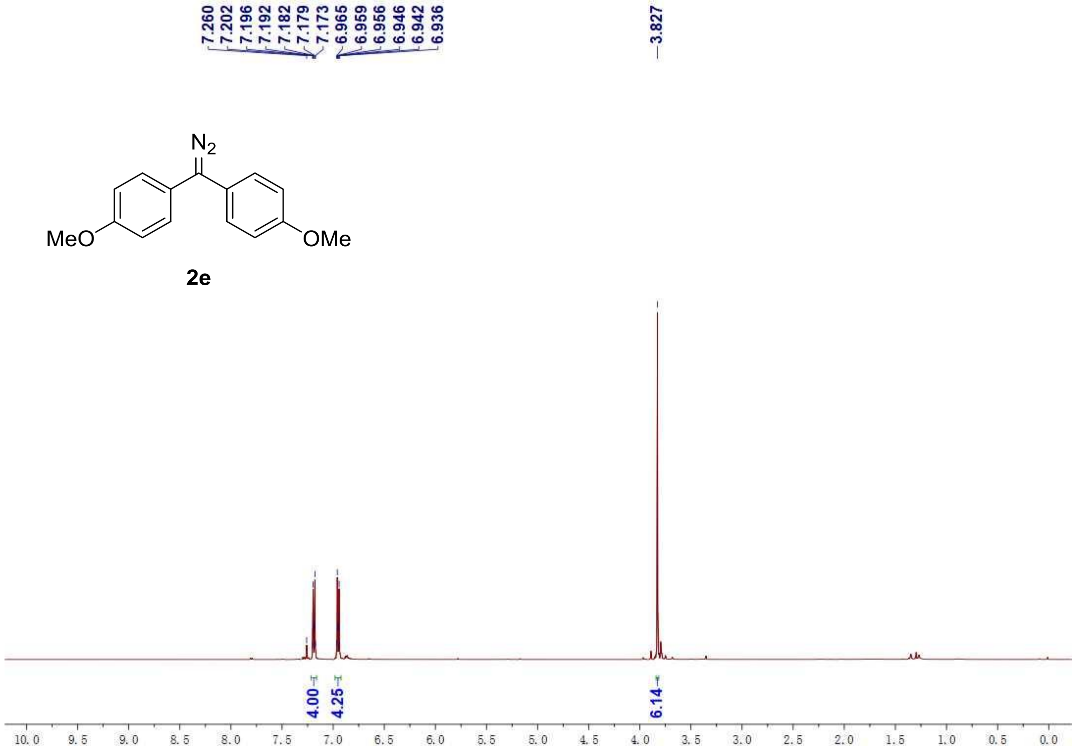

Supplementary Figure 105. ^{1}H NMR spectrum of compound 2e

3,3'-(diazomethylene)bis(methoxybenzene)(2f)

^{1}H NMR (500 MHz, room temperature, $CDCl_3$)

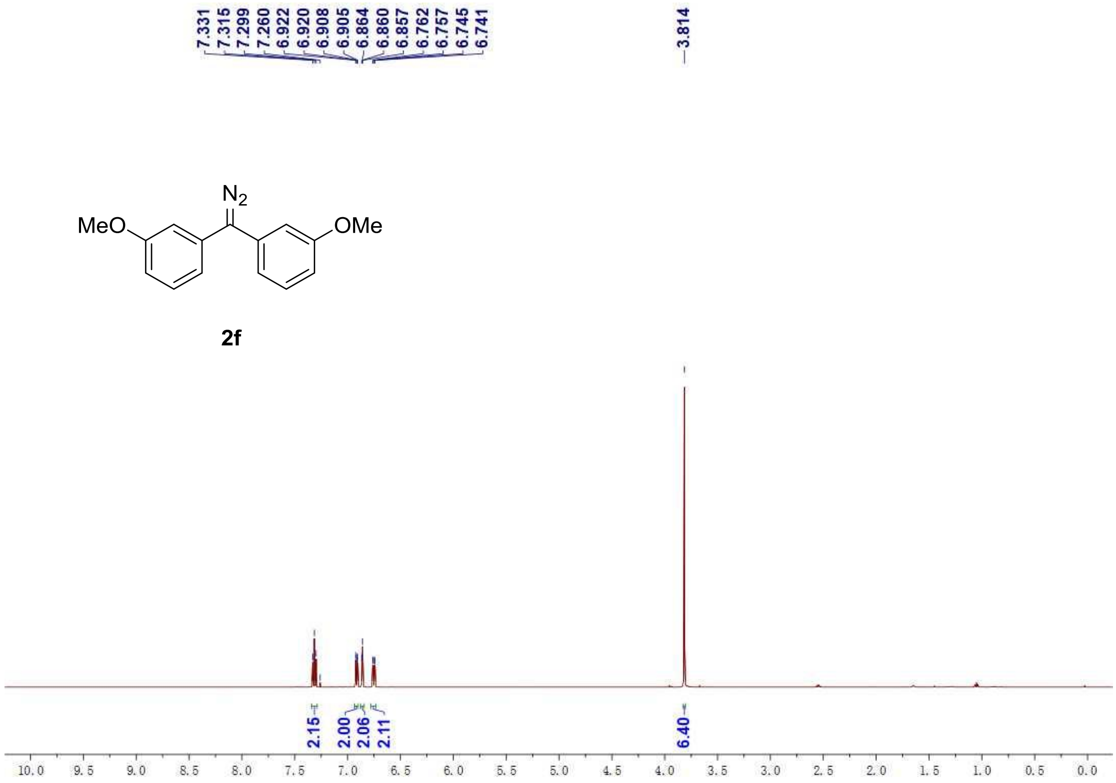

Supplementary Figure 106. ^{1}H NMR spectrum of compound 2f

^{13}C NMR (126 MHz, room temperature, $CDCl_3$)

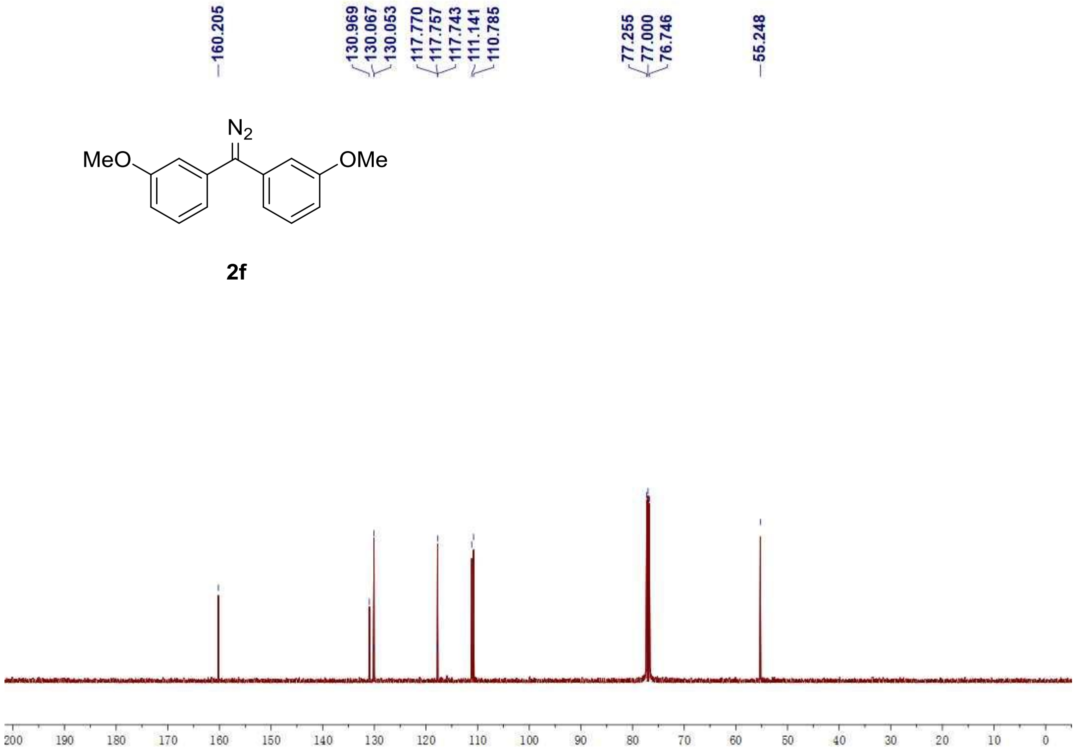

Supplementary Figure 107. ^{13}C NMR spectrum of compound 2f

3,3'-(diazomethylene)bis(methylbenzene)(2g)

^{1}H NMR (500 MHz, room temperature, $CDCl_3$)

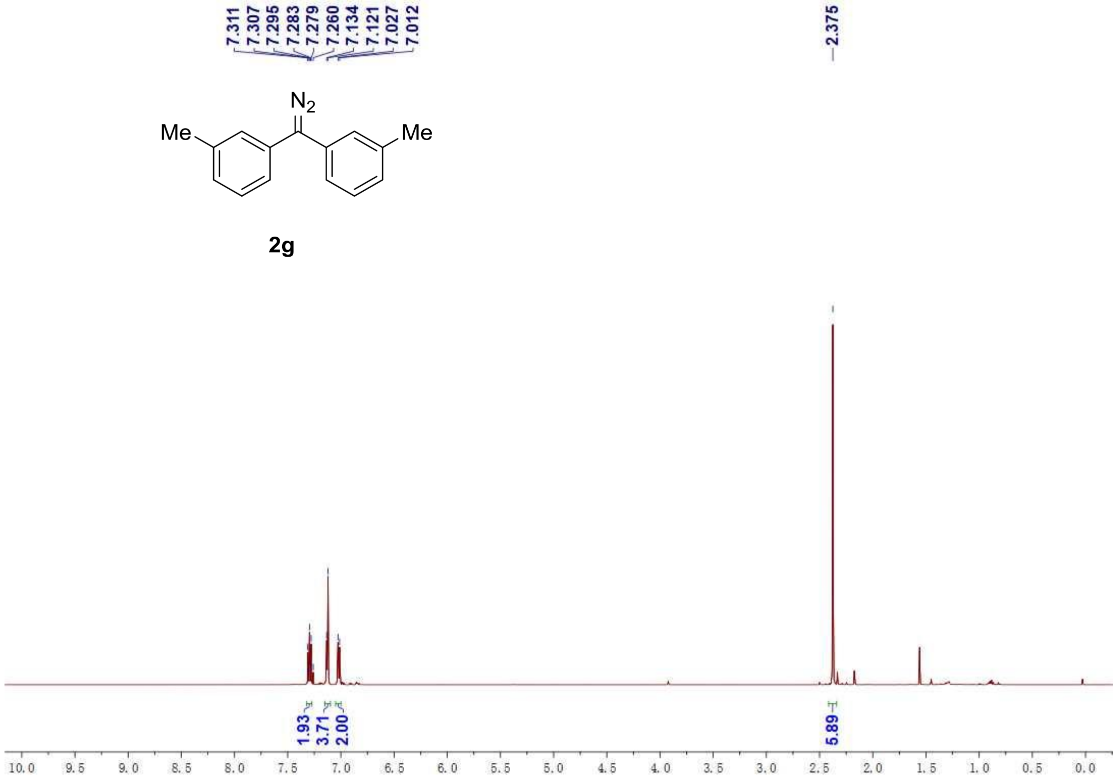

Supplementary Figure 108. ^{1}H NMR spectrum of compound 2g

^{13}C NMR (126 MHz, room temperature, $CDCl_3$)

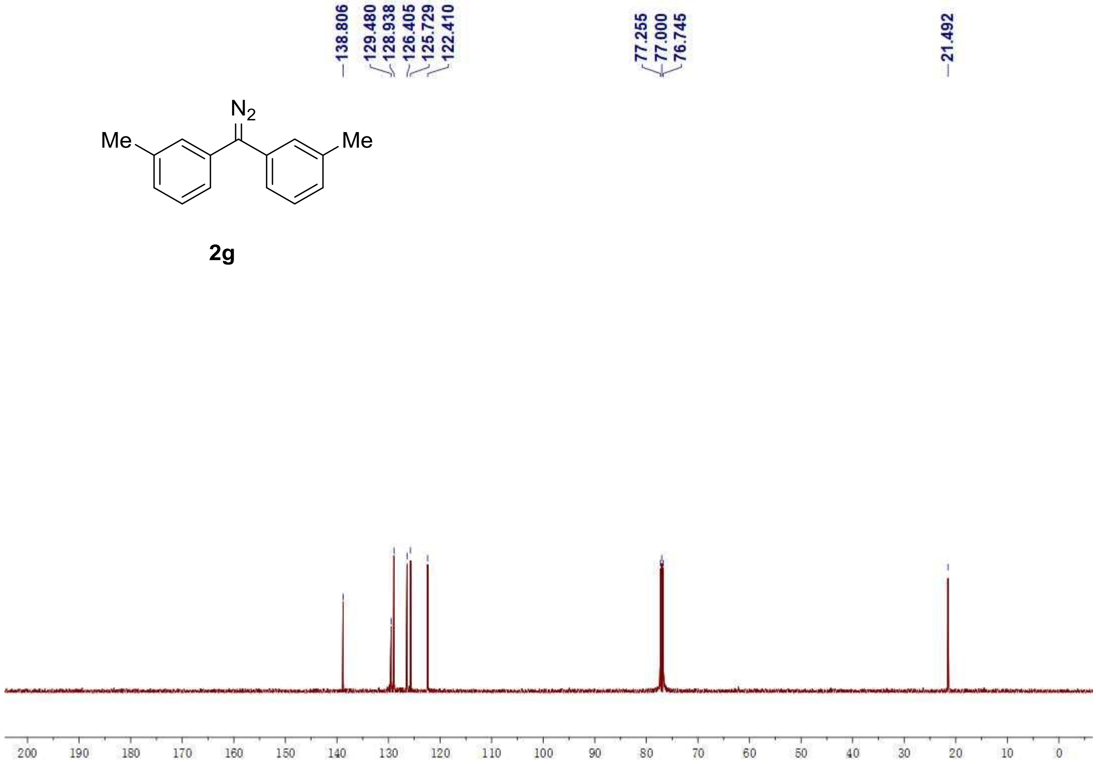

Supplementary Figure 109. ^{13}C NMR spectrum of compound 2g

2,7-dibromo-9-diazo-9H-fluorene (2h)

^{1}H NMR (400 MHz, room temperature, $CDCl_3$)

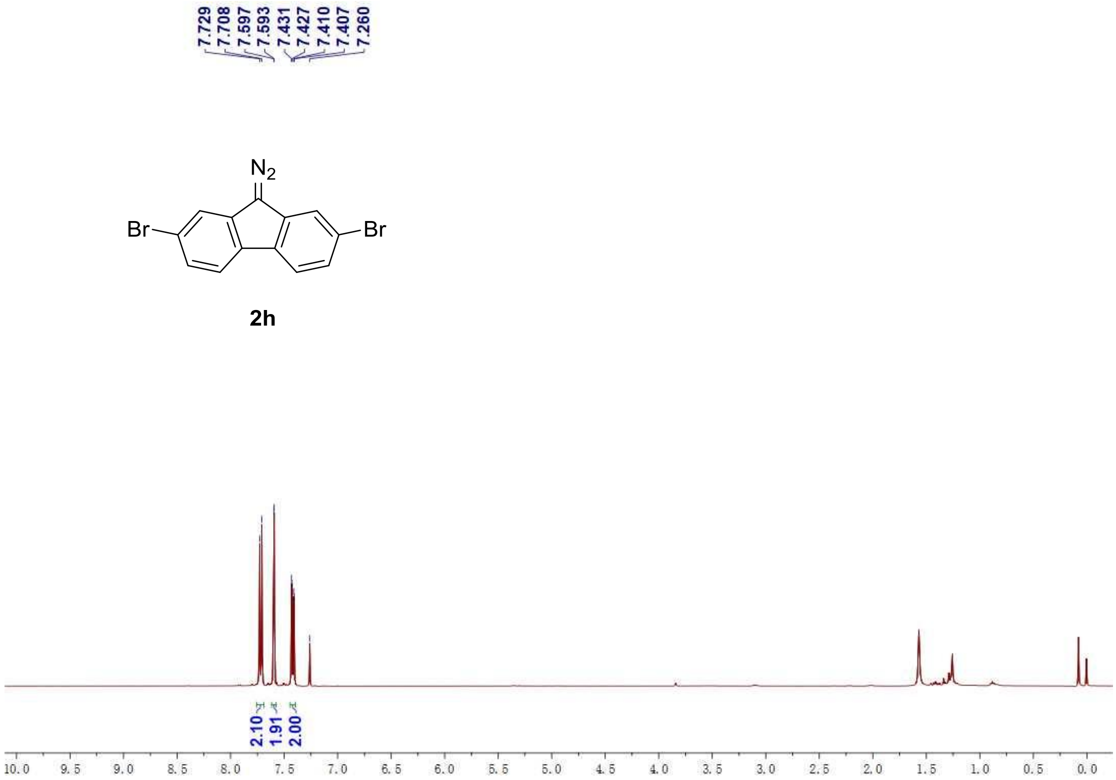

Supplementary Figure 110. ^{1}H NMR spectrum of compound 2h

^{13}C NMR (101 MHz, room temperature, $CDCl_3$)

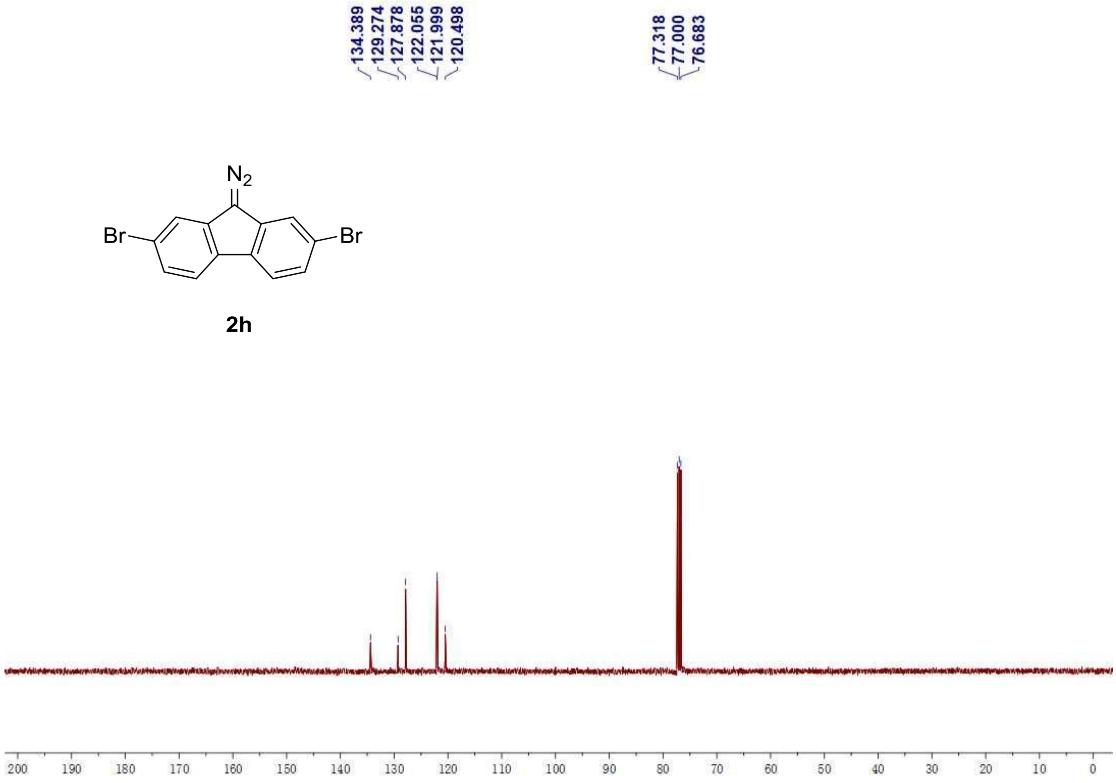

Supplementary Figure 111. ^{13}C NMR spectrum of compound 2h

9-diazo-9H-fluorene (2i)

^{1}H NMR (500 MHz, room temperature, $CDCl_3$)

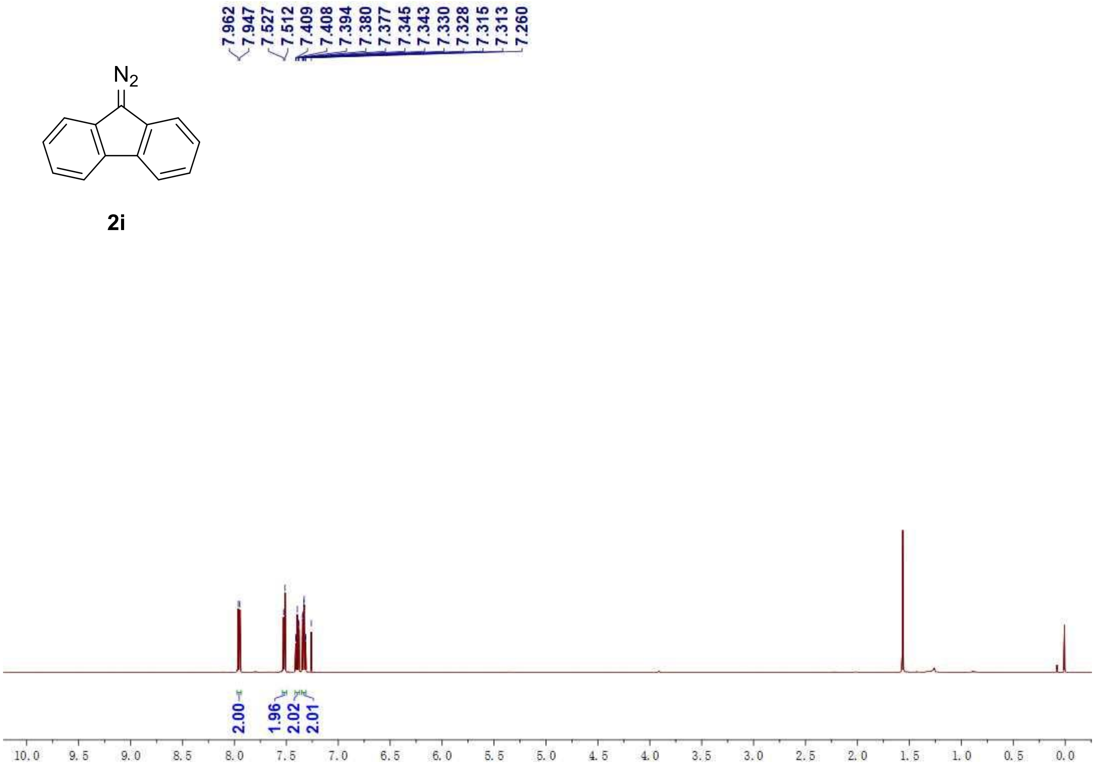

Supplementary Figure 112. ^{1}H NMR spectrum of compound 2i

9-diazo-9H-thioxanthene (2j)

^{1}H NMR (500 MHz, room temperature, $CDCl_3$)

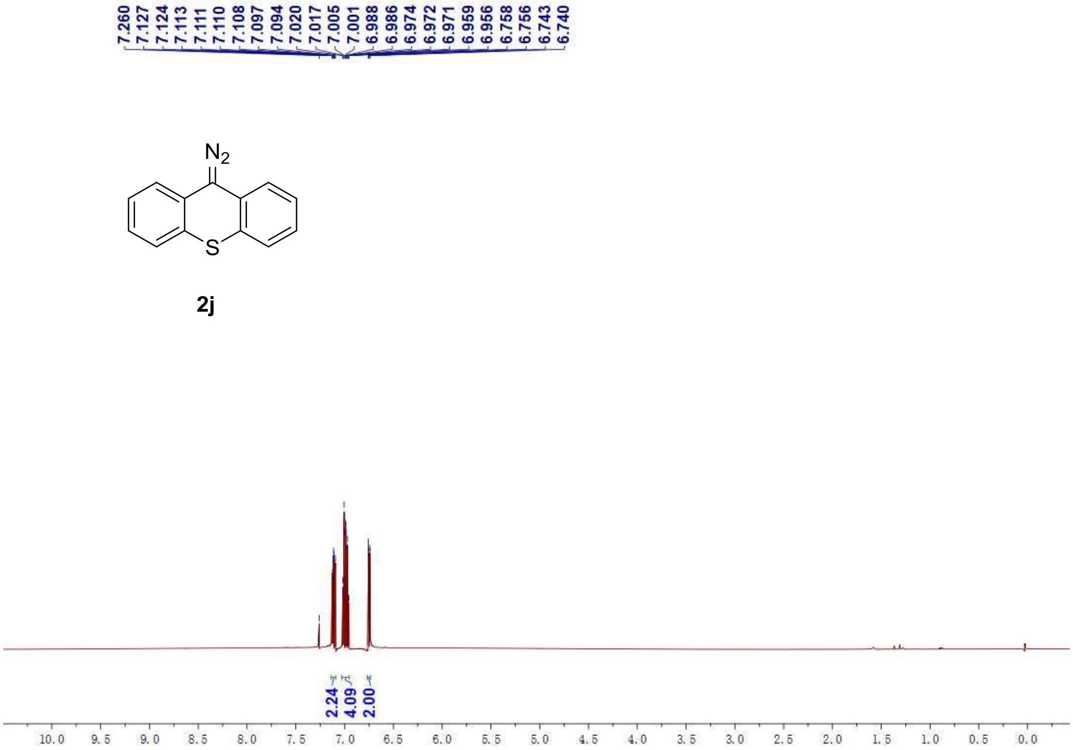

Supplementary Figure 113. ^{1}H NMR spectrum of compound 2j

10-diazoanthracen-9(10H)-one (2k)

^{1}H NMR (400 MHz, room temperature, $CDCl_3$)

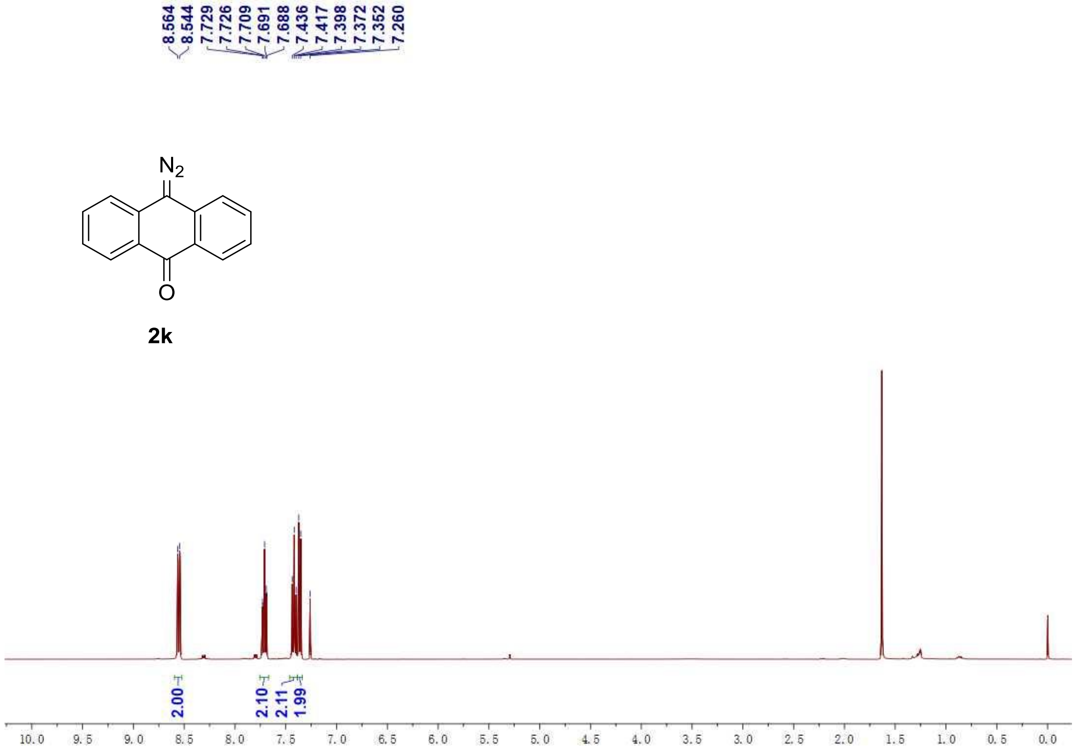

Supplementary Figure 114. ^{1}H NMR spectrum of compound 2k

6H-5 ^{4},6 ^{4}-benzo[3,4][1,2]azaborolo[1,5-a]pyridine-6,6-*d*$_2$ (10-*d*$_2$)

^{1}H NMR (400 MHz, room temperature, $CDCl_3$)

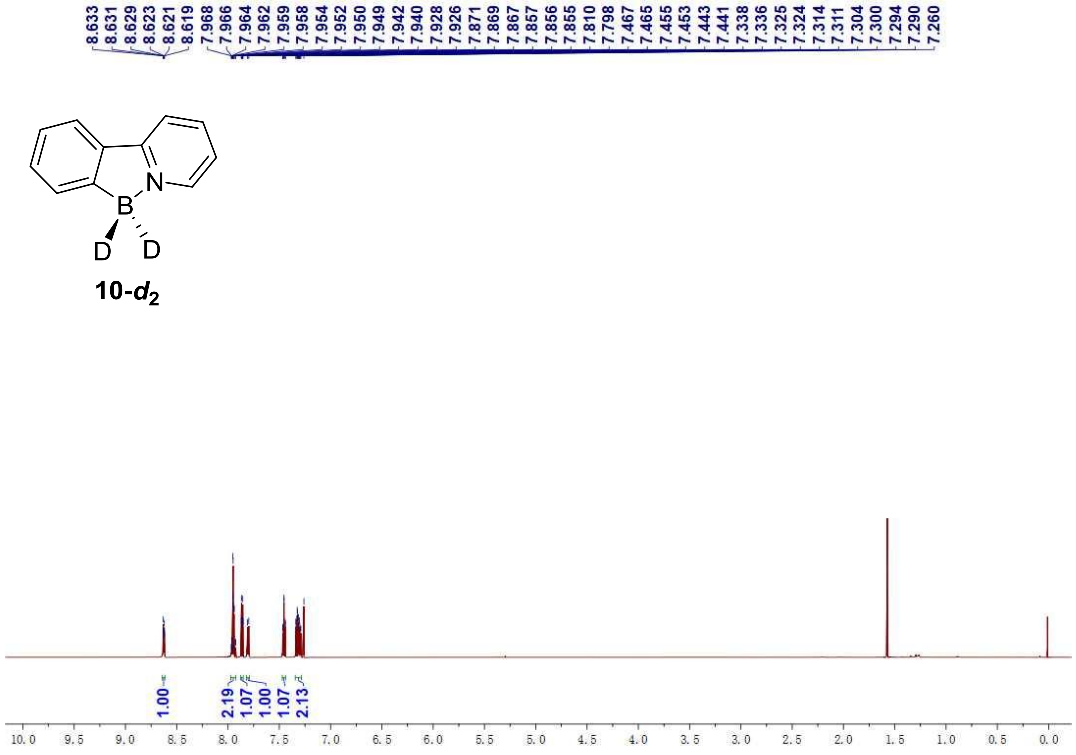

Supplementary Figure 115. ^{1}H NMR spectrum of compound 10-*d*$_2$

ethyl 2-diazo-2-phenylacetate (4a)

^{1}H NMR (600 MHz, room temperature, $CDCl_3$)

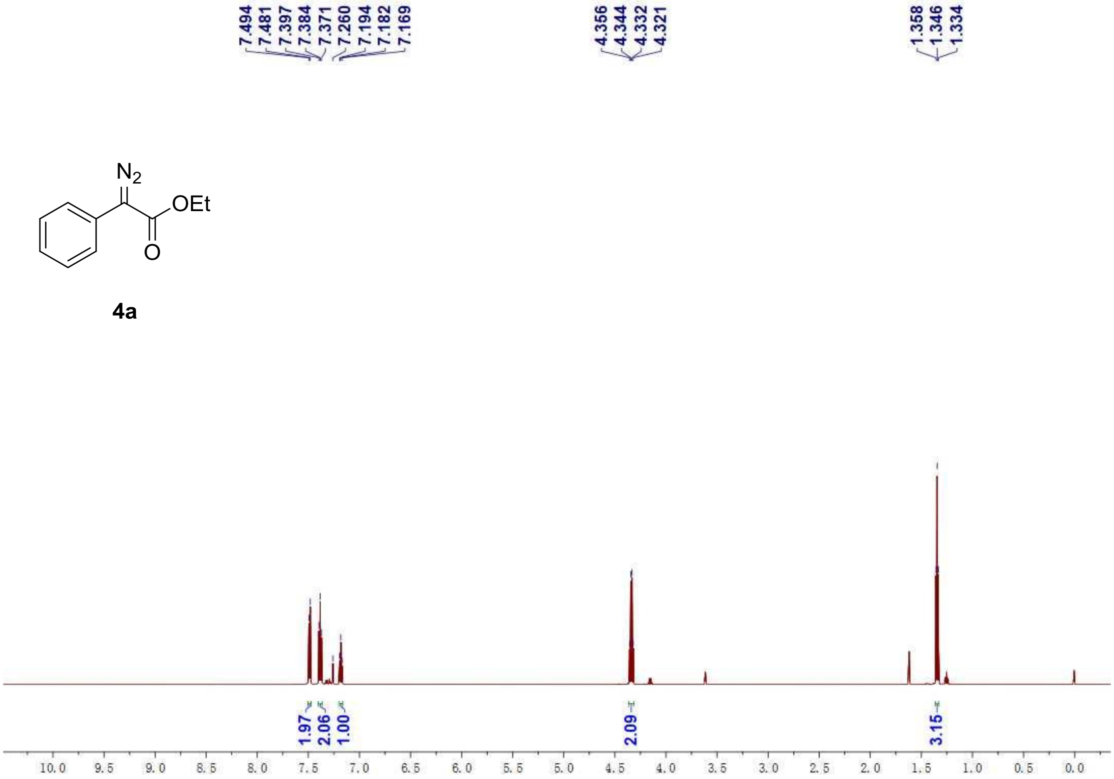

Supplementary Figure 116. ^{1}H NMR spectrum of compound 4a

methyl 2-diazo-2-phenylacetate(4b)

^{1}H NMR (500 MHz, room temperature, $CDCl_3$)

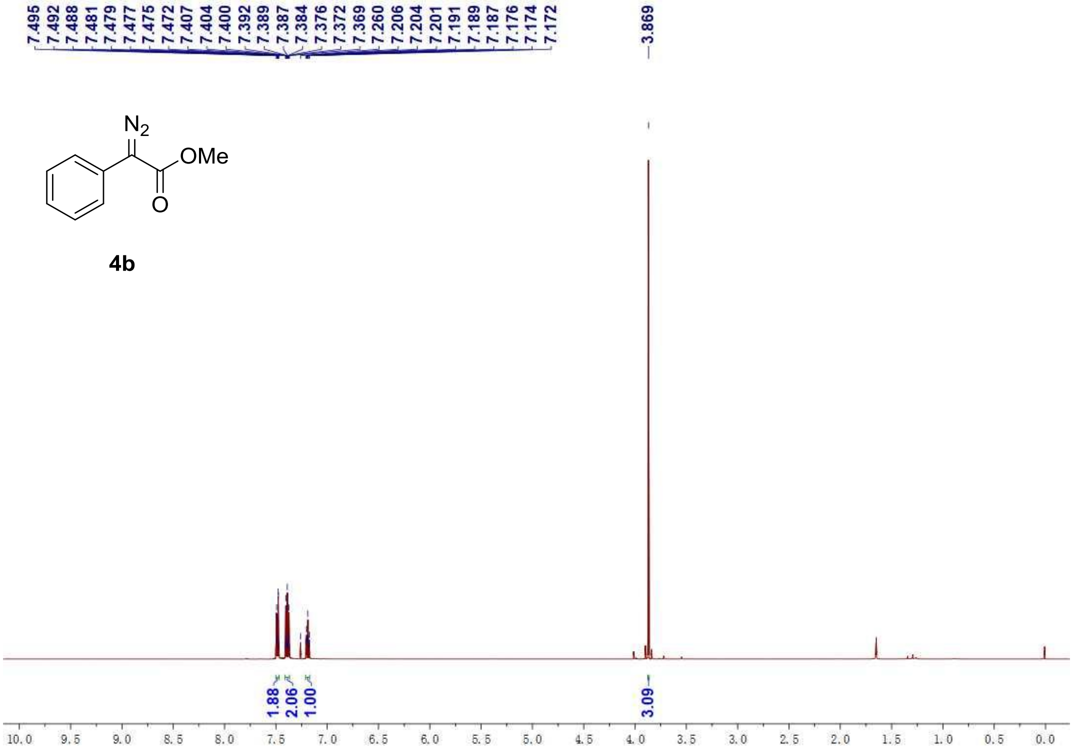

Supplementary Figure 117. ^{1}H NMR spectrum of compound 4b

benzyl 2-diazo-2-phenylacetate(4c)

^{1}H NMR (500 MHz, room temperature, $CDCl_3$)

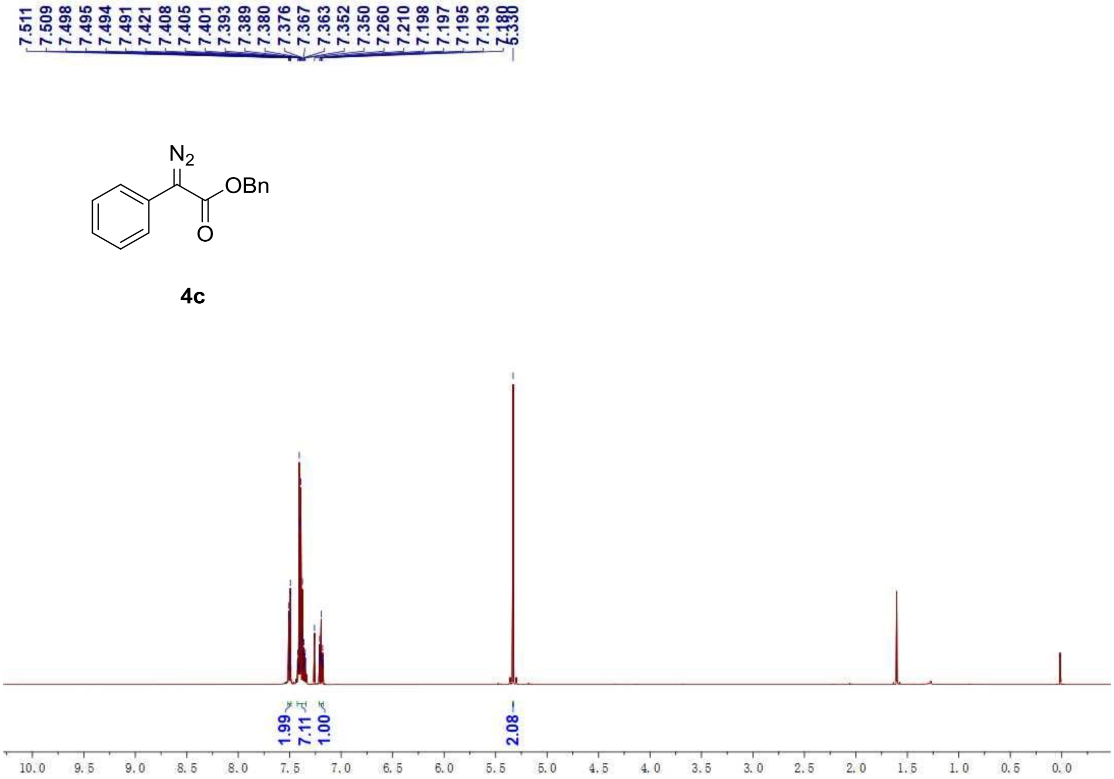

Supplementary Figure 118. ^{1}H NMR spectrum of compound 4c

***tert*-butyl 2-diazo-2-phenylacetate (4d)**

^{1}H NMR (400 MHz, room temperature, $CDCl_3$)

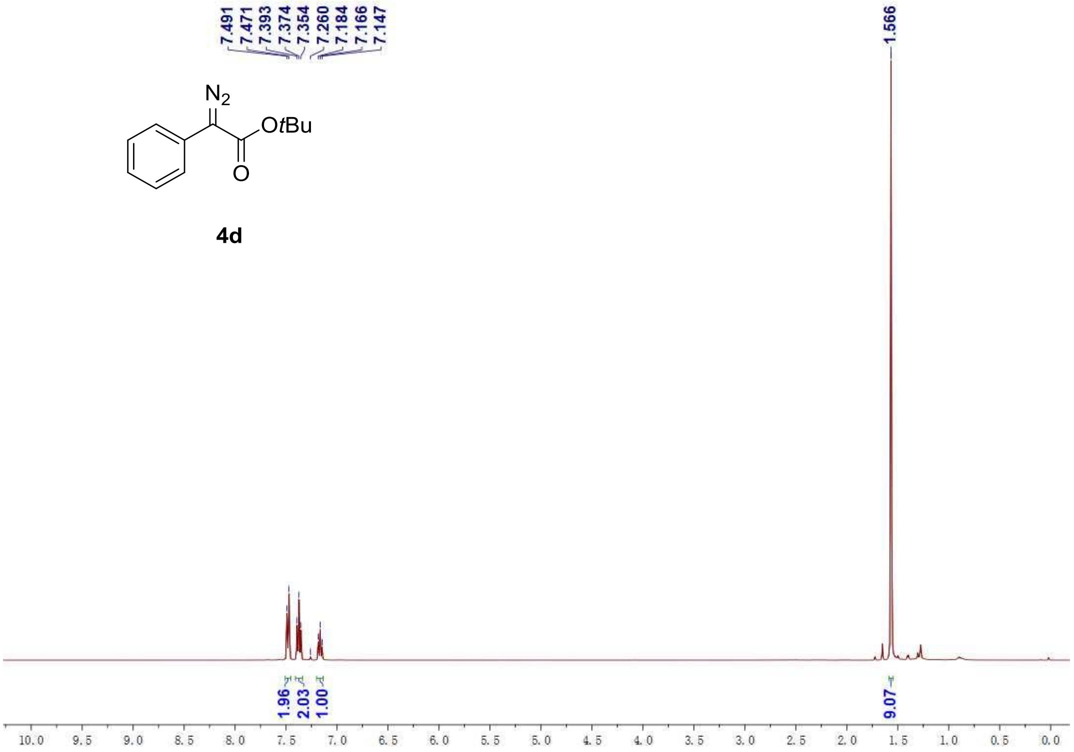

Supplementary Figure 119. ^{1}H NMR spectrum of compound 4d

phenyl 2-diazo-2-phenylacetate (4e)

^{1}H NMR (400 MHz, room temperature, $CDCl_3$)

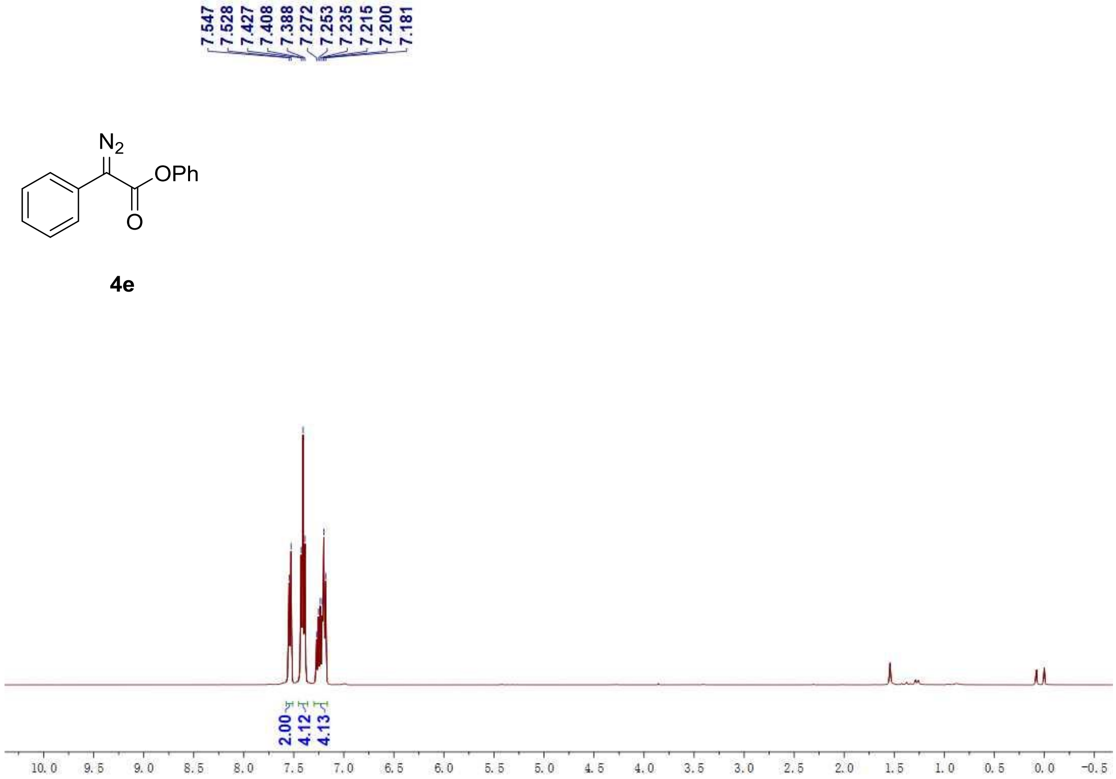

Supplementary Figure 120. ^{1}H NMR spectrum of compound 4e

ethyl 2-(4-(tert-butyl)phenyl)-2-diazoacetate (4f)

^{1}H NMR (500 MHz, room temperature, $CDCl_3$)

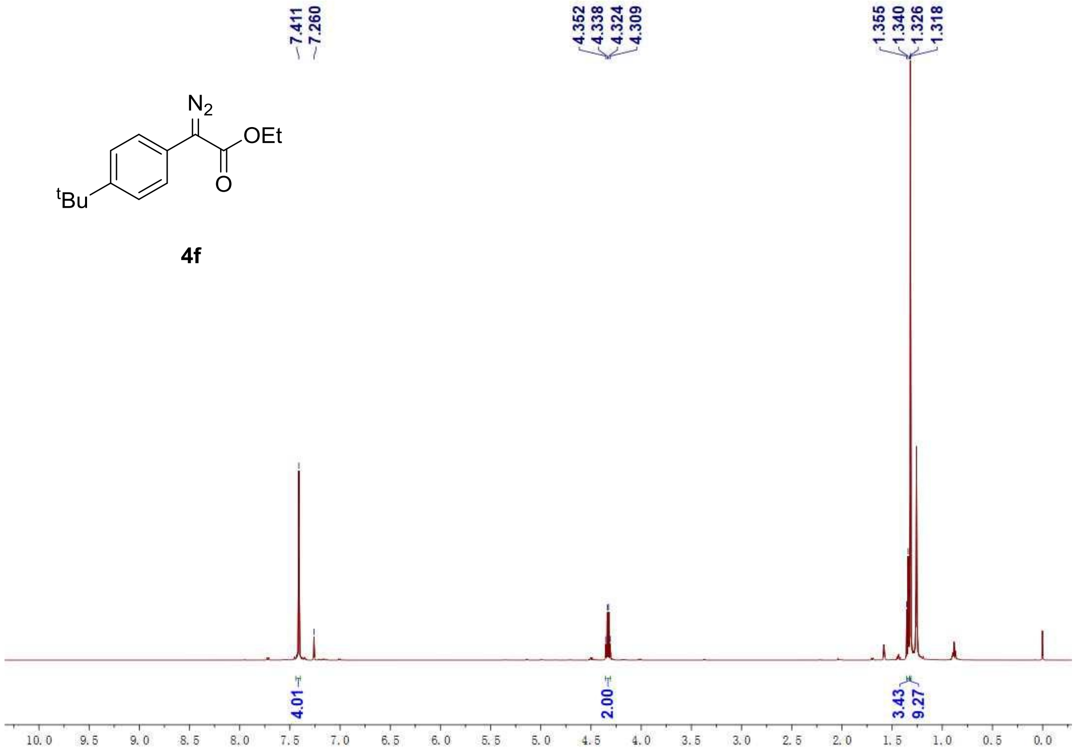

Supplementary Figure 121. NMR spectrum of compound 4f

ethyl 2-diazo-2-(p-tolyl)acetate (4g)

^{1}H NMR (600 MHz, room temperature, $CDCl_3$)

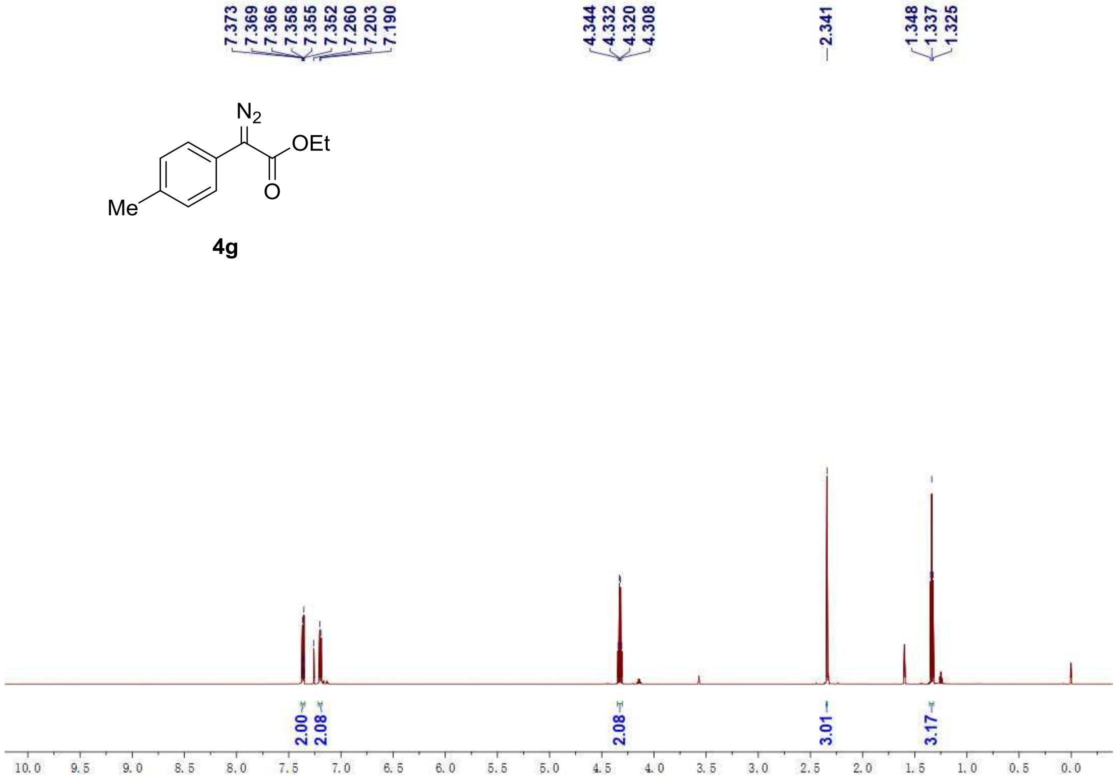

Supplementary Figure 122. ^{1}H NMR spectrum of compound 4g

ethyl 2-diazo-2-(m-tolyl)acetate (4h)

^{1}H NMR (500 MHz, room temperature, $CDCl_3$)

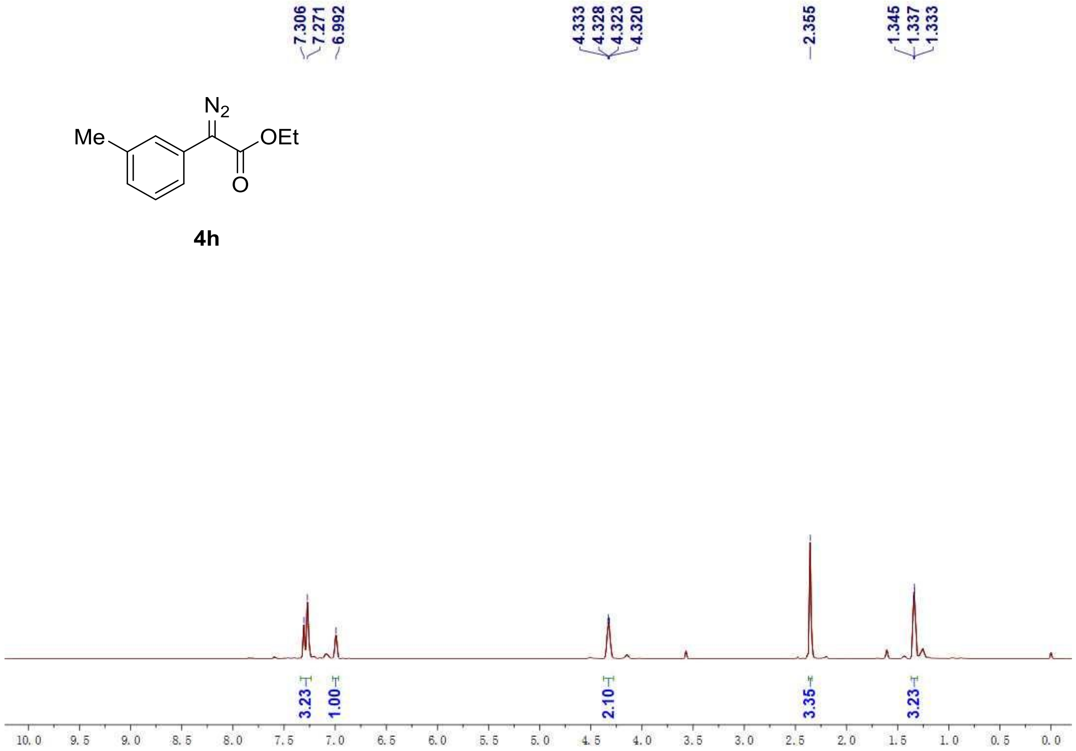

Supplementary Figure 123. ^{1}H NMR spectrum of compound 4h

ethyl 2-diazo-2-(3-methoxyphenyl)acetate (4i)

^{1}H NMR (400 MHz, room temperature, $CDCl_3$)

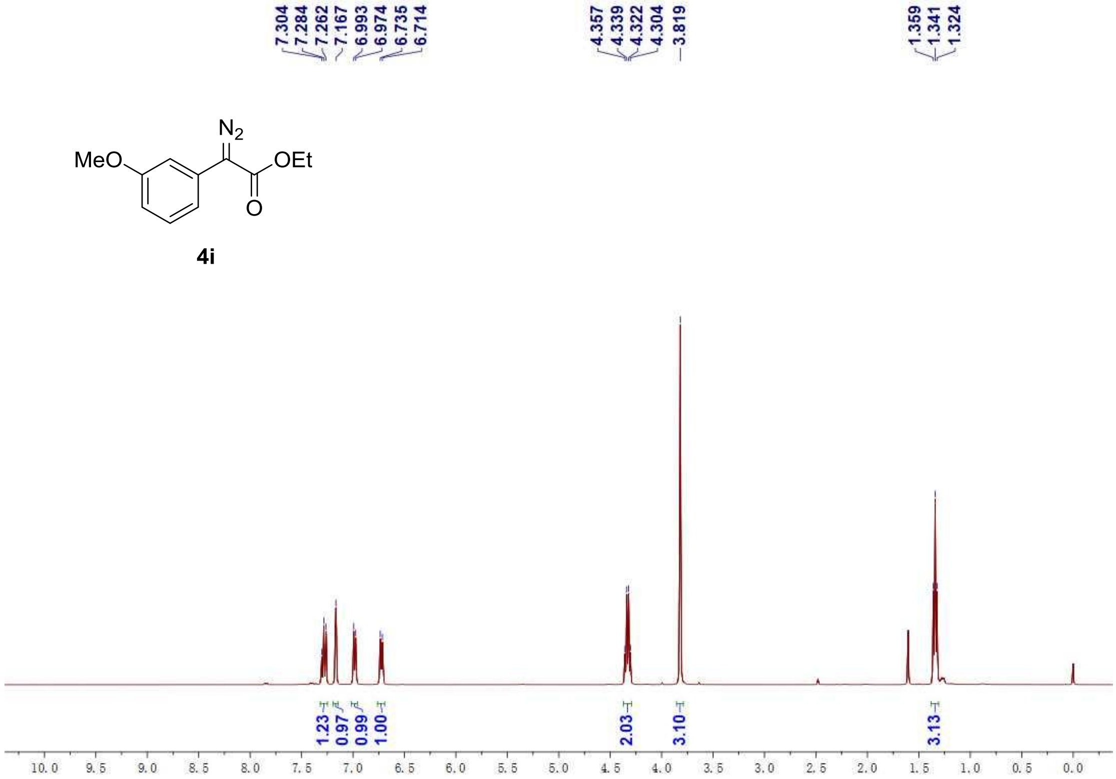

Supplementary Figure 124. ^{1}H NMR spectrum of compound 4i

ethyl 2-(benzo[d][1,3]dioxol-5-yl)-2-diazoacetate (4j)

^{1}H NMR (600 MHz, room temperature, $CDCl_3$)

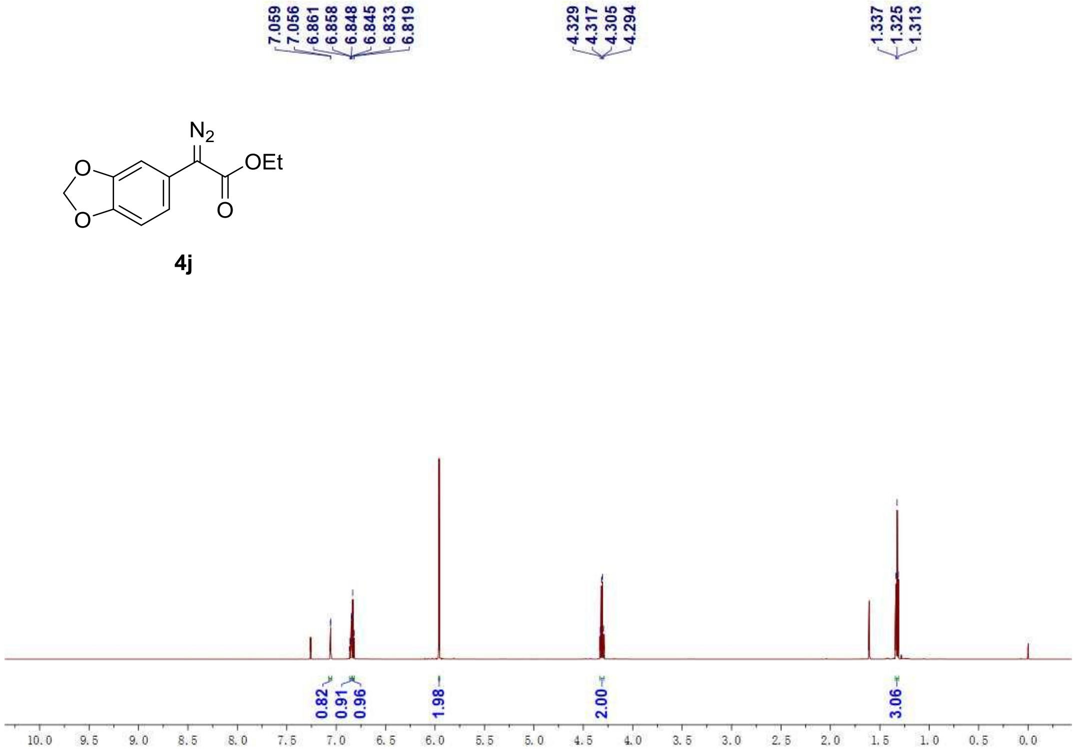

Supplementary Figure 125. ^{1}H NMR spectrum of compound 4j

ethyl 2-([1,1'-biphenyl]-4-yl)-2-diazoacetate (4k)

^{1}H NMR (500 MHz, room temperature, $CDCl_3$)

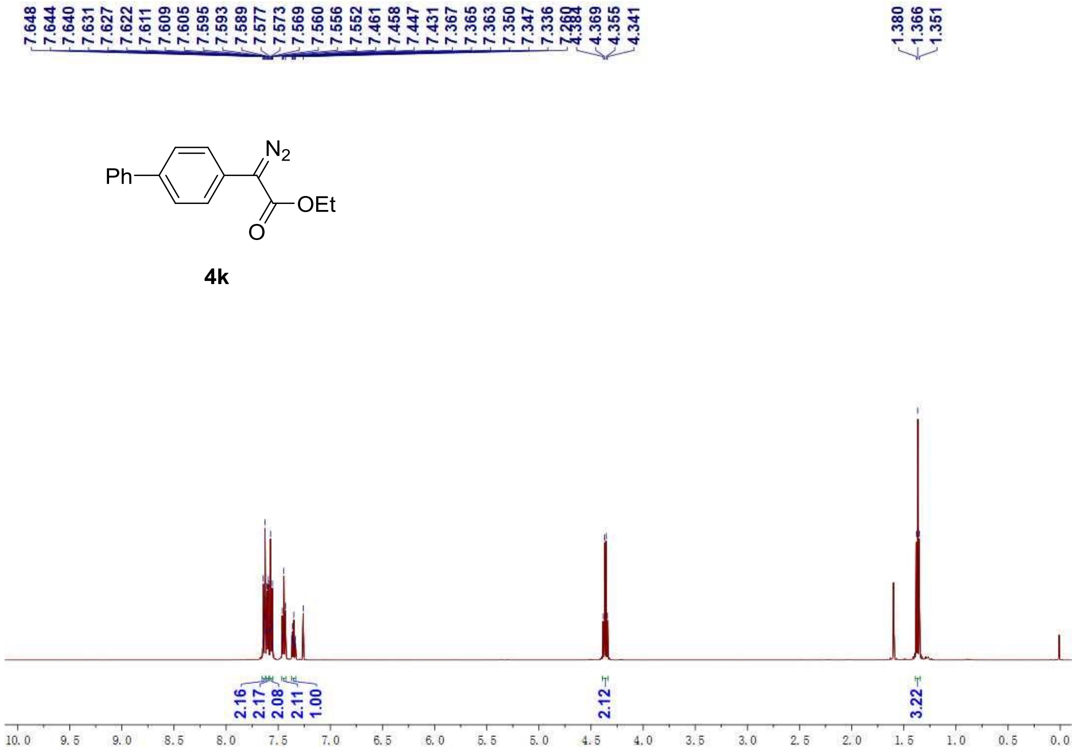

Supplementary Figure 126. ^{1}H NMR spectrum of compound 4k

methyl 3-(1-diazo-2-ethoxy-2-oxoethyl)benzoate (4l)

^{1}H NMR (500 MHz, room temperature, $CDCl_3$)

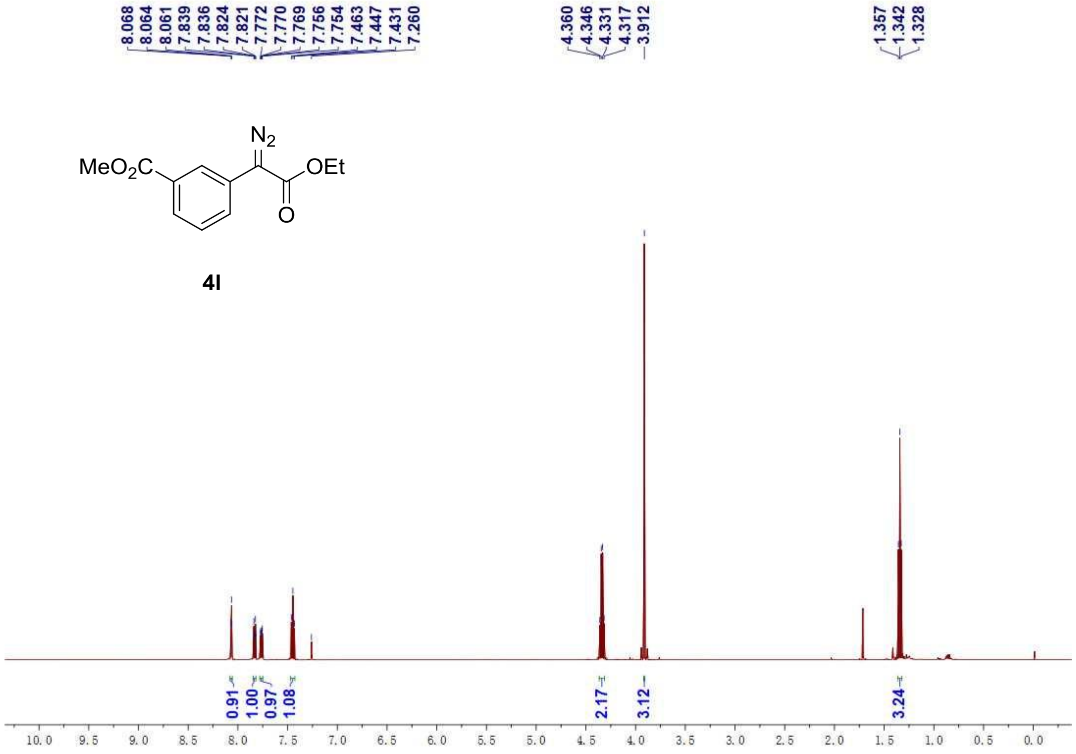

Supplementary Figure 127. ^{1}H NMR spectrum of compound 4l

ethyl 2-diazo-2-(4-fluorophenyl)acetate (4m)

^{1}H NMR (500 MHz, room temperature, $CDCl_3$)

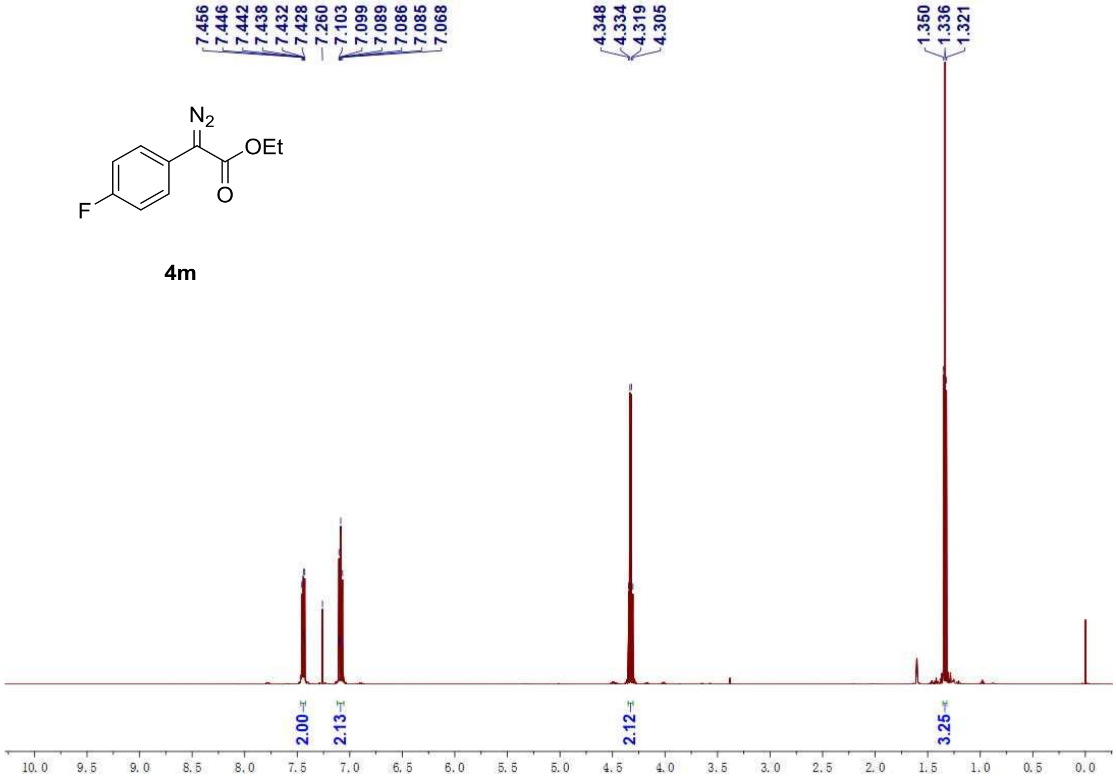

Supplementary Figure 128. ^{1}H NMR spectrum of compound 4m

^{19}F NMR (376 MHz, room temperature, $CDCl_3$)

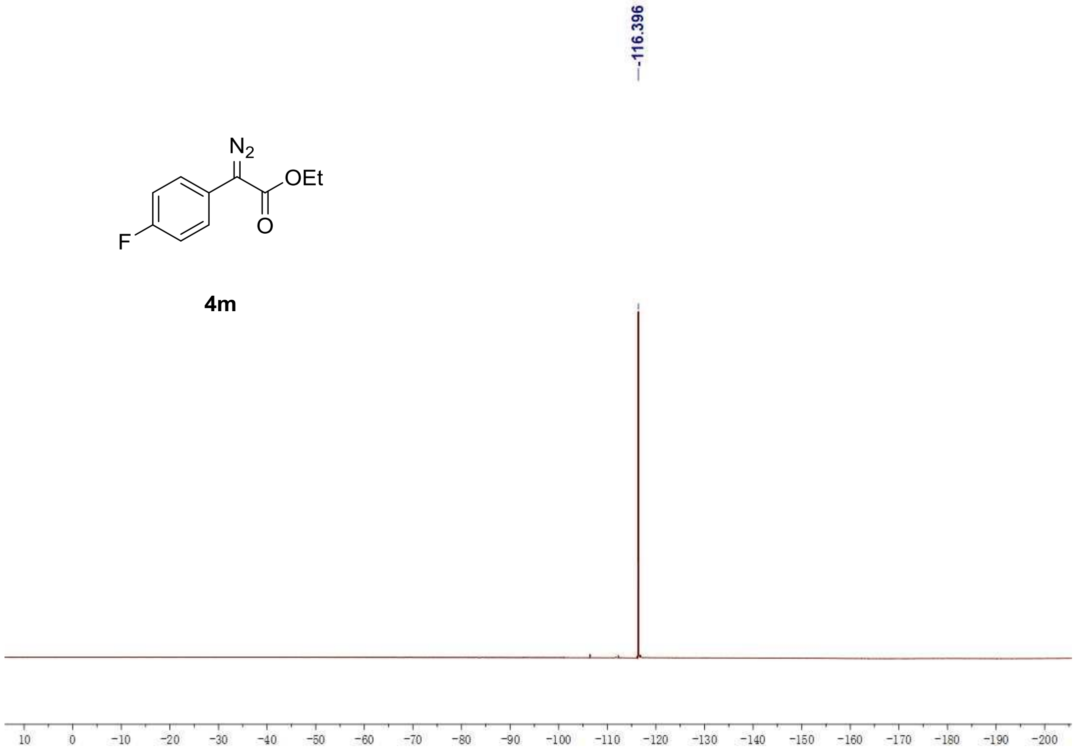

Supplementary Figure 129. ^{19}F NMR spectrum of compound 4m

ethyl 2-(4-chlorophenyl)-2-diazoacetate (4n)

^{1}H NMR (500 MHz, room temperature, CDCl$_3$)

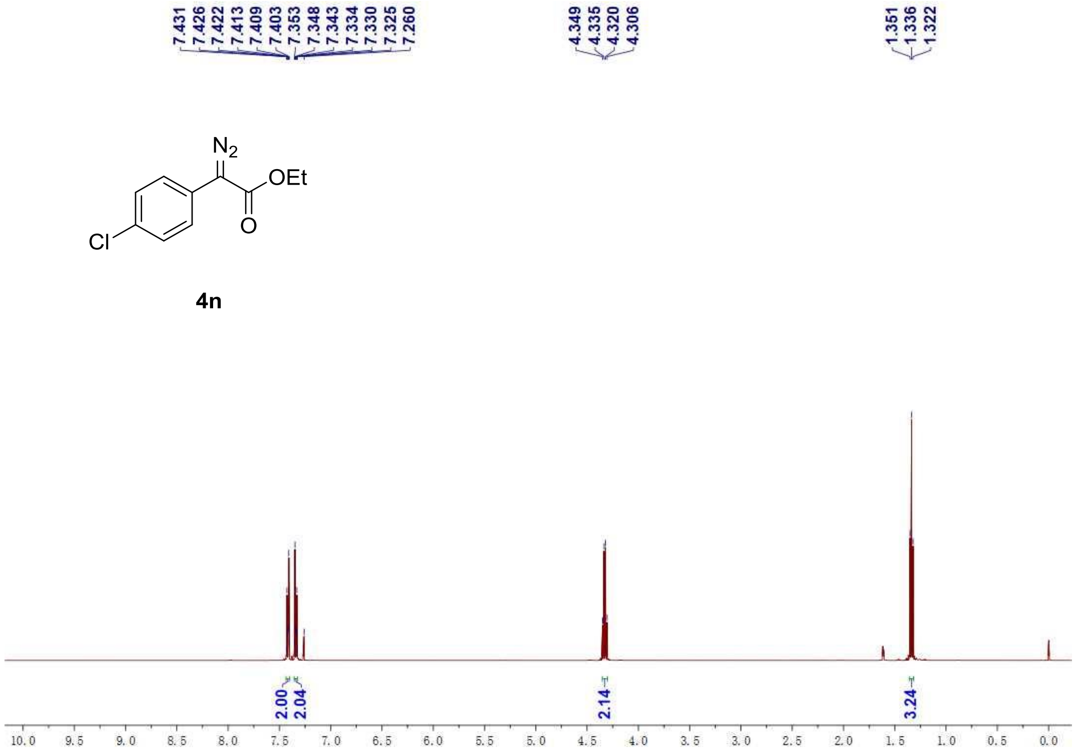

Supplementary Figure 130. ^{1}H NMR spectrum of compound 4n

ethyl 2-(4-bromophenyl)-2-diazoacetate (4o)

^{1}H NMR (500 MHz, room temperature, CDCl$_3$)

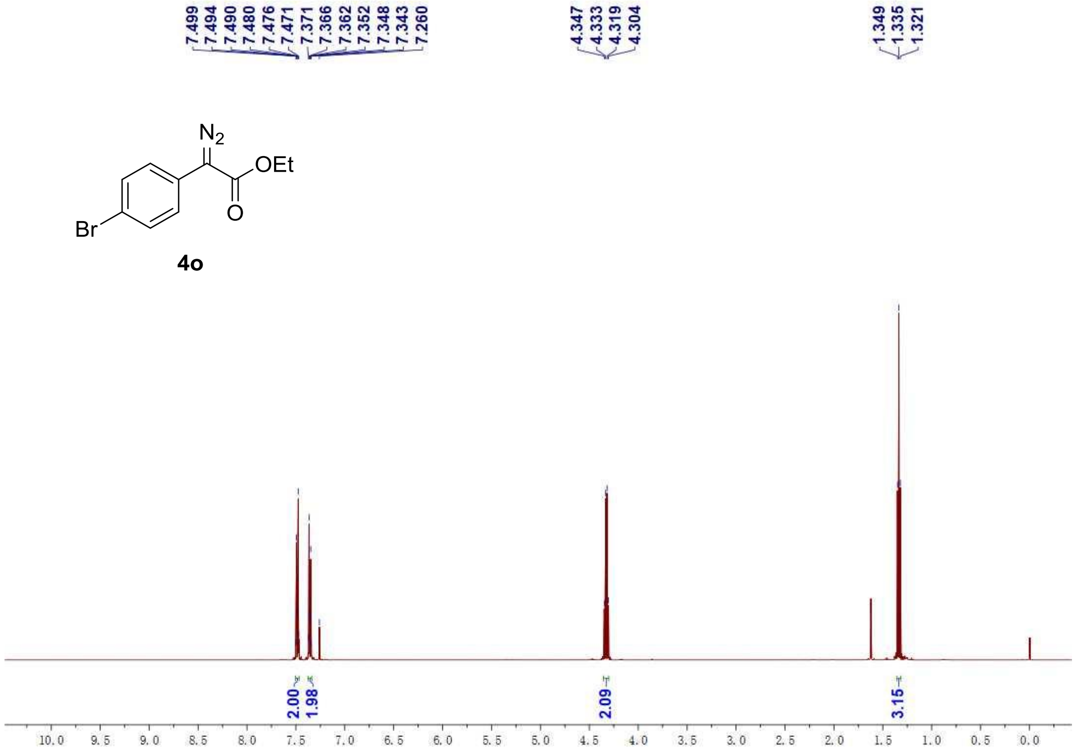

Supplementary Figure 131. ^{1}H NMR spectrum of compound 4o

ethyl 2-diazo-2-(naphthalen-2-yl)acetate (4p)

^{1}H NMR (500 MHz, room temperature, $CDCl_3$)

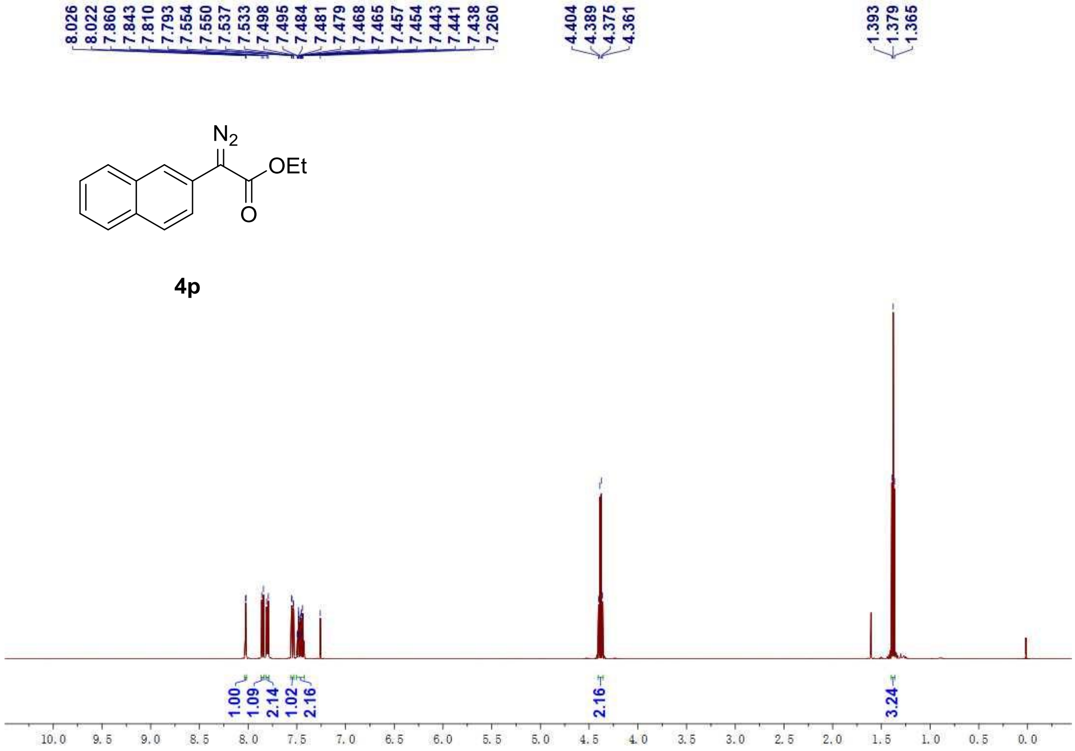

Supplementary Figure 132. ^{1}H NMR spectrum of compound 4p

6-benzhydryl-7-chloro-6H-5 ^{4}-benzo[3,4][1,2]azaborolo[1,5-a]pyridine (3a)

^{1}H NMR (500 MHz, room temperature, $CDCl_3$)

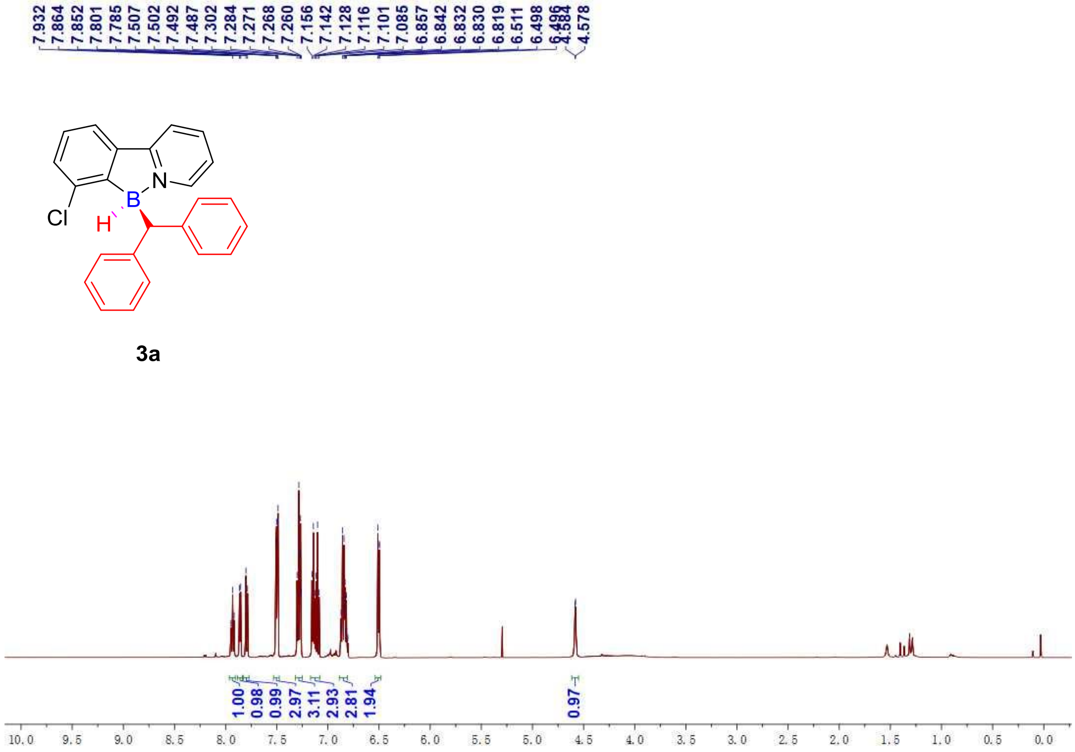

Supplementary Figure 133. ^{1}H NMR spectrum of compound 3a

^{13}C NMR (126 MHz, room temperature, $CDCl_3$)

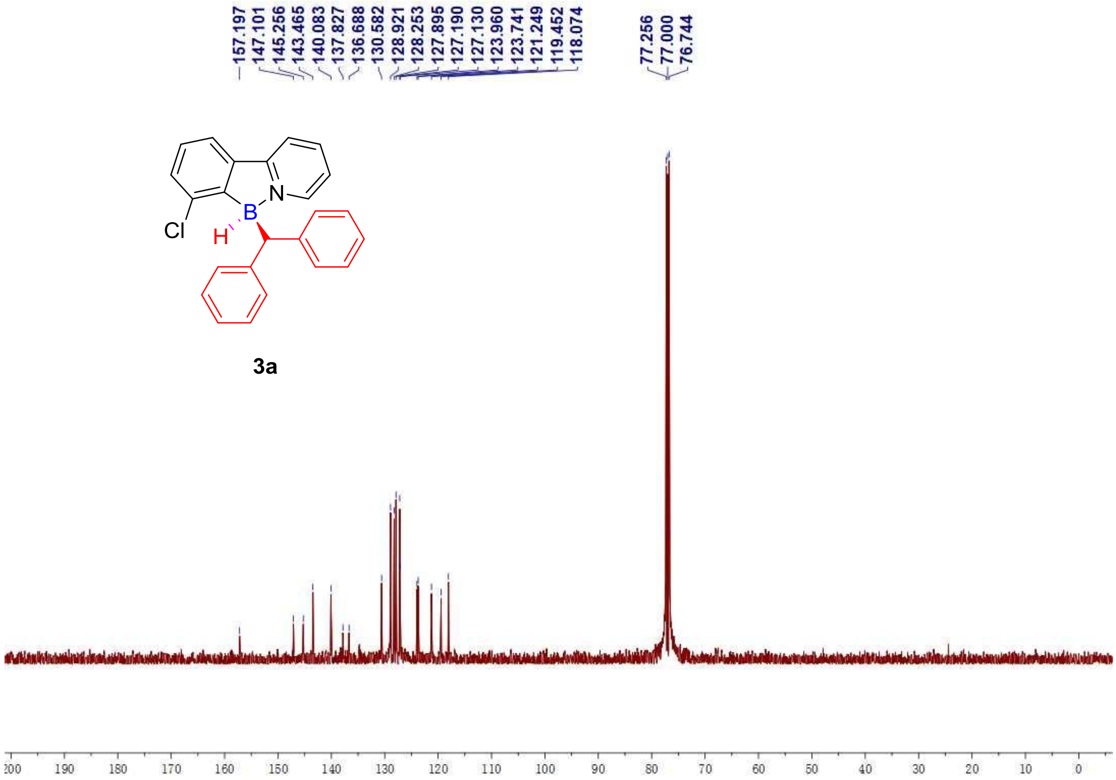

Supplementary Figure 134. ^{13}**C NMR spectrum of compound 3a**

^{11}B NMR (128 MHz, room temperature, $CDCl_3$)

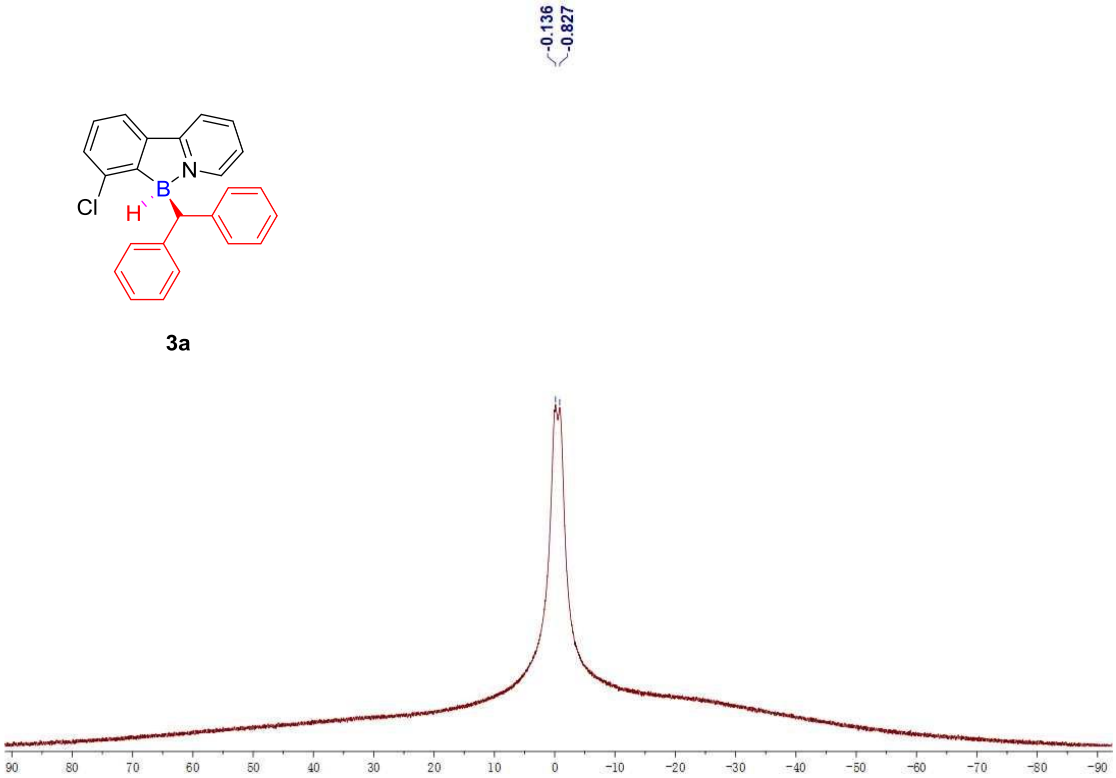

Supplementary Figure 135. ^{11}**B NMR spectrum of compound 3a**

6-(bis(4-fluorophenyl)methyl)-7-chloro-6H-5 ^{4}-benzo[3,4][1,2]azaborolo[1,5-a]pyridine (3b)

^{1}H NMR (400 MHz, room temperature, $CDCl_3$)

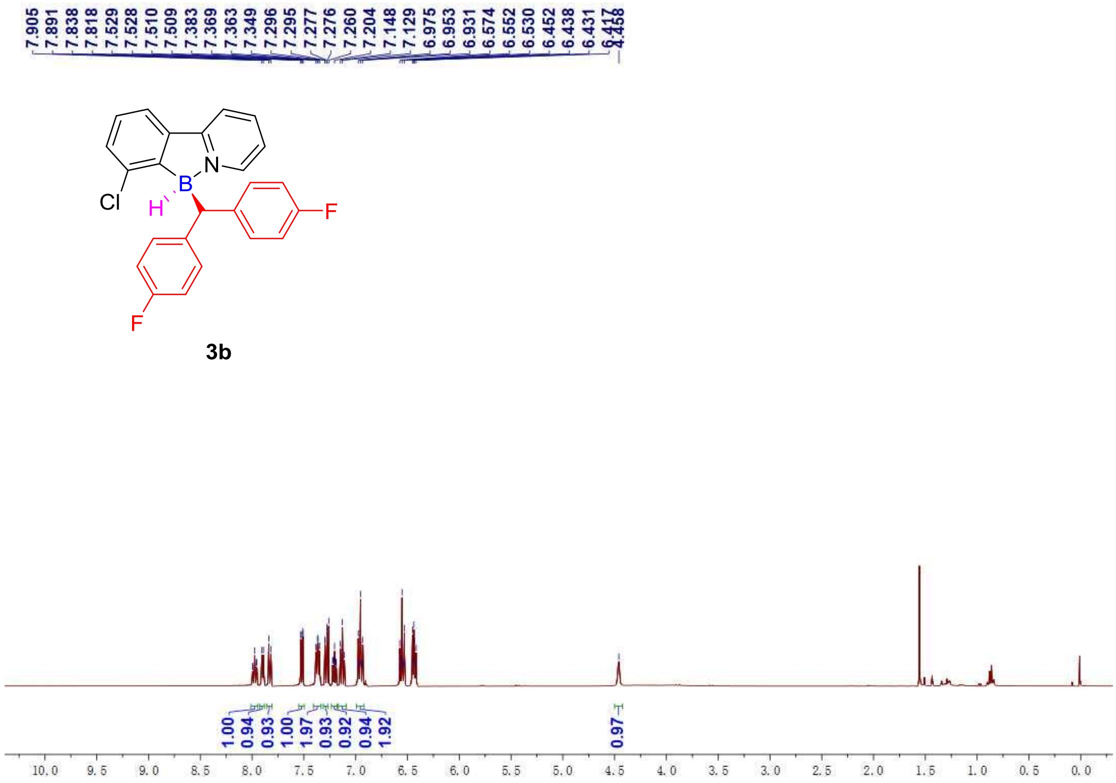

Supplementary Figure 136. ^{1}H NMR spectrum of compound 3b

^{13}C NMR (101 MHz, room temperature, $CDCl_3$)

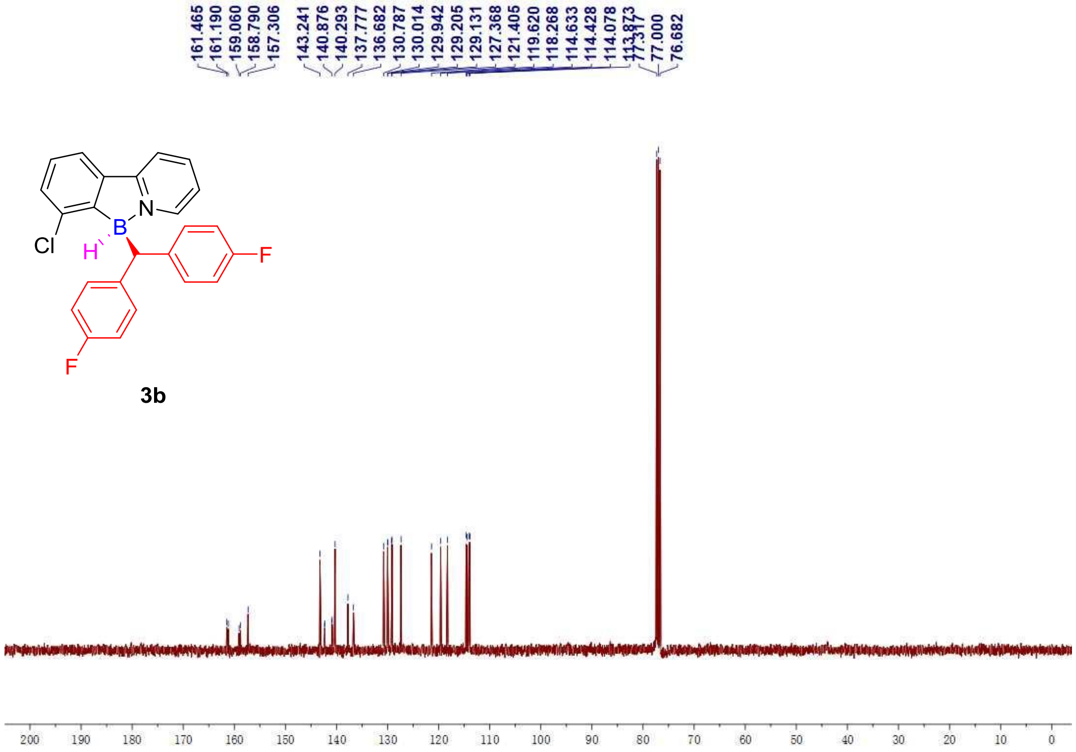

Supplementary Figure 137. ^{13}C NMR spectrum of compound 3b

^{11}B NMR (128 MHz, room temperature, $CDCl_3$)

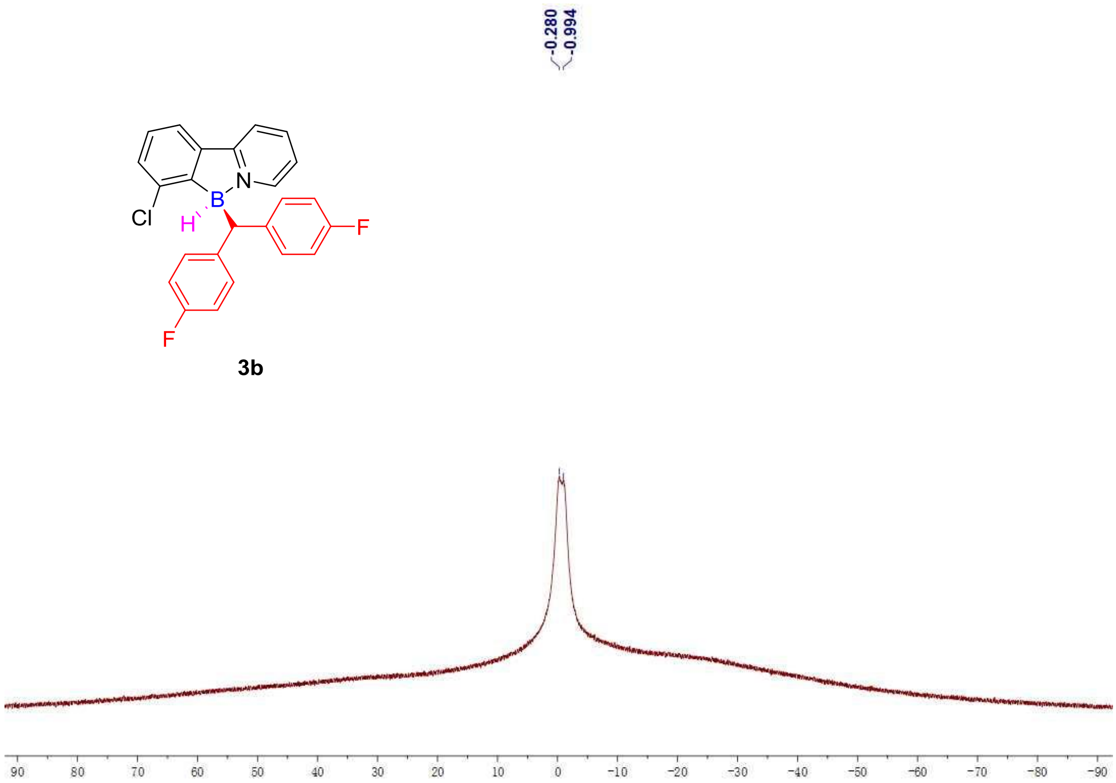

Supplementary Figure 138. ^{11}B NMR spectrum of compound 3b

^{19}F NMR (376 MHz, room temperature, $CDCl_3$)

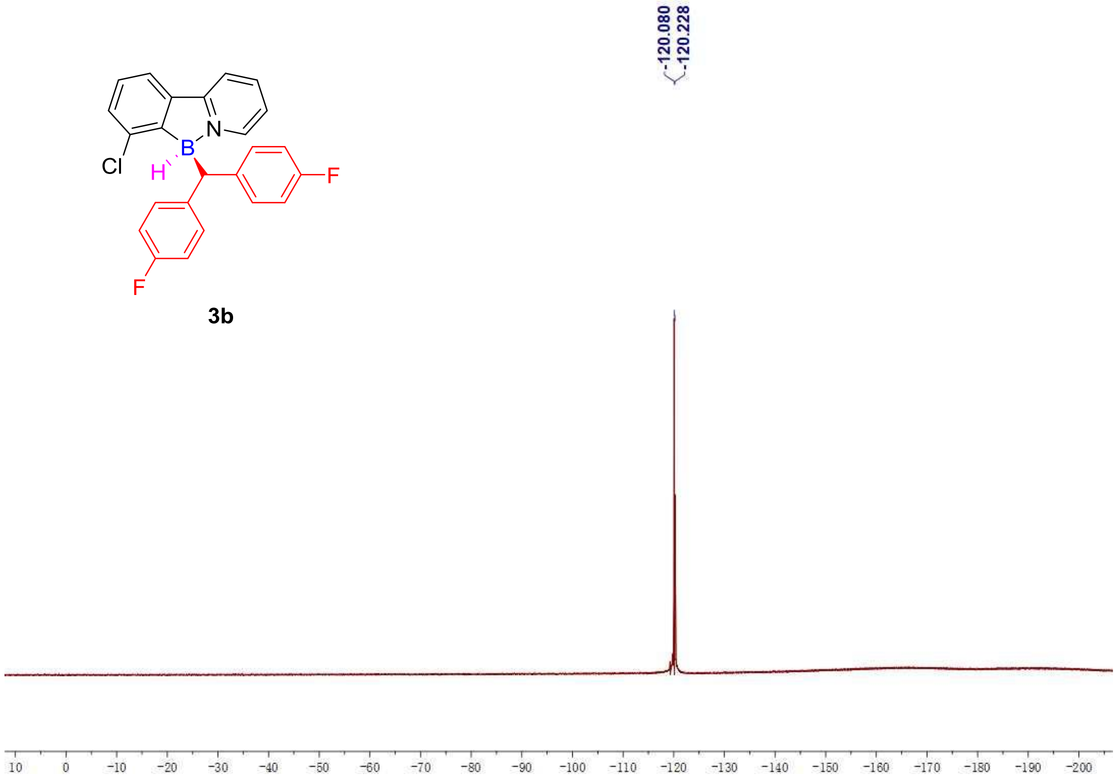

Supplementary Figure 139. ^{19}F NMR spectrum of compound 3b

6-(bis(4-chlorophenyl)methyl)-7-chloro-6H-5 ^{4}-benzo[3,4][1,2]azaborolo[1,5-a]pyridine (3c)

^{1}H NMR (400 MHz, room temperature, $CDCl_3$)

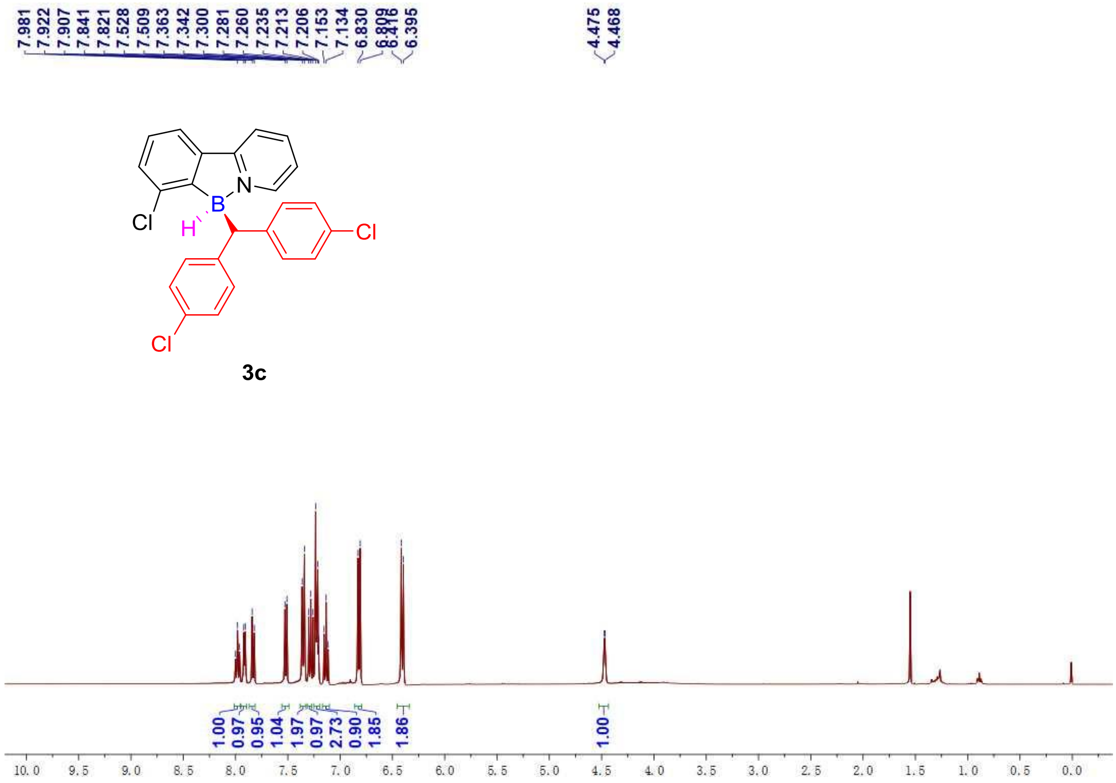

Supplementary Figure 140. ^{1}H NMR spectrum of compound 3c

^{13}C NMR (101 MHz, room temperature, $CDCl_3$)

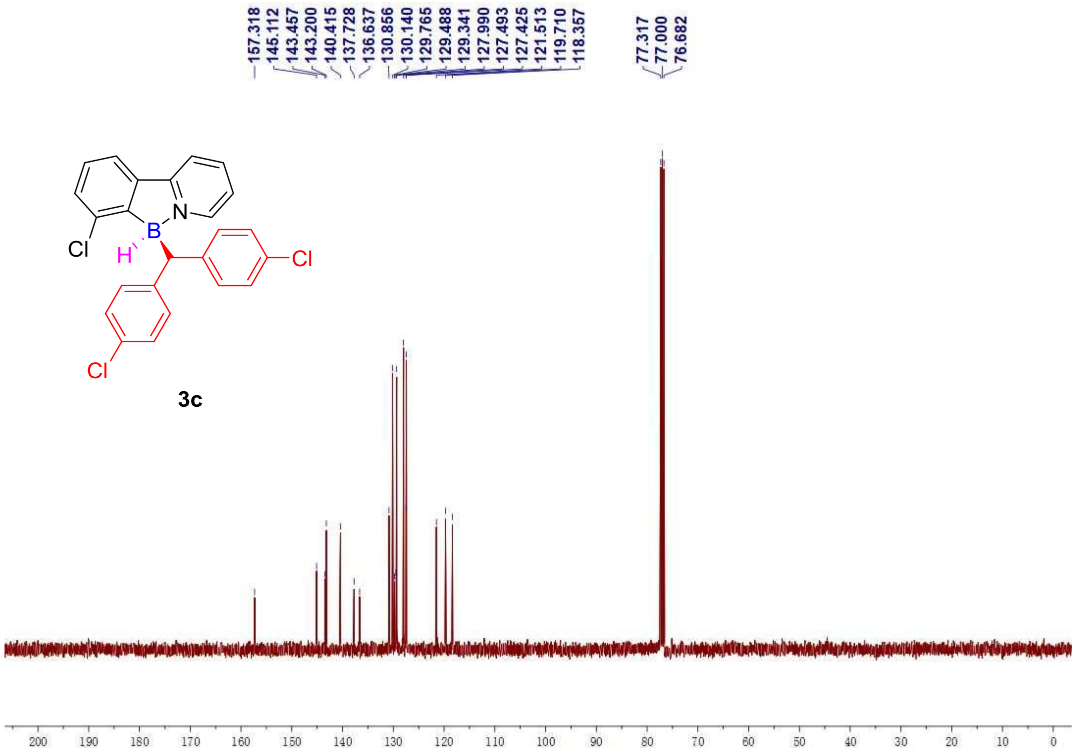

Supplementary Figure 141. ^{13}C NMR spectrum of compound 3c

^{11}B NMR (128 MHz, room temperature, $CDCl_3$)

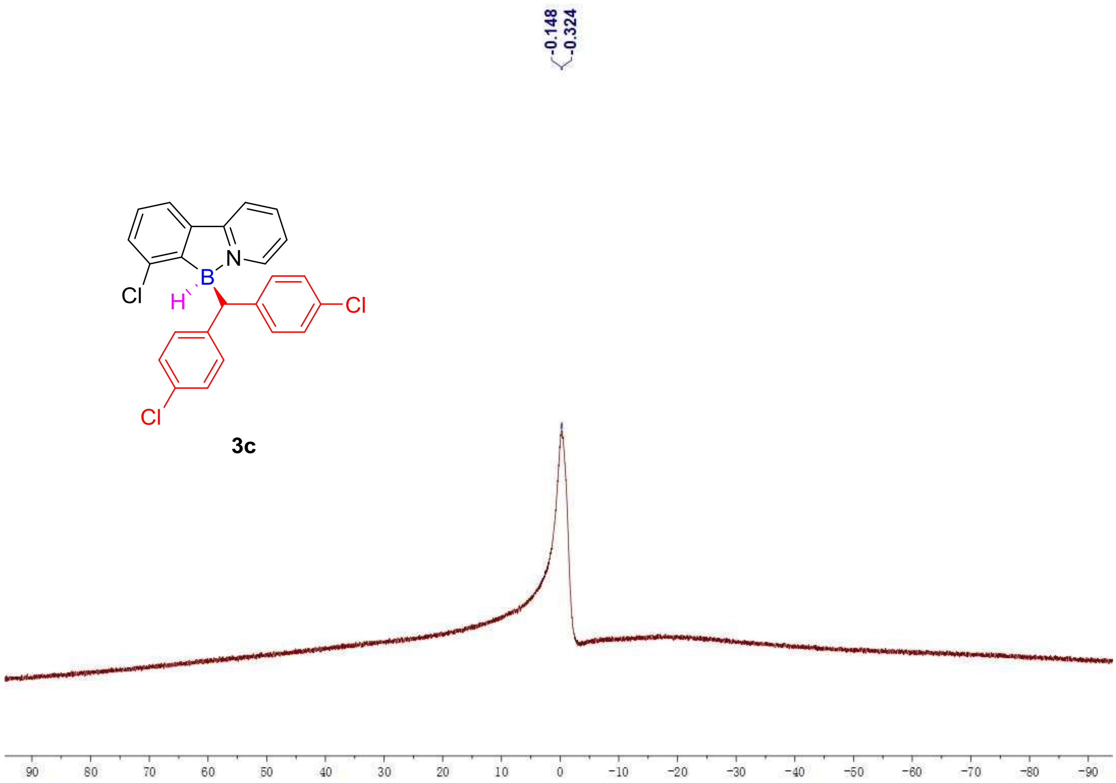

Supplementary Figure 142. ^{11}B NMR spectrum of compound 3c

6-(bis(3-chlorophenyl)methyl)-7-chloro-6H-5 ^{4}-benzo[3,4][1,2]azaborolo[1,5-a]pyridine (3d)

^{1}H NMR (400 MHz, room temperature, $CDCl_3$)

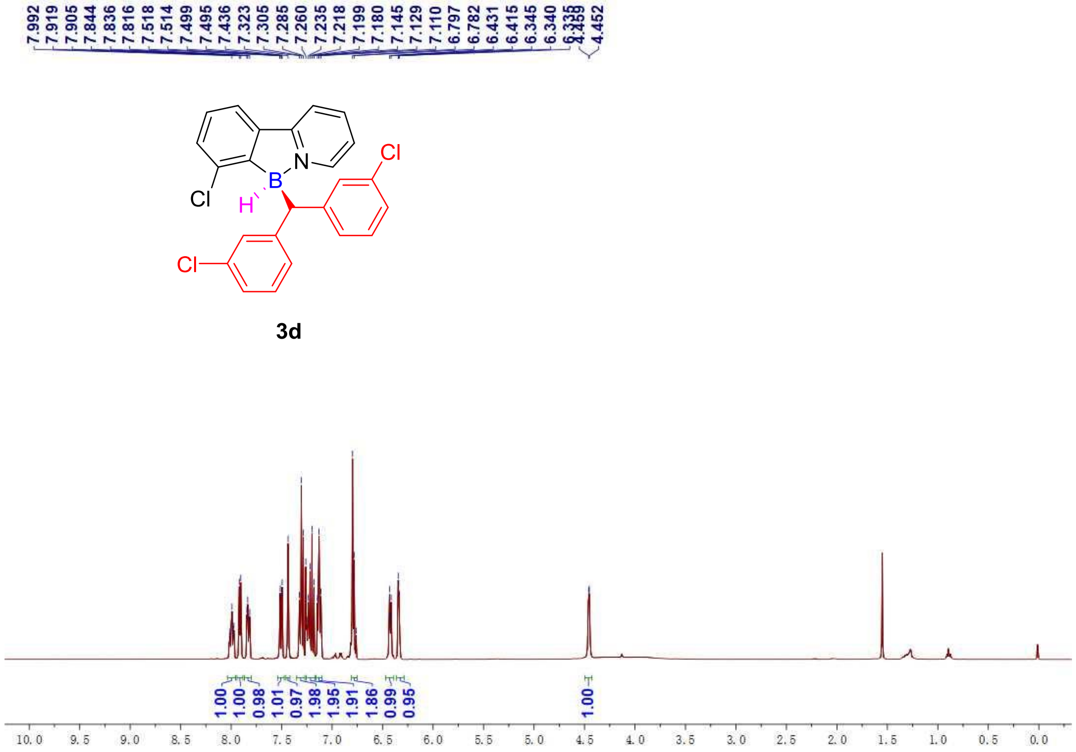

Supplementary Figure 143. ^{1}H NMR spectrum of compound 3d

^{13}C NMR (126 MHz, room temperature, $CDCl_3$)

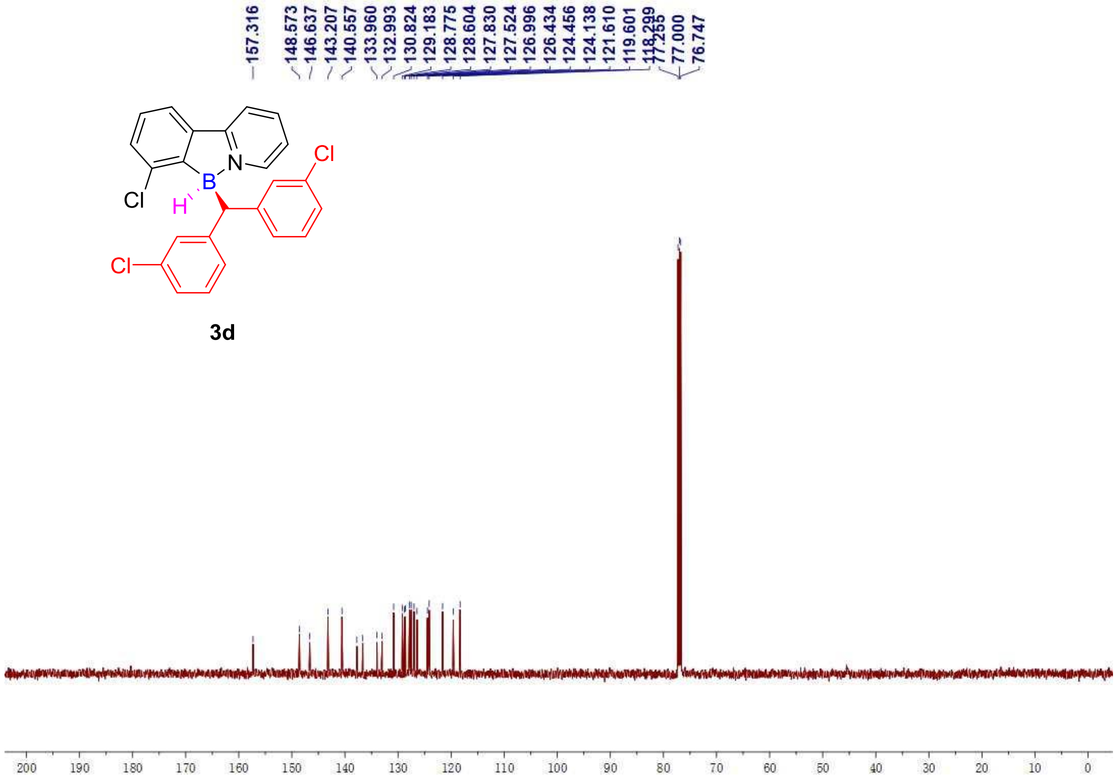

Supplementary Figure 144. ^{13}C NMR spectrum of compound 3d

^{11}B NMR (128 MHz, room temperature, $CDCl_3$)

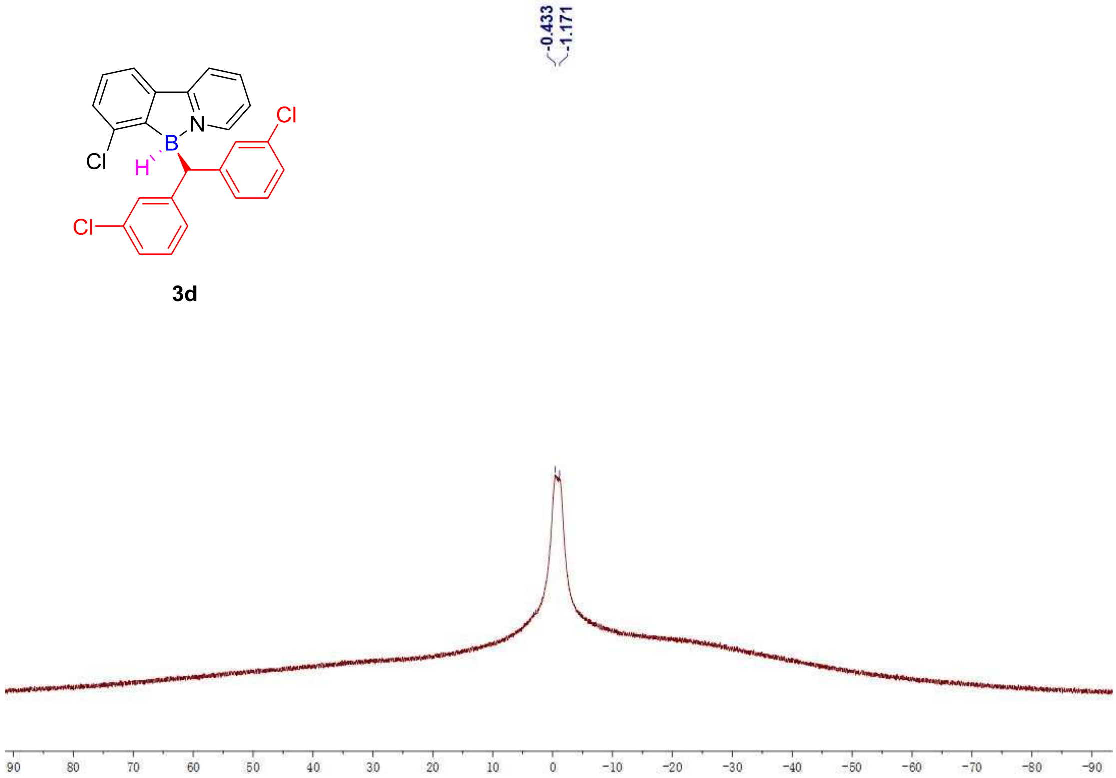

Supplementary Figure 145. ^{11}B NMR spectrum of compound 3d

6-(bis(4-methoxyphenyl)methyl)-7-chloro-6H-5 ^{4}-benzo[3,4][1,2]azaborolo[1,5-a]pyridine (3e)

^{1}H NMR (500 MHz, room temperature, CDCl$_3$)

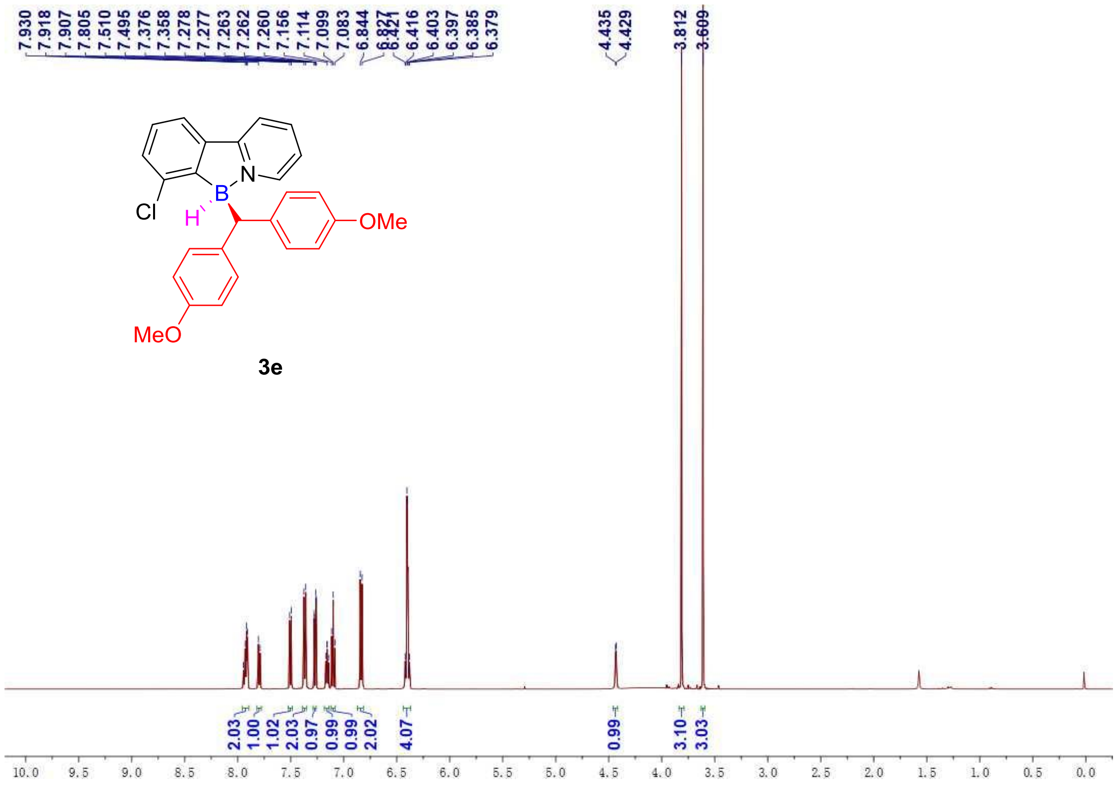

Supplementary Figure 146. ^{1}H NMR spectrum of compound 3e

^{13}C NMR (101 MHz, room temperature, CDCl$_3$)

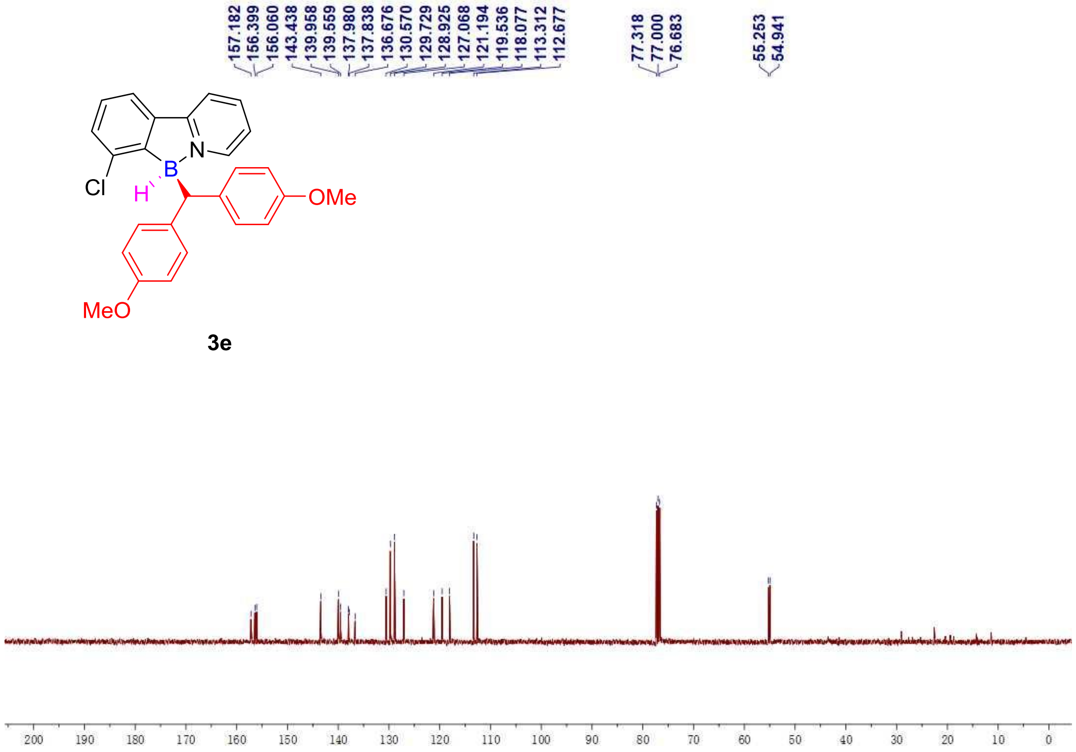

Supplementary Figure 147. ^{13}C NMR spectrum of compound 3e

^{11}B NMR (128 MHz, room temperature, $CDCl_3$)

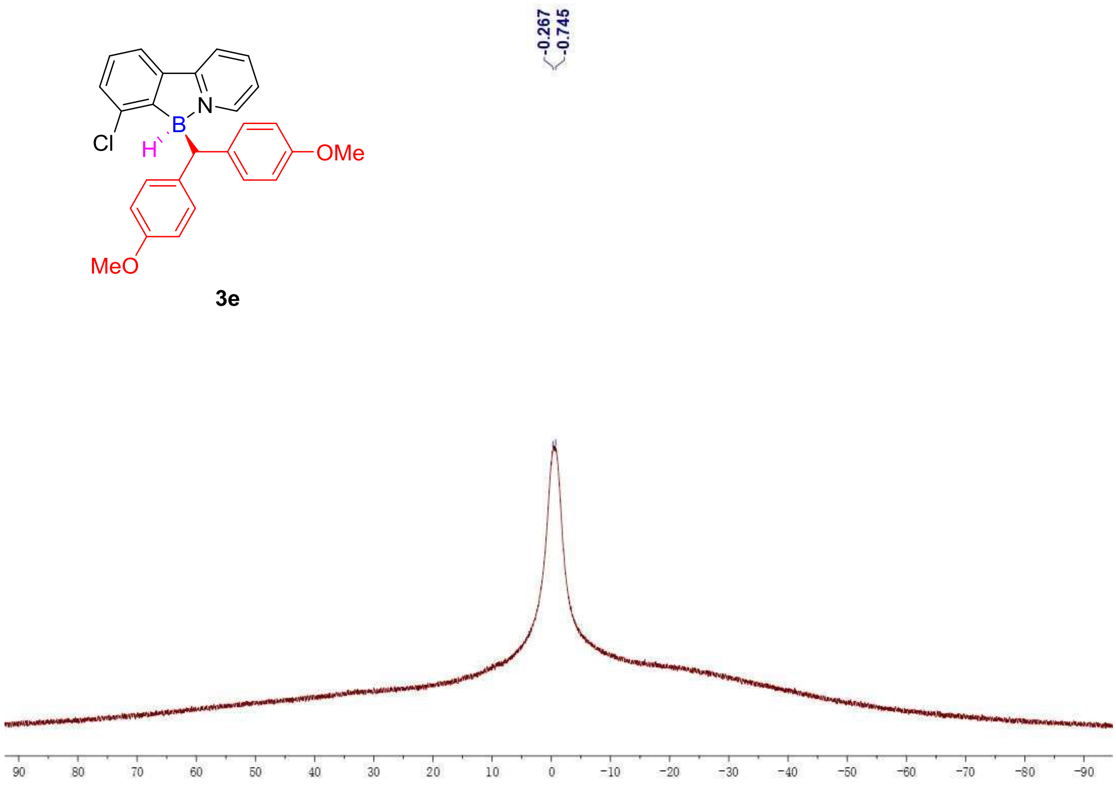

Supplementary Figure 148. ^{11}B NMR spectrum of compound 3e

6-(bis(3-methoxyphenyl)methyl)-7-chloro-6H-5 ^{4}-benzo[3,4][1,2]azaborolo[1,5-a]pyridine (3f)

^{1}H NMR (500 MHz, room temperature, $CDCl_3$)

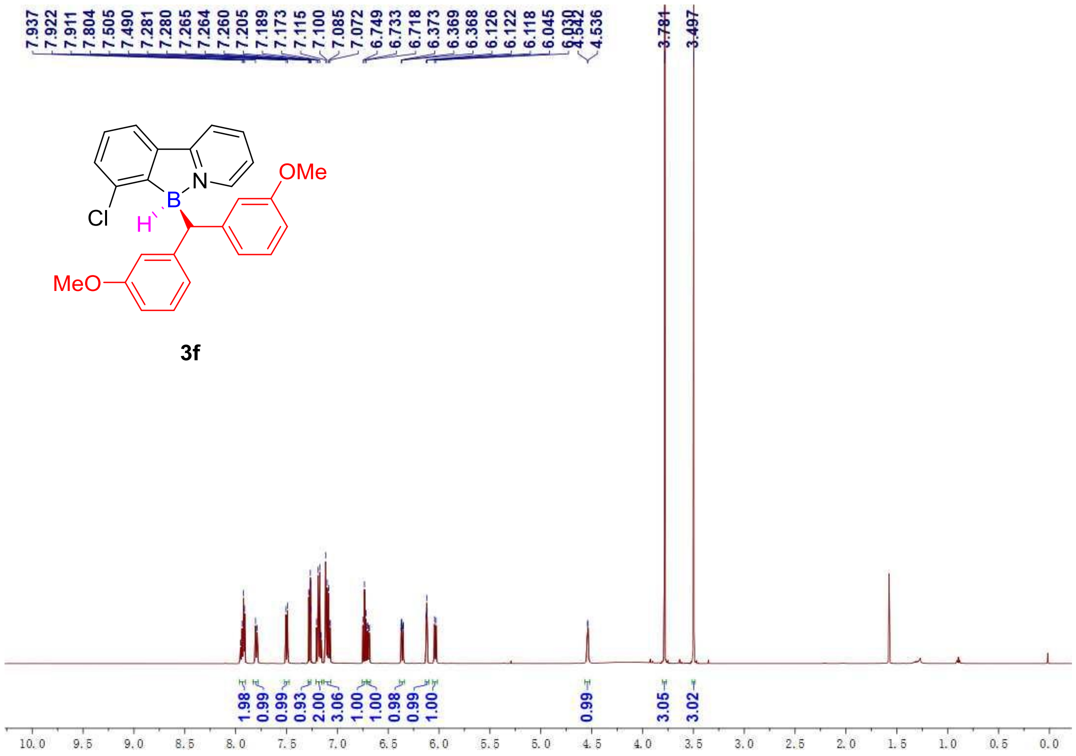

Supplementary Figure 149. ^{1}H NMR spectrum of compound 3f

^{13}C NMR (101 MHz, room temperature, $CDCl_3$)

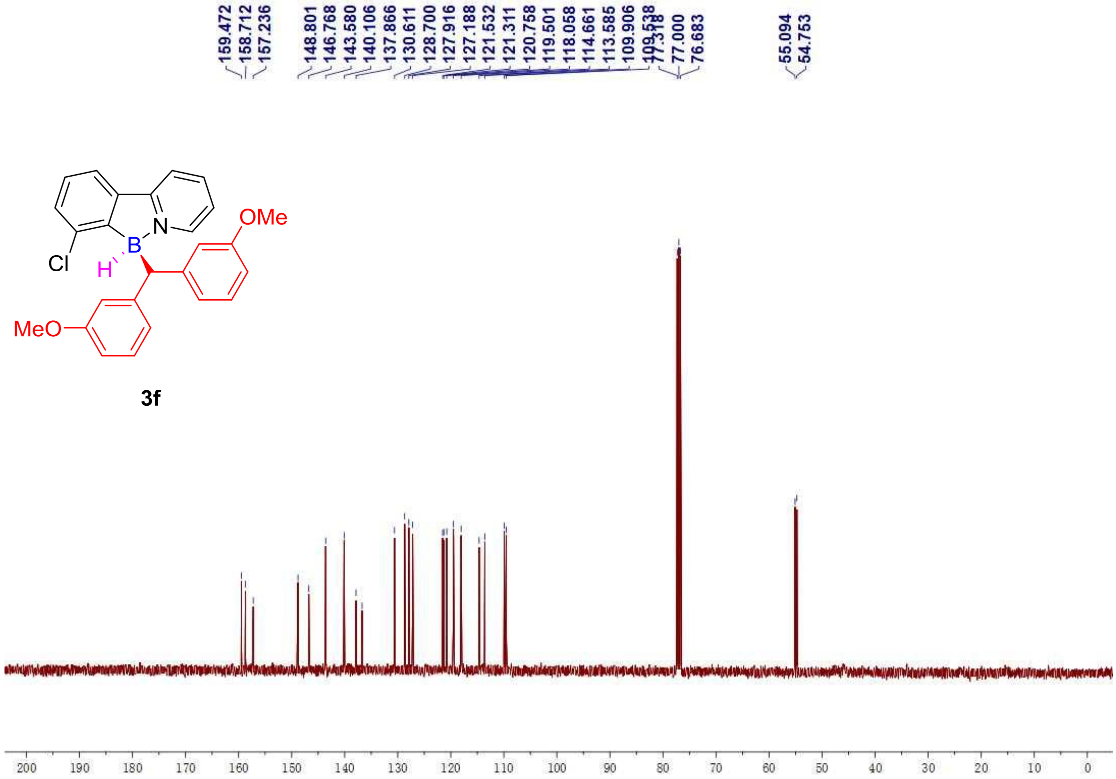

3f

Supplementary Figure 150. ^{13}**C NMR spectrum of compound 3f**

^{11}B NMR (128 MHz, room temperature, $CDCl_3$)

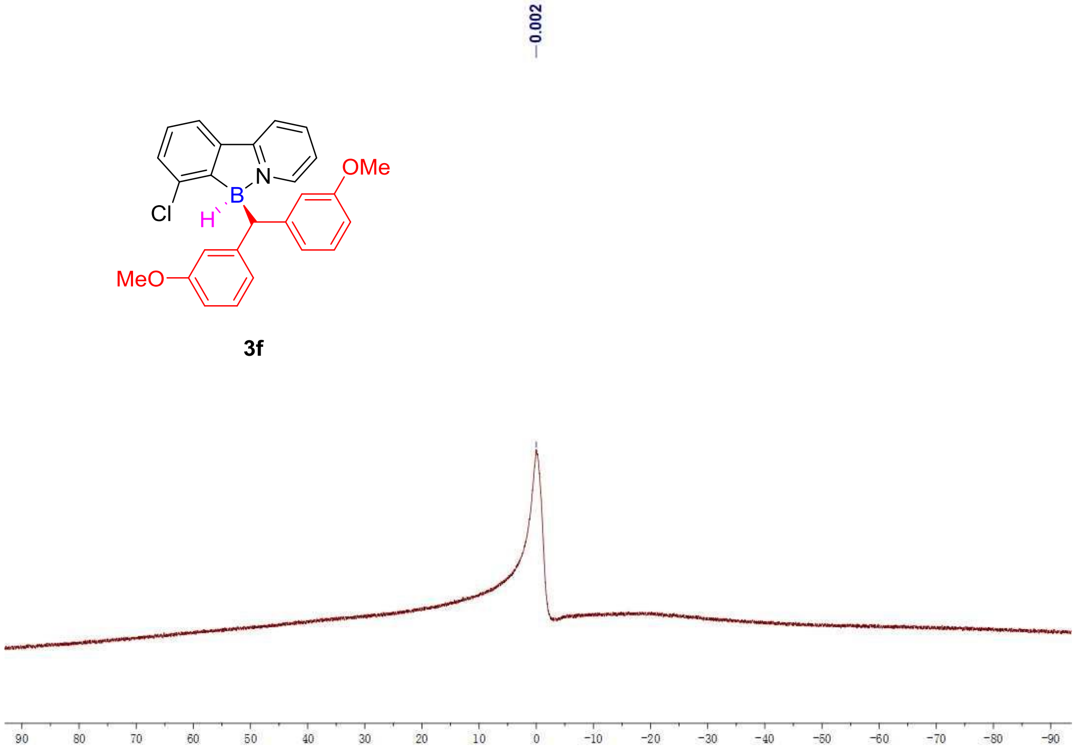

3f

Supplementary Figure 151. ^{11}**B NMR spectrum of compound 3f**

7-chloro-6-(di-m-tolylmethyl)-6H-5 ^{4}-benzo[3,4][1,2]azaborolo[1,5-a]pyridine (3g)

^{1}H NMR (500 MHz, room temperature, CDCl$_3$)

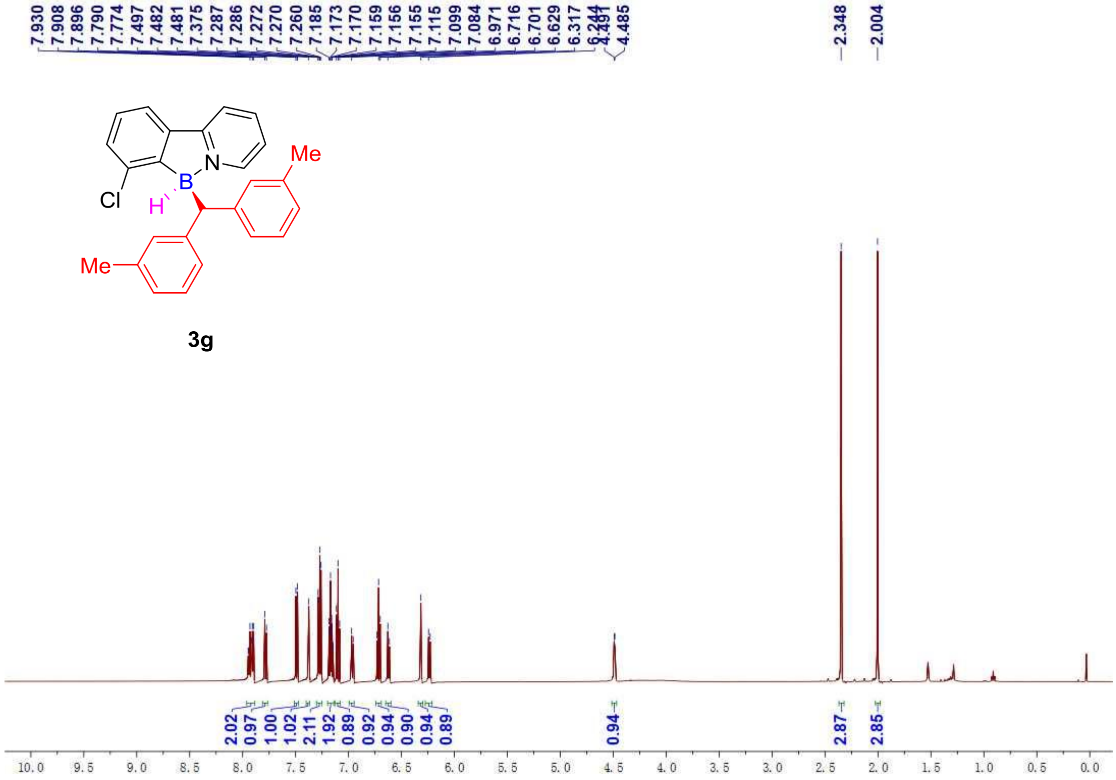

Supplementary Figure 152. ^{1}H NMR spectrum of compound 3g

^{13}C NMR (126 MHz, room temperature, CDCl$_3$)

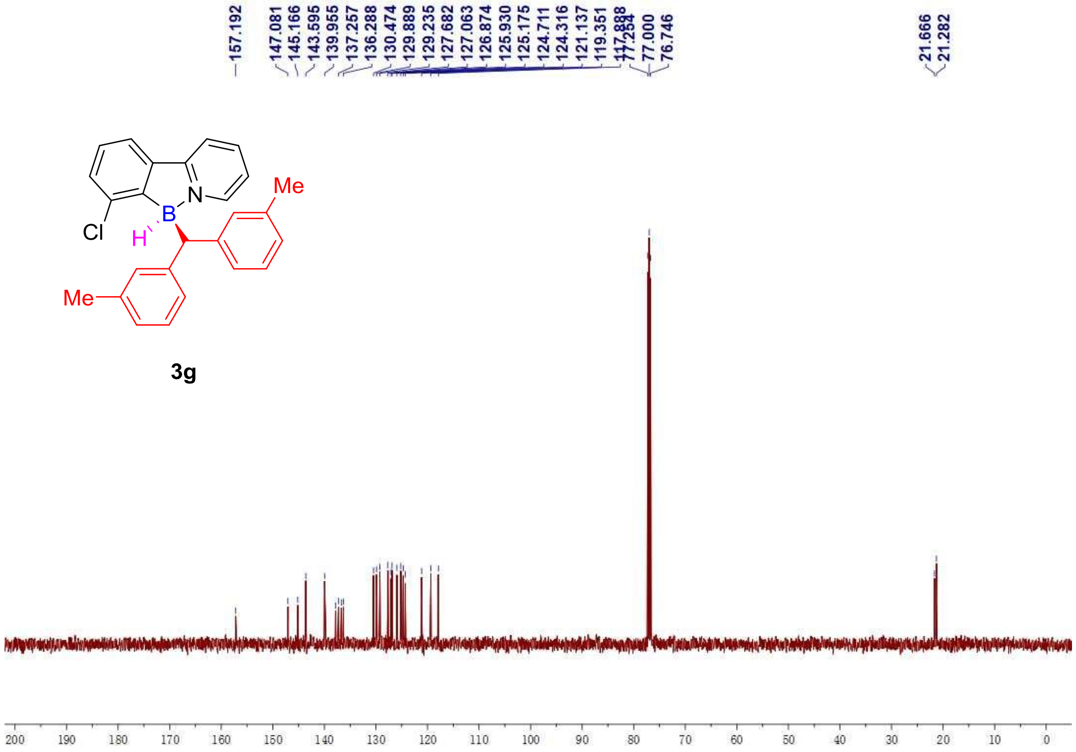

Supplementary Figure 153. ^{13}C NMR spectrum of compound 3g

^{11}B NMR (128 MHz, room temperature, $CDCl_3$)

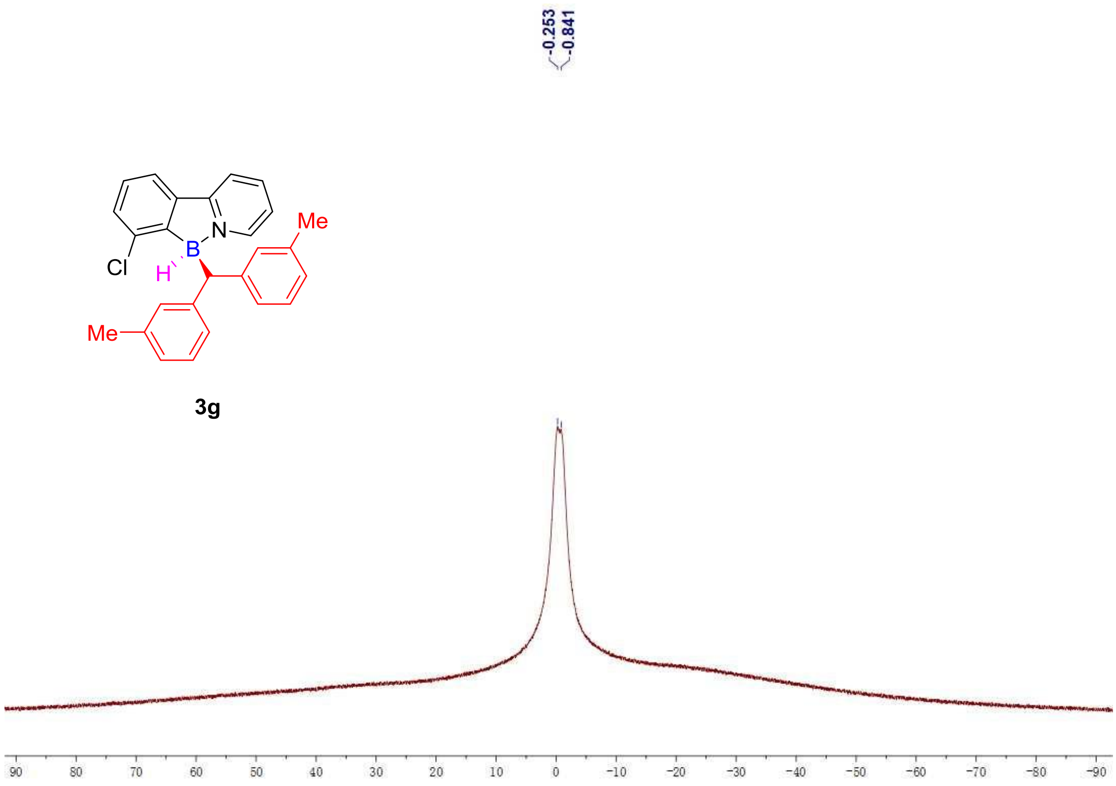

Supplementary Figure 154. **^{11}B NMR spectrum of compound 3g**

7-chloro-6-(2,7-dibromo-9H-fluoren-9-yl)-6H-5 ^{4}-benzo[3,4][1,2]azaborolo[1,5-a]pyridine (3h)

^{1}H NMR (400 MHz, room temperature, $CDCl_3$)

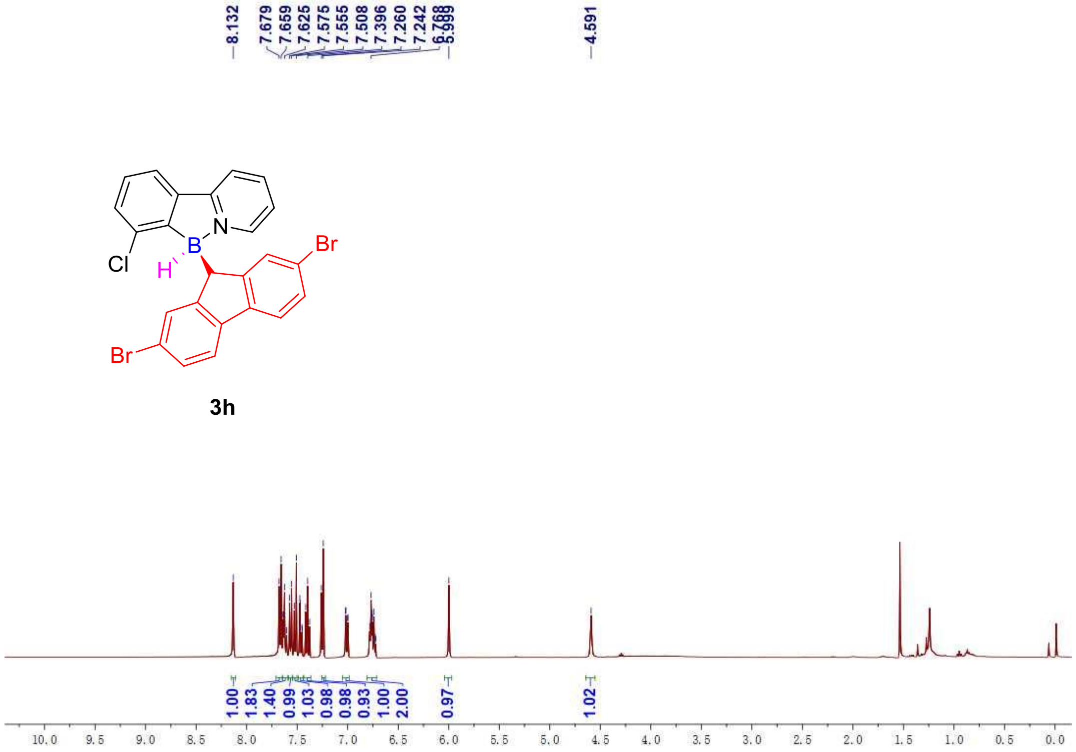

Supplementary Figure 155. **^{1}H NMR spectrum of compound 3h**

^{13}C NMR (101 MHz, room temperature, $CDCl_3$)

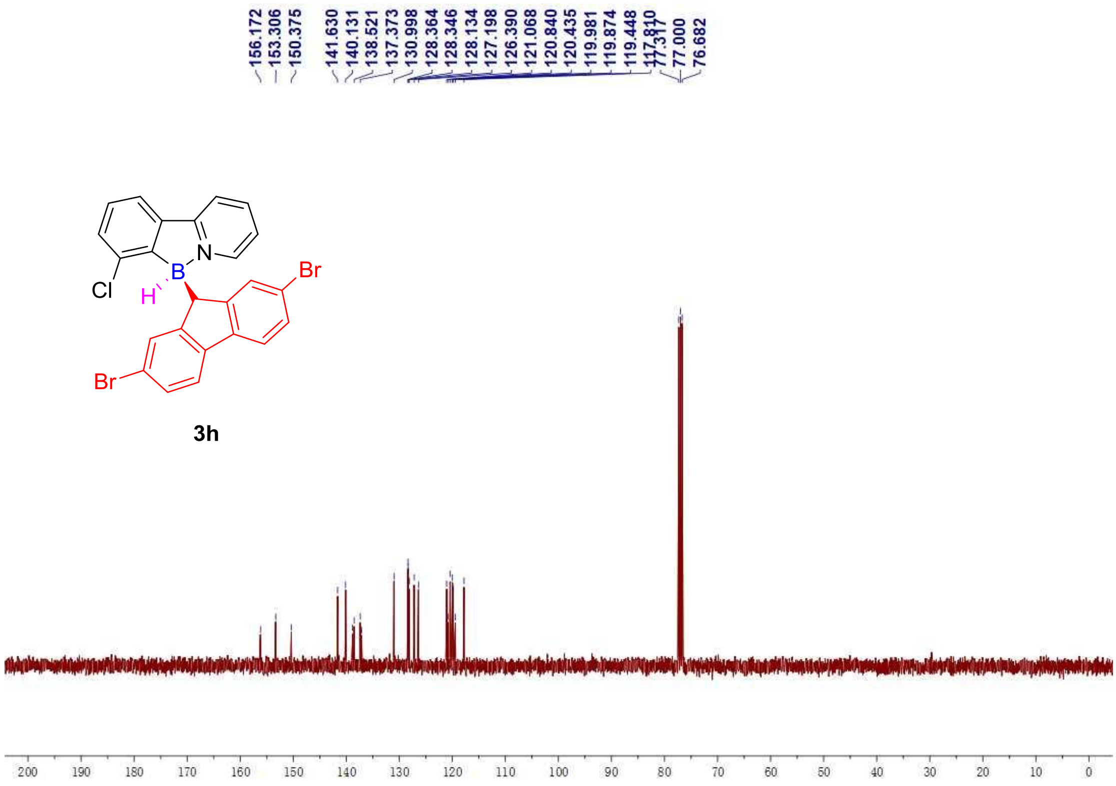

Supplementary Figure 156. ^{13}C NMR spectrum of compound 3h

^{11}B NMR (128 MHz, room temperature, $CDCl_3$)

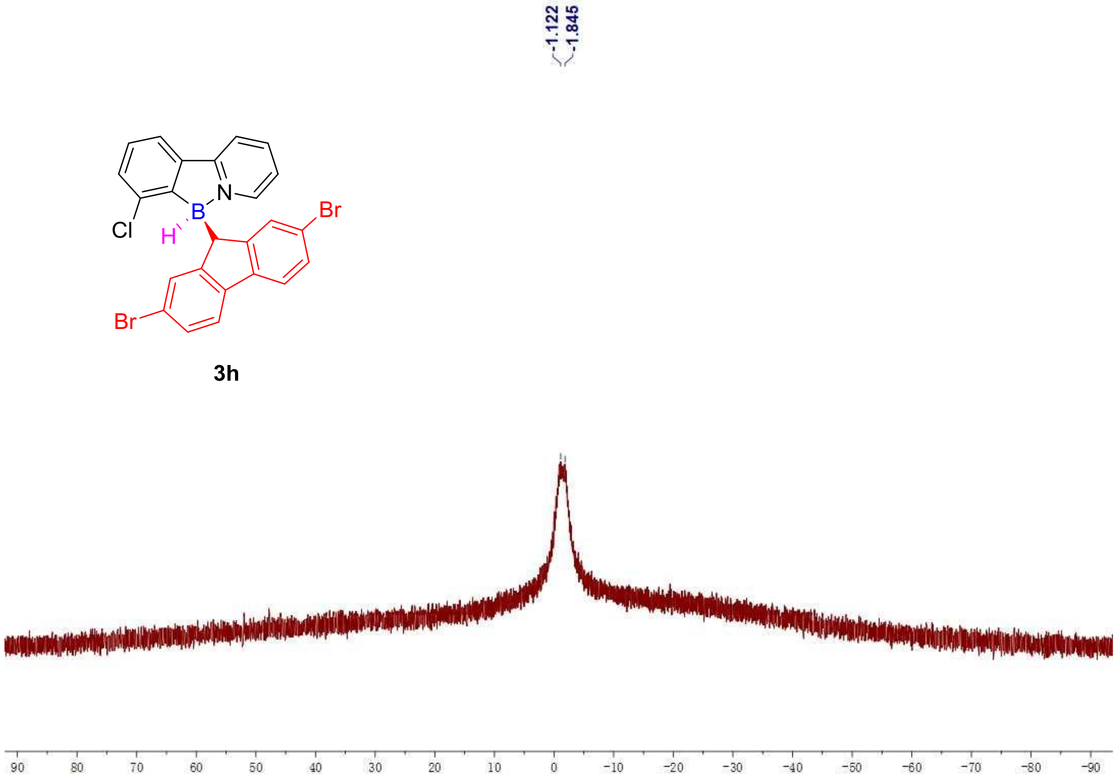

Supplementary Figure 157. ^{11}B NMR spectrum of compound 3h

7-chloro-6-(9H-fluoren-9-yl)-6H-5 ^{4}-benzo[3,4][1,2]azaborolo[1,5-a]pyridine (3i)

^{1}H NMR (400 MHz, room temperature, $CDCl_3$)

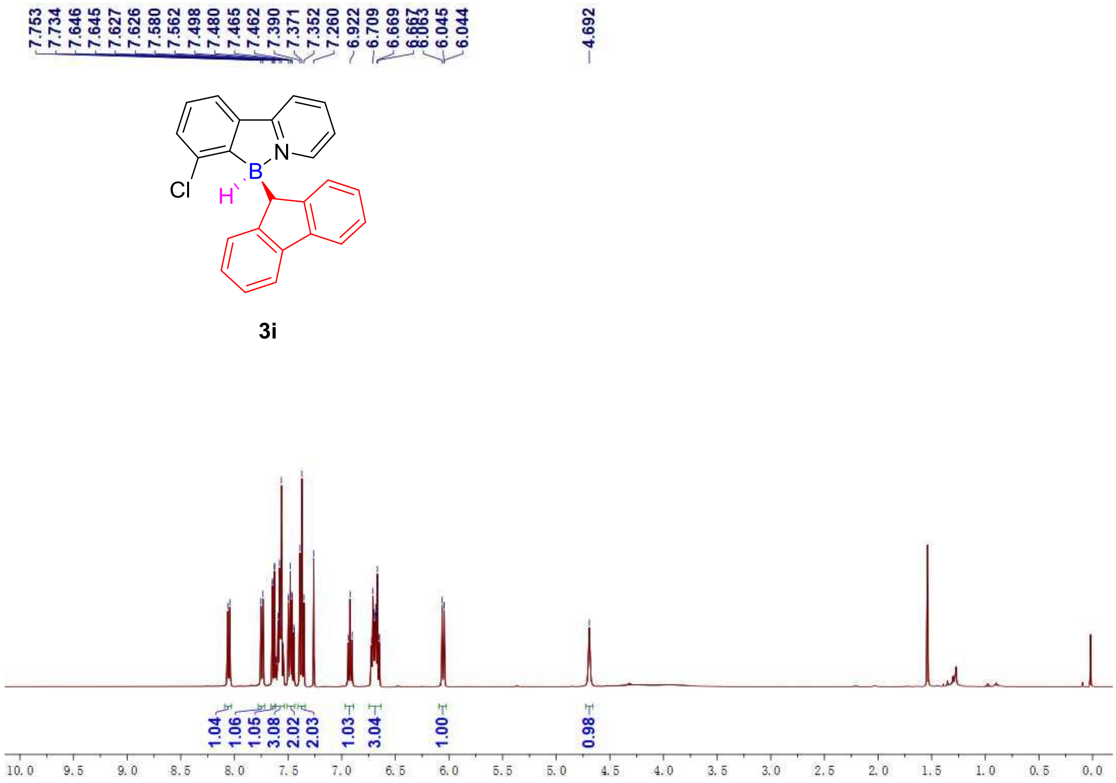

Supplementary Figure 158. ^{1}H NMR spectrum of compound 3i

^{13}C NMR (101 MHz, room temperature, $CDCl_3$)

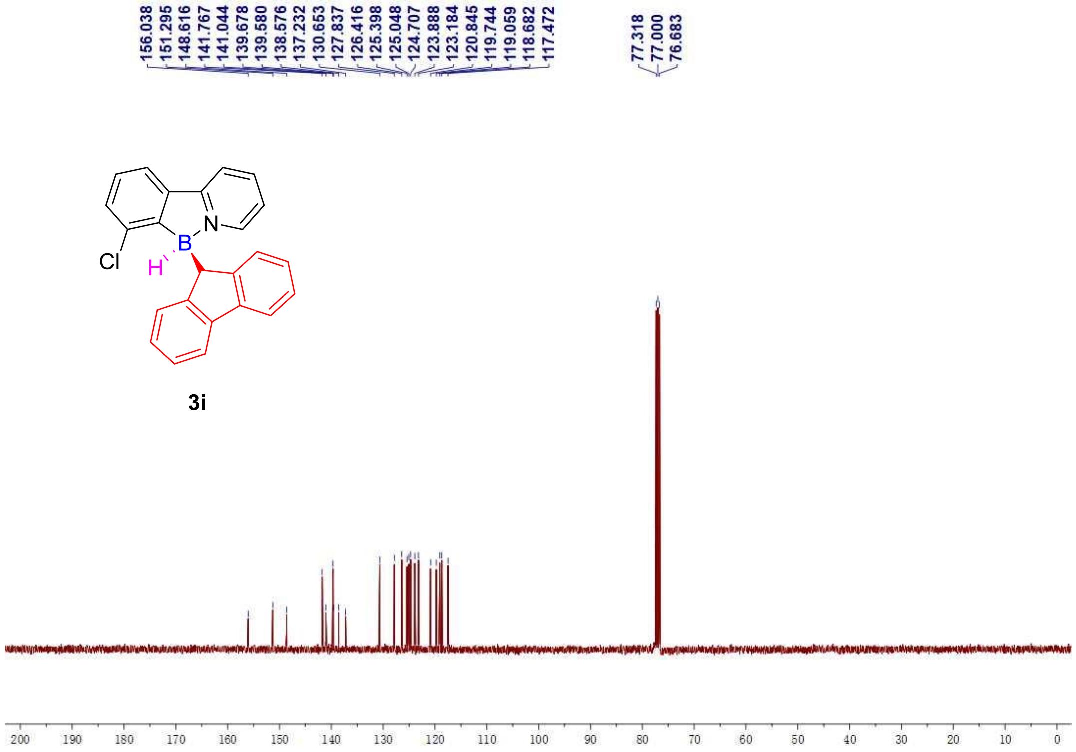

Supplementary Figure 159. ^{13}C NMR spectrum of compound 3i

^{11}B NMR (128 MHz, room temperature, $CDCl_3$)

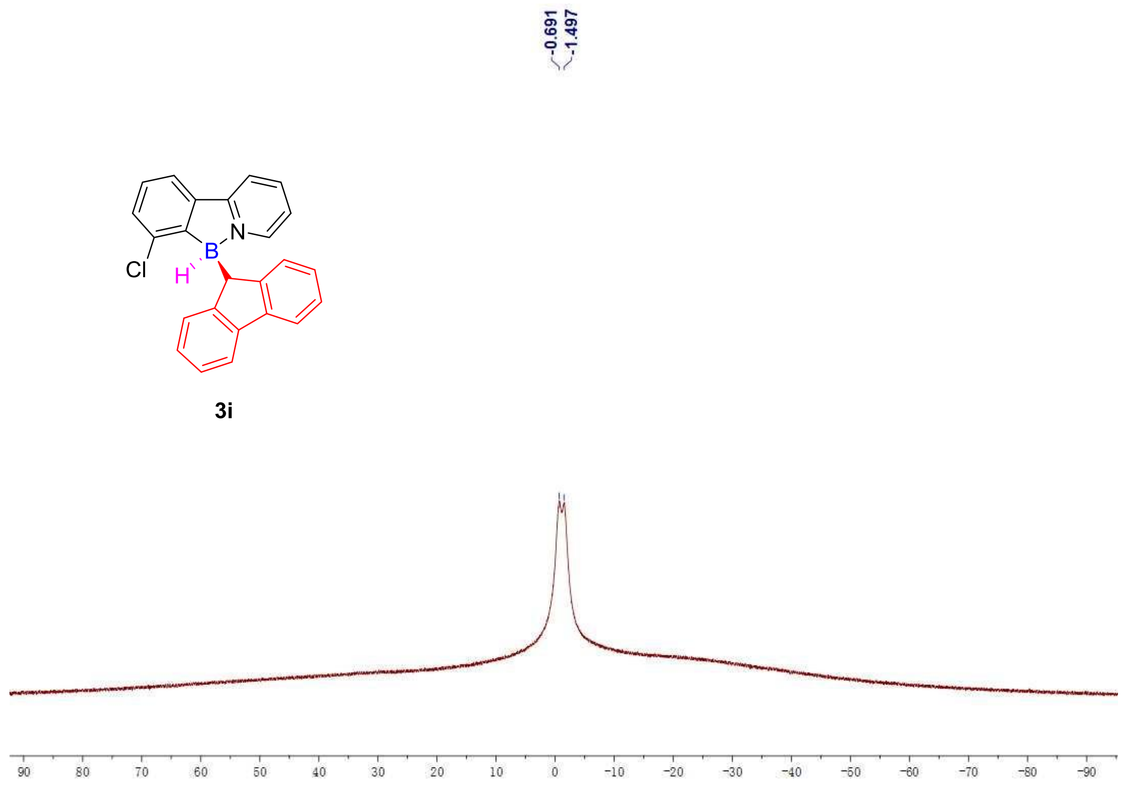

Supplementary Figure 160. ^{11}B NMR spectrum of compound 3i

7-chloro-6-(9H-thioxanthen-9-yl)-6H-5 ^{4}-benzo[3,4][1,2]azaborolo[1,5-a]pyridine (3j)

^{1}H NMR (400 MHz, room temperature, $CDCl_3$)

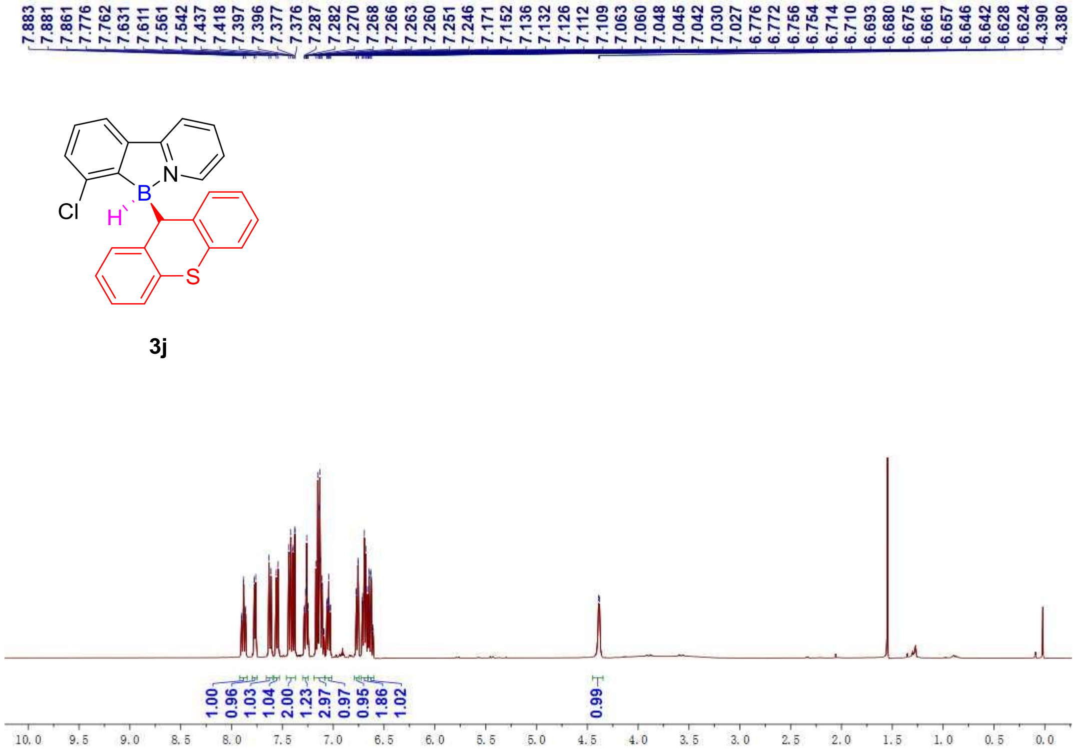

Supplementary Figure 161. ^{1}H NMR spectrum of compound 3j

^{13}C NMR (101 MHz, room temperature, CDCl$_3$)

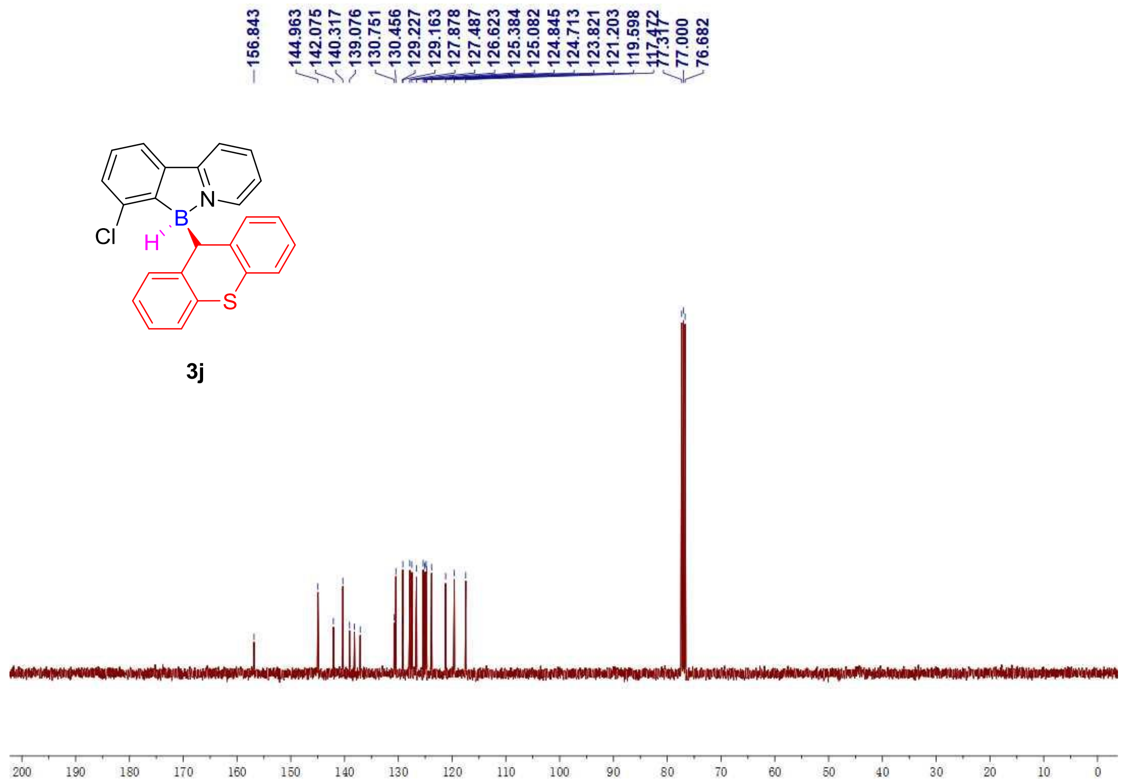

Supplementary Figure 162. ^{13}C NMR spectrum of compound 3j

^{11}B NMR (128 MHz, room temperature, CDCl$_3$)

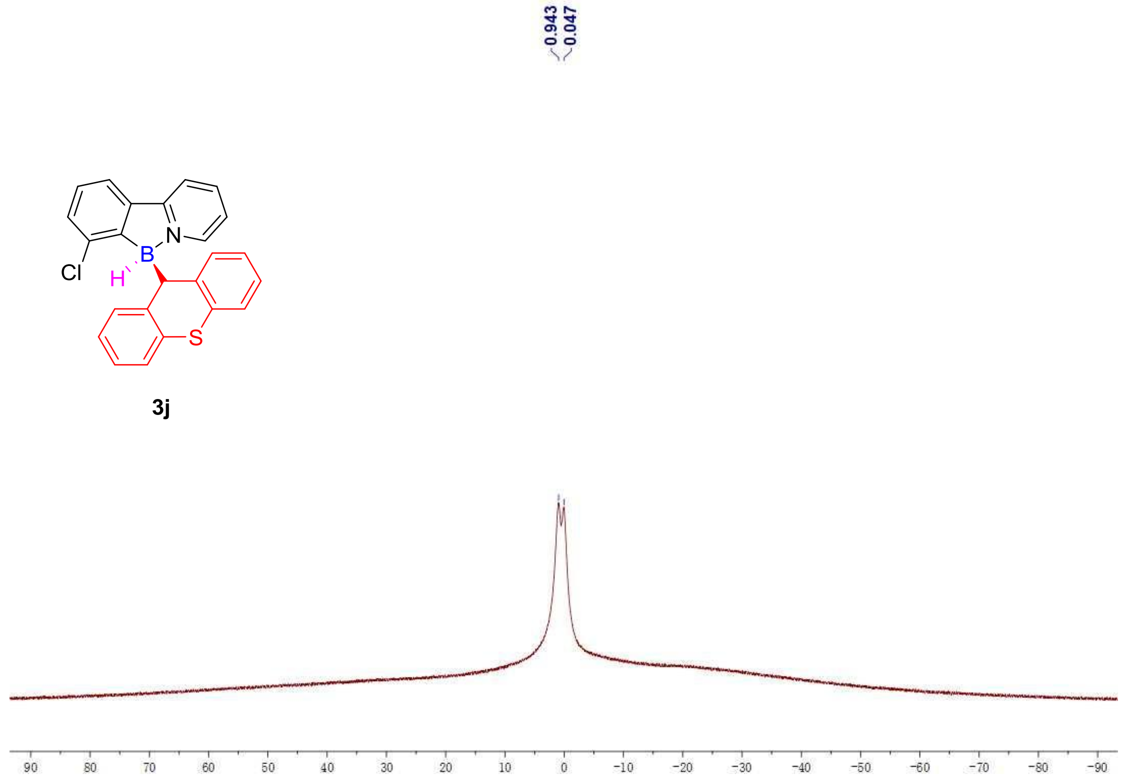

Supplementary Figure 163. ^{11}B NMR spectrum of compound 3j

10-(7-chloro-6H-5 ^{4}-benzo[3,4][1,2]azaborolo[1,5-a]pyridin-6-yl)anthracen-9(10H)-one (3k)

^{1}H NMR (500 MHz, room temperature, $CDCl_3$)

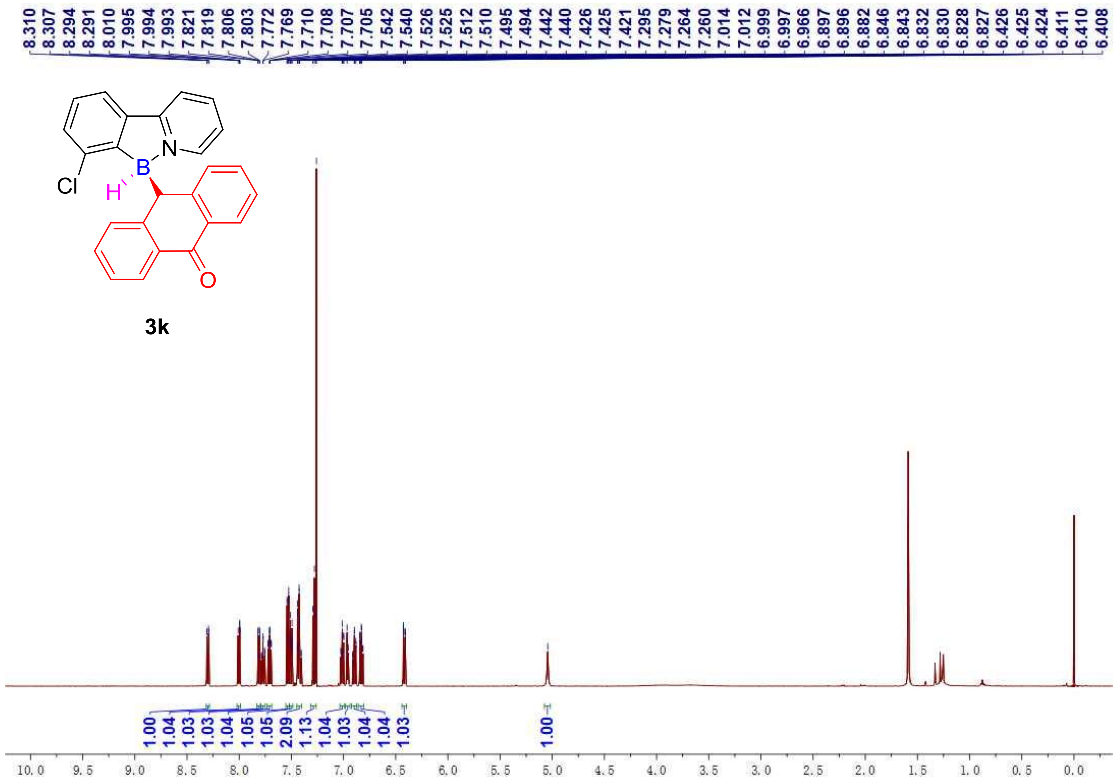

Supplementary Figure 164. ^{1}H NMR spectrum of compound 3k

^{13}C NMR (126 MHz, room temperature, $CDCl_3$)

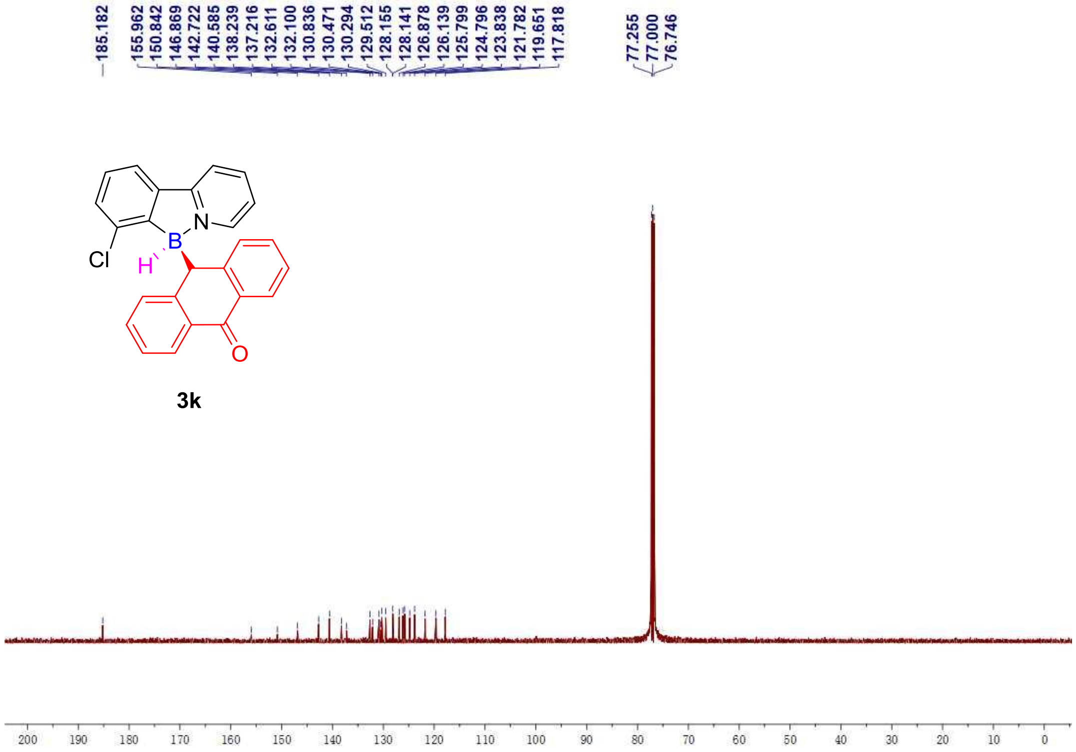

Supplementary Figure 165. ^{13}C NMR spectrum of compound 3k

^{11}B NMR (128 MHz, room temperature, $CDCl_3$)

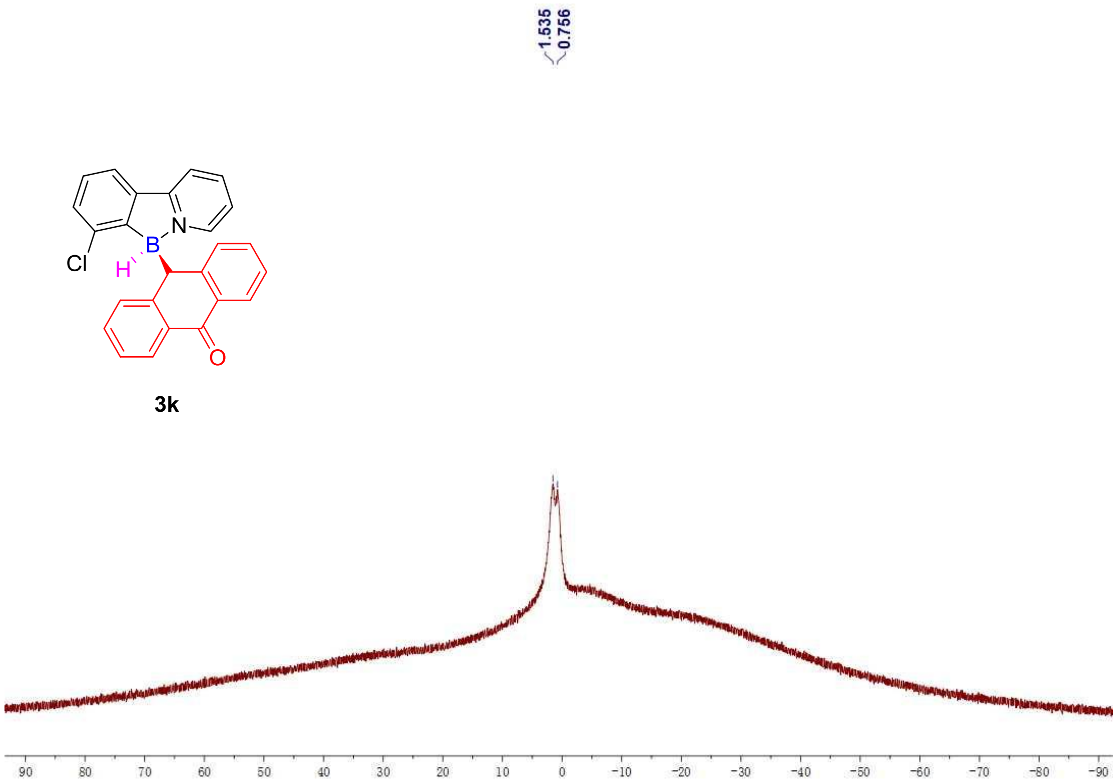

Supplementary Figure 166. ^{11}B NMR spectrum of compound 3k

6-benzhydryl-7-fluoro-6H-5 ^{4}-benzo[3,4][1,2]azaborolo[1,5-a]pyridine (3l)

^{1}H NMR (500 MHz, room temperature, $CDCl_3$)

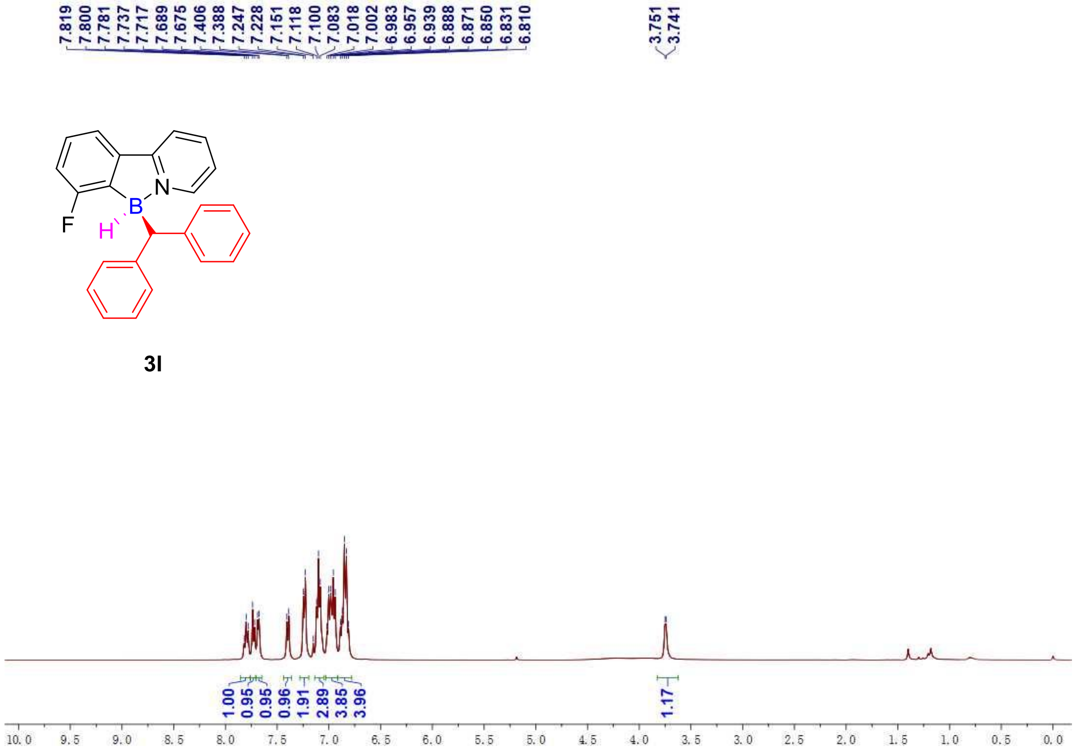

Supplementary Figure 167. ^{1}H NMR spectrum of compound 3l

^{13}C NMR (101 MHz, room temperature, $CDCl_3$)

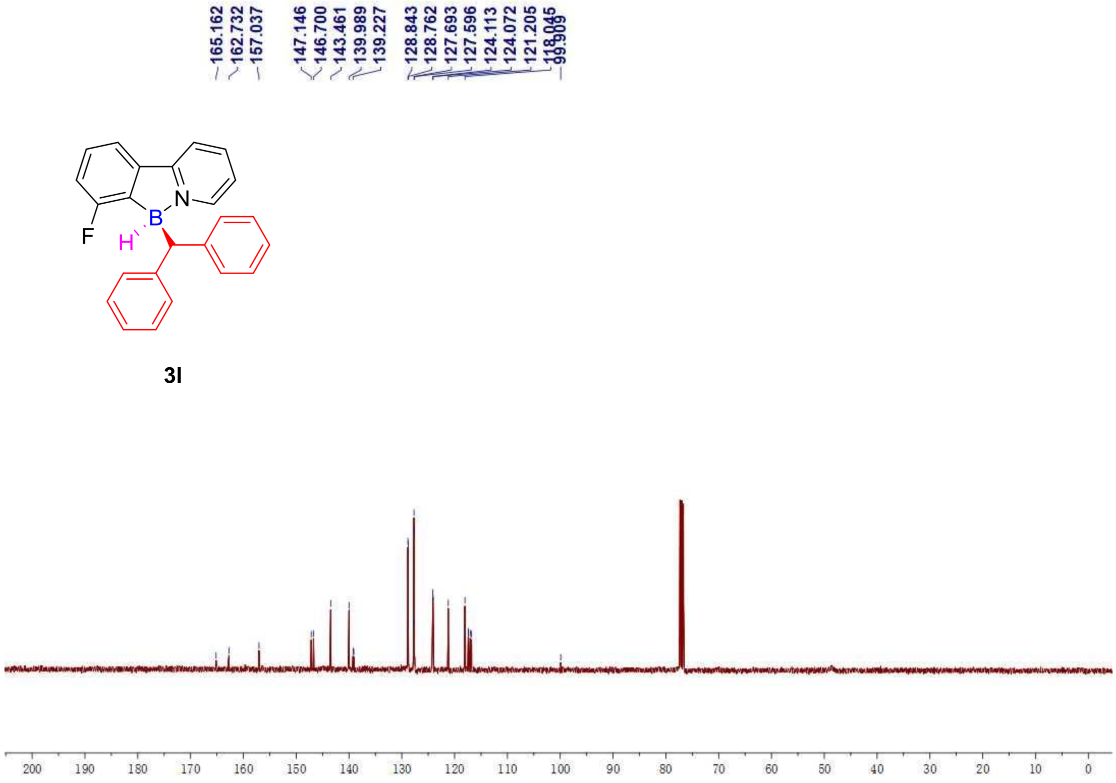

Supplementary Figure 168. ^{13}**C NMR spectrum of compound 3l**

^{11}B NMR (128 MHz, room temperature, $CDCl_3$)

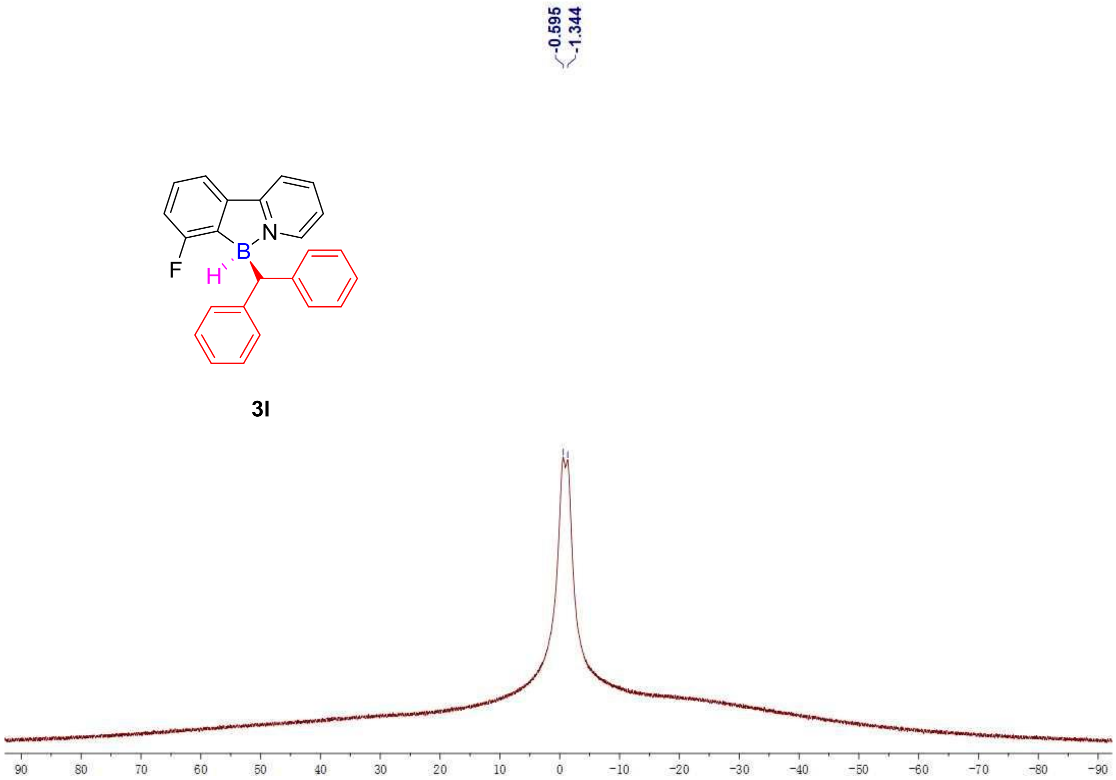

Supplementary Figure 169. ^{11}**B NMR spectrum of compound 3l**

^{19}F NMR (376 MHz, room temperature, $CDCl_3$)

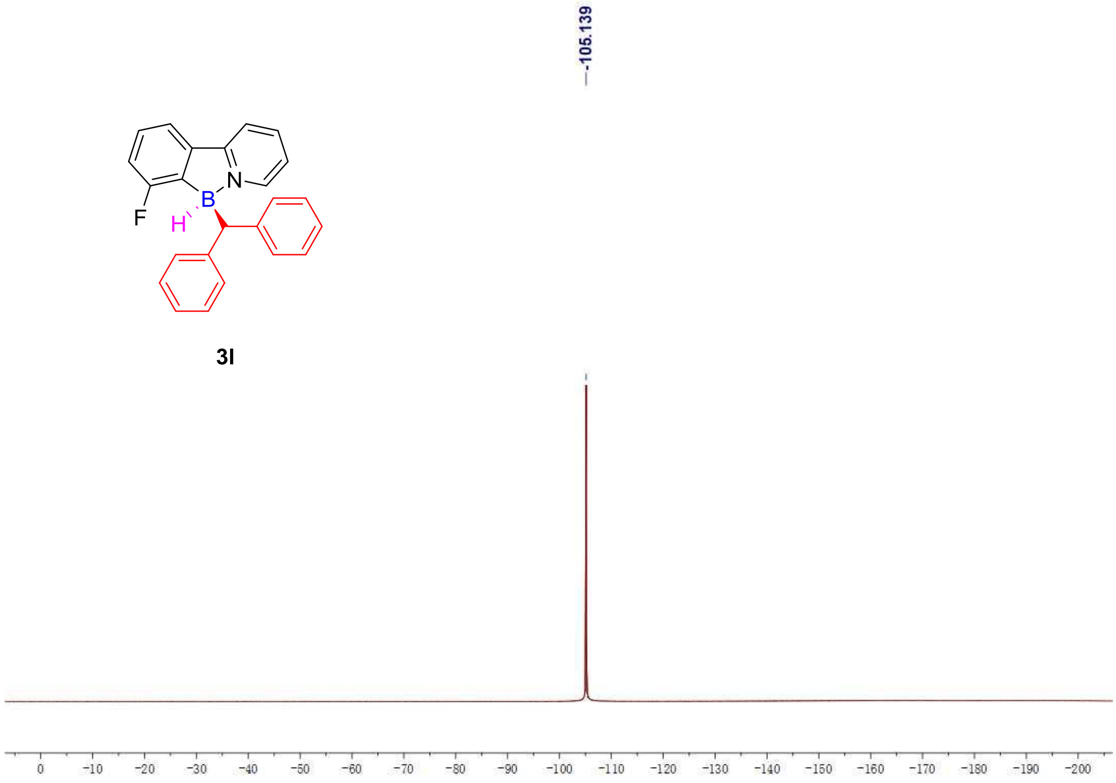

Supplementary Figure 170. ^{19}F NMR spectrum of compound 3l

6-benzhydryl-7-bromo-6H-5 ^{4}-benzo[3,4][1,2]azaborolo[1,5-a]pyridine (3m)

^{1}H NMR (500 MHz, room temperature, $CDCl_3$)

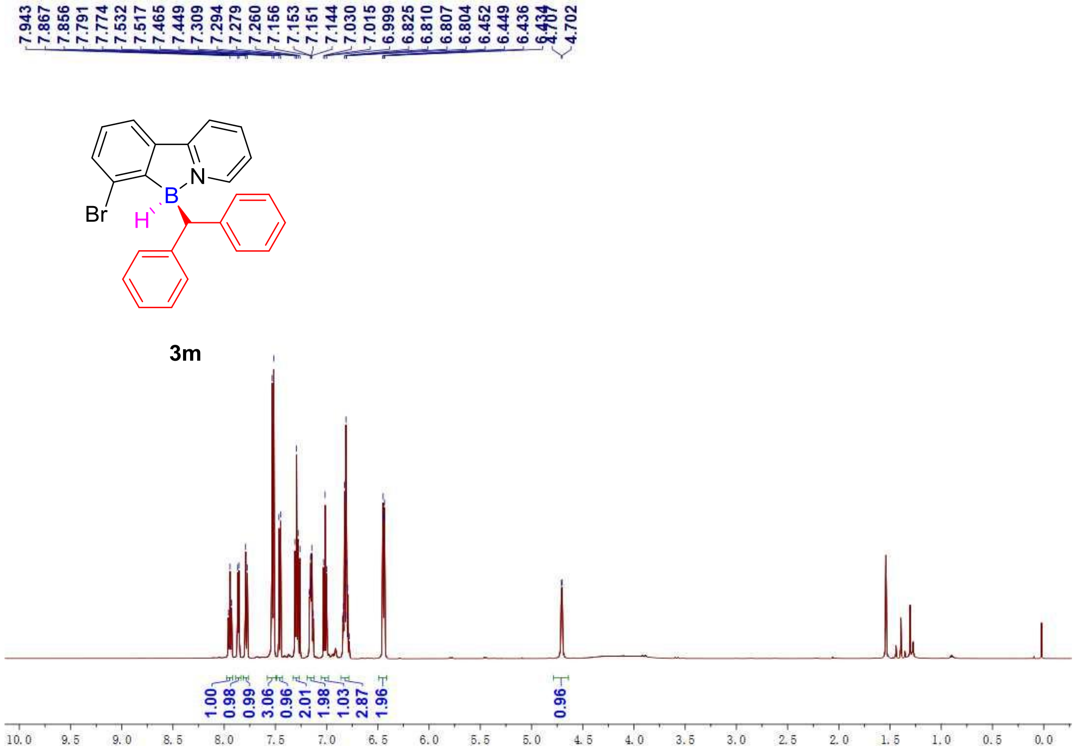

Supplementary Figure 171. ^{1}H NMR spectrum of compound 3m

^{13}C NMR (126 MHz, room temperature, $CDCl_3$)

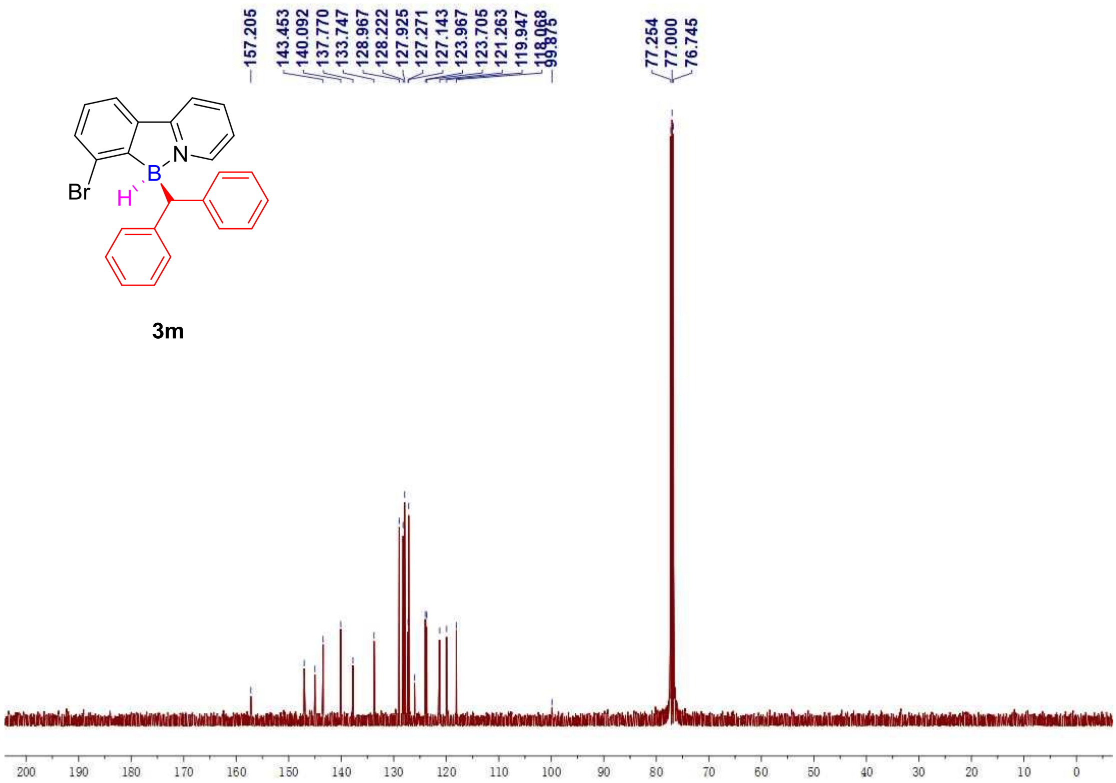

Supplementary Figure 172. ^{13}C NMR spectrum of compound 3m

^{11}B NMR (128 MHz, room temperature, $CDCl_3$)

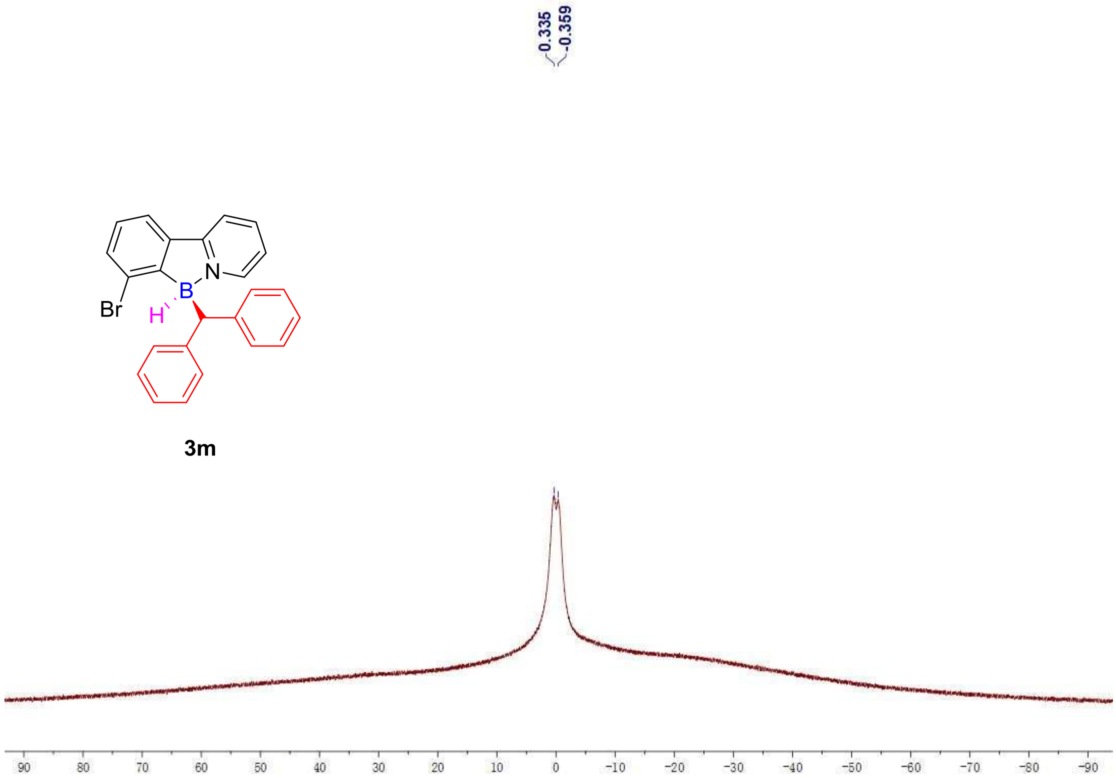

Supplementary Figure 173. ^{11}B NMR spectrum of compound 3m

6-benzhydryl-7-chloro-10-fluoro-6H-5 ^{4}-benzo[3,4][1,2]azaborolo[1,5-a]pyridine (3n)

^{1}H NMR (400 MHz, room temperature, CDCl$_3$)

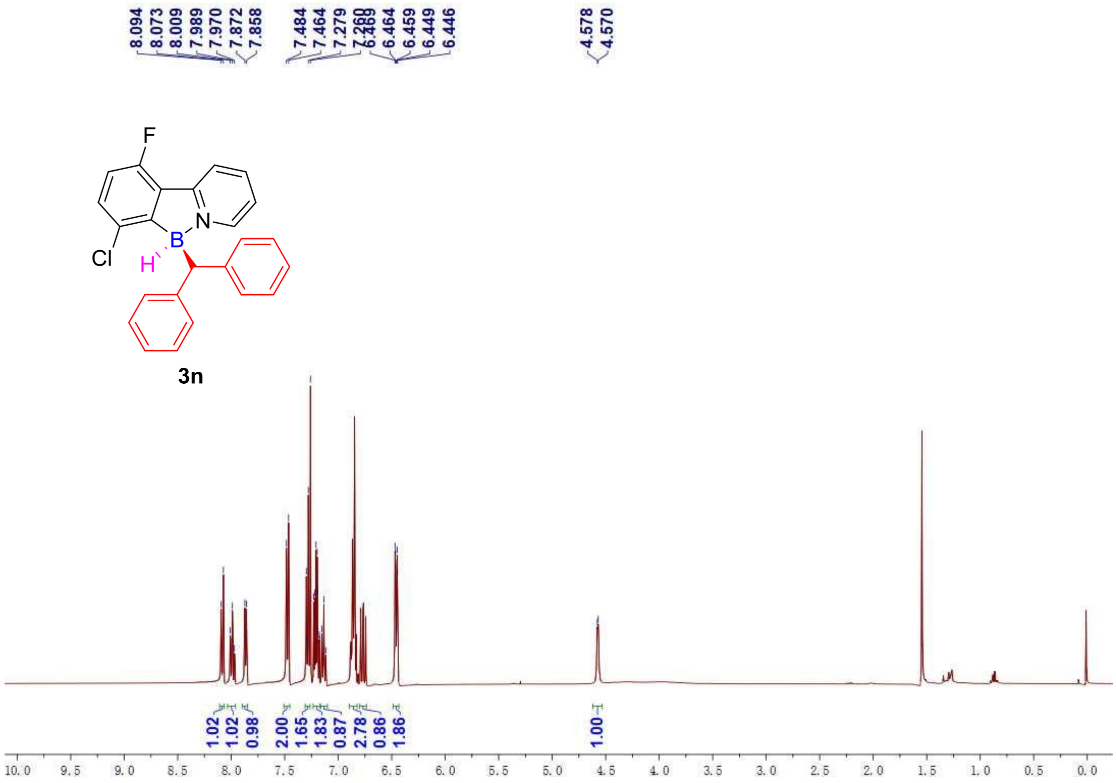

Supplementary Figure 174. ^{1}H NMR spectrum of compound 3n

^{13}C NMR (101 MHz, room temperature, CDCl$_3$)

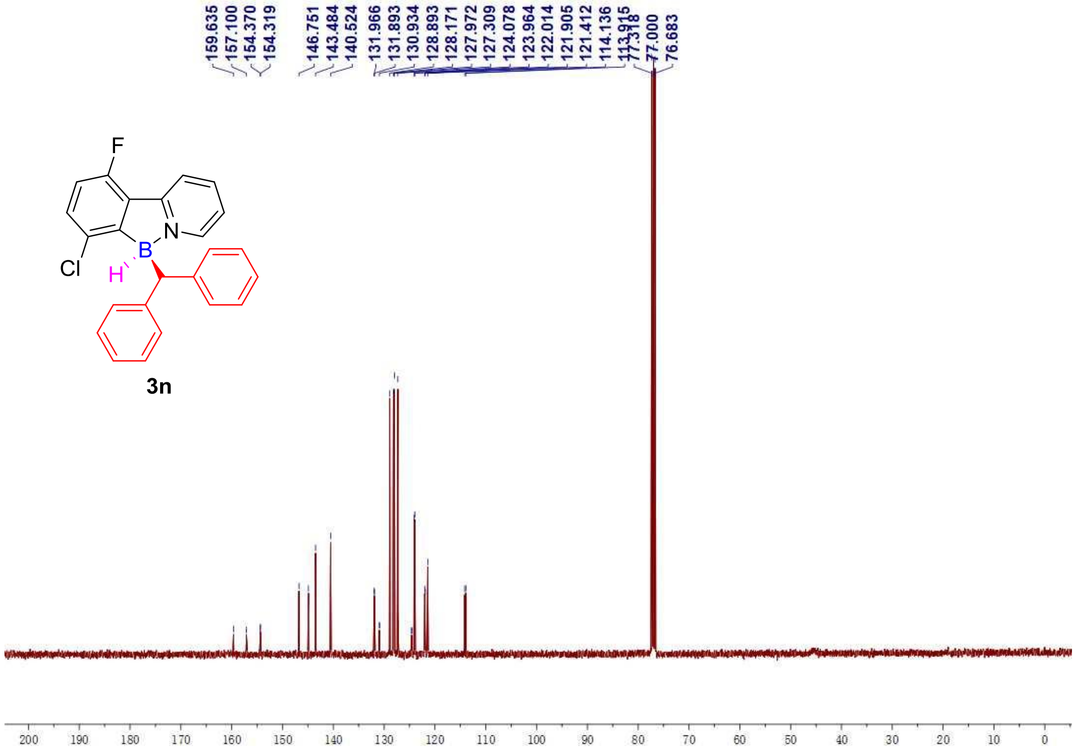

Supplementary Figure 175. ^{13}C NMR spectrum of compound 3n

^{11}B NMR (128 MHz, room temperature, $CDCl_3$)

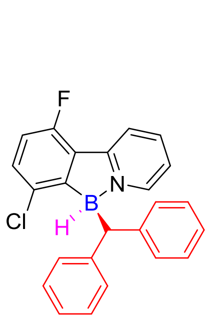

3n

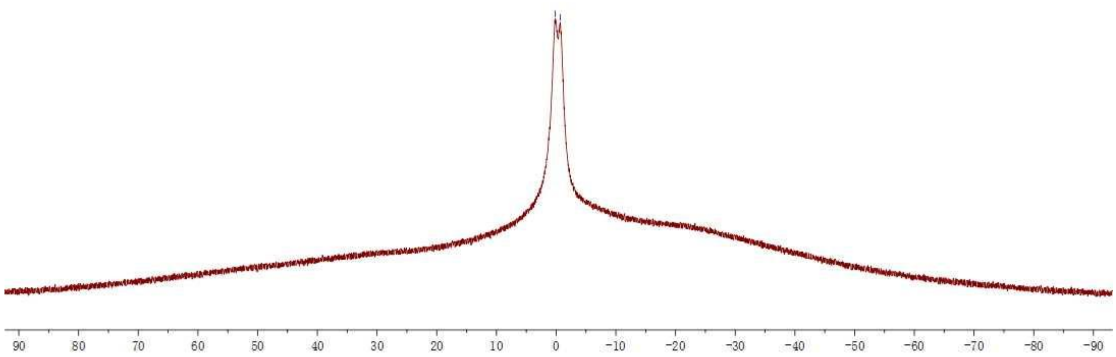

Supplementary Figure 176. ^{11}**B NMR spectrum of compound 3n**

^{19}F NMR (376 MHz, room temperature, $CDCl_3$)

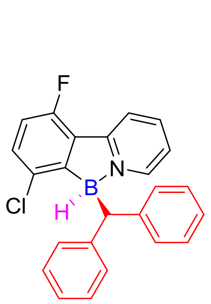

3n

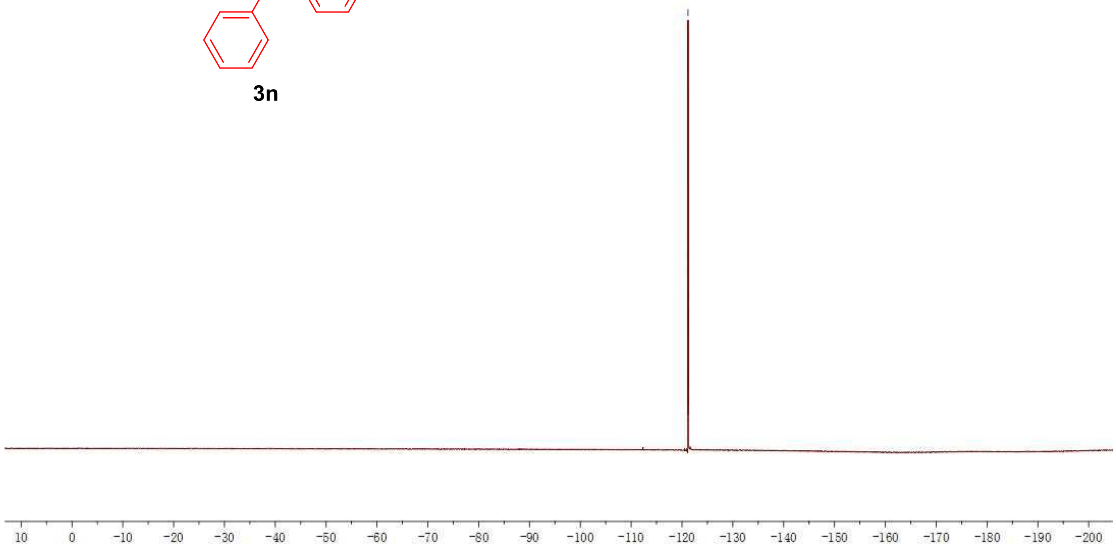

Supplementary Figure 177. ^{19}**F NMR spectrum of compound 3n**

6-benzhydryl-7-chloro-8-fluoro-6H-5 ^{4}-benzo[3,4][1,2]azaborolo[1,5-a]pyridine (3o)

^{1}H NMR (400 MHz, room temperature, CDCl$_3$)

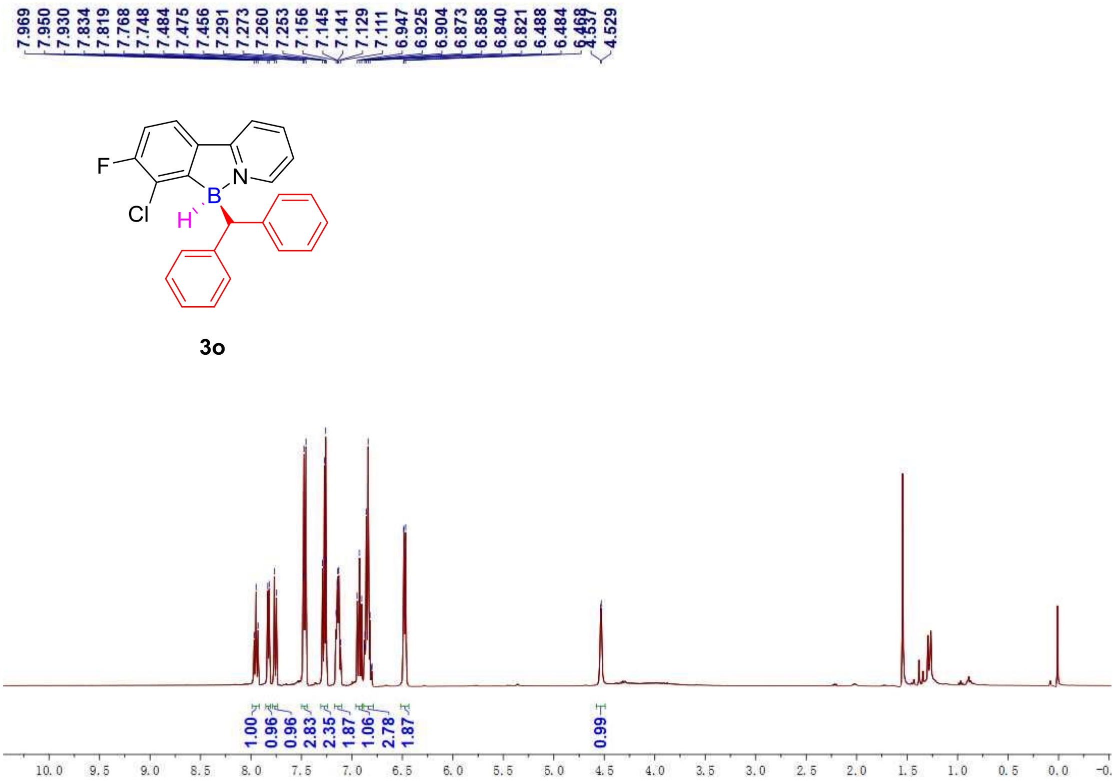

Supplementary Figure 178. ^{1}H NMR spectrum of compound 3o

^{13}C NMR (101 MHz, room temperature, CDCl$_3$)

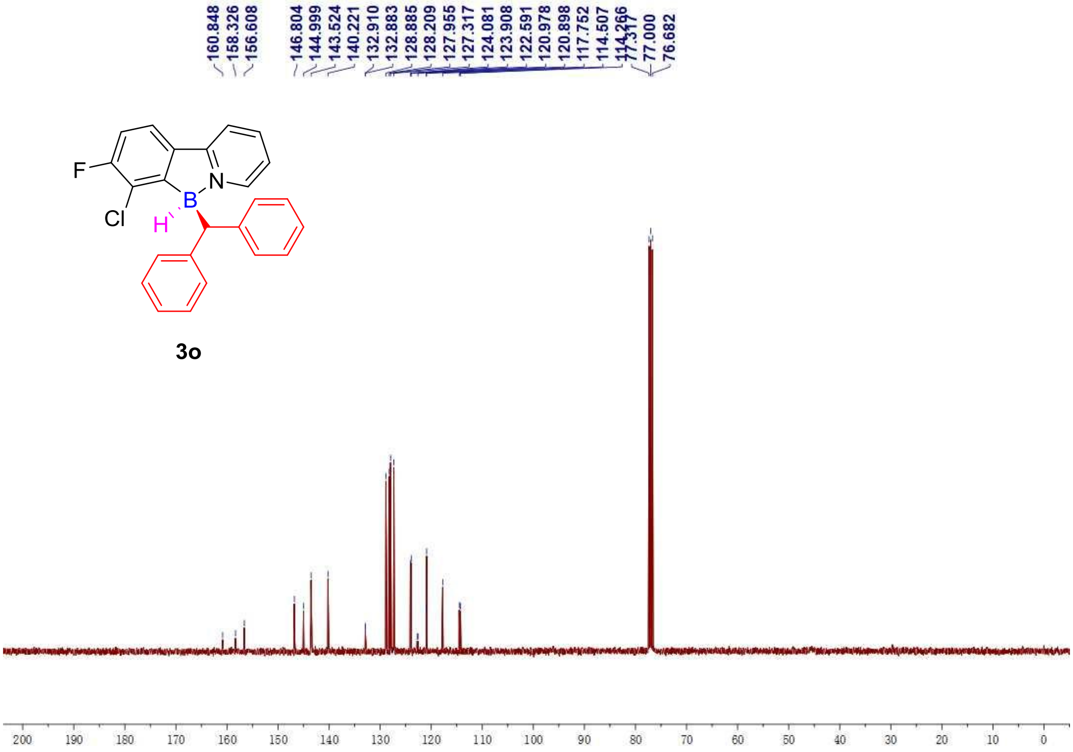

Supplementary Figure 179. ^{13}C NMR spectrum of compound 3o

^{11}B NMR (128 MHz, room temperature, CDCl$_3$)

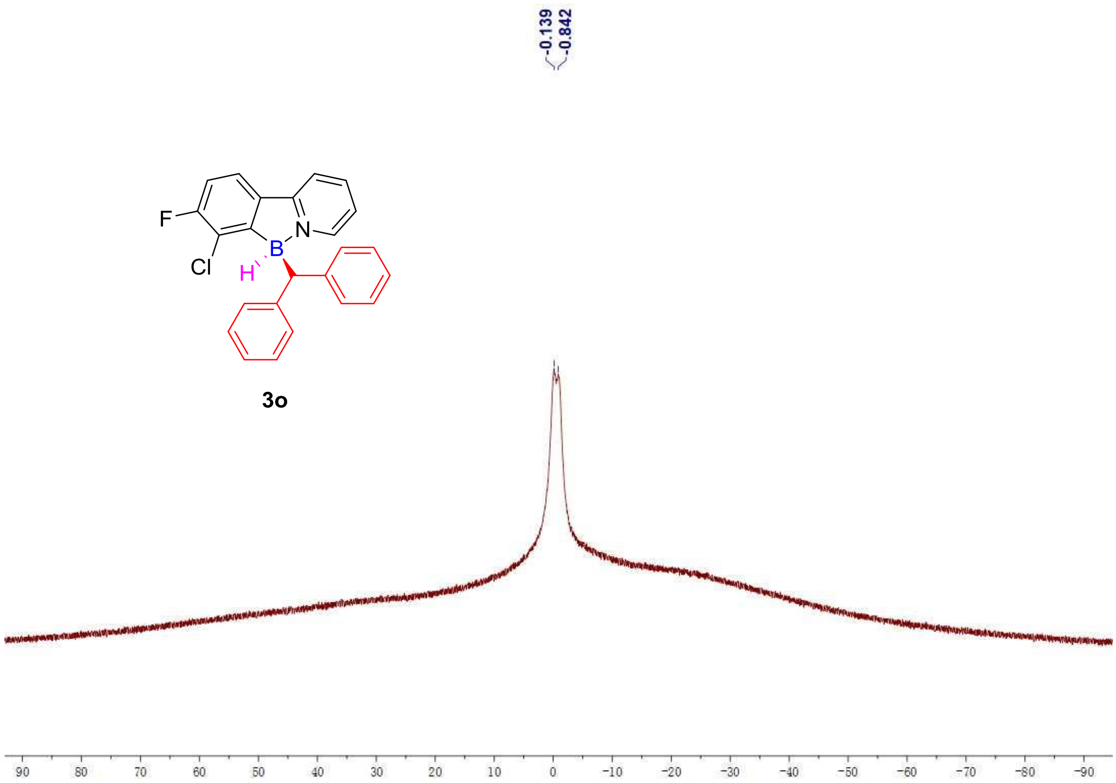

Supplementary Figure 180. ^{11}B NMR spectrum of compound 3o

^{19}F NMR (376 MHz, room temperature, CDCl$_3$)

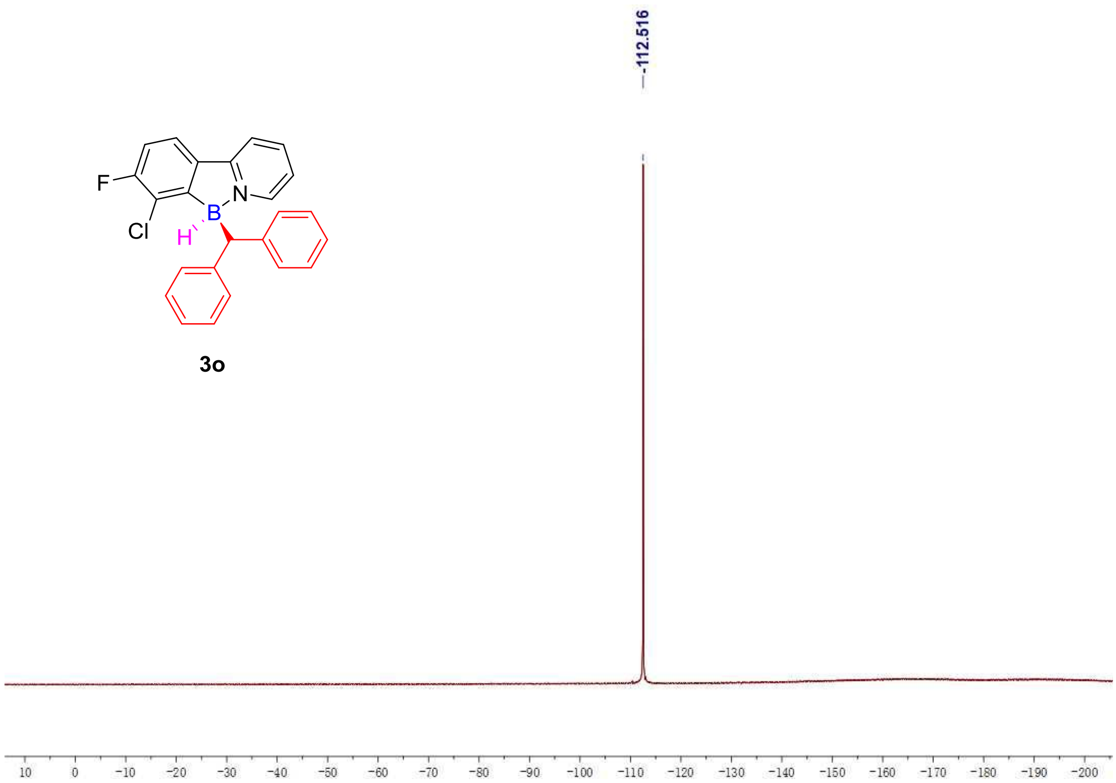

Supplementary Figure 181. ^{19}F NMR spectrum of compound 3o

6-benzhydryl-7,8-dichloro-6H-5 ^{4}-benzo[3,4][1,2]azaborolo[1,5-a]pyridine (3p)

^{1}H NMR (500 MHz, room temperature, $CDCl_3$)

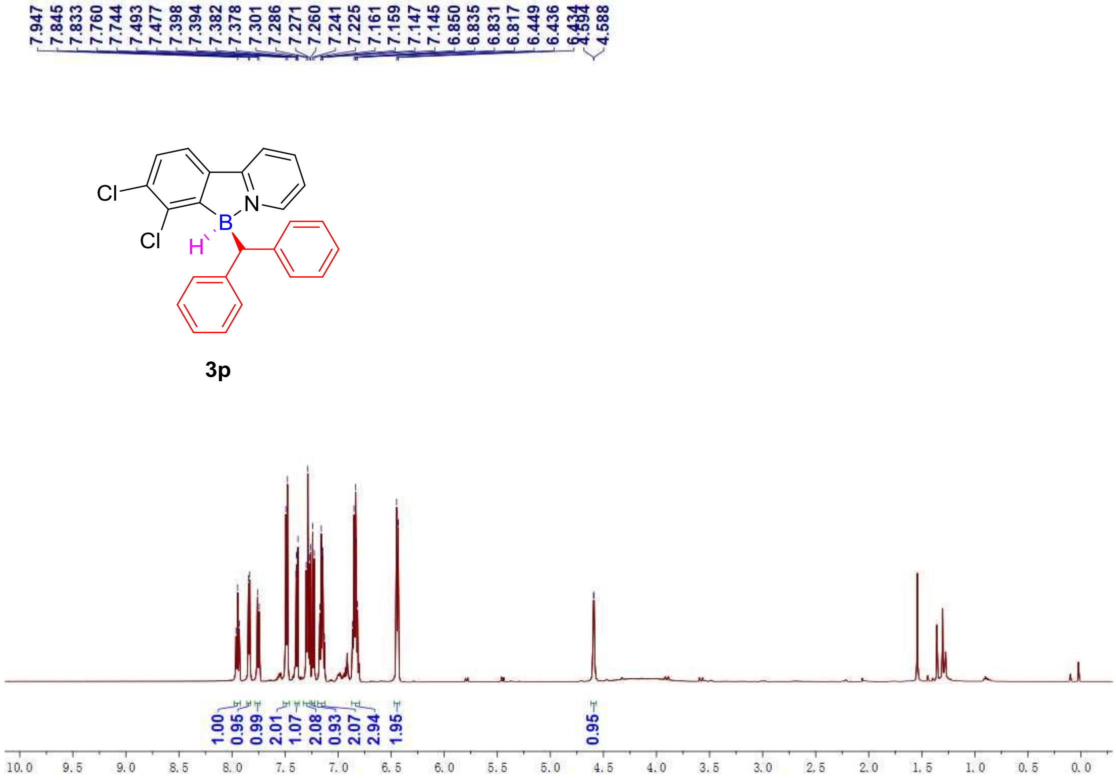

Supplementary Figure 182. ^{1}H NMR spectrum of compound 3p

^{13}C NMR (101 MHz, room temperature, $CDCl_3$)

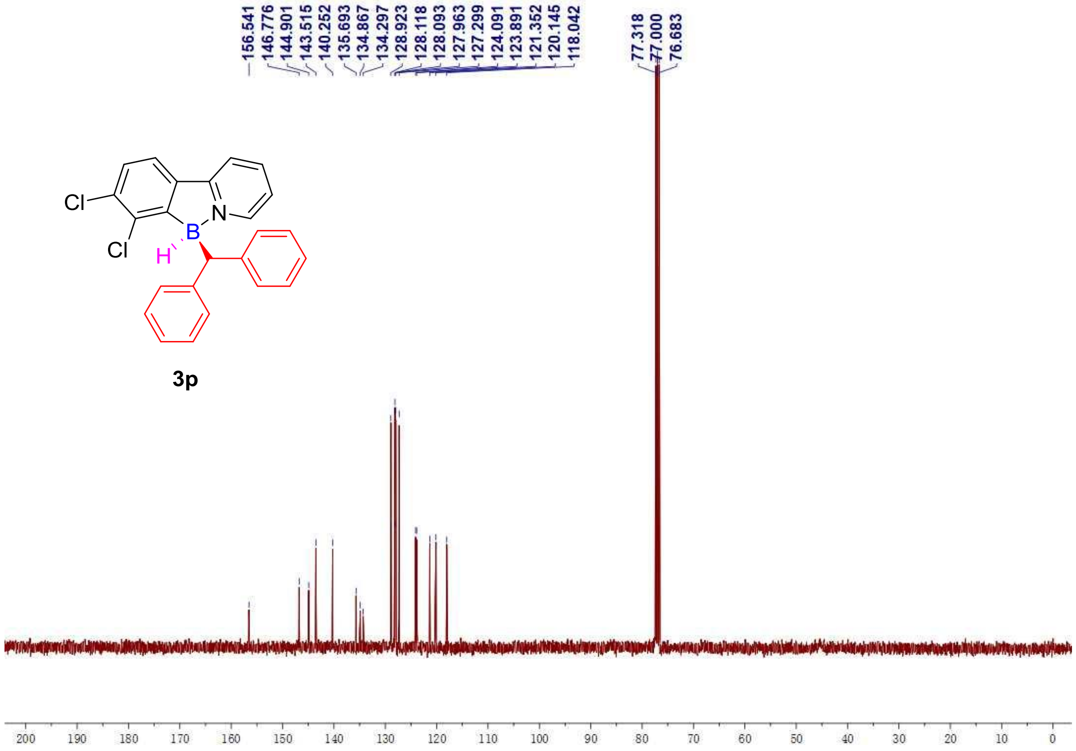

Supplementary Figure 183. ^{13}C NMR spectrum of compound 3p

^{11}B NMR (128 MHz, room temperature, $CDCl_3$)

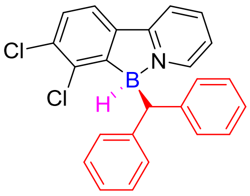

3p

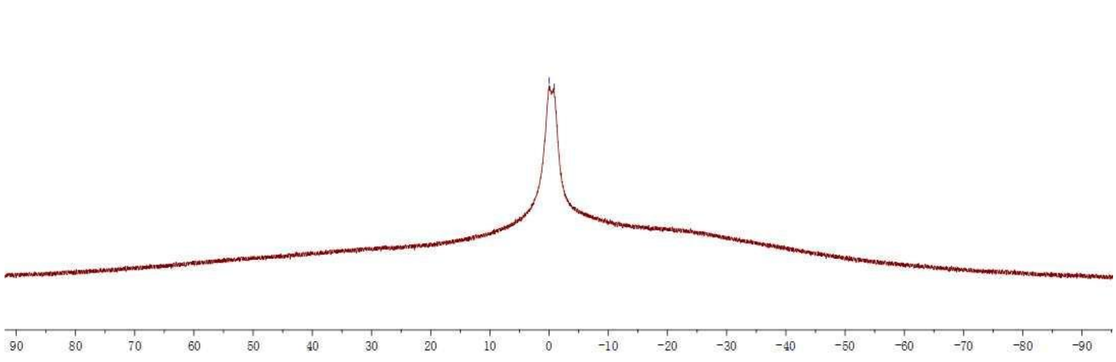

Supplementary Figure 184. ^{11}B NMR spectrum of compound 3p

6-benzhydryl-7,9-dichloro-6H-5 ^{4}-benzo[3,4][1,2]azaborolo[1,5-a]pyridine (3q)

^{1}H NMR (500 MHz, room temperature, $CDCl_3$)

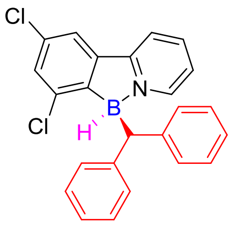

3q

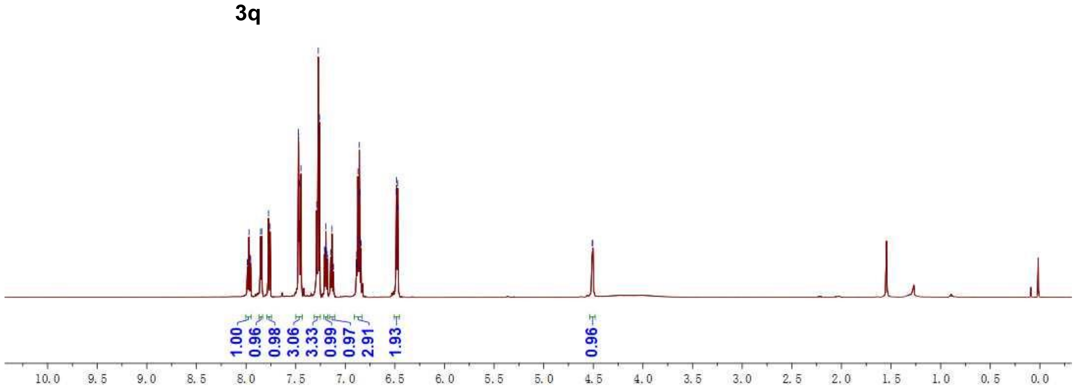

Supplementary Figure 185. ^{1}H NMR spectrum of compound 3q

^{13}C NMR (101 MHz, room temperature, $CDCl_3$)

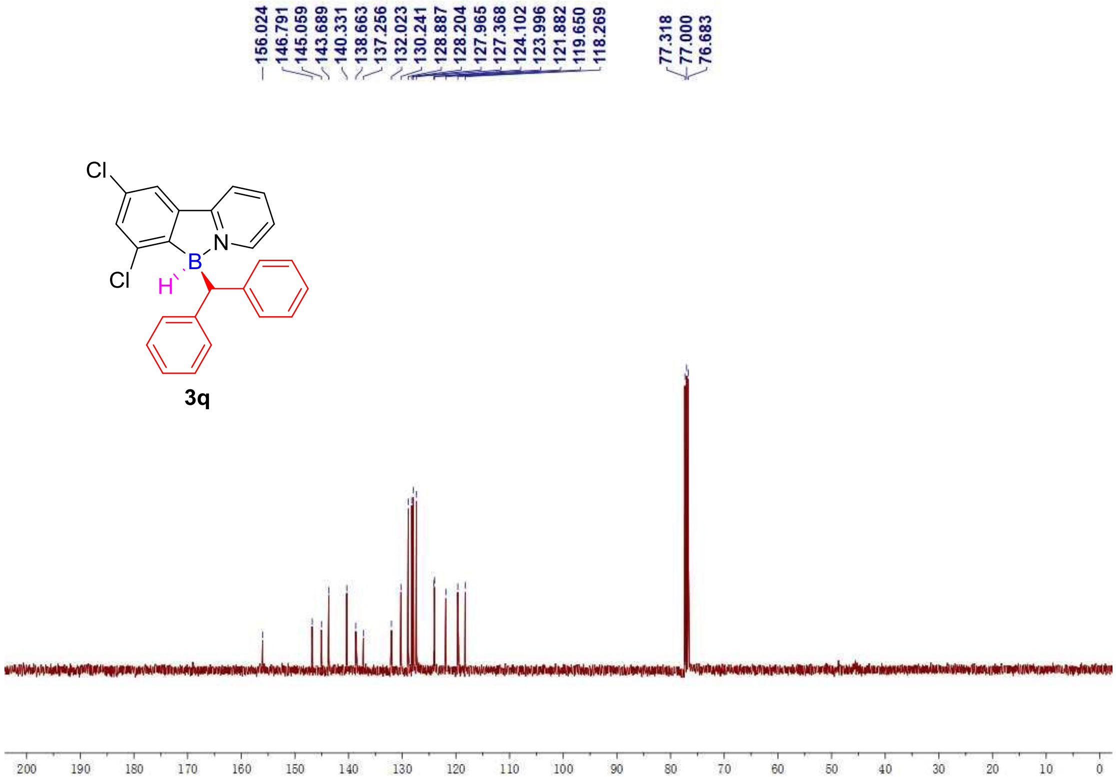

Supplementary Figure 186. ^{13}C NMR spectrum of compound 3q

^{11}B NMR (128 MHz, room temperature, $CDCl_3$)

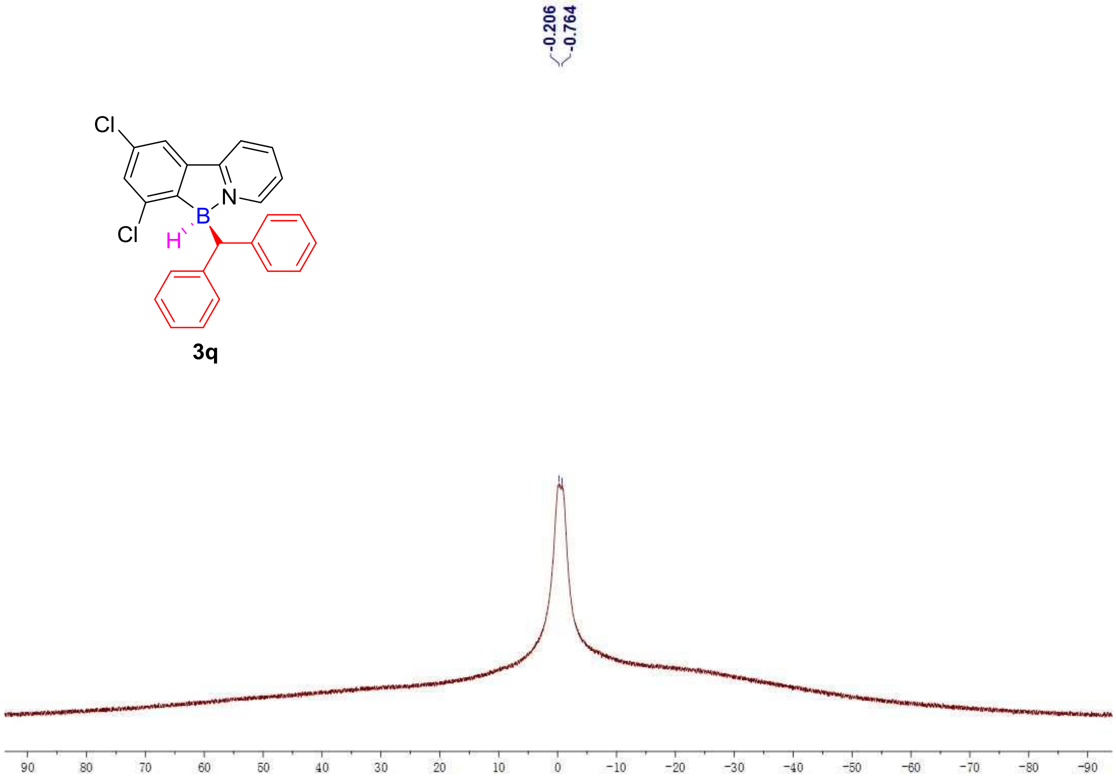

Supplementary Figure 187. ^{11}B NMR spectrum of compound 3q

6-benzhydryl-7-chloro-10-methyl-6H-5 ^{4}-benzo[3,4][1,2]azaborolo[1,5-a]pyridine (3r)

^{1}H NMR (500 MHz, room temperature, CDCl$_3$)

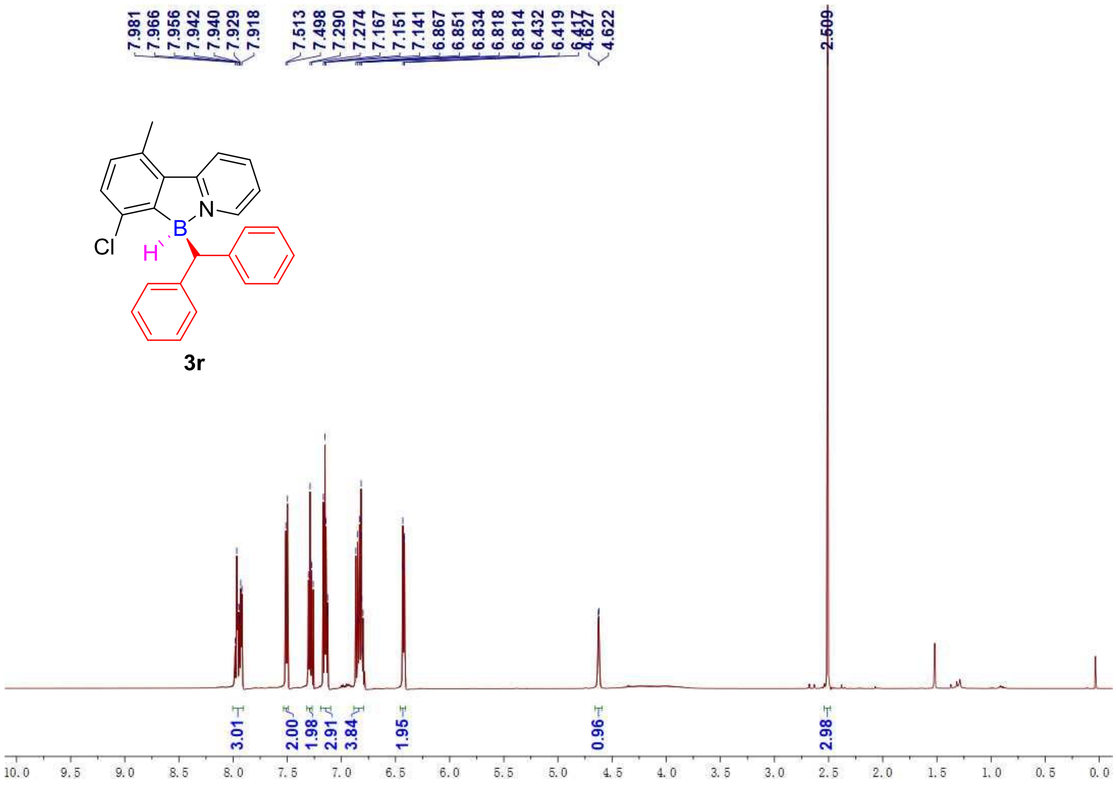

Supplementary Figure 188. ^{1}H NMR spectrum of compound 3r

^{13}C NMR (126 MHz, room temperature, CDCl$_3$)

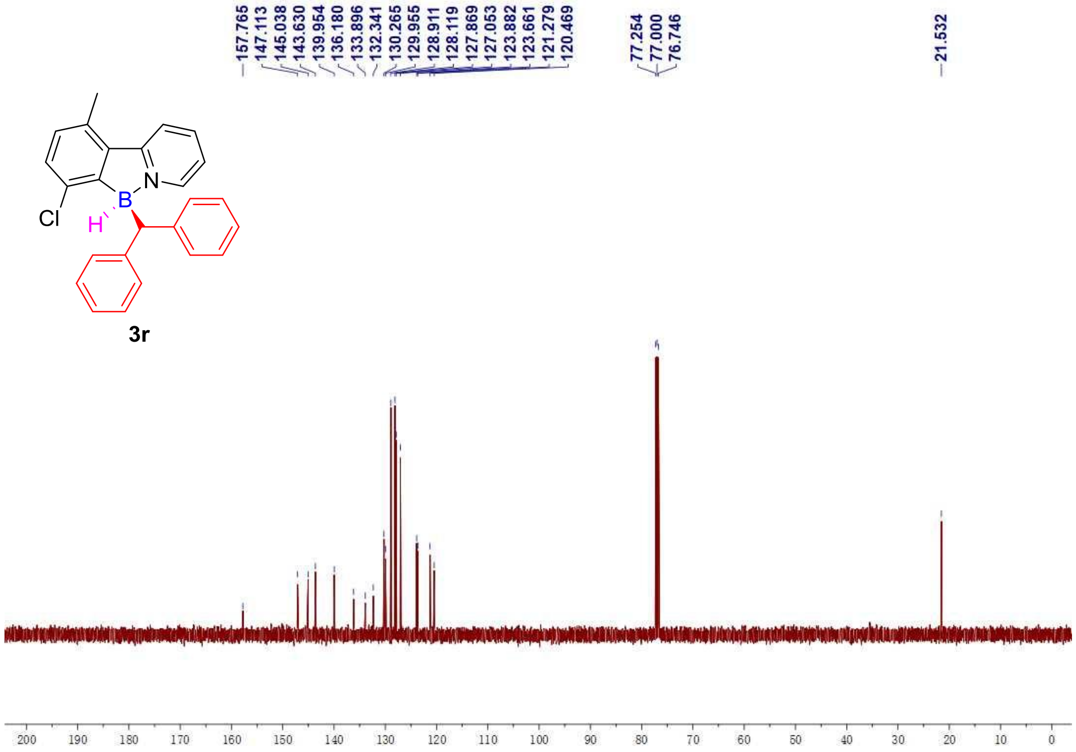

Supplementary Figure 189. ^{13}C NMR spectrum of compound 3r

^{11}B NMR (128 MHz, room temperature, CDCl$_3$)

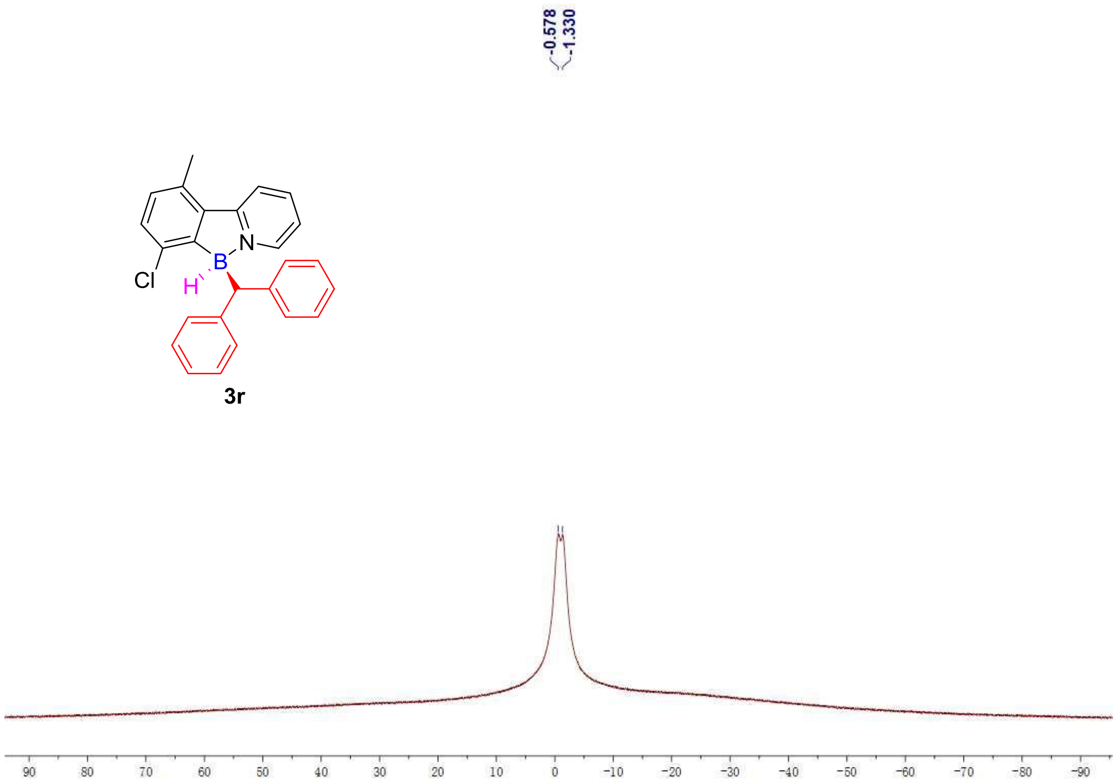

Supplementary Figure 190. ^{11}B NMR spectrum of compound 3r

12-benzhydryl-12H-11 ^{4}-naphtho[1',2':3,4][1,2]azaborolo[1,5-a]pyridine (3s)

^{1}H NMR (500 MHz, room temperature, CDCl$_3$)

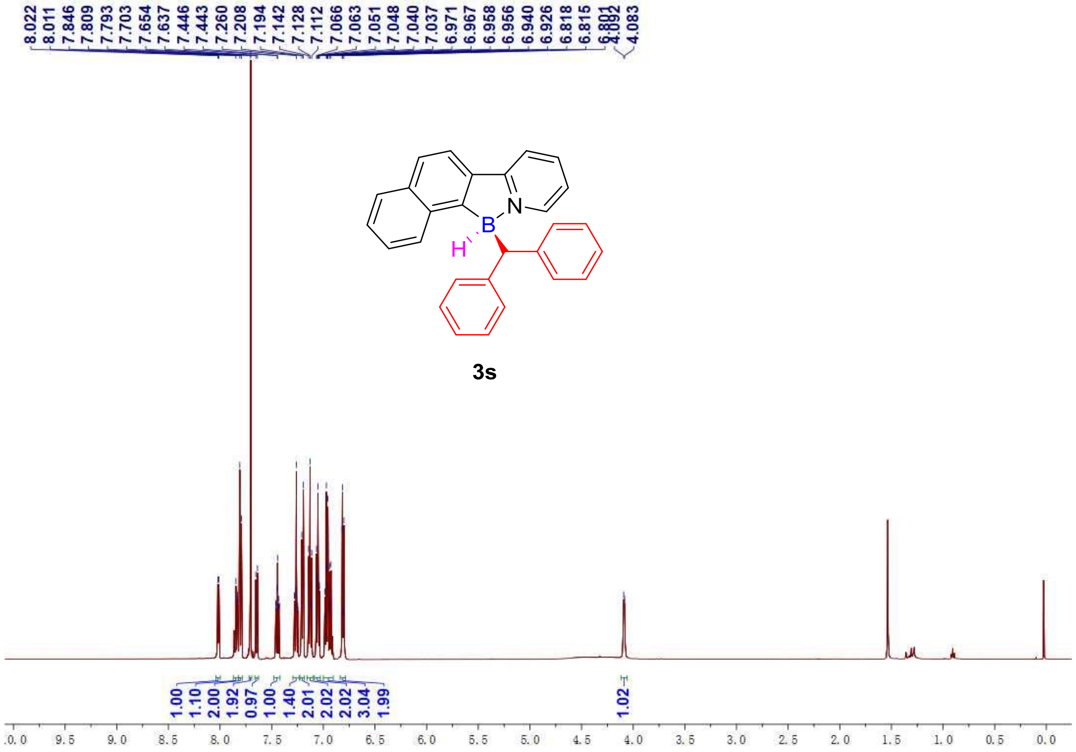

Supplementary Figure 191. ^{1}H NMR spectrum of compound 3s

^{13}C NMR (101 MHz, room temperature, $CDCl_3$)

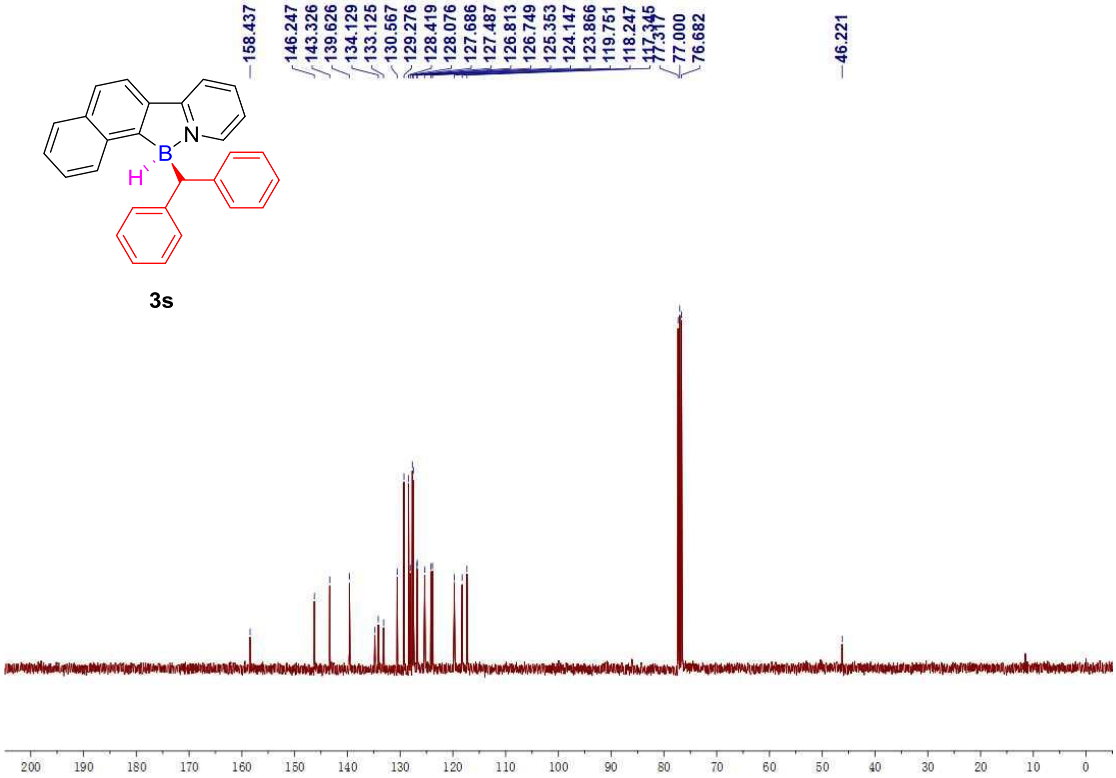

Supplementary Figure 192. ^{13}C NMR spectrum of compound 3s

^{11}B NMR (128 MHz, room temperature, $CDCl_3$)

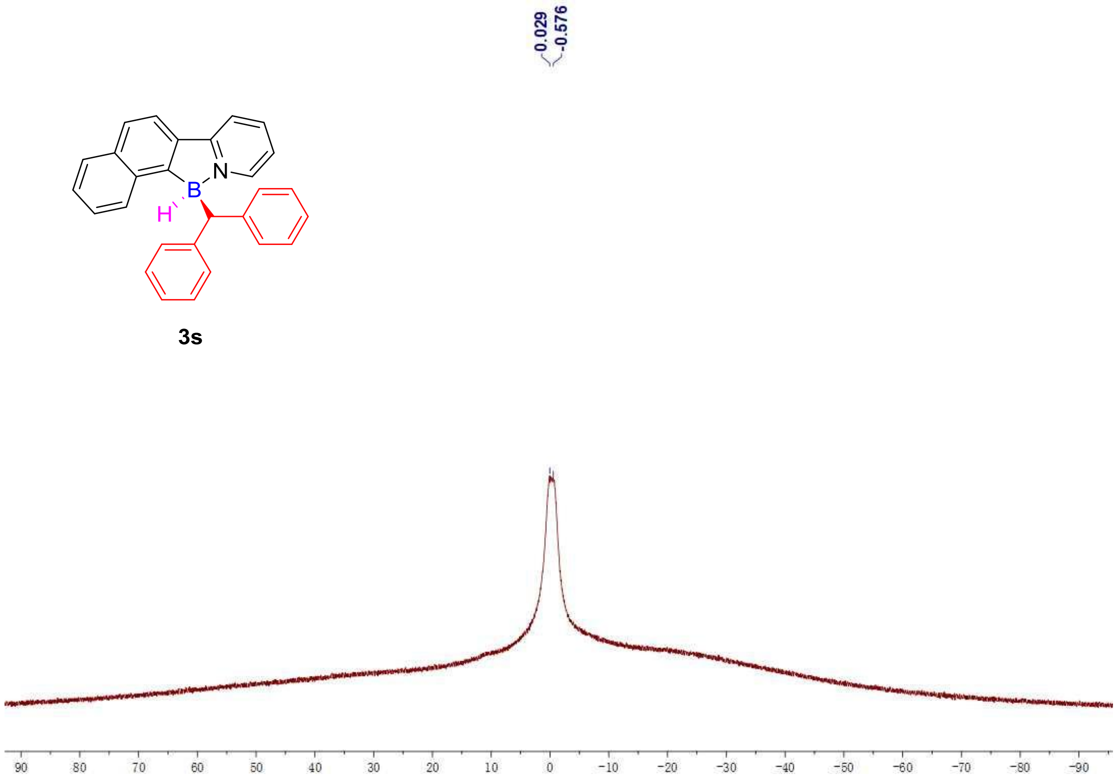

Supplementary Figure 193. ^{11}B NMR spectrum of compound 3s

ethyl (*S*)-2-(7-chloro-(*R*)6H-5 ^{4}-benzo[3,4][1,2]azaborolo[1,5-a]pyridin-6-yl)-2-phenylacetate (5a)

^{1}H NMR (500 MHz, room temperature, $CDCl_3$)

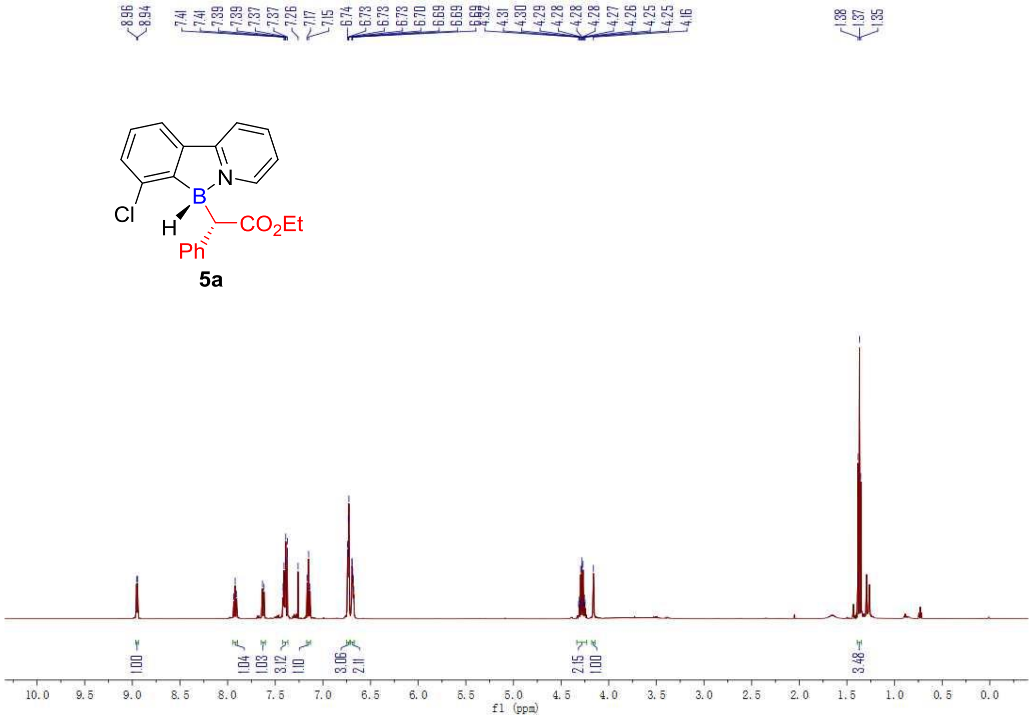

Supplementary Figure 194. ^{1}H NMR spectrum of compound 5a

^{13}C NMR (126 MHz, room temperature, $CDCl_3$)

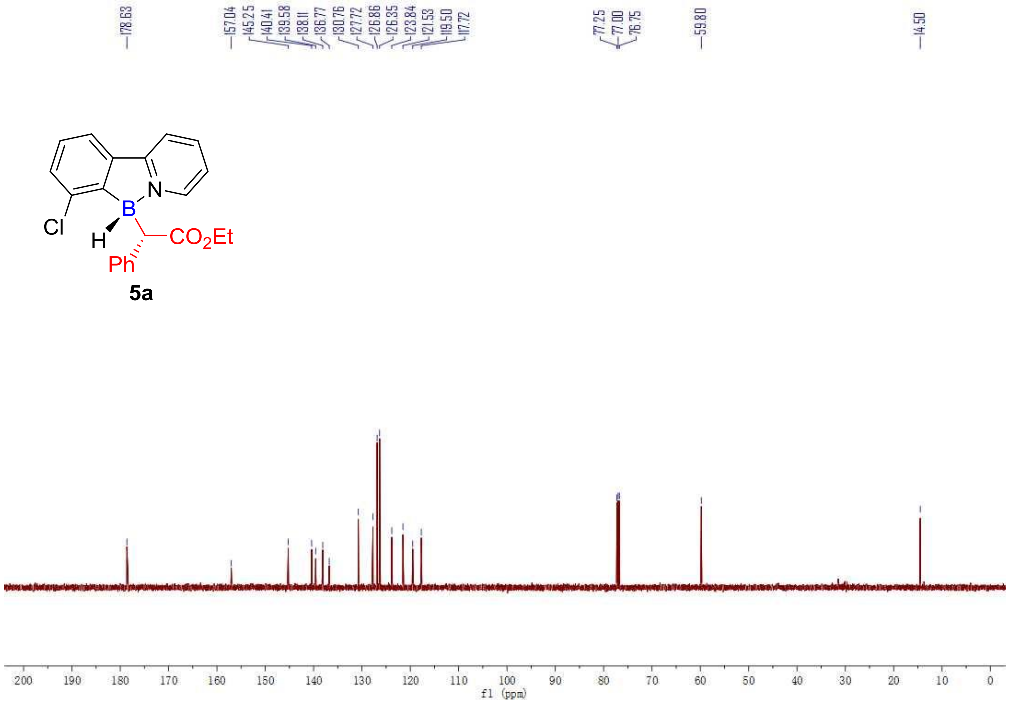

Supplementary Figure 195. ^{13}C NMR spectrum of compound 5a

^{11}B NMR (128 MHz, room temperature, CDCl$_3$)

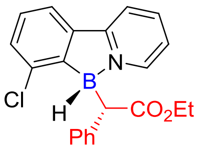

5a

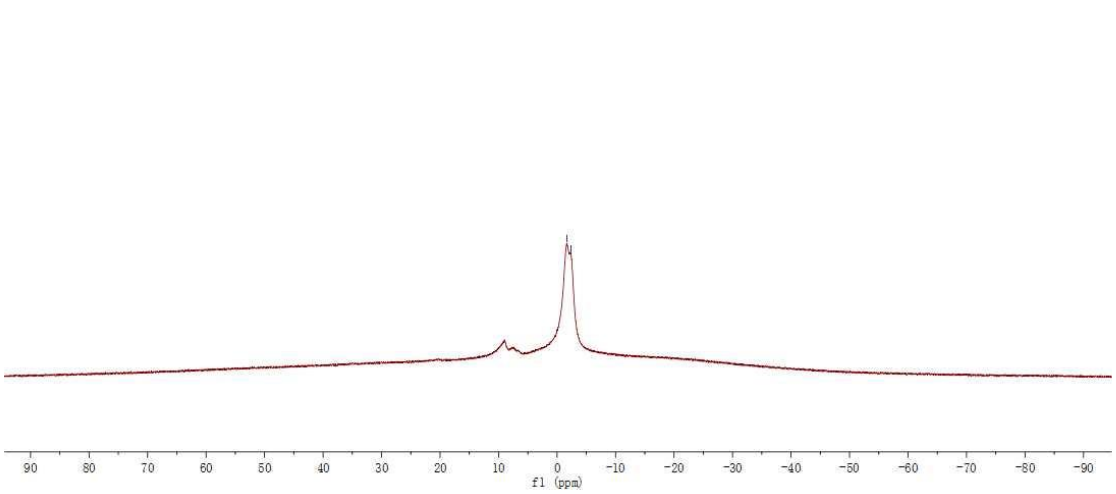

Supplementary Figure 196. ^{11}B NMR spectrum of compound 5a

ethyl 2-(7-chloro-6H-5 ^{4}-benzo[3,4][1,2]azaborolo[1,5-a]pyridin-6-yl)-2-phenylacetate (5a')

^{1}H NMR (400 MHz, room temperature, CDCl$_3$)

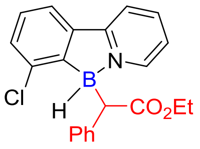

5a'

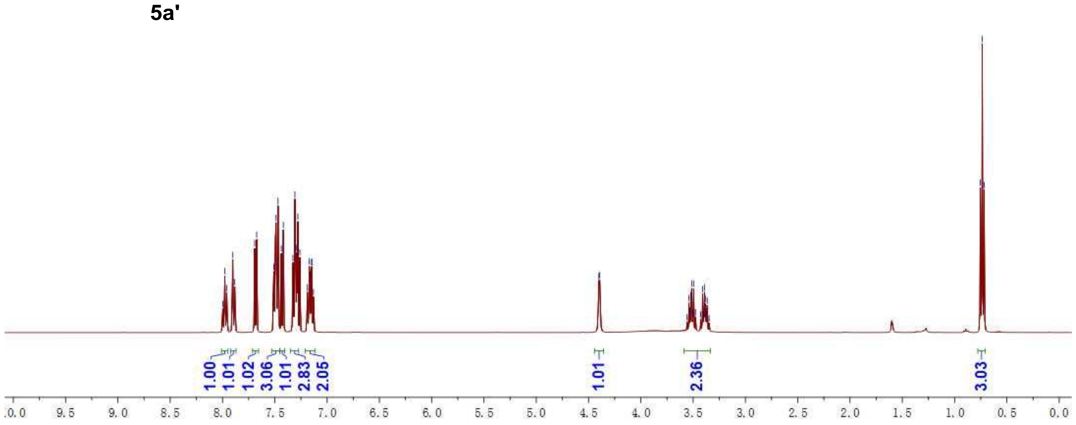

Supplementary Figure 197. ^{1}H NMR spectrum of compound 5a'

^{13}C NMR (101 MHz, room temperature, $CDCl_3$)

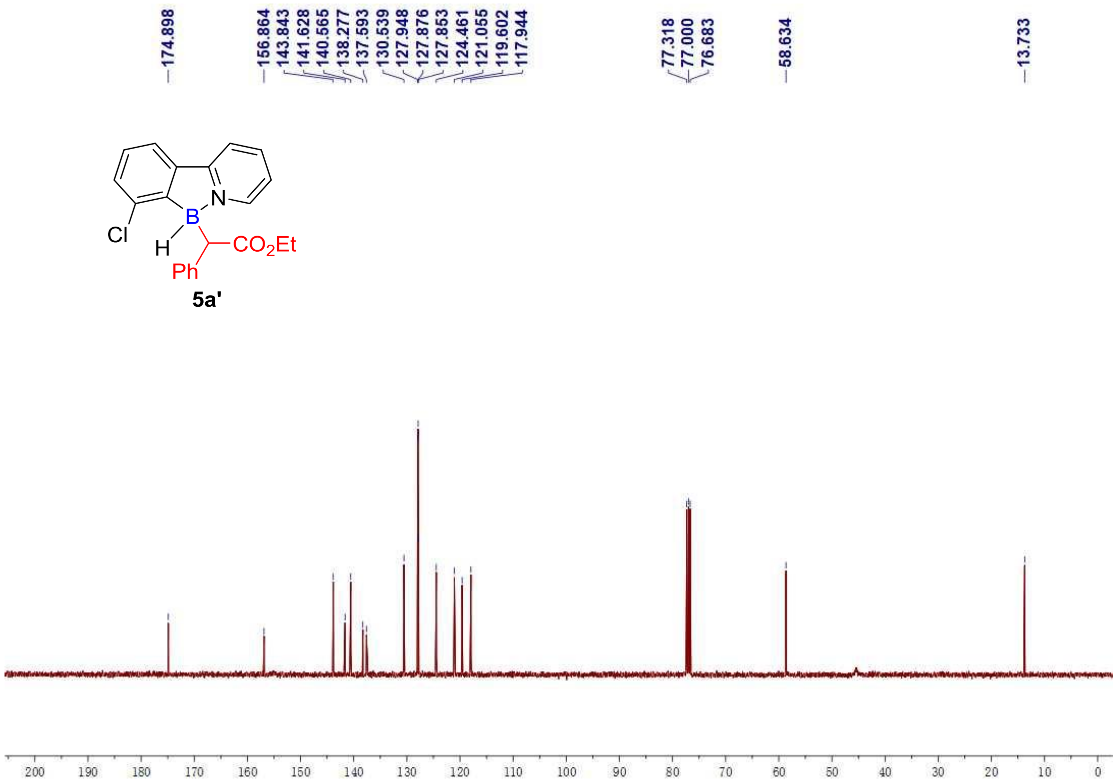

Supplementary Figure 198. ^{13}C NMR spectrum of compound 5a'

^{11}B NMR (128 MHz, room temperature, $CDCl_3$)

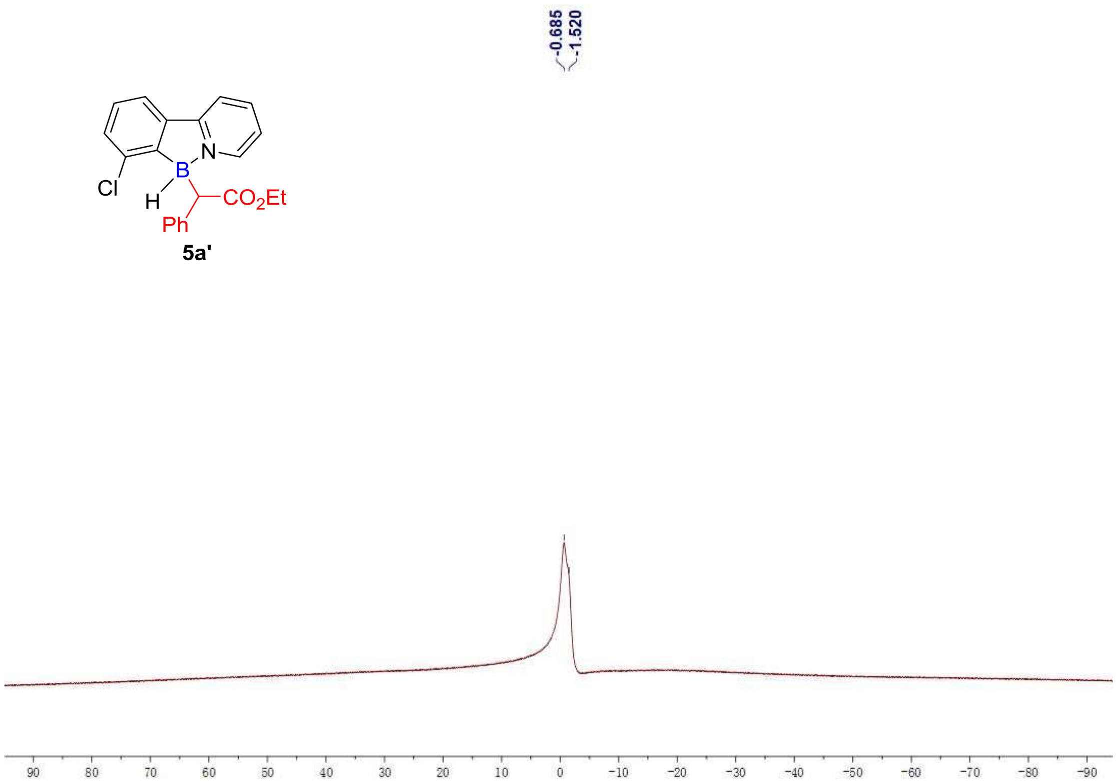

Supplementary Figure 199. ^{11}B NMR spectrum of compound 5a'

ethyl (S)-2-(7-fluoro-6H-5 ^{4}-benzo[3,4][1,2]azaborolo[1,5-a]pyridin-6-yl)-2-phenylacetate (5b)

^{1}H NMR (400 MHz, room temperature, $CDCl_3$)

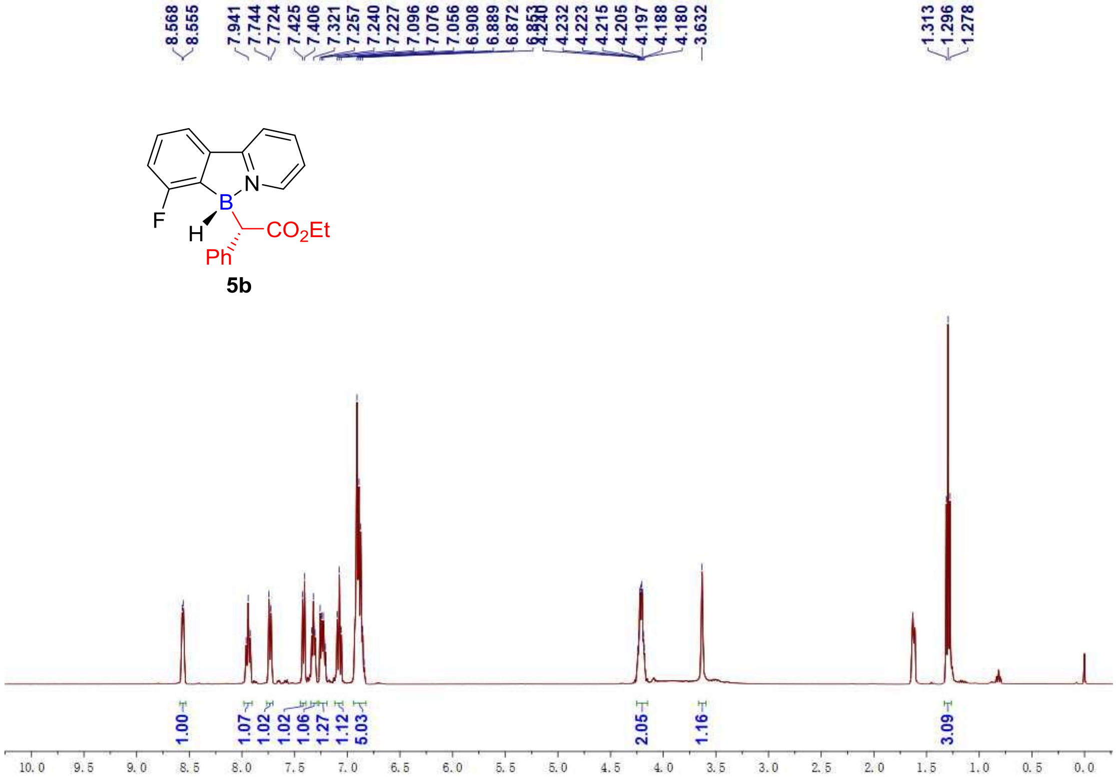

Supplementary Figure 200. ^{1}H NMR spectrum of compound 5b

^{13}C NMR (101 MHz, room temperature, $CDCl_3$)

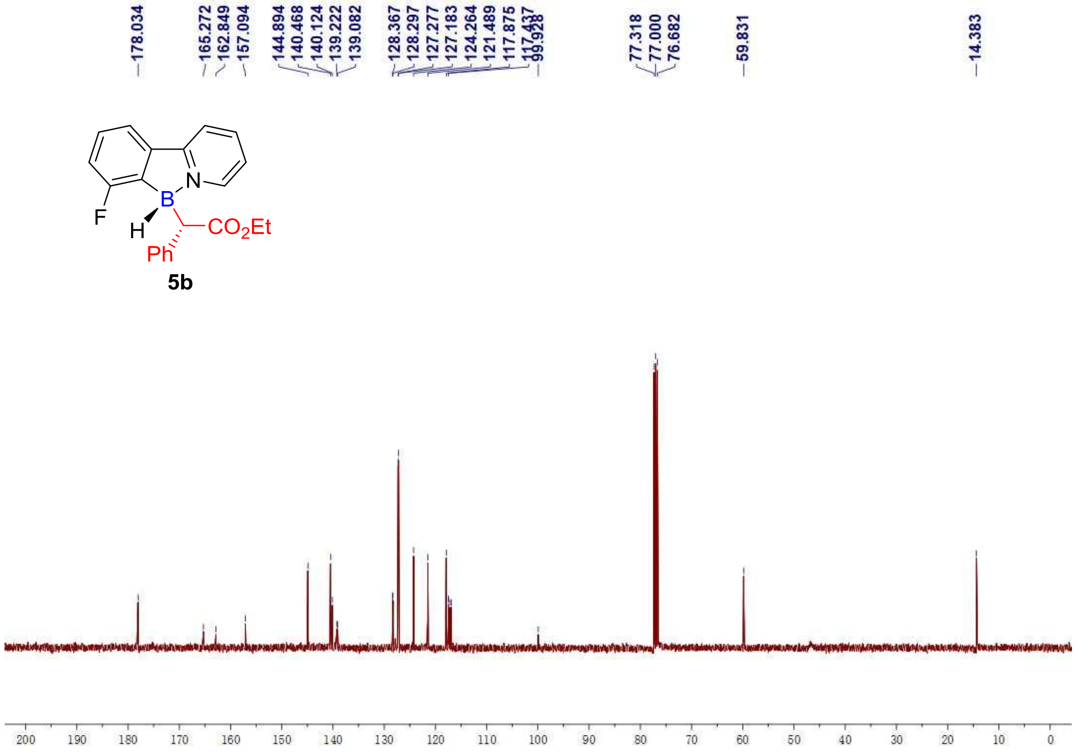

Supplementary Figure 201. ^{13}C NMR spectrum of compound 5b

^{11}B NMR (128 MHz, room temperature, $CDCl_3$)

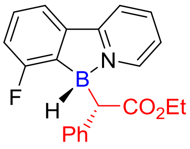

5b

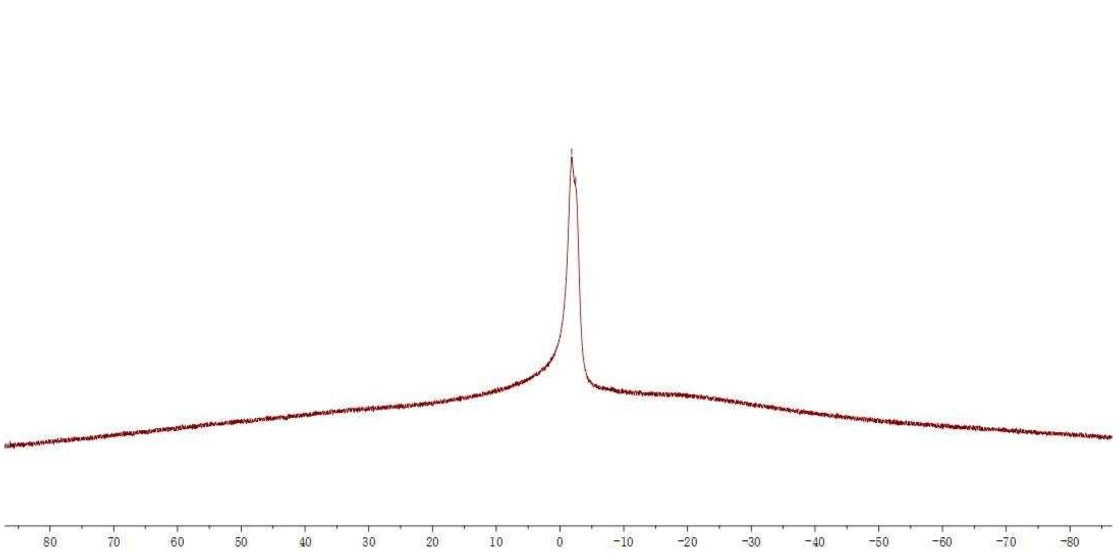

Supplementary Figure 202. ^{11}B NMR spectrum of compound 5b

^{19}F NMR (376 MHz, room temperature, $CDCl_3$)

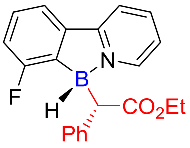

5b

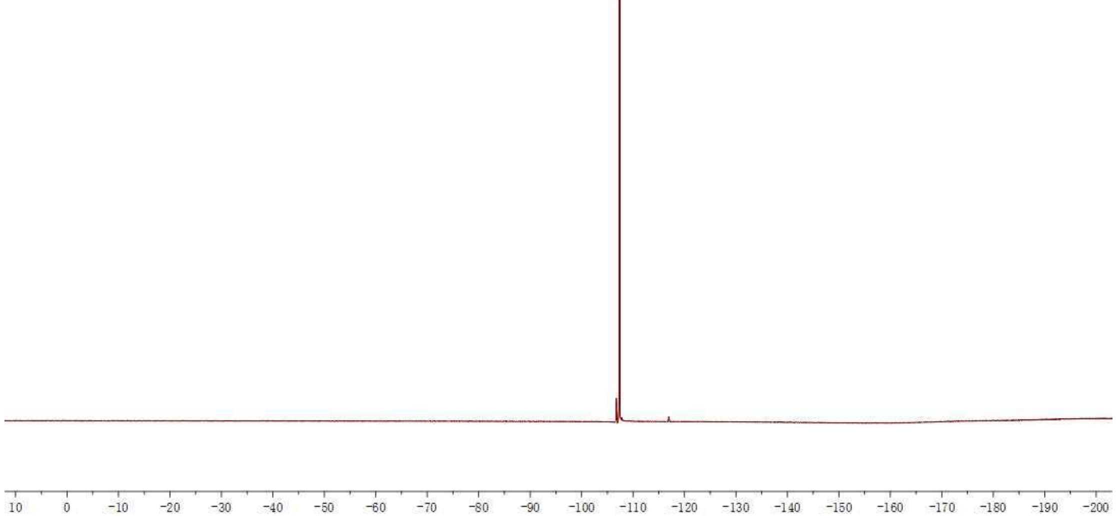

Supplementary Figure 203. ^{19}F NMR spectrum of compound 5b

ethyl(*S*)-2-(8-fluoro-(*R*)6H-5 ^{4}-benzo[3,4][1,2]azaborolo[1,5-a]pyridin-6-yl)-2-phenylacetate (5c)

^{1}H NMR (500 MHz, room temperature, $CDCl_3$)

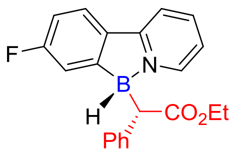

5c

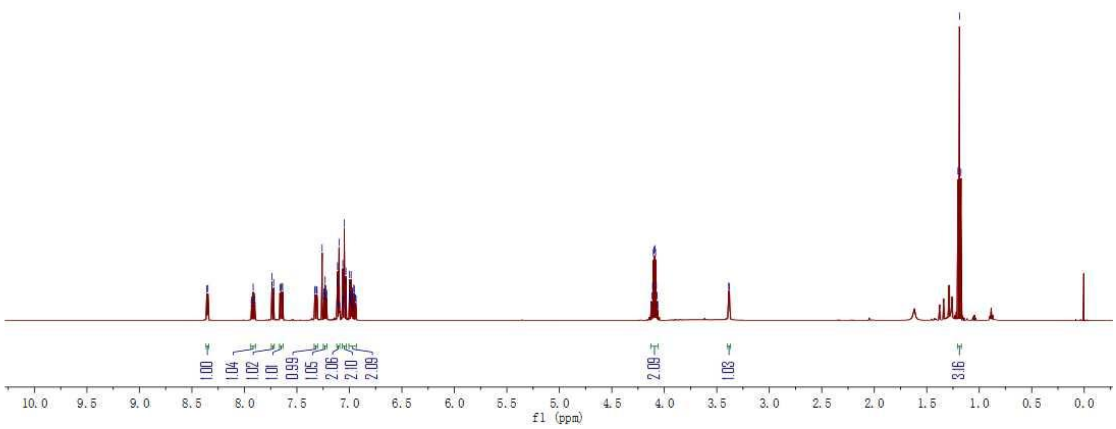

Supplementary Figure 204. ^{1}H NMR spectrum of compound 5c

^{13}C NMR (126 MHz, room temperature, $CDCl_3$)

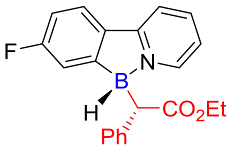

5c

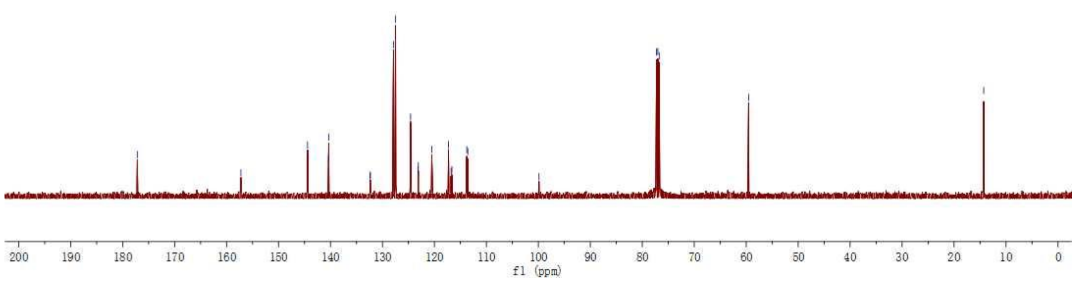

Supplementary Figure 205. ^{13}C NMR spectrum of compound 5c

^{11}B NMR (128 MHz, room temperature, $CDCl_3$)

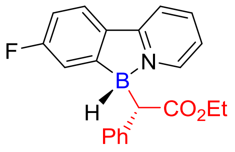

5c

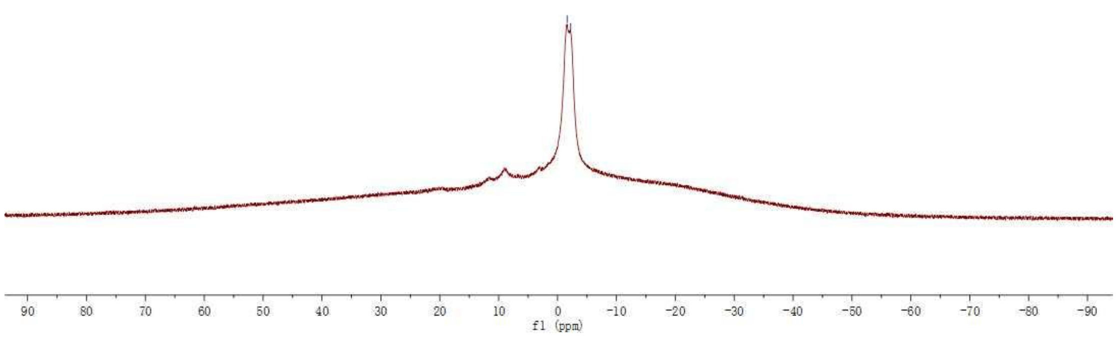

Supplementary Figure 206. ^{11}**B NMR spectrum of compound 5c**

^{19}F NMR (471 MHz, room temperature, $CDCl_3$)

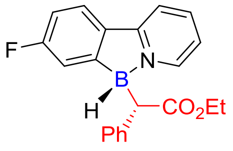

5c

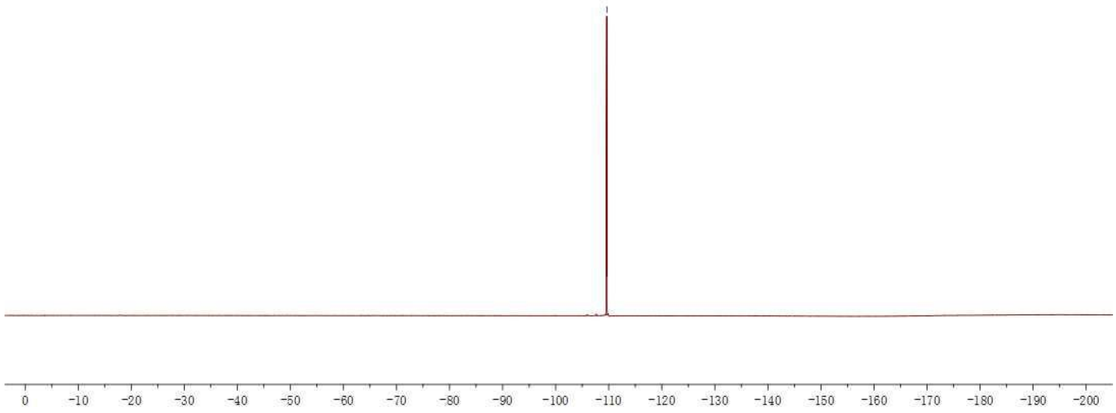

Supplementary Figure 207. ^{19}**F NMR spectrum of compound 5c**

ethyl (S)-2-(7-bromo-6H-5 ^{4}-benzo[3,4][1,2]azaborolo[1,5-a]pyridin-6-yl)-2-phenylacetate (5d)

^{1}H NMR (500 MHz, room temperature, CDCl$_3$)

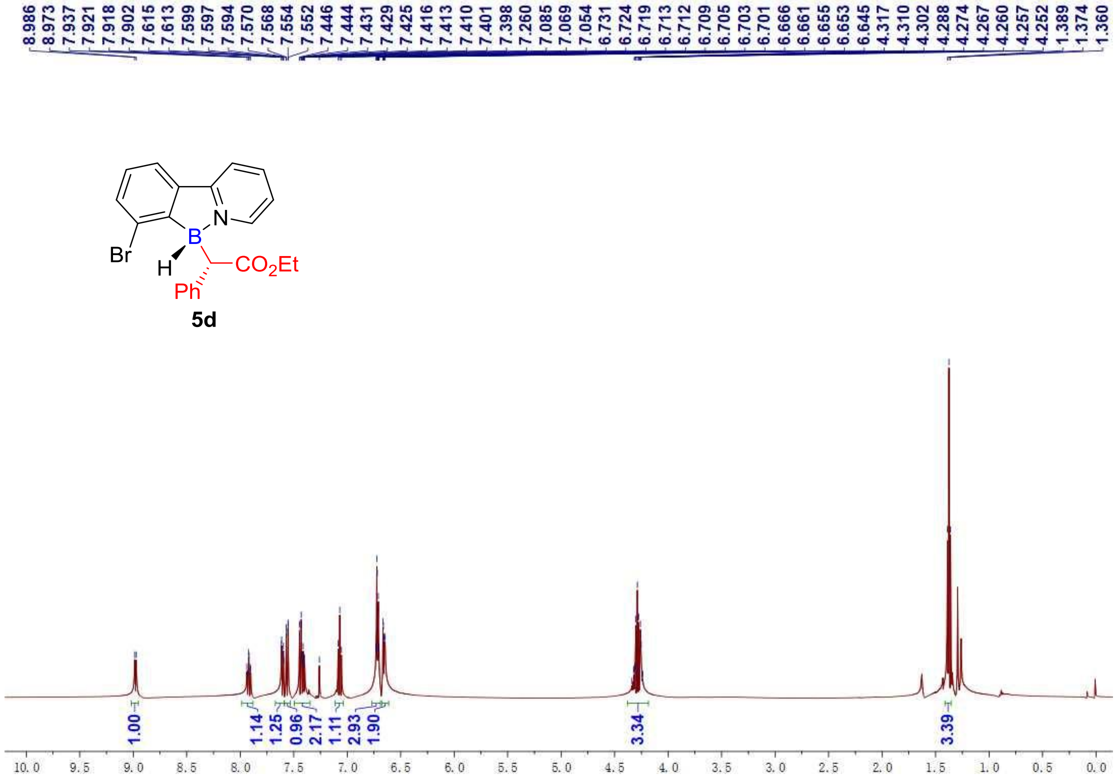

Supplementary Figure 208. ^{1}H NMR spectrum of compound 5d

^{13}C NMR (126 MHz, room temperature, CDCl$_3$)

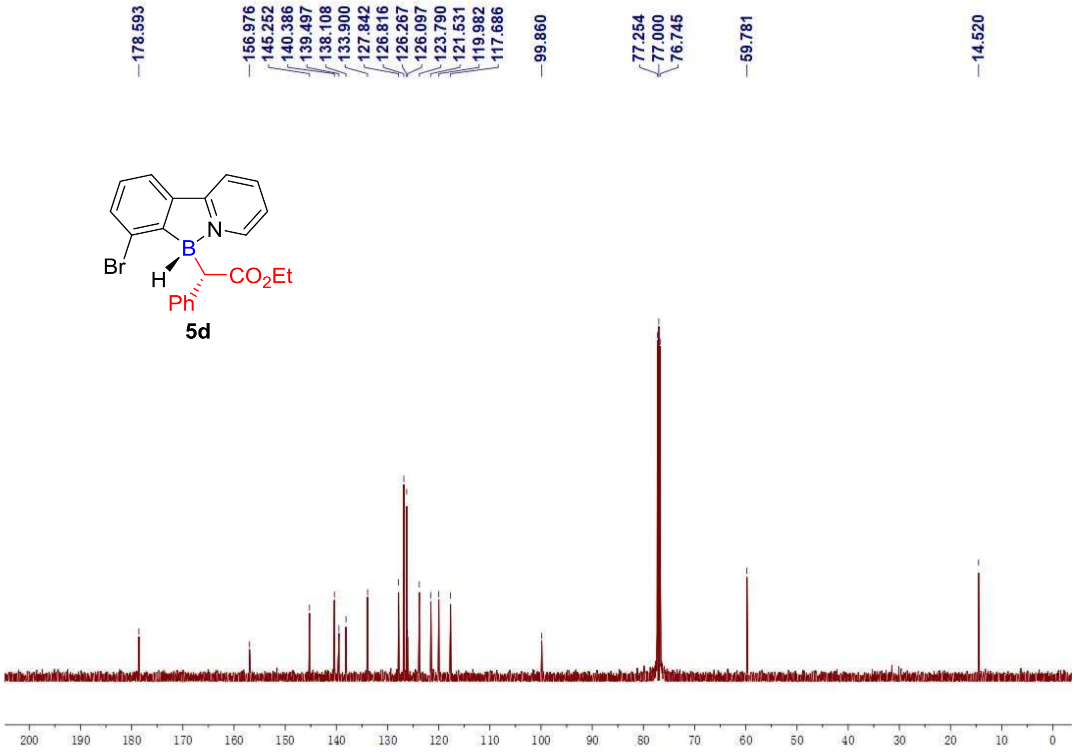

Supplementary Figure 209. ^{13}C NMR spectrum of compound 5d

^{11}B NMR (128 MHz, room temperature, $CDCl_3$)

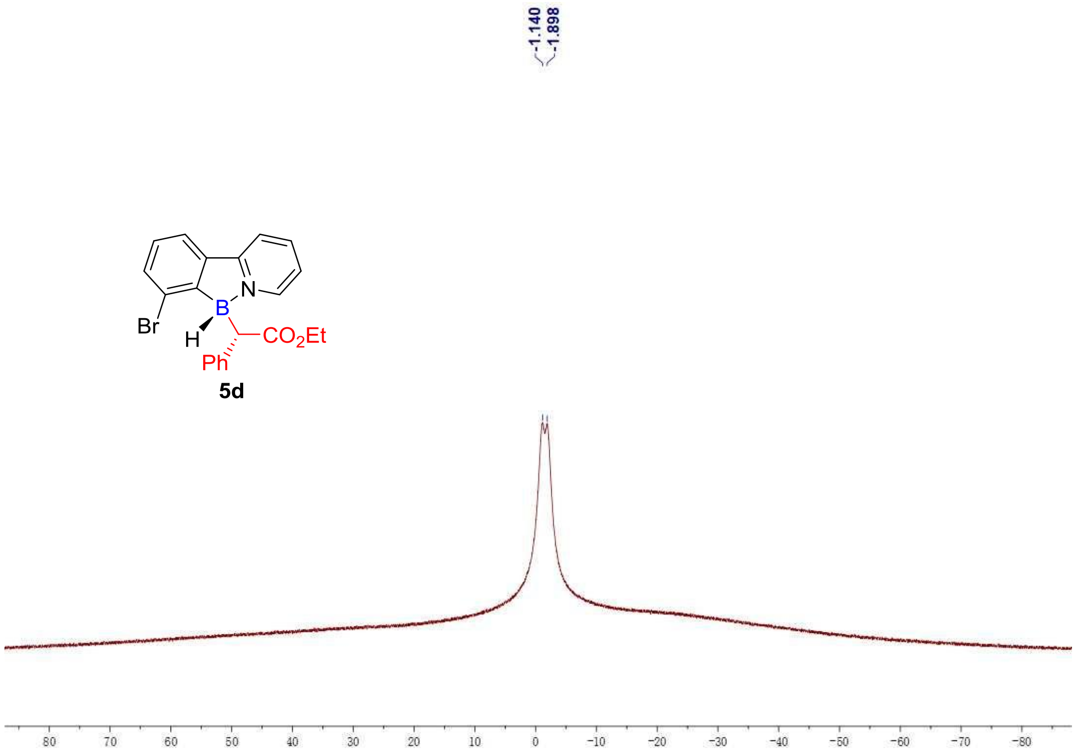

Supplementary Figure 210. ^{11}B NMR spectrum of compound 5d

ethyl(S)-2-(7-chloro-8-fluoro-6H-5l4-benzo[3,4][1,2]azaborolo[1,5-a]pyridin-6-yl)-2-phenylacetate (5e)

^{1}H NMR (400 MHz, room temperature, $CDCl_3$)

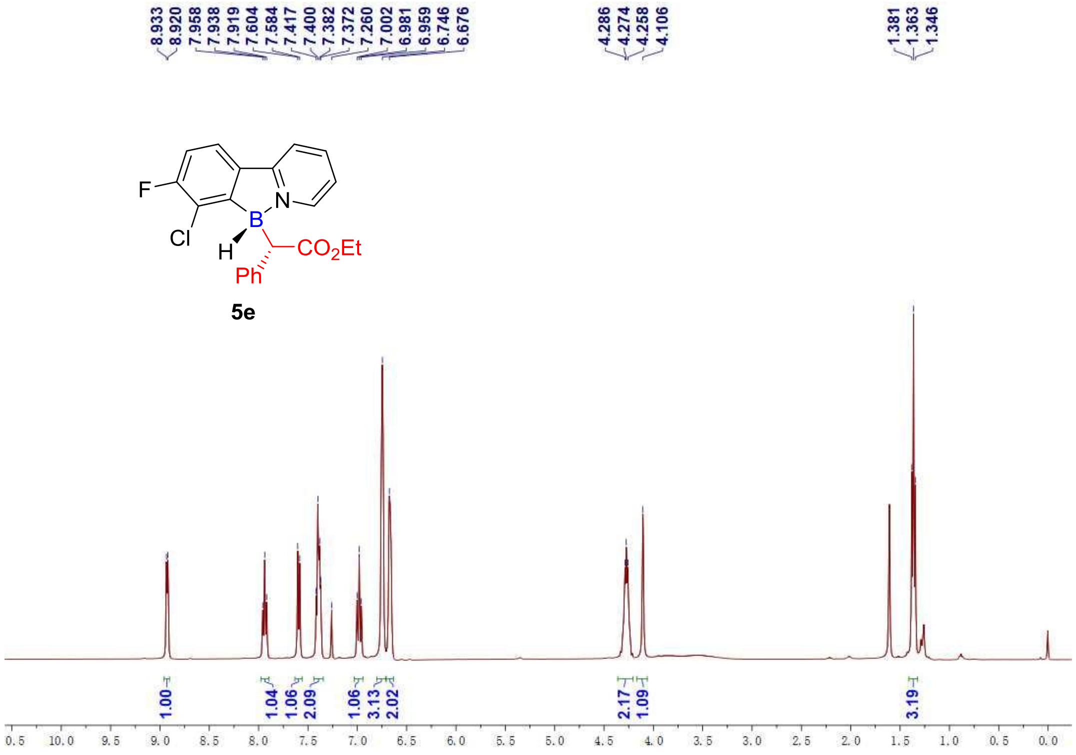

Supplementary Figure 211. ^{1}H NMR spectrum of compound 5e

^{13}C NMR (101 MHz, room temperature, $CDCl_3$)

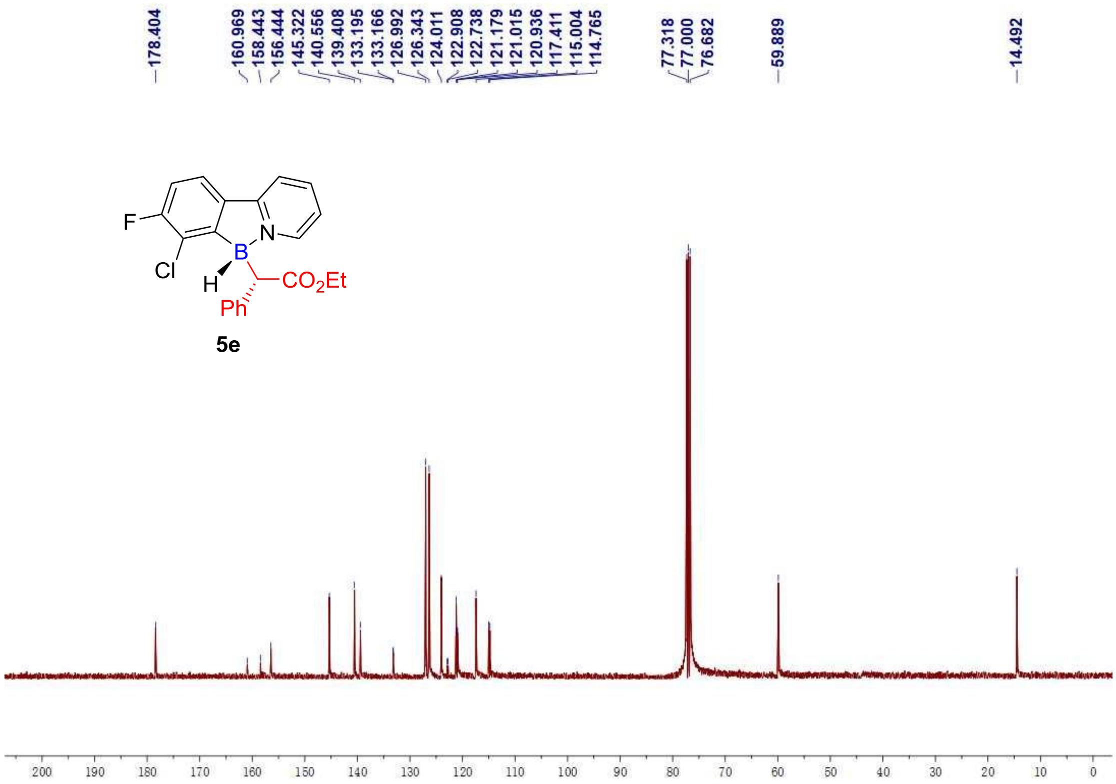

Supplementary Figure 212. ^{13}C **NMR spectrum of compound 5e**

^{11}B NMR (128 MHz, room temperature, $CDCl_3$)

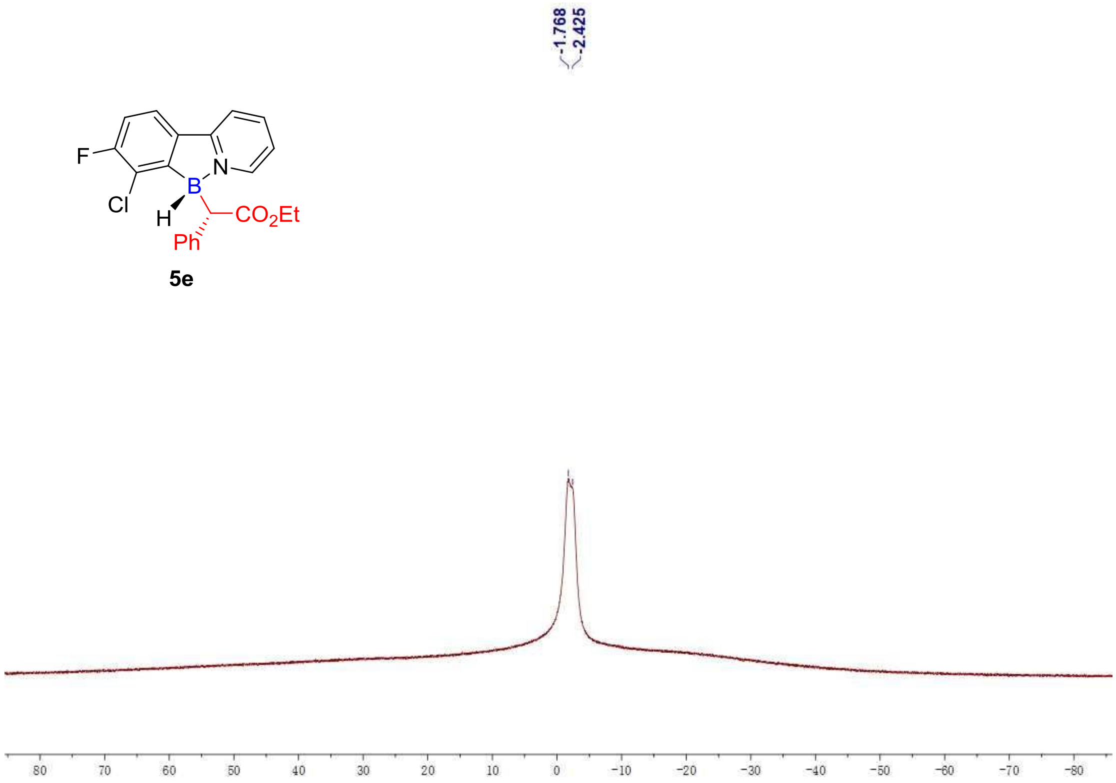

Supplementary Figure 213. ^{11}B **NMR spectrum of compound 5e**

^{19}F NMR (376 MHz, room temperature, $CDCl_3$)

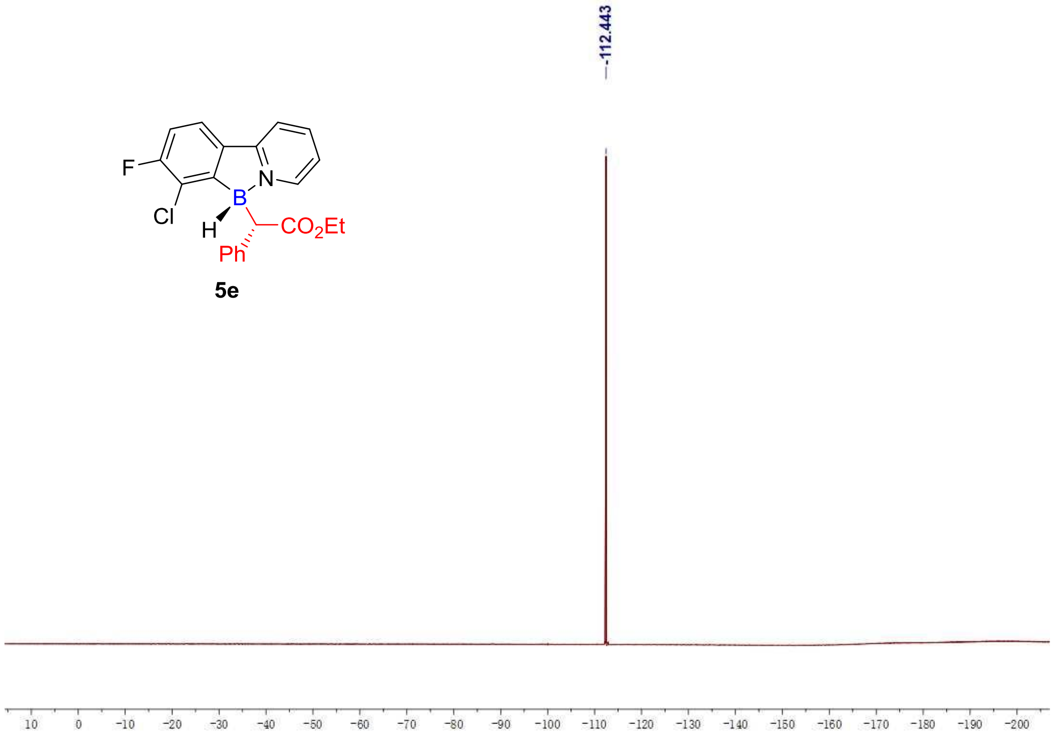

Supplementary Figure 214. ^{19}F NMR spectrum of compound 5e

ethyl (S)-2-(7,8-dichloro-6H-5 ^{4}-benzo[3,4][1,2]azaborolo[1,5-a]pyridin-6-yl)-2-phenylacetate (5f)

^{1}H NMR (400 MHz, room temperature, $CDCl_3$)

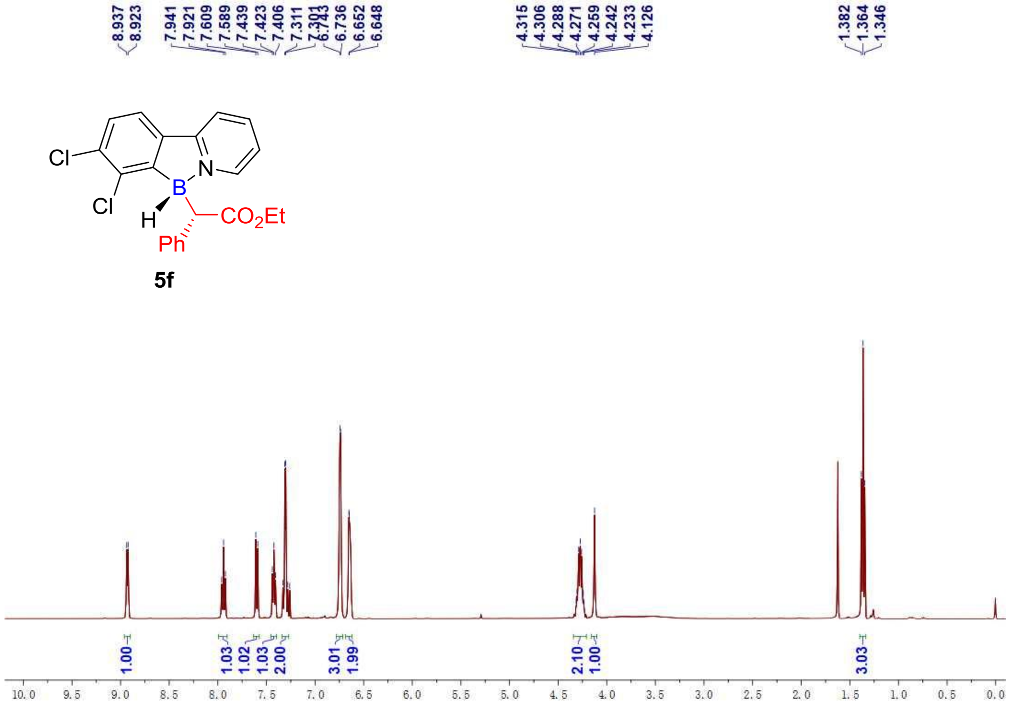

Supplementary Figure 215. ^{1}H NMR spectrum of compound 5f

^{13}C NMR (101 MHz, room temperature, $CDCl_3$)

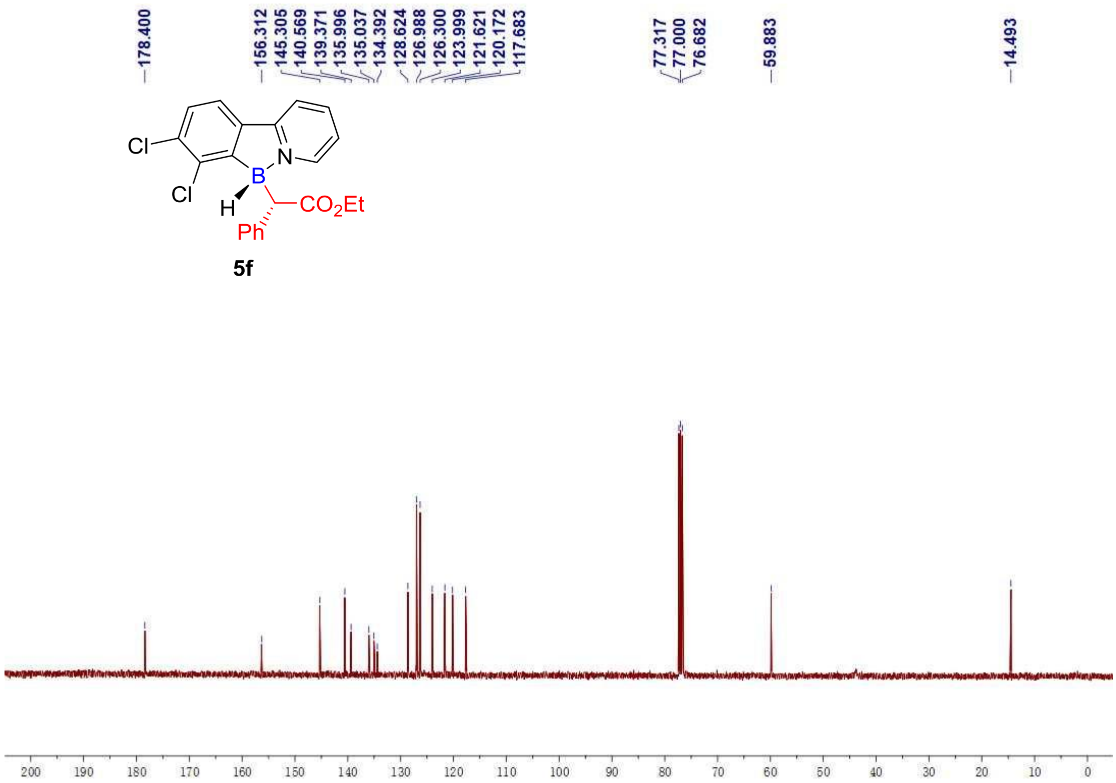

Supplementary Figure 216. ^{13}**C NMR spectrum of compound 5f**

^{11}B NMR (128 MHz, room temperature, $CDCl_3$)

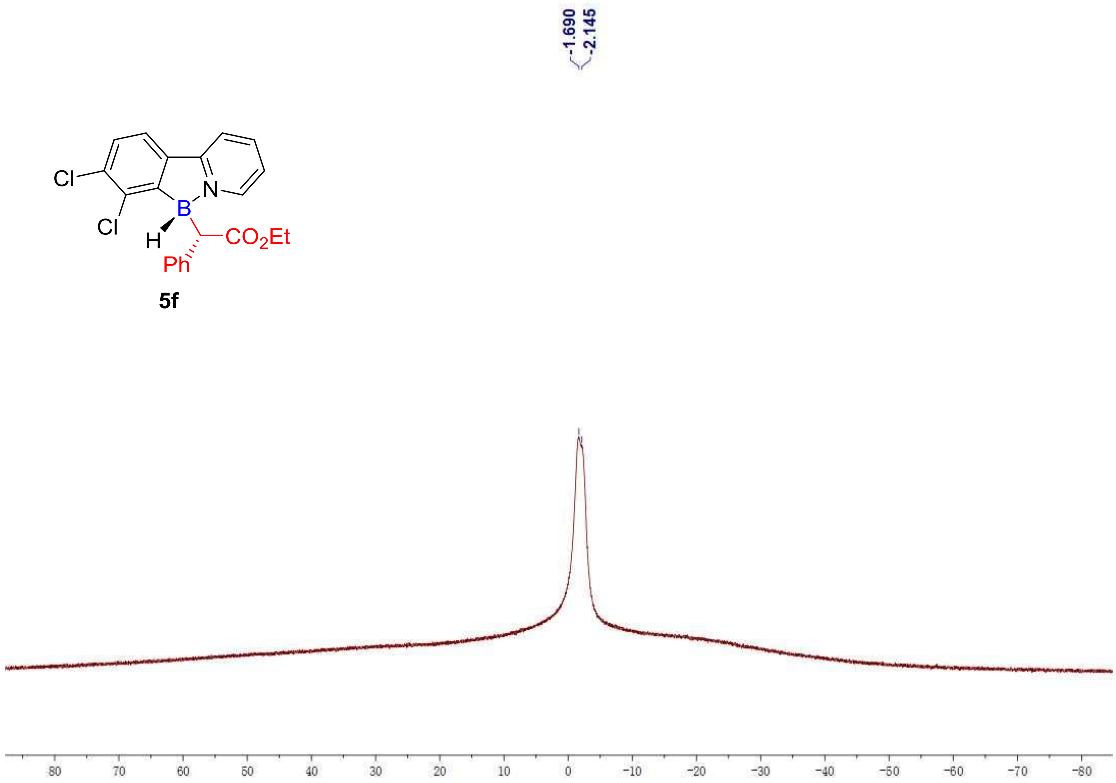

Supplementary Figure 217. ^{11}**B NMR spectrum of compound 5f**

ethyl(S)-2-(7-chloro-8-methyl-6H-5 ^{4}-benzo[3,4][1,2]azaborolo[1,5-a]pyridin-6-yl)-2-phenylacetat-e (5g)

^{1}H NMR (400 MHz, room temperature, CDCl$_3$)

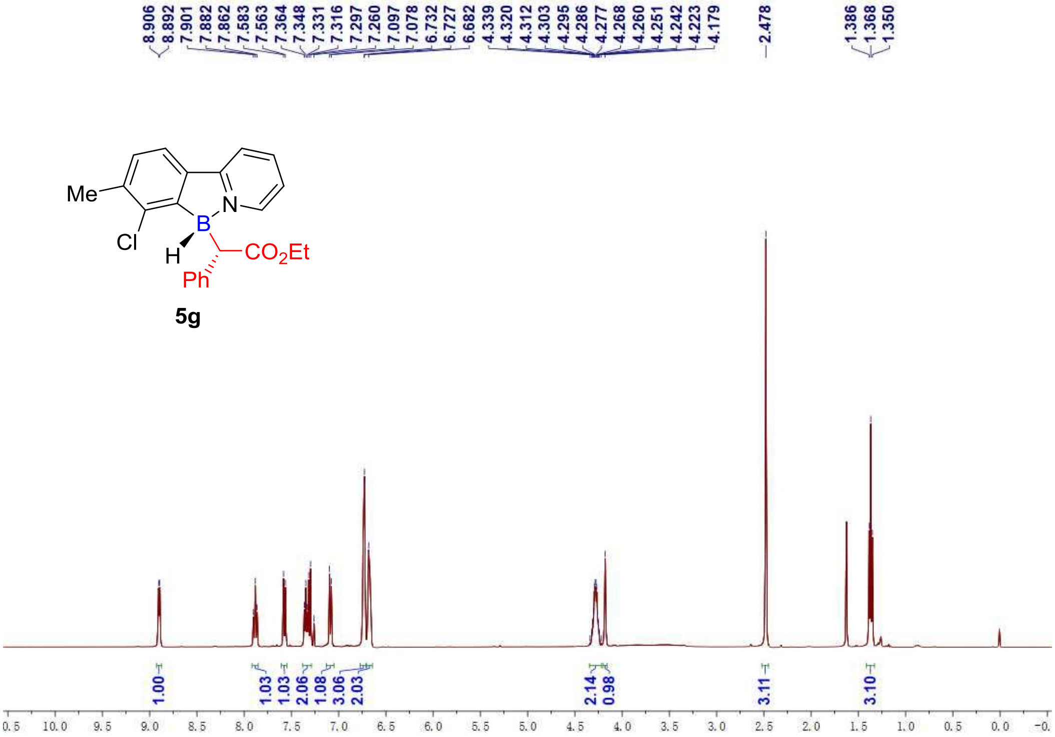

Supplementary Figure 218. ^{1}H NMR spectrum of compound 5g

^{13}C NMR (101 MHz, room temperature, CDCl$_3$)

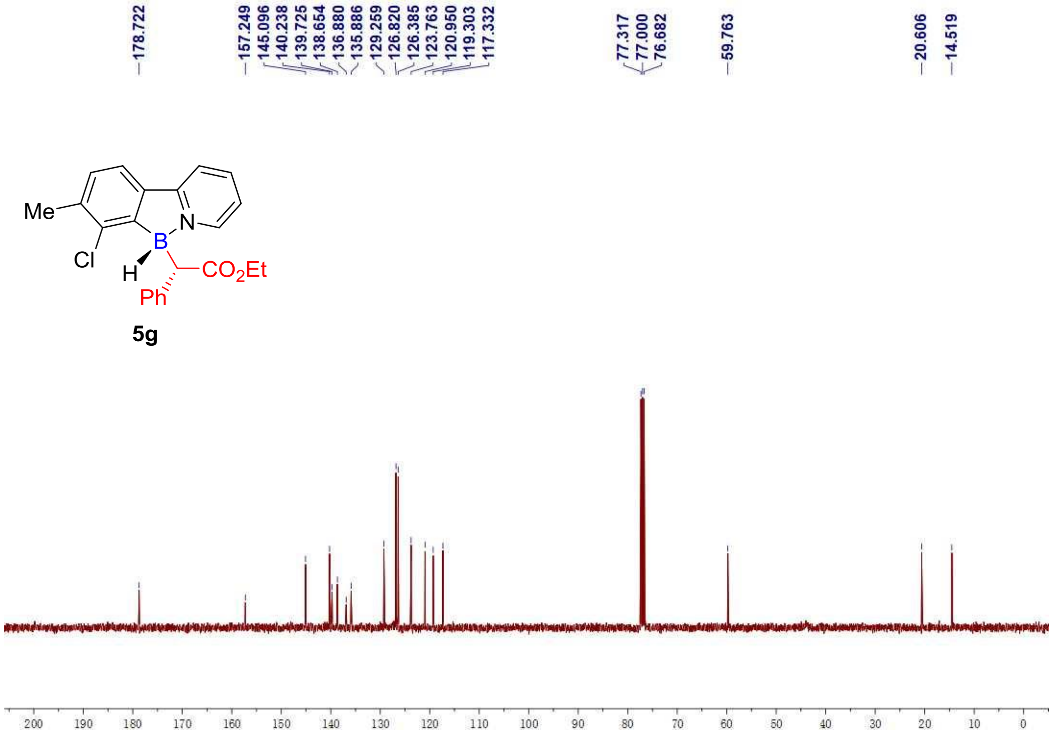

Supplementary Figure 219. ^{13}C NMR spectrum of compound 5g

^{11}B NMR (128 MHz, room temperature, $CDCl_3$)

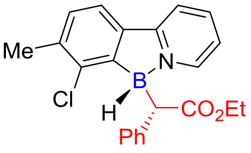

5g

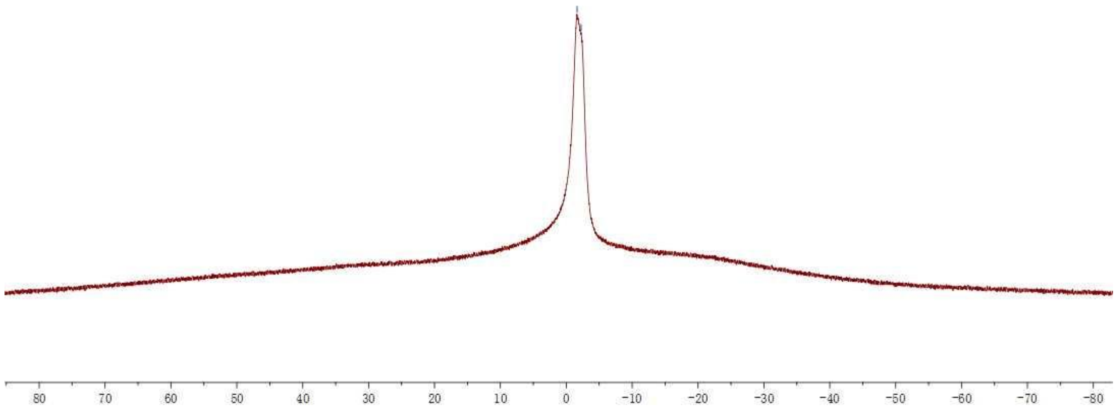

Supplementary Figure 220. ^{11}B NMR spectrum of compound 5g

ethyl(*S*)-2-(7,9-dichloro-(*R*)6H-5 ^{4}-benzo[3,4][1,2]azaborolo[1,5-a]pyridin-6-yl)-2-phenylacetate (5h)

^{1}H NMR NMR (500 MHz, room temperature, $CDCl_3$)

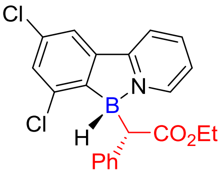

5h

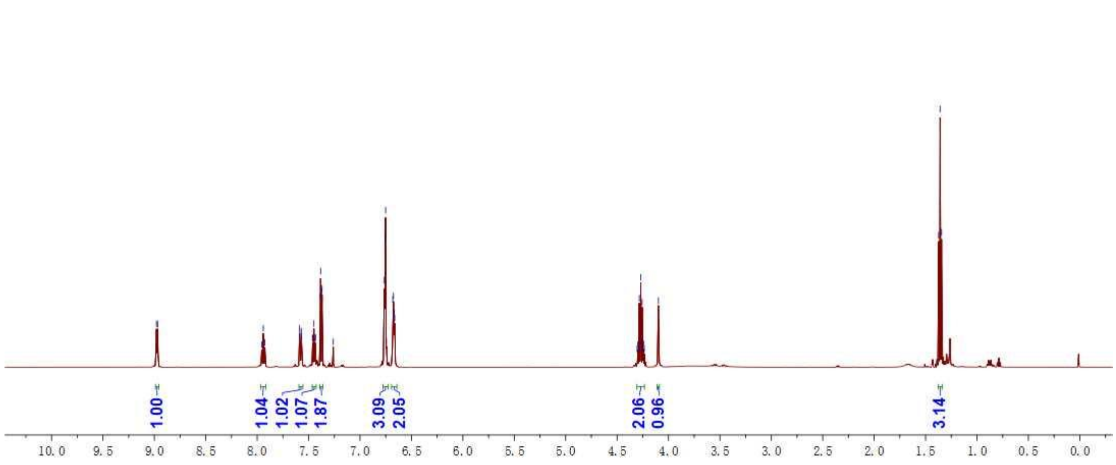

Supplementary Figure 221. ^{1}H NMR spectrum of compound 5h

^{13}C NMR (126 MHz, room temperature, $CDCl_3$)

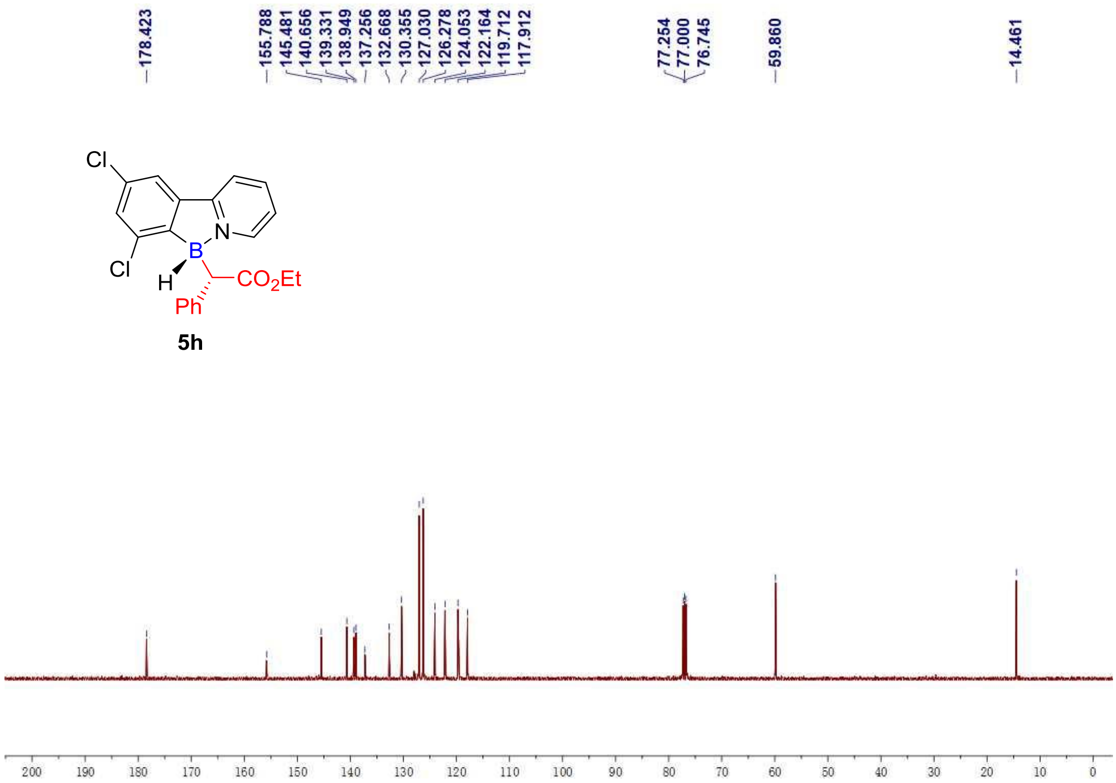

Supplementary Figure 222. ^{13}C NMR spectrum of compound 5h

^{11}B NMR (128 MHz, room temperature, $CDCl_3$)

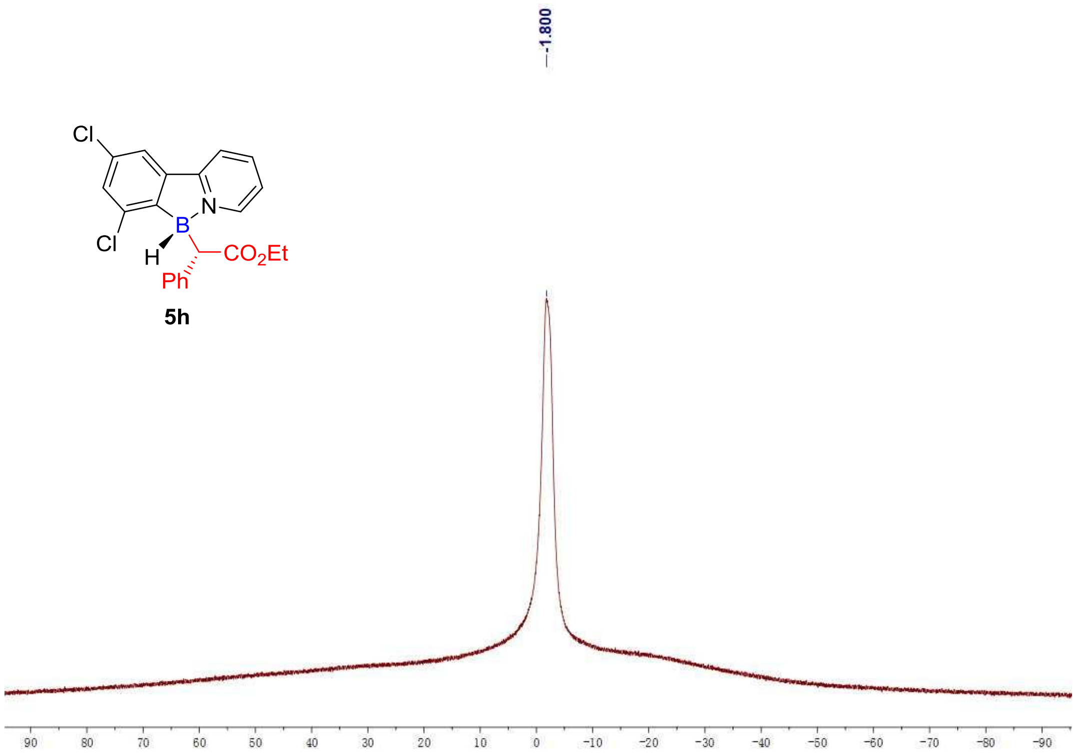

Supplementary Figure 223. ^{11}B NMR spectrum of compound 5h

ethyl (S)-2-(7,10-dichloro-6H-5 ^{4}-benzo[3,4][1,2]azaborolo[1,5-a]pyridin-6-yl)-2-phenylacetate (5i)

^{1}H NMR (400 MHz, room temperature, $CDCl_3$)

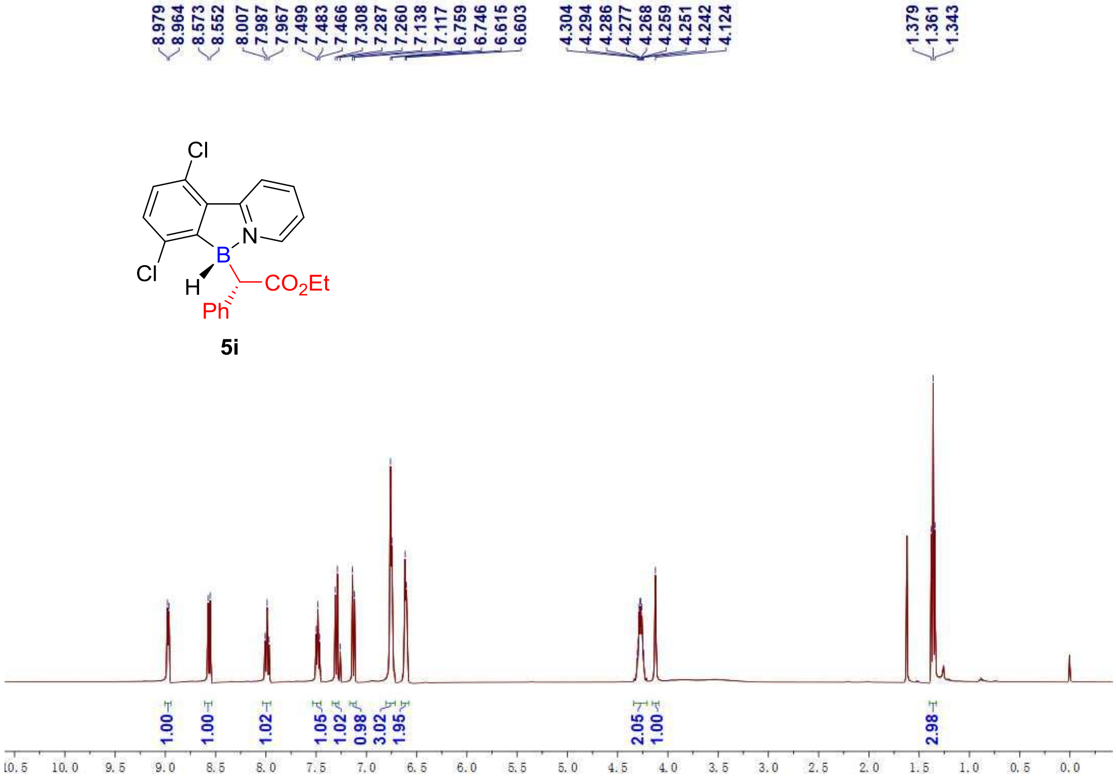

Supplementary Figure 224. ^{1}H NMR spectrum of compound 5i

^{13}C NMR (101 MHz, room temperature, $CDCl_3$)

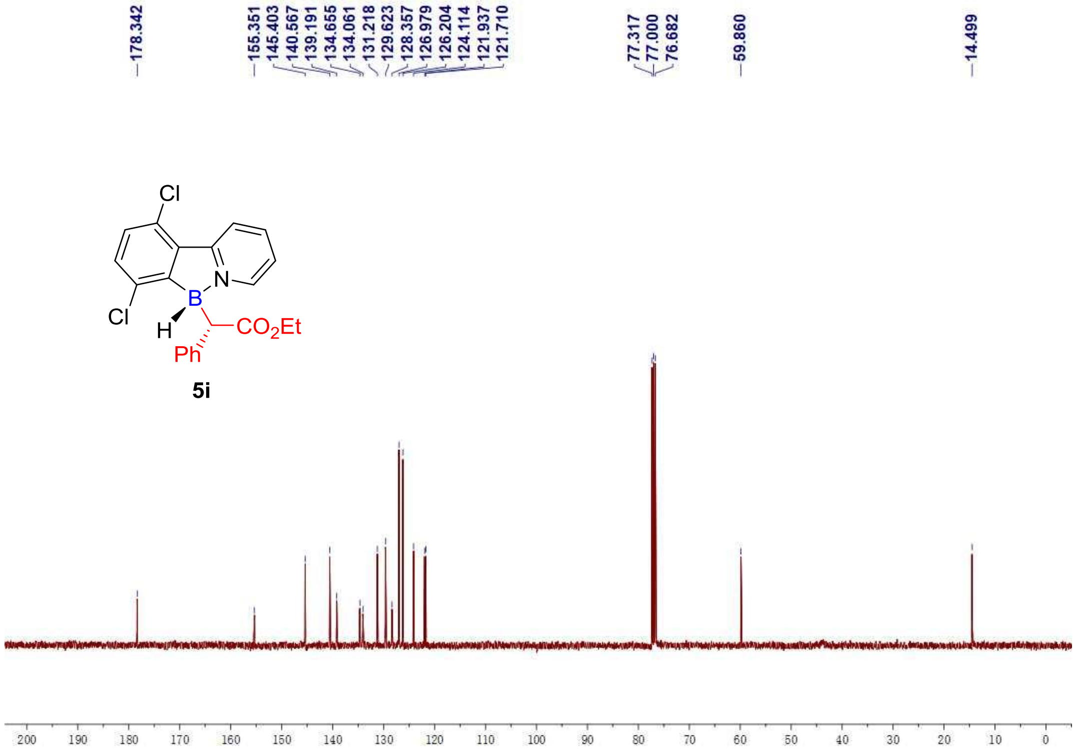

Supplementary Figure 225. ^{13}C NMR spectrum of compound 5i

^{11}B NMR (128 MHz, room temperature, $CDCl_3$)

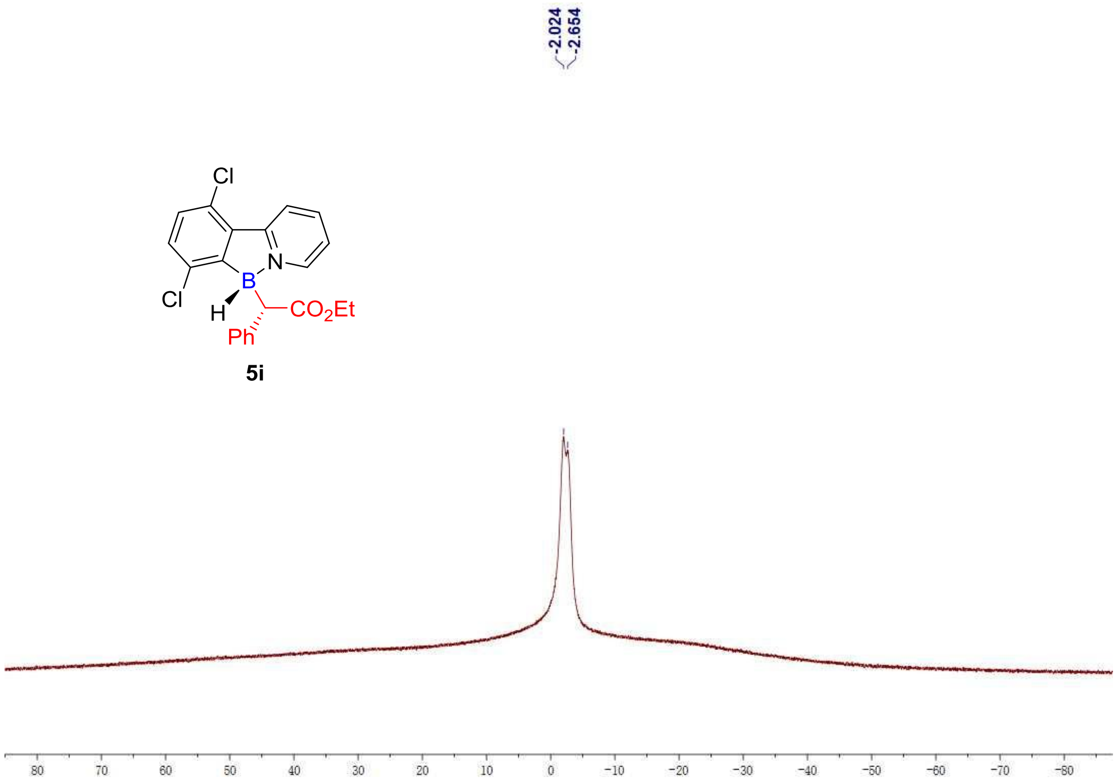

Supplementary Figure 226. ^{11}B NMR spectrum of compound 5i

ethyl(S)-2-(7-chloro-10-fluoro-6H-5l4-benzo[3,4][1,2]azaborolo[1,5-a]pyridin-6-yl)-2-phenylaceta-te (5j)

^{1}H NMR (400 MHz, room temperature, $CDCl_3$)

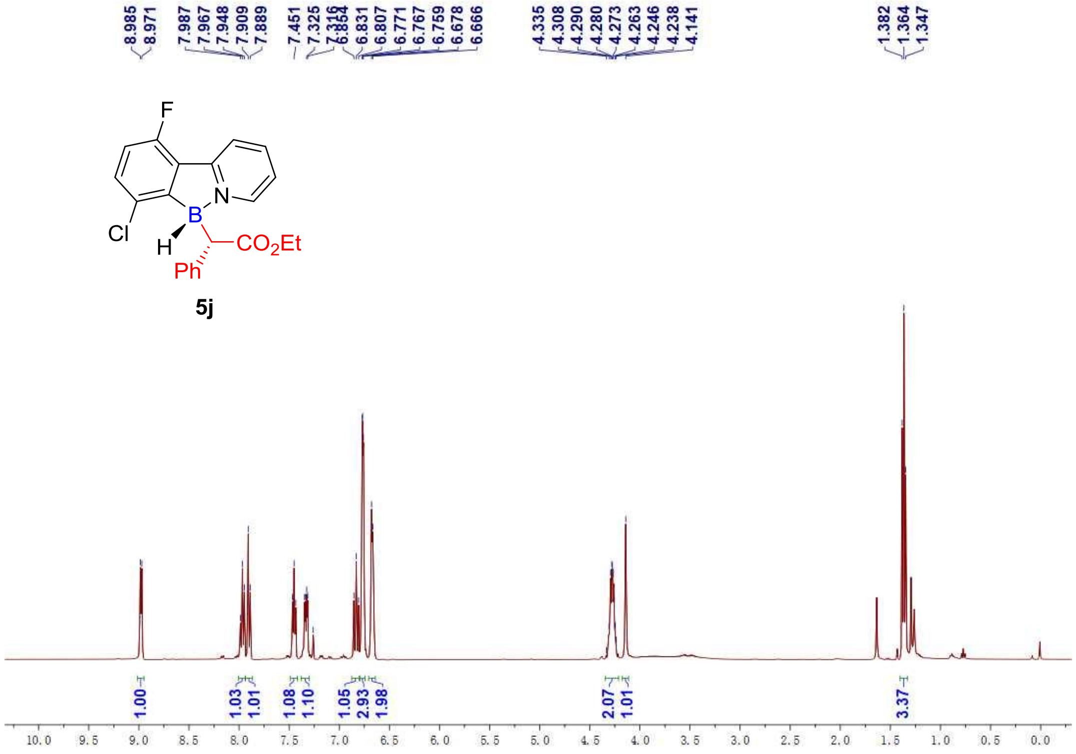

Supplementary Figure 227. ^{1}H NMR spectrum of compound 5j

^{13}C NMR (101 MHz, room temperature, $CDCl_3$)

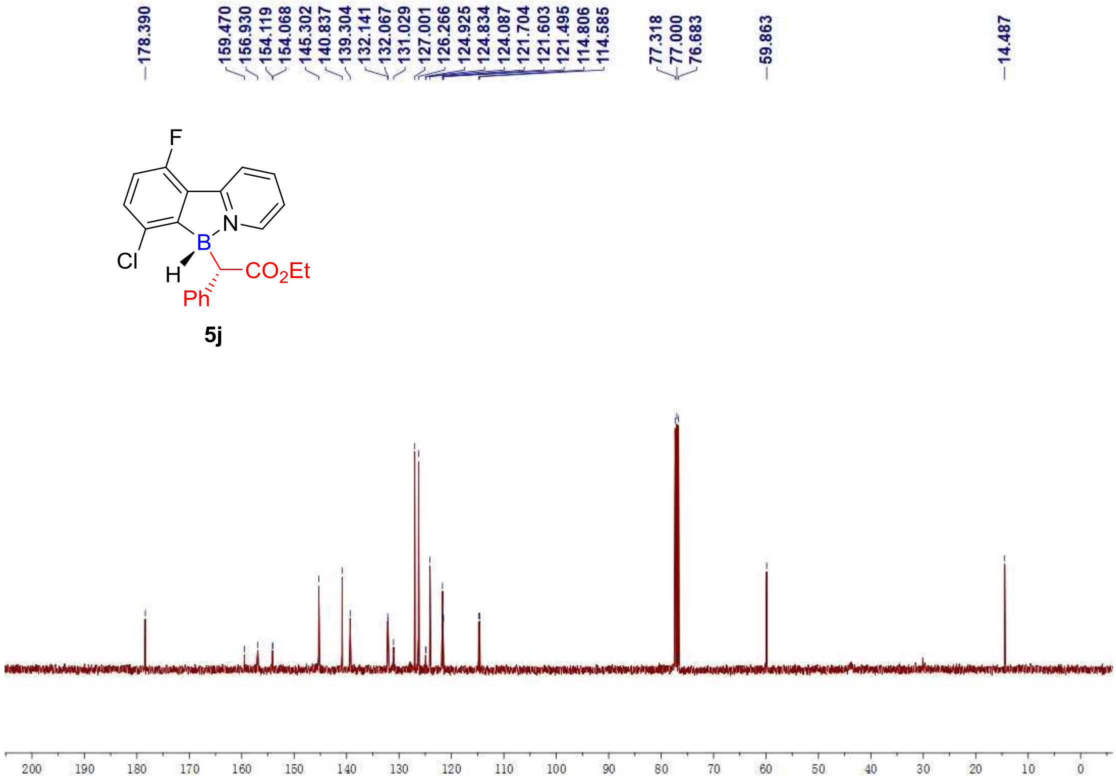

Supplementary Figure 228. ^{13}**C NMR spectrum of compound 5j**

^{11}B NMR (128 MHz, room temperature, $CDCl_3$)

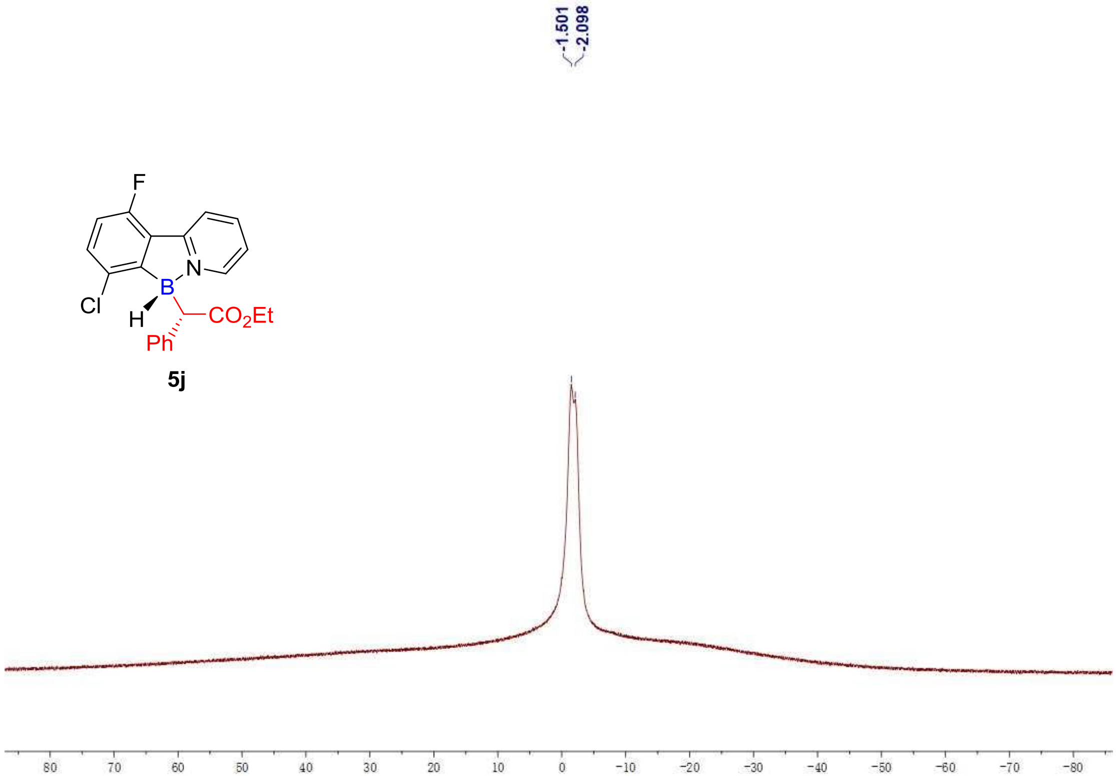

Supplementary Figure 229. ^{11}**B NMR spectrum of compound 5j**

^{19}F NMR (376 MHz, room temperature, $CDCl_3$)

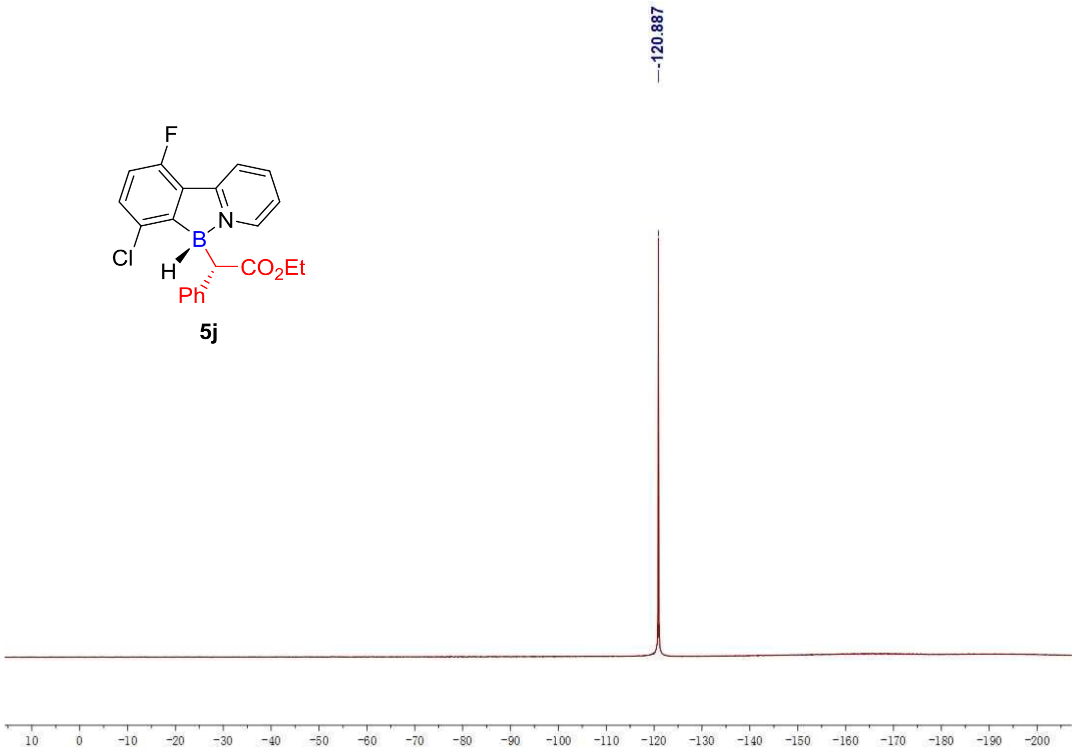

Supplementary Figure 230. ^{19}F NMR spectrum of compound 5j

ethyl(S)-2-(7-chloro-10-methyl-6H-5 ^{4}-benzo[3,4][1,2]azaborolo[1,5-a]pyridin-6-yl)-2-phenylace t-ate (5k)

^{1}H NMR (400 MHz, room temperature, $CDCl_3$)

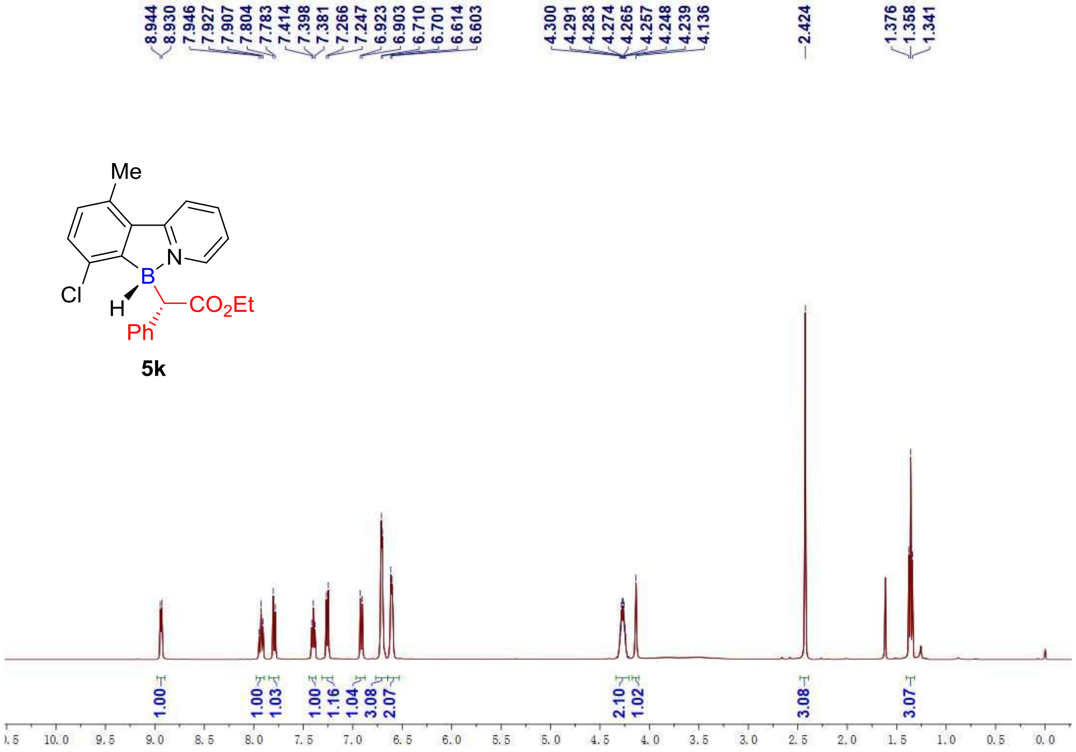

Supplementary Figure 231. ^{1}H NMR spectrum of compound 5k

^{13}C NMR (101 MHz, room temperature, $CDCl_3$)

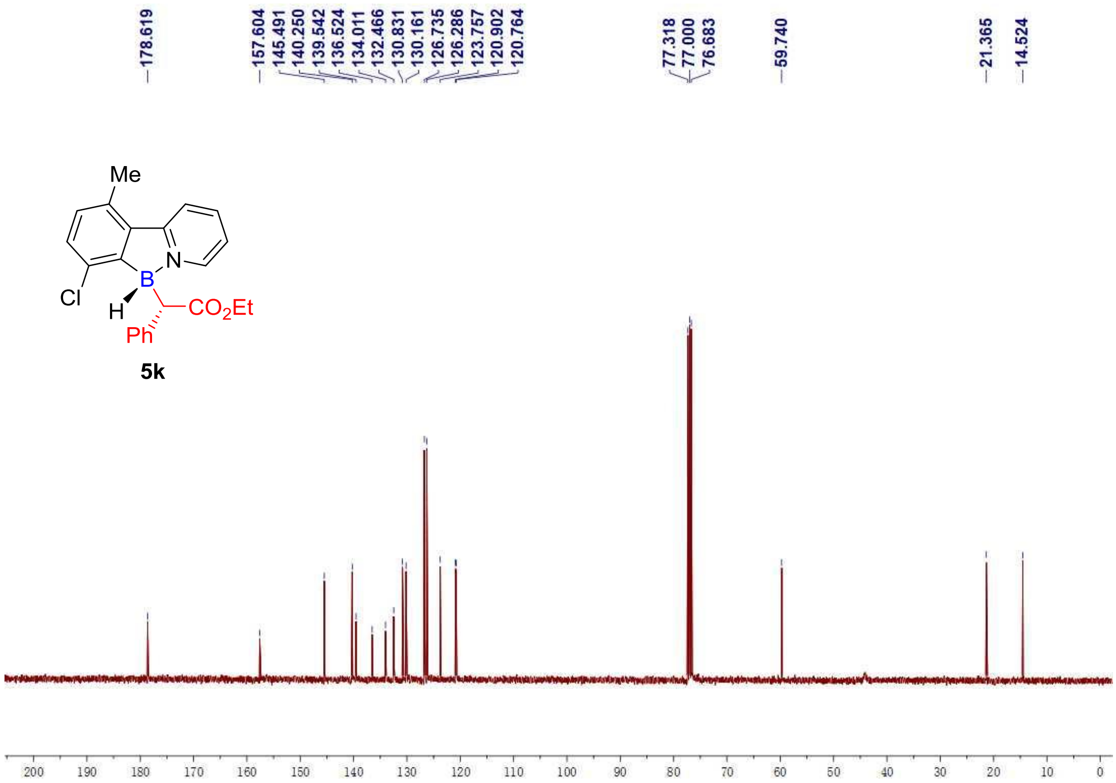

Supplementary Figure 232. ^{13}**C NMR spectrum of compound 5k**

^{11}B NMR (128 MHz, room temperature, $CDCl_3$)

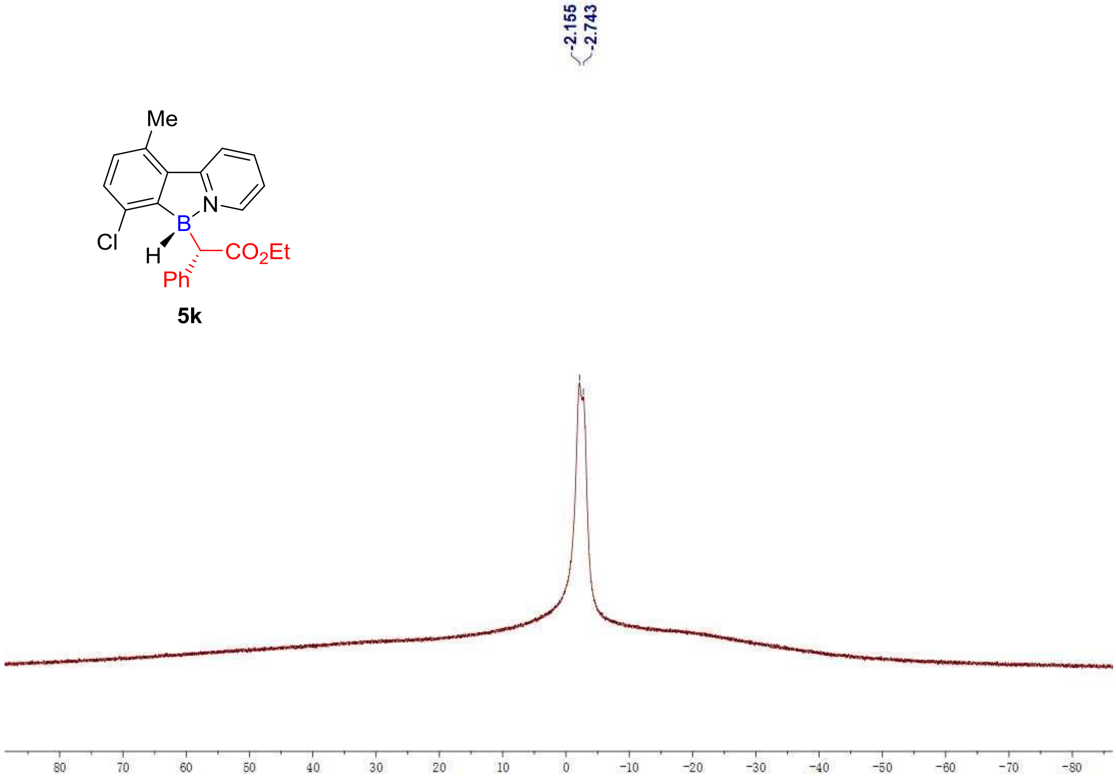

Supplementary Figure 233. ^{11}**B NMR spectrum of compound 5k**

ethyl(S)-2-phenyl-2-(7-(trifluoromethoxy)-6H-5 ^{4}-benzo[3,4][1,2]azaborolo[1,5-a]pyridin-6-y-l)acetate (5l)

^{1}H NMR (400 MHz, room temperature, $CDCl_3$)

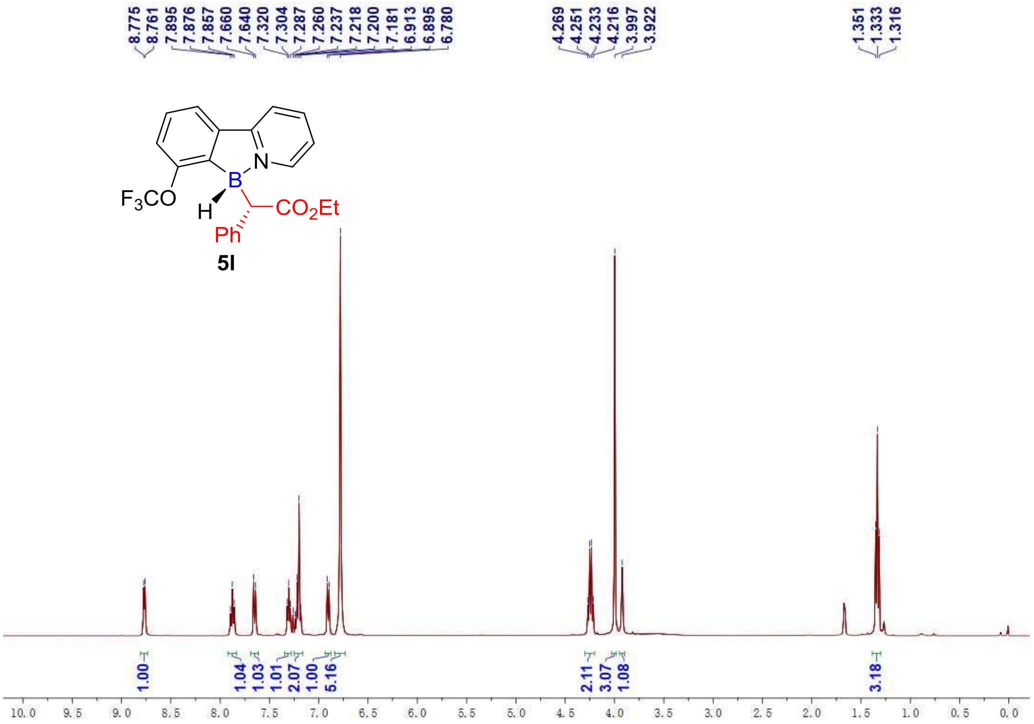

Supplementary Figure 234. ^{1}H NMR spectrum of compound 5l

^{13}C NMR (101 MHz, room temperature, $CDCl_3$)

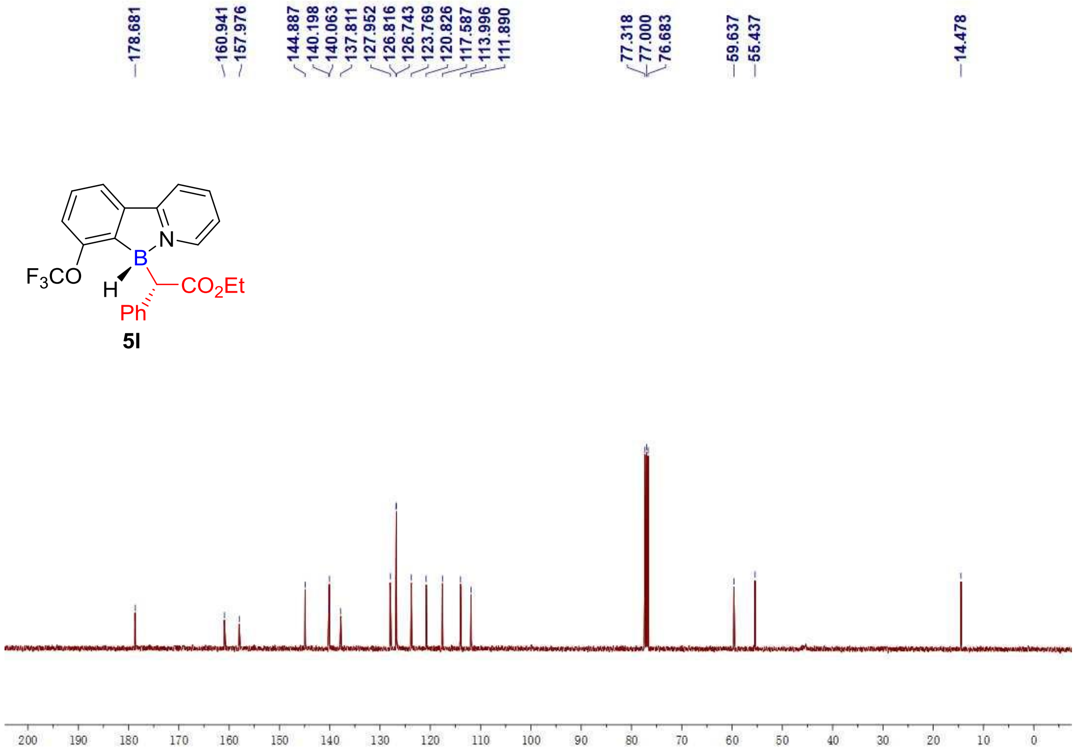

Supplementary Figure 235. ^{13}C NMR spectrum of compound 5l

^{11}B NMR (128 MHz, room temperature, $CDCl_3$)

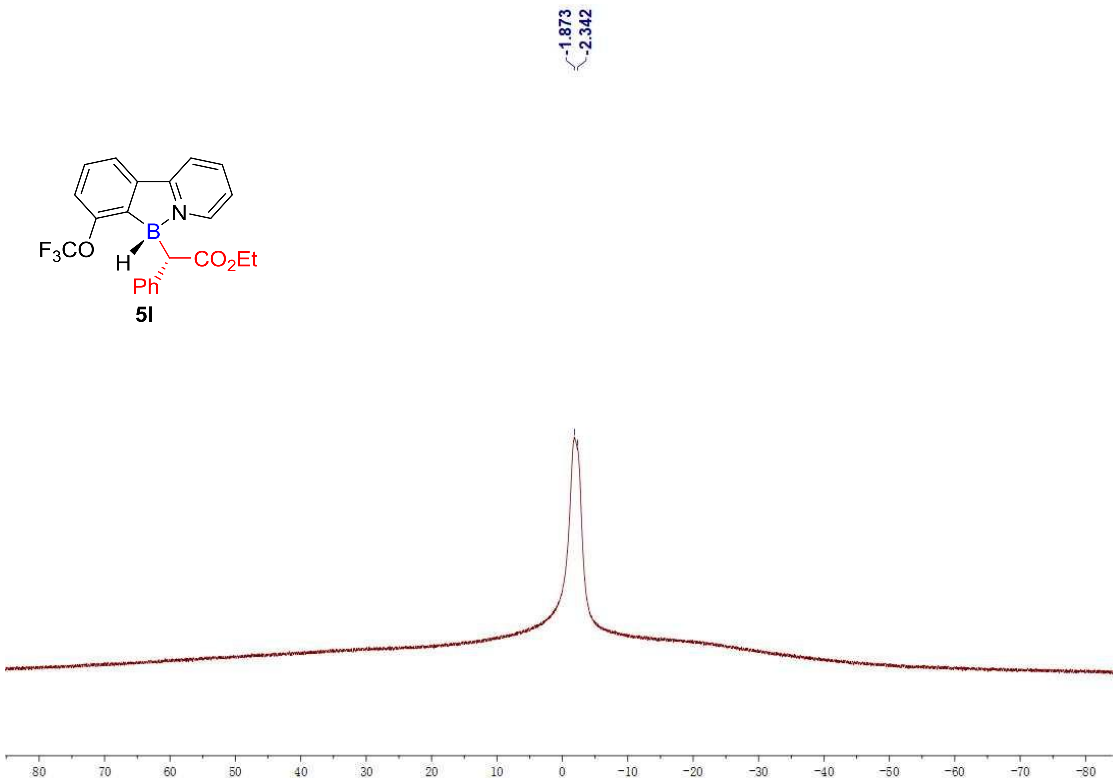

Supplementary Figure 236. ^{11}B NMR spectrum of compound 5l

^{19}F NMR (376 MHz, room temperature, $CDCl_3$)

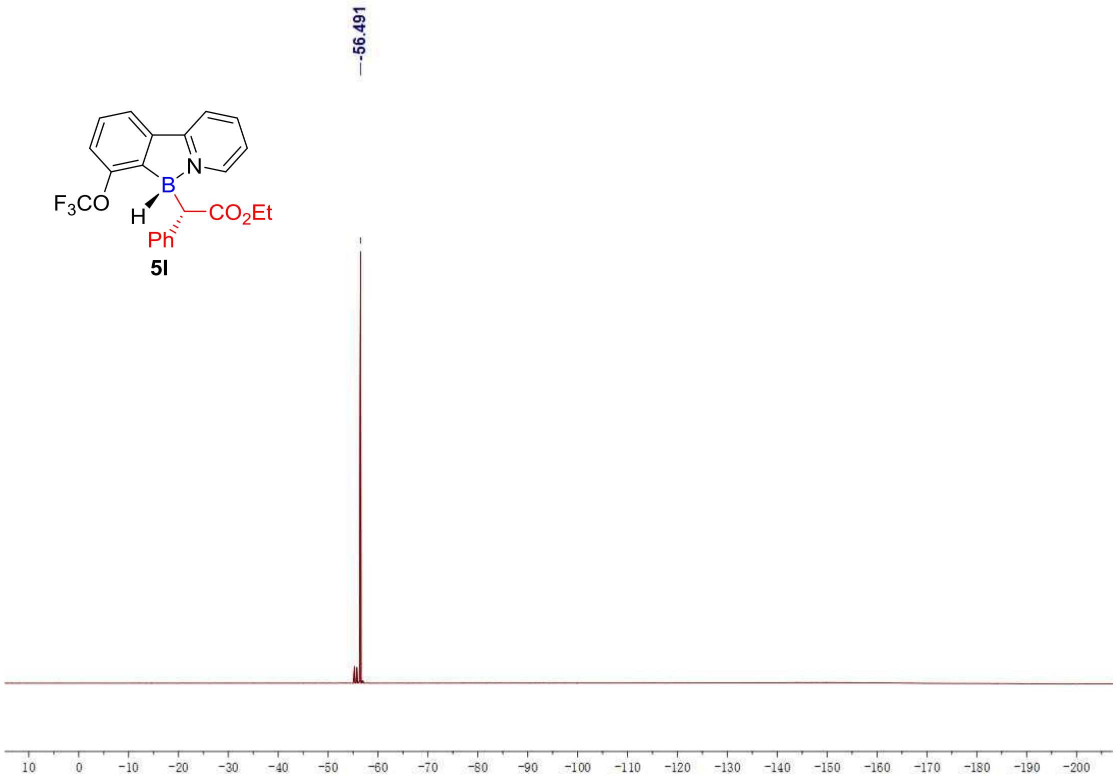

Supplementary Figure 237. ^{19}F NMR spectrum of compound 5l

ethyl(*S*)-2-(7,9-dimethyl-(*R*)6H-5 ^{4}-benzo[3,4][1,2]azaborolo[1,5-a]pyridin-6-yl)-2-phenylacetate (5m)

^{1}H NMR (400 MHz, room temperature, CDCl$_3$)

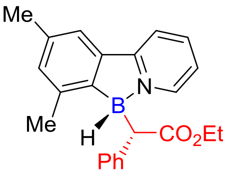

5m

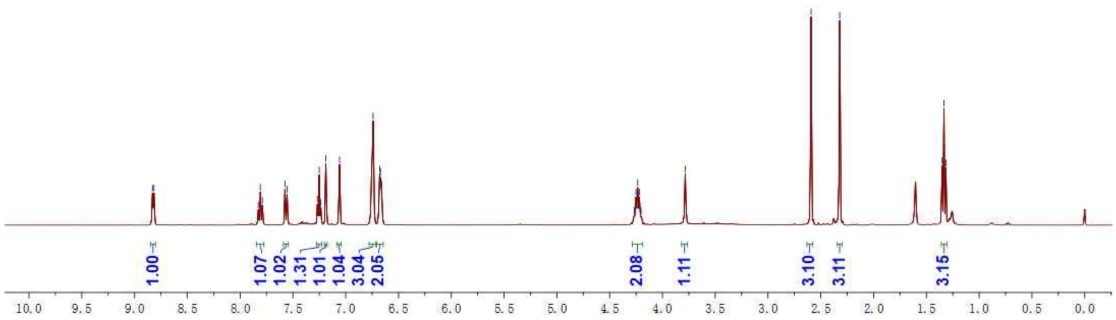

Supplementary Figure 238. ^{1}H NMR spectrum of compound 5m

^{13}C NMR (126 MHz, room temperature, CDCl$_3$)

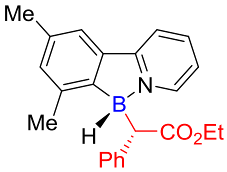

5m

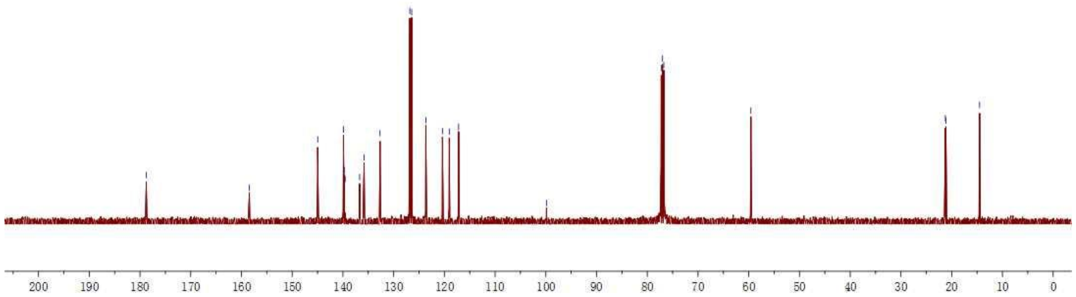

Supplementary Figure 239. ^{13}C NMR spectrum of compound 5m

^{11}B NMR (128 MHz, room temperature, $CDCl_3$)

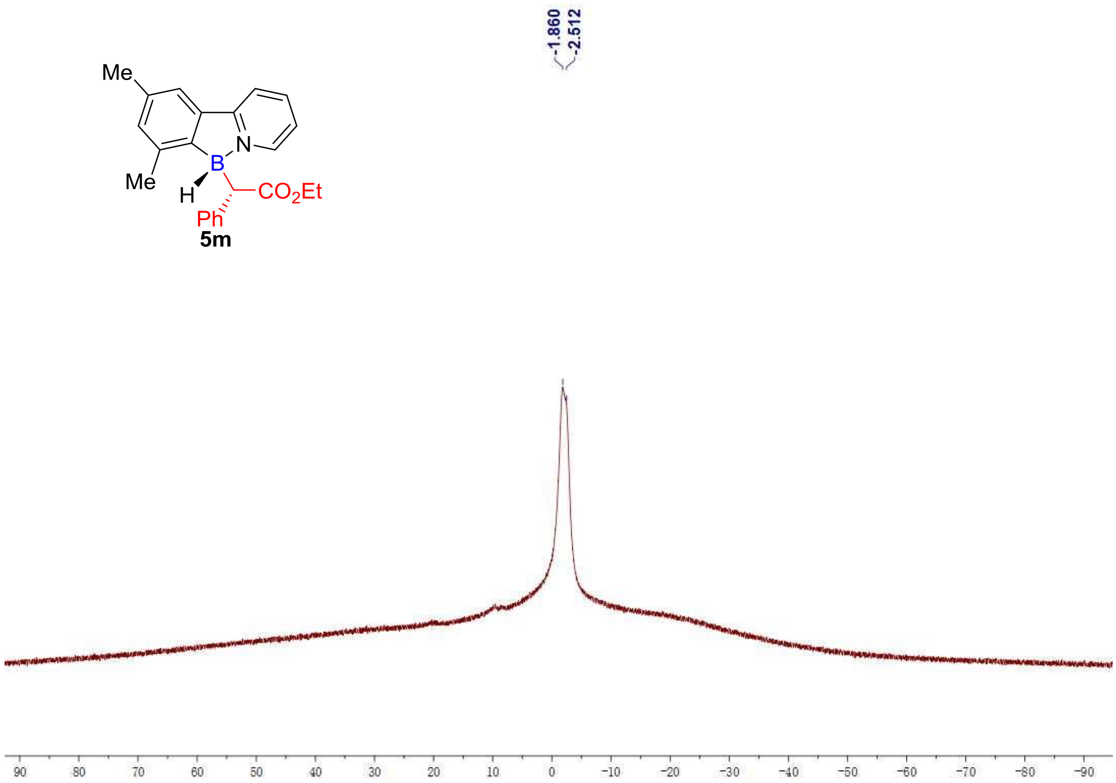

Supplementary Figure 240. ^{11}B NMR spectrum of compound 5m

ethyl (*S*)-2-((*R*)9H-8 ^{4}-thieno[2',3':3,4][1,2]azaborolo[1,5-a]pyridin-9-yl)-2-phenylacetate (5n)

^{1}H NMR (500 MHz, room temperature, $CDCl_3$)

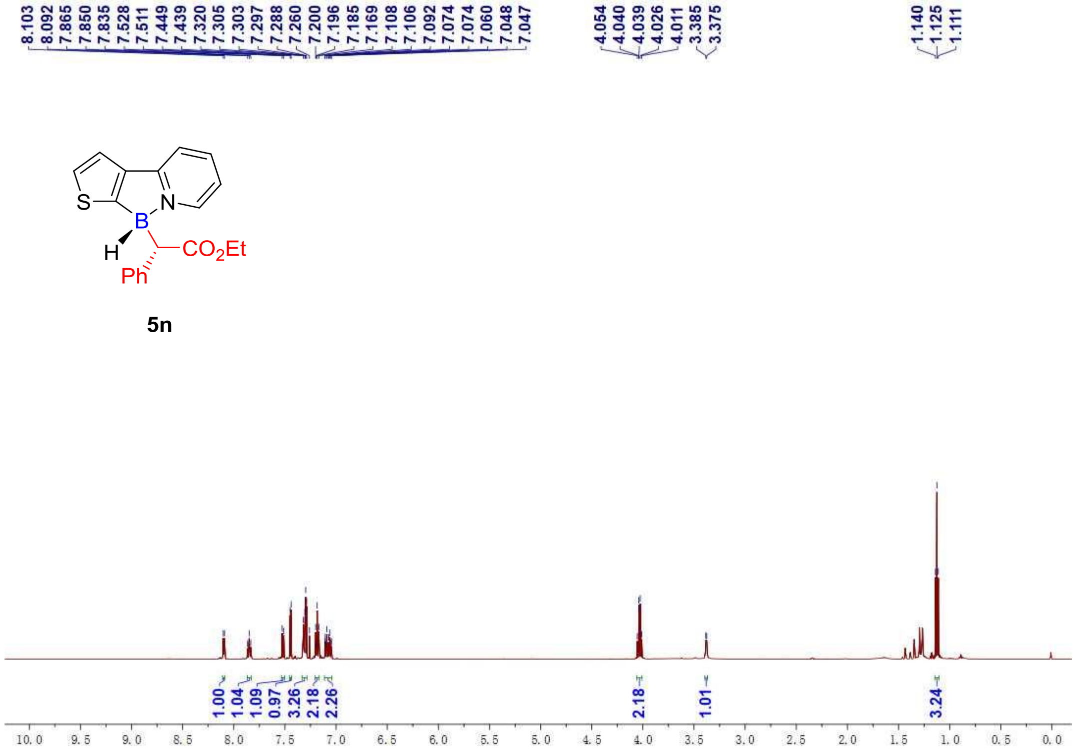

Supplementary Figure 241. ^{1}H NMR spectrum of compound 5n

^{13}C NMR (126 MHz, room temperature, $CDCl_3$)

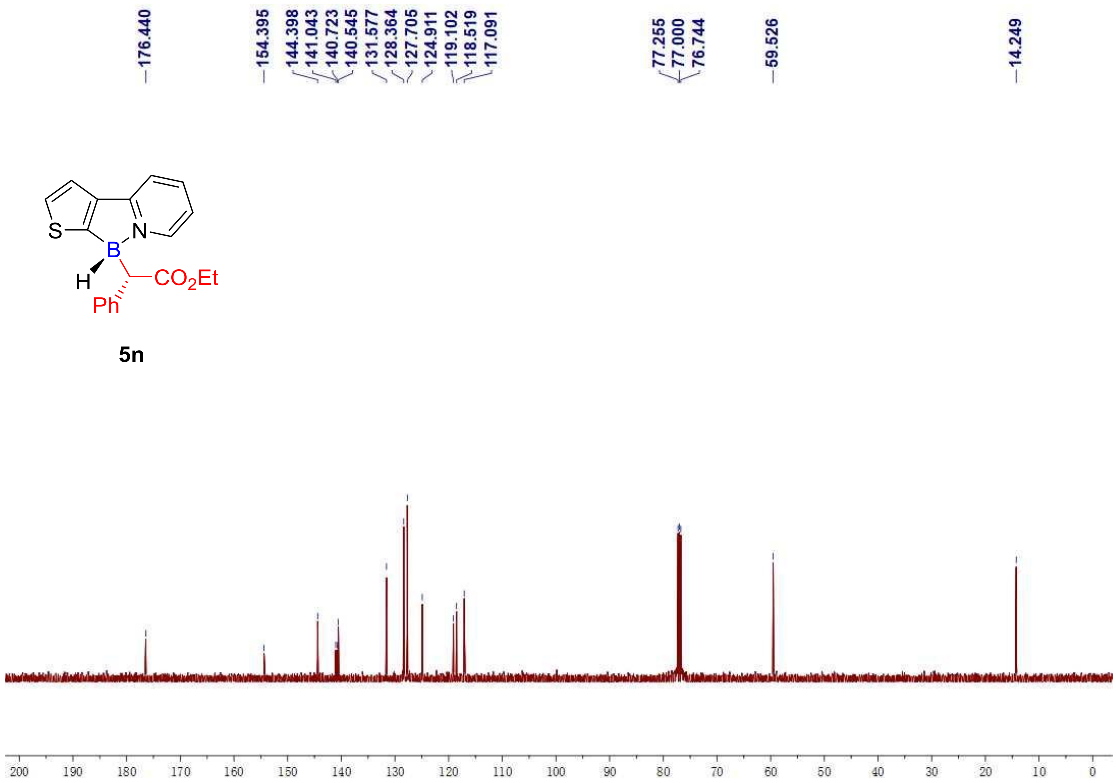

Supplementary Figure 242. ^{13}C NMR spectrum of compound 5n

^{11}B NMR (128 MHz, room temperature, $CDCl_3$)

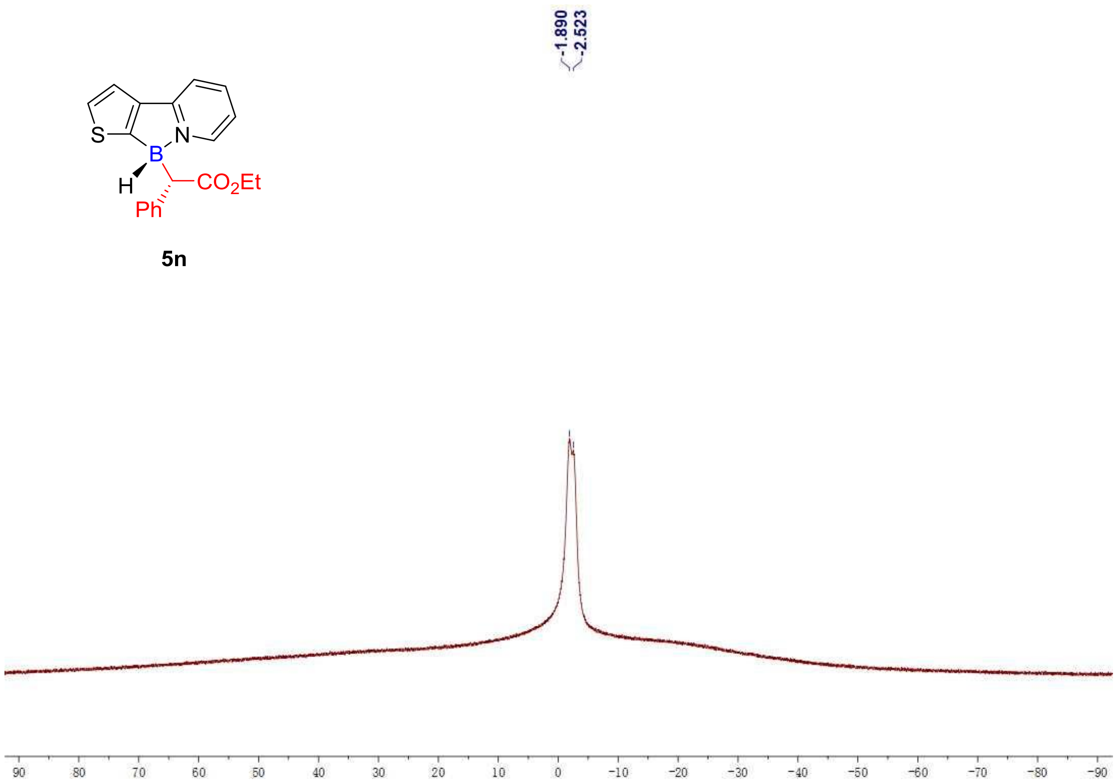

Supplementary Figure 243. ^{11}B NMR spectrum of compound 5n

ethyl(*S*)-2-((*R*)6H-7 ^{4}-benzo[4',5']thieno[2',3':3,4][1,2]azaborolo[1,5-a]pyridin-6-yl)-2-phenylacetate (5o)

^{1}H NMR (400 MHz, room temperature, $CDCl_3$)

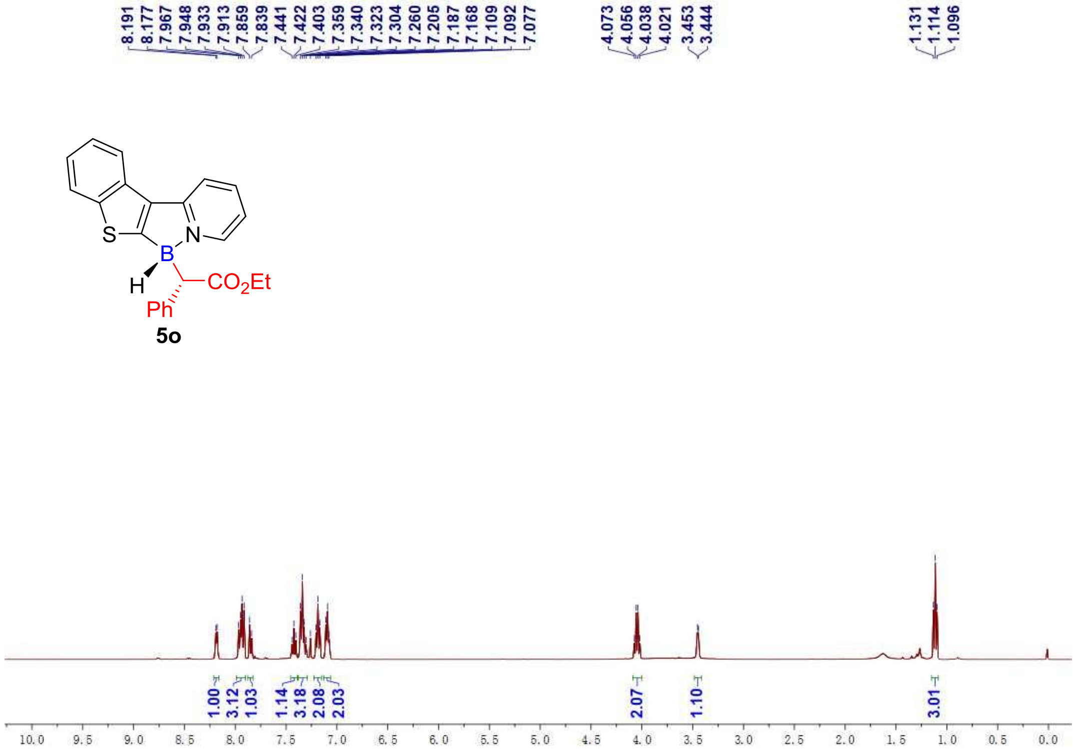

Supplementary Figure 244. ^{1}H NMR spectrum of compound 5o

^{13}C NMR (101 MHz, room temperature, $CDCl_3$)

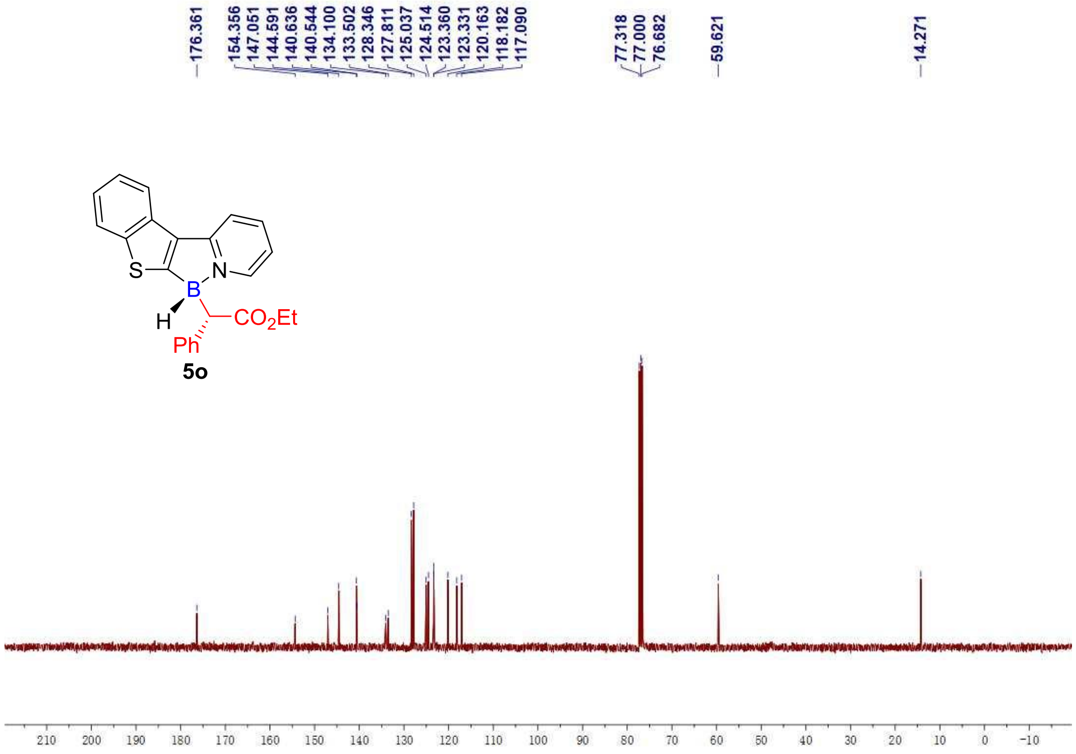

Supplementary Figure 245. ^{13}C NMR spectrum of compound 5o

^{11}B NMR (128 MHz, room temperature, $CDCl_3$)

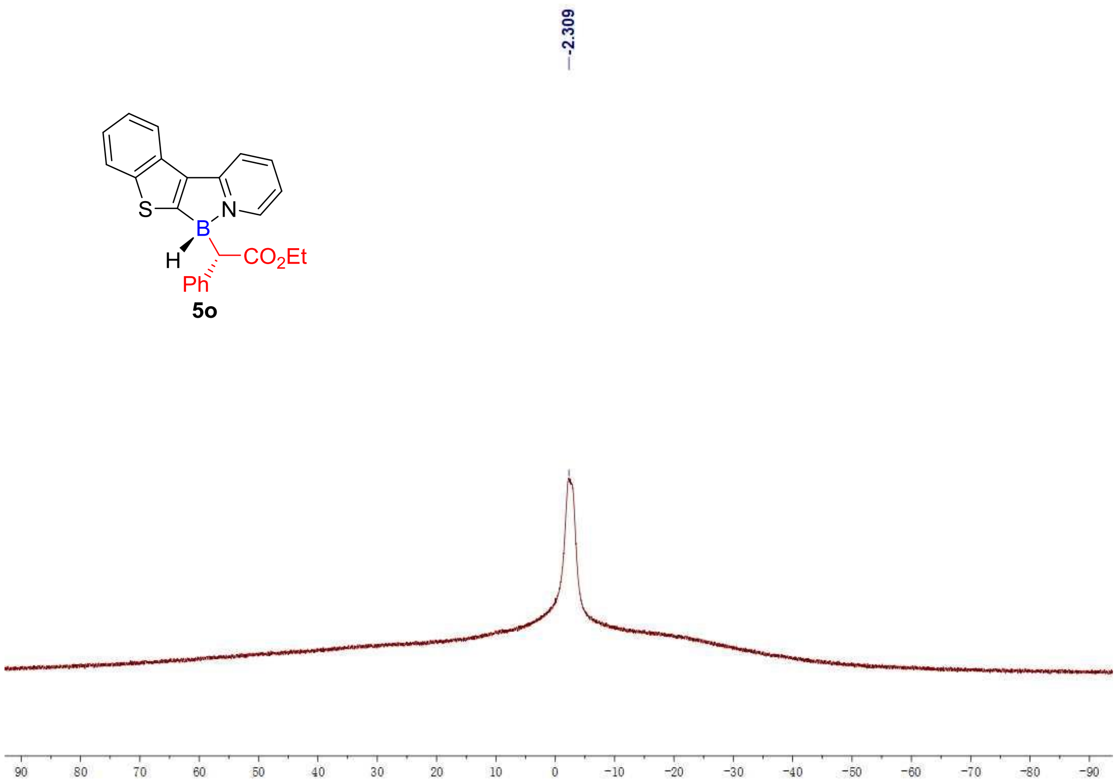

Supplementary Figure 246. ^{11}B NMR spectrum of compound 5o

ethyl (*S*)-2-(1-fluoro-(*R*)6H-5 ^{4}-benzo[3,4][1,2]azaborolo[1,5-a]pyridin-6-yl)-2-phenylacetate (5p)

^{1}H NMR (500 MHz, room temperature, $CDCl_3$)

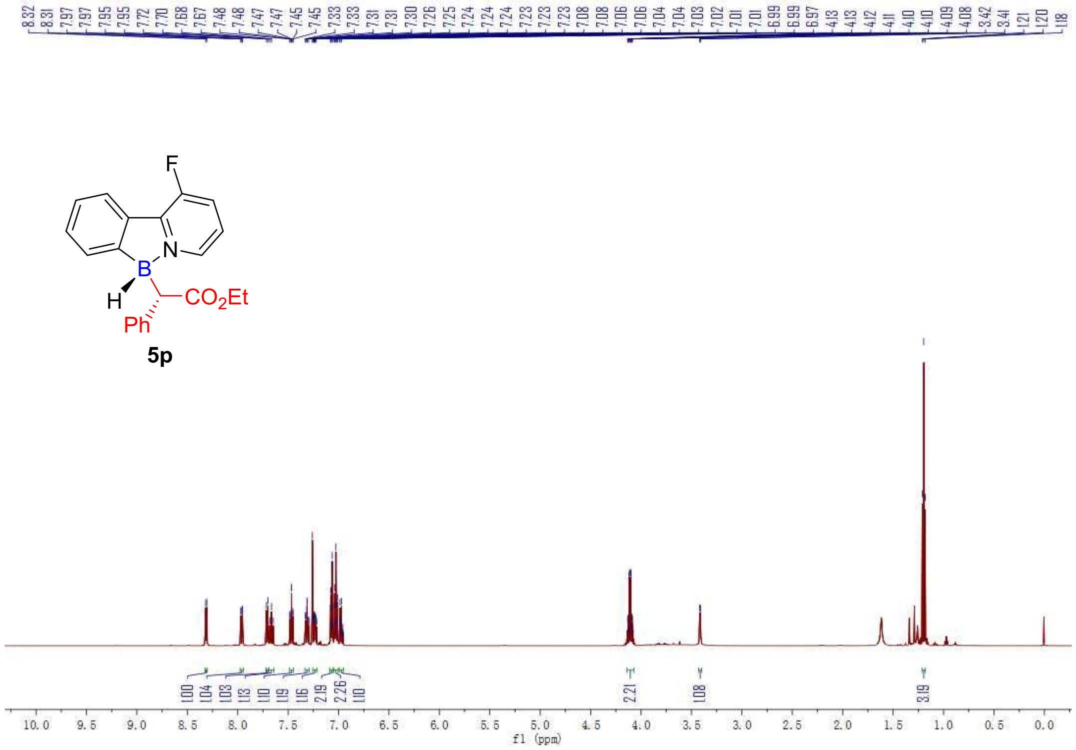

Supplementary Figure 247. ^{1}H NMR spectrum of compound 5p

^{13}C NMR (151 MHz, room temperature, $CDCl_3$)

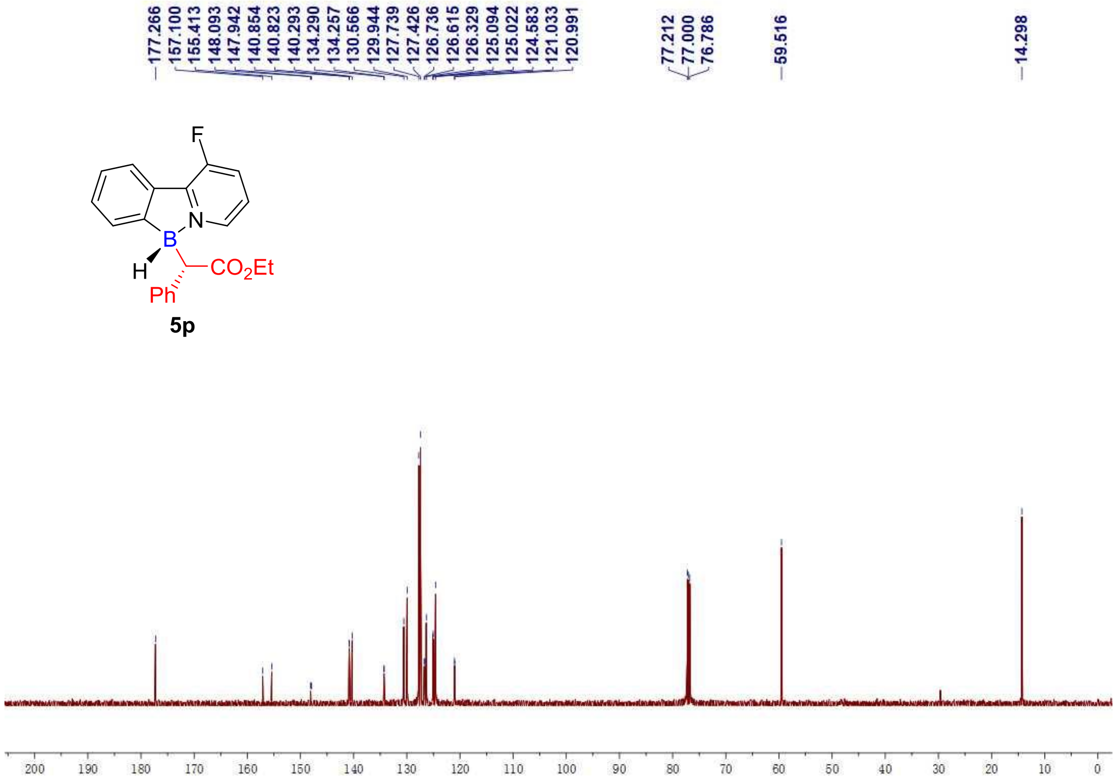

Supplementary Figure 248. ^{13}C NMR spectrum of compound 5p

^{11}B NMR (160 MHz, room temperature, $CDCl_3$)

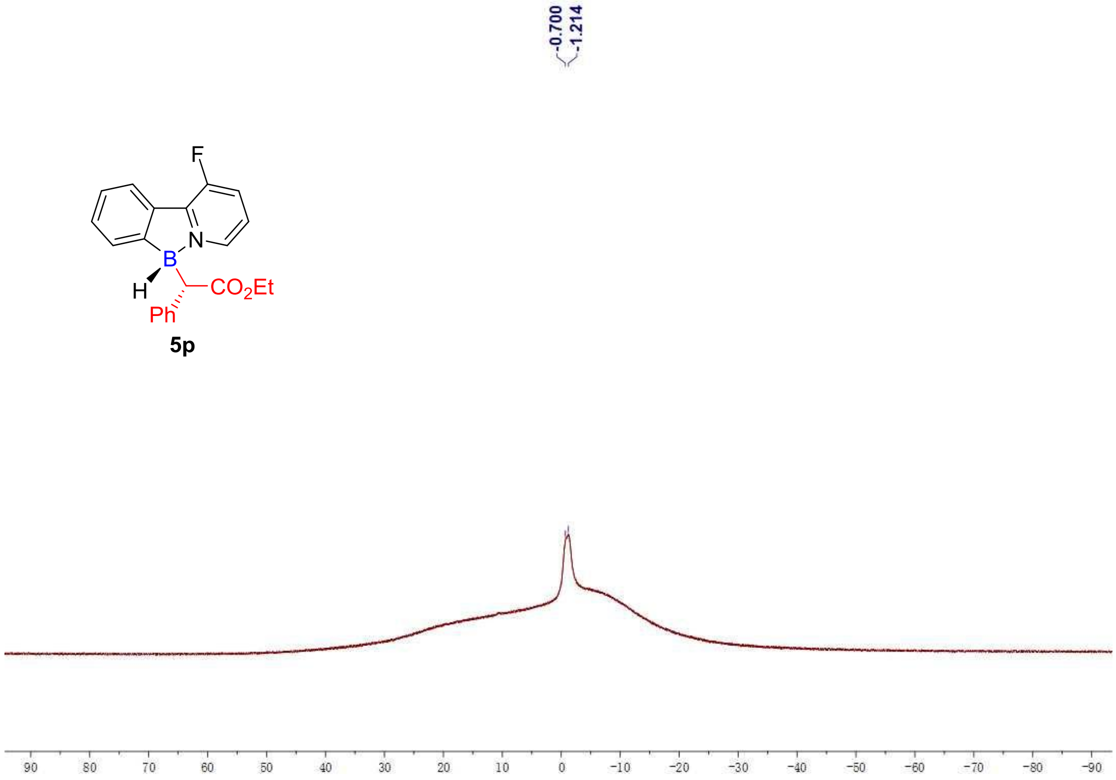

Supplementary Figure 249. ^{11}B NMR spectrum of compound 5p

^{19}F NMR (565 MHz, room temperature, $CDCl_3$)

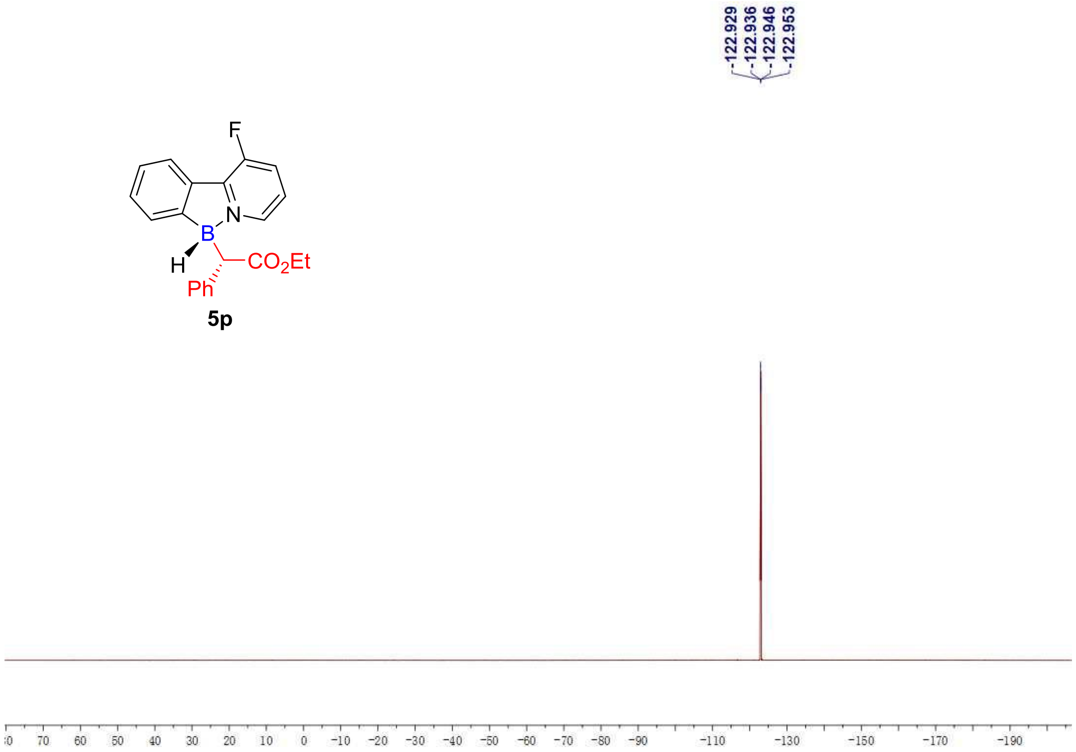

Supplementary Figure 250. ^{19}F NMR spectrum of compound 5p

methyl (*S*)-2-(7-chloro-6H-5 ^{4}-benzo[3,4][1,2]azaborolo[1,5-a]pyridin-6-yl)-2-phenylacetate (5q)

^{1}H NMR (500 MHz, room temperature, $CDCl_3$)

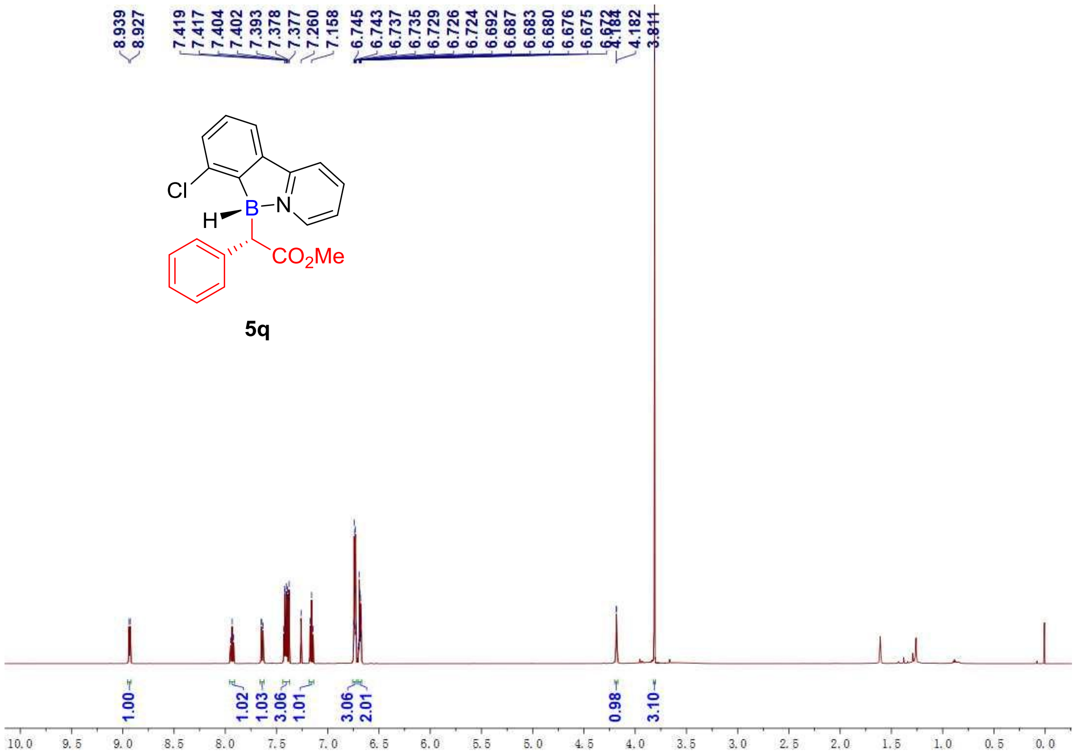

Supplementary Figure 251. ^{1}H NMR spectrum of compound 5q

^{13}C NMR (101 MHz, room temperature, $CDCl_3$)

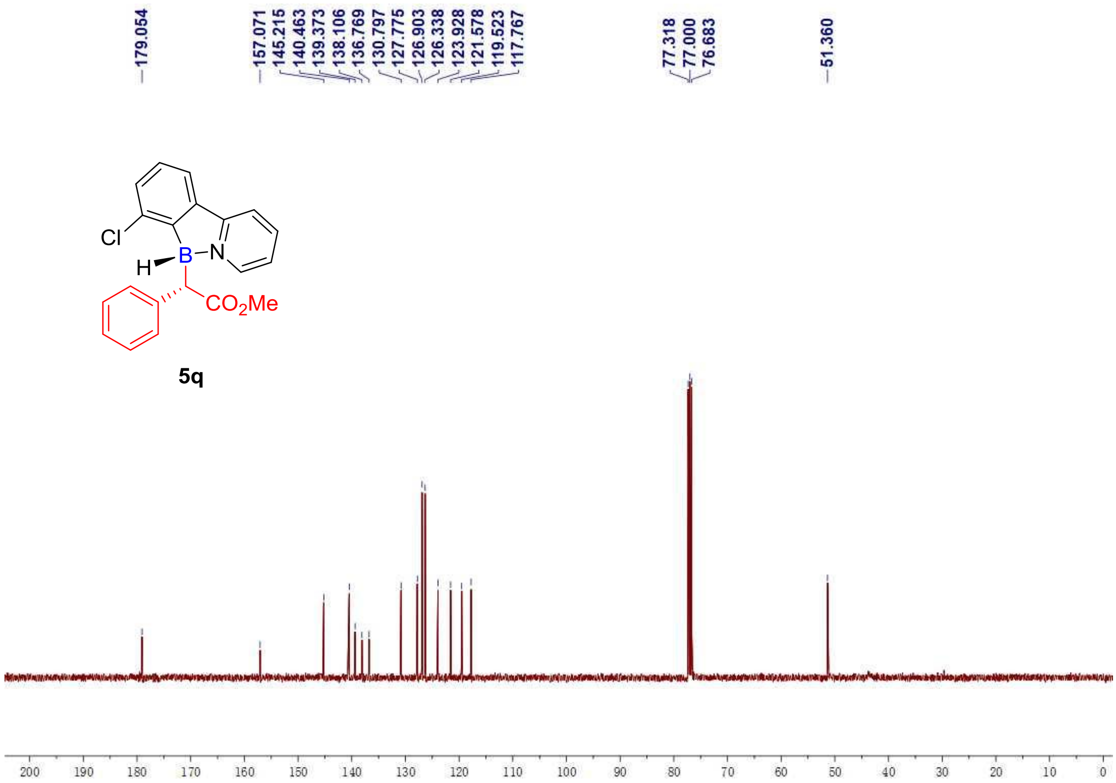

Supplementary Figure 252. ^{13}C **NMR spectrum of compound 5q**

^{11}B NMR (128 MHz, room temperature, $CDCl_3$)

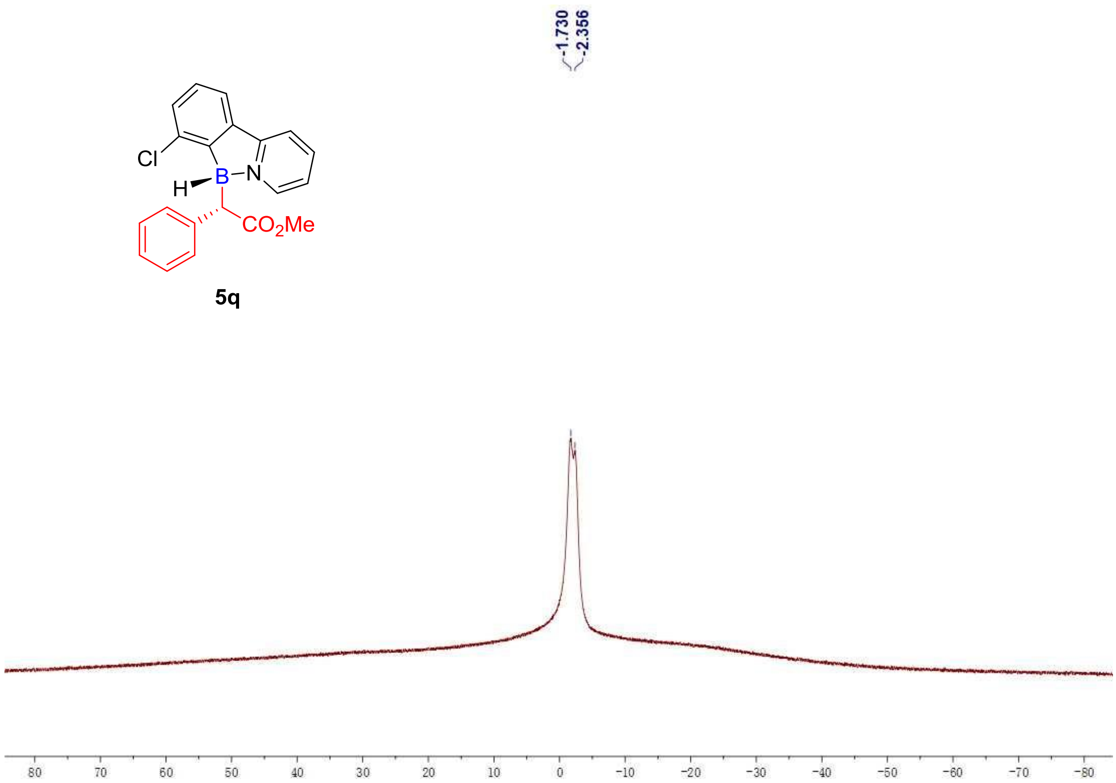

Supplementary Figure 253. ^{11}B **NMR spectrum of compound 5q**

benzyl (S)-2-(7-chloro-6H-5 ^{4}-benzo[3,4][1,2]azaborolo[1,5-a]pyridin-6-yl)-2-phenylacetate (5r)

^{1}H NMR (400 MHz, room temperature, $CDCl_3$)

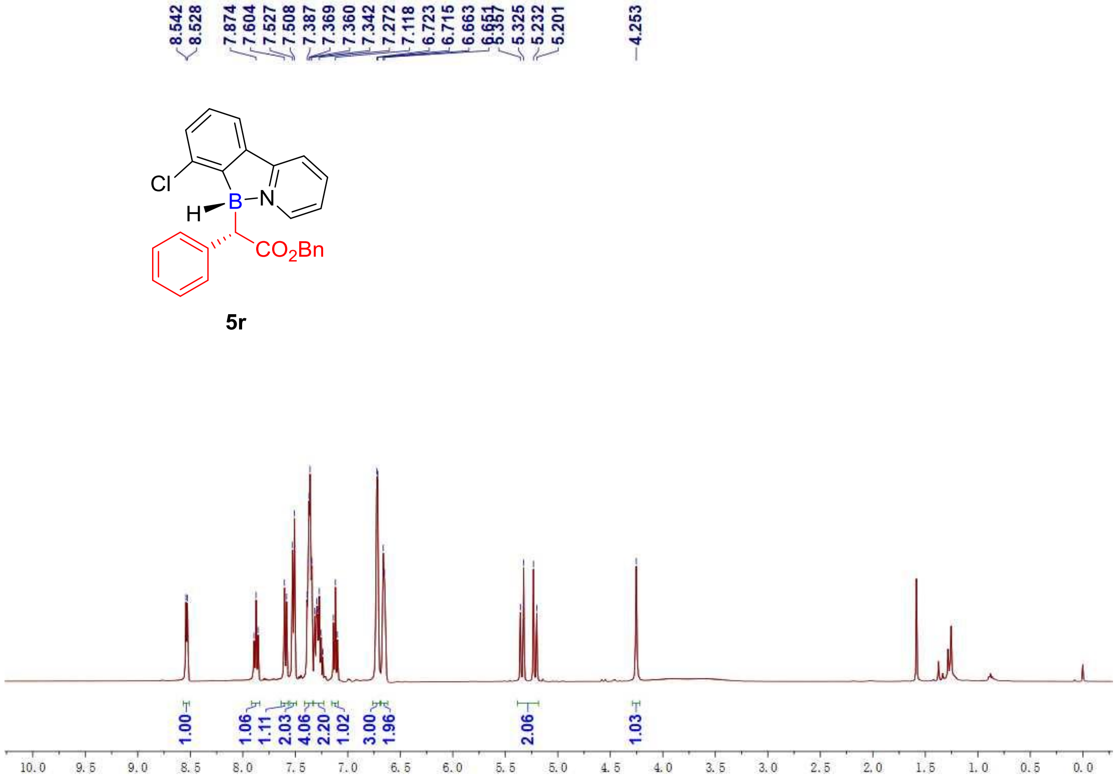

Supplementary Figure 254. ^{1}H NMR spectrum of compound 5r

^{13}C NMR (126 MHz, room temperature, $CDCl_3$)

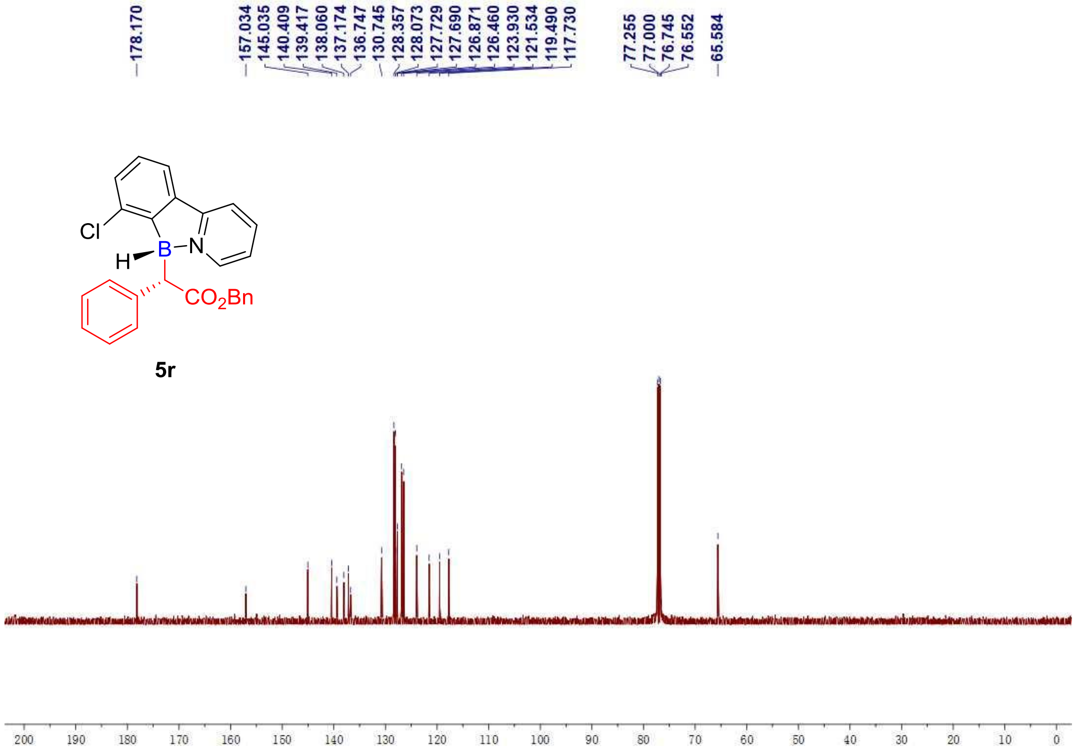

Supplementary Figure 255. ^{13}C NMR spectrum of compound 5r

^{11}B NMR (128 MHz, room temperature, $CDCl_3$)

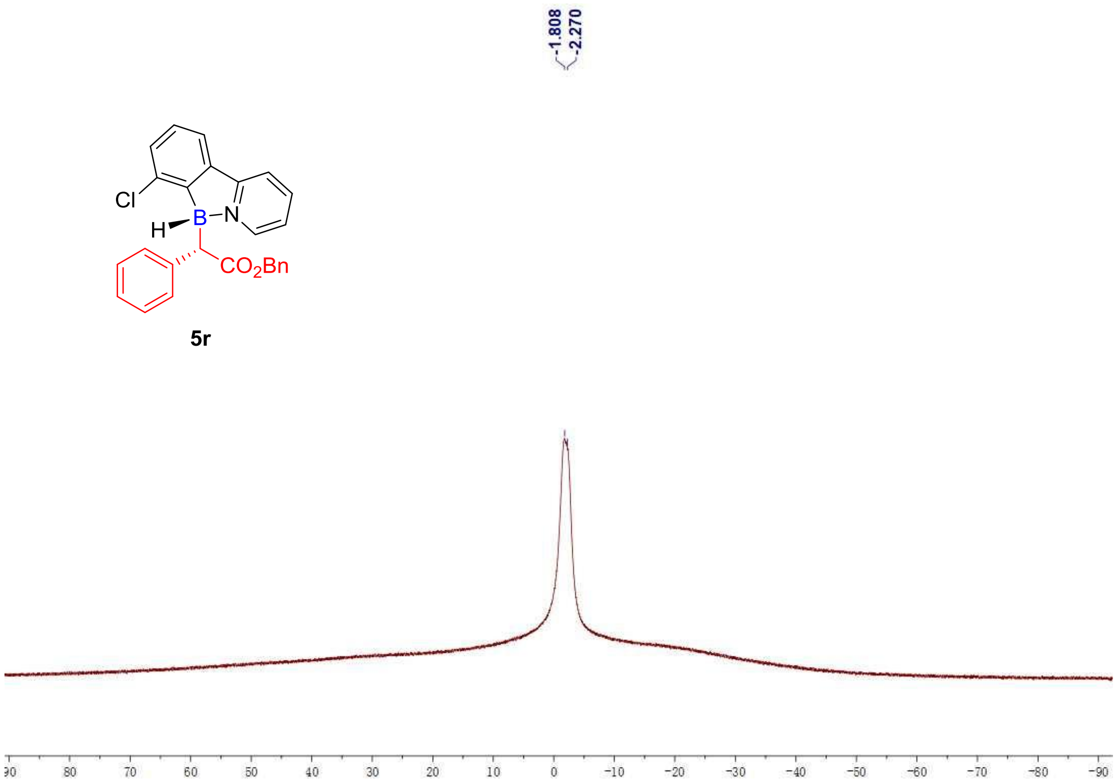

Supplementary Figure 256. ^{11}**B NMR spectrum of compound 5r**

benzyl (S)-2-(7-chloro-6H-5 ^{4}-benzo[3,4][1,2]azaborolo[1,5-a]pyridin-6-yl)-2-phenylacetate(5s)

^{1}H NMR (400 MHz, room temperature, $CDCl_3$)

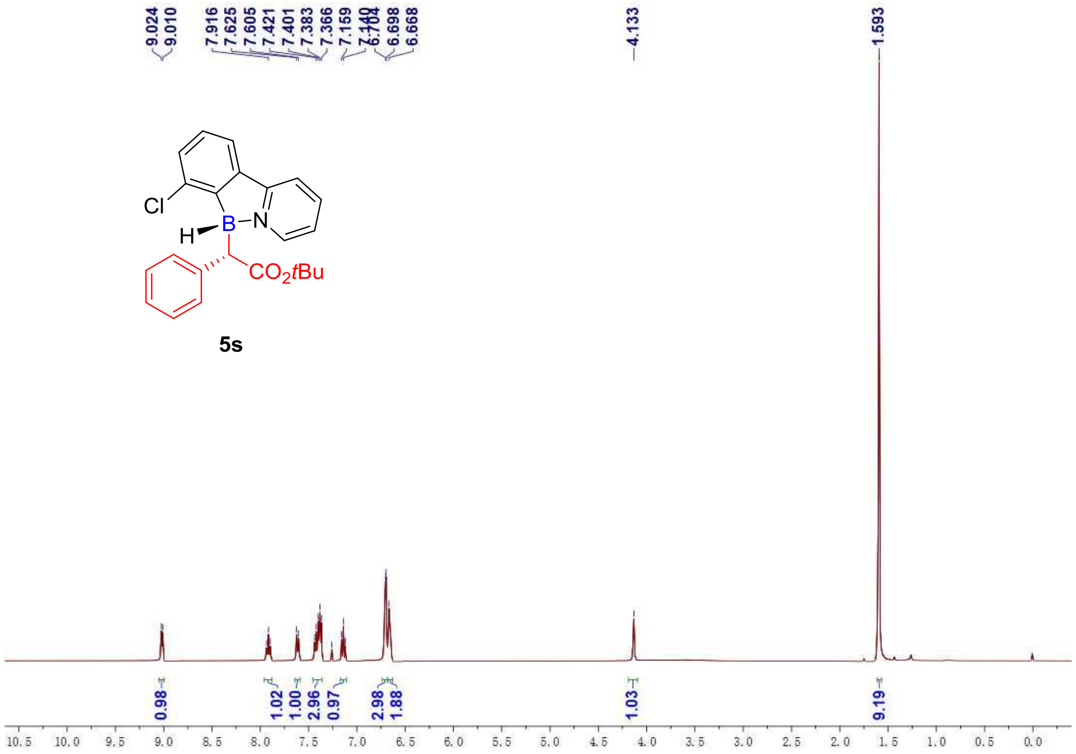

Supplementary Figure 257. ^{1}**H NMR spectrum of compound 5s**

^{13}C NMR (101 MHz, room temperature, $CDCl_3$)

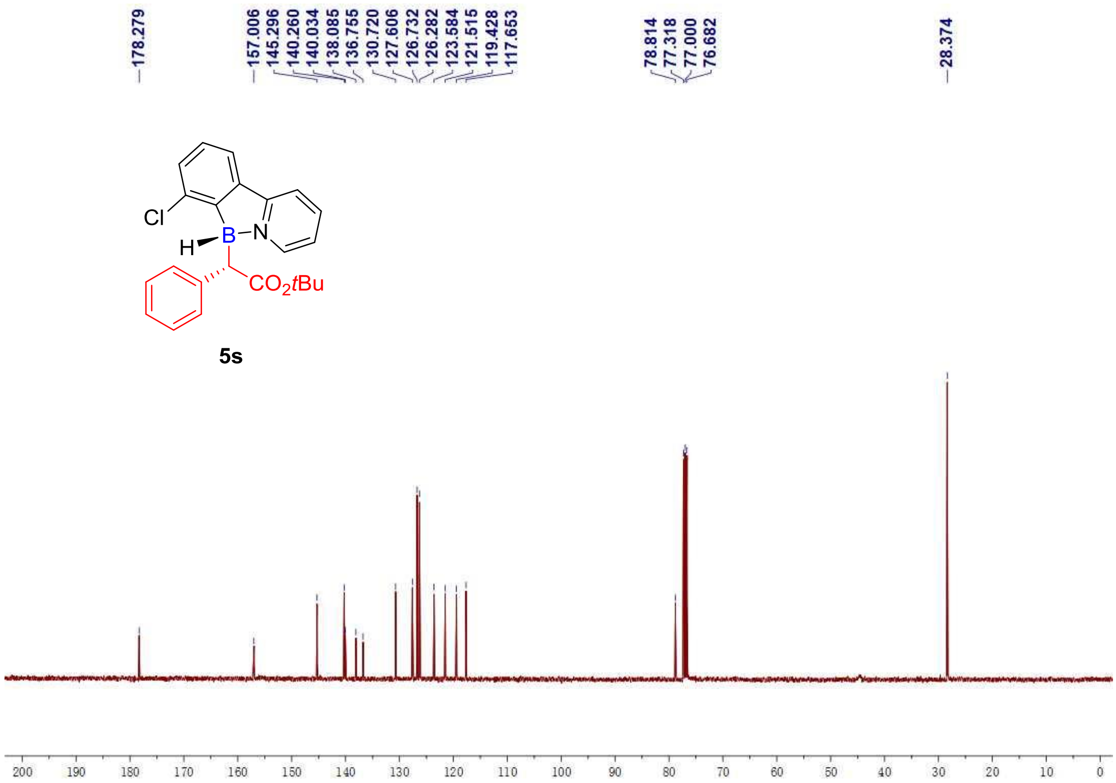

Supplementary Figure 258. ^{13}C NMR spectrum of compound 5s

^{11}B NMR (128 MHz, room temperature, $CDCl_3$)

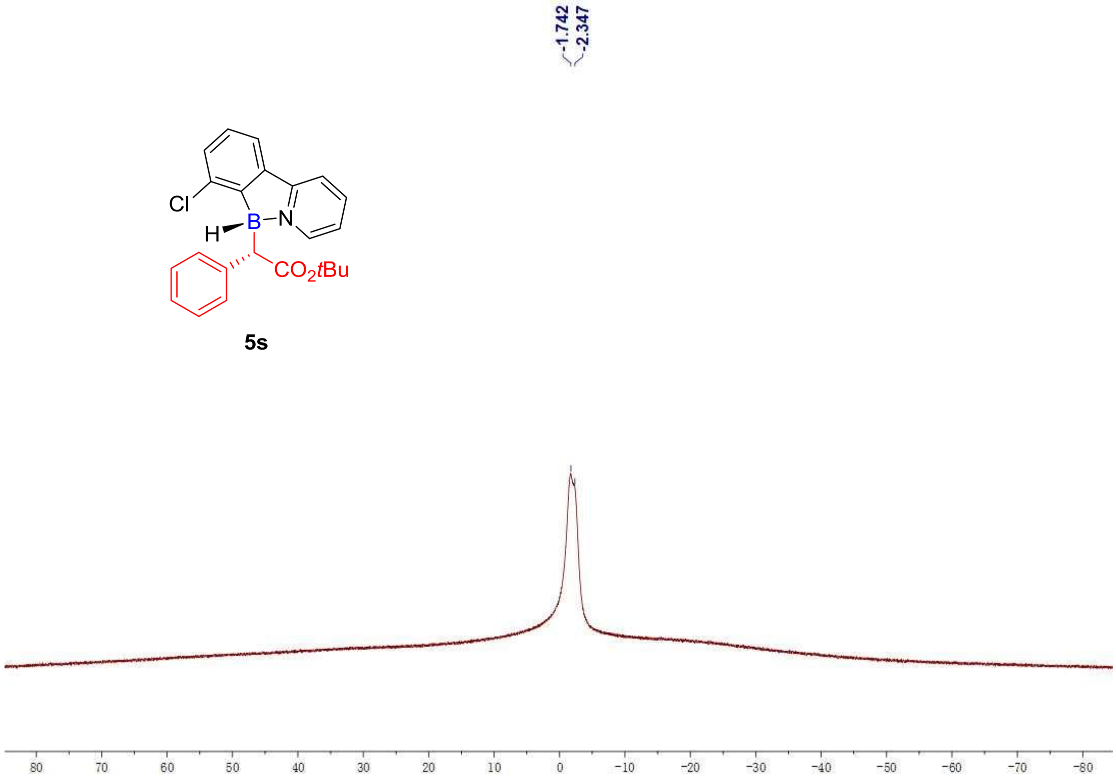

Supplementary Figure 259. ^{11}B NMR spectrum of compound 5s

phenyl (S)-2-(7-chloro-6H-5 ^{4}-benzo[3,4][1,2]azaborolo[1,5-a]pyridin-6-yl)-2-phenylacetate(5t)

^{1}H NMR (400 MHz, room temperature, $CDCl_3$)

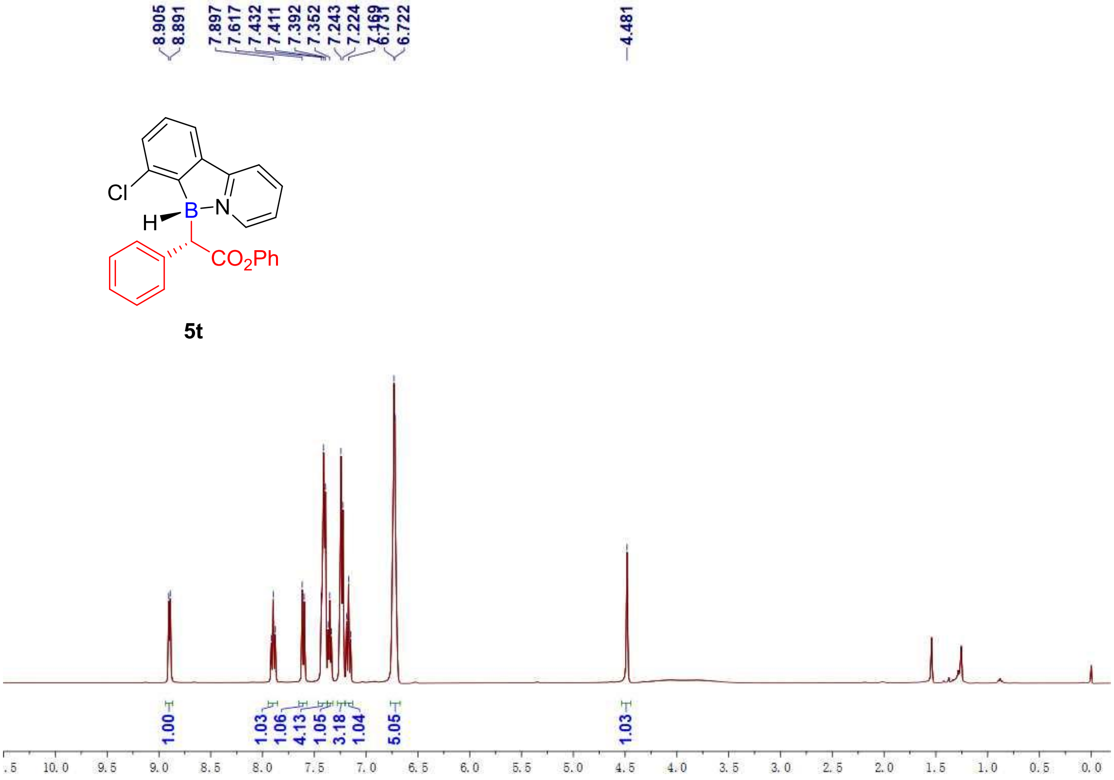

Supplementary Figure 260. ^{1}H NMR spectrum of compound 5t

^{13}C NMR (126 MHz, room temperature, $CDCl_3$)

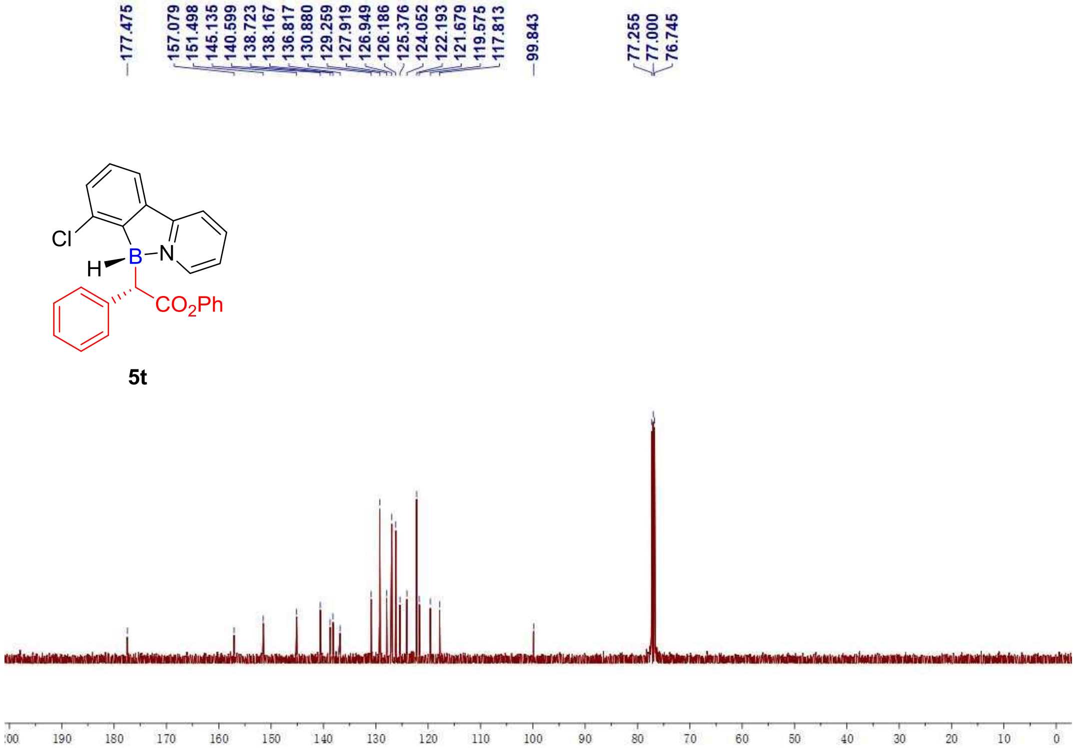

Supplementary Figure 261. ^{13}C NMR spectrum of compound 5t

^{11}B NMR (128 MHz, room temperature, $CDCl_3$)

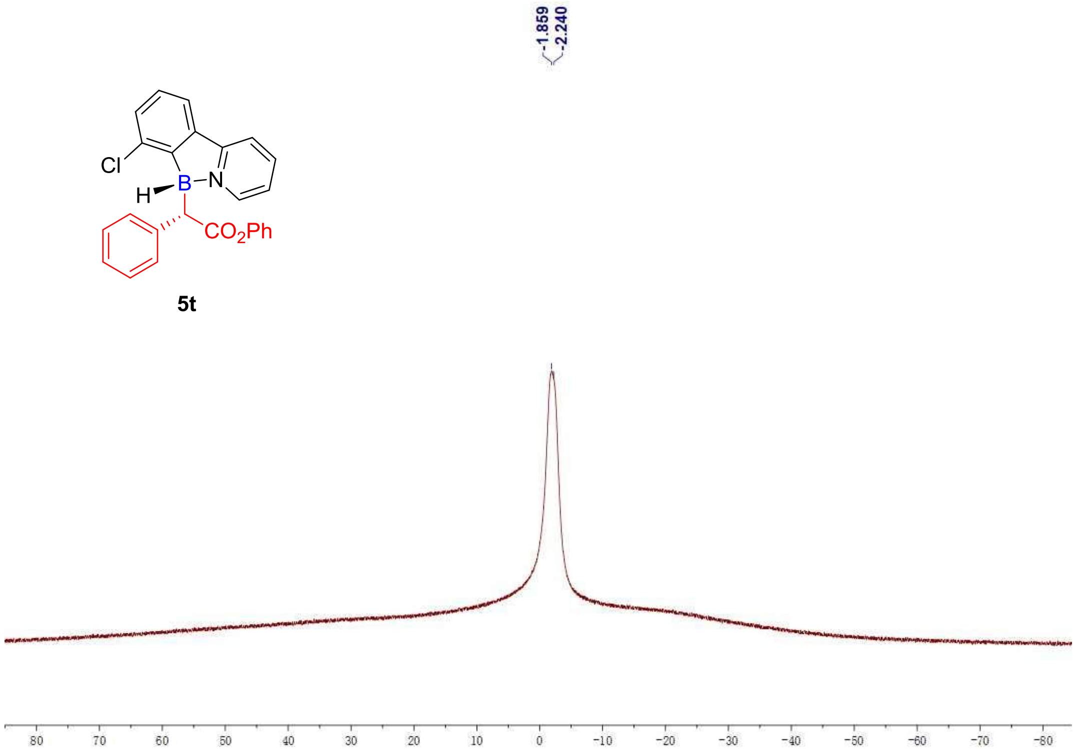

Supplementary Figure 262. ^{11}**B NMR spectrum of compound 5t**

ethyl(*S*)-2-(4-(tert-butyl)phenyl)-2-(7-chloro-6H-5 ^{4}-benzo[3,4][1,2]azaborolo[1,5-a]pyridin-6-yl)acetate (5u)

^{1}H NMR (500 MHz, room temperature, $CDCl_3$)

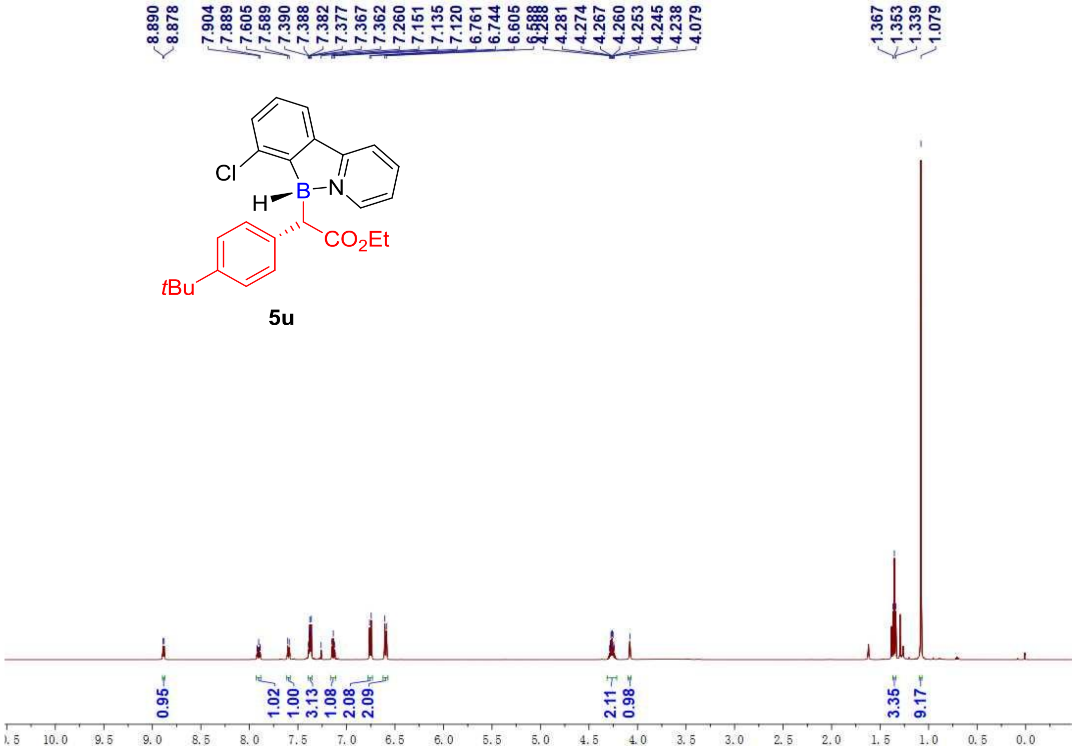

Supplementary Figure 263. ^{1}**H NMR spectrum of compound 5u**

^{13}C NMR (126 MHz, room temperature, $CDCl_3$)

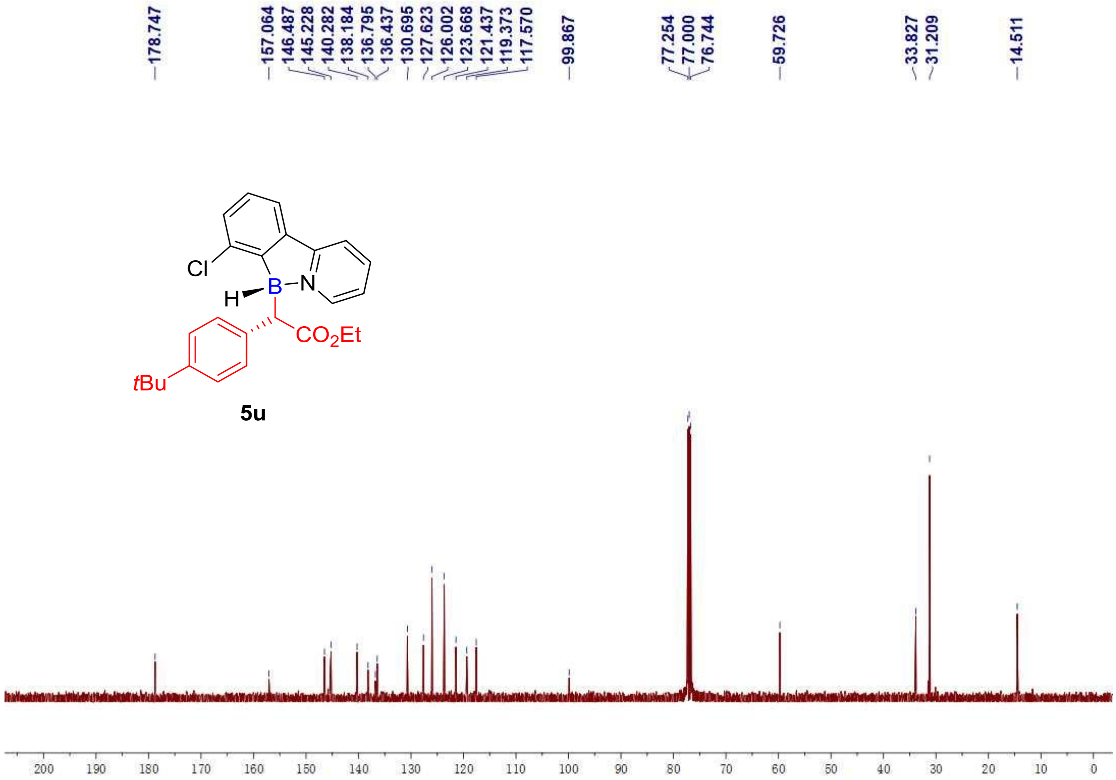

Supplementary Figure 264. ^{13}C **NMR spectrum of compound 5u**

^{11}B NMR (128 MHz, room temperature, $CDCl_3$)

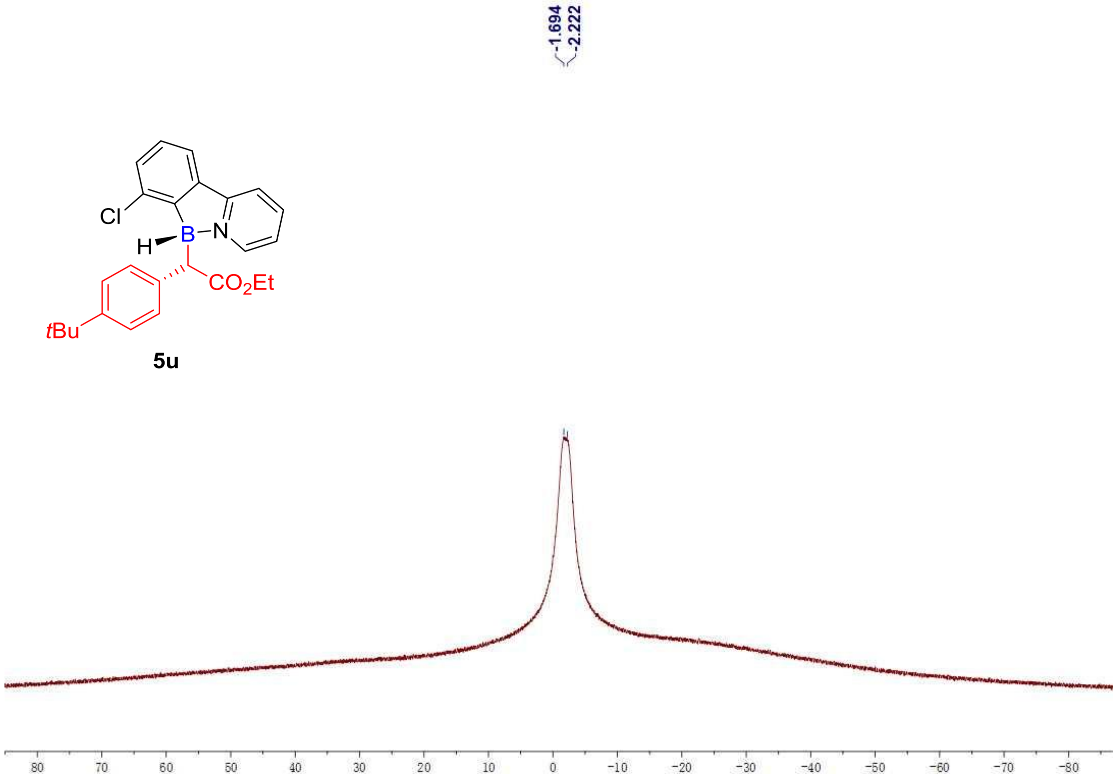

Supplementary Figure 265. ^{11}B **NMR spectrum of compound 5u**

ethyl (S)-2-(7-chloro-6H-5 ^{4}-benzo[3,4][1,2]azaborolo[1,5-a]pyridin-6-yl)-2-(p-tolyl)acetate (5v)

^{1}H NMR (500 MHz, room temperature, $CDCl_3$)

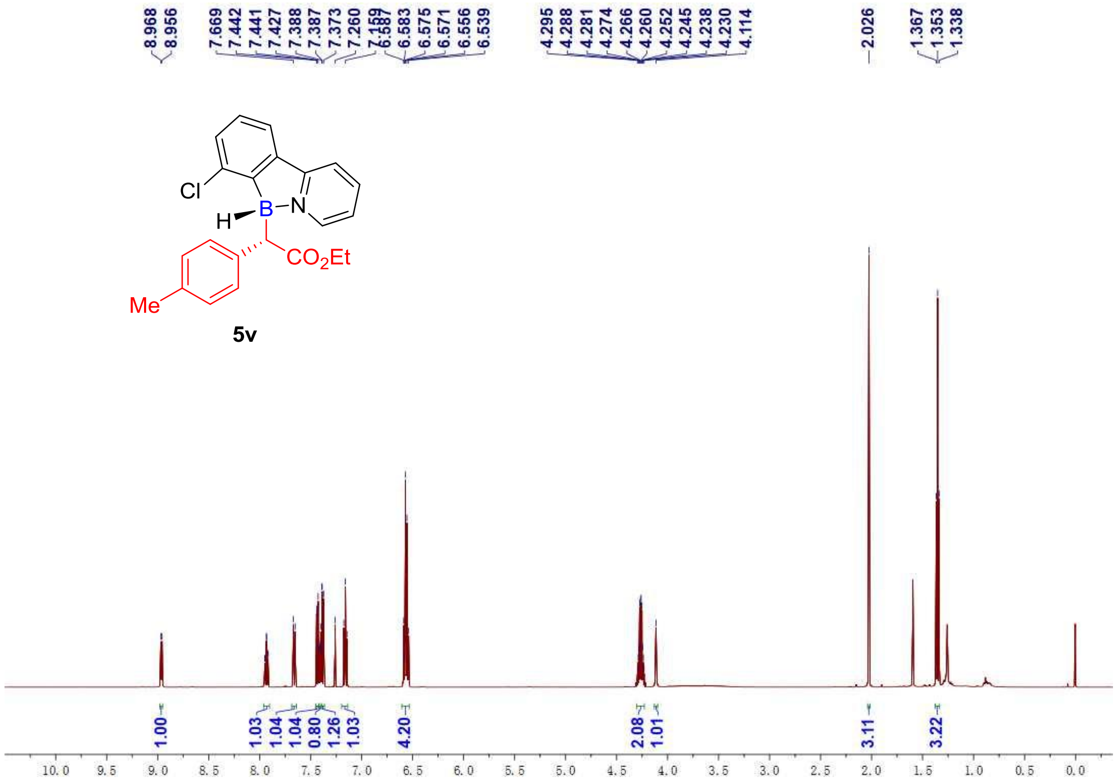

Supplementary Figure 266. ^{1}H NMR spectrum of compound 5v

^{13}C NMR (126 MHz, room temperature, $CDCl_3$)

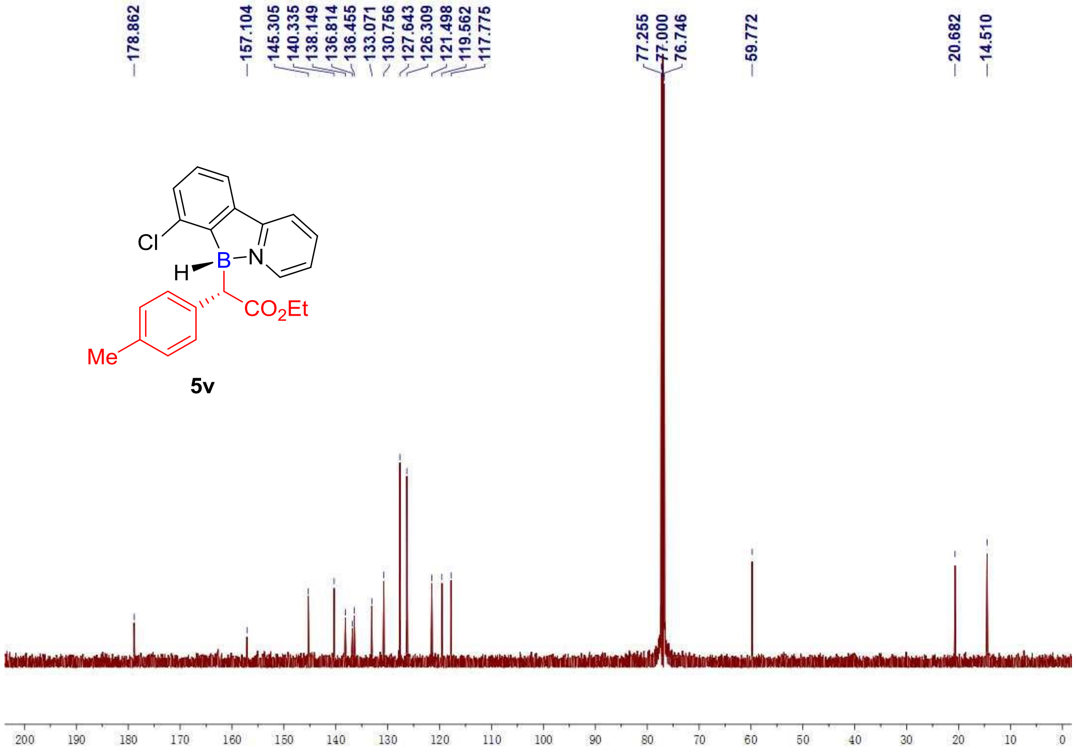

Supplementary Figure 267. ^{13}C NMR spectrum of compound 5v

^{11}B NMR (128 MHz, room temperature, $CDCl_3$)

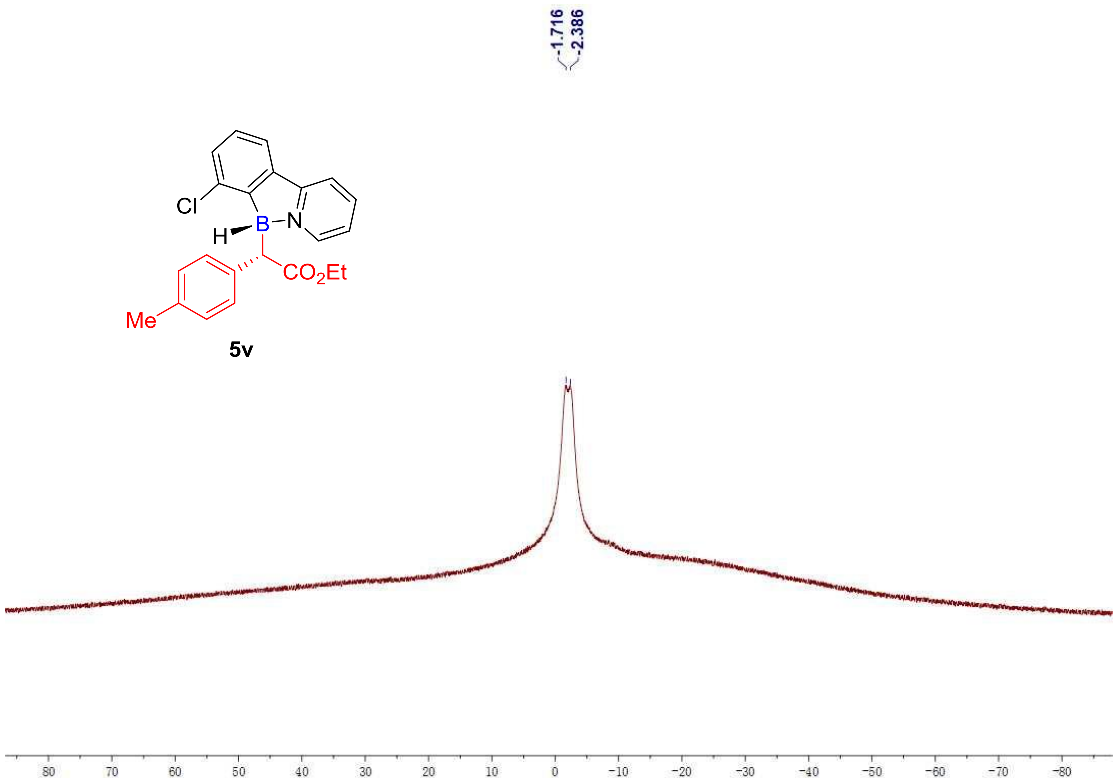

Supplementary Figure 268. ^{11}B NMR spectrum of compound 5v

ethyl (*S*)-2-(7-chloro-6H-5 ^{4}-benzo[3,4][1,2]azaborolo[1,5-a]pyridin-6-yl)-2-(m-tolyl)acetate (5w)

^{1}H NMR (500 MHz, room temperature, $CDCl_3$)

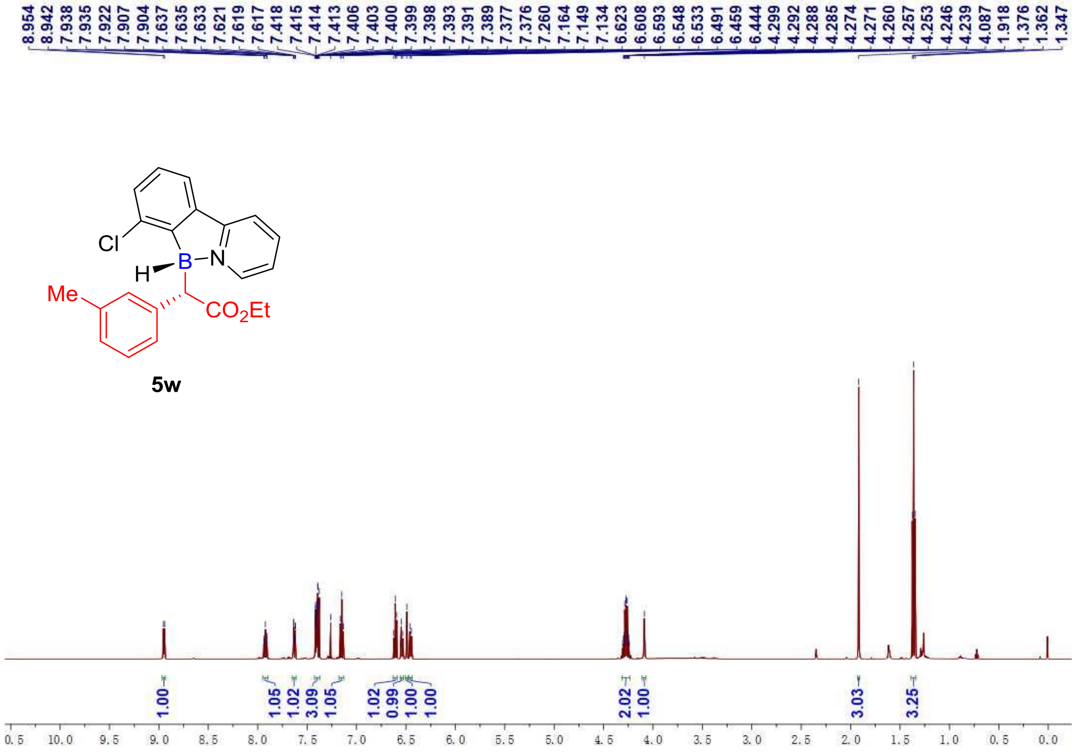

Supplementary Figure 269. ^{1}H NMR spectrum of compound 5w

^{13}C NMR (126 MHz, room temperature, $CDCl_3$)

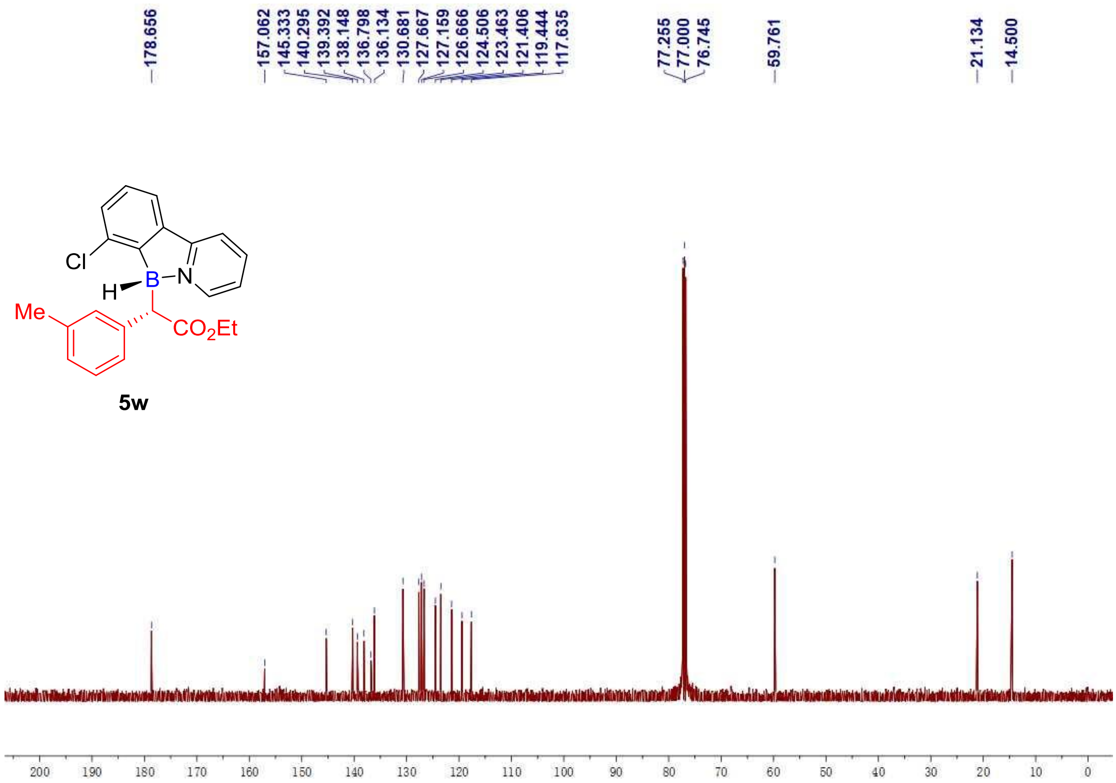

Supplementary Figure 270. ^{13}C NMR spectrum of compound 5w

^{11}B NMR (128 MHz, room temperature, $CDCl_3$)

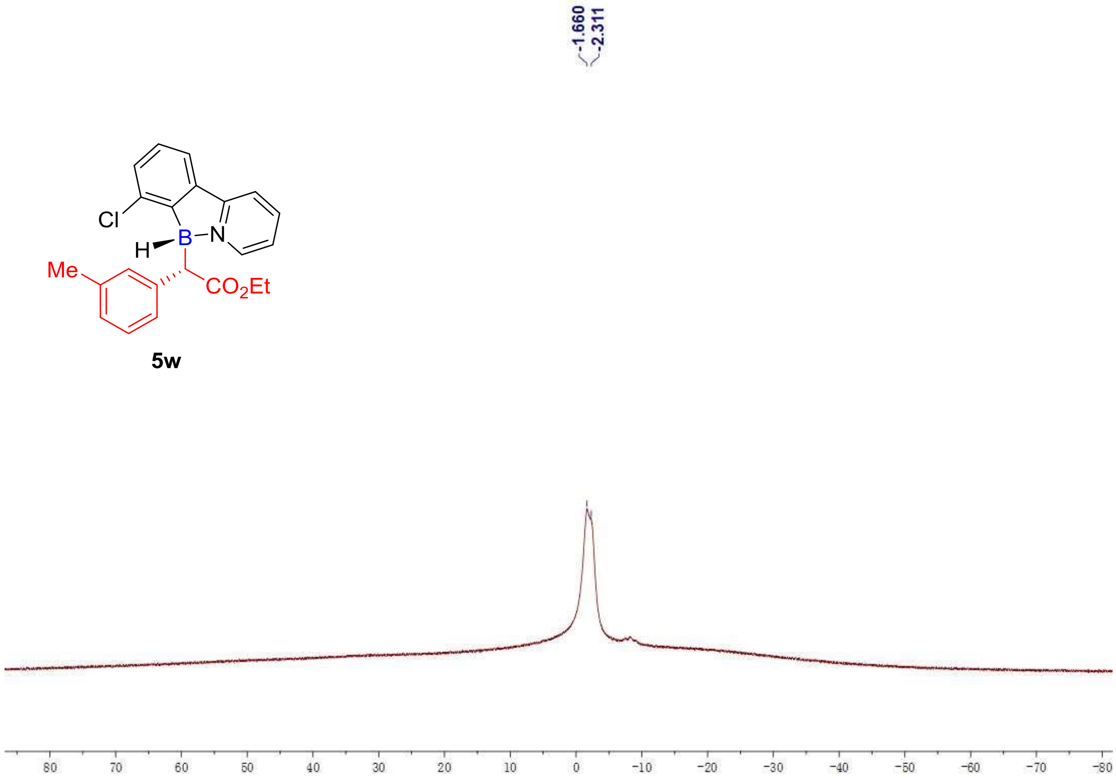

Supplementary Figure 271. ^{11}B NMR spectrum of compound 5w

ethyl(*S*)-2-(7-chloro-6H-5 ^{4}-benzo[3,4][1,2]azaborolo[1,5-a]pyridin-6-yl)-2-(3-methoxyphenyl)acetate (5x)

^{1}H NMR (500 MHz, room temperature, $CDCl_3$)

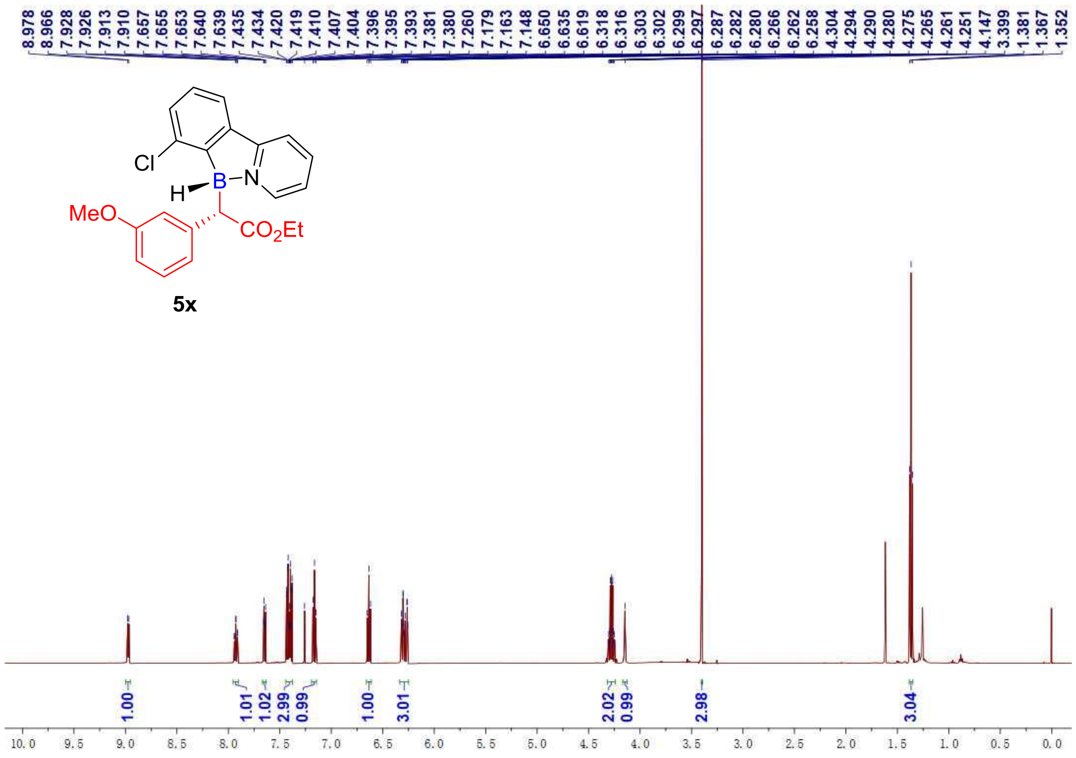

Supplementary Figure 272. ^{1}H NMR spectrum of compound 5x

^{13}C NMR (126 MHz, room temperature, $CDCl_3$)

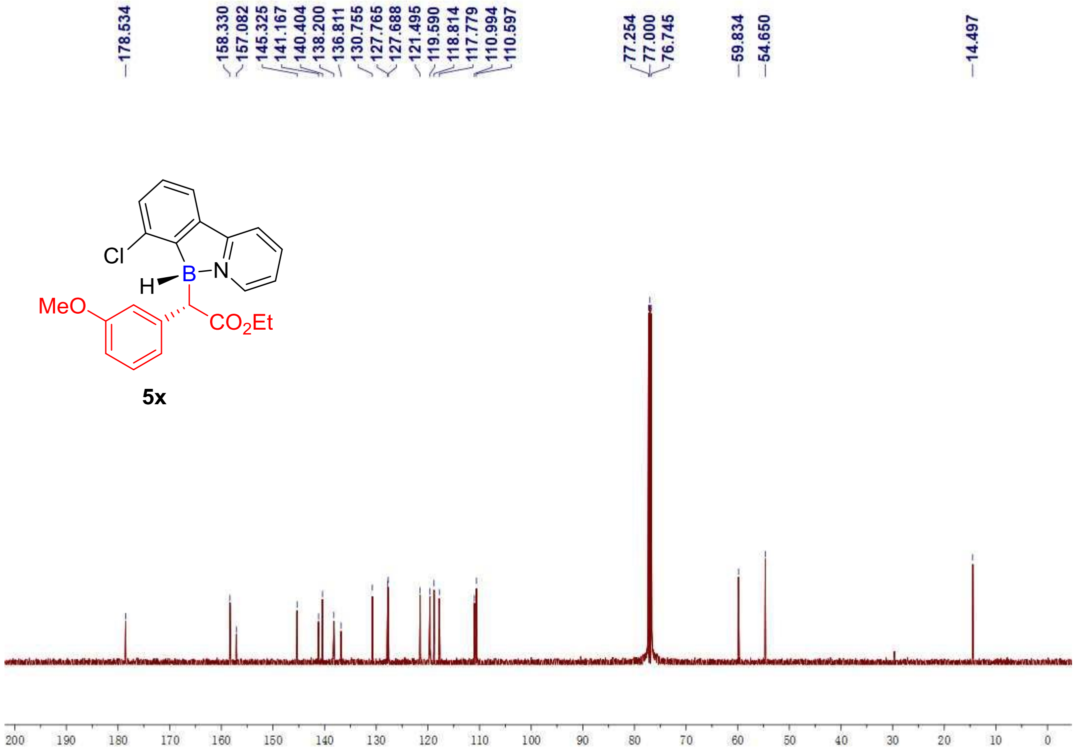

Supplementary Figure 273. ^{13}C NMR spectrum of compound 5x

^{11}B NMR (128 MHz, room temperature, $CDCl_3$)

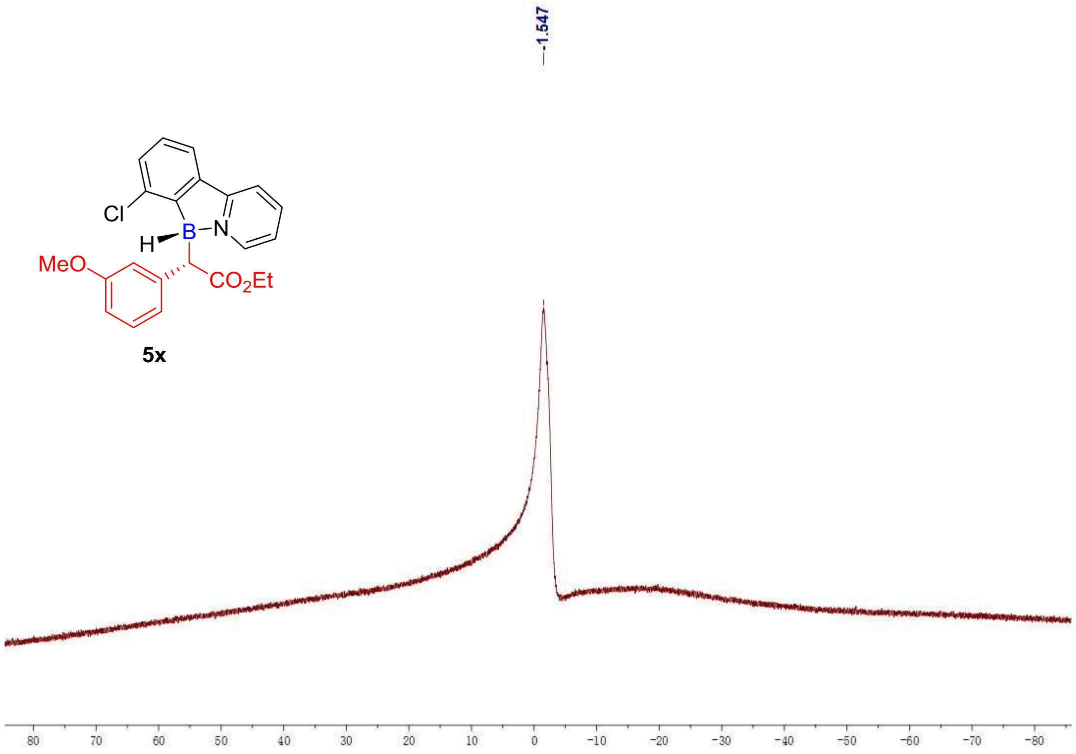

Supplementary Figure 274. ^{11}B NMR spectrum of compound 5x

ethyl(*S*)-2-(benzo[d][1,3]dioxol-5-yl)-2-(7-chloro-6H-5 ^{4}-benzo[3,4][1,2]azaborolo[1,5-a]pyridin-6-yl)acetate (5y)

^{1}H NMR (400 MHz, room temperature, $CDCl_3$)

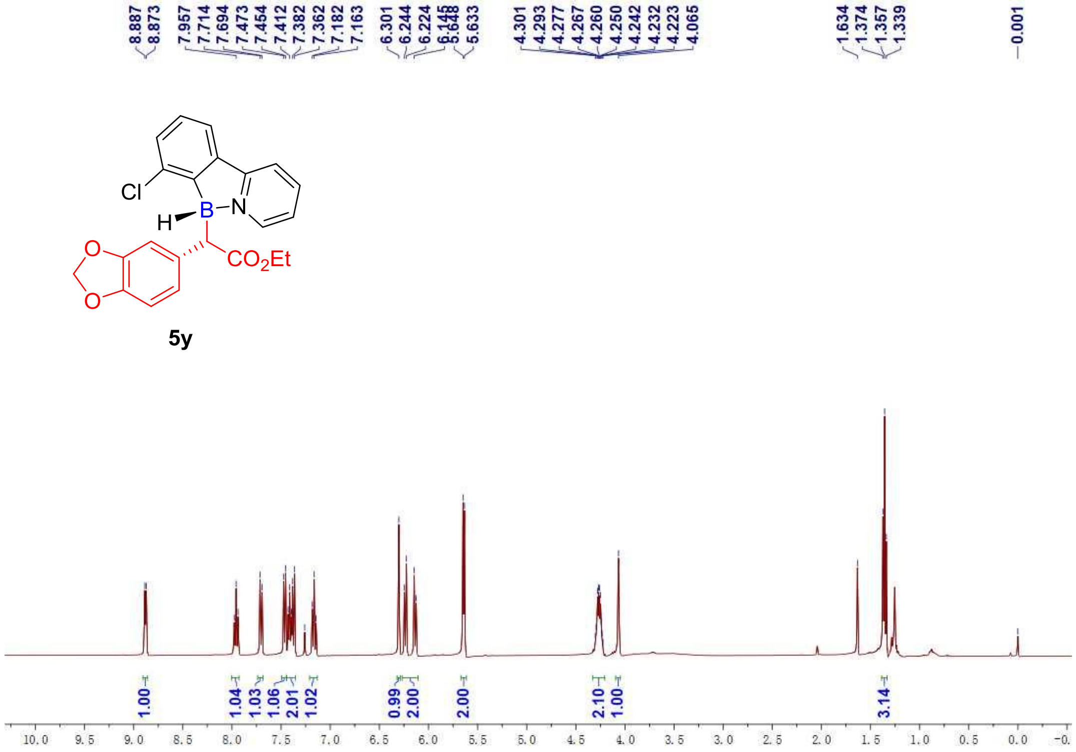

Supplementary Figure 275. ^{1}H NMR spectrum of compound 5y

^{13}C NMR (126 MHz, room temperature, $CDCl_3$)

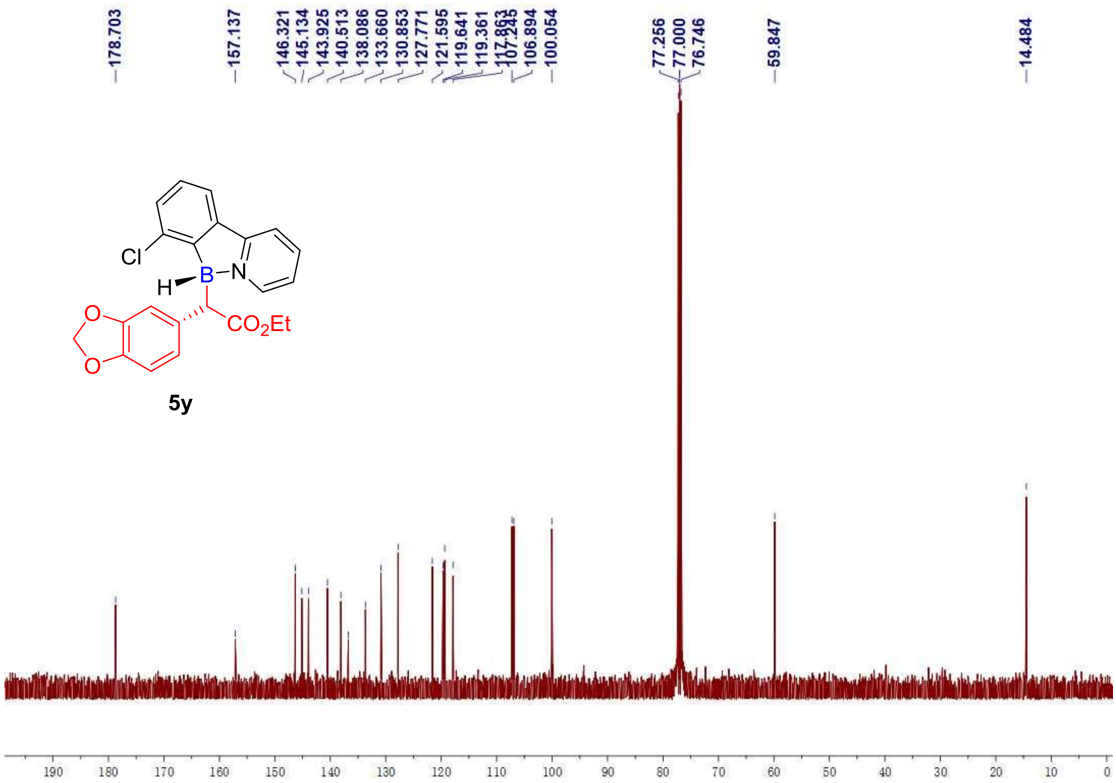

Supplementary Figure 276. ^{13}C NMR spectrum of compound 5y

^{11}B NMR (128 MHz, room temperature, $CDCl_3$)

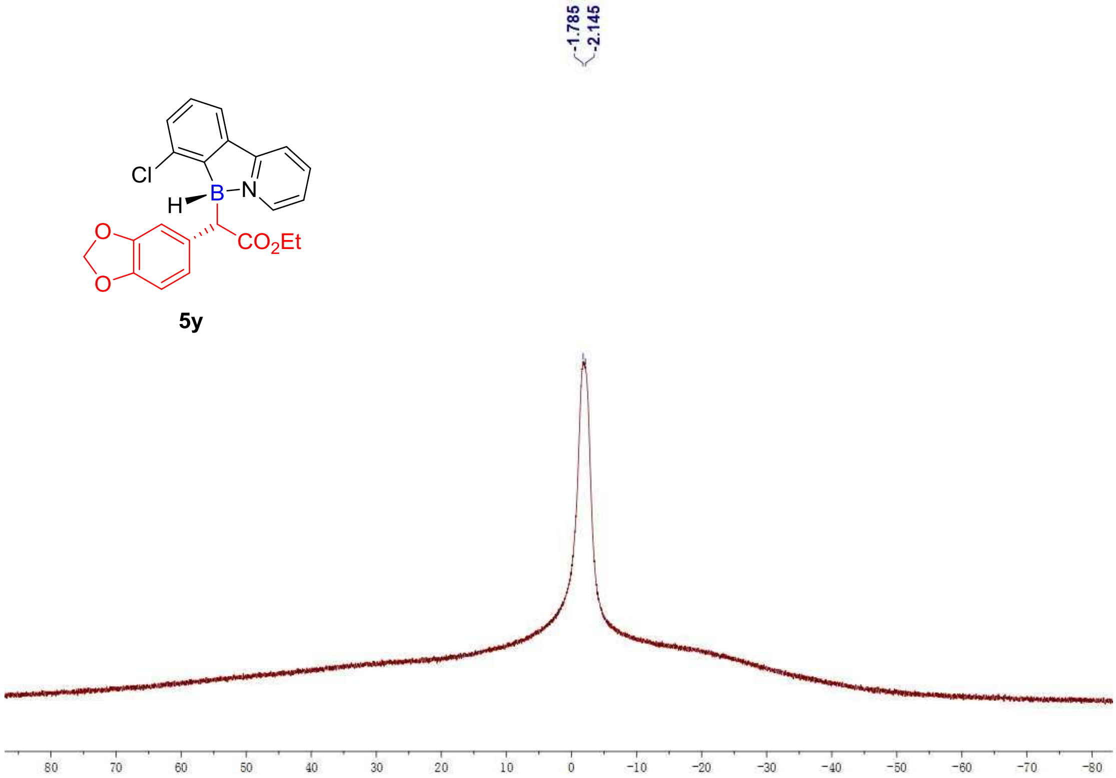

Supplementary Figure 277. ^{11}B NMR spectrum of compound 5y

ethyl(*S*)-2-([1,1'-biphenyl]-4-yl)-2-(7-chloro-6H-5 ^{4}-benzo[3,4][1,2]azaborolo[1,5-a]pyridin-6-yl) acetate (5z)

^{1}H NMR (400 MHz, room temperature, $CDCl_3$)

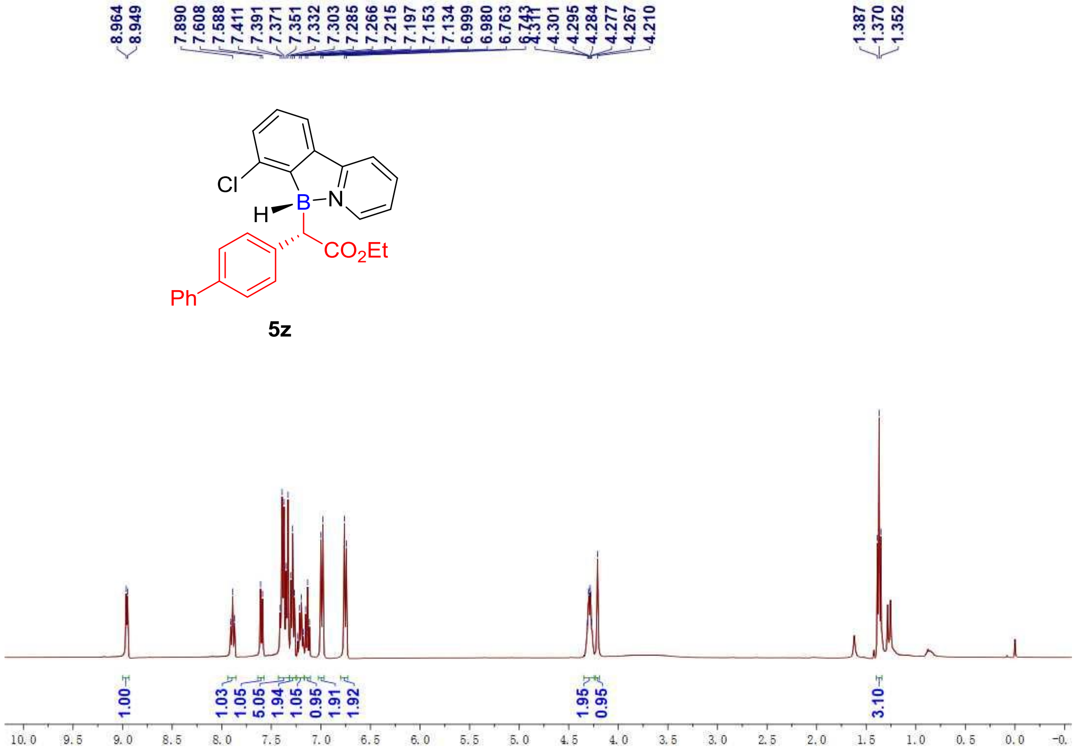

Supplementary Figure 278. ^{1}H NMR spectrum of compound 5z

^{13}C NMR (101 MHz, room temperature, $CDCl_3$)

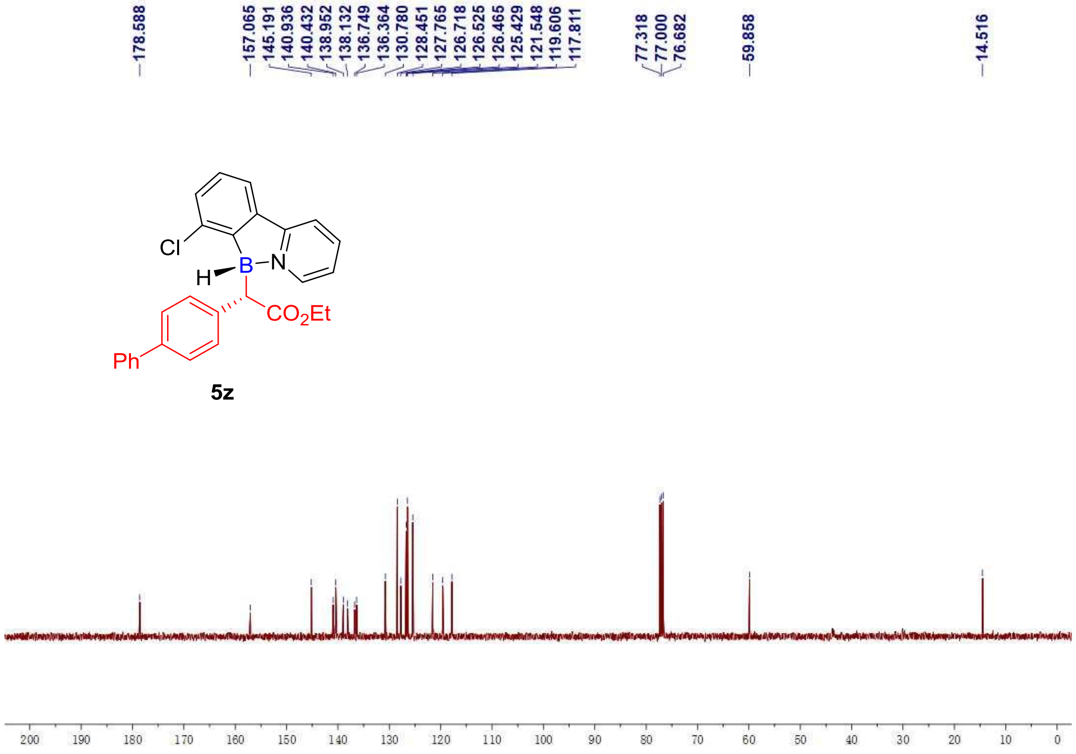

Supplementary Figure 279. ^{13}C NMR spectrum of compound 5z

^{11}B NMR (128 MHz, room temperature, $CDCl_3$)

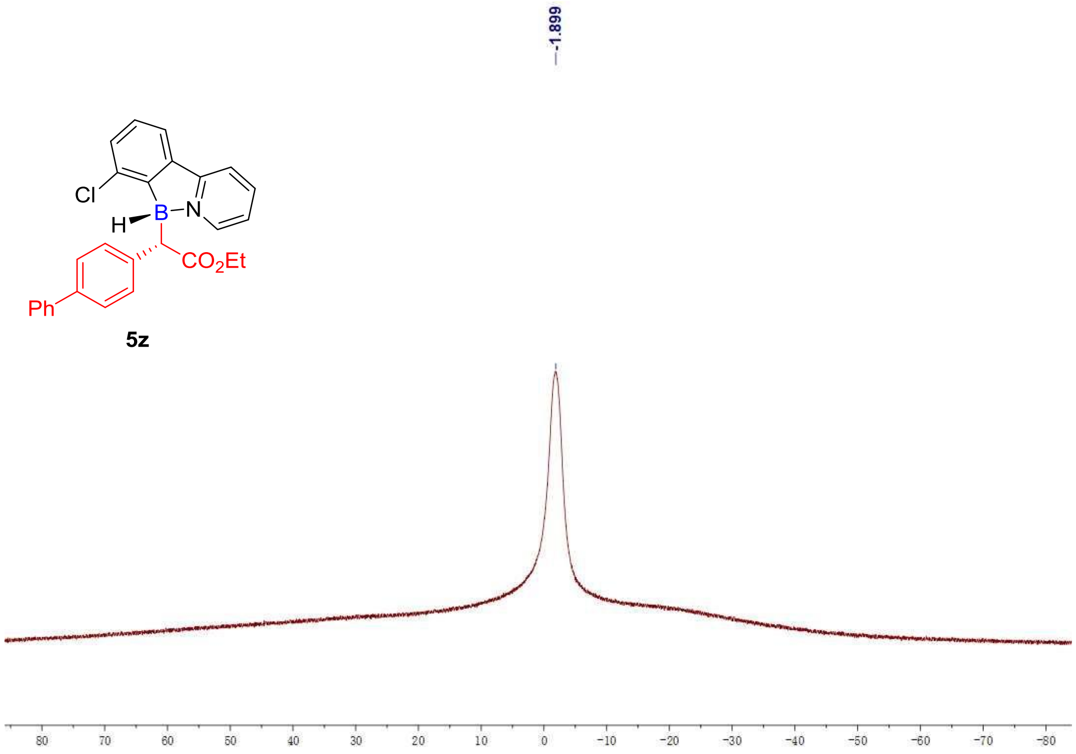

Supplementary Figure 280. ^{11}B NMR spectrum of compound 5z

methyl(*S*)-3-(1-(7-chloro-(*R*)6H-5 ^{4}-benzo[3,4][1,2]azaborolo[1,5-a]pyridin-6-yl)-2-ethoxy-2-oxoethyl)benz-oate (5aa)

^{1}H NMR (500 MHz, room temperature, $CDCl_3$)

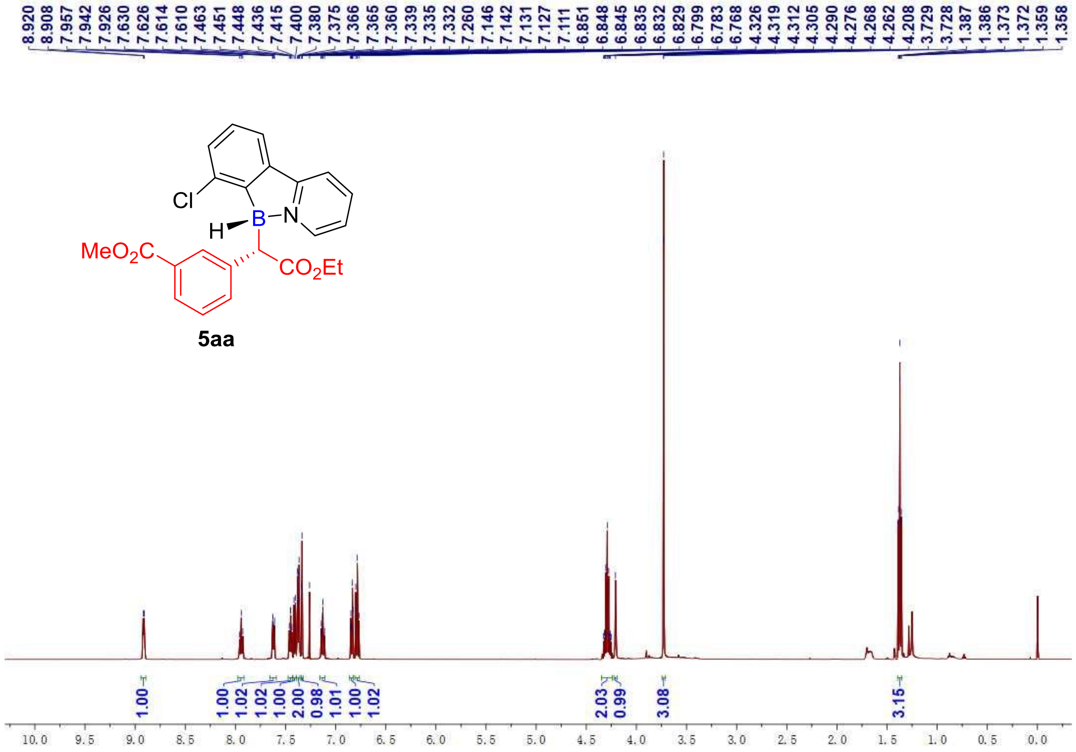

Supplementary Figure 281. ^{1}H NMR spectrum of compound 5aa

^{13}C NMR (101 MHz, room temperature, $CDCl_3$)

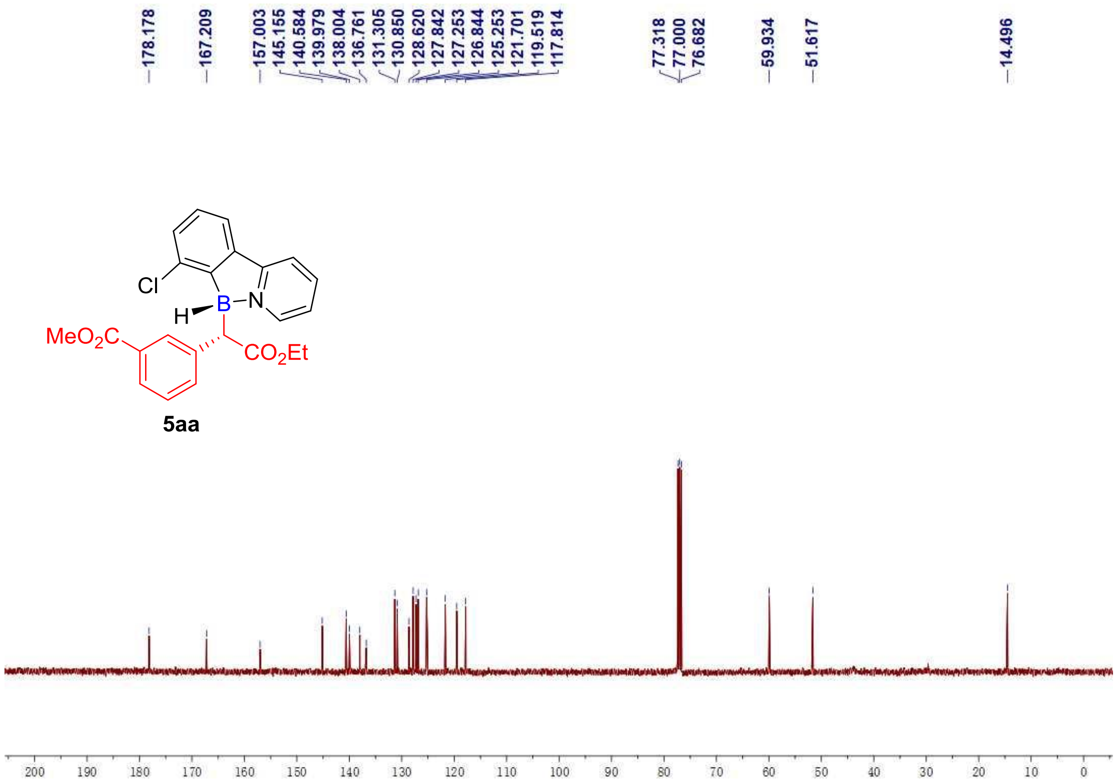

Supplementary Figure 282. ^{13}C NMR spectrum of compound 5aa

^{11}B NMR (128 MHz, room temperature, $CDCl_3$)

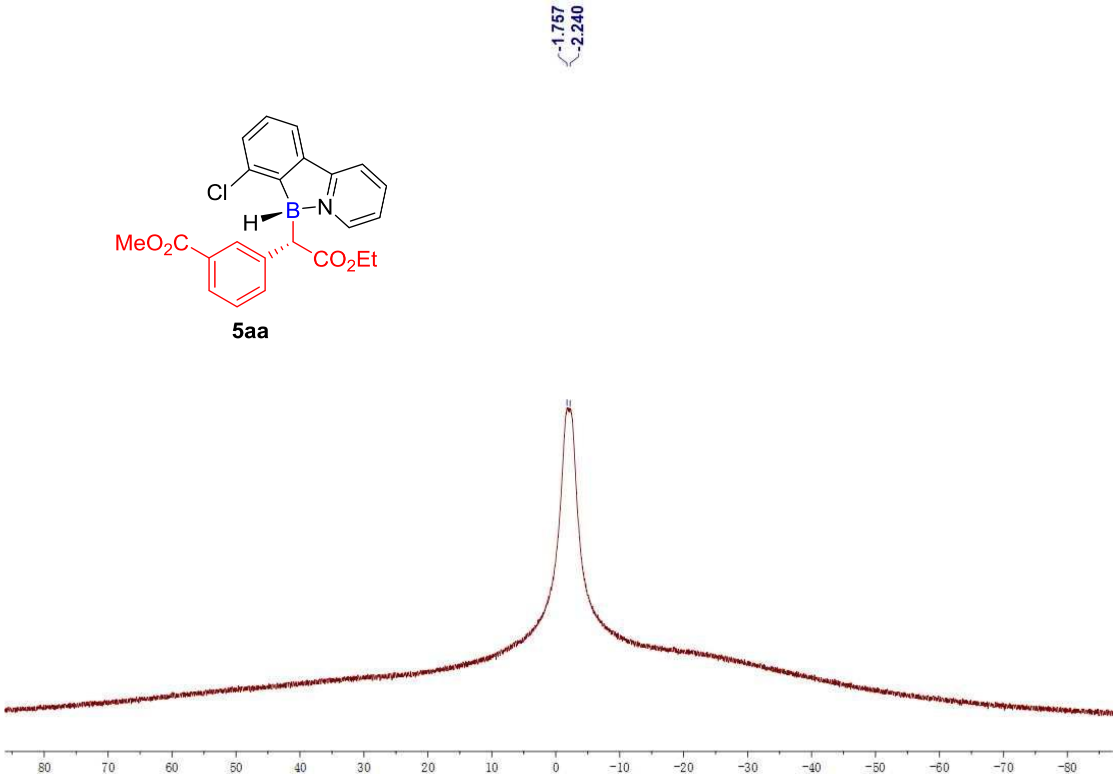

Supplementary Figure 283. ^{11}B NMR spectrum of compound 5aa

ethyl(S)-2-(7-chloro-6H-5 ^{4}-benzo[3,4][1,2]azaborolo[1,5-a]pyridin-6-yl)-2-(4-fluorophenyl)acetate (5ab)

^{1}H NMR (500 MHz, room temperature, $CDCl_3$)

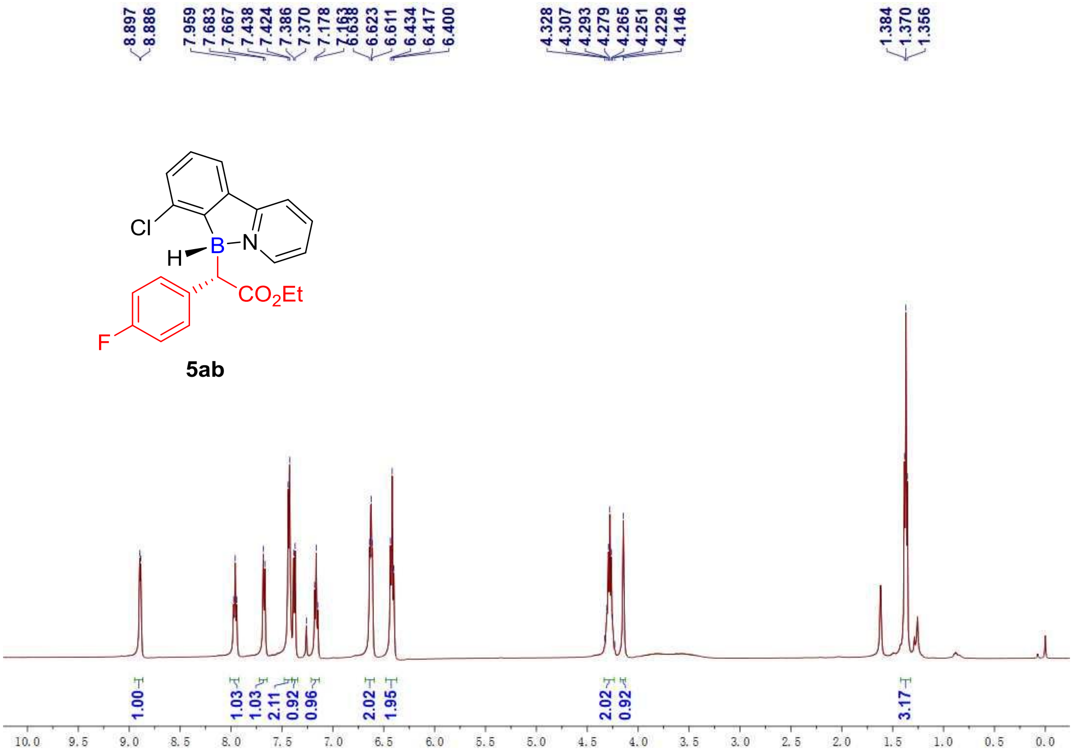

Supplementary Figure 284. ^{1}H NMR spectrum of compound 5ab

^{13}C NMR (101 MHz, room temperature, $CDCl_3$)

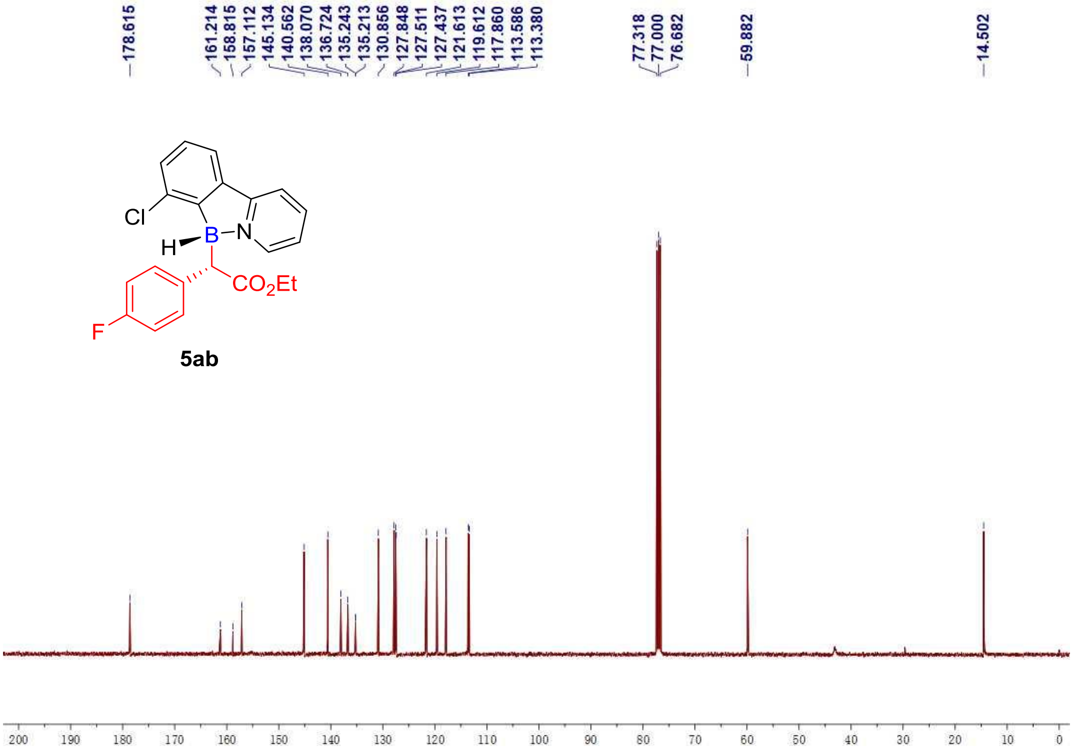

Supplementary Figure 285. ^{13}C NMR spectrum of compound 5ab

^{11}B NMR (128 MHz, room temperature, $CDCl_3$)

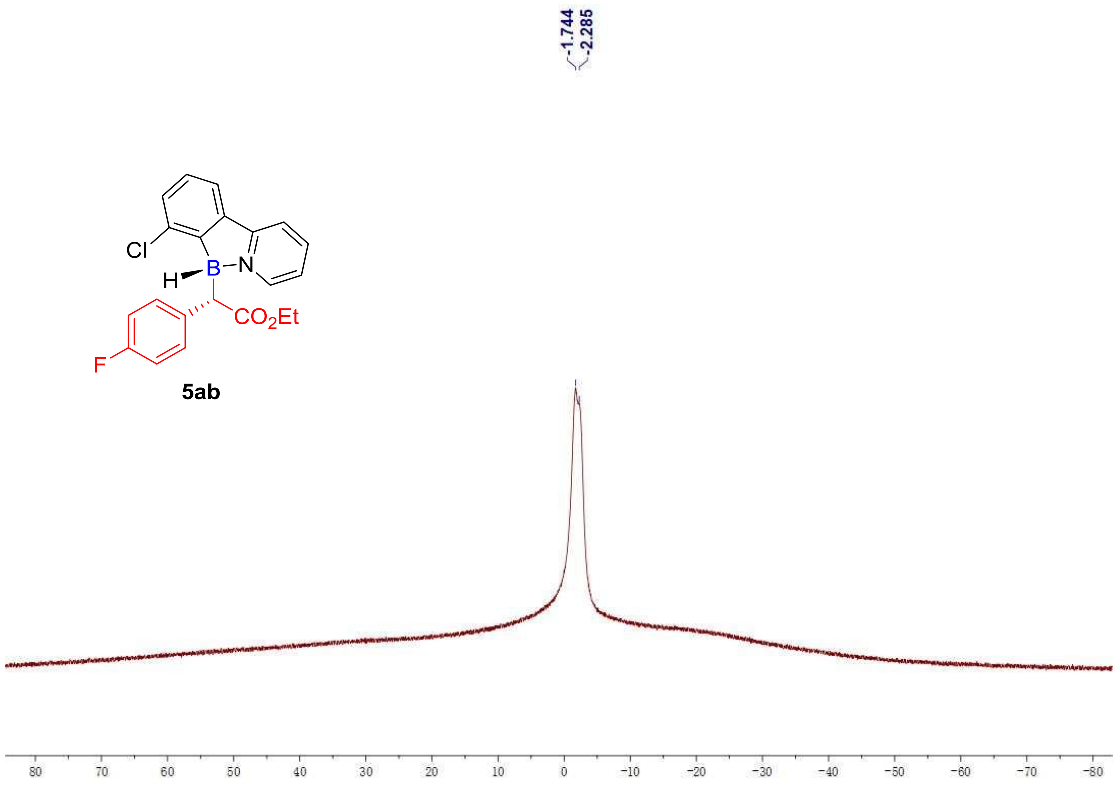

Supplementary Figure 286. ^{11}B NMR spectrum of compound 5ab

^{19}F NMR (376 MHz, room temperature, $CDCl_3$)

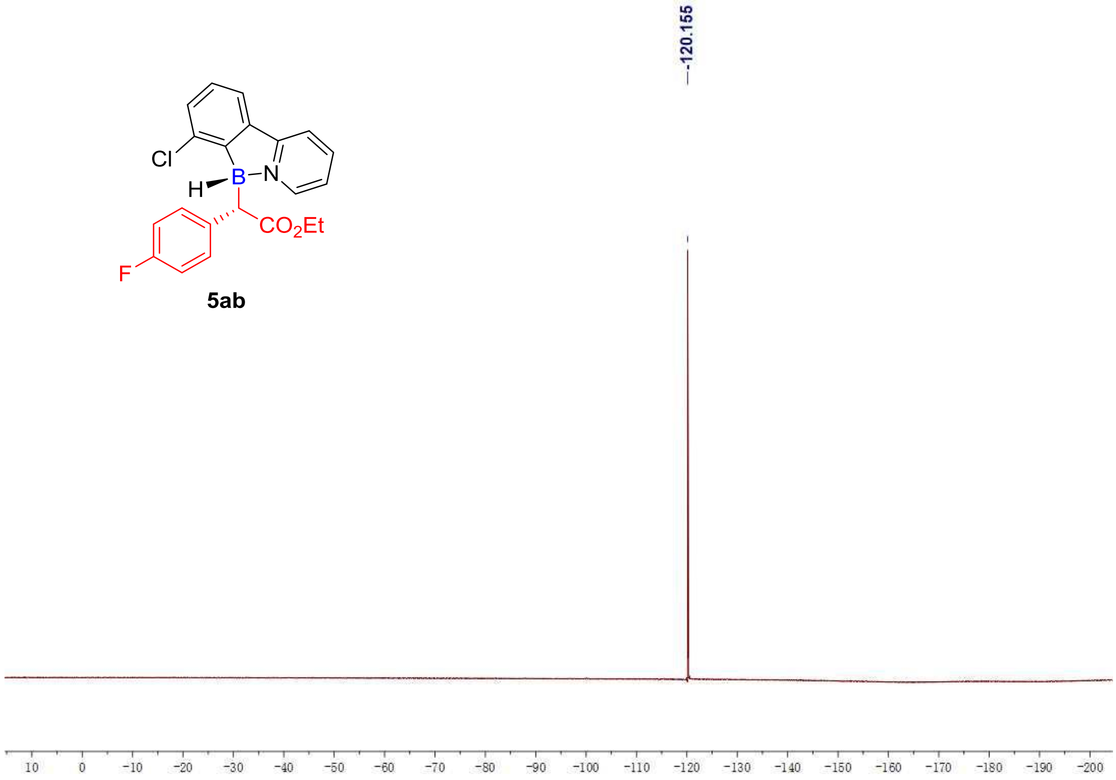

Supplementary Figure 287. ^{19}F NMR spectrum of compound 5ab

ethyl(*S*)-2-(7-chloro-6H-5 ^{4}-benzo[3,4][1,2]azaborolo[1,5-a]pyridin-6-yl)-2-(4-chlorophenyl)acetate (5ac)

^{1}H NMR (500 MHz, room temperature, $CDCl_3$)

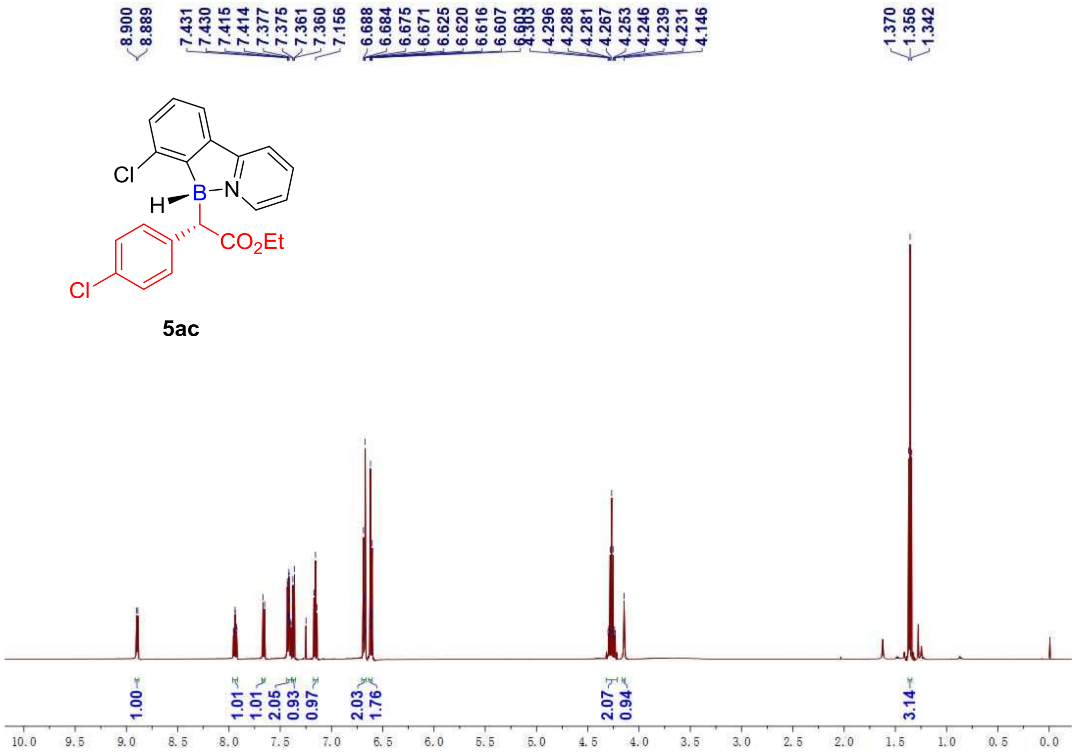

Supplementary Figure 288. ^{1}H NMR spectrum of compound 5ac

^{13}C NMR (126 MHz, room temperature, $CDCl_3$)

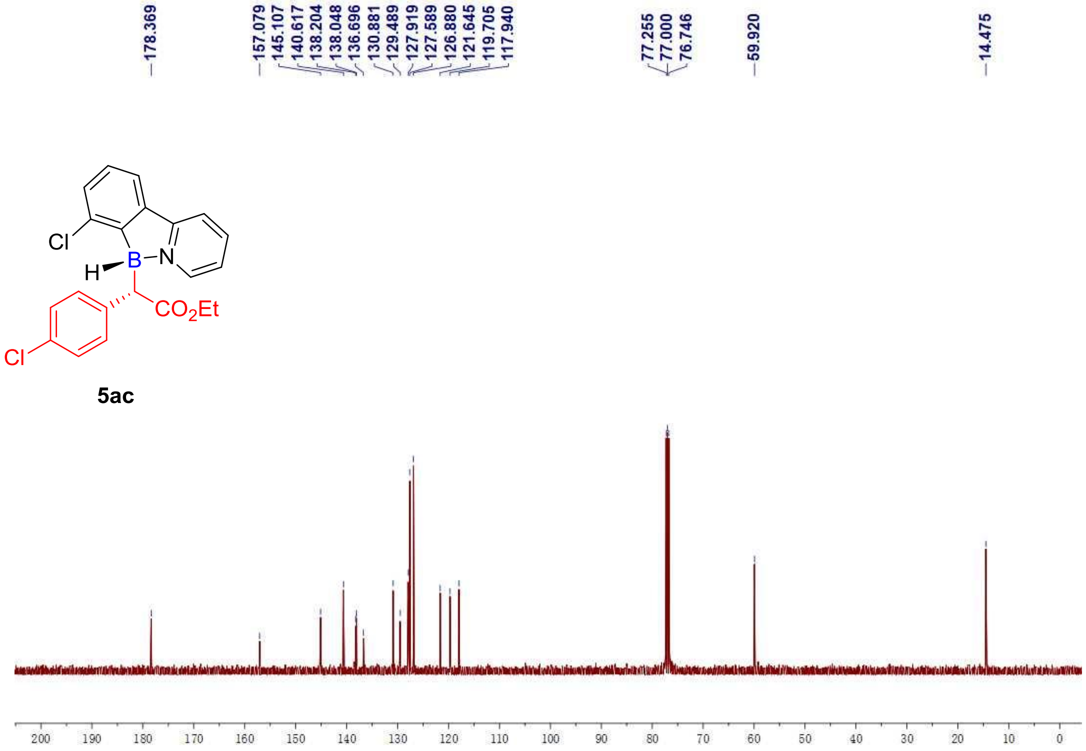

Supplementary Figure 289. ^{13}C NMR spectrum of compound 5ac

^{11}B NMR (128 MHz, room temperature, $CDCl_3$)

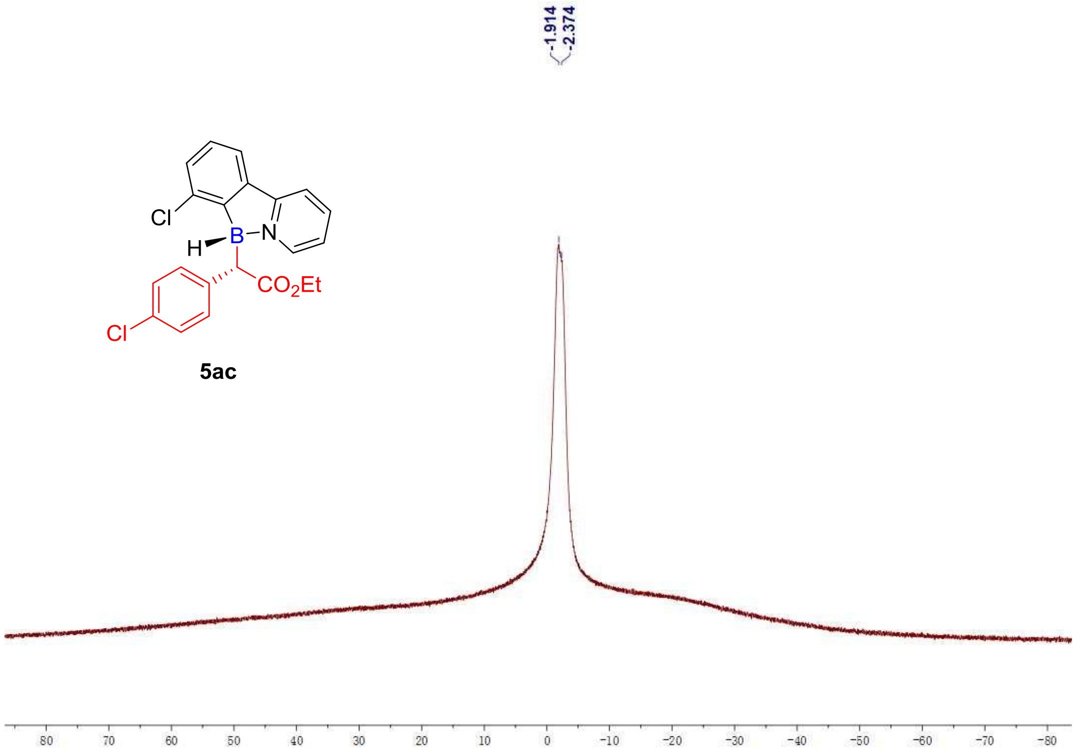

Supplementary Figure 290. ^{11}B NMR spectrum of compound 5ac

ethyl(*S*)-2-(4-bromophenyl)-2-(7-chloro-6H-5 ^{4}-benzo[3,4][1,2]azaborolo[1,5-a]pyridin-6-yl)acetate (5ad)

^{1}H NMR (400 MHz, room temperature, $CDCl_3$)

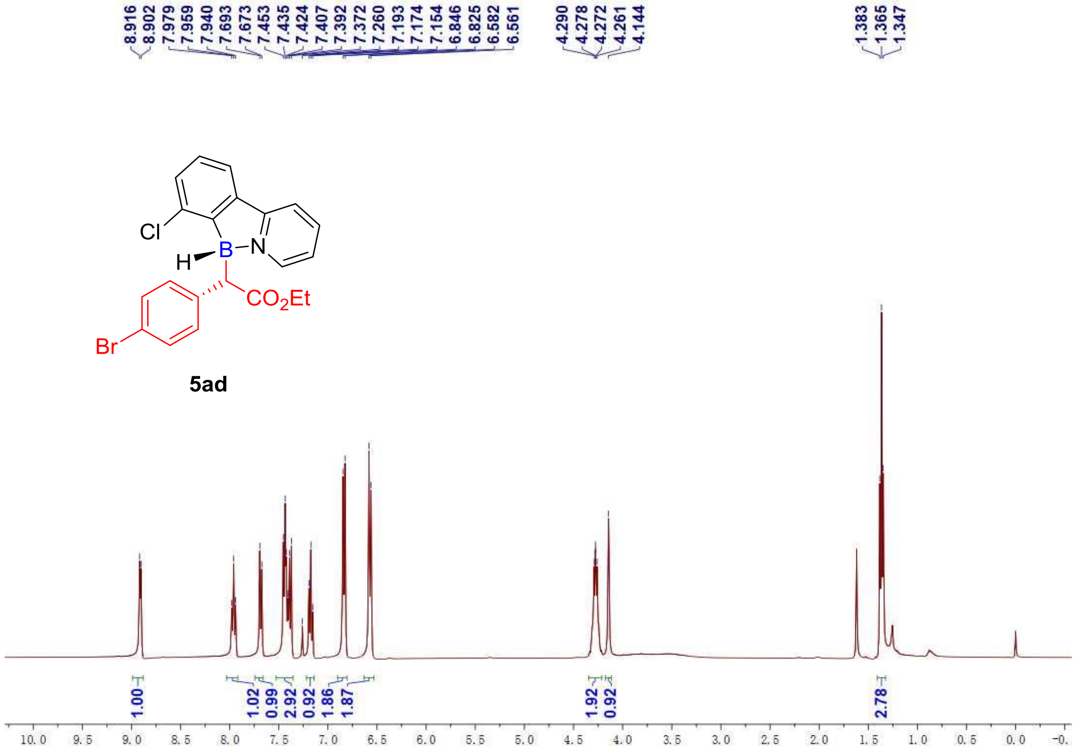

Supplementary Figure 291. ^{1}H NMR spectrum of compound 5ad

^{13}C NMR (101 MHz, room temperature, $CDCl_3$)

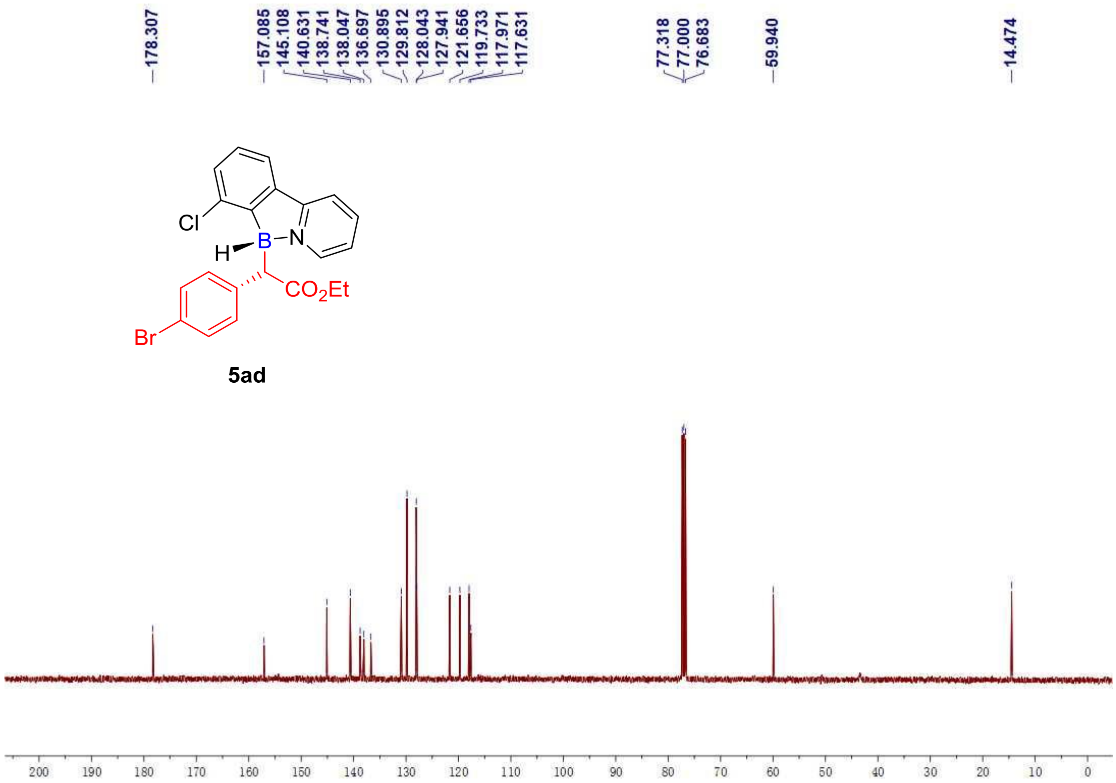

Supplementary Figure 292. ^{13}C NMR spectrum of compound 5ad

^{11}B NMR (128 MHz, room temperature, $CDCl_3$)

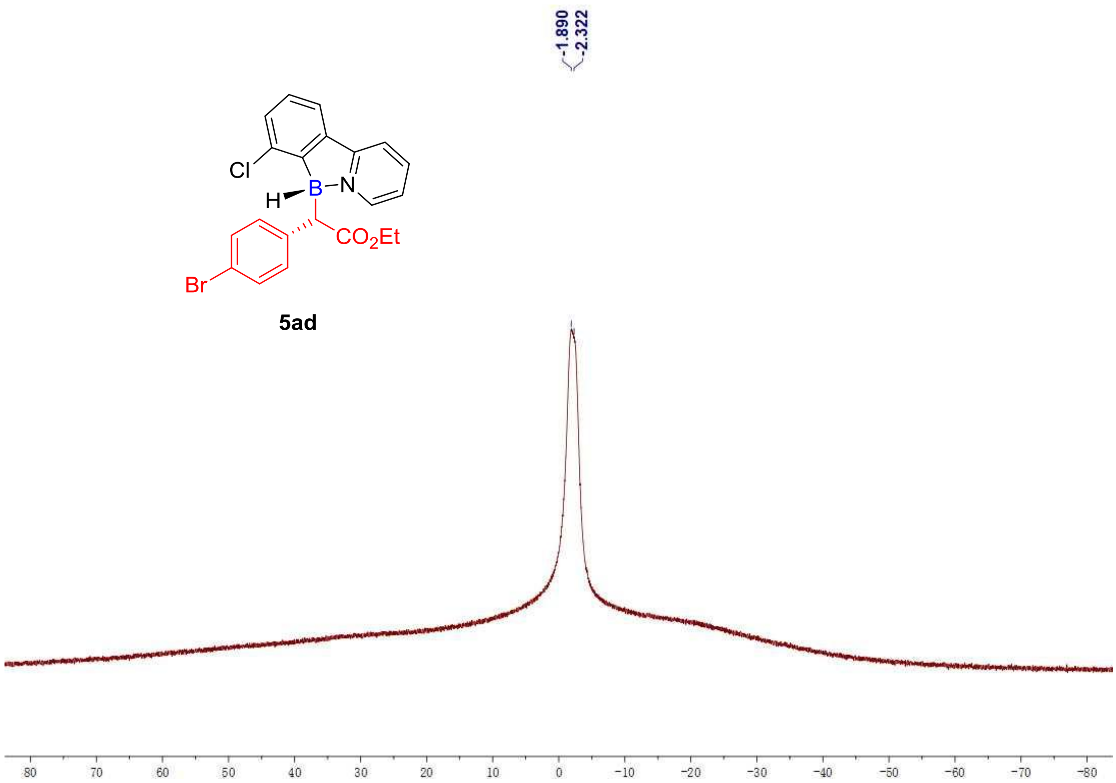

Supplementary Figure 293. NMR spectrum of compound 5ad

ethyl(*S*)-2-(7-chloro-6H-5 ^{4}-benzo[3,4][1,2]azaborolo[1,5-a]pyridin-6-yl)-2-(naphthalen-2-yl)acetate(5ae)

^{1}H NMR (400 MHz, room temperature, $CDCl_3$)

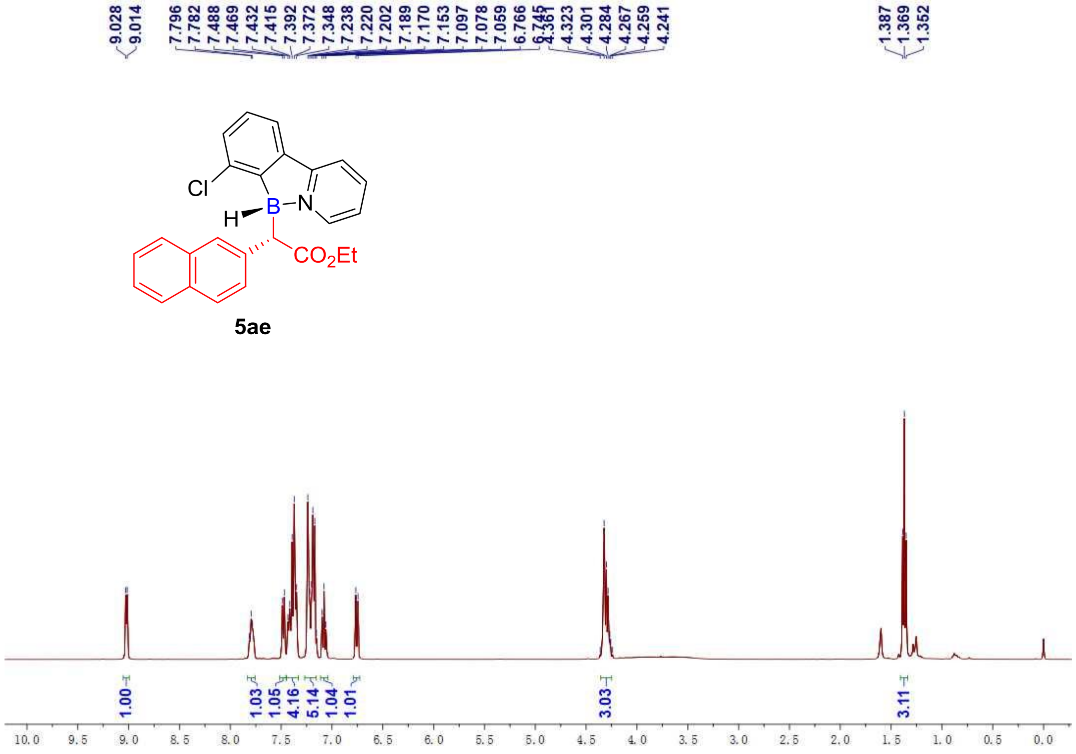

Supplementary Figure 294. ^{1}H NMR spectrum of compound 5ae

^{13}C NMR (101 MHz, room temperature, $CDCl_3$)

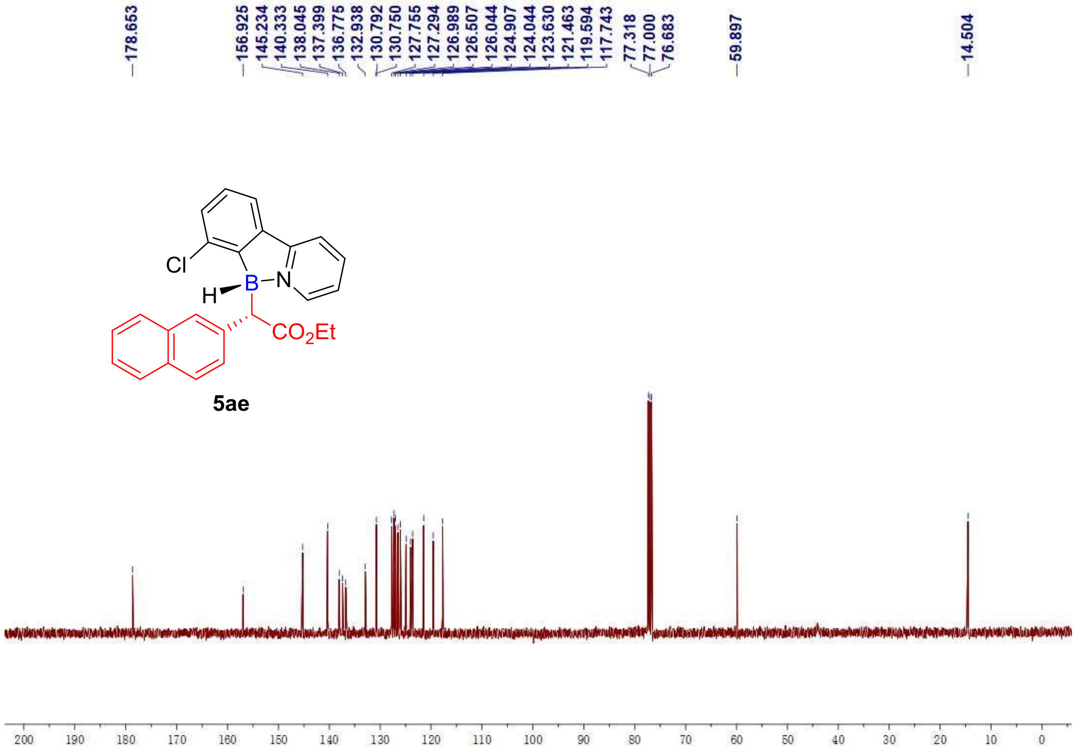

Supplementary Figure 295. ^{13}C NMR spectrum of compound 5ae

^{11}B NMR (128 MHz, room temperature, $CDCl_3$)

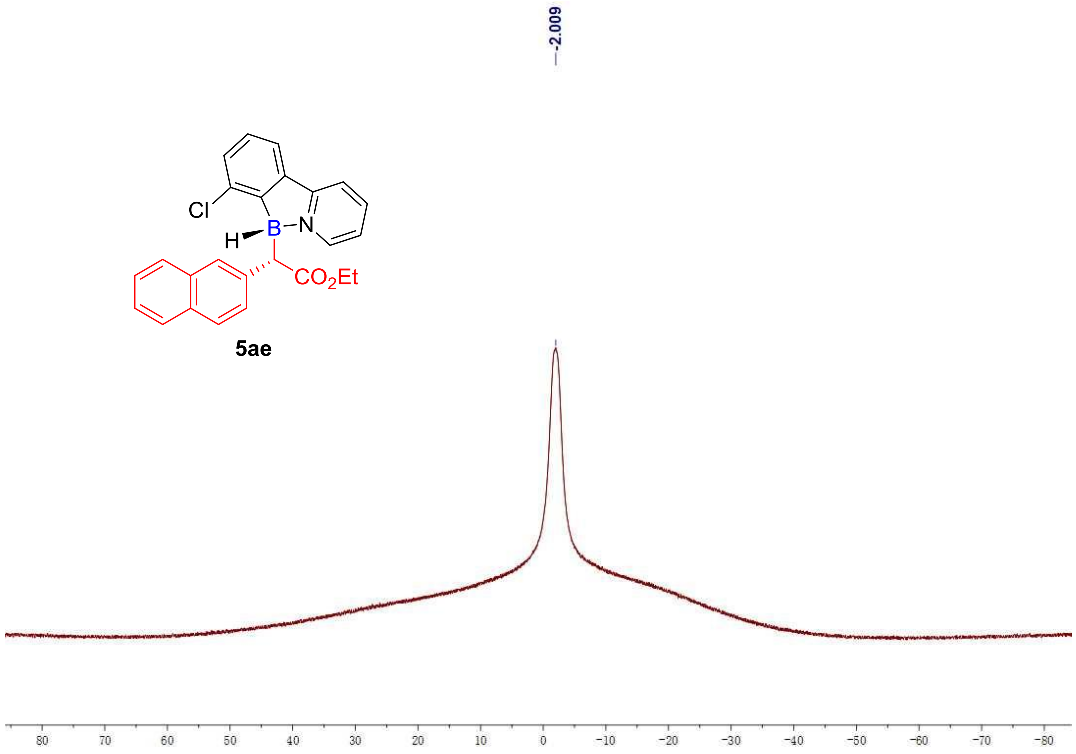

Supplementary Figure 296. ^{11}B NMR spectrum of compound 5ae

ethyl(S)-2-(7-chloro-6-(2-ethoxy-2-oxoethyl)-6H-5^{4},6^{4}-benzo[3,4][1,2]azaborolo[1,5-a]pyridin-6-yl)-2-phenylacetate (6)

^{1}H NMR (500 MHz, room temperature, $CDCl_3$)

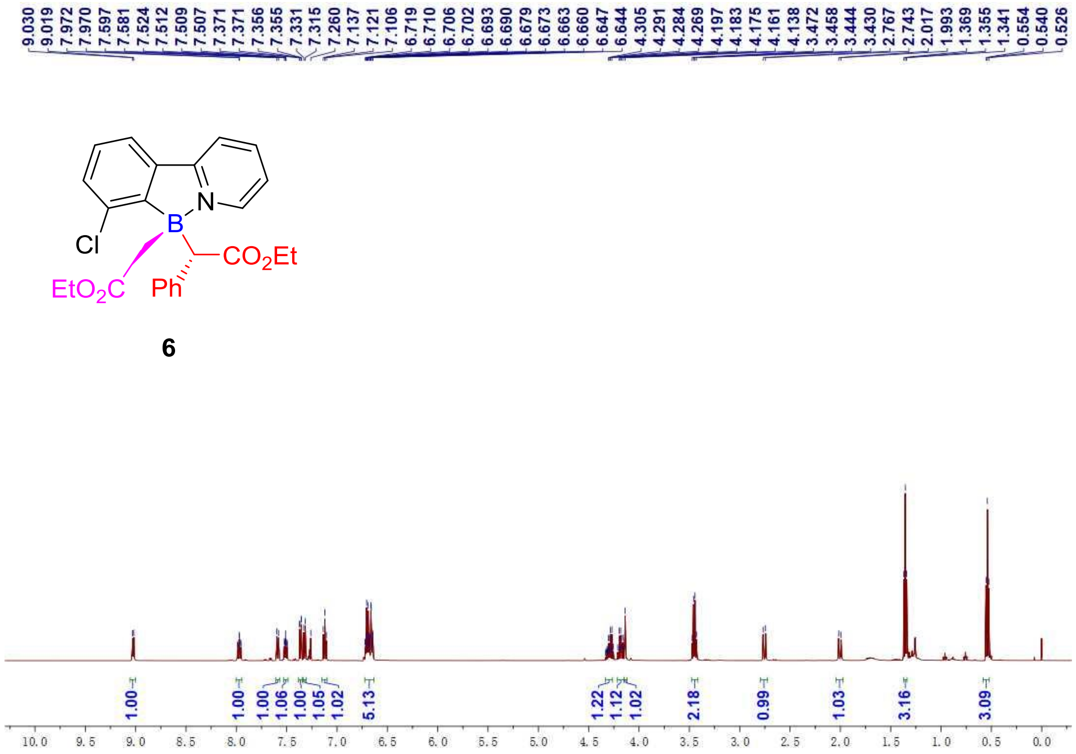

Supplementary Figure 297. ^{1}H NMR spectrum of compound 6

^{13}C NMR (126 MHz, room temperature, $CDCl_3$)

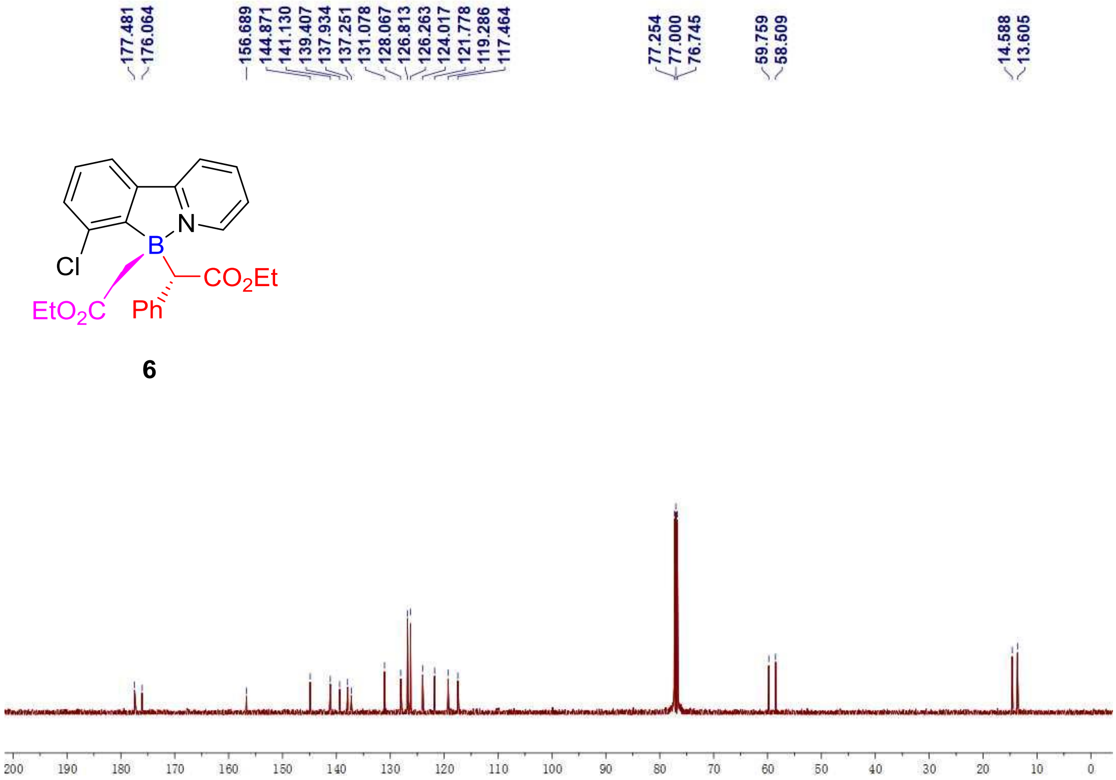

Supplementary Figure 298. ^{13}C NMR spectrum of compound 6

^{11}B NMR (128 MHz, room temperature, $CDCl_3$)

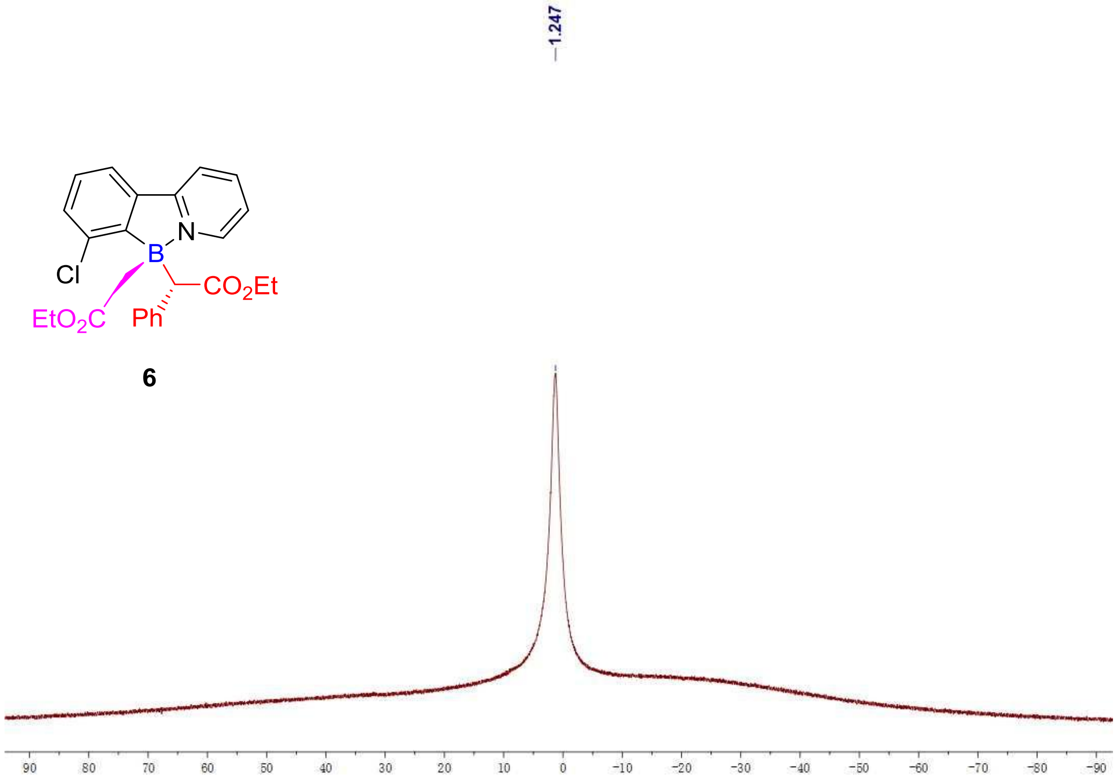

Supplementary Figure 299. ^{11}B NMR spectrum of compound 6

(S)-2-(7-chloro-6H-5 ^{4}-benzo[3,4][1,2]azaborolo[1,5-a]pyridin-6-yl)-2-phenylethan-1-ol (7)

^{1}H NMR (500 MHz, room temperature, $CDCl_3$)

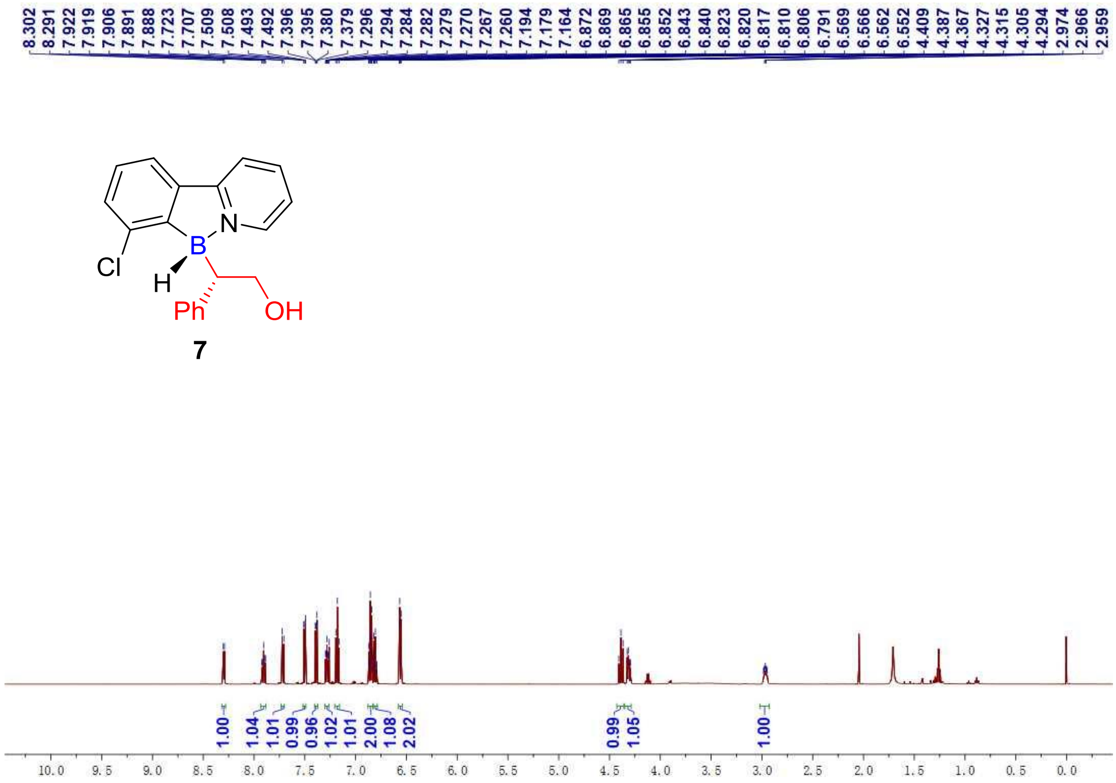

Supplementary Figure 300. ^{1}H NMR spectrum of compound 7

^{13}C NMR (126 MHz, room temperature, $CDCl_3$)

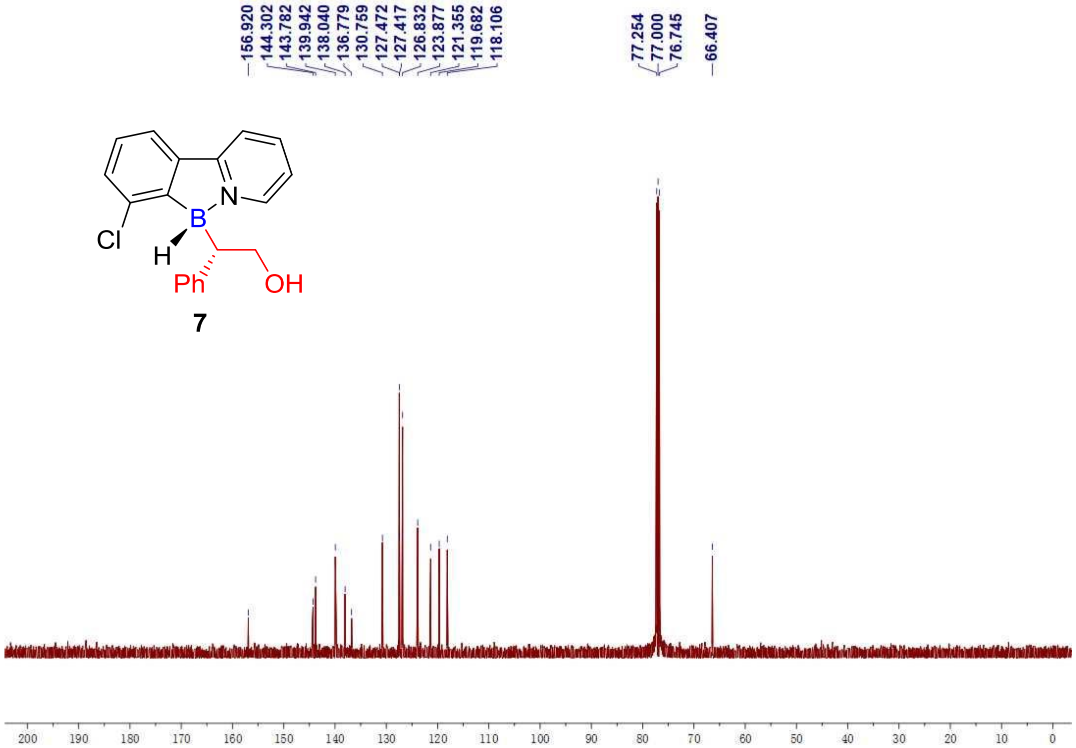

Supplementary Figure 301. ^{13}C NMR spectrum of compound 7

^{11}B NMR (128 MHz, room temperature, $CDCl_3$)

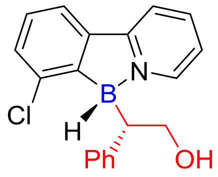

7

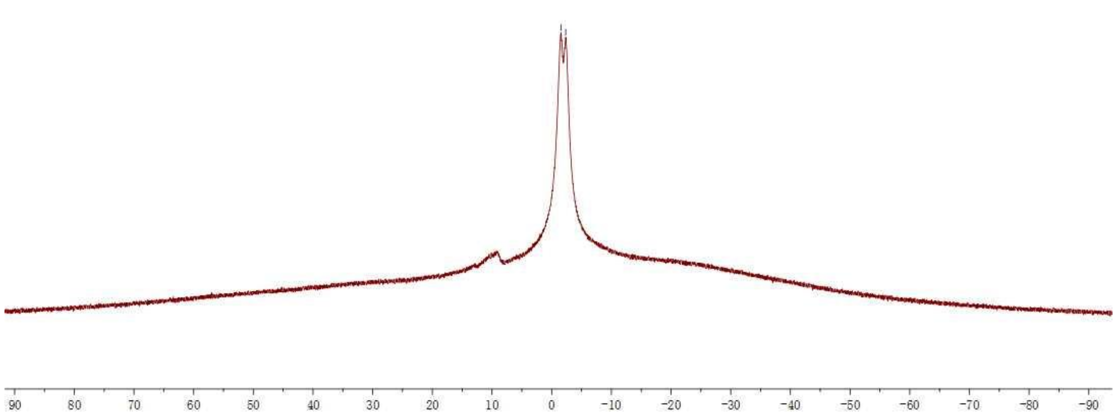

Supplementary Figure 302. ^{11}**B NMR spectrum of compound 7**

ethyl (S)-2-phenyl-2-(7-phenyl-6H-5 ^{4}-benzo[3,4][1,2]azaborolo[1,5-a]pyridin-6-yl)acetate (8)

^{1}H NMR (500 MHz, room temperature, $CDCl_3$)

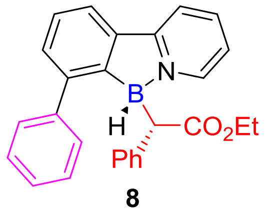

8

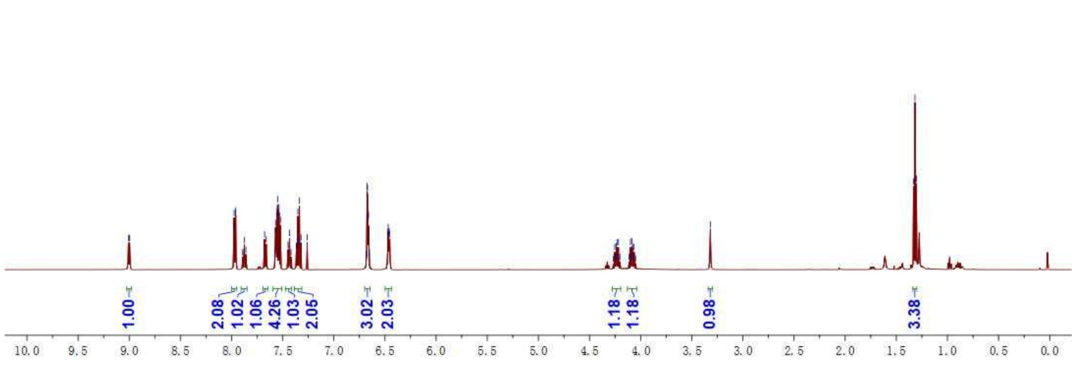

Supplementary Figure 303. ^{1}**H NMR spectrum of compound 8**

^{13}C NMR (126 MHz, room temperature, $CDCl_3$)

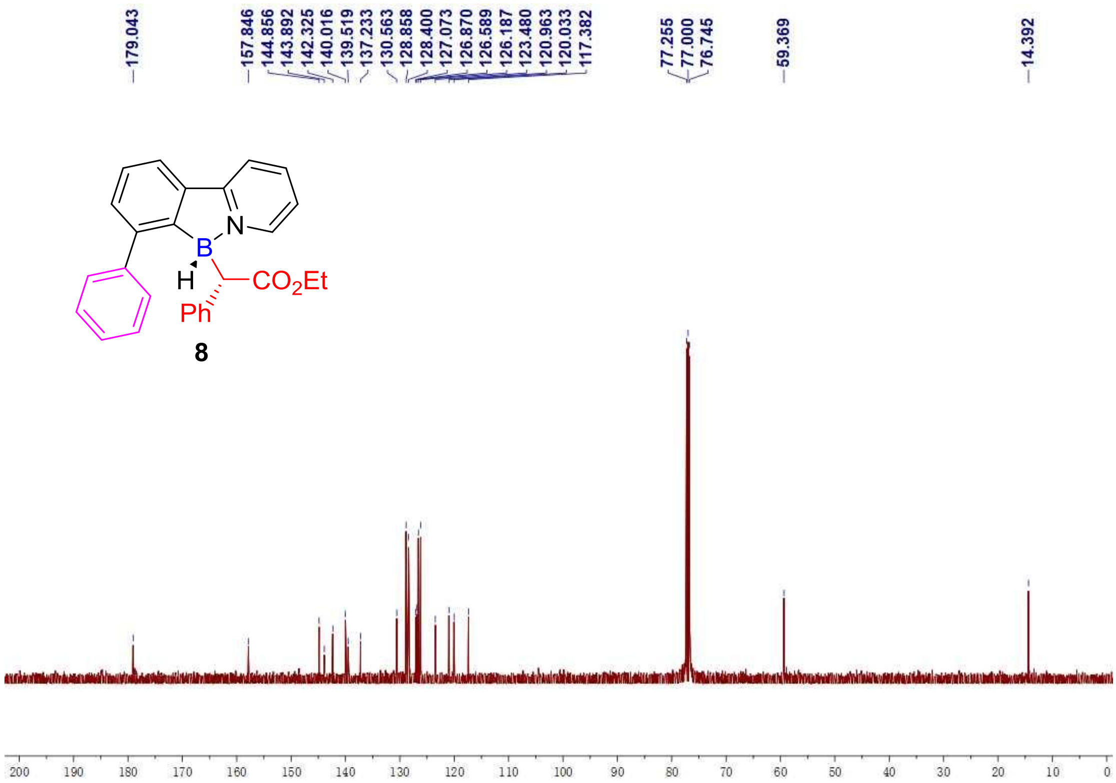

Supplementary Figure 304. ^{13}C NMR spectrum of compound 8

^{11}B NMR (128 MHz, room temperature, $CDCl_3$)

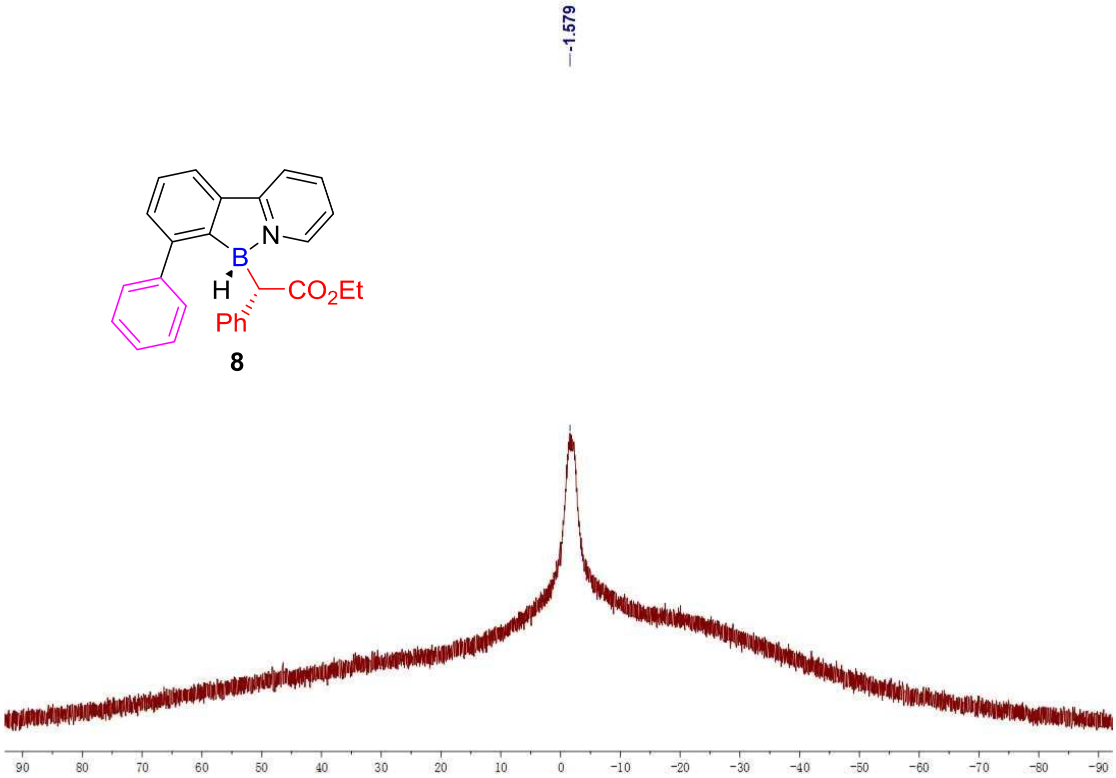

Supplementary Figure 305. ^{11}B NMR spectrum of compound 8

4.2. Chiral HPLC charts

6-benzhydryl-7-chloro-6H-5λ[4]-benzo[3,4][1,2]azaborolo[1,5-a]pyridine (3a)

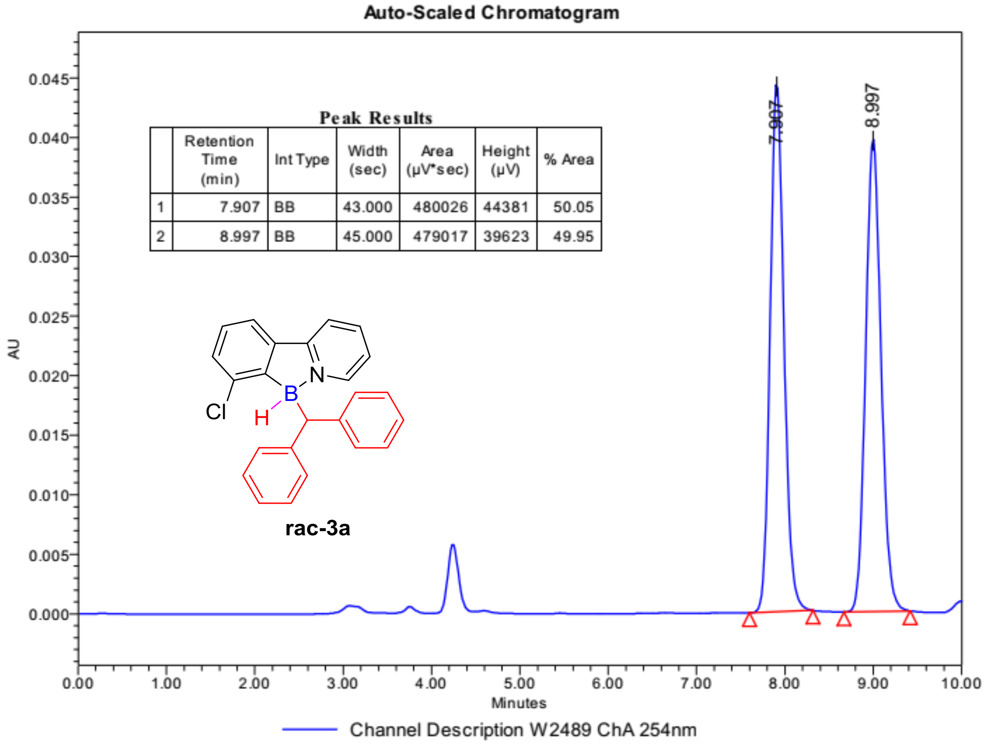

	Retention Time (min)	Int Type	Width (sec)	Area (μV*sec)	Height (μV)	% Area
1	7.907	BB	43.000	480026	44381	50.05
2	8.997	BB	45.000	479017	39623	49.95

Supplementary Figure 306. HPLC of compound rac-3a

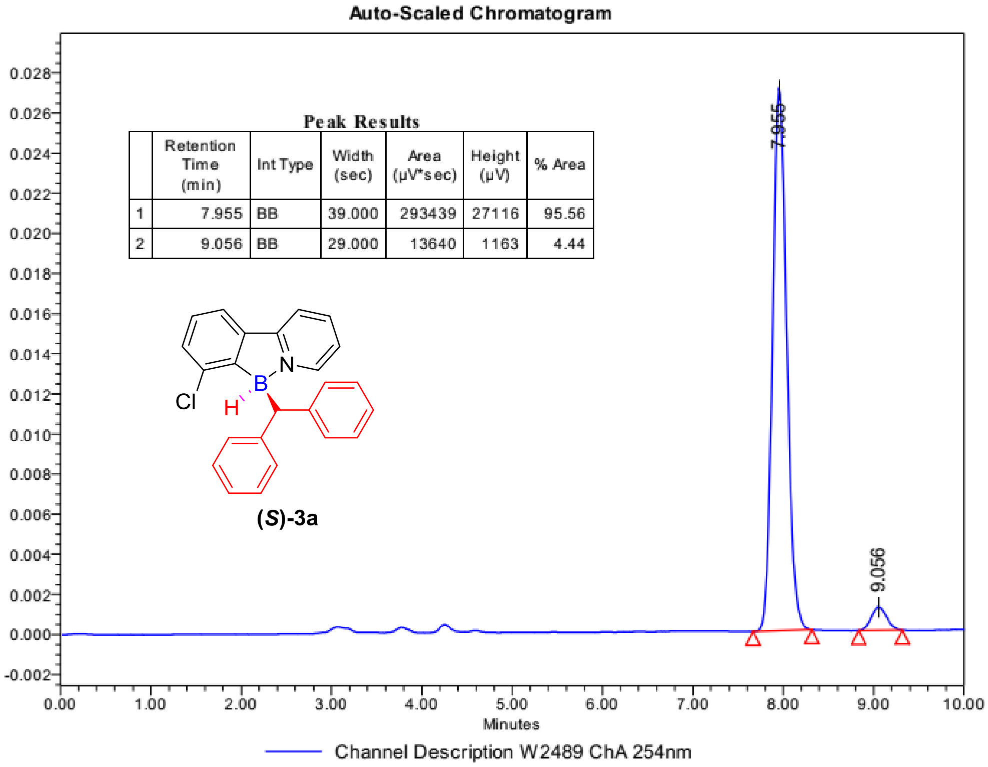

	Retention Time (min)	Int Type	Width (sec)	Area (μV*sec)	Height (μV)	% Area
1	7.955	BB	39.000	293439	27116	95.56
2	9.056	BB	29.000	13640	1163	4.44

Supplementary Figure 307. HPLC of compound (S)-3a

6-(bis(4-fluorophenyl)methyl)-7-chloro-6H-5λ[4]-benzo[3,4][1,2]azaborolo[1,5-a]pyridine (3b)

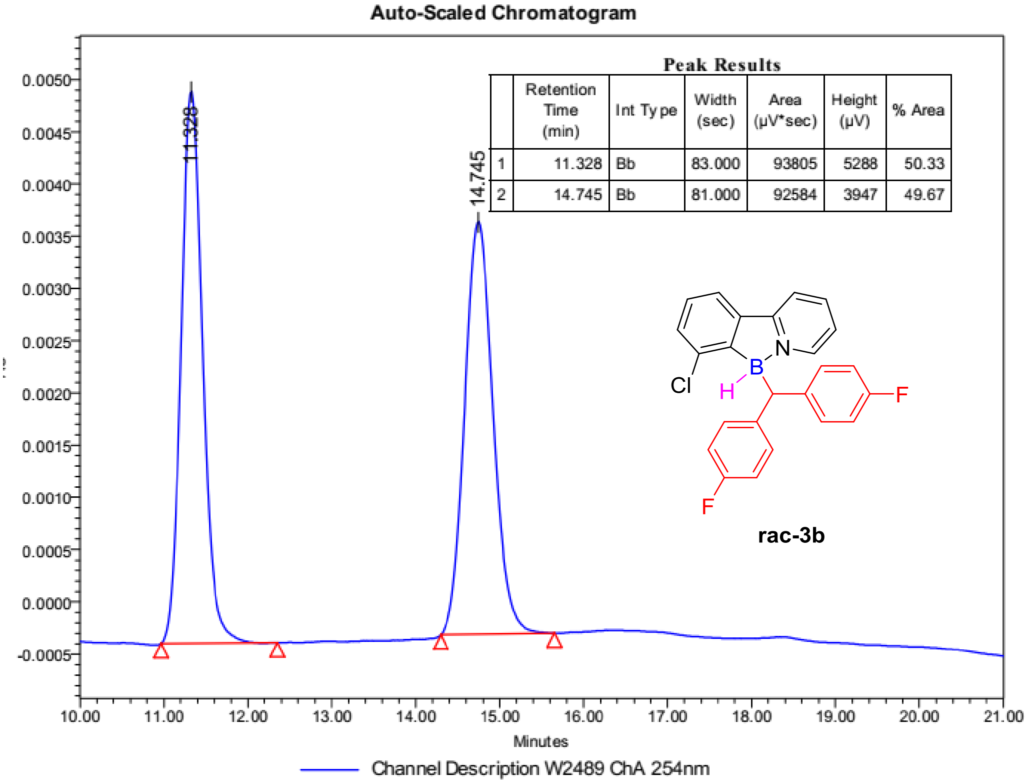

	Retention Time (min)	Int Type	Width (sec)	Area (μV*sec)	Height (μV)	% Area
1	11.328	Bb	83.000	93805	5288	50.33
2	14.745	Bb	81.000	92584	3947	49.67

Supplementary Figure 308. HPLC of compound rac-3b

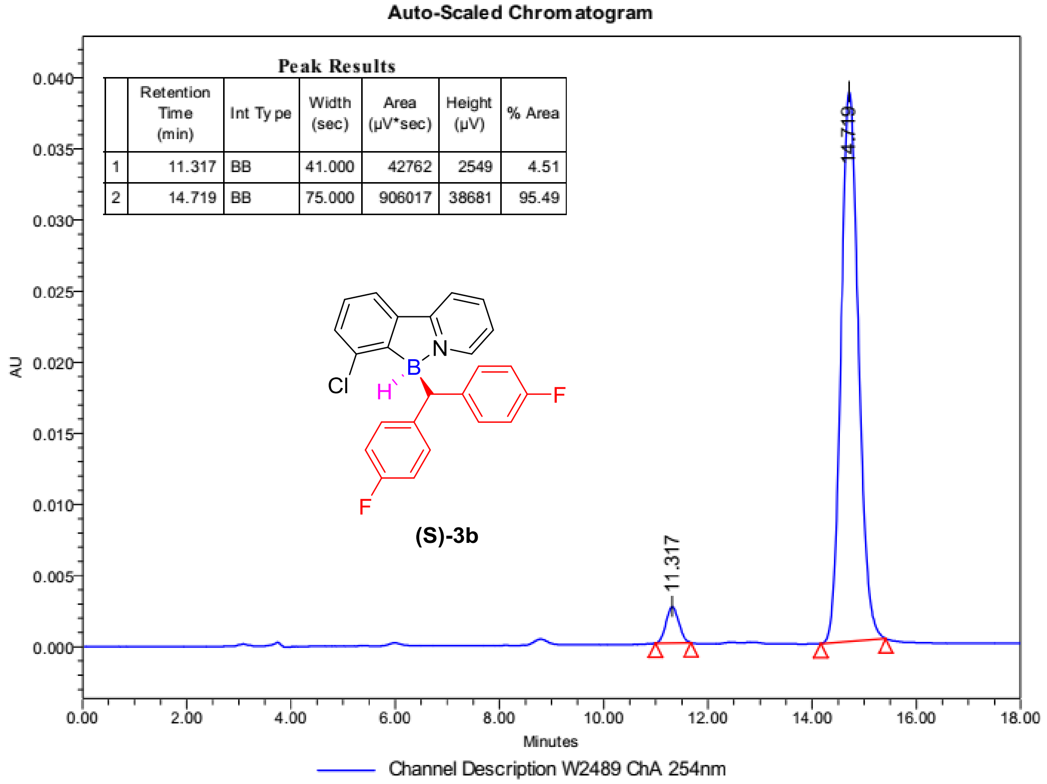

	Retention Time (min)	Int Type	Width (sec)	Area (μV*sec)	Height (μV)	% Area
1	11.317	BB	41.000	42762	2549	4.51
2	14.719	BB	75.000	906017	38681	95.49

Supplementary Figure 309. HPLC of compound (S)-3b

6-(bis(4-chlorophenyl)methyl)-7-chloro-6H-5λ[4]-benzo[3,4][1,2]azaborolo[1,5-a]pyridine (3c)

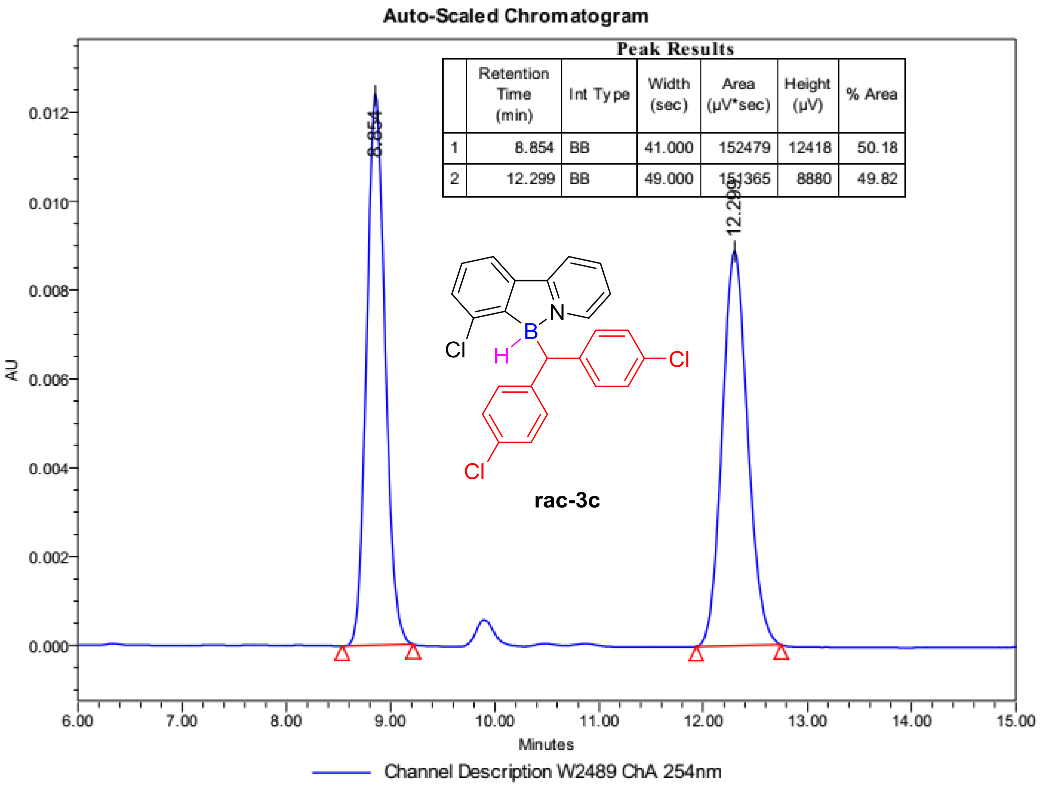

Peak Results

	Retention Time (min)	Int Type	Width (sec)	Area (μV*sec)	Height (μV)	% Area
1	8.854	BB	41.000	152479	12418	50.18
2	12.299	BB	49.000	151365	8880	49.82

Supplementary Figure 310. HPLC of compound rac-3c

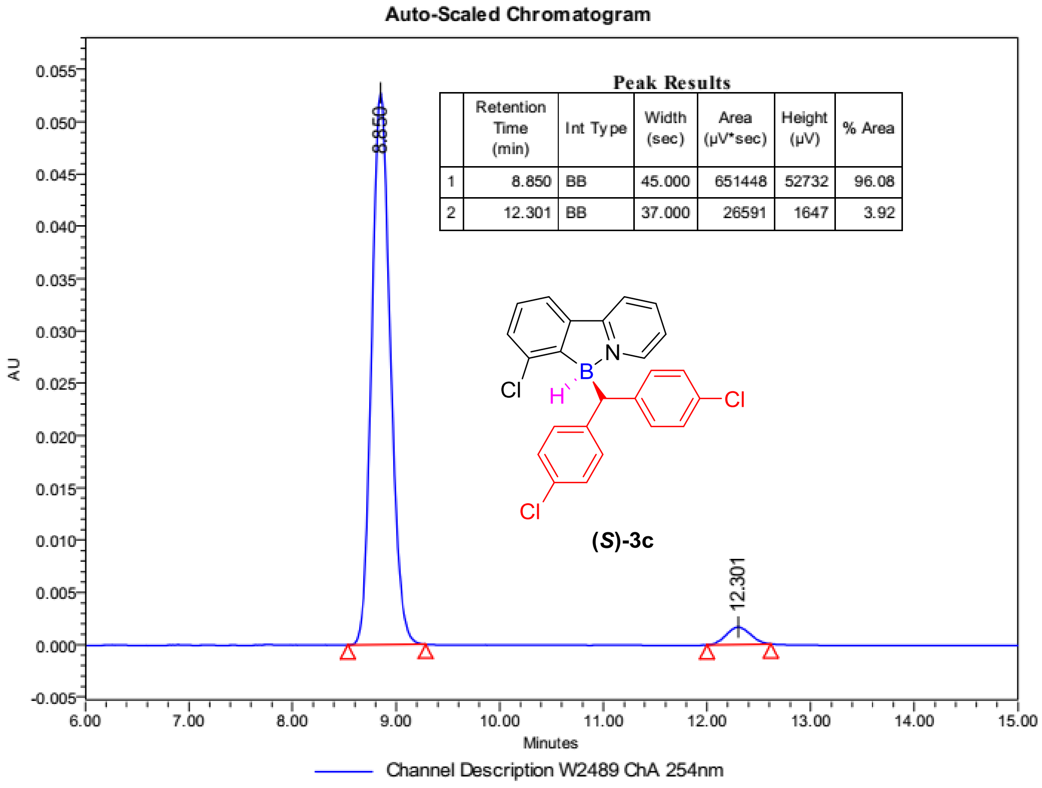

Peak Results

	Retention Time (min)	Int Type	Width (sec)	Area (μV*sec)	Height (μV)	% Area
1	8.850	BB	45.000	651448	52732	96.08
2	12.301	BB	37.000	26591	1647	3.92

Supplementary Figure 311. HPLC of compound (S)-3c

6-(bis(3-chlorophenyl)methyl)-7-chloro-6H-5λ4-benzo[3,4][1,2]azaborolo[1,5-a]pyridine (3d)

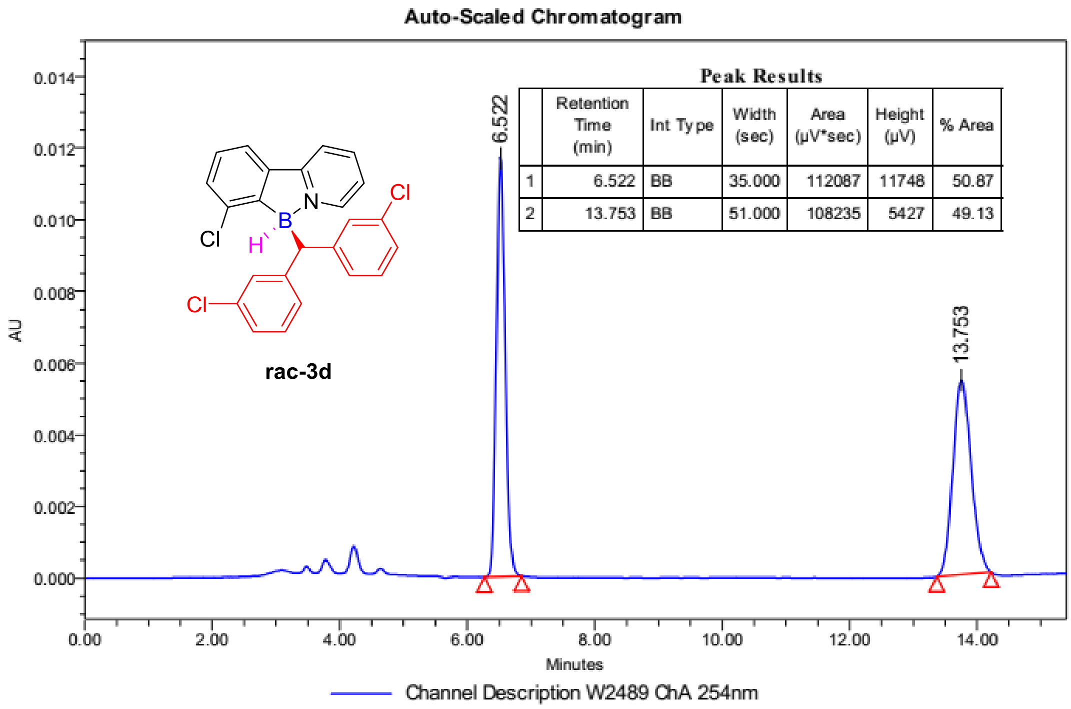

Peak Results

	Retention Time (min)	Int Type	Width (sec)	Area (μV*sec)	Height (μV)	% Area
1	6.522	BB	35.000	112087	11748	50.87
2	13.753	BB	51.000	108235	5427	49.13

Supplementary Figure 312. HPLC of compound rac-3d

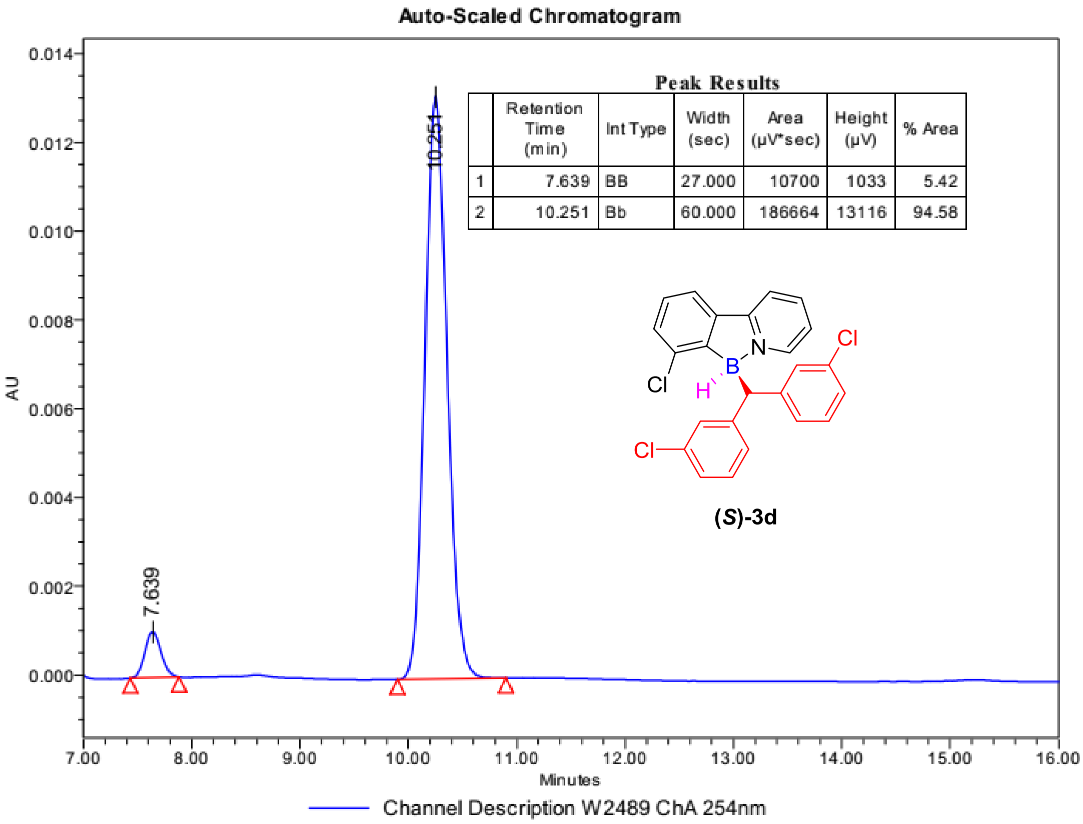

Peak Results

	Retention Time (min)	Int Type	Width (sec)	Area (μV*sec)	Height (μV)	% Area
1	7.639	BB	27.000	10700	1033	5.42
2	10.251	Bb	60.000	186664	13116	94.58

Supplementary Figure 313. HPLC of compound (S)-3d

6-(bis(4-methoxyphenyl)methyl)-7-chloro-6H-5λ[4]-benzo[3,4][1,2]azaborolo[1,5-a]pyridine (3e)

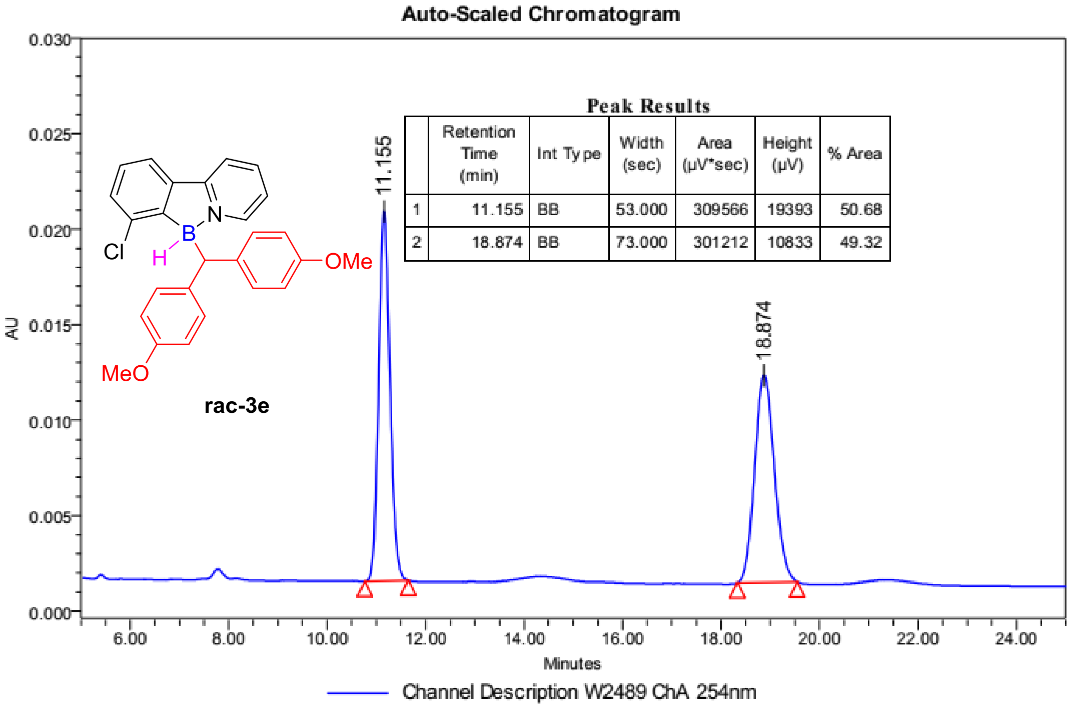

	Retention Time (min)	Int Type	Width (sec)	Area (μV*sec)	Height (μV)	% Area
1	11.155	BB	53.000	309566	19393	50.68
2	18.874	BB	73.000	301212	10833	49.32

Supplementary Figure 314. HPLC of compound rac-3e

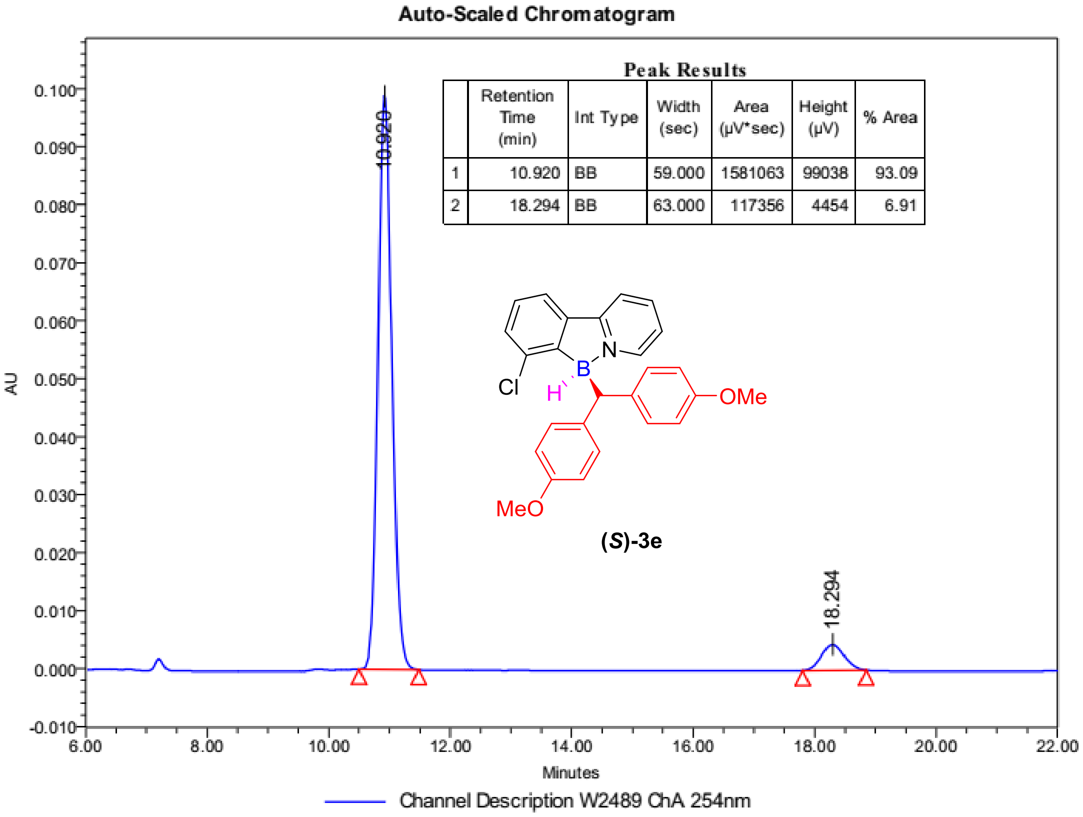

	Retention Time (min)	Int Type	Width (sec)	Area (μV*sec)	Height (μV)	% Area
1	10.920	BB	59.000	1581063	99038	93.09
2	18.294	BB	63.000	117356	4454	6.91

Supplementary Figure 315. HPLC of compound (S)-3e

6-(bis(3-methoxyphenyl)methyl)-7-chloro-6H-5λ[4]-benzo[3,4][1,2]azaborolo[1,5-a]pyridine (3f)

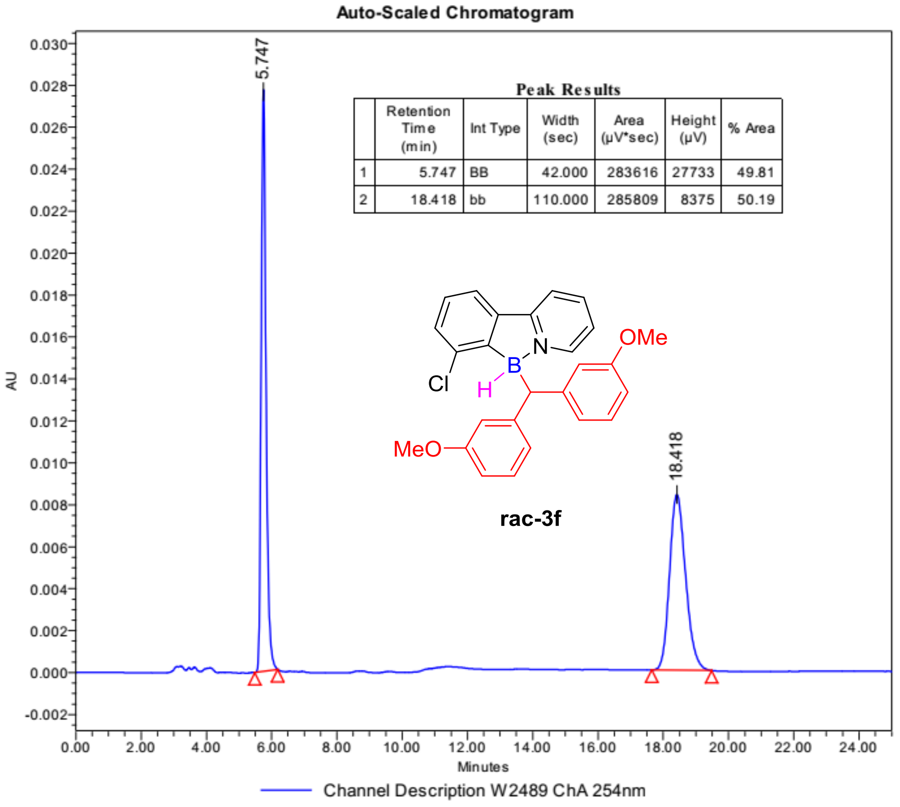

	Retention Time (min)	Int Type	Width (sec)	Area (µV*sec)	Height (µV)	% Area
1	5.747	BB	42.000	283616	27733	49.81
2	18.418	bb	110.000	285809	8375	50.19

Supplementary Figure 316. HPLC of compound rac-3f

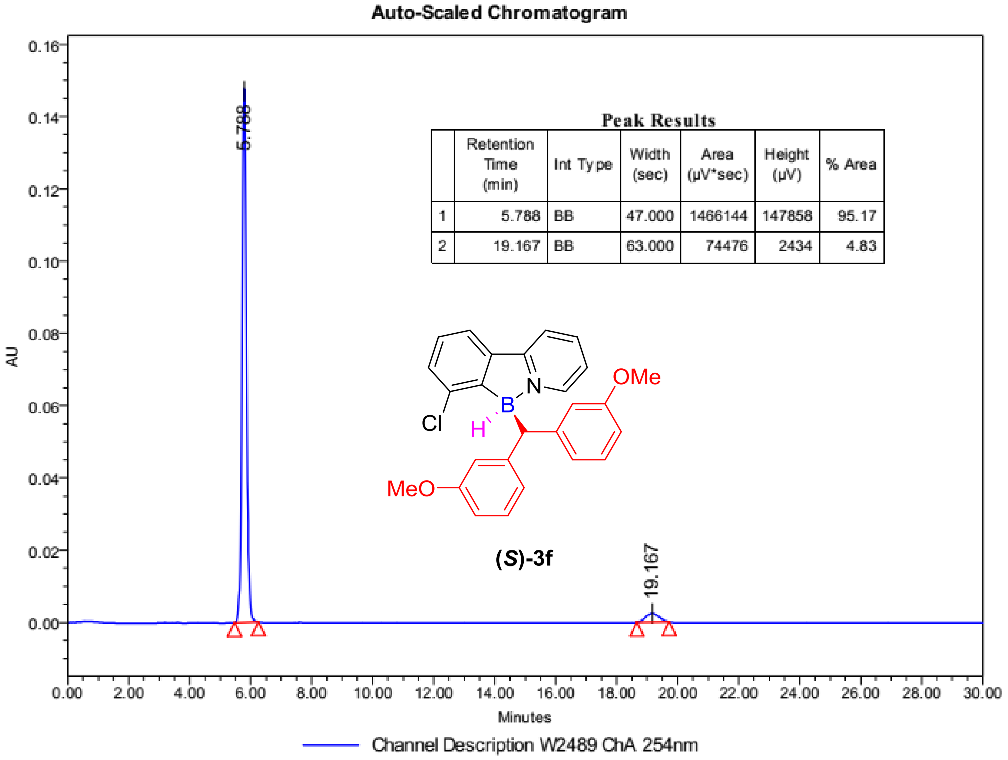

	Retention Time (min)	Int Type	Width (sec)	Area (µV*sec)	Height (µV)	% Area
1	5.788	BB	47.000	1466144	147858	95.17
2	19.167	BB	63.000	74476	2434	4.83

Supplementary Figure 317. HPLC of compound (S)-3f

7-chloro-6-(di-m-tolylmethyl)-6H-5λ[4]-benzo[3,4][1,2]azaborolo[1,5-a]pyridine (3g)

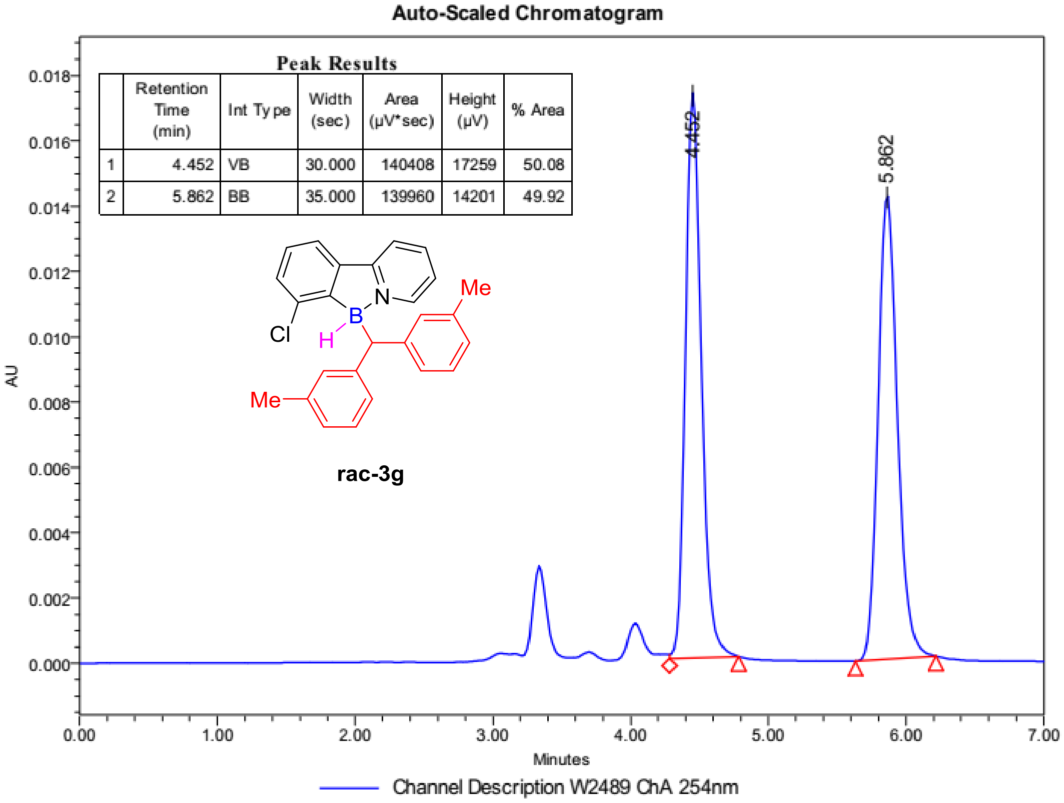

	Retention Time (min)	Int Type	Width (sec)	Area (μV*sec)	Height (μV)	% Area
1	4.452	VB	30.000	140408	17259	50.08
2	5.862	BB	35.000	139960	14201	49.92

Supplementary Figure 318. HPLC of compound rac-3g

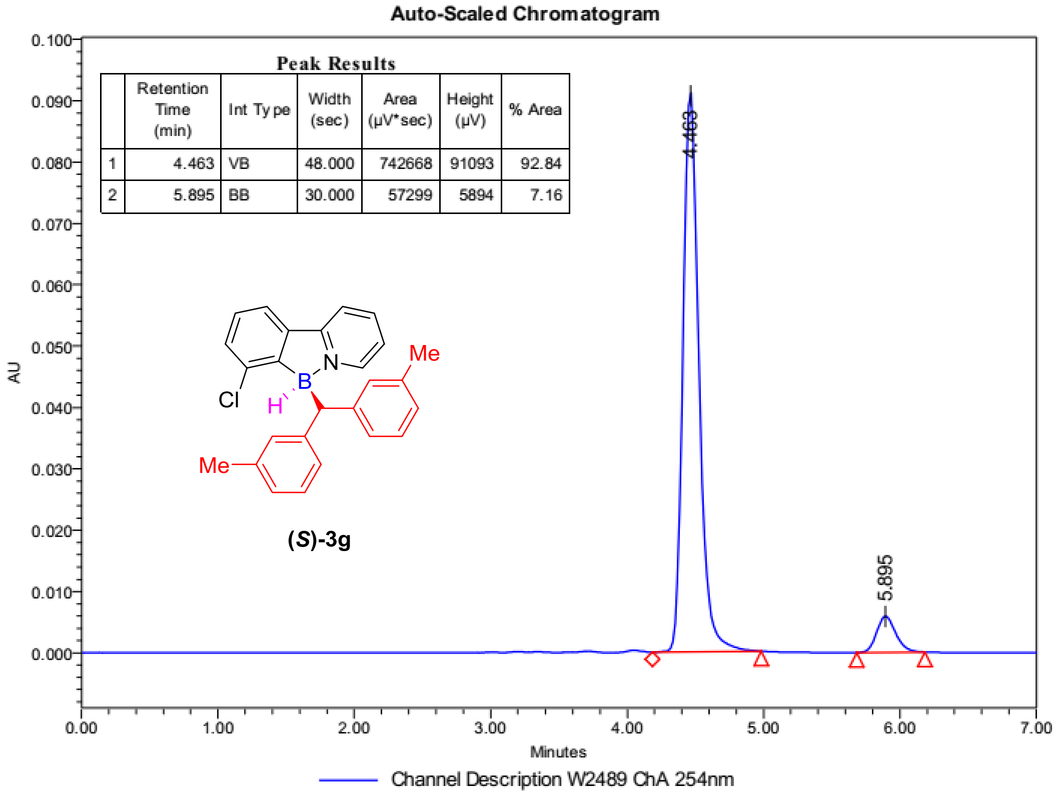

	Retention Time (min)	Int Type	Width (sec)	Area (μV*sec)	Height (μV)	% Area
1	4.463	VB	48.000	742668	91093	92.84
2	5.895	BB	30.000	57299	5894	7.16

Supplementary Figure 319. HPLC of compound (S)-3g

7-chloro-6-(2,7-dibromo-9H-fluoren-9-yl)-6H-5λ^4-benzo[3,4][1,2]azaborolo[1,5-a]pyridine (3h)

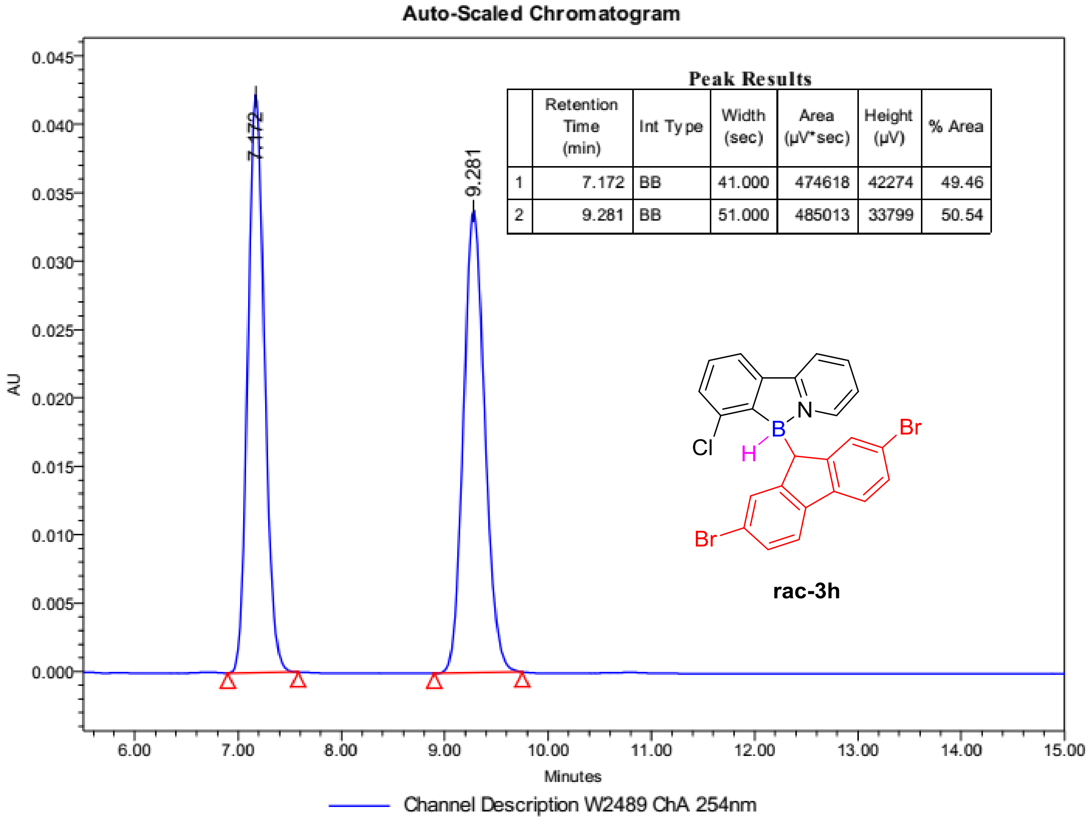

Peak Results

	Retention Time (min)	Int Type	Width (sec)	Area (µV*sec)	Height (µV)	% Area
1	7.172	BB	41.000	474618	42274	49.46
2	9.281	BB	51.000	485013	33799	50.54

Supplementary Figure 320. HPLC of compound rac-3h

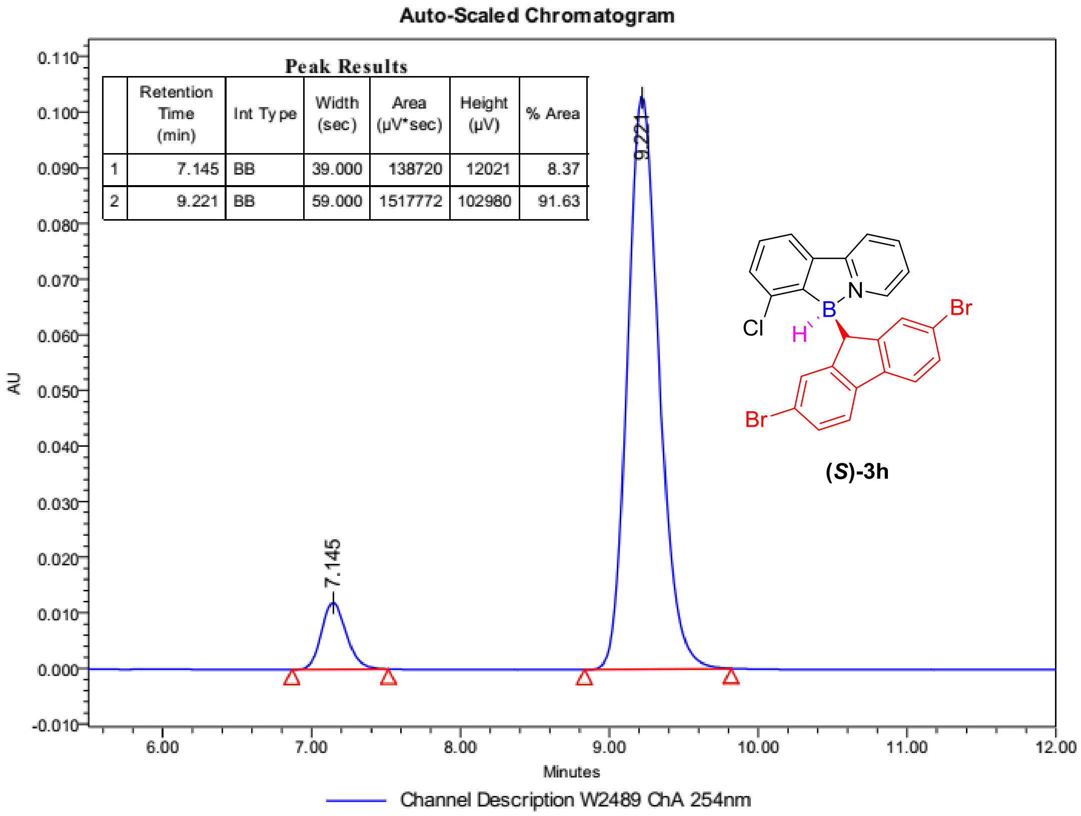

Peak Results

	Retention Time (min)	Int Type	Width (sec)	Area (µV*sec)	Height (µV)	% Area
1	7.145	BB	39.000	138720	12021	8.37
2	9.221	BB	59.000	1517772	102980	91.63

Supplementary Figure 321. HPLC of compound (S)-3h

7-chloro-6-(9H-fluoren-9-yl)-6H-5λ^4-benzo[3,4][1,2]azaborolo[1,5-a]pyridine (3i)

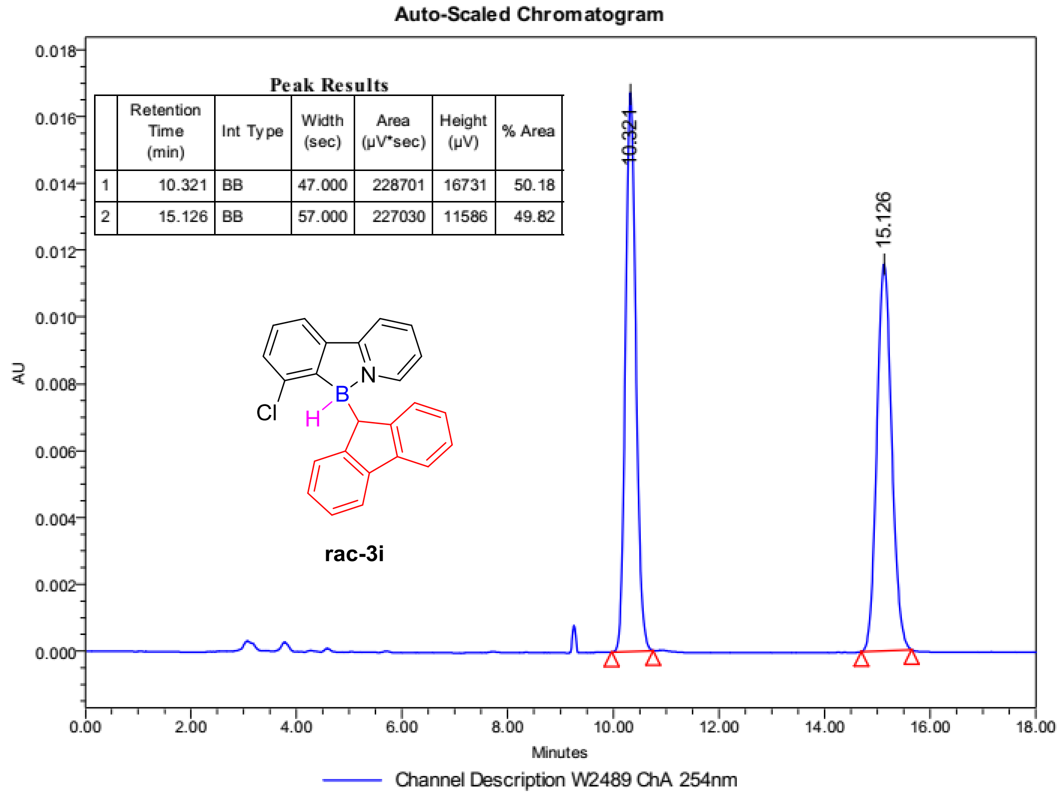

Supplementary Figure 322. HPLC of compound rac-3i

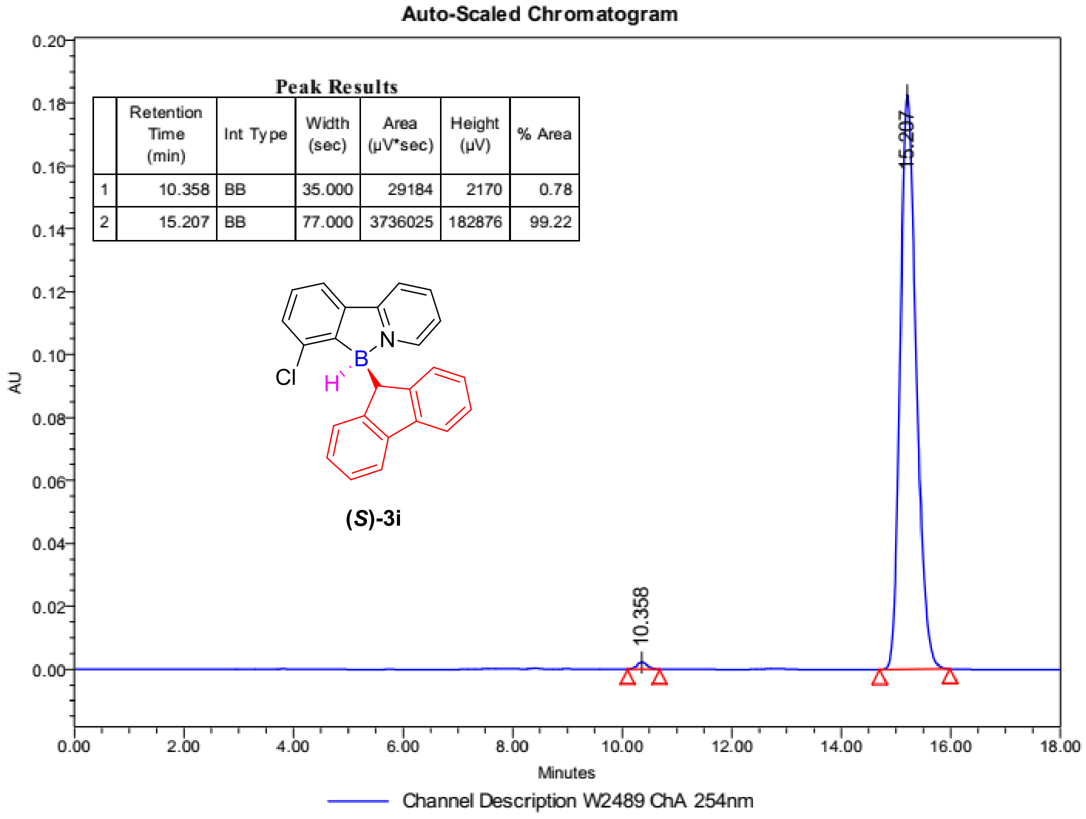

Supplementary Figure 323. HPLC of compound (S)-3i

7-chloro-6-(9H-thioxanthen-9-yl)-6H-5λ[4]-benzo[3,4][1,2]azaborolo[1,5-a]pyridine (3j)

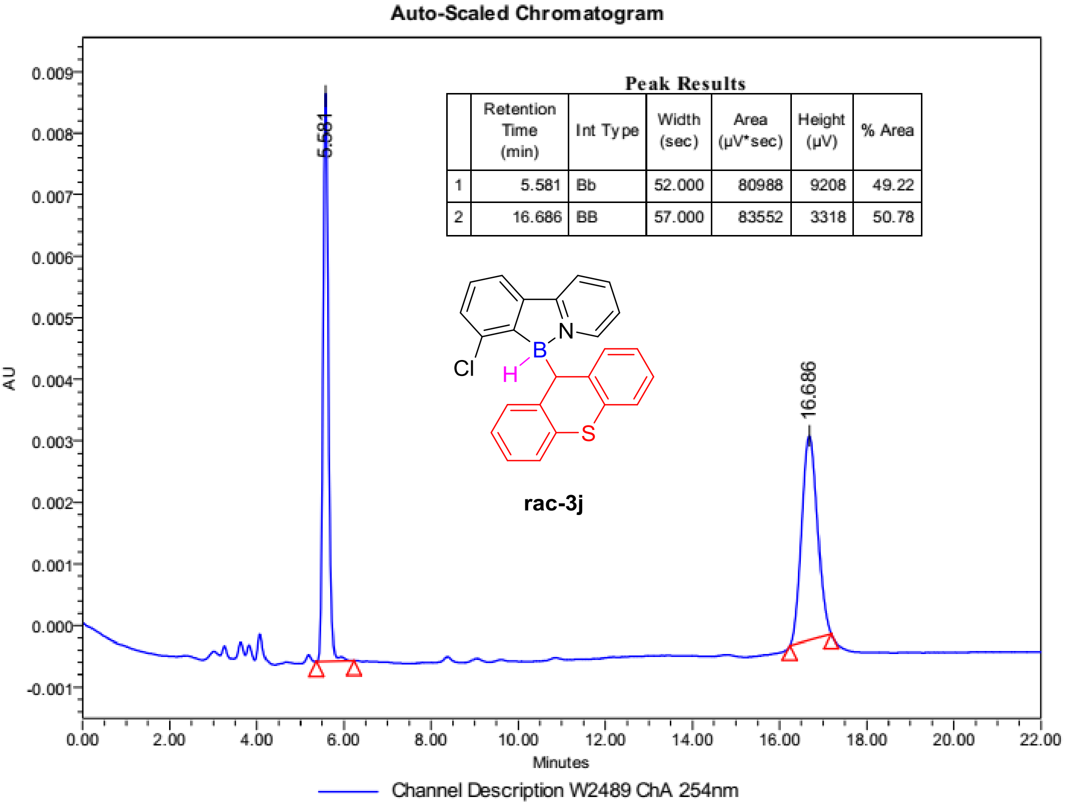

	Retention Time (min)	Int Type	Width (sec)	Area (μV*sec)	Height (μV)	% Area
1	5.581	Bb	52.000	80988	9208	49.22
2	16.686	BB	57.000	83552	3318	50.78

Supplementary Figure 324. HPLC of compound rac-3j

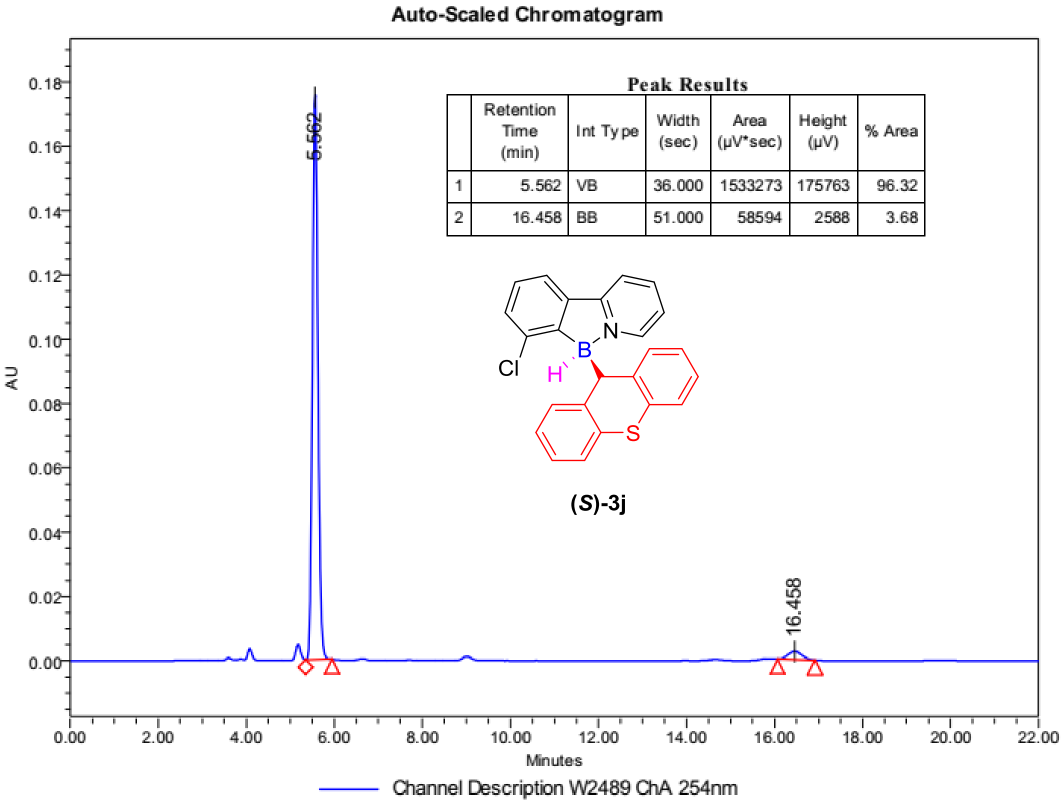

	Retention Time (min)	Int Type	Width (sec)	Area (μV*sec)	Height (μV)	% Area
1	5.562	VB	36.000	1533273	175763	96.32
2	16.458	BB	51.000	58594	2588	3.68

Supplementary Figure 325. HPLC of compound (S)-3j

10-(7-chloro-6H-5λ[4]-benzo[3,4][1,2]azaborolo[1,5-a]pyridin-6-yl)anthracen-9(10H)-one (3k)

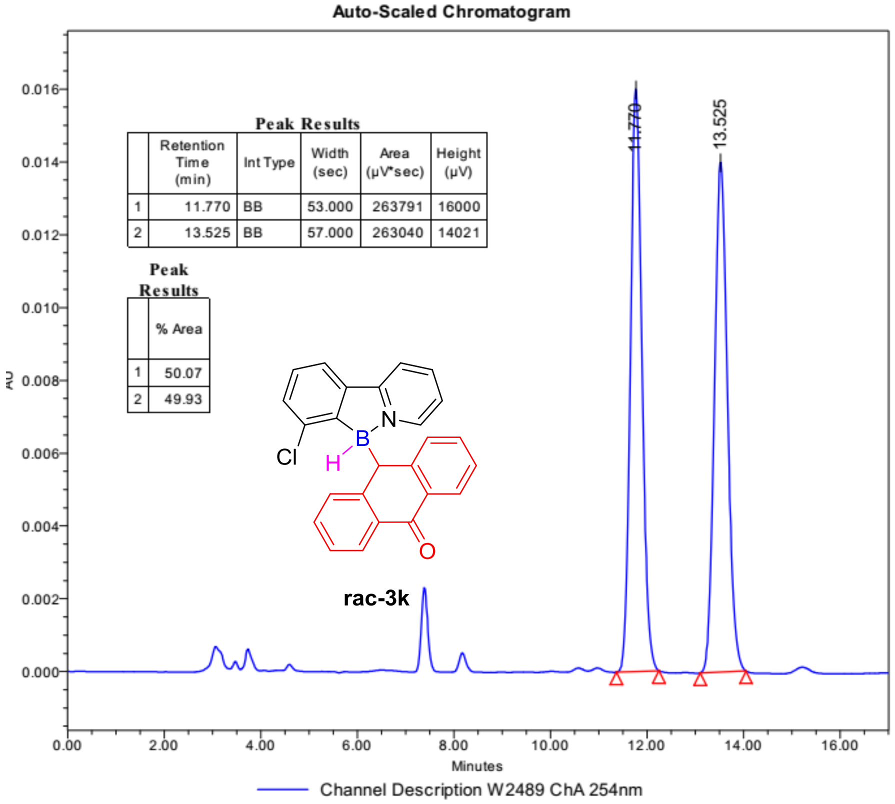

Supplementary Figure 326. HPLC of compound rac-3k

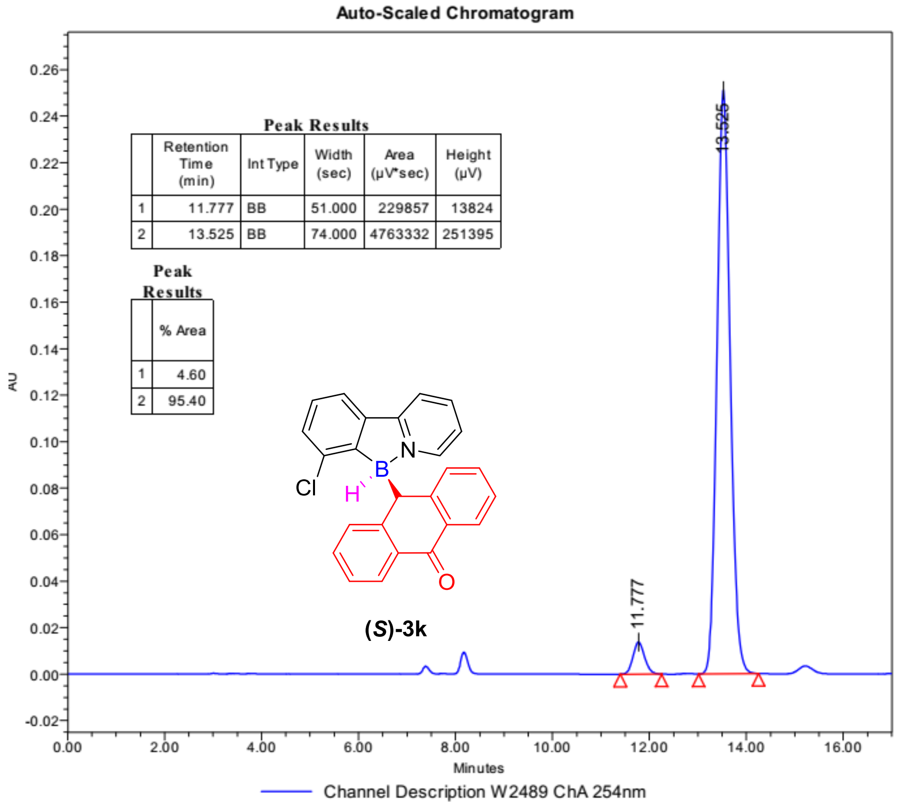

Supplementary Figure 327. HPLC of compound (S)-3k

6-benzhydryl-7-fluoro-6H-5λ[4]-benzo[3,4][1,2]azaborolo[1,5-a]pyridine (3l)

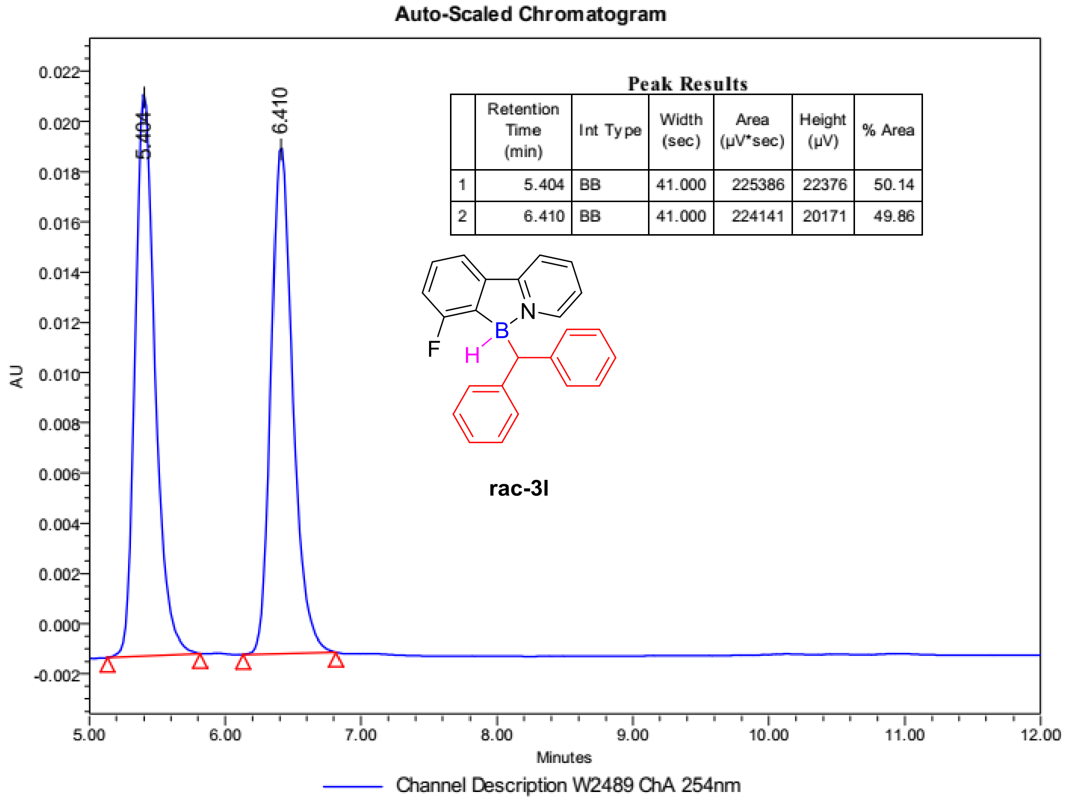

	Retention Time (min)	Int Type	Width (sec)	Area (µV*sec)	Height (µV)	% Area
1	5.404	BB	41.000	225386	22376	50.14
2	6.410	BB	41.000	224141	20171	49.86

Supplementary Figure 328. HPLC of compound rac-3l

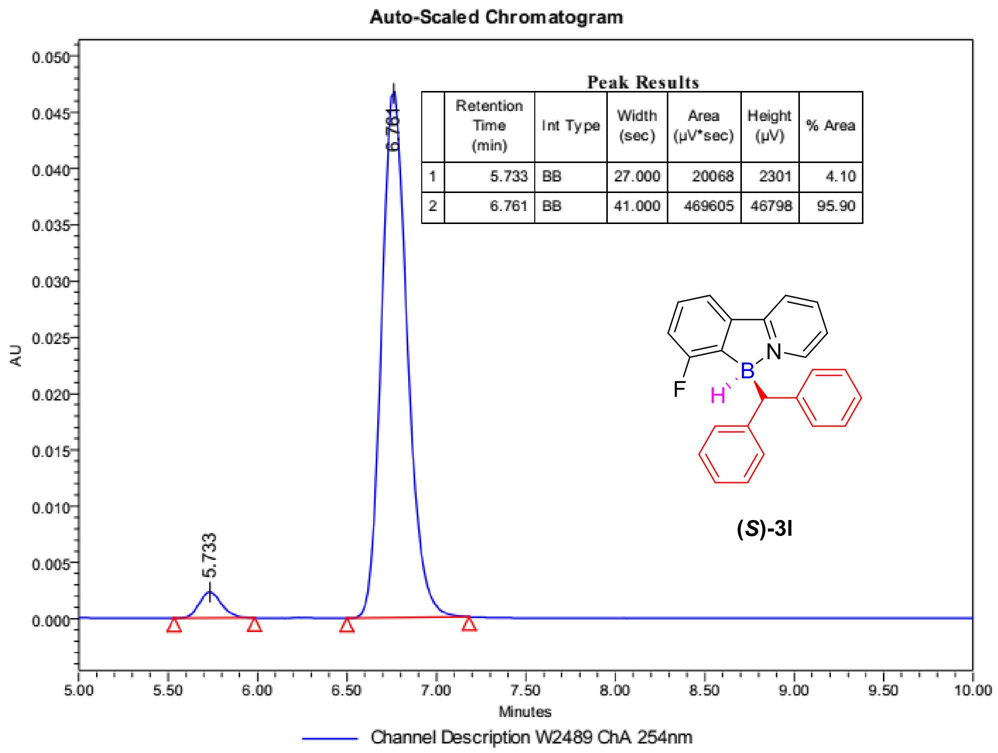

	Retention Time (min)	Int Type	Width (sec)	Area (µV*sec)	Height (µV)	% Area
1	5.733	BB	27.000	20068	2301	4.10
2	6.761	BB	41.000	469605	46798	95.90

Supplementary Figure 329. HPLC of compound (S)-3l

6-benzhydryl-7-bromo-6H-5λ[4]-benzo[3,4][1,2]azaborolo[1,5-a]pyridine (3m)

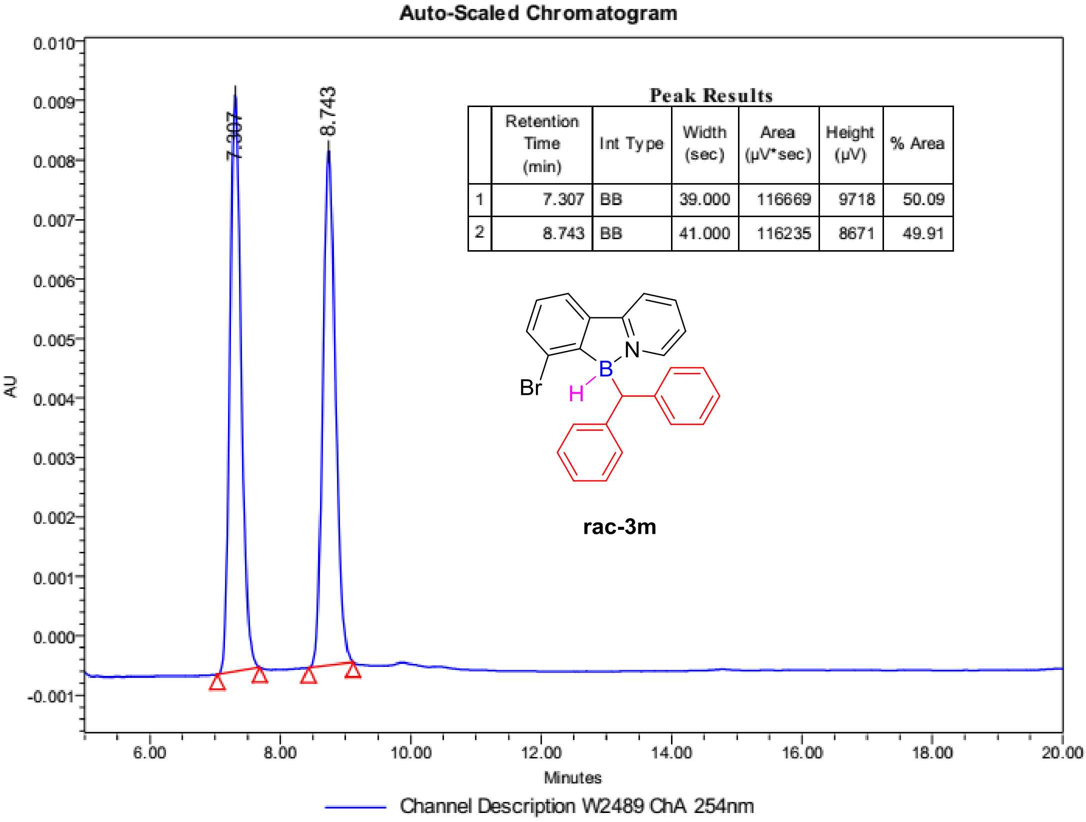

Peak Results

	Retention Time (min)	Int Type	Width (sec)	Area (μV*sec)	Height (μV)	% Area
1	7.307	BB	39.000	116669	9718	50.09
2	8.743	BB	41.000	116235	8671	49.91

Supplementary Figure 330. HPLC of compound rac-3m

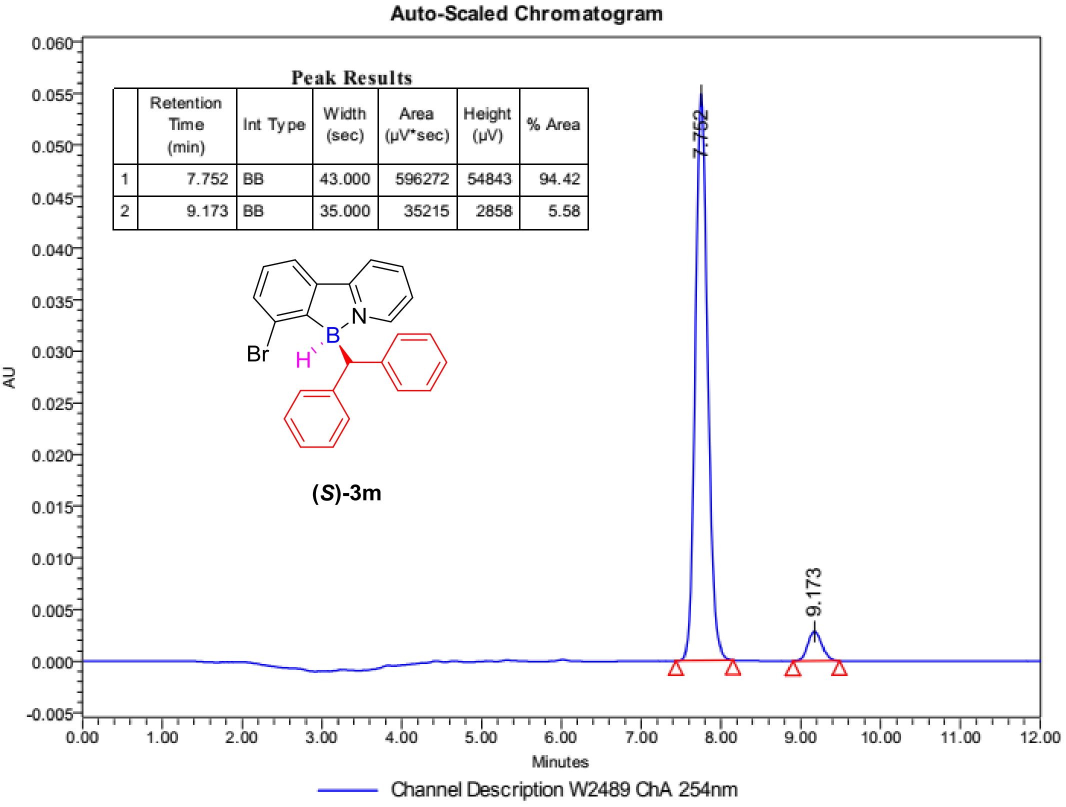

Peak Results

	Retention Time (min)	Int Type	Width (sec)	Area (μV*sec)	Height (μV)	% Area
1	7.752	BB	43.000	596272	54843	94.42
2	9.173	BB	35.000	35215	2858	5.58

Supplementary Figure 331. HPLC of compound (S)-3m

6-benzhydryl-7-chloro-10-fluoro-6H-5λ^4-benzo[3,4][1,2]azaborolo[1,5-a]pyridine (3n)

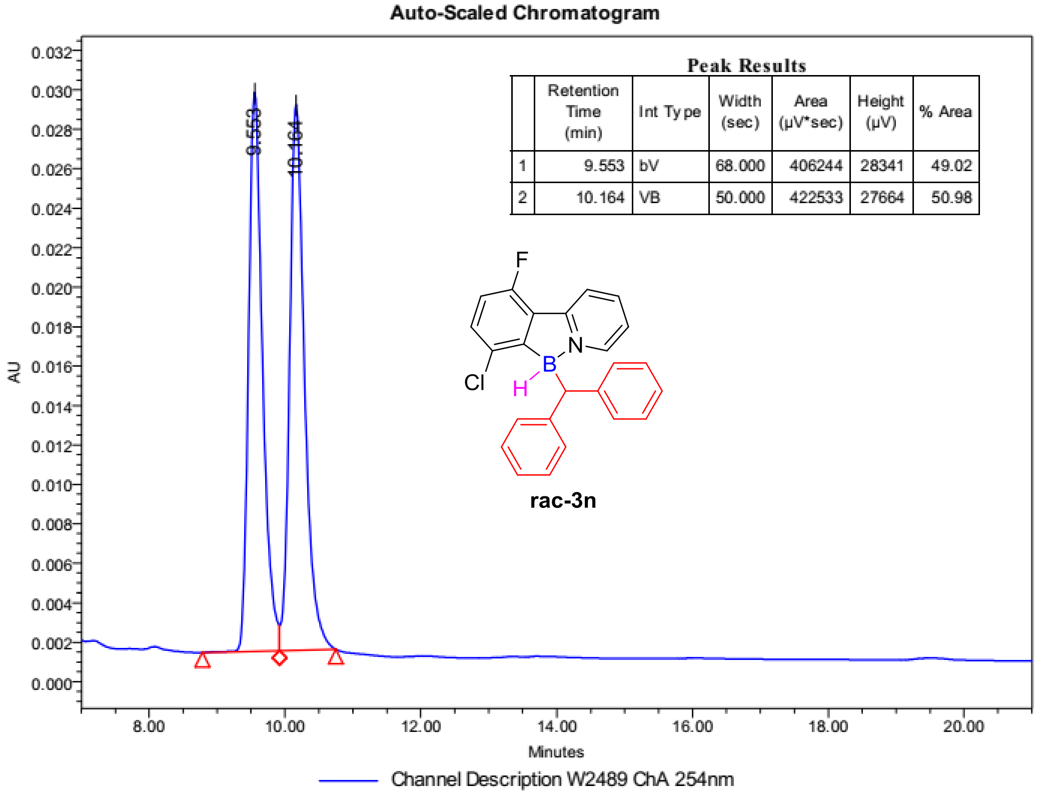

Peak Results

	Retention Time (min)	Int Type	Width (sec)	Area (µV*sec)	Height (µV)	% Area
1	9.553	bV	68.000	406244	28341	49.02
2	10.164	VB	50.000	422533	27664	50.98

Supplementary Figure 332. HPLC of compound rac-3n

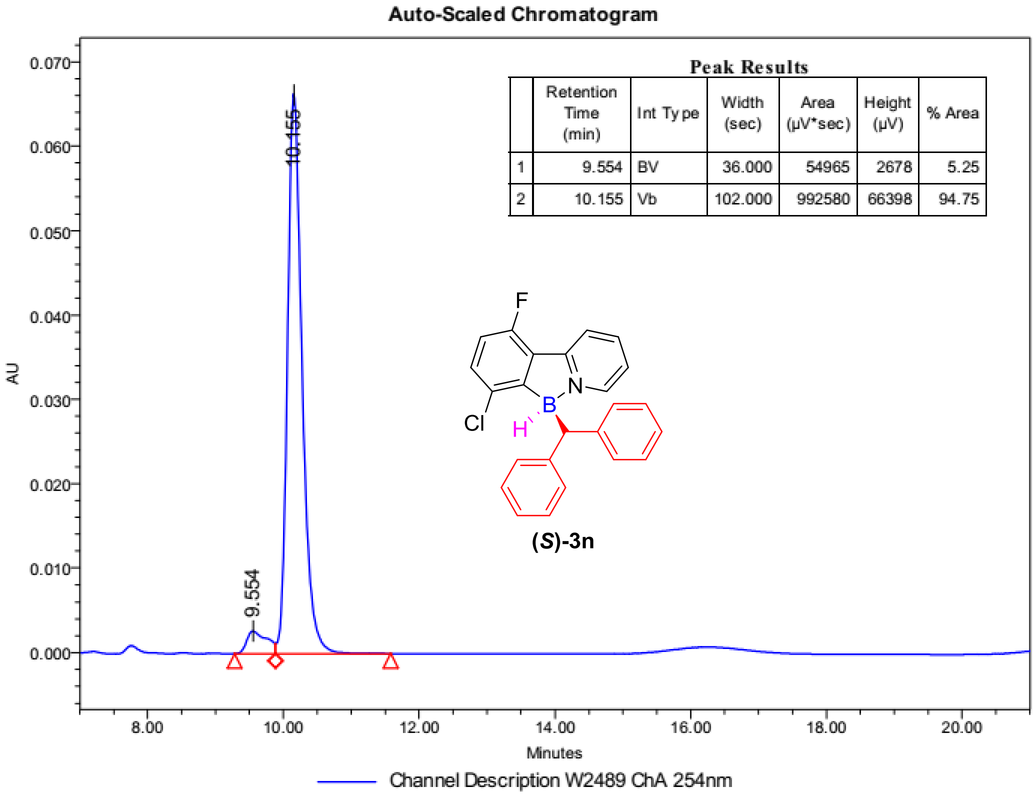

Peak Results

	Retention Time (min)	Int Type	Width (sec)	Area (µV*sec)	Height (µV)	% Area
1	9.554	BV	36.000	54965	2678	5.25
2	10.155	Vb	102.000	992580	66398	94.75

Supplementary Figure 333. HPLC of compound (S)-3n

6-benzhydryl-7-chloro-8-fluoro-6H-5λ[4]-benzo[3,4][1,2]azaborolo[1,5-a]pyridine (3o)

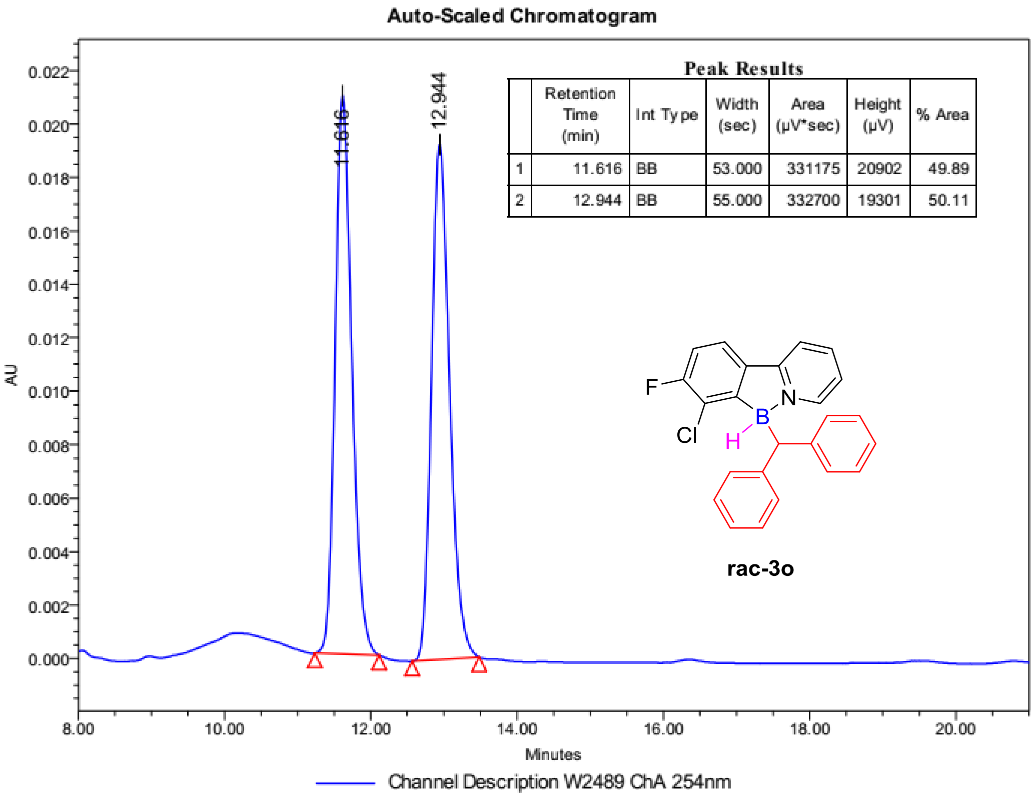

Peak Results

	Retention Time (min)	Int Type	Width (sec)	Area (μV*sec)	Height (μV)	% Area
1	11.616	BB	53.000	331175	20902	49.89
2	12.944	BB	55.000	332700	19301	50.11

Supplementary Figure 334. HPLC of compound rac-3o

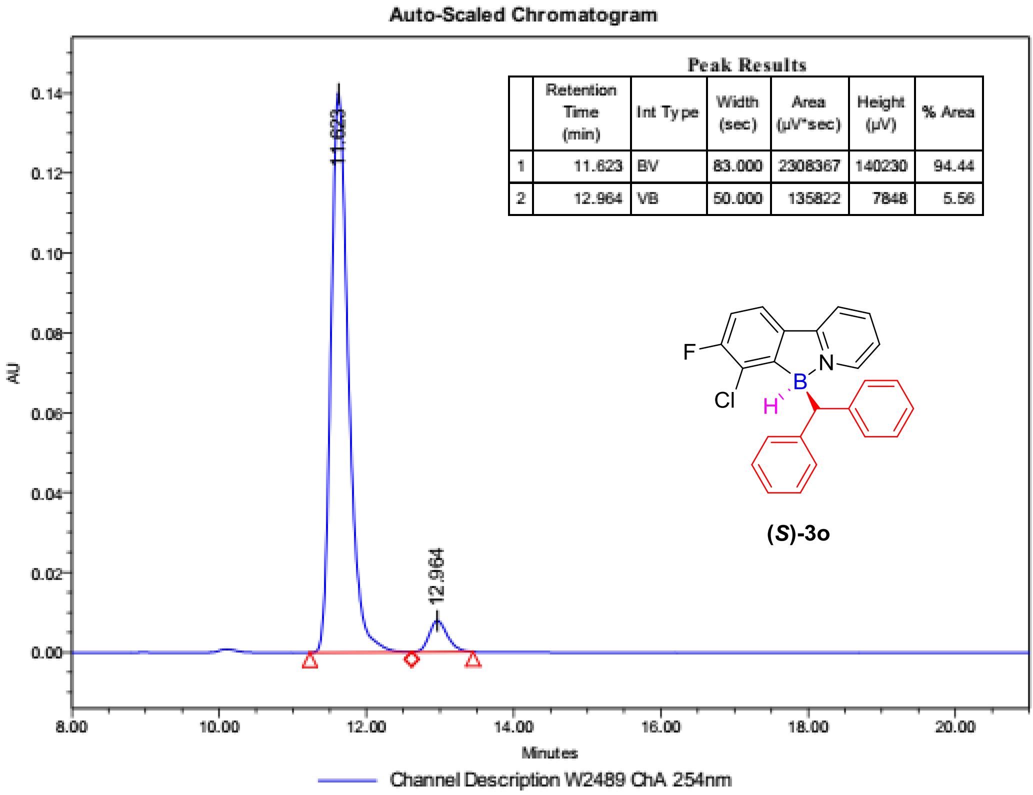

Peak Results

	Retention Time (min)	Int Type	Width (sec)	Area (μV*sec)	Height (μV)	% Area
1	11.623	BV	83.000	2308367	140230	94.44
2	12.964	VB	50.000	135822	7848	5.56

Supplementary Figure 335. HPLC of compound (S)-3o

6-benzhydryl-7,8-dichloro-6H-5λ^{4}-benzo[3,4][1,2]azaborolo[1,5-a]pyridine (3p)

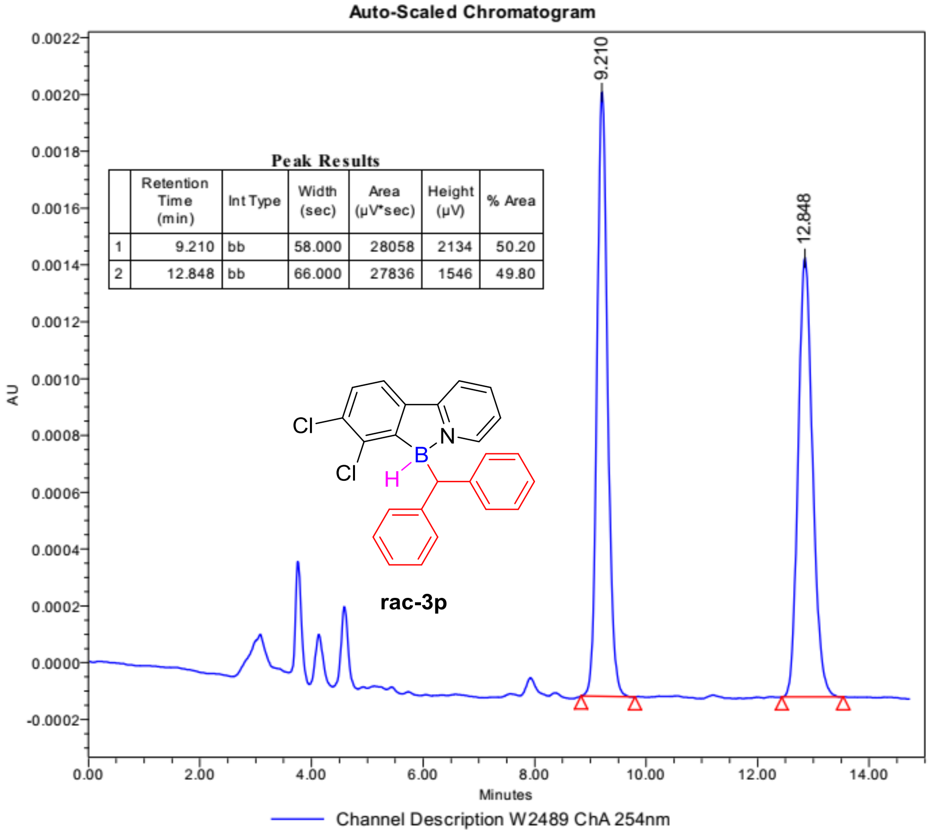

	Retention Time (min)	Int Type	Width (sec)	Area (μV*sec)	Height (μV)	% Area
1	9.210	bb	58.000	28058	2134	50.20
2	12.848	bb	66.000	27836	1546	49.80

Supplementary Figure 336. HPLC of compound rac-3p

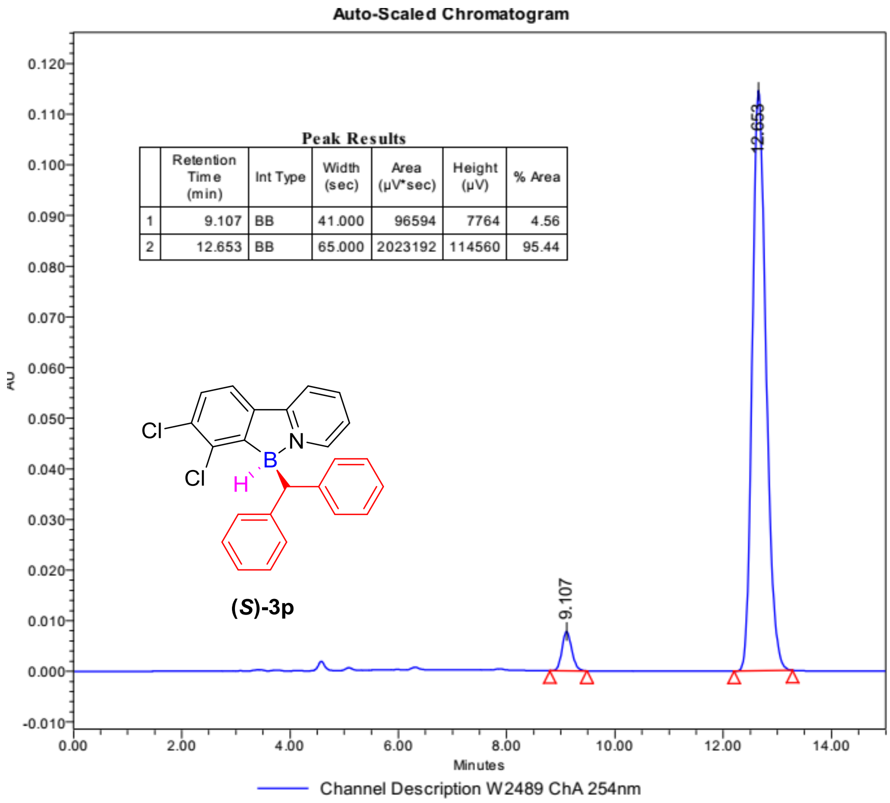

	Retention Time (min)	Int Type	Width (sec)	Area (μV*sec)	Height (μV)	% Area
1	9.107	BB	41.000	96594	7764	4.56
2	12.653	BB	65.000	2023192	114560	95.44

Supplementary Figure 337. HPLC of compound (S)-3p

6-benzhydryl-7,9-dichloro-6H-5λ^{4}-benzo[3,4][1,2]azaborolo[1,5-a]pyridine (3q)

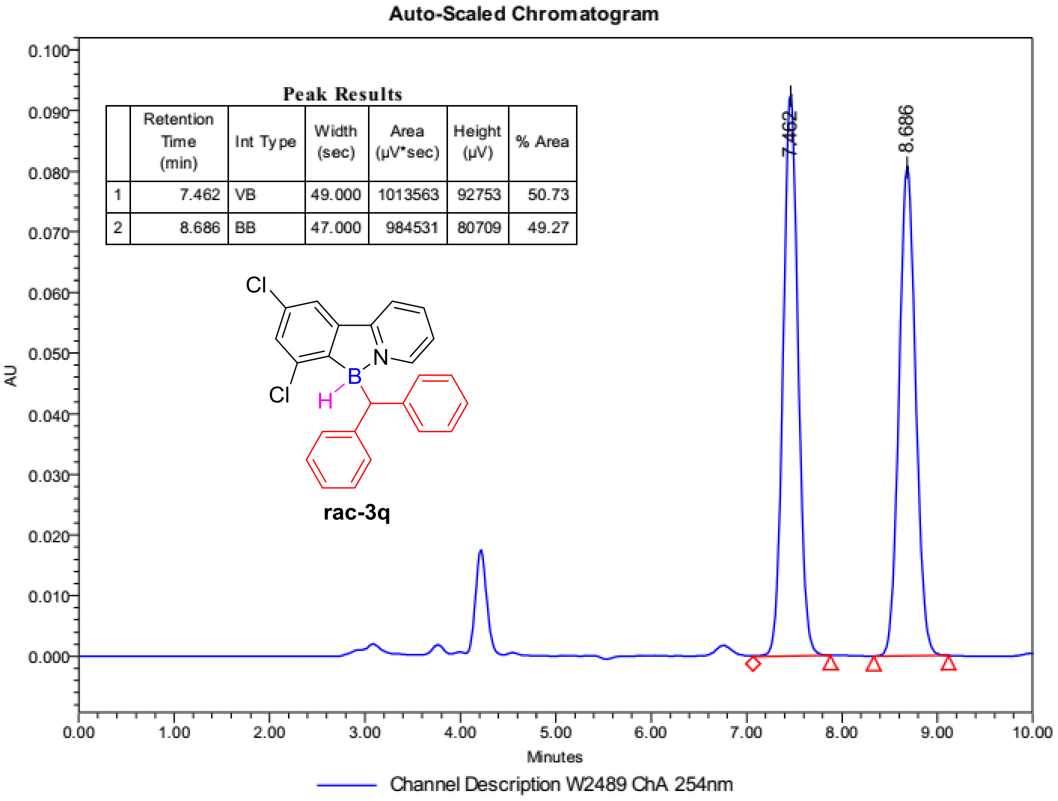

	Retention Time (min)	Int Type	Width (sec)	Area (μV*sec)	Height (μV)	% Area
1	7.462	VB	49.000	1013563	92753	50.73
2	8.686	BB	47.000	984531	80709	49.27

Supplementary Figure 338. HPLC of compound rac-3q

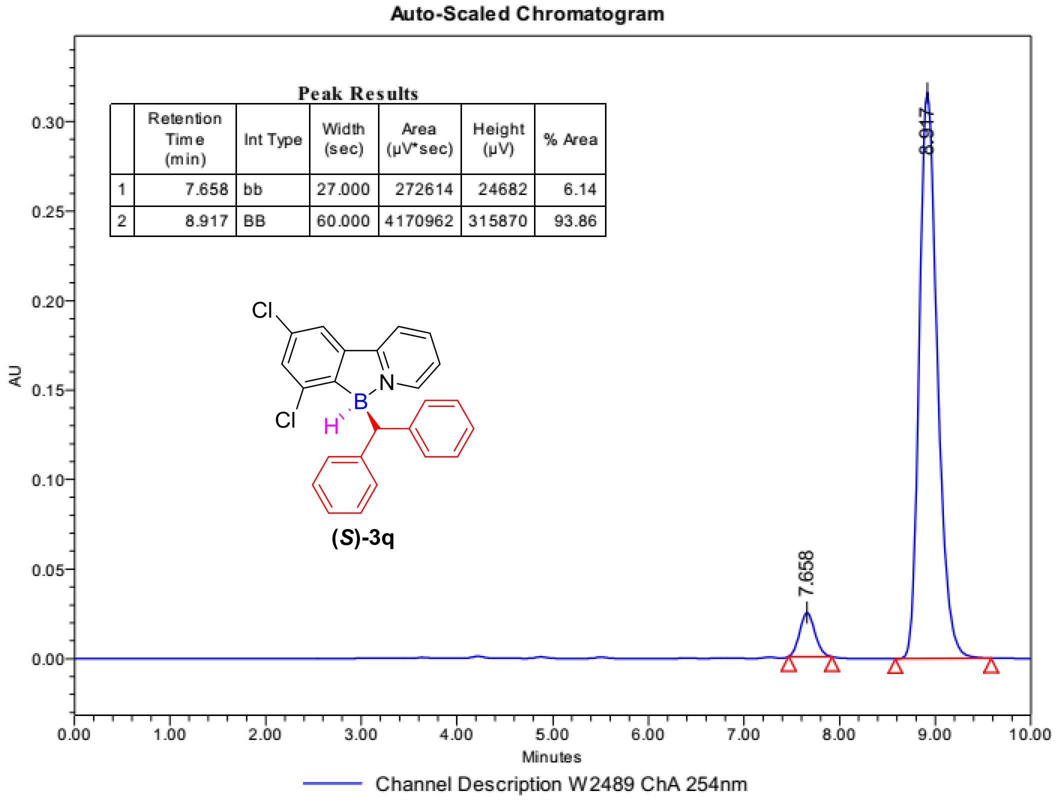

	Retention Time (min)	Int Type	Width (sec)	Area (μV*sec)	Height (μV)	% Area
1	7.658	bb	27.000	272614	24682	6.14
2	8.917	BB	60.000	4170962	315870	93.86

Supplementary Figure 339. HPLC of compound (S)-3q

6-benzhydryl-7-chloro-10-methyl-6H-5λ^4-benzo[3,4][1,2]azaborolo[1,5-a]pyridine (3r)

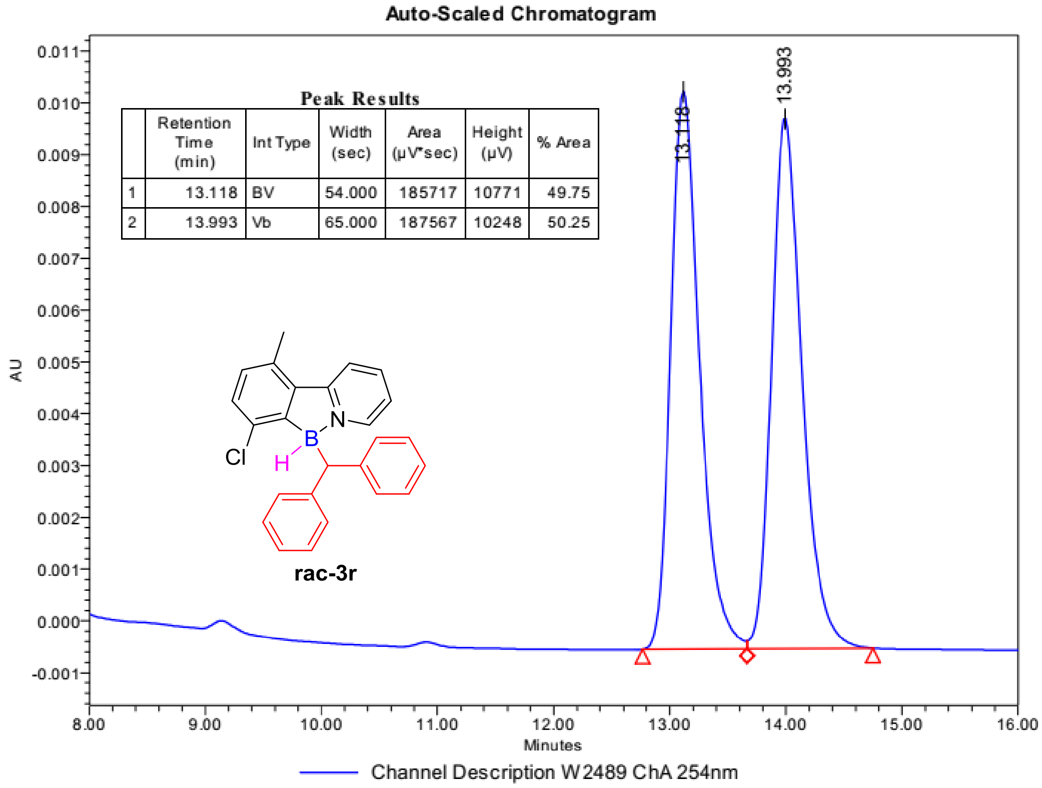

	Retention Time (min)	Int Type	Width (sec)	Area (µV*sec)	Height (µV)	% Area
1	13.118	BV	54.000	185717	10771	49.75
2	13.993	Vb	65.000	187567	10248	50.25

Supplementary Figure 340. HPLC of compound rac-3r

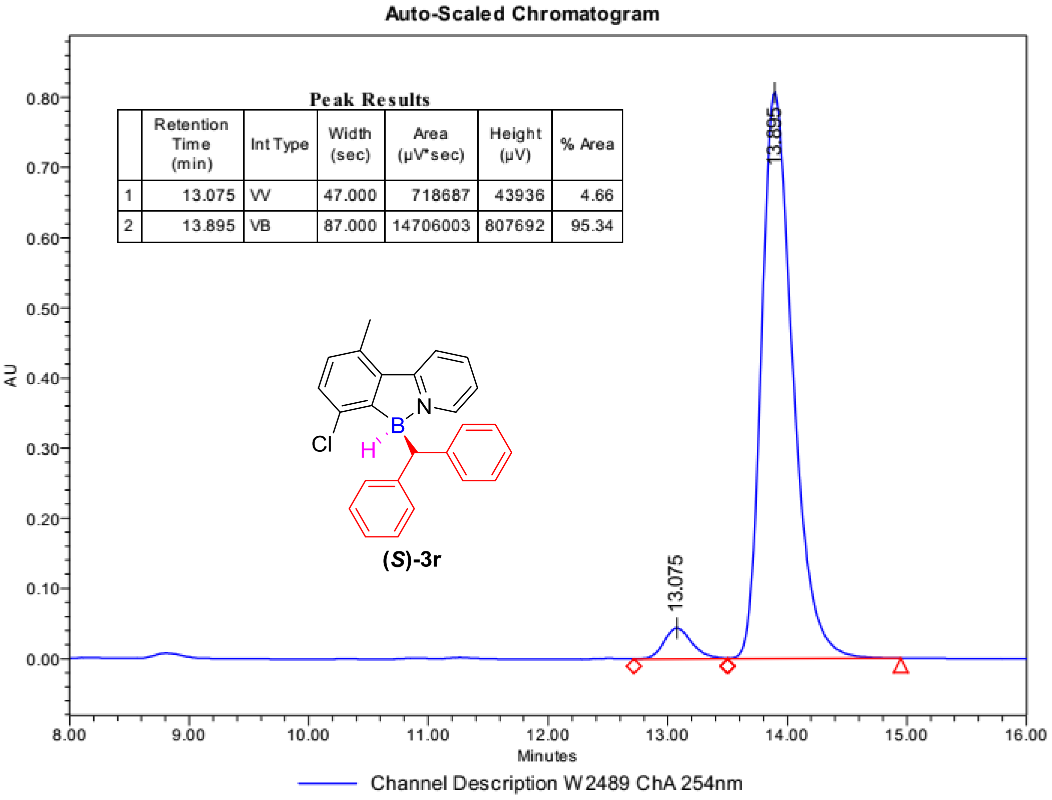

	Retention Time (min)	Int Type	Width (sec)	Area (µV*sec)	Height (µV)	% Area
1	13.075	VV	47.000	718687	43936	4.66
2	13.895	VB	87.000	14706003	807692	95.34

Supplementary Figure 341. HPLC of compound (S)-3r

12-benzhydryl-12H-11λ[4]-naphtho[1',2':3,4][1,2]azaborolo[1,5-a]pyridine (3s)

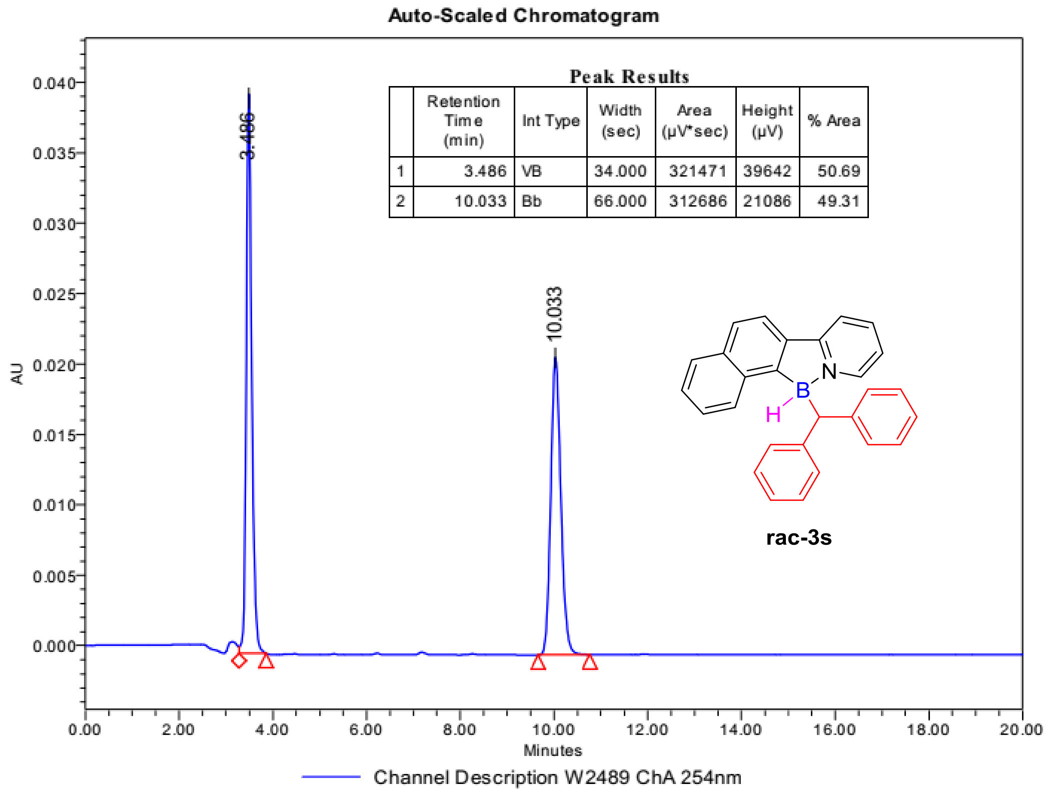

	Retention Time (min)	Int Type	Width (sec)	Area (μV*sec)	Height (μV)	% Area
1	3.486	VB	34.000	321471	39642	50.69
2	10.033	Bb	66.000	312686	21086	49.31

Supplementary Figure 342. HPLC of compound rac-3s

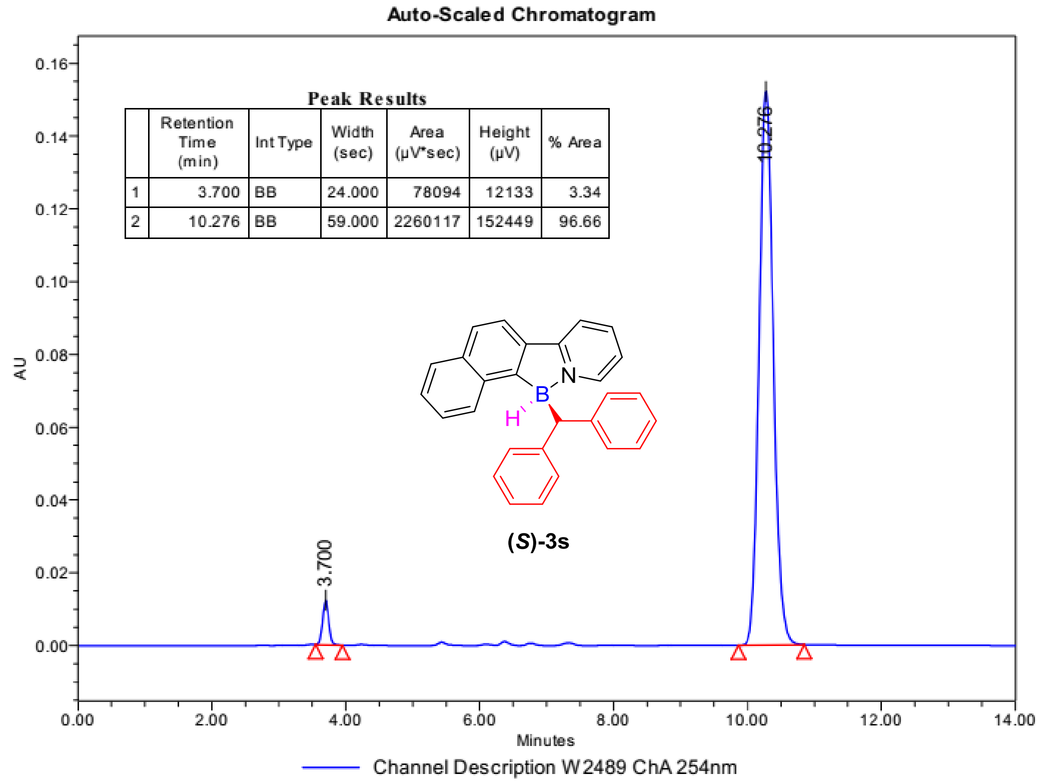

	Retention Time (min)	Int Type	Width (sec)	Area (μV*sec)	Height (μV)	% Area
1	3.700	BB	24.000	78094	12133	3.34
2	10.276	BB	59.000	2260117	152449	96.66

Supplementary Figure 343. HPLC of compound (S)-3s

ethyl (*S*)-2-(7-chloro-(*R*)6H-5λ[4]-benzo[3,4][1,2]azaborolo[1,5-a]pyridin-6-yl)-2-phenylacetate (5a)

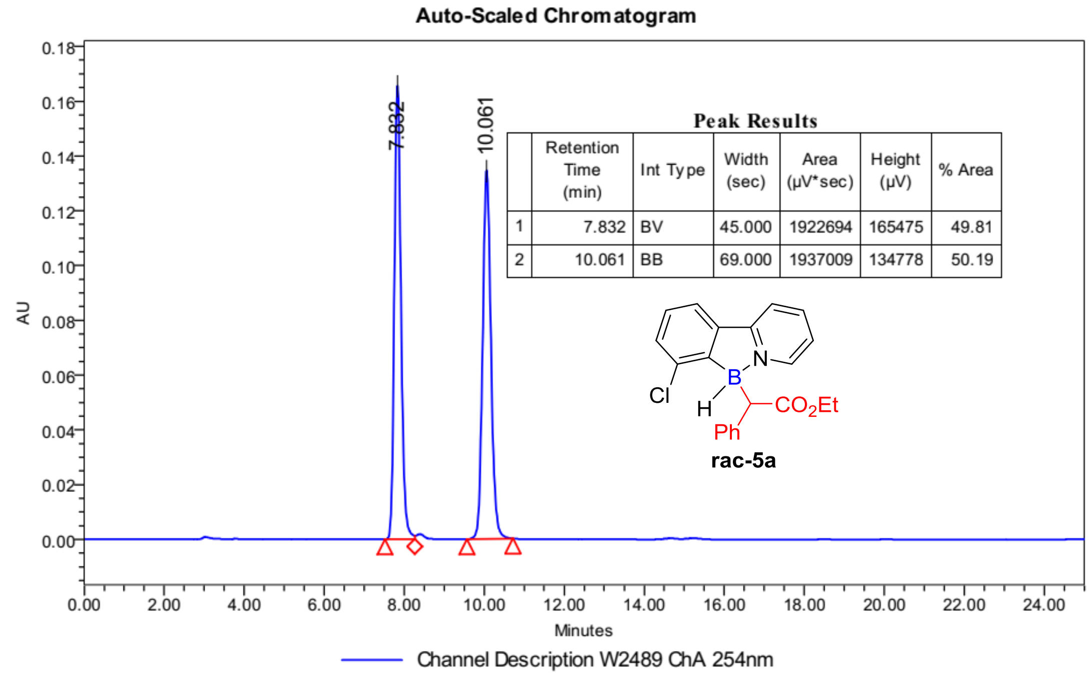

	Retention Time (min)	Int Type	Width (sec)	Area (µV*sec)	Height (µV)	% Area
1	7.832	BV	45.000	1922694	165475	49.81
2	10.061	BB	69.000	1937009	134778	50.19

Supplementary Figure 344. HPLC of compound rac-5a

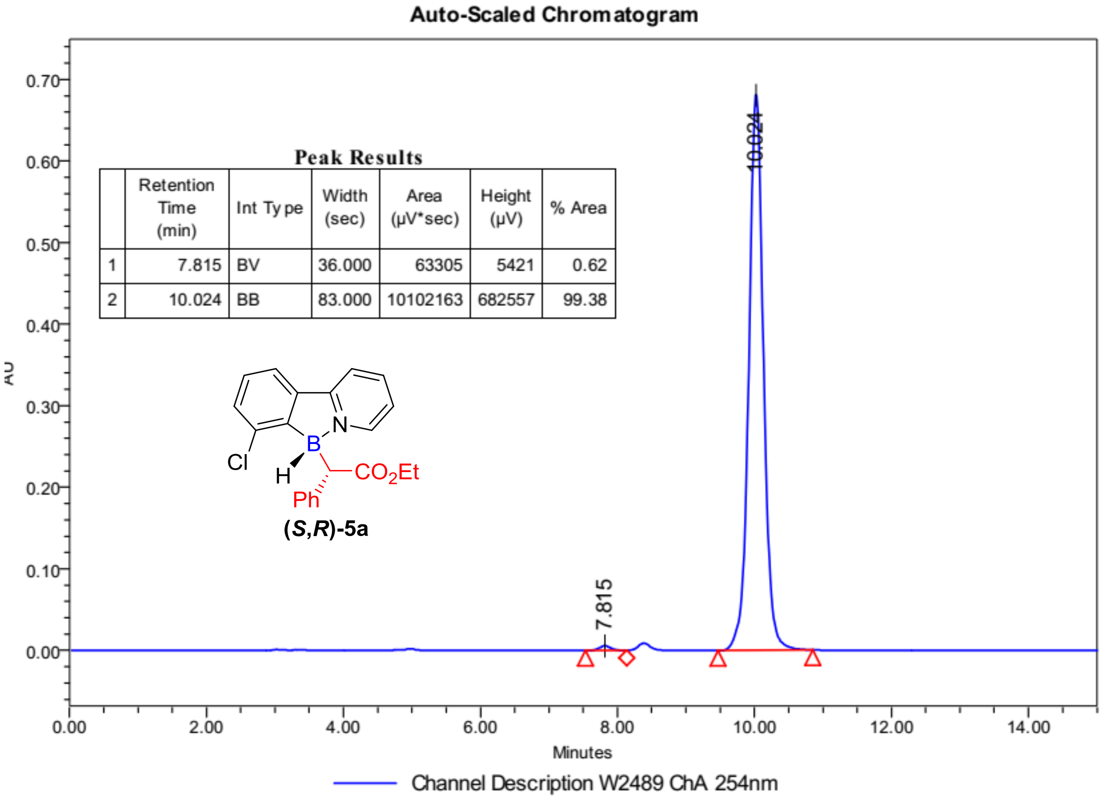

	Retention Time (min)	Int Type	Width (sec)	Area (µV*sec)	Height (µV)	% Area
1	7.815	BV	36.000	63305	5421	0.62
2	10.024	BB	83.000	10102163	682557	99.38

Supplementary Figure 345. HPLC of compound (S,R)-5a

ethyl (S)-2-(7-fluoro-6H-5λ[4]-benzo[3,4][1,2]azaborolo[1,5-a]pyridin-6-yl)-2-phenylacetate (5b)

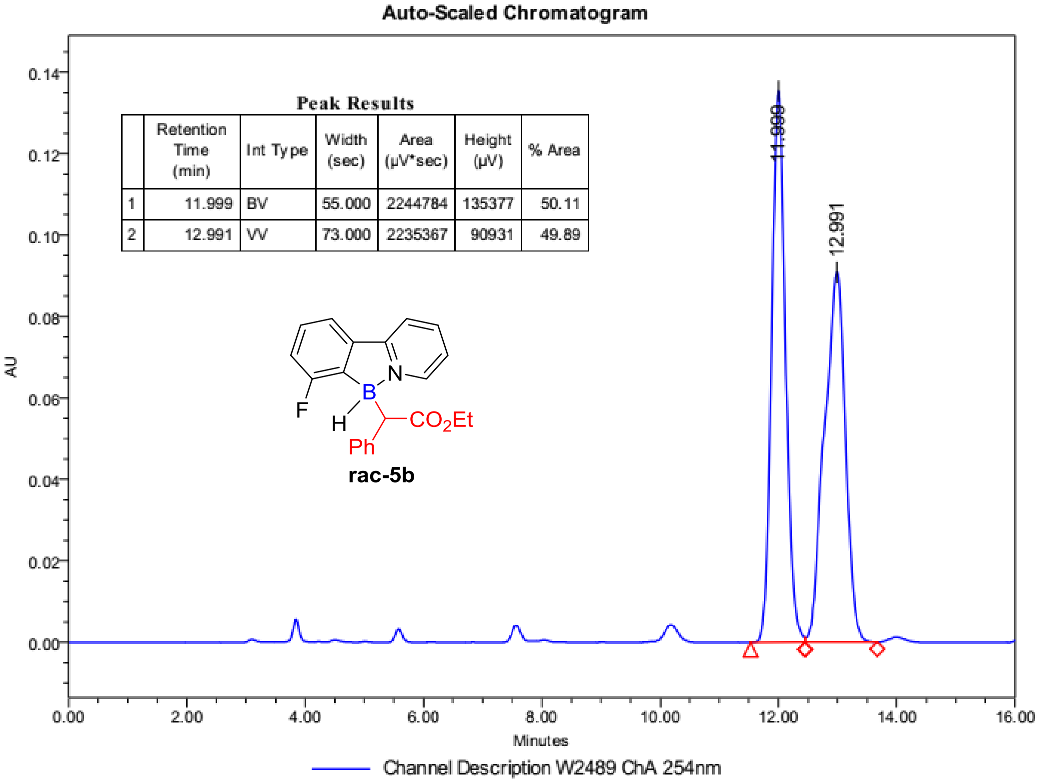

	Retention Time (min)	Int Type	Width (sec)	Area (µV*sec)	Height (µV)	% Area
1	11.999	BV	55.000	2244784	135377	50.11
2	12.991	VV	73.000	2235367	90931	49.89

Supplementary Figure 346. HPLC of compound rac-5b

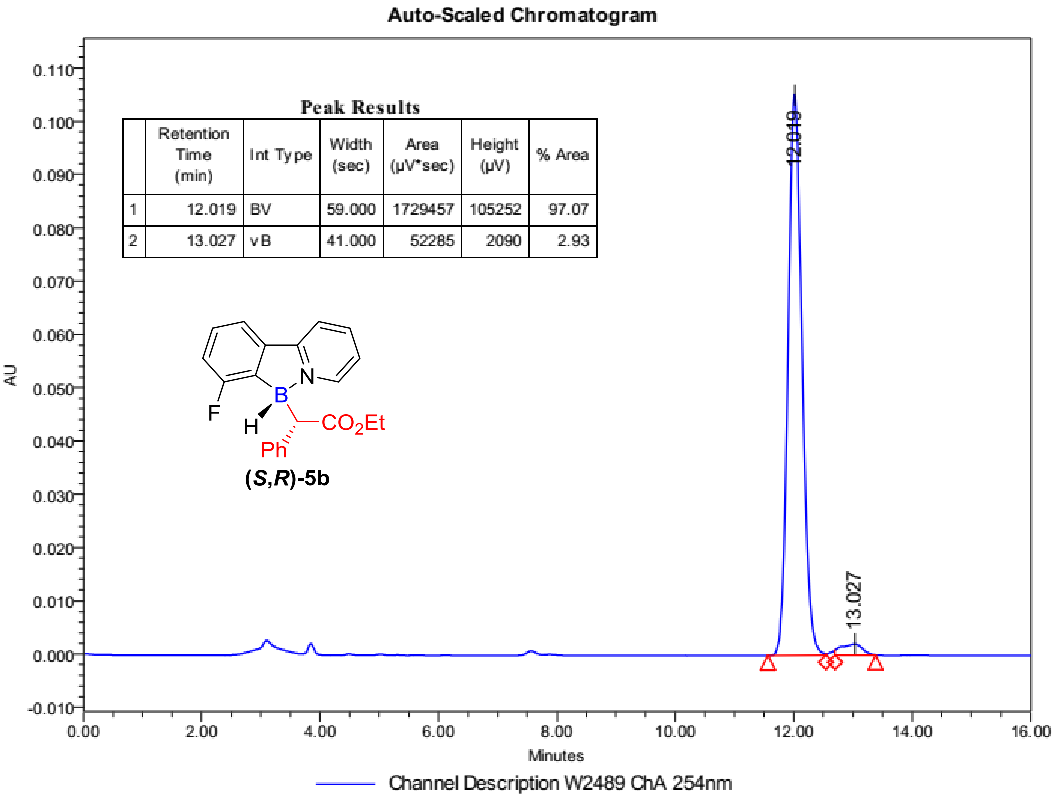

	Retention Time (min)	Int Type	Width (sec)	Area (µV*sec)	Height (µV)	% Area
1	12.019	BV	59.000	1729457	105252	97.07
2	13.027	vB	41.000	52285	2090	2.93

Supplementary Figure 347. HPLC of compound (S,R)-5b

ethyl(*S*)-2-(8-fluoro-(*R*)6H-5λ[4]-benzo[3,4][1,2]azaborolo[1,5-a]pyridin-6-yl)-2-phenylacetate (5c)

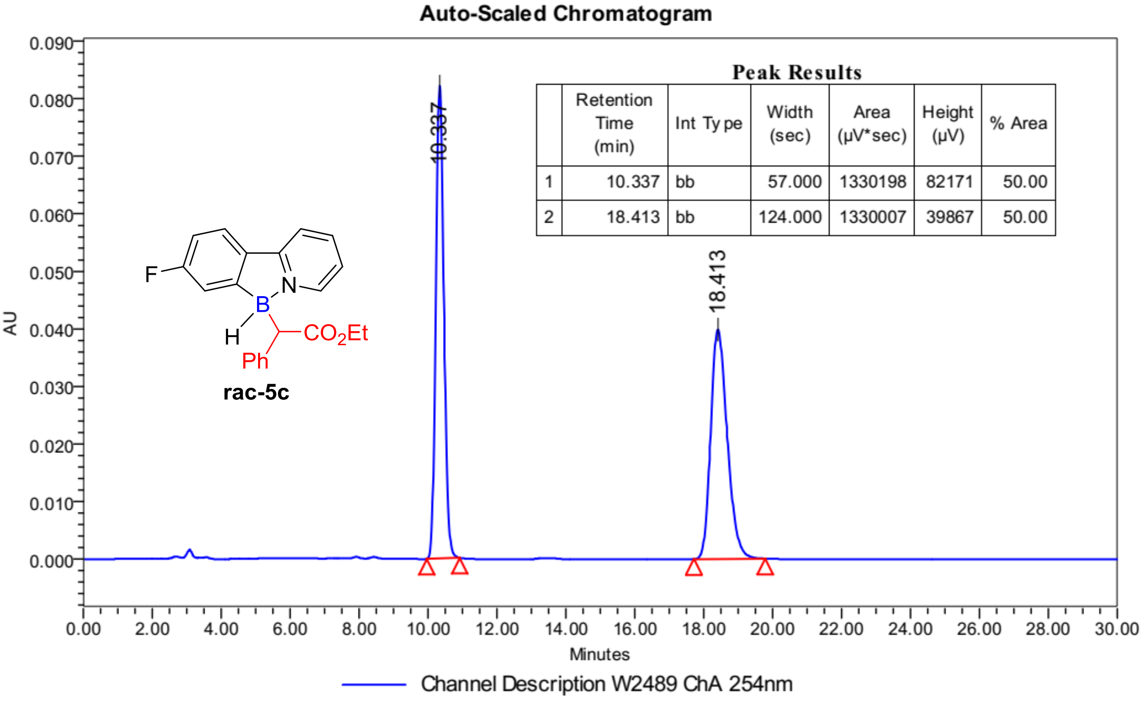

Peak Results

	Retention Time (min)	Int Type	Width (sec)	Area (µV*sec)	Height (µV)	% Area
1	10.337	bb	57.000	1330198	82171	50.00
2	18.413	bb	124.000	1330007	39867	50.00

Supplementary Figure 348. HPLC of compound rac-5c

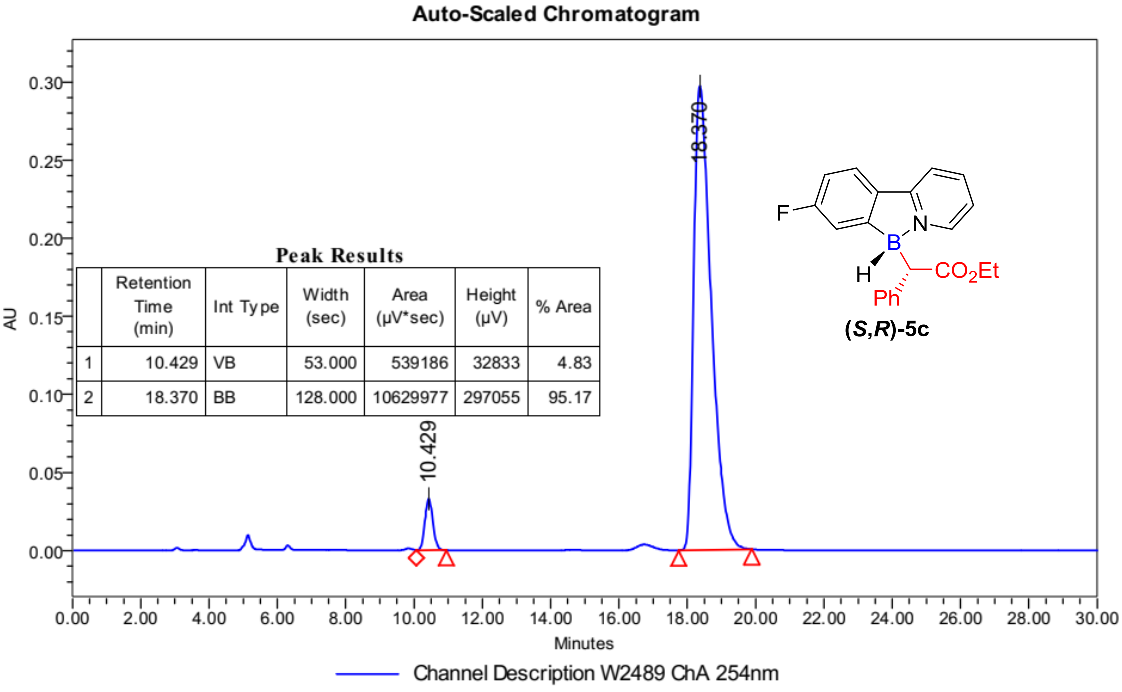

Peak Results

	Retention Time (min)	Int Type	Width (sec)	Area (µV*sec)	Height (µV)	% Area
1	10.429	VB	53.000	539186	32833	4.83
2	18.370	BB	128.000	10629977	297055	95.17

Supplementary Figure 349. HPLC of compound (S,R)-5c

ethyl (S)-2-(7-bromo-6H-5λ[4]-benzo[3,4][1,2]azaborolo[1,5-a]pyridin-6-yl)-2-phenylacetate (5d)

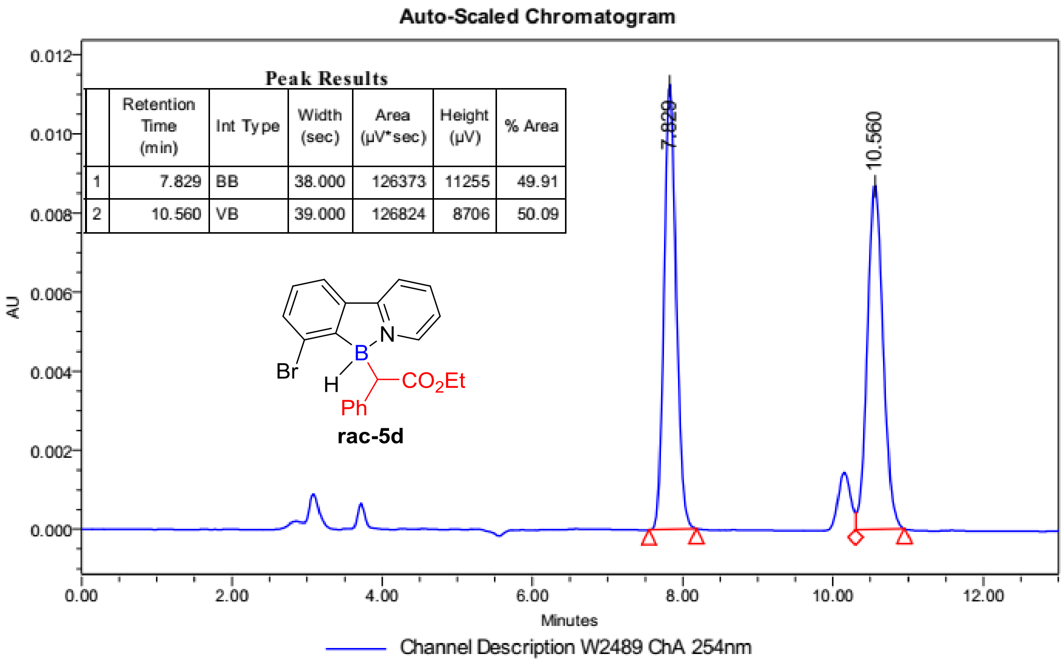

	Retention Time (min)	Int Type	Width (sec)	Area (µV*sec)	Height (µV)	% Area
1	7.829	BB	38.000	126373	11255	49.91
2	10.560	VB	39.000	126824	8706	50.09

Supplementary Figure 350. HPLC of compound rac-5d

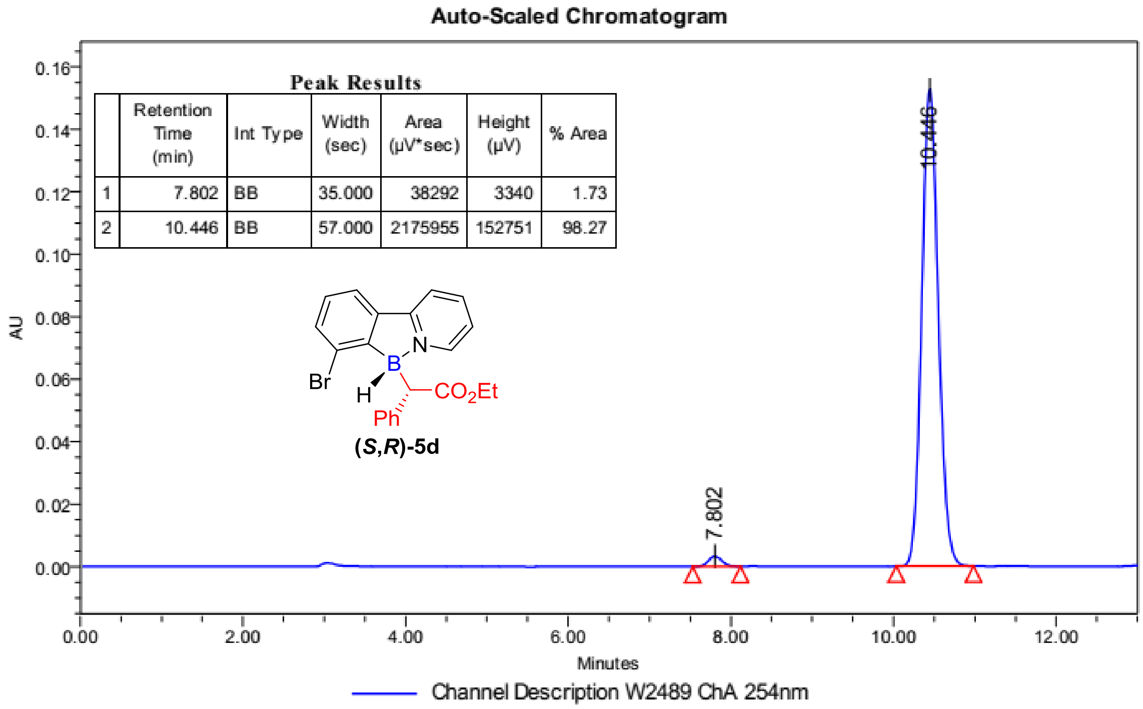

	Retention Time (min)	Int Type	Width (sec)	Area (µV*sec)	Height (µV)	% Area
1	7.802	BB	35.000	38292	3340	1.73
2	10.446	BB	57.000	2175955	152751	98.27

Supplementary Figure 351. HPLC of compound (S,R)-5d

ethyl(S)-2-(7-chloro-8-fluoro-6H-5l4-benzo[3,4][1,2]azaborolo[1,5-a]pyridin-6-yl)-2-phenylacetate (5e)

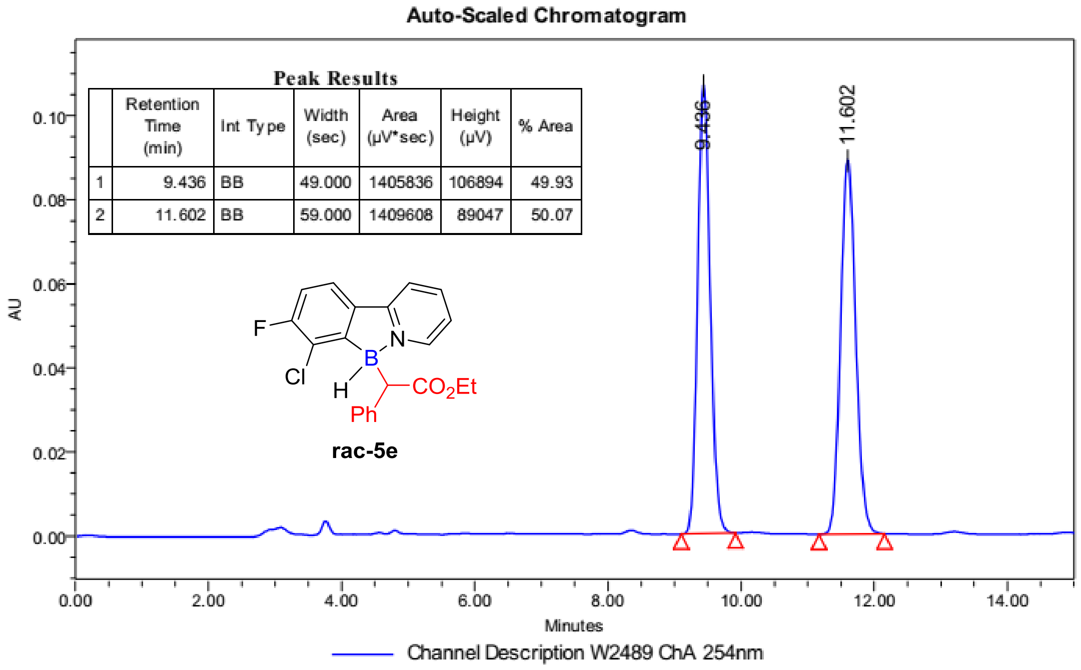

	Retention Time (min)	Int Type	Width (sec)	Area (μV*sec)	Height (μV)	% Area
1	9.436	BB	49.000	1405836	106894	49.93
2	11.602	BB	59.000	1409608	89047	50.07

Supplementary Figure 352. HPLC of compound rac-5e

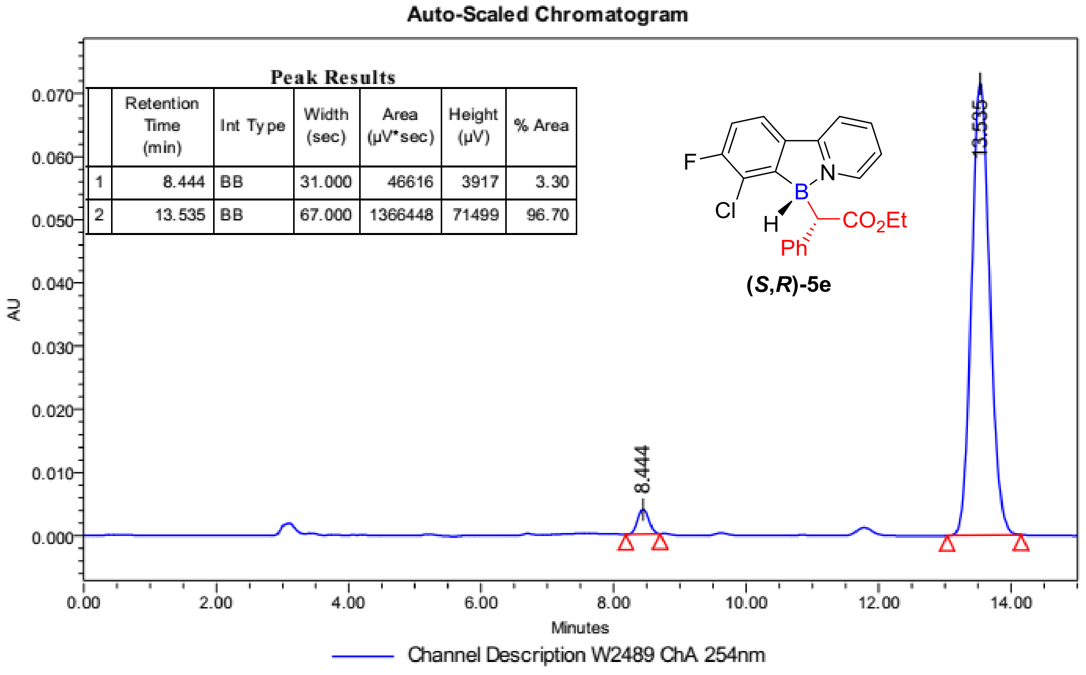

	Retention Time (min)	Int Type	Width (sec)	Area (μV*sec)	Height (μV)	% Area
1	8.444	BB	31.000	46616	3917	3.30
2	13.535	BB	67.000	1366448	71499	96.70

Supplementary Figure 353. HPLC of compound (S,R)-5e

ethyl (S)-2-(7,8-dichloro-6H-5λ[4]-benzo[3,4][1,2]azaborolo[1,5-a]pyridin-6-yl)-2-phenylacetate (5f)

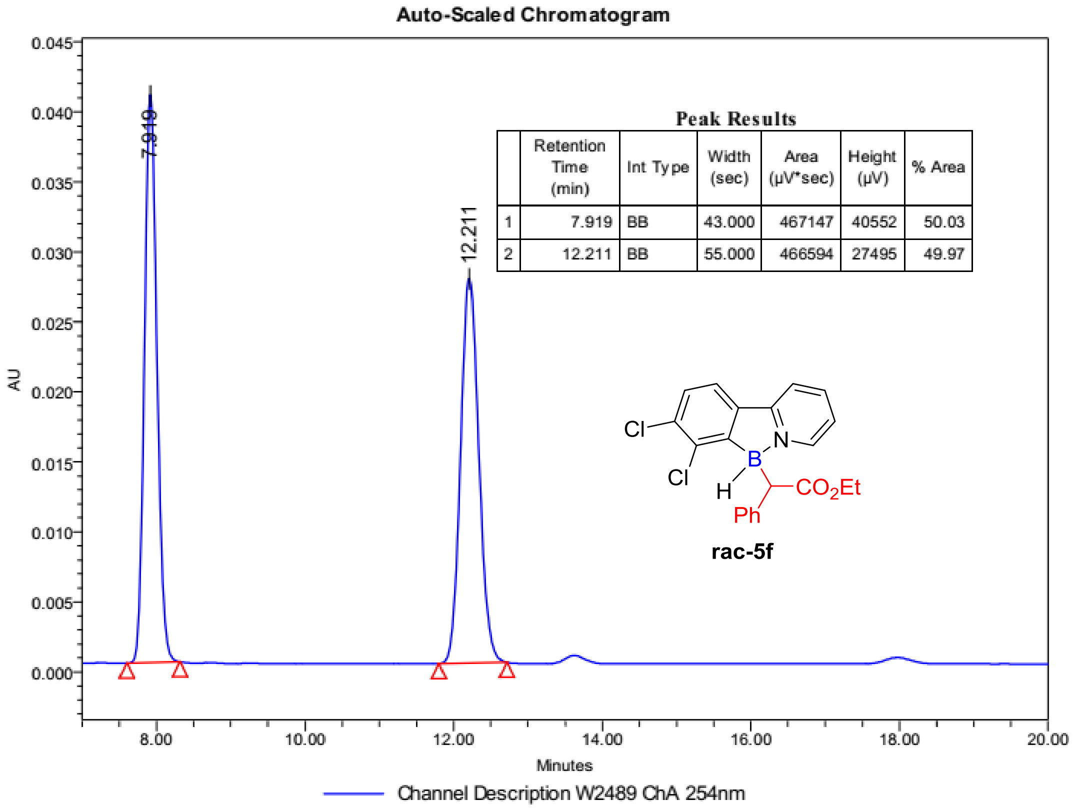

Peak Results

	Retention Time (min)	Int Type	Width (sec)	Area (µV*sec)	Height (µV)	% Area
1	7.919	BB	43.000	467147	40552	50.03
2	12.211	BB	55.000	466594	27495	49.97

Supplementary Figure 354. HPLC of compound rac-5f

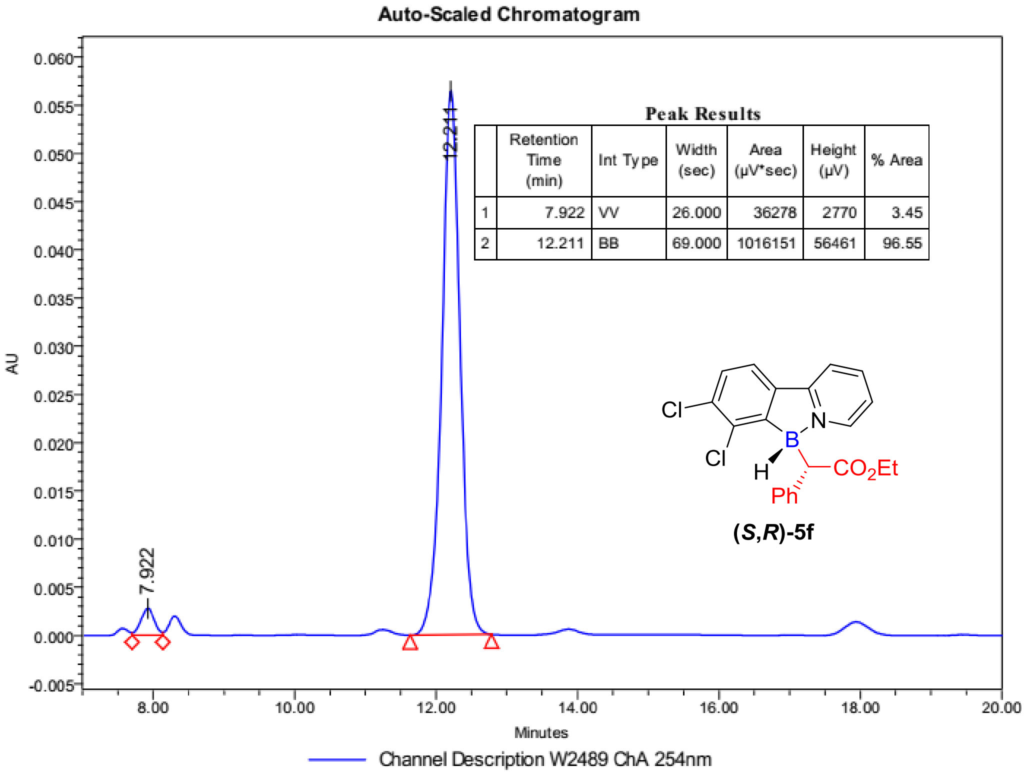

Peak Results

	Retention Time (min)	Int Type	Width (sec)	Area (µV*sec)	Height (µV)	% Area
1	7.922	VV	26.000	36278	2770	3.45
2	12.211	BB	69.000	1016151	56461	96.55

Supplementary Figure 355. HPLC of compound (S,R)-5f

ethyl(S)-2-(7-chloro-8-methyl-6H-5λ^4-benzo[3,4][1,2]azaborolo[1,5-a]pyridin-6-yl)-2-phenylacetat-e (5g)

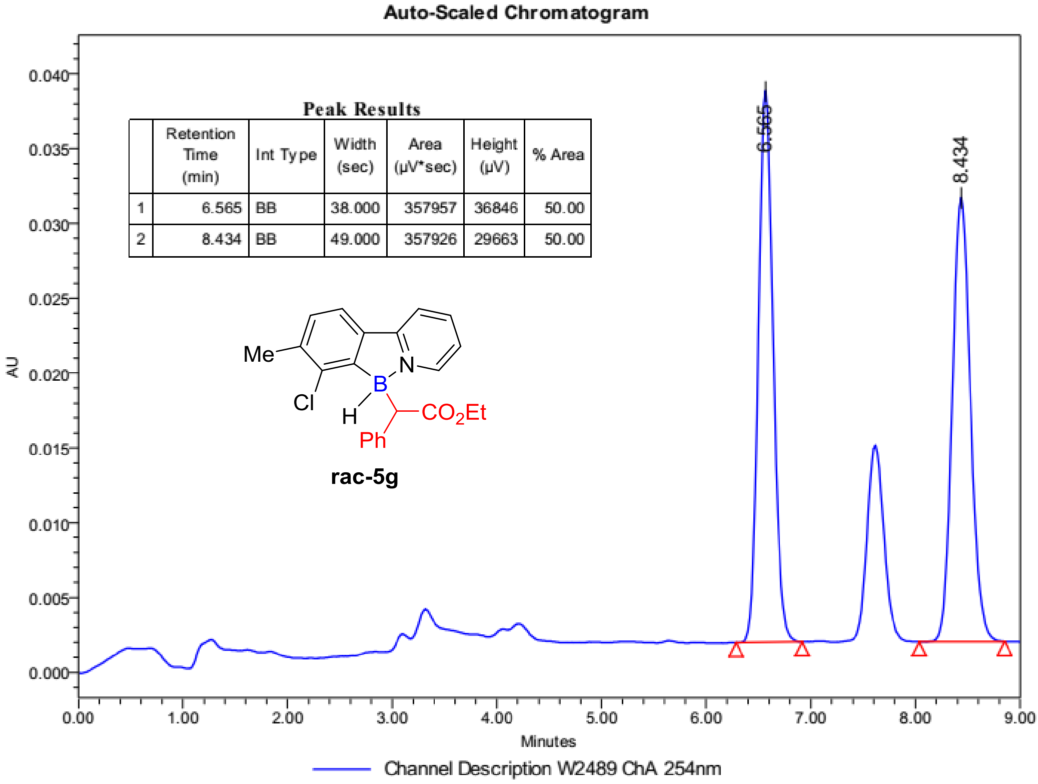

	Retention Time (min)	Int Type	Width (sec)	Area (µV*sec)	Height (µV)	% Area
1	6.565	BB	38.000	357957	36846	50.00
2	8.434	BB	49.000	357926	29663	50.00

Supplementary Figure 356. HPLC of compound rac-5g

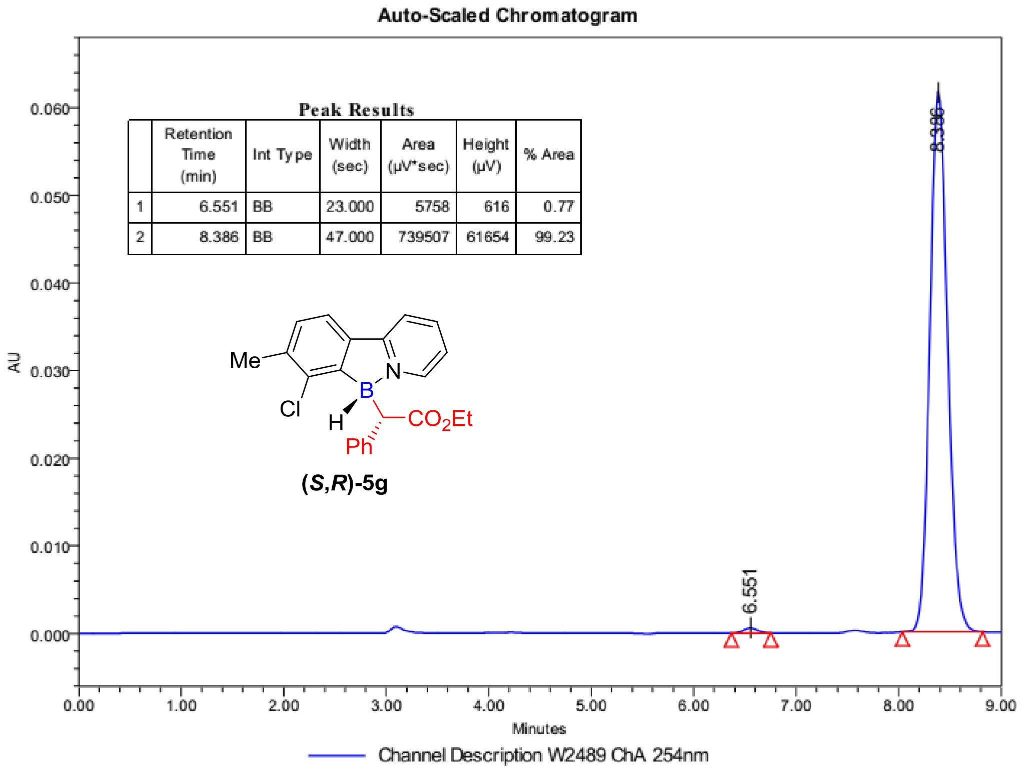

	Retention Time (min)	Int Type	Width (sec)	Area (µV*sec)	Height (µV)	% Area
1	6.551	BB	23.000	5758	616	0.77
2	8.386	BB	47.000	739507	61654	99.23

Supplementary Figure 357. HPLC of compound (S,R)-5g

ethyl(*S*)-2-(7,9-dichloro-(*R*)6H-5λ[4]-benzo[3,4][1,2]azaborolo[1,5-a]pyridin-6-yl)-2-phenylacetate (5h)

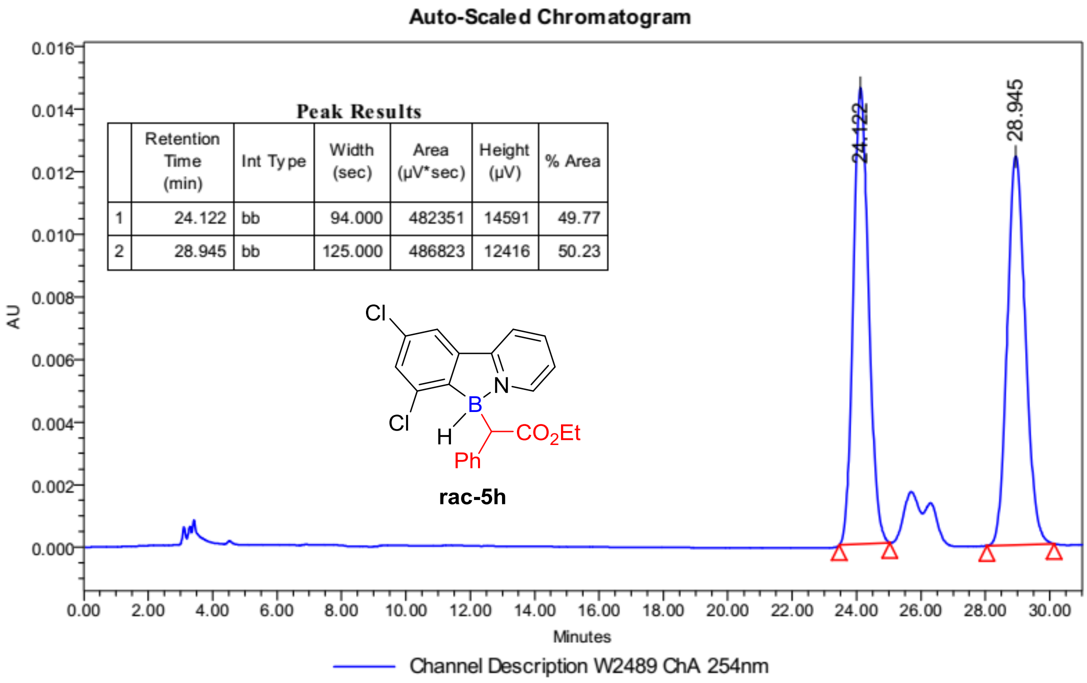

	Retention Time (min)	Int Type	Width (sec)	Area (μV*sec)	Height (μV)	% Area
1	24.122	bb	94.000	482351	14591	49.77
2	28.945	bb	125.000	486823	12416	50.23

Supplementary Figure 358. HPLC of compound rac-5h

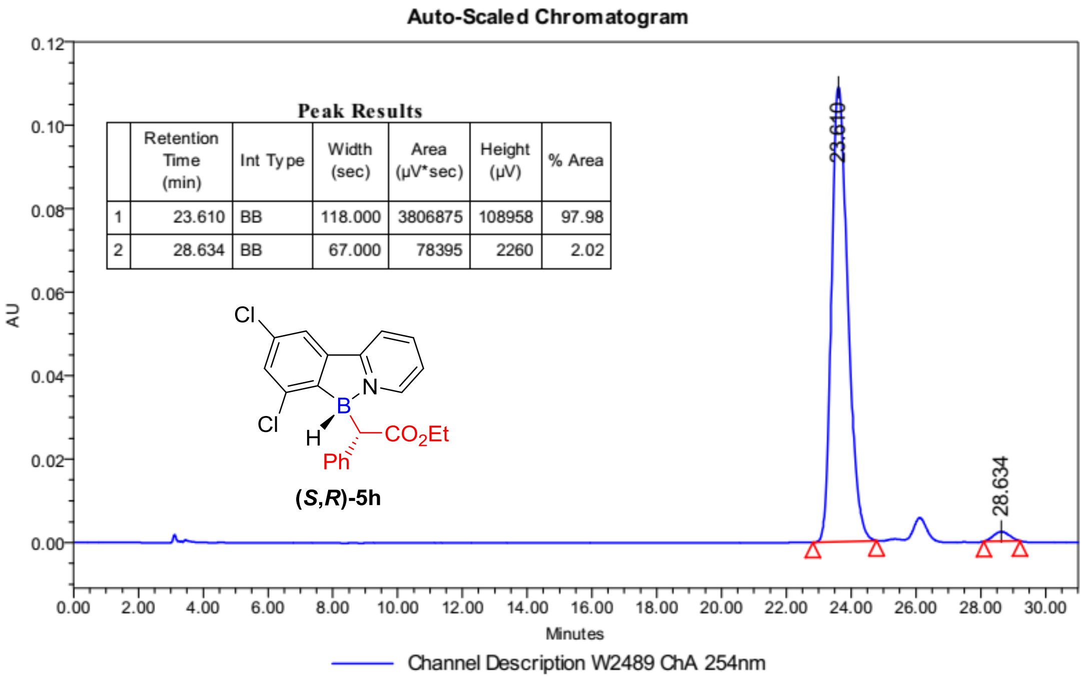

	Retention Time (min)	Int Type	Width (sec)	Area (μV*sec)	Height (μV)	% Area
1	23.610	BB	118.000	3806875	108958	97.98
2	28.634	BB	67.000	78395	2260	2.02

Supplementary Figure 359. HPLC of compound (S,R)-5h

ethyl (S)-2-(7,10-dichloro-6H-5λ[4]-benzo[3,4][1,2]azaborolo[1,5-a]pyridin-6-yl)-2-phenylacetate (5i)

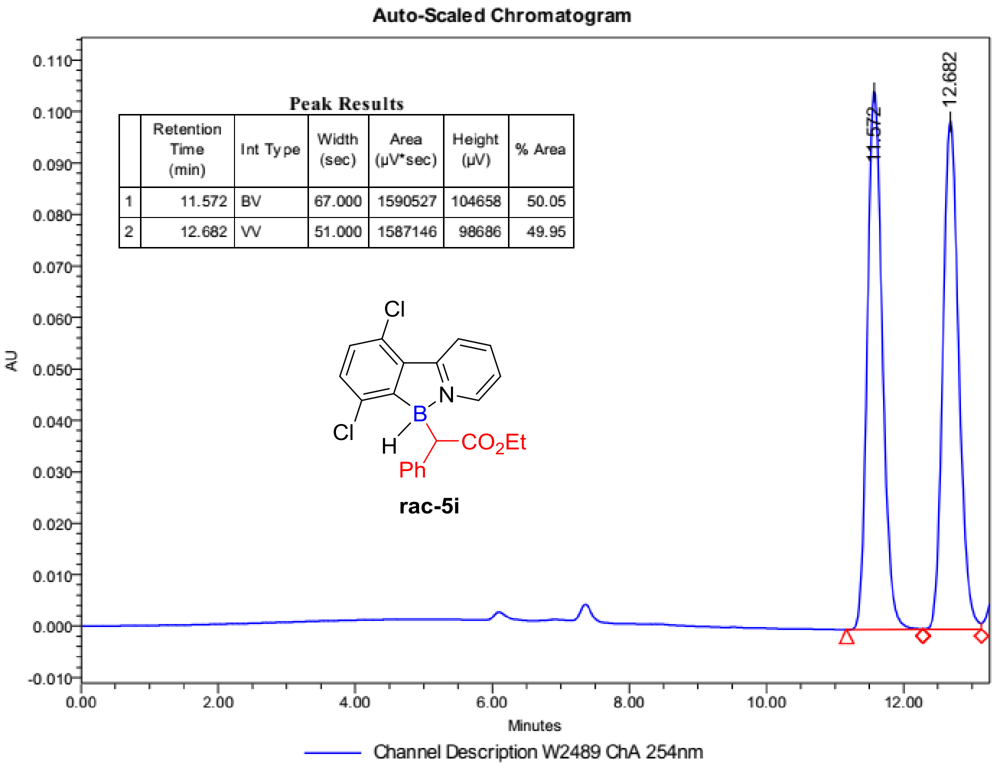

	Retention Time (min)	Int Type	Width (sec)	Area (μV*sec)	Height (μV)	% Area
1	11.572	BV	67.000	1590527	104658	50.05
2	12.682	VV	51.000	1587146	98686	49.95

Supplementary Figure 360. HPLC of compound rac-5i

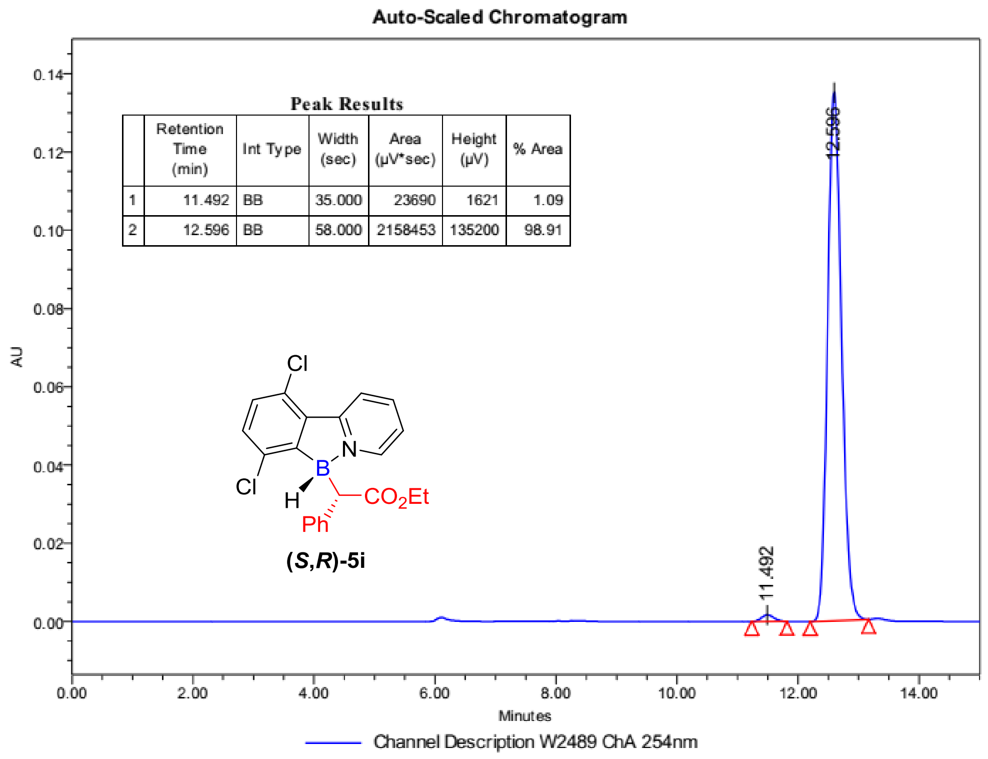

	Retention Time (min)	Int Type	Width (sec)	Area (μV*sec)	Height (μV)	% Area
1	11.492	BB	35.000	23690	1621	1.09
2	12.596	BB	58.000	2158453	135200	98.91

Supplementary Figure 361. HPLC of compound (S,R)-5i

ethyl(S)-2-(7-chloro-10-fluoro-6H-5l4-benzo[3,4][1,2]azaborolo[1,5-a]pyridin-6-yl)-2-phenylaceta-te (**5j**)

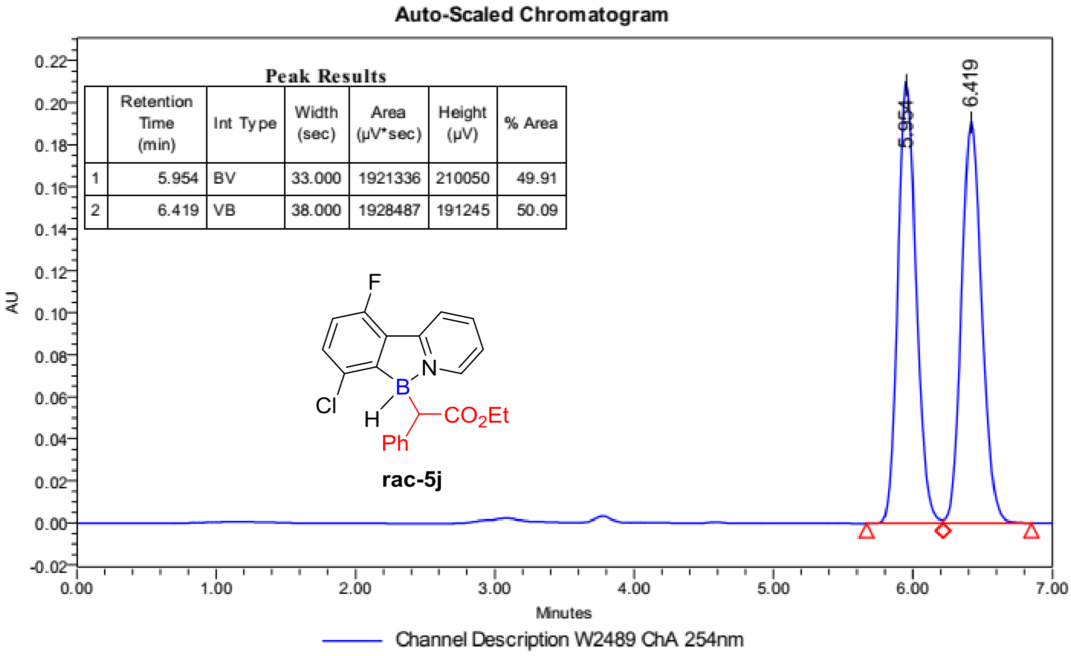

	Retention Time (min)	Int Type	Width (sec)	Area (µV*sec)	Height (µV)	% Area
1	5.954	BV	33.000	1921336	210050	49.91
2	6.419	VB	38.000	1928487	191245	50.09

Supplementary Figure 362. HPLC of compound rac-5j

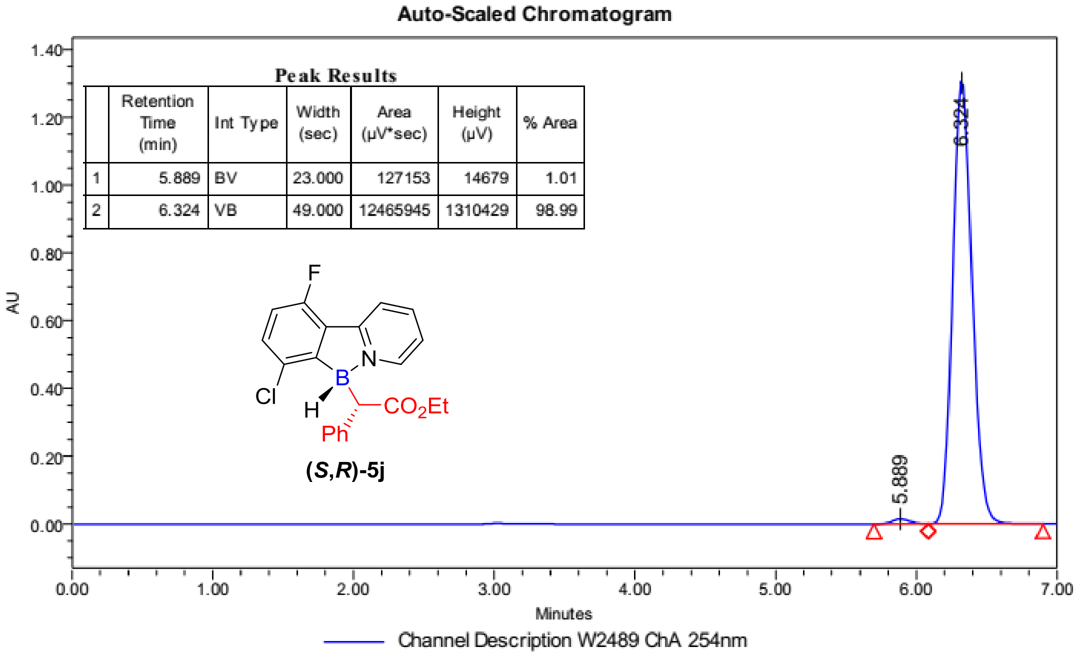

	Retention Time (min)	Int Type	Width (sec)	Area (µV*sec)	Height (µV)	% Area
1	5.889	BV	23.000	127153	14679	1.01
2	6.324	VB	49.000	12465945	1310429	98.99

Supplementary Figure 363. HPLC of compound (S,R)-5j

ethyl(S)-2-(7-chloro-10-methyl-6H-5λ^4-benzo[3,4][1,2]azaborolo[1,5-a]pyridin-6-yl)-2-phenylacetate (**5k**)

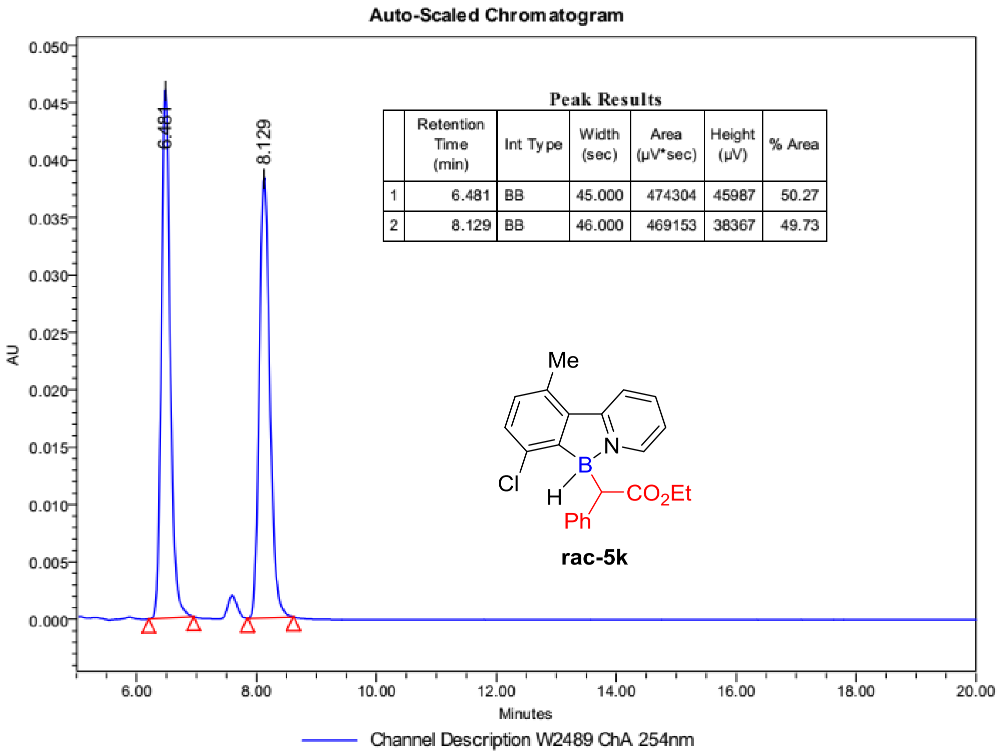

	Retention Time (min)	Int Type	Width (sec)	Area (μV*sec)	Height (μV)	% Area
1	6.481	BB	45.000	474304	45987	50.27
2	8.129	BB	46.000	469153	38367	49.73

Supplementary Figure 364. HPLC of compound rac-5k

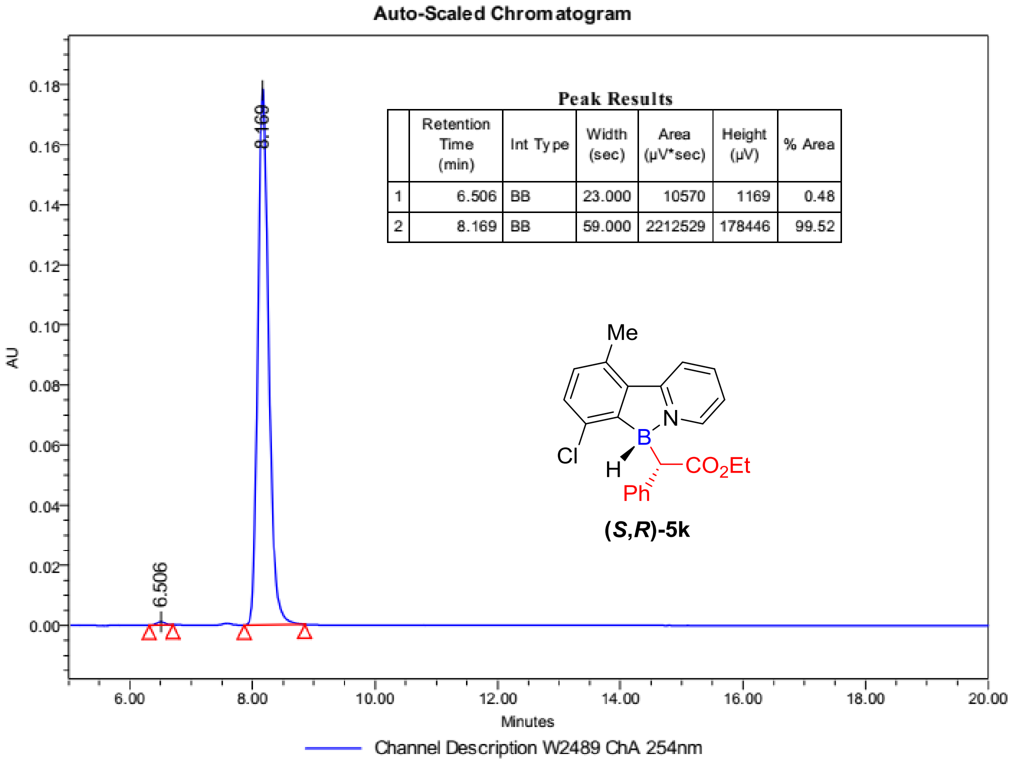

	Retention Time (min)	Int Type	Width (sec)	Area (μV*sec)	Height (μV)	% Area
1	6.506	BB	23.000	10570	1169	0.48
2	8.169	BB	59.000	2212529	178446	99.52

Supplementary Figure 365. HPLC of compound (S,R)-5k

ethyl(S)-2-phenyl-2-(7-(trifluoromethoxy)-6H-5λ[4]-benzo[3,4][1,2]azaborolo[1,5-a]pyridin-6-y -l)acetate (5l)

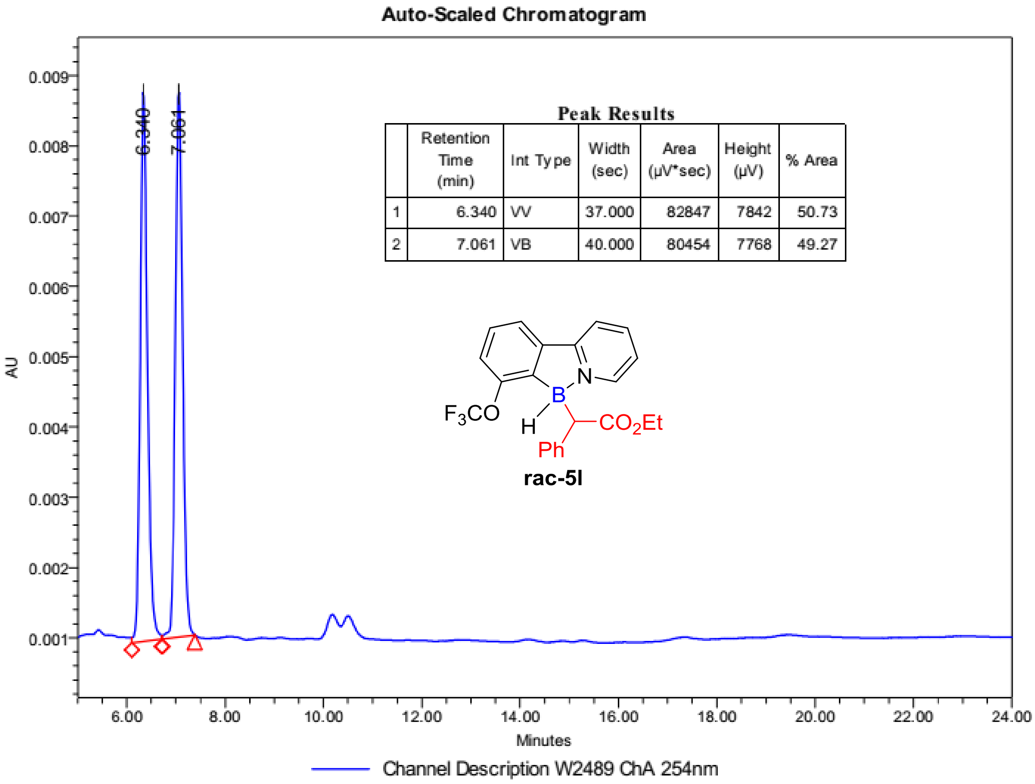

Peak Results

	Retention Time (min)	Int Type	Width (sec)	Area (μV*sec)	Height (μV)	% Area
1	6.340	VV	37.000	82847	7842	50.73
2	7.061	VB	40.000	80454	7768	49.27

Supplementary Figure 366. HPLC of compound rac-5l

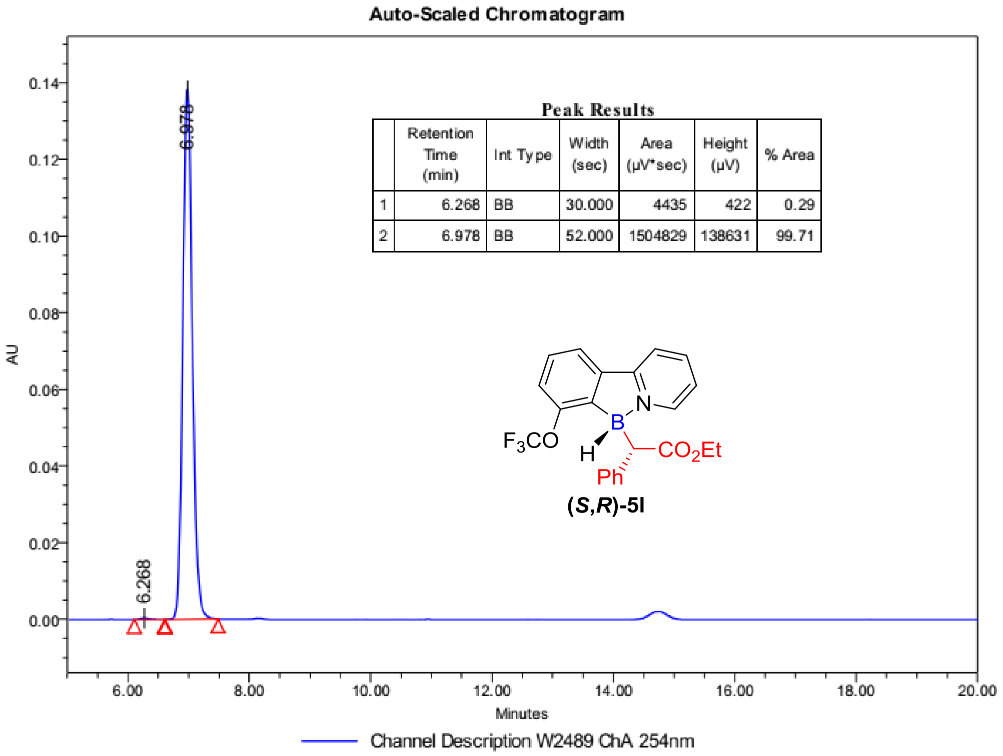

Peak Results

	Retention Time (min)	Int Type	Width (sec)	Area (μV*sec)	Height (μV)	% Area
1	6.268	BB	30.000	4435	422	0.29
2	6.978	BB	52.000	1504829	138631	99.71

Supplementary Figure 367. HPLC of compound (S,R)-5l

ethyl(*S*)-2-(7,9-dimethyl-(*R*)6H-5λ^4-benzo[3,4][1,2]azaborolo[1,5-a]pyridin-6-yl)-2-phenylacetate (5m)

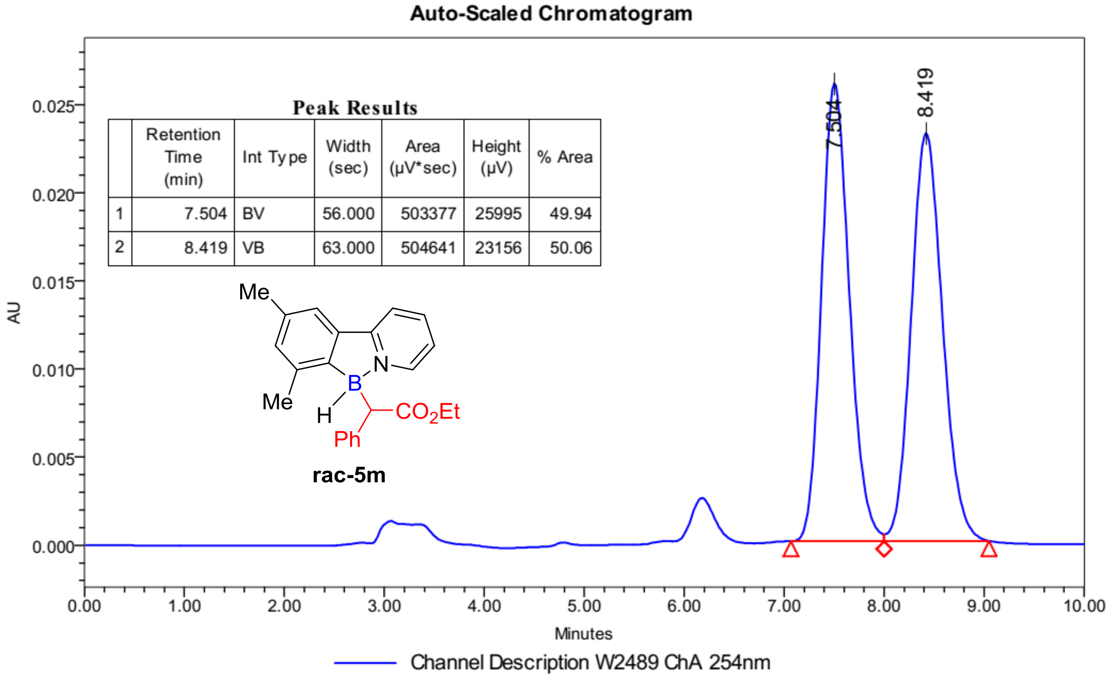

	Retention Time (min)	Int Type	Width (sec)	Area (µV*sec)	Height (µV)	% Area
1	7.504	BV	56.000	503377	25995	49.94
2	8.419	VB	63.000	504641	23156	50.06

Supplementary Figure 368. HPLC of compound rac-5m

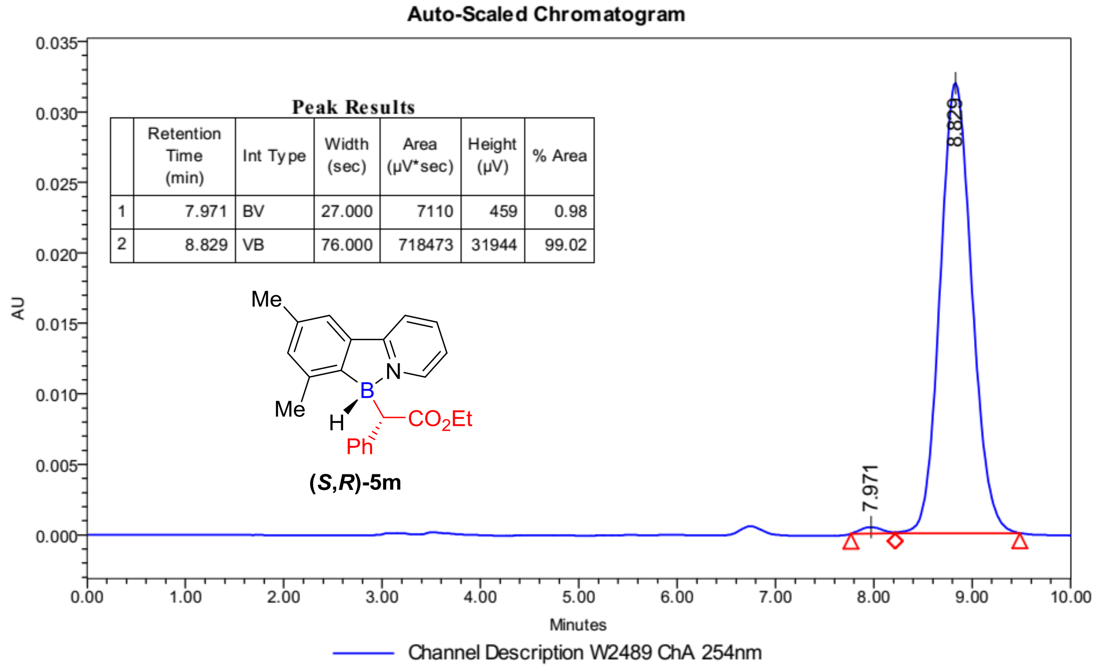

	Retention Time (min)	Int Type	Width (sec)	Area (µV*sec)	Height (µV)	% Area
1	7.971	BV	27.000	7110	459	0.98
2	8.829	VB	76.000	718473	31944	99.02

Supplementary Figure 369. HPLC of compound (S,R)-5m

ethyl (*S*)-2-((*R*)9H-8λ[4]-thieno[2',3':3,4][1,2]azaborolo[1,5-a]pyridin-9-yl)-2-phenylacetate (5n)

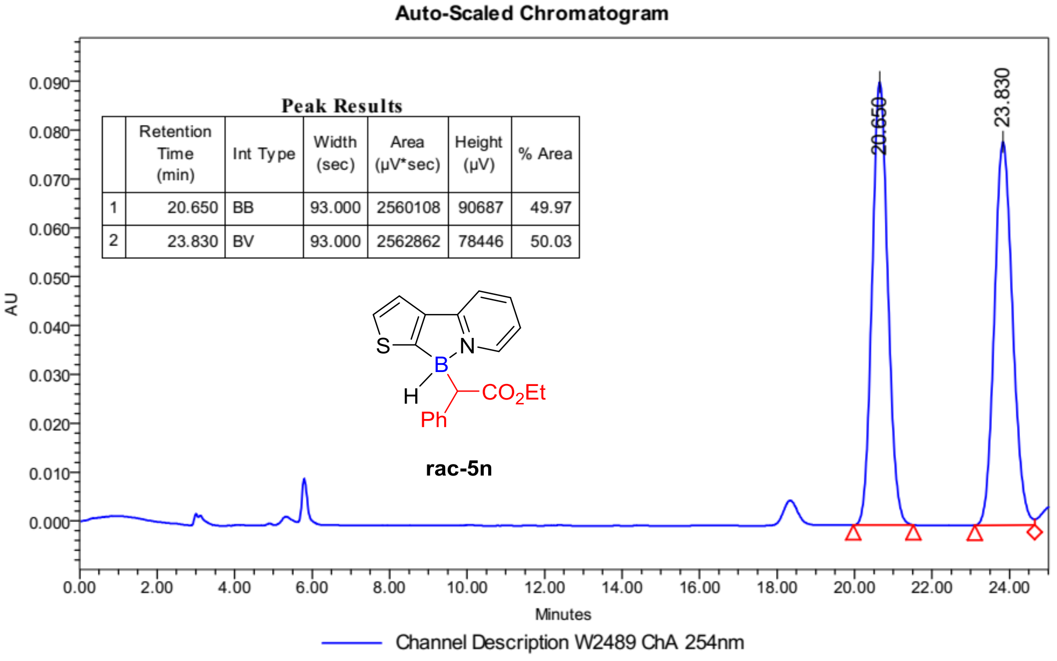

	Retention Time (min)	Int Type	Width (sec)	Area (μV*sec)	Height (μV)	% Area
1	20.650	BB	93.000	2560108	90687	49.97
2	23.830	BV	93.000	2562862	78446	50.03

Supplementary Figure 370. HPLC of compound rac-5n

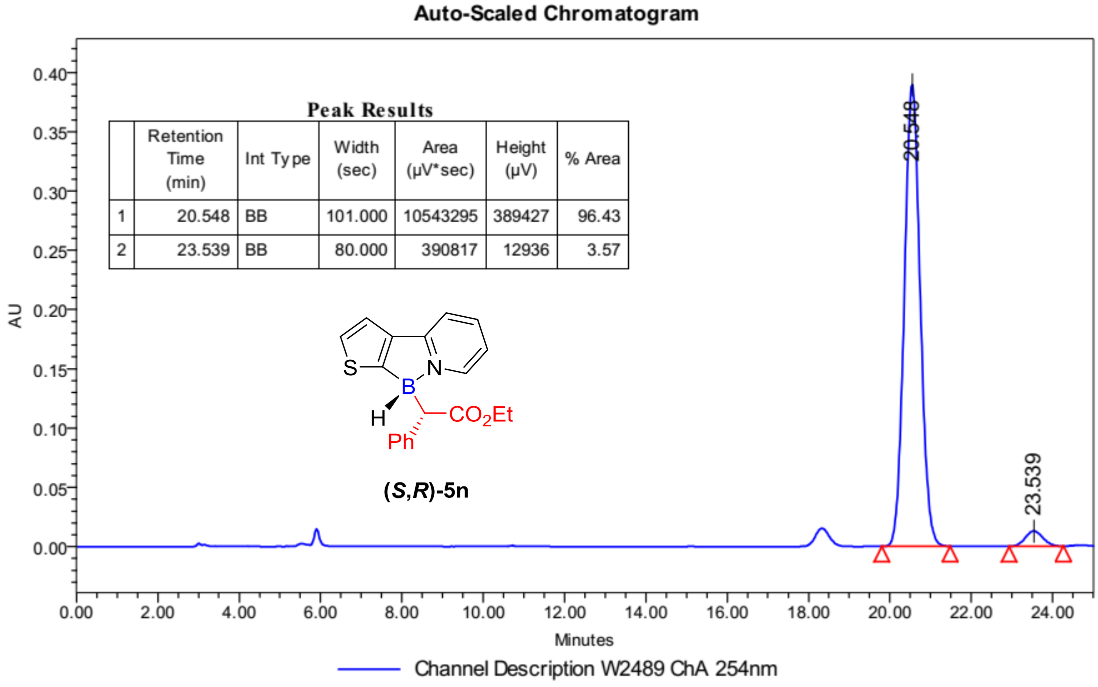

	Retention Time (min)	Int Type	Width (sec)	Area (μV*sec)	Height (μV)	% Area
1	20.548	BB	101.000	10543295	389427	96.43
2	23.539	BB	80.000	390817	12936	3.57

Supplementary Figure 371. HPLC of compound (S,R)-5n

ethyl(*S*)-2-((*R*)6H-7λ[4]-benzo[4',5']thieno[2',3':3,4][1,2]azaborolo[1,5-a]pyridin-6-yl)-2-phenylacetate (5o)

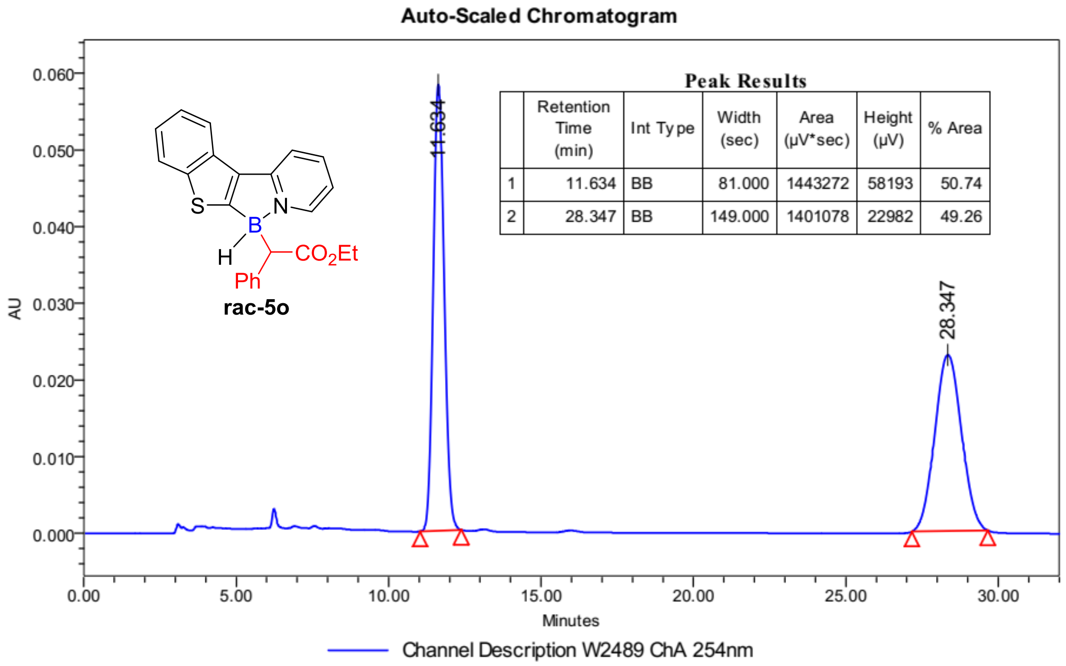

Peak Results

	Retention Time (min)	Int Type	Width (sec)	Area (μV*sec)	Height (μV)	% Area
1	11.634	BB	81.000	1443272	58193	50.74
2	28.347	BB	149.000	1401078	22982	49.26

Supplementary Figure 372. HPLC of compound rac-5o

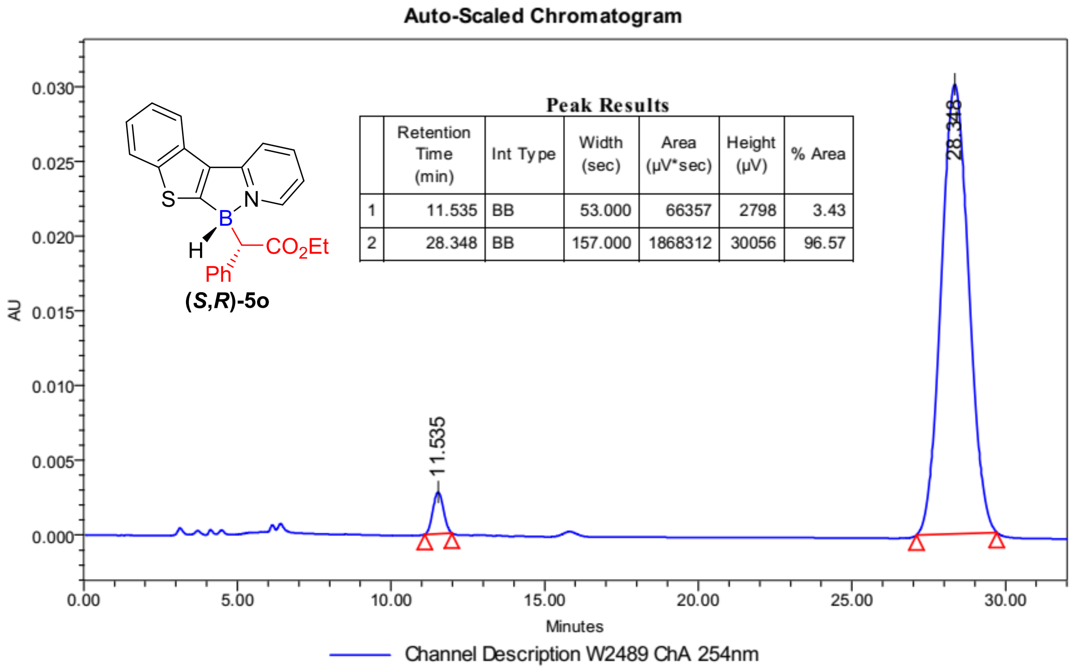

Peak Results

	Retention Time (min)	Int Type	Width (sec)	Area (μV*sec)	Height (μV)	% Area
1	11.535	BB	53.000	66357	2798	3.43
2	28.348	BB	157.000	1868312	30056	96.57

Supplementary Figure 373. HPLC of compound (S,R)-5o

ethyl (*S*)-2-(1-fluoro-(*R*)6H-5λ[4]-benzo[3,4][1,2]azaborolo[1,5-a]pyridin-6-yl)-2-phenylacetate (5p)

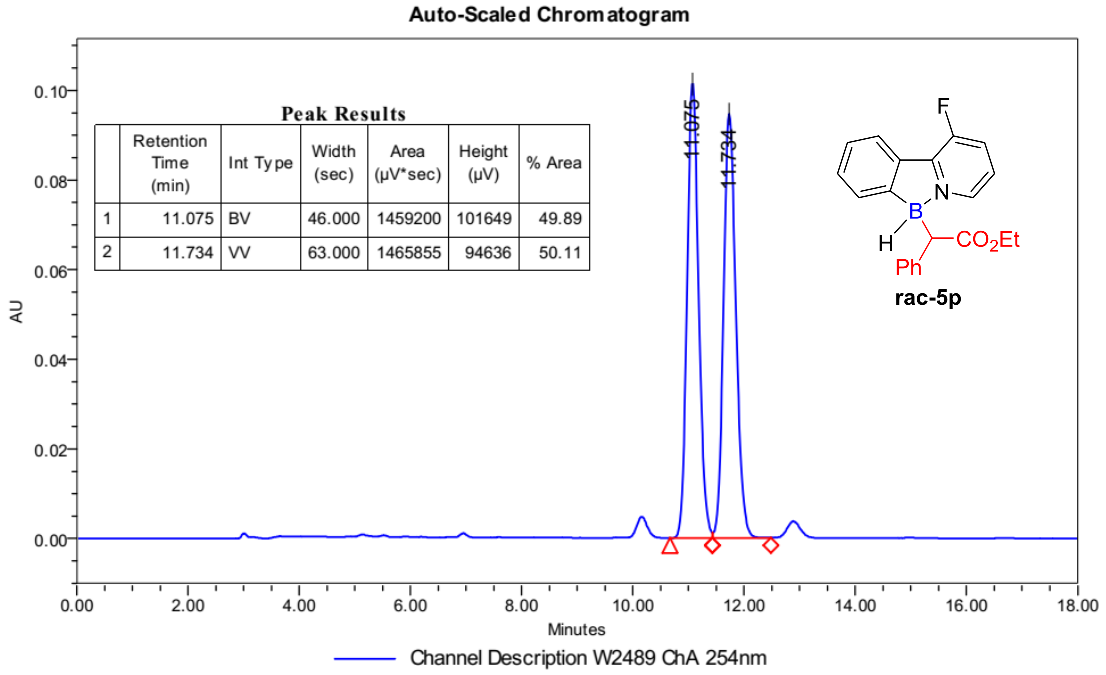

	Retention Time (min)	Int Type	Width (sec)	Area (μV*sec)	Height (μV)	% Area
1	11.075	BV	46.000	1459200	101649	49.89
2	11.734	VV	63.000	1465855	94636	50.11

Supplementary Figure 374. HPLC of compound rac-5p

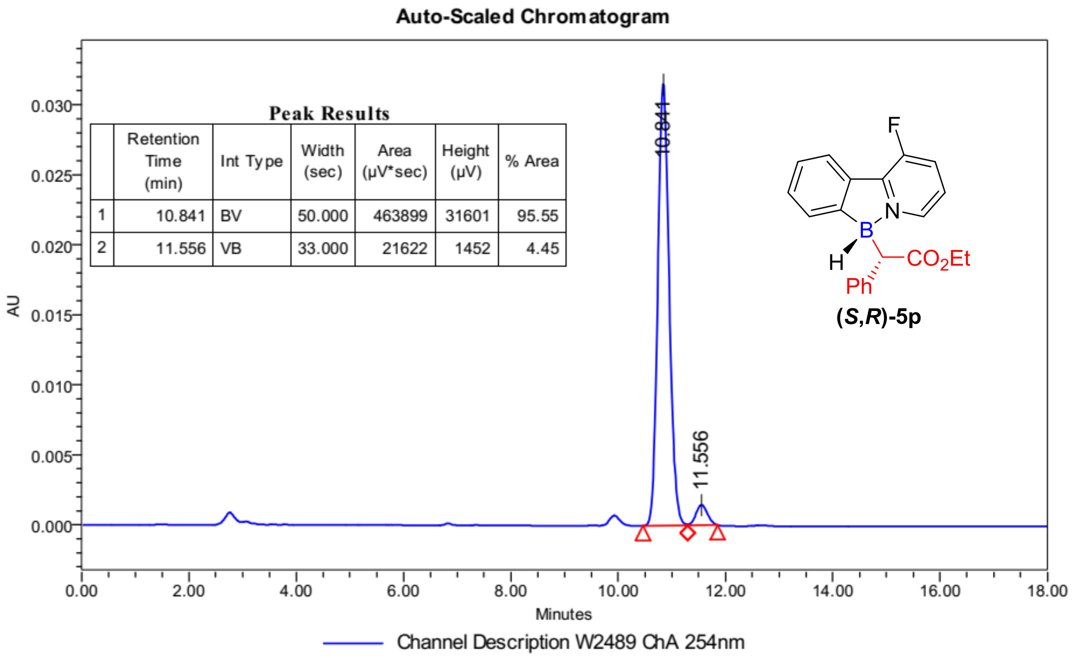

	Retention Time (min)	Int Type	Width (sec)	Area (μV*sec)	Height (μV)	% Area
1	10.841	BV	50.000	463899	31601	95.55
2	11.556	VB	33.000	21622	1452	4.45

Supplementary Figure 375. HPLC of compound (S,R)-5p

methyl (*S*)-2-(7-chloro-6H-5λ[4]-benzo[3,4][1,2]azaborolo[1,5-a]pyridin-6-yl)-2-phenylacetate (5q)

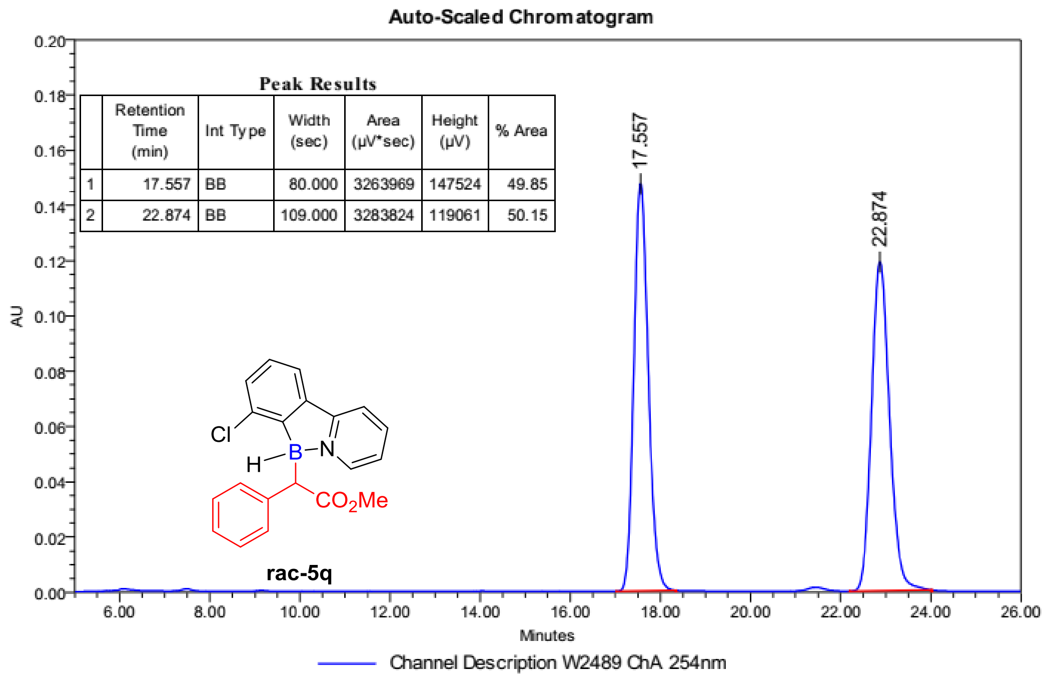

	Retention Time (min)	Int Type	Width (sec)	Area (µV*sec)	Height (µV)	% Area
1	17.557	BB	80.000	3263969	147524	49.85
2	22.874	BB	109.000	3283824	119061	50.15

Supplementary Figure 376. HPLC of compound rac-5q

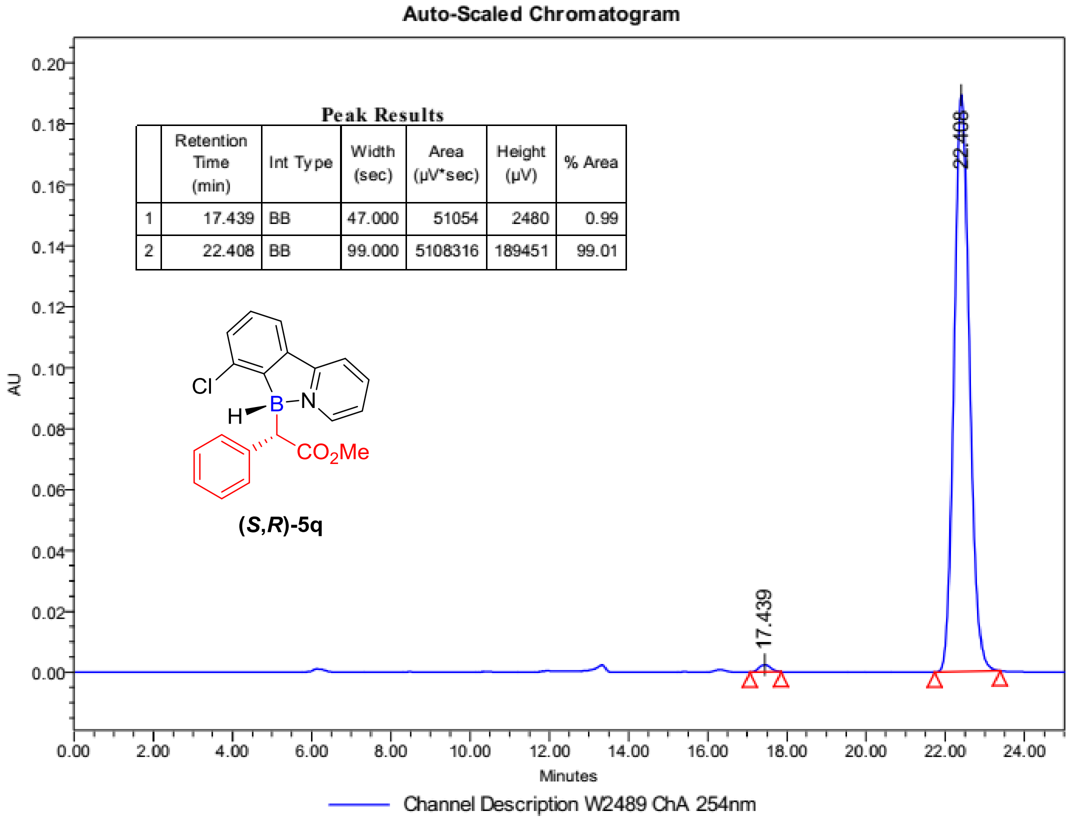

	Retention Time (min)	Int Type	Width (sec)	Area (µV*sec)	Height (µV)	% Area
1	17.439	BB	47.000	51054	2480	0.99
2	22.408	BB	99.000	5108316	189451	99.01

Supplementary Figure 377. HPLC of compound (S,R)-5q

benzyl (S)-2-(7-chloro-6H-5λ[4]-benzo[3,4][1,2]azaborolo[1,5-a]pyridin-6-yl)-2-phenylacetate (5r)

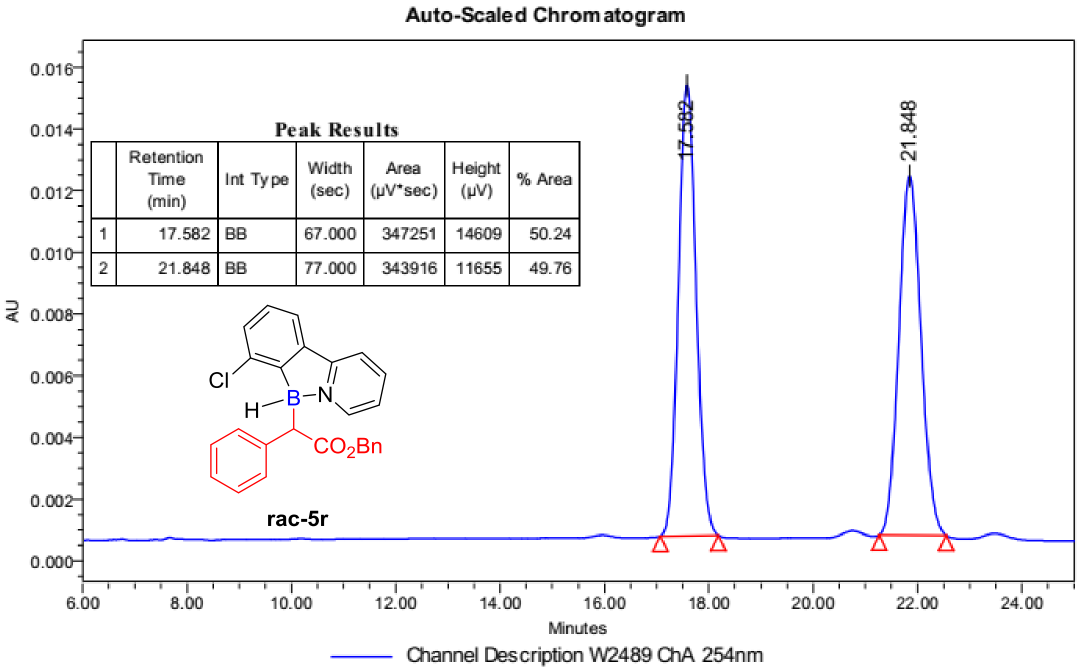

Peak Results

	Retention Time (min)	Int Type	Width (sec)	Area (µV*sec)	Height (µV)	% Area
1	17.582	BB	67.000	347251	14609	50.24
2	21.848	BB	77.000	343916	11655	49.76

Supplementary Figure 378. HPLC of compound rac-5r

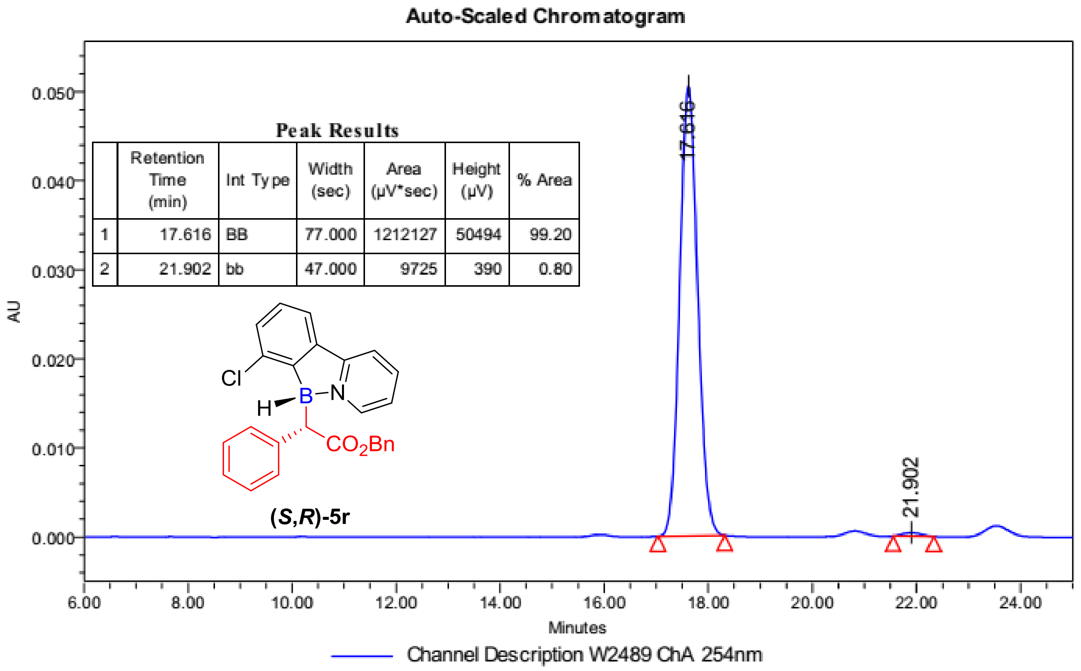

Peak Results

	Retention Time (min)	Int Type	Width (sec)	Area (µV*sec)	Height (µV)	% Area
1	17.616	BB	77.000	1212127	50494	99.20
2	21.902	bb	47.000	9725	390	0.80

Supplementary Figure 379. HPLC of compound (S,R)-5r

benzyl (S)-2-(7-chloro-6H-5λ^4-benzo[3,4][1,2]azaborolo[1,5-a]pyridin-6-yl)-2-phenylacetate(5s)

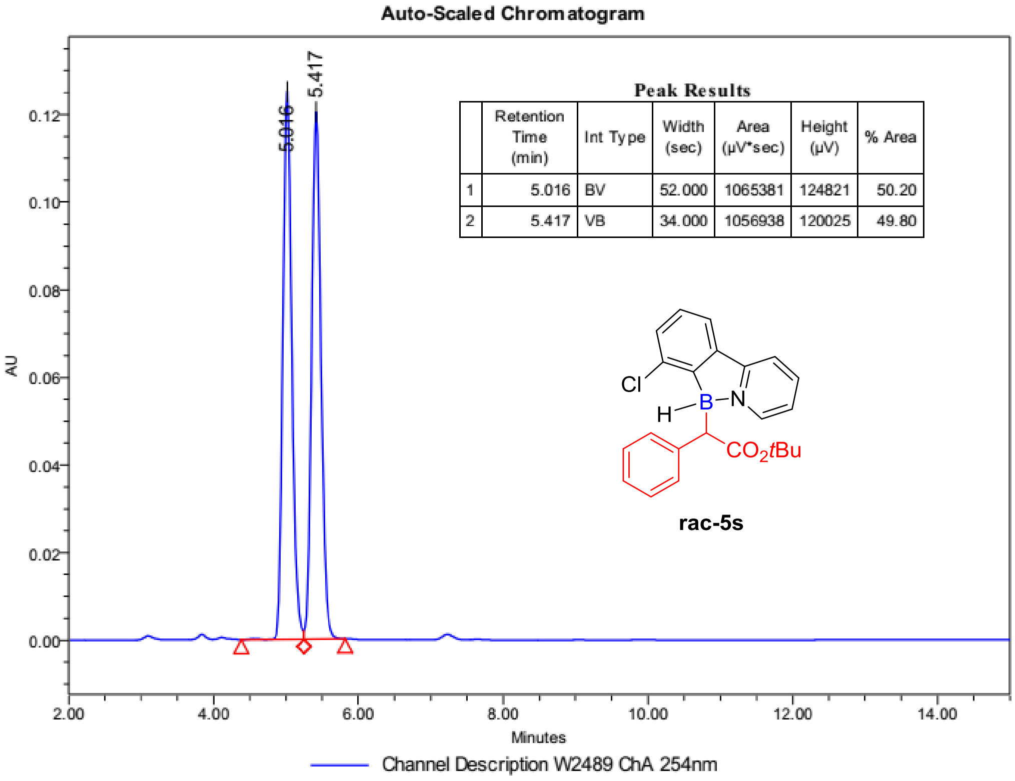

	Retention Time (min)	Int Type	Width (sec)	Area (µV*sec)	Height (µV)	% Area
1	5.016	BV	52.000	1065381	124821	50.20
2	5.417	VB	34.000	1056938	120025	49.80

Supplementary Figure 380. HPLC of compound rac-5s

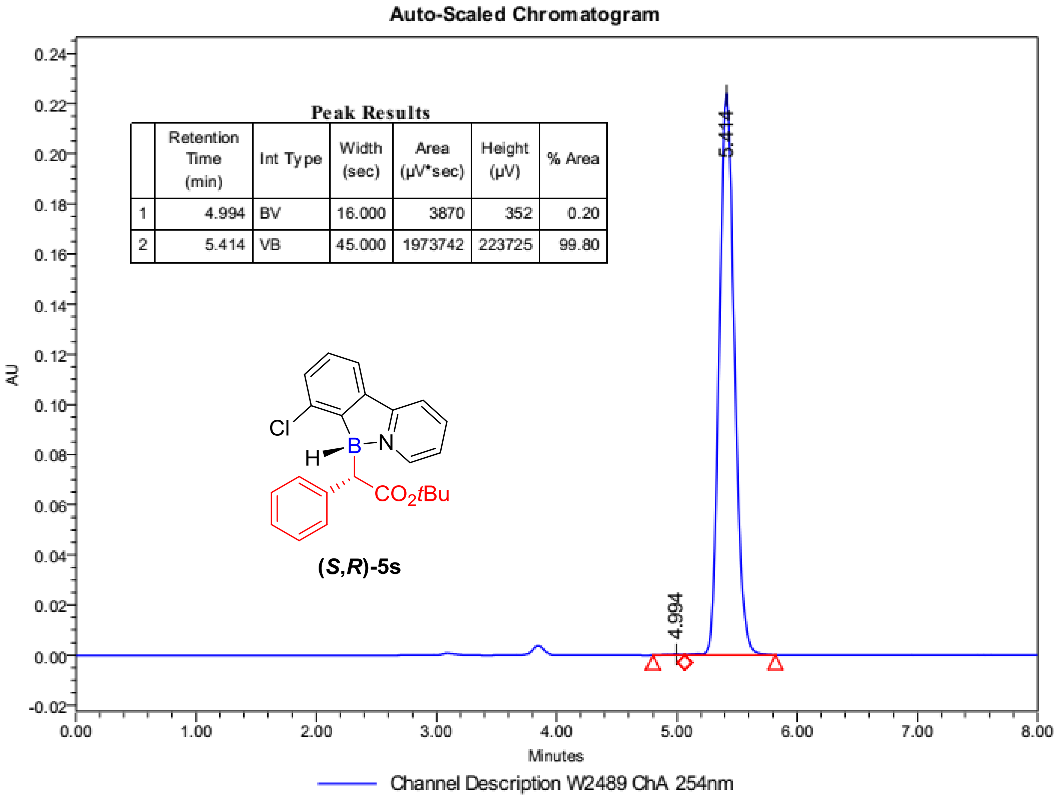

	Retention Time (min)	Int Type	Width (sec)	Area (µV*sec)	Height (µV)	% Area
1	4.994	BV	16.000	3870	352	0.20
2	5.414	VB	45.000	1973742	223725	99.80

Supplementary Figure 381. HPLC of compound (S,R)-5s

phenyl (S)-2-(7-chloro-6H-5λ[4]-benzo[3,4][1,2]azaborolo[1,5-a]pyridin-6-yl)-2-phenylacetate(5t)

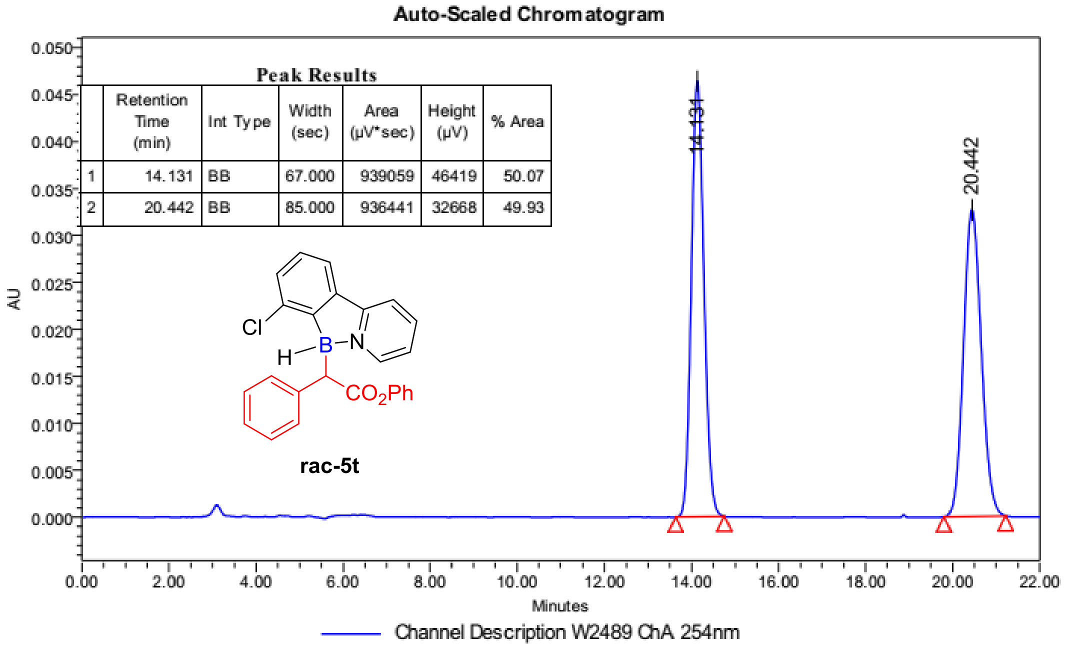

	Retention Time (min)	Int Type	Width (sec)	Area (μV*sec)	Height (μV)	% Area
1	14.131	BB	67.000	939059	46419	50.07
2	20.442	BB	85.000	936441	32668	49.93

Supplementary Figure 382. HPLC of compound rac-5t

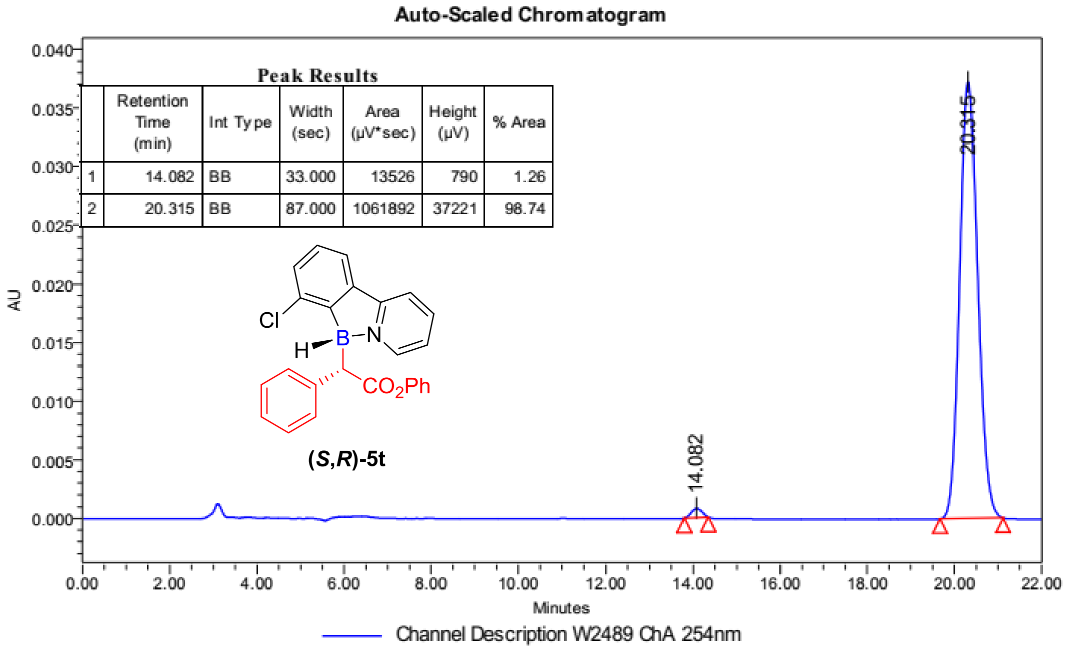

	Retention Time (min)	Int Type	Width (sec)	Area (μV*sec)	Height (μV)	% Area
1	14.082	BB	33.000	13526	790	1.26
2	20.315	BB	87.000	1061892	37221	98.74

Supplementary Figure 383. HPLC of compound (S,R)-5t

ethyl(*S*)-2-(4-(tert-butyl)phenyl)-2-(7-chloro-6H-5λ[4]-benzo[3,4][1,2]azaborolo[1,5-a]pyridin-6-yl) acetate (5u)

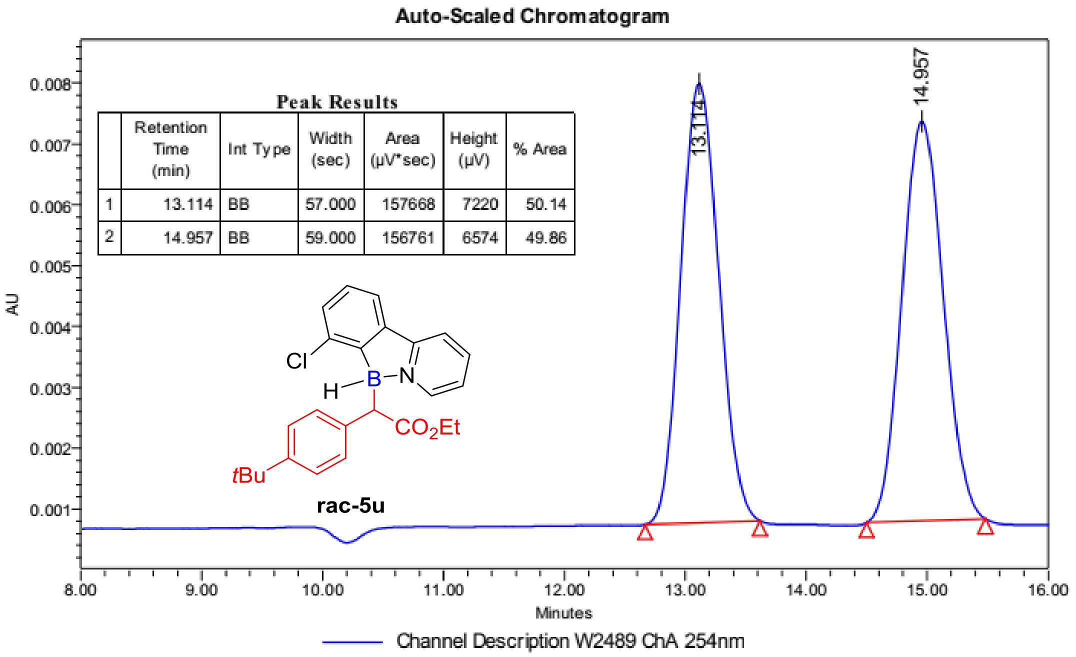

	Retention Time (min)	Int Type	Width (sec)	Area (µV*sec)	Height (µV)	% Area
1	13.114	BB	57.000	157668	7220	50.14
2	14.957	BB	59.000	156761	6574	49.86

Supplementary Figure 384. HPLC of compound rac-5u

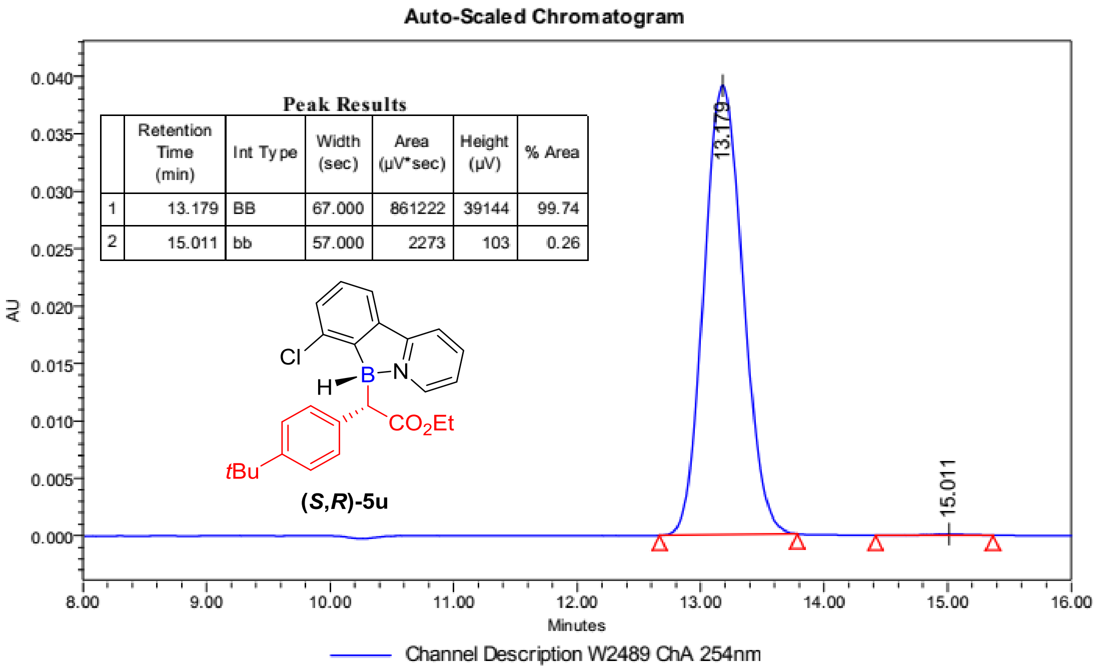

	Retention Time (min)	Int Type	Width (sec)	Area (µV*sec)	Height (µV)	% Area
1	13.179	BB	67.000	861222	39144	99.74
2	15.011	bb	57.000	2273	103	0.26

Supplementary Figure 385. HPLC of compound (S,R)-5u

ethyl (S)-2-(7-chloro-6H-5λ[4]-benzo[3,4][1,2]azaborolo[1,5-a]pyridin-6-yl)-2-(p-tolyl)acetate (5v)

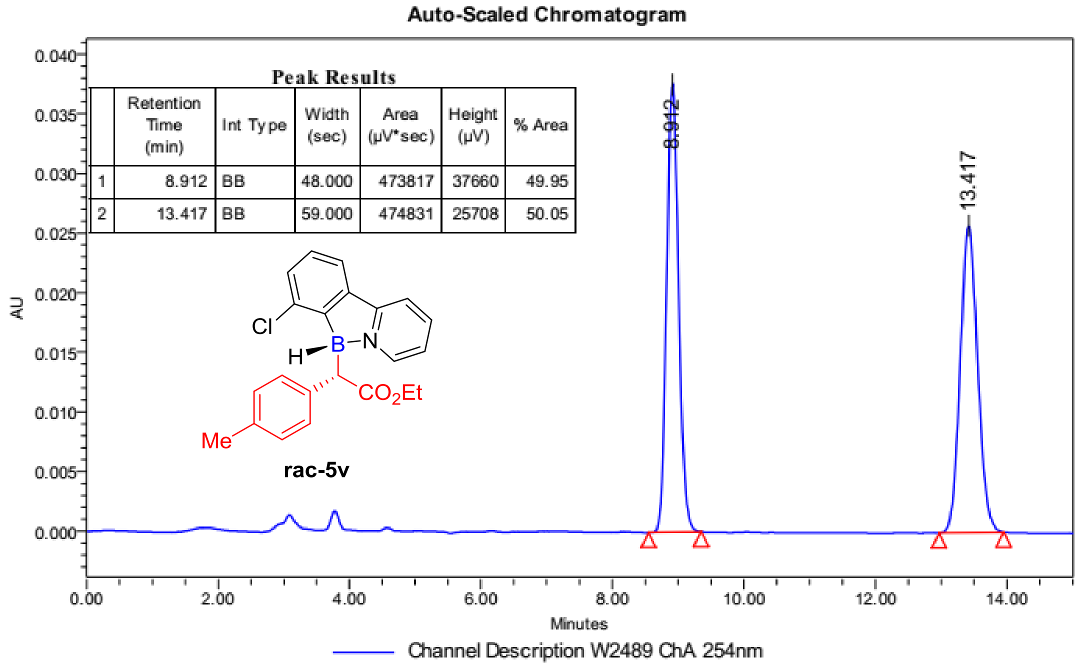

	Retention Time (min)	Int Type	Width (sec)	Area (µV*sec)	Height (µV)	% Area
1	8.912	BB	48.000	473817	37660	49.95
2	13.417	BB	59.000	474831	25708	50.05

Supplementary Figure 386. HPLC of compound rac-5v

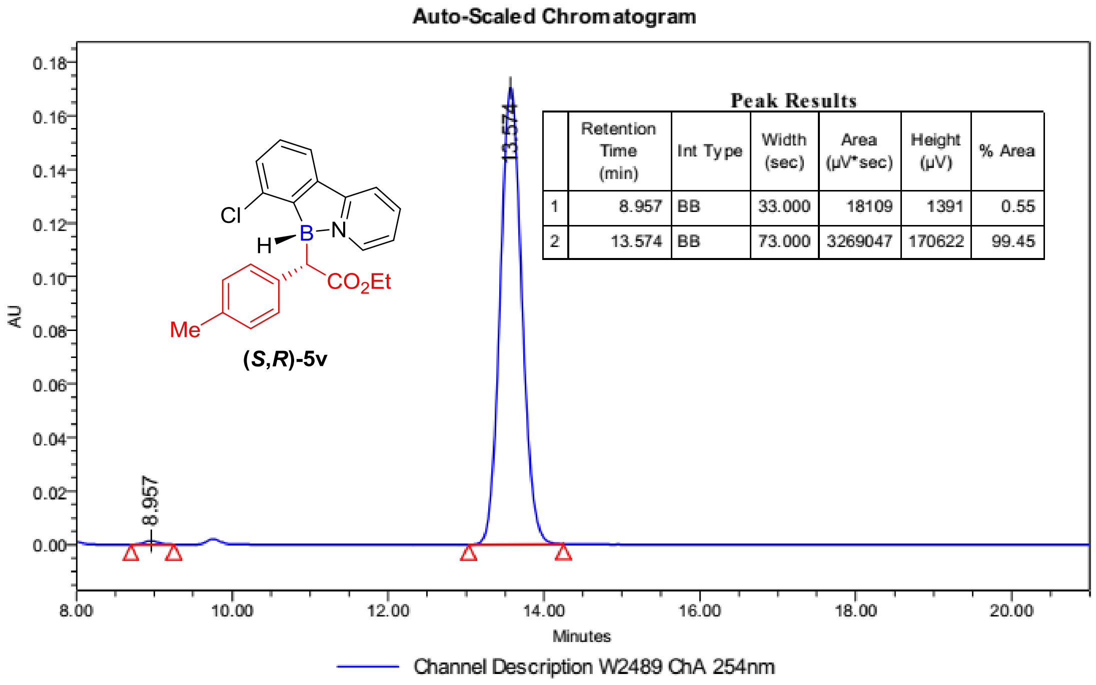

	Retention Time (min)	Int Type	Width (sec)	Area (µV*sec)	Height (µV)	% Area
1	8.957	BB	33.000	18109	1391	0.55
2	13.574	BB	73.000	3269047	170622	99.45

Supplementary Figure 387. HPLC of compound (S,R)-5v

ethyl (*S*)-2-(7-chloro-6H-5λ[4]-benzo[3,4][1,2]azaborolo[1,5-a]pyridin-6-yl)-2-(m-tolyl)acetate (5w)

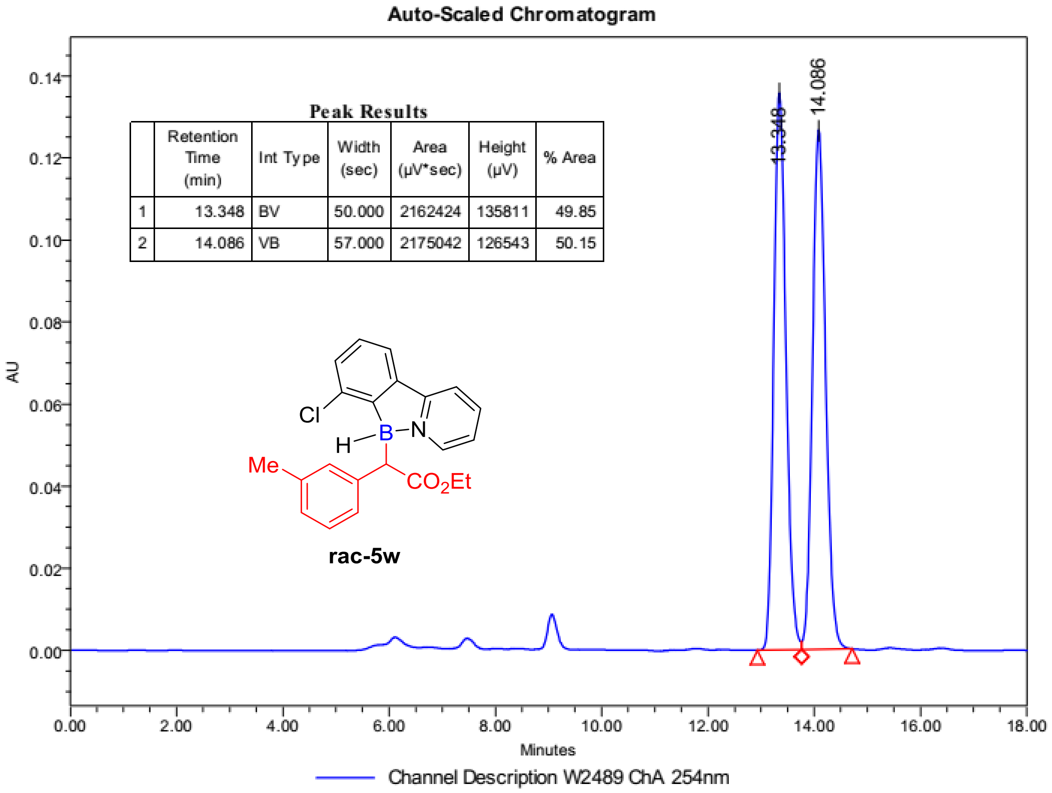

	Retention Time (min)	Int Type	Width (sec)	Area (μV*sec)	Height (μV)	% Area
1	13.348	BV	50.000	2162424	135811	49.85
2	14.086	VB	57.000	2175042	126543	50.15

Supplementary Figure 388. HPLC of compound rac-5w

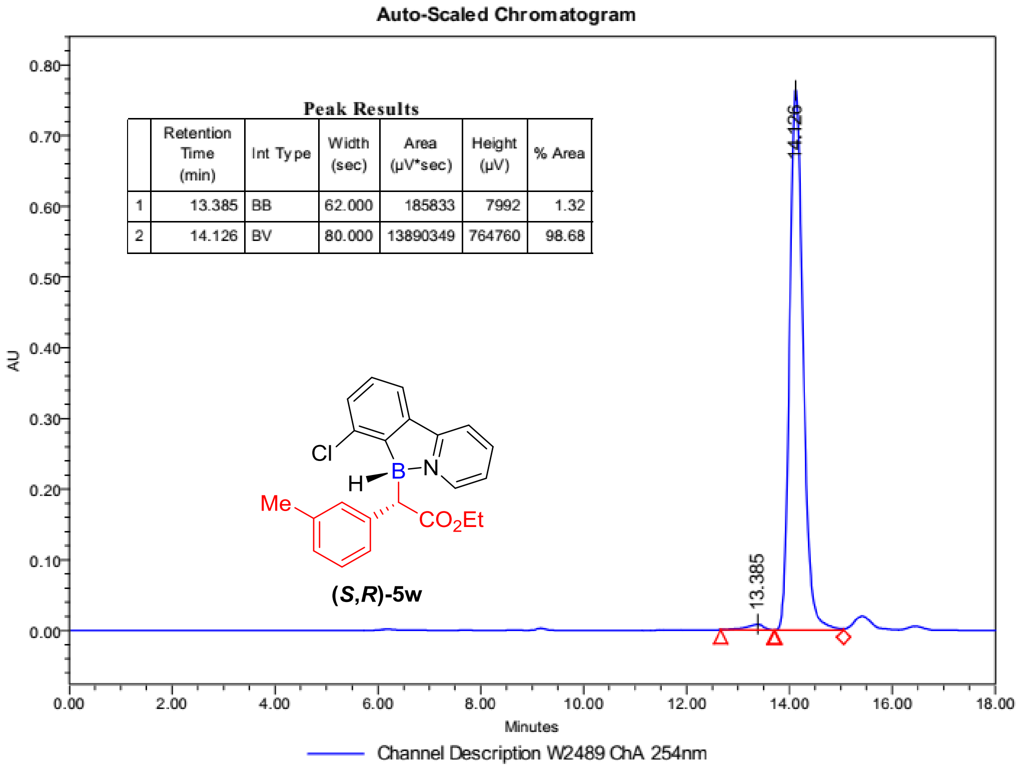

	Retention Time (min)	Int Type	Width (sec)	Area (μV*sec)	Height (μV)	% Area
1	13.385	BB	62.000	185833	7992	1.32
2	14.126	BV	80.000	13890349	764760	98.68

Supplementary Figure 389. HPLC of compound (S,R)-5w

ethyl(*S*)-2-(7-chloro-6H-5λ[4]-benzo[3,4][1,2]azaborolo[1,5-a]pyridin-6-yl)-2-(3-methoxyphenyl)acetate (5x)

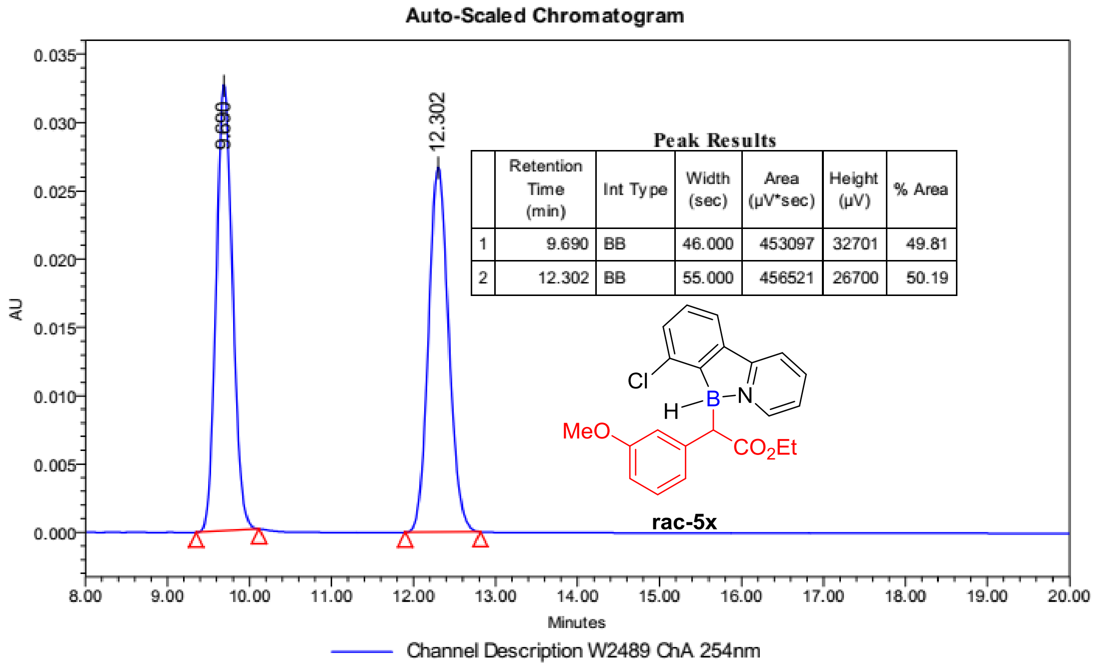

Peak Results

	Retention Time (min)	Int Type	Width (sec)	Area (µV*sec)	Height (µV)	% Area
1	9.690	BB	46.000	453097	32701	49.81
2	12.302	BB	55.000	456521	26700	50.19

Supplementary Figure 390. HPLC of compound rac-5x

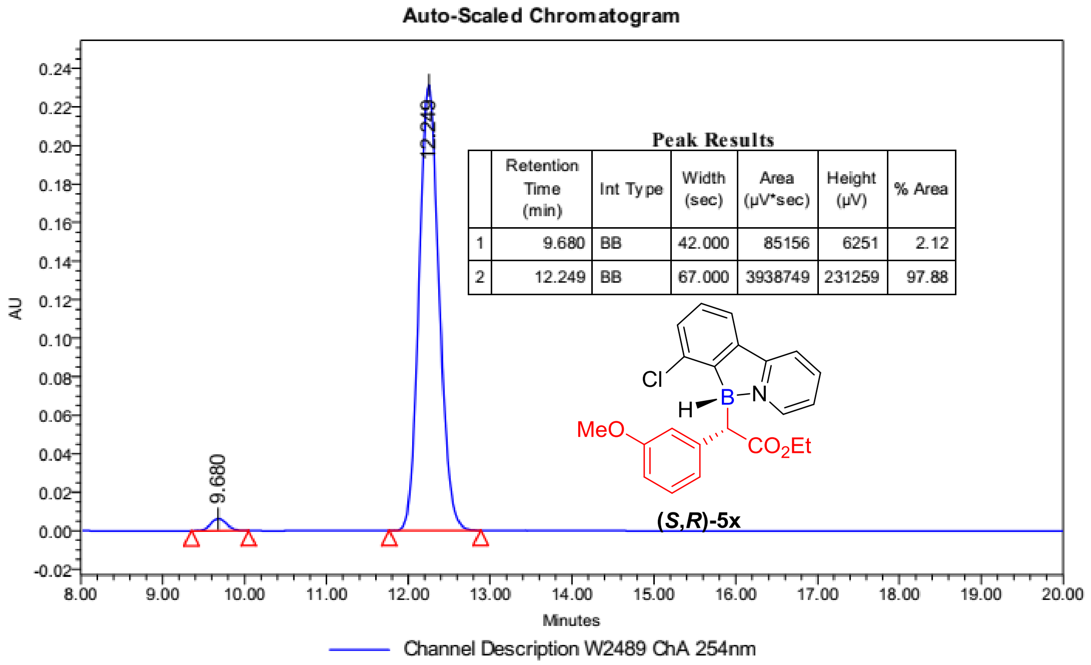

Peak Results

	Retention Time (min)	Int Type	Width (sec)	Area (µV*sec)	Height (µV)	% Area
1	9.680	BB	42.000	85156	6251	2.12
2	12.249	BB	67.000	3938749	231259	97.88

Supplementary Figure 391. HPLC of compound (S,R)-5x

ethyl(*S*)-2-(benzo[d][1,3]dioxol-5-yl)-2-(7-chloro-6H-5λ^4-benzo[3,4][1,2]azaborolo[1,5-a]pyridin-6-yl)acetate (5y)

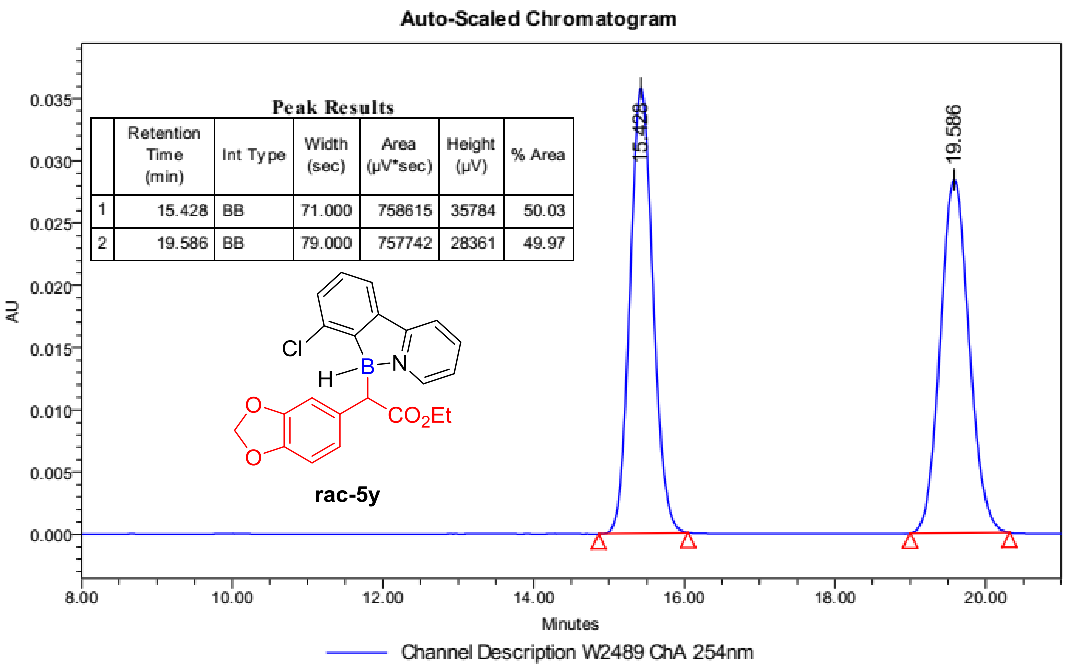

	Retention Time (min)	Int Type	Width (sec)	Area (µV*sec)	Height (µV)	% Area
1	15.428	BB	71.000	758615	35784	50.03
2	19.586	BB	79.000	757742	28361	49.97

Supplementary Figure 392. HPLC of compound rac-5y

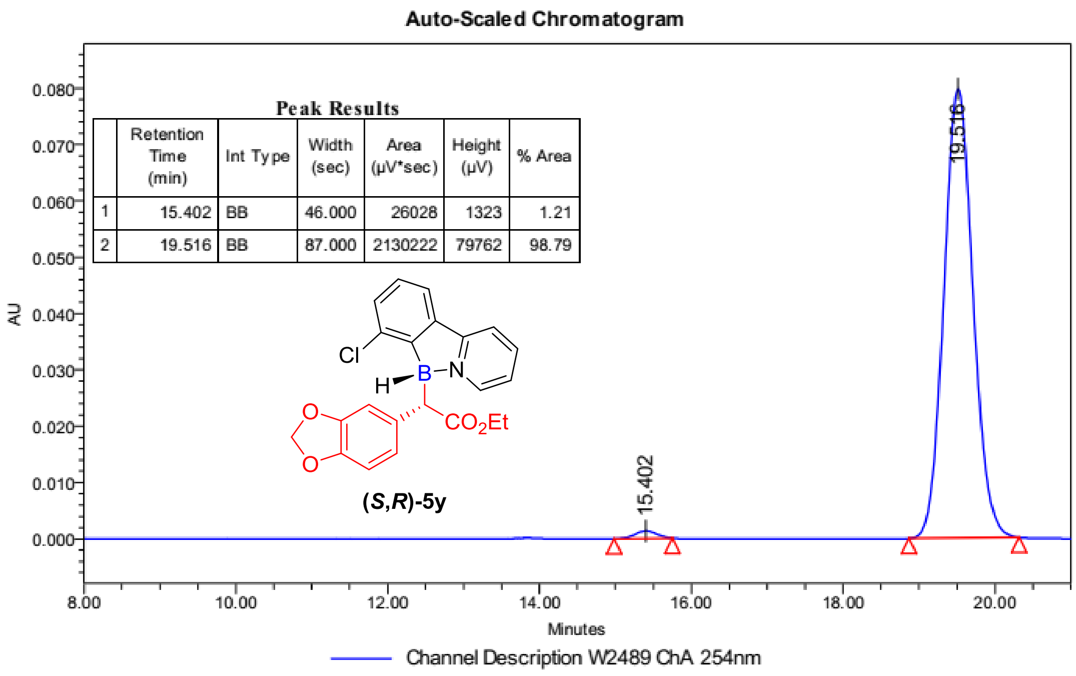

	Retention Time (min)	Int Type	Width (sec)	Area (µV*sec)	Height (µV)	% Area
1	15.402	BB	46.000	26028	1323	1.21
2	19.516	BB	87.000	2130222	79762	98.79

Supplementary Figure 393. HPLC of compound (S,R)-5y

ethyl(*S*)-2-([1,1'-biphenyl]-4-yl)-2-(7-chloro-6H-5λ[4]-benzo[3,4][1,2]azaborolo[1,5-a]pyridin-6-yl)acetate (5z)

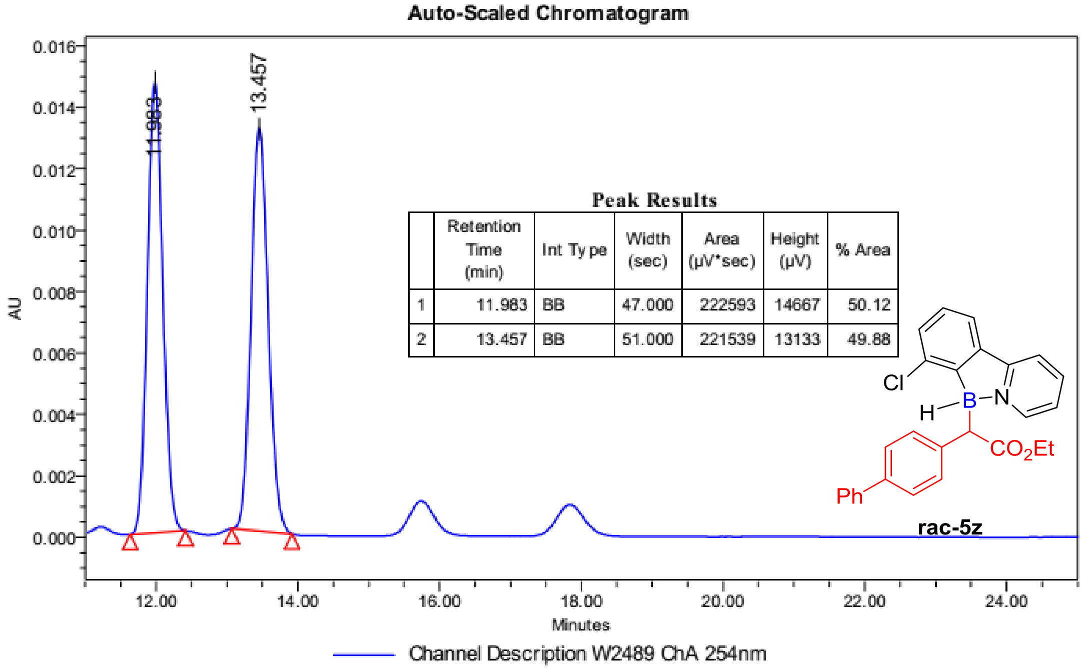

Peak Results

	Retention Time (min)	Int Type	Width (sec)	Area (µV*sec)	Height (µV)	% Area
1	11.983	BB	47.000	222593	14667	50.12
2	13.457	BB	51.000	221539	13133	49.88

Supplementary Figure 394. HPLC of compound rac-5z

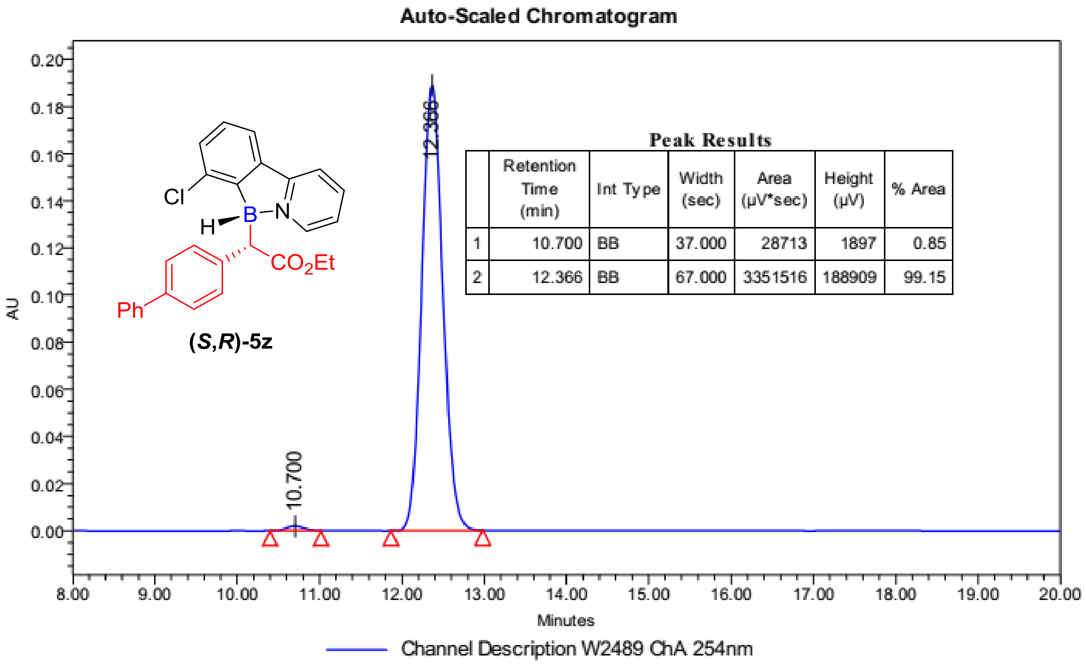

Peak Results

	Retention Time (min)	Int Type	Width (sec)	Area (µV*sec)	Height (µV)	% Area
1	10.700	BB	37.000	28713	1897	0.85
2	12.366	BB	67.000	3351516	188909	99.15

Supplementary Figure 395. HPLC of compound (S,R)-5z

methyl(*S*)-3-(1-(7-chloro-(*R*)6H-5λ[4]-benzo[3,4][1,2]azaborolo[1,5-a]pyridin-6-yl)-2-ethoxy-2-oxoethyl)benz-oate (5aa)

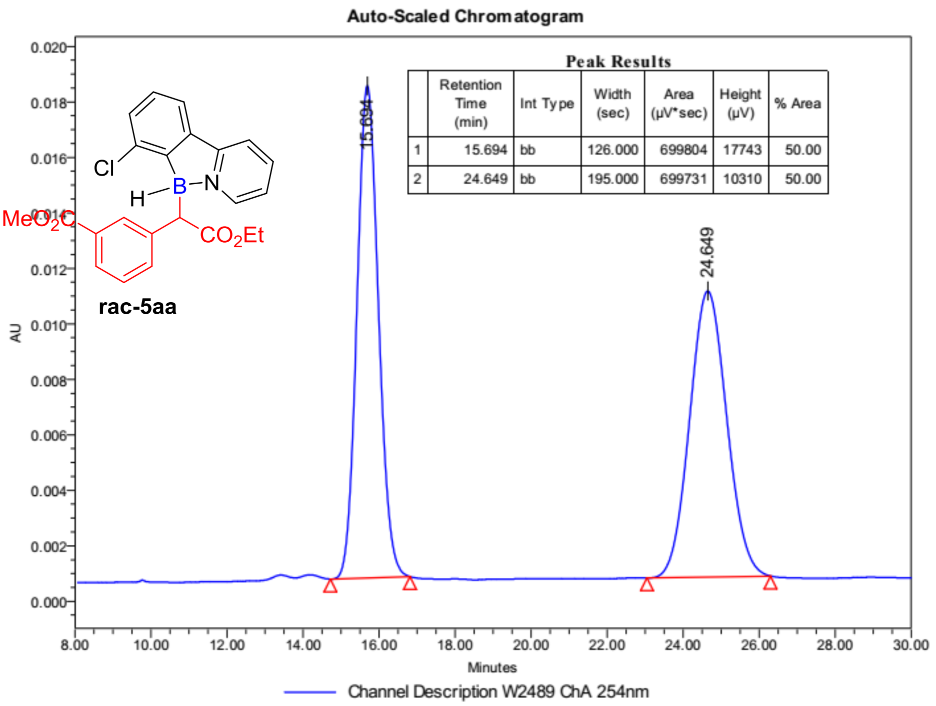

	Retention Time (min)	Int Type	Width (sec)	Area (µV*sec)	Height (µV)	% Area
1	15.694	bb	126.000	699804	17743	50.00
2	24.649	bb	195.000	699731	10310	50.00

Supplementary Figure 396. HPLC of compound rac-5aa

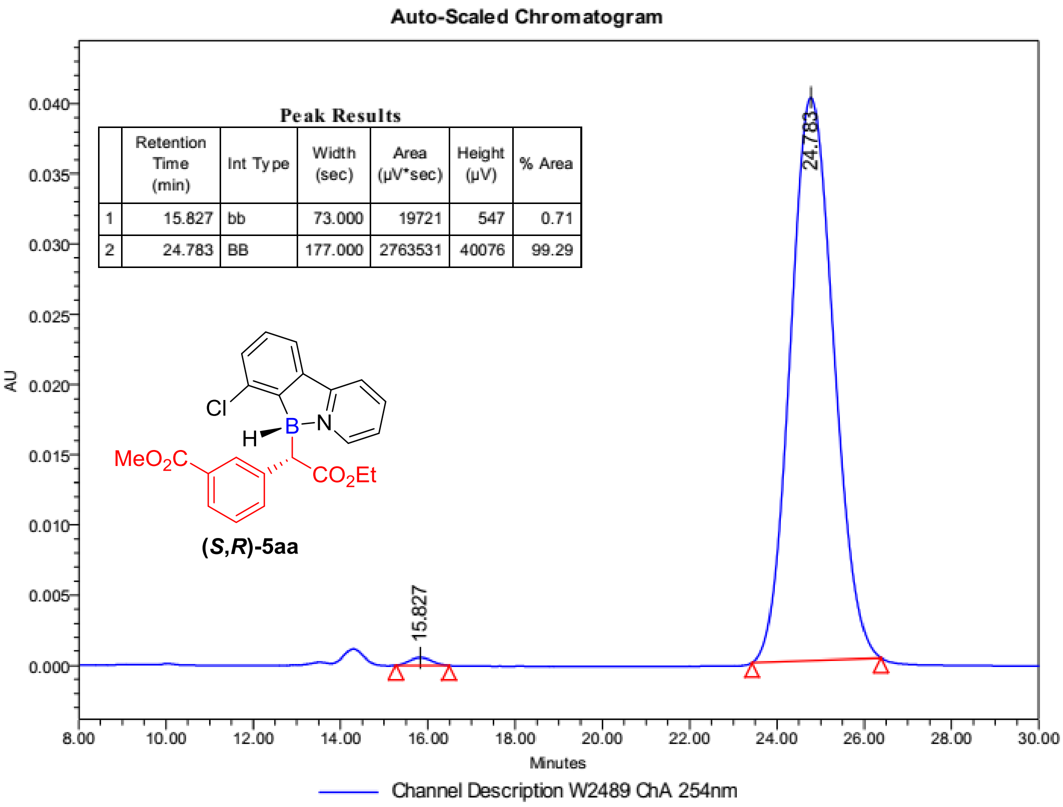

	Retention Time (min)	Int Type	Width (sec)	Area (µV*sec)	Height (µV)	% Area
1	15.827	bb	73.000	19721	547	0.71
2	24.783	BB	177.000	2763531	40076	99.29

Supplementary Figure 397. HPLC of compound (S,R)-5aa

ethyl(S)-2-(7-chloro-6H-5λ^4-benzo[3,4][1,2]azaborolo[1,5-a]pyridin-6-yl)-2-(4-fluorophenyl)acetate (5ab)

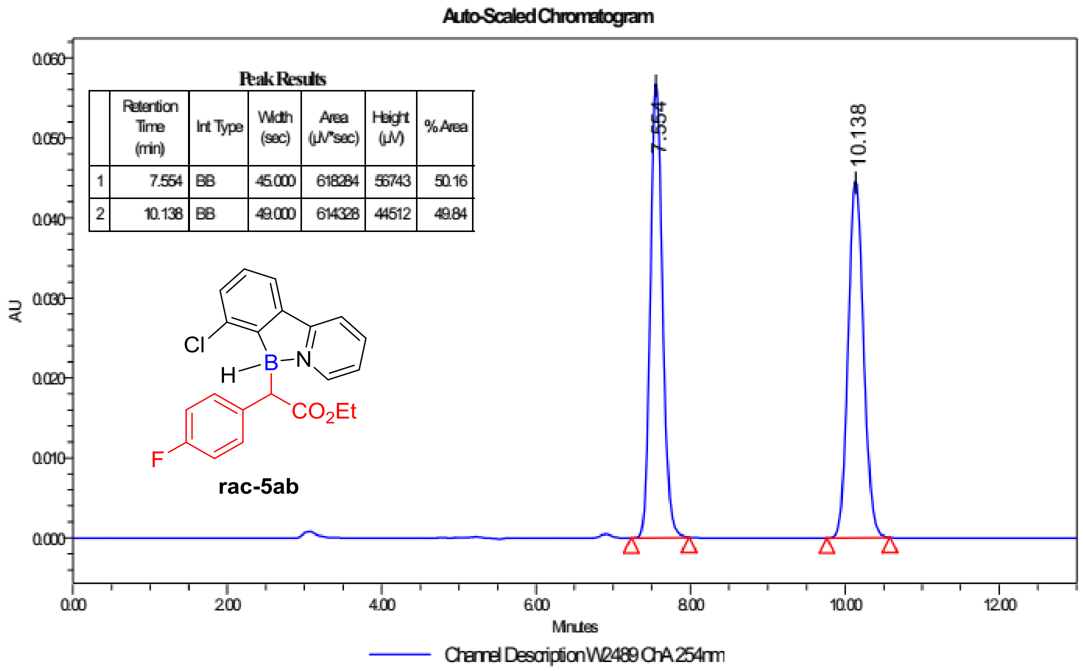

	Retention Time (min)	Int Type	Width (sec)	Area (µV*sec)	Height (µV)	% Area
1	7.554	BB	45.000	618284	56743	50.16
2	10.138	BB	49.000	614328	44512	49.84

Supplementary Figure 398. HPLC of compound rac-5ab

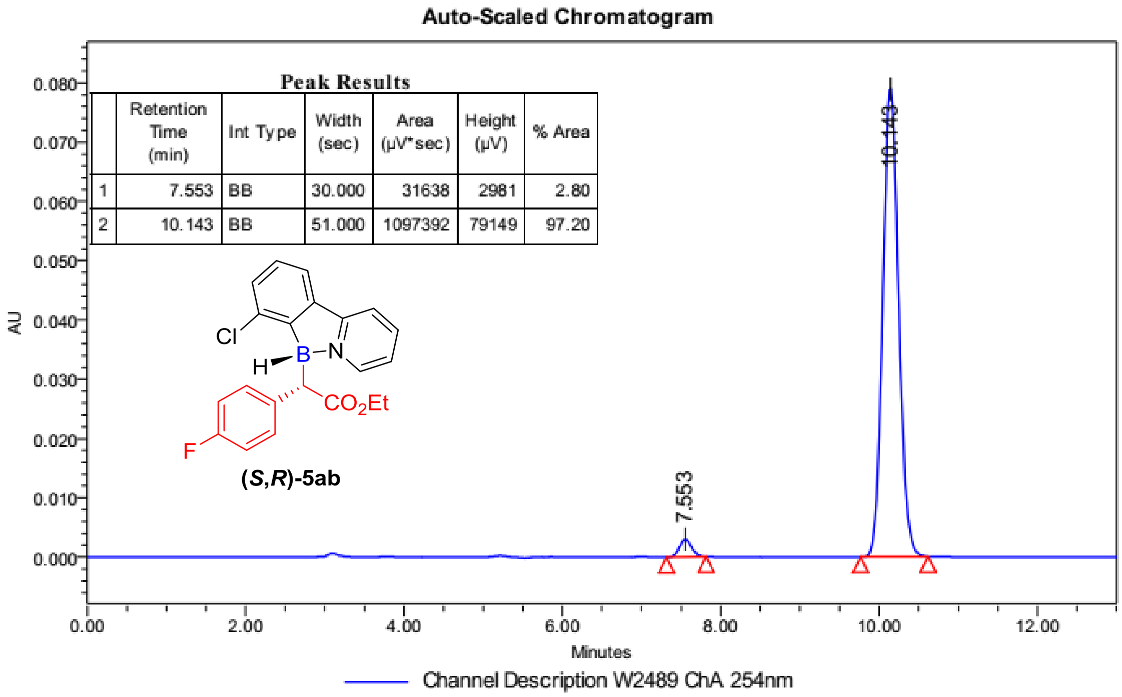

	Retention Time (min)	Int Type	Width (sec)	Area (µV*sec)	Height (µV)	% Area
1	7.553	BB	30.000	31638	2981	2.80
2	10.143	BB	51.000	1097392	79149	97.20

Supplementary Figure 399. HPLC of compound (S,R)-5ab

ethyl(*S*)-2-(7-chloro-6H-5λ[4]-benzo[3,4][1,2]azaborolo[1,5-a]pyridin-6-yl)-2-(4-chlorophenyl)acetate (5ac)

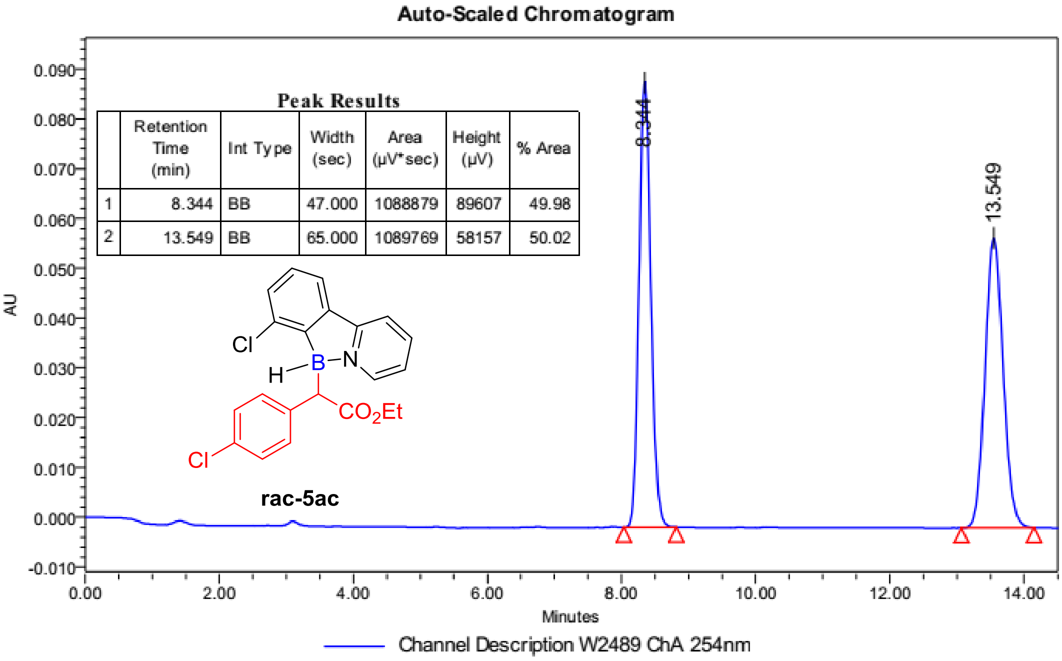

	Retention Time (min)	Int Type	Width (sec)	Area (µV*sec)	Height (µV)	% Area
1	8.344	BB	47.000	1088879	89607	49.98
2	13.549	BB	65.000	1089769	58157	50.02

Supplementary Figure 400. HPLC of compound rac-5ac

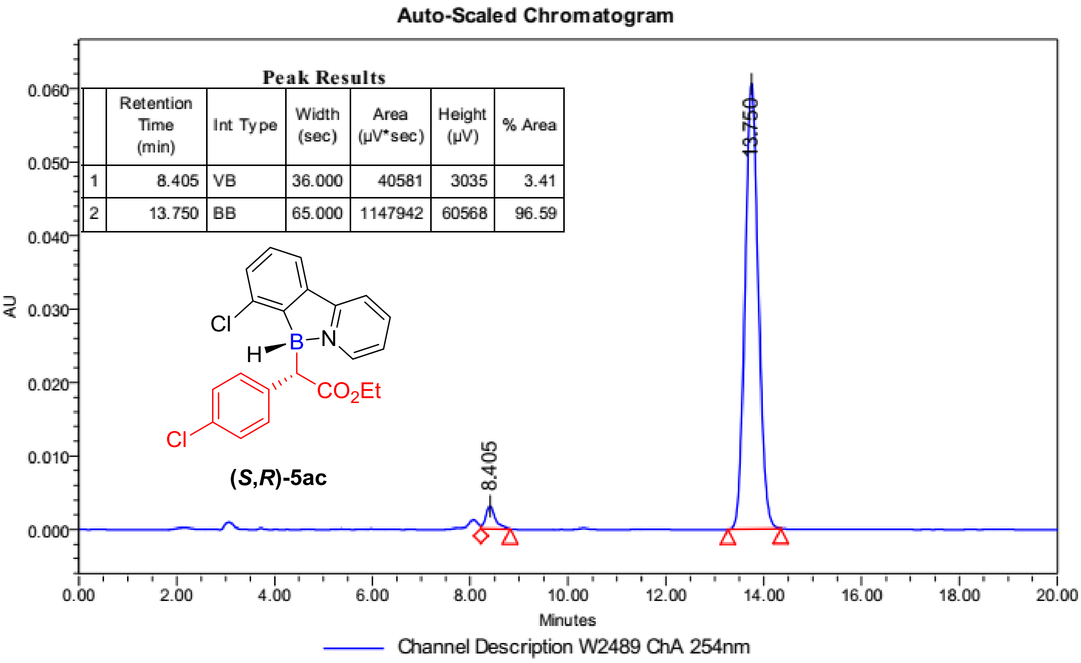

	Retention Time (min)	Int Type	Width (sec)	Area (µV*sec)	Height (µV)	% Area
1	8.405	VB	36.000	40581	3035	3.41
2	13.750	BB	65.000	1147942	60568	96.59

Supplementary Figure 401. HPLC of compound (S,R)-5ac

ethyl(*S*)-2-(4-bromophenyl)-2-(7-chloro-6H-5λ[4]-benzo[3,4][1,2]azaborolo[1,5-a]pyridin-6-yl)acetate (5ad)

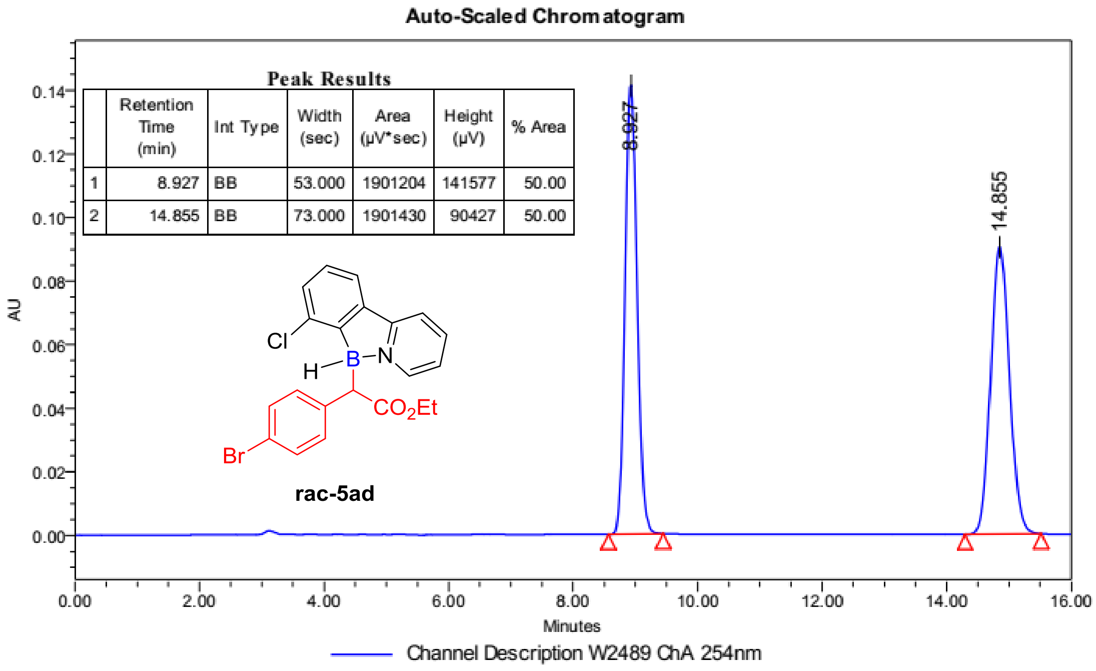

	Retention Time (min)	Int Type	Width (sec)	Area (µV*sec)	Height (µV)	% Area
1	8.927	BB	53.000	1901204	141577	50.00
2	14.855	BB	73.000	1901430	90427	50.00

Supplementary Figure 402. HPLC of compound rac-5ad

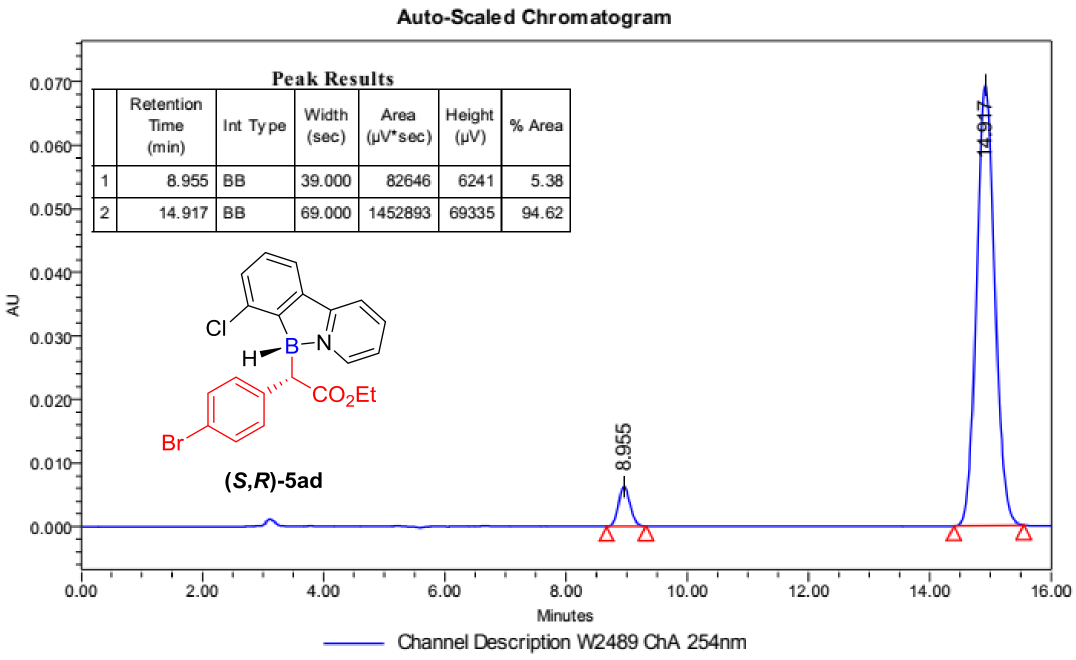

	Retention Time (min)	Int Type	Width (sec)	Area (µV*sec)	Height (µV)	% Area
1	8.955	BB	39.000	82646	6241	5.38
2	14.917	BB	69.000	1452893	69335	94.62

Supplementary Figure 403. HPLC of compound (S,R)-5ad

ethyl(*S*)-2-(7-chloro-6H-5λ[4]-benzo[3,4][1,2]azaborolo[1,5-a]pyridin-6-yl)-2-(naphthalen-2-yl)acetate(5ae)

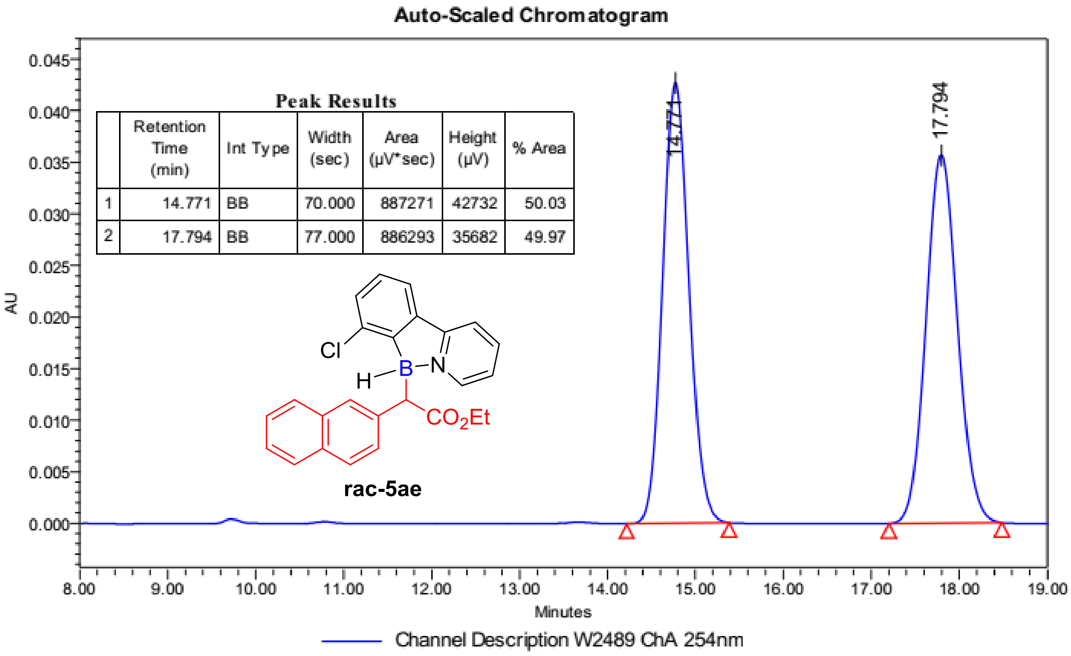

Supplementary Figure 404. HPLC of compound rac-5ae

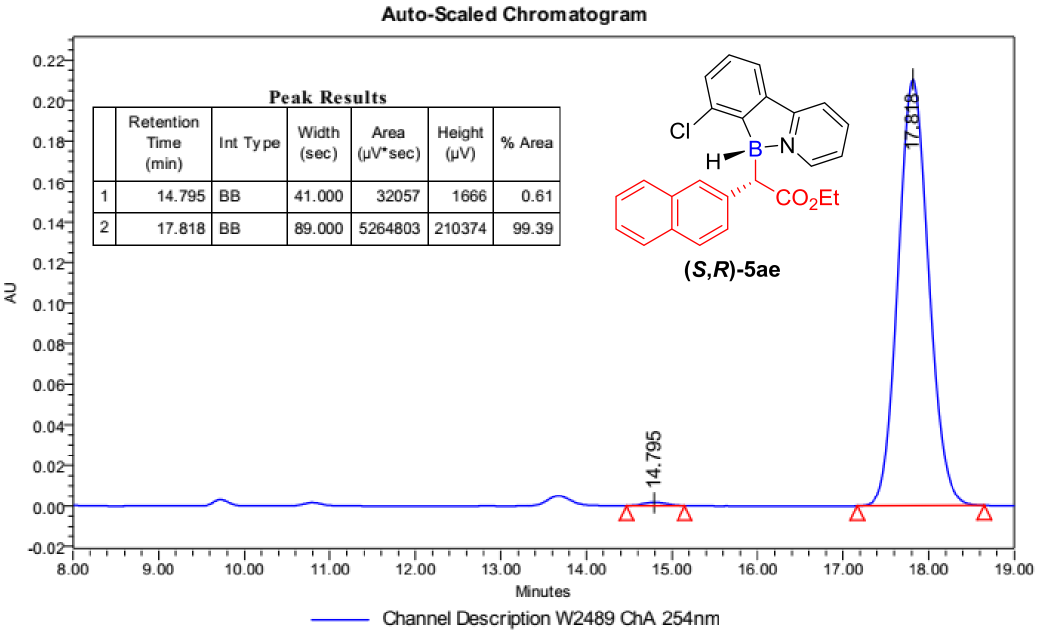

Supplementary Figure 405. HPLC of compound (S,R)-5ae

ethyl(S)-2-(7-chloro-6-(2-ethoxy-2-oxoethyl)-6H-5λ[4],6l4-benzo[3,4][1,2]azaborolo[1,5-a]pyridin-6-yl)-2-phenylacetate (6)

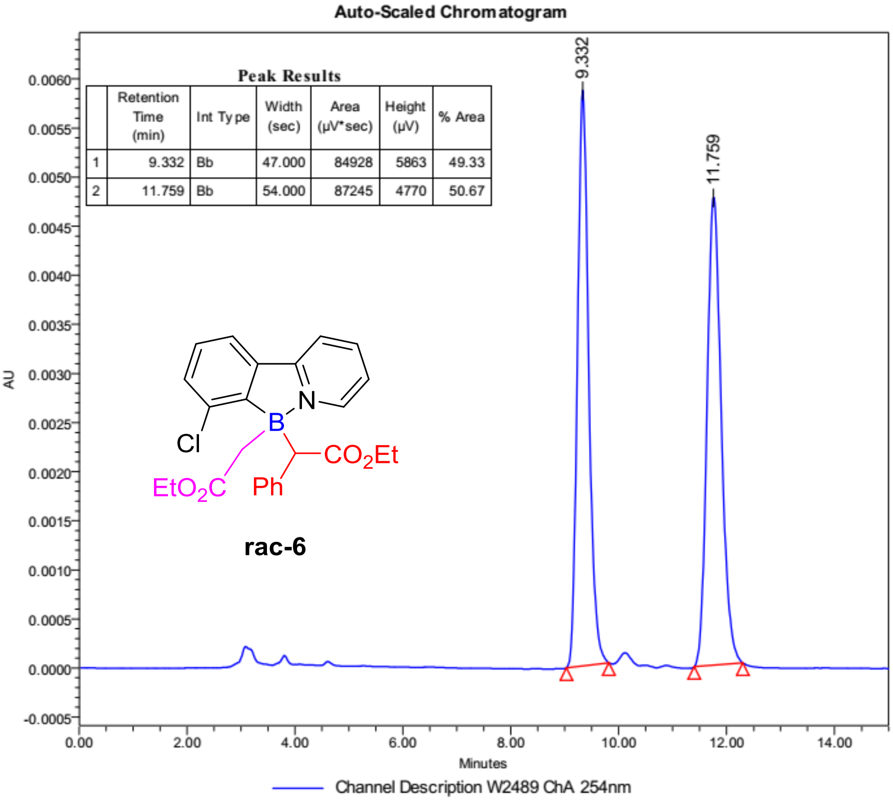

Supplementary Figure 406. HPLC of compound rac-6

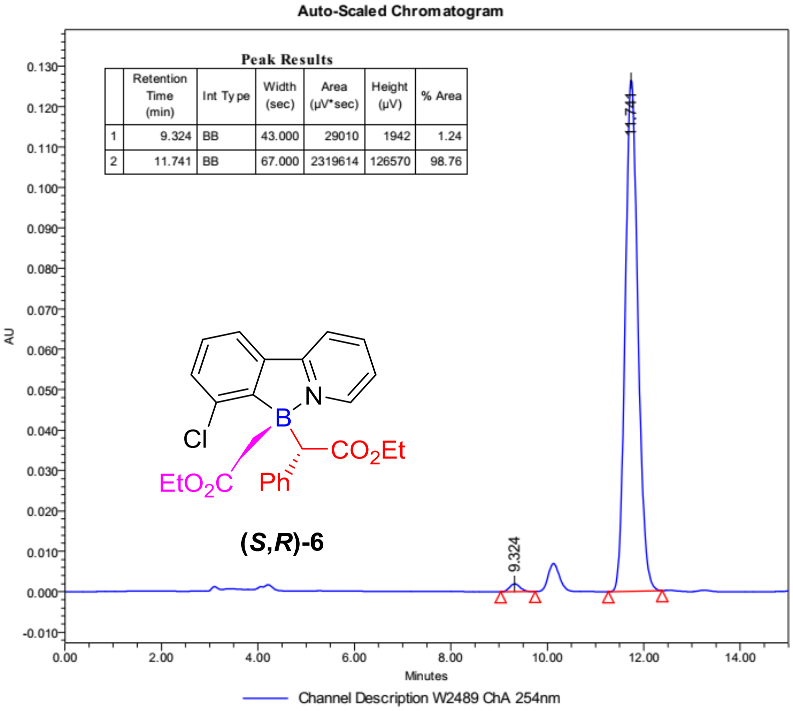

Supplementary Figure 407. HPLC of compound (S,R)-6

(S)-2-(7-chloro-6H-5λ[4]-benzo[3,4][1,2]azaborolo[1,5-a]pyridin-6-yl)-2-phenylethan-1-ol (7)

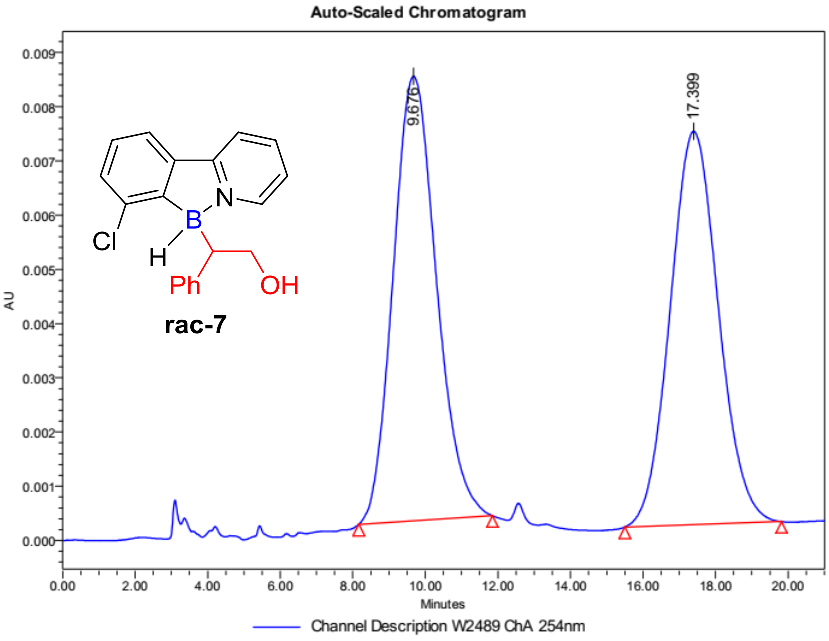

Peak Results

	Retention Time (min)	Int Type	Width (sec)	Area (μV*sec)	Height (μV)	% Area
1	9.676	bb	221.000	680838	8204	50.39
2	17.399	bb	259.000	670250	7257	49.61

Supplementary Figure 408. HPLC of compound rac-7

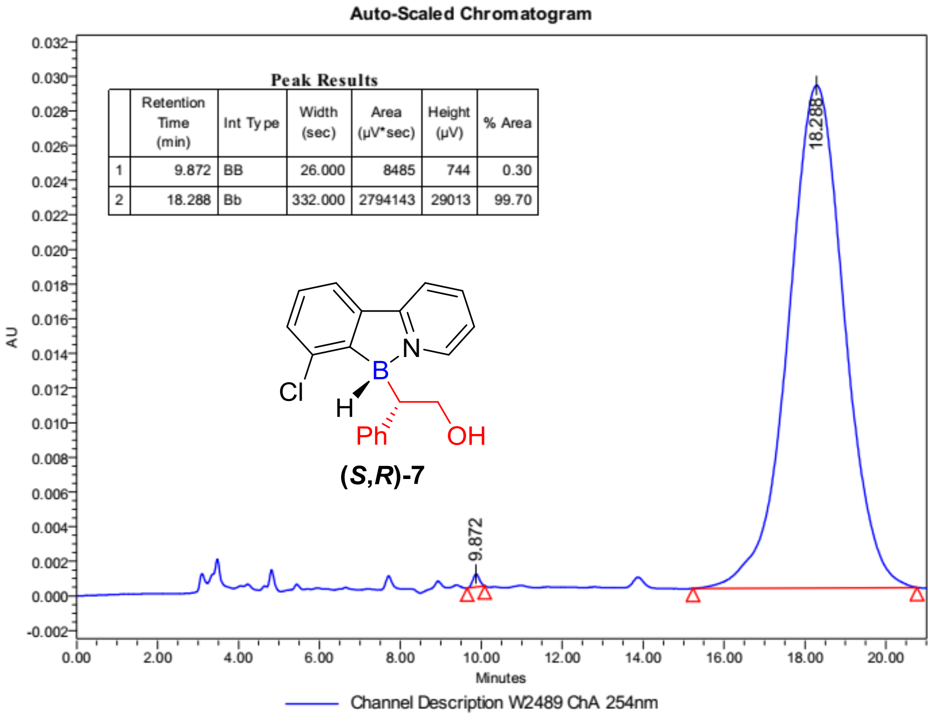

Peak Results

	Retention Time (min)	Int Type	Width (sec)	Area (μV*sec)	Height (μV)	% Area
1	9.872	BB	26.000	8485	744	0.30
2	18.288	Bb	332.000	2794143	29013	99.70

Supplementary Figure 409. HPLC of compound (S,R)-7

ethyl(S)-2-phenyl-2-(7-phenyl-6H-5λ[4]-benzo[3,4][1,2]azaborolo[1,5-a]pyridin-6-yl)acetate(8)

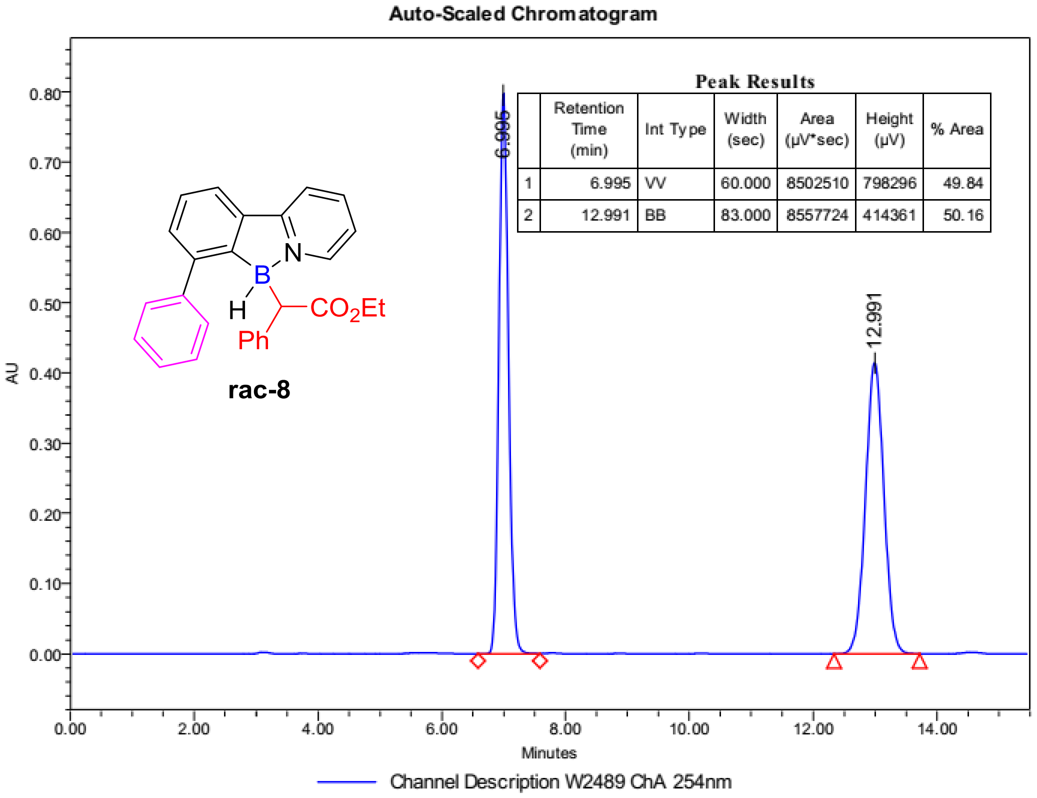

	Retention Time (min)	Int Type	Width (sec)	Area (µV*sec)	Height (µV)	% Area
1	6.995	VV	60.000	8502510	798296	49.84
2	12.991	BB	83.000	8557724	414361	50.16

Supplementary Figure 410. HPLC of compound rac-8

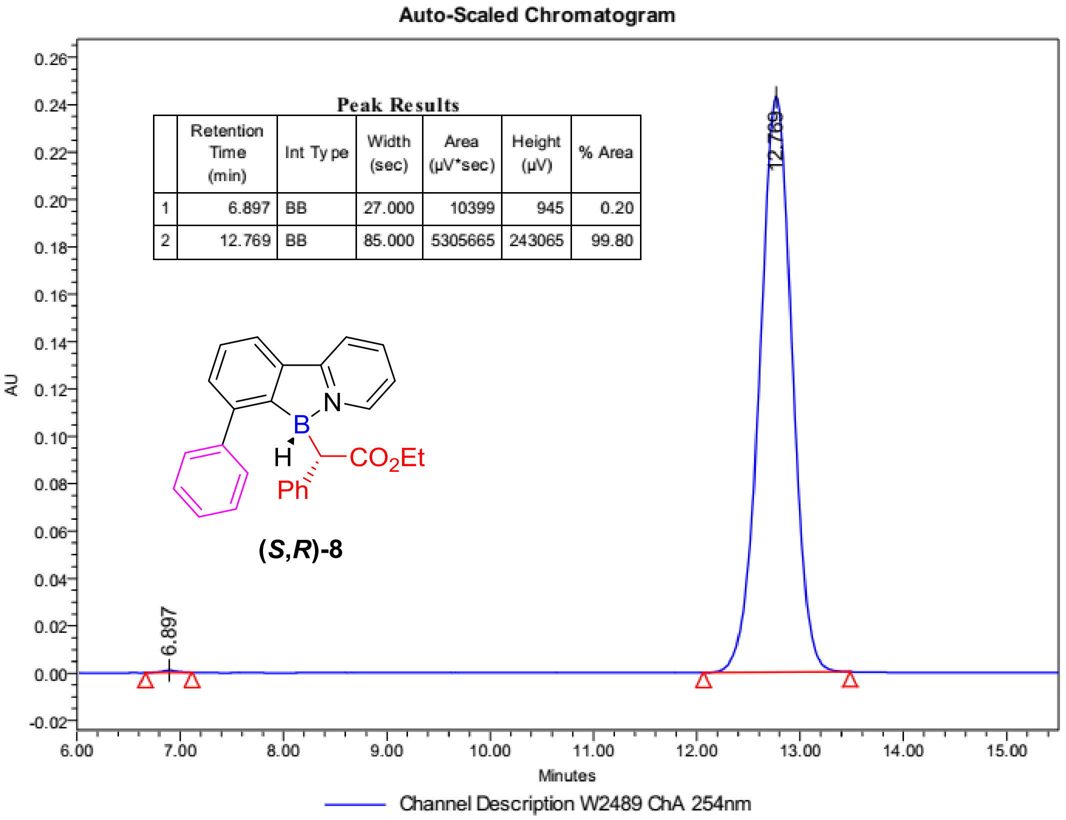

	Retention Time (min)	Int Type	Width (sec)	Area (µV*sec)	Height (µV)	% Area
1	6.897	BB	27.000	10399	945	0.20
2	12.769	BB	85.000	5305665	243065	99.80

Supplementary Figure 411. HPLC of compound (S,R)-8

5. Supplementary References

[1] N. Ishida, T. Moriya, T. Goya, M. Murakami, *J. Org. Chem.* **2010**, *75*, 8709-8712.

[2] W. Liu, J. Twilton, B. Wei, M. Lee, M. N. Hopkins, J. Bacsa, S. S. Stahl, H. M. L. Davies, *ACS Catalysis* **2021,** *11*, 2676-2683.

[3] Q. Wang, A. Liu, Y. Wang, C. Ni, J. Hu, *Org. Lett.* **2021,** *23*, 6919-6924.

[4] É. Lévesque, S. T. Laporte, A. B. Charette, *Angew. Chem. Int. Ed.* **2017,** *56*, 837-841.

[5] A. Levy, S. Cohen, S. Pogodin, I. Agranat, *Struct. Chem.* **2015,** *26*, 1565-1584.

[6] G. M. Green, N. P. Peet, W. A. Metz, *J. Org. Chem.* **2001,** *66*, 2509-2511.

[7] H. Keipour, T. Ollevier, *Org. Lett.* **2017**, *19*, 5736-5739.

[8] P. Gu, Y. Su, X.-P. Wu, J. Sun, W. Liu, P. Xue, R. Li, *Org. Lett*. **2012**, *14*, 2246-2249.

[9] H. Guo, S. Zhang, X. Yu, X. Feng, Y. Yamamoto, M. Bao, *ACS Catal.* **2021**, *11*, 10789-10795.

[10] X.-Q. Hu, *J*.-B. Han, C.-P. Zhang, *Eur. J. Org. Chem.* **2017**, *2017*, 324-331.

[11] R. Panish, R. Selvaraj, *J*. M. Fox, *Org. Lett.* **2015**, *17*, 3978-3981.

[12] L. Lu, C. Chen, H. *J*iang, B. Yin, *J. Org. Chem.* **2018**, *83*, 14385-14395.

[13] M. J. Frisch, G. W. T., H. B. Schlegel, G. E. Scuseria, ; M. A. Robb, J. R. C., G. Scalmani, V. Barone, ; G. A. Petersson, H. N., X. Li, M. Caricato, A. V. Marenich, ; J. Bloino, B. G. J., R. Gomperts, B. Mennucci, H. P. Hratchian, ; J. V. Ortiz, A. F. I., J. L. Sonnenberg, D. Williams-Young, ; F. Ding, F. L., F. Egidi, J. Goings, B. Peng, A. Petrone, ; T. Henderson, D. R., V. G. Zakrzewski, J. Gao, N. Rega, ; G. Zheng, W. L., M. Hada, M. Ehara, K. Toyota, R. Fukuda, ; J. Hasegawa, M. I., T. Nakajima, Y. Honda, O. Kitao, H. Nakai, ; T. Vreven, K. T., J. A. Montgomery, Jr., J. E. Peralta, ; F. Ogliaro, M. J. B., J. J. Heyd, E. N. Brothers, K. N. Kudin, ; V. N. Staroverov, T. A. K., R. Kobayashi, J. Normand, ; K. Raghavachari, A. P. R., J. C. Burant, S. S. Iyengar, ; J. Tomasi, M. C., J. M. Millam, M. Klene, C. Adamo, R. Cammi, ; J. W. Ochterski, R. L. M., K. Morokuma, O. Farkas, ; J. B. Foresman, a. D. J. F., Gaussian, Inc., Wallingford CT, 2016., *Gaussian 16, Revision A.03*, Gaussian, Inc., Wallingford CT, 2016.

[14] A. D. Becke, *J. Chem. Phys.* **1993,** *98*, 5648-5652.

[15] A. V. Marenich,; C. J. Cramer,; D. G. Truhlar, *J. Phys. Chem. B* **2009,** *113*, 6378-6396.

[16] F. Weigend,; R. Ahlrichs, *Phys. Chem. Chem. Phys.* **2005,** *7*, 3297-3305.

[17] Y. Zhao, D. G. Truhlar, *Theor. Chem. Acc.* **2008,** *120*, 215-241.

[18] Fukui, K., The path of chemical reactions-the IRC approach. *Acc. Chem. Res.* **1981,** *14*, 363-368.

[19] Y. Zhao,; D. G. Truhlar, *Theor. Chem. Acc.* **2008,** *120* , 215-241.

[20] Legault, C. Y. CYLview, version 1.0b; Université de Sherbrooke: Quebec, Canada, 2009; http://www.cylview.org.
