# Supplementary material for: Construction of boron-stereogenic compounds via enantioselective Cu-catalyzed desymmetric B–H bond insertion reaction
Source: Nat Commun. 2022 May 12;13:2624. doi: 10.1038/s41467-022-30287-7 (PMC9098526; doi:10.1038/s41467-022-30287-7)
Supplement: Supplementary file 3 — Description of Additional Supplementary Files [file 41467_2022_30287_MOESM3_ESM.pdf]

Description of Additional Supplementary Files

File name: Supplementary Data 1

Description: Energies (in Hartree) and Cartesian Coordinates of DFT-Computed Structures.
